# Selective Reduction of Esters to Access Aldehydes Using Fiddler Crab-Type Boranes

**DOI:** 10.1021/jacs.4c14596

**Published:** 2024-12-26

**Authors:** Ádám Dudás, Ádám Gyömöre, Bence Balázs Mészáros, Stefánia Gondár, Renáta Adamik, Dániel Fegyverneki, Dávid Papp, Konrad Bernhard Otte, Sergio Ayala, János Daru, József Répási, Tibor Soós

**Affiliations:** † Organocatalysis Research Group, Institute of Organic Chemistry, HUN-REN Research Centre for Natural Sciences, Magyar tudósok körútja 2, Budapest H-1117, Hungary; ‡ Hevesy György PhD School of Chemistry, Eötvös Loránd University, Pázmány Péter sétány 1/A, Budapest H-1117, Hungary; § MTA-ELTE Lendület Ion Mobility Mass Spectrometry Research Group, Eötvös Loránd University, Pázmány Péter sétány 1/A, Budapest H-1117, Hungary; ∥ Provivi, Inc., Santa Monica, California 90404, United States; ⊥ Department of Organic Chemistry, Eötvös Loránd University, Pázmány Péter sétány 1/A, Budapest H-1117, Hungary; # Aldexchem Ltd., Érd H-2030, Hungary

## Abstract

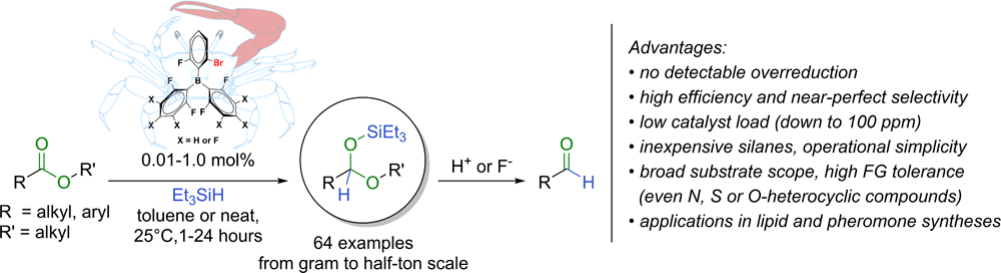

The partial reduction of esters to aldehydes is a fundamentally important transformation for the synthesis of numerous fine chemicals and consumer goods. However, despite the many efforts, limitations have persisted, such as competing overreduction, low reproducibility, use of exigent reaction conditions and hazardous chemicals. Here, we report a novel catalyst family with a unique steric design which promotes the catalytic partial reduction of esters with unprecedented, near-perfect selectivity and efficiency. This metal-free catalytic method is ready to be placed at the disposal of chemists to provide valuable aldehyde intermediates and products and shows promise for streamlining synthetic methods in academic and industrial settings.

## Introduction

**Figure 1:**
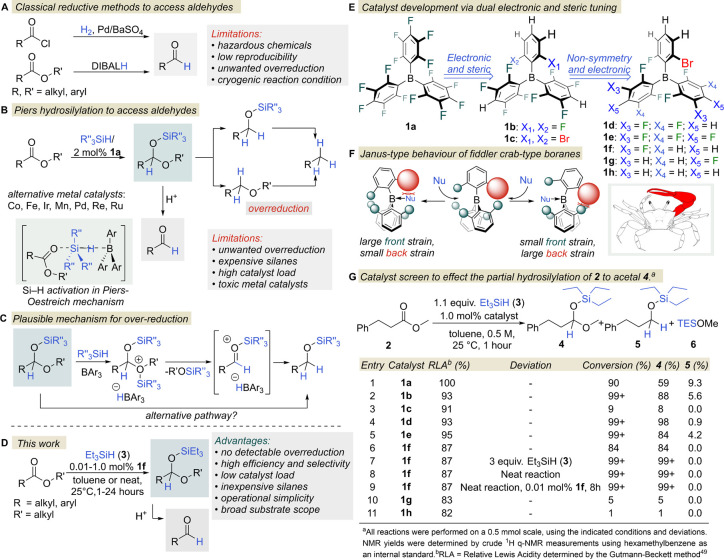
Methods to access aldehydes and catalyst design for near-perfect selectivity in partial ester reduction.

Aldehydes are among the most important building blocks in organic chemistry and have been applied in many areas, including the pharmaceutical, polymer, and fine chemical industries.^1−6^ In addition, synthetic and naturally occurring aldehydes are widely used as flavor and fragrance ingredients and as components of many moth sex pheromones and specific attractants for mosquitos.^7−9^ Consequently, the efficient and selective formation of the aldehyde functionalities from inexpensive feedstock materials has been a long-standing academic and industrial pursuit. In addition to the hydroformylation of alkenes,^10,11^ the most common method for accessing aldehydes is the redox manipulation of alcohols and carboxylic acid derivatives. With respect to alcohol oxidation, the advent of new reagents and catalysts has largely solved the challenges of selectivity and practicality.^12−18^ Thus, several robust methods, such as the stoichiometric Swern and Dess-Martin oxidations, as well as a variety of Au, Pd, Fe, Cu, and oxoammonium catalytic oxidative processes have been described. In contrast, the partial reduction of carboxylic acid derivatives to aldehydes is a more challenging task because aldehydes tend to readily overreduce to alcohols. Therefore, the most common synthetic route for accessing many aldehydes is often the full reduction of carboxylic acid derivatives to alcohols followed by an oxidation step, despite the poor redox economy and the waste generated by this bypass approach. Nevertheless, there are a few methods in the synthetic toolbox that have been applied to the partial reduction of carboxylic acid derivatives (Figure 1A).^19^ The Rosenmund reduction^20^ is a venerable hydrogenation process in which acyl chlorides are reduced to their corresponding aldehydes using poisoned heterogeneous Pd catalysts. However, various problems with catalyst recovery and reproducibility, as well as the obligatory use of hazardous acyl chlorides, limit the feasibility of the Rosenmund reaction in modern chemistry. An important milestone was the discovery that the partial reduction of esters, as a cheap and readily available feedstock, could also be achieved under cryogenic conditions using the bulky diisobutylaluminum hydride (DIBALH).^21^ However, these experiments are often hindered by several problems, including reproducibility, frequent overreduction and the high flammability of DIBALH, which have generated a notorious reputation for this reagent and precipitated widespread concern about its use in industry.^22,23^ In addition, modified Red-Al reducing agents have also been investigated with some success (mainly among aromatic esters), although the examples outlined seem to be exceptions rather than indicative of the general applicability of this reductant.^24,25^ Because of the significant limitations of these previous methods, chemists have continually pursued additional approaches to this important transformation. As a result, catalytic hydrosilylation has emerged as a promising option (Figure 1B). In 1996, Piers and co-workers discovered that using B(C_6_F_5_)_3_ (**1a**) as a catalyst, the hydrosilylation of esters could be interrupted in a way that silyl acetals were the major products formed (60–70%) and the respective aldehydes were isolated after hydrolytic workup.^26^ It was also shown that borane catalyst **1a** promotes hydrosilylation via a counterintuitive S_N_2-like Si–H activation mechanism (Figure 1B).^27−29^ Although the significant overreduction could not be avoided, Piers’ work triggered further improvements, and several metal-catalyzed hydrosilylations have been developed^19,20^ among which the Ir-catalyzed version of Brookhart stands out.^30^ The tangible, practical advantage of these efforts is that, compared to DIBALH, silanes are less hazardous reagents, do not require exigent reaction conditions and are more compatible with many functional groups. However, these catalytic hydrosilylations still suffer from many practical and economical restrictions that impede their widespread use, especially in industry (Figure 1B). These disadvantages include significant overreduction, limited substrate scope, inconvenient reaction conditions for catalyst activation, high catalyst loads, the use of expensive silane reagents and the application of toxic and expensive catalysts.

Despite the current restrictions on the partial hydrosilylation of esters, inspiration for major improvements has been derived; in particular, pioneering studies of Piers’ borane-promoted hydrosilylation^26,27^ have influenced our efforts. In addition to their cost and environmental advantages, metal-free borane catalysts offer the prospect of a mechanistic distinction that may overcome some of the limitations of metal-catalyzed procedures. Herein, we report the development of highly efficient borane catalysts with special steric, fiddler crab-type design that can promote the partial hydrosilylation of esters to aldehyde acetals with near-perfect selectivity, using commercially available and affordable silanes (Figure 1D). This catalytic process offers advantages in efficiency, safety, operational simplicity, and robustness over currently employed reductive methods and is amenable to scale-up for large-scale production.

## Results and Discussion

To significantly further the scope and practicality of the borane **1a** catalyzed partial hydrosilylation of esters (Figure 1B), we aimed to develop new borane catalysts, an endeavor that posed several distinct challenges. First, we were confronted with the dilemma of increased moisture sensitivity and substrate and/or product inhibition due to the high oxophilicity of the boron center. Due to these properties, borane catalysts inherently suffer from limited practicality and substrate scope as well as low catalytic activity, which necessitates the application of relatively high catalyst loads. Furthermore, a particular challenge was to ensure that the process ceased completely in the acetal phase, i.e., that the borane catalyst did not promote further reduction of the acetal product via subsequent Si–H activation. To mitigate or eliminate the overreduction problem, the fundamental question is whether only increasing the kinetic barrier(s) for the subsequent reductive step (via the putative oxocarbenium intermediate) would be an appropriate solution, or whether an unknown mechanism is responsible for the observed overreduction (Figure 1C). Due to this quandary regarding the mechanistic provenance of the overreduction, and the lack of a blueprint for designing highly efficient and selective borane-based catalysts to partially reduce esters, we explored a dual steric and electronic fine-tuning strategy for catalyst development (Figure 1E). Our and others’ previous work on the development of borane-based frustrated Lewis pair (FLP) hydrogenation catalysts indicated that electronic tuning alone provided weaker, even water-tolerant, Lewis acids with higher functional group tolerance.^31−41^ In addition, steric effects were an important additional tool for further improving the catalytic performance of boranes. We found that the front and back strains^42,43^ not only modulate Lewis acidity but also inhibit the complexation with small Lewis bases and secure the formation of kinetically labile, structurally spring-loaded borohydride intermediates during hydrogenation, a design concept that could be usefully extended to hydrosilylation reactions (Figure 1F).^40,41^

### Catalyst Design and Reaction Optimization

Our studies started with the hydrosilylation of methyl hydrocinnamate (**2**) as a representative substrate (Figure 1G). As expected, the previously used borane **1a** promoted the hydrosilylation of **2** with triethylsilane (TESH, **3**) to generate the desired product **4** (Figure 1G, entry 1). However, extensive overreduction occurred, in which the silyl ether **5** was formed to the greatest extent with the concomitant formation of triethylmethoxysilane (TESOMe, **6**). The less Lewis acidic but similarly crowded heteroleptic fluorinated borane **1b** (Figure 1E) was a more competent, but still not a sufficiently selective catalyst (Figure 1G, entry 2). Further optimization efforts were then aimed at minimizing overreduction, so the highly crowded catalyst **1c** was tested with enhanced front- and back-strains. Disappointingly, the bulky bromine substituents significantly lowered the catalytic activity (Figure 1G, entry 3). We reasoned that the Si–H activation might be blocked by the enhanced front-strain in **1c**. Therefore, we presumed that removing one bromine substituent might alleviate the steric repulsion during Si–H activation (lower front strain) and provide a kinetically more labile hydride adduct due to the large back strain (Figure 1F). This hypothesis gained credence as fiddler crab-type borane **1d** exhibited a marked improvement in activity and selectivity, with complete conversion and a crude product containing only less than 1 mol % of overreduced product **5** (Figure 1G, entry 4). Following the success of this special, nonsymmetric steric tuning, the effect of electronic fine-tuning was investigated by modifying the number of fluorine atoms in the *meta-* and *para-*positions of the nonbrominated aryl rings.^44^ The more Lewis acidic **1e**, with highly electron-withdrawing pentafluoro rings, showed high reactivity, but its selectivity toward acetal formation was diminished (Figure 1G, entry 5). However, selectivity was surprisingly improved with catalyst **1f**, which has a lower Lewis acidity, as the desired acetal **4** was exclusively formed without any overreduced side products (Figure 1G, entry 6). However, due to the slightly lower catalytic activity of **1f**, this near-perfect selective partial hydrosilylation catalyst reached 84% conversion in 1 h. The absence of competing overreduction side reaction during the catalytic ester-to-acetal conversion was unprecedented in the field of aldehyde synthesis and inspired further study. First, we applied 3 equiv of the TESH (**3**) reducing agent to facilitate the reaction. Gratifyingly, full conversion was achieved within 1 h, and the exclusive selectivity of the process was maintained despite the excess amount of the reducing agent **3** (Figure 1G, entry 7). Then, as TESH (**3**) is a liquid, the reaction was simply carried out without solvent. Remarkably, the hydrosilylation reaction retained its “click chemistry” characteristics,^45^ with full conversion and exclusive selectivity for acetal formation (Figure 1G, entry 8). We then performed the reaction with only 0.01 mol % catalyst **1f**. To our delight, full conversion was achieved within 8 h without any deterioration in the selectivity of the process, demonstrating the high potential of this catalyst (Figure 1G, entry 9). To further probe the effect of electronic fine-tuning on catalyst activity, we conducted reactions with catalysts **1g** and **1h**; however, poor conversions were observed under the reaction conditions applied (Figure 1G, entries 10, 11), most likely due to their lower capacity for Si–H activation. Although these Lewis acidic catalysts do not appear to be suitable promoters for this substrate, they may be useful for enabling bifunctional Si–H activation and reduction when more Lewis basic ester substrates (e.g., lactones) are used. Finally, the Lewis acidity of boranes **1a**–**h** was evaluated using the Gutmann-Beckett method, yet no correlation was identified between the experimentally obtained relative Lewis acidities and the catalytic performance (Figure 1G), only within the **1d**–**h** series. The reason for this contradiction seems to be the steric factor as the introduction of a bulky substituent bromine in the *ortho*-positions can profoundly reshape the energy landscape of the dative adducts (e.g., with Et_3_P=O) through the counteracting change in steric effects.

### Exploring the Substrate Scope

**Figure 2:**
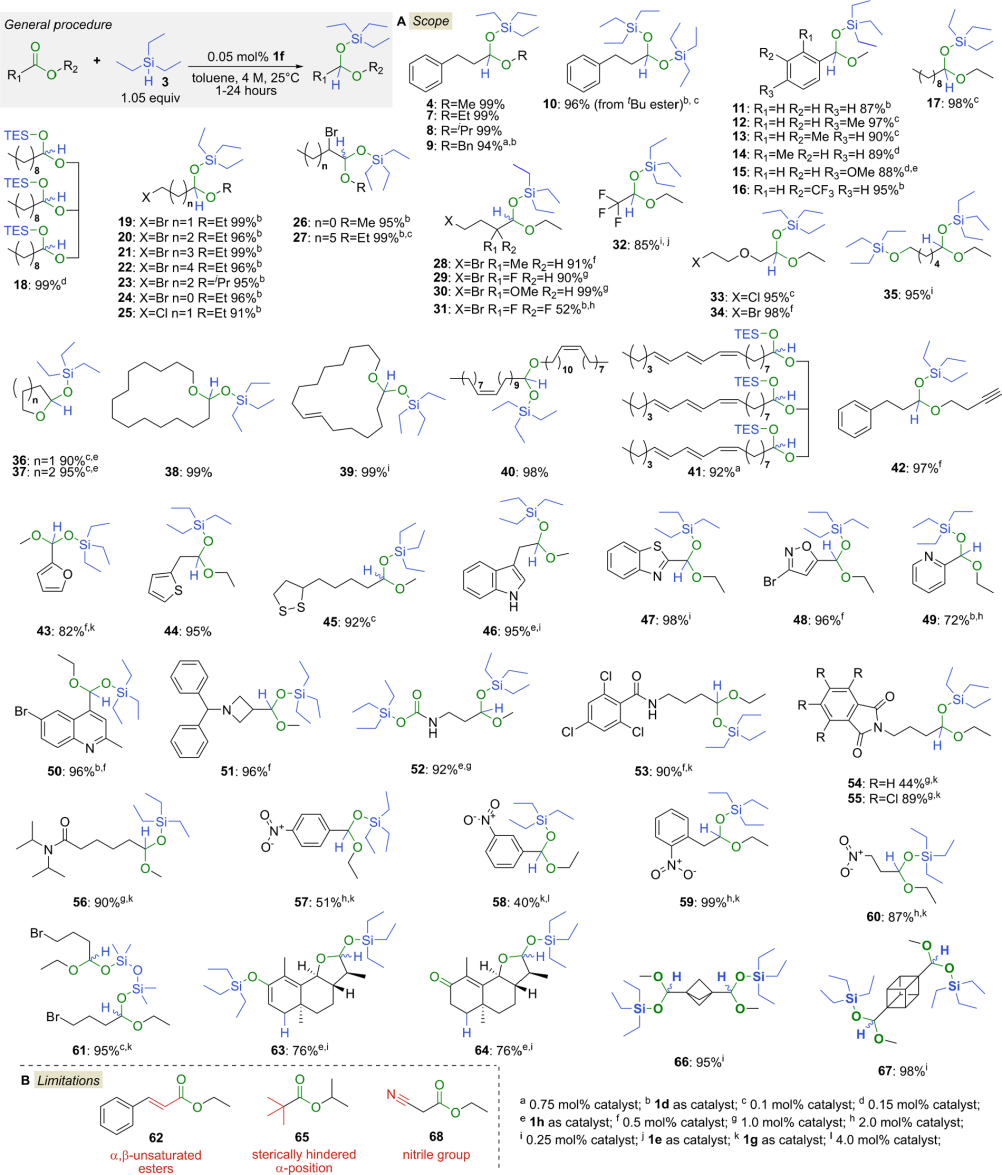
Scope and limitations of the near-perfect selective ester hydrosilylation.

With an optimized set of conditions in hand, the scope of esters was then evaluated (Figure 2A). During this study, a broad collection of esters and lactones with different Lewis basicities was investigated, which sometimes necessitated the use of **1d,e** and **1g,h** boranes instead of catalyst **1f** to maximize yield and selectivity. First, the effect of the ester’s alkoxy component on the partial reduction was investigated; thus, we subjected various hydrocinnamates decorated with alkyl groups having different steric and electronic properties. In accordance with the previous findings, the primary, secondary, and benzylic alcohol derivates afforded the desired **4**, **7**–**9** acetals with high efficiency and selectivity, the only limitation was *t*-butyl hydrocinnamate, as the first equivalent of the silane removed the *t*-Bu group. Nevertheless, using 2 equiv of TESH (**3**), the appropriate bis-silyl acetal **10** was obtained in high yield and selectivity. Electron-rich and electron-deficient aromatic esters with different substitution patterns also served as viable substrates for this partial reduction, and although full conversion and selectivity were observed by NMR, the volatility of aromatic acetals **11**–**16** resulted in slightly lower isolated yields. This reduction could be performed on aliphatic substrates with high selectivity and yields (e.g., **17**). Notably, the borane catalyst **1f** could directly convert a triglyceride to the acetal product **18** with near-perfect conversion and selectivity. Next, the tolerance of the method to sensitive functionalities was investigated. Various esters with halogenide (F, Cl, Br) and alkoxy substituents were efficiently and selectively reduced to the corresponding acetals **19**–**34**. Importantly, the catalytic rate was inversely proportional to the steric parameters of the substrates. Thus, for more sterically hindered substrates, higher catalyst loads were used to achieve full conversion within a reasonable time (e.g., **27**–**31**). Experiments have shown that a maximum of two substituents (alkyl/halogen/alkoxy) at the α position of the ester group can be accommodated during the reduction, unless fluorine(s) are included, in which case the substrate can be trisubstituted at the α position (**31**, **32**). Even a substrate with a free hydroxy group could be directly reduced; however, in that case, two equivalents of TESH (**3**) must be used to form the alkoxy-protected acetal **35**. Selected examples of lactones^46^ with an *E* or *Z* ester conformation were also subjected to catalytic hydrosilylation. Gratifyingly, small- and large-ring lactones underwent smooth reduction with high selectivity (**36**–**38**). Another compelling area of application may be the reductive transformation of natural products, especially vegetable oils with olefinic bonds. We found that catalyst **1f** tolerated the presence of olefinic groups, allowing various applications, such as the efficient reduction of ambrettolide to silyl lactol **39**. Similarly, readily available and inexpensive jojoba oil and tung oil were transformed to acetal derivatives **40** and **41**. In addition to olefinic bonds, acetylenic bonds also endured during the reduction process, as exemplified by the formation of acetylenic acetal **42**. To further evaluate the limits of our catalytic approach, we reduced various aromatic and saturated heterocyclic esters. Esters with either furan or thiophene rings were easily transformed to the desired **43** and **44** acetals. Notably, catalyst **1f** was competent in the partial reduction of a lipoic acid-derived ester to yield **45**, while neither substrate/product inhibition, nor reduction of the S–S bond occurred. With ample precedent in hand, the reduction of nitrogen-containing heterocyclic esters was then explored to demonstrate the increased functional group tolerance of this methodology and access numerous heterocyclic motifs used in medicinal chemistry developments. Importantly, these structures often present an inherent challenge in FLP chemistry because their Lewis basic sites can inhibit the hard-type Lewis acid catalysts. Gratifyingly, various nitrogen-containing heterocyclic compounds were found to be suitable substrates for this reduction, and the reduction of the heteroaromatic rings was not observed. First, an indolic ester was converted to **46** using the less Lewis acidic **1h** catalyst, while benzothiazole and isoxazole derivatives were smoothly reduced using **1f** (**47**, **48**), all in high yields and selectivities. Then, to our delight, pyridine (**49**) and quinoline (**50**) containing acetals could be accessed using the more Lewis acidic **1d** catalyst, albeit using higher catalyst loadings. In addition, an ester derivative with tertiary amine functionality could be catalytically reduced, as exemplified by the formation of acetal **51** incorporating a benzhydryl protected azetidine. These results gave further confidence in the reductive method developed and propelled further studies toward other amine and nitro derivatives. Accordingly, amide derivatives have been targeted as the next substrates to be explored, due to their extensive use in synthetic chemistry. To gauge their applicability, the influence of the electron density in acyl protecting groups was investigated. As shown in Figure 2, various primary and secondary amide derivatives were also suitable substrates when less Lewis acidic catalysts **1g** and **1h** were applied, providing functional handles for further downstream condensation chemistry.^47^ Importantly, the Boc-protected amino ester engaged in a deprotection step first, in accordance with the above findings with **10**, affording bis-silyl acetal **52**. As expected, 2,4,6-trichlorobenzamide, having an electron withdrawing acyl moiety, was found to be a suitable protecting group leading to acetal **53** in good yield. Evaluation of the phthalimide protecting groups confirmed a preference for the less Lewis basic protecting group in the catalytic process; phthalimide acetal **54** was accessed in only 44% yield, however, tetrachlorophthalimide acetal **55** was generated in 89% yield. An attempt to alleviate the restriction among electron-rich amide substrates has shown that steric effect can be exploited, so the hindered tertiary amide containing substrates could be reduced using catalyst **1g** with high efficiency (**56**) and without the reduction of the amide group. Similarly, the method also tolerated the presence of the nitro group if the less Lewis acidic **1g** catalyst was used. Nitrobenzoates afforded only mediocre yields (**57**, **58**), probably due to lower basicity of their ester groups. On the other hand, aliphatic nitro-acetals **59** and **60** could be obtained in high yields using the same catalyst. Finally, we examined whether this methodology could accommodate other easily available and inexpensive silanes. Gratifyingly, we found that using tetramethyl-disiloxane (TMDS)^48^ and the less Lewis acidic catalyst **1g** afforded the desired bisacetal **61** in high yield and selectivity, further highlighting the utility and generality of this distinct catalyst family.

Although this catalytic method has a broad scope in its current form, it is not without limitations (Figure 2B). For example, the product arising from the reduction of the conjugated unsaturated ester **62** is not the desired acetal product formed in a 1,2-addition but rather a ketene silyl acetal formed in a 1,4-addition.^49^ Preliminary study also pointed to the preferred 1,4-addition of conjugated carbonyls in this reaction. For instance, the reduction of α-santonin using two equivalents of TESH (**3**) resulted in the concomitant reduction of its lactone functionality and the regioselective 1,4-reduction of its α,β-unsaturated carbonyl moiety. By varying the workup procedure, either the silyl-ether containing **63** (column chromatography on basic alumina), or the carbonyl compound **64** (column chromatography on silica gel) can be obtained. During exploring the substrate scope, we also found that an increase in steric hindrance around the carbonyl center resulted in a decreased reaction rate. This could even be such an extreme drop-off that no reaction was observed in the case of the alpha trisubstituted pivalate **65**. There are, however, a few glaring exceptions among these types of compounds that are also important in medicinal chemistry. Thus, bis-ester analogues, which are sterically slightly less demanding because of the ring strain, could still be reduced using two equivalents of TESH (**3**), as exemplified by the bicyclo[1.1.1]pentane derivative **66** and cubane derivative **67**.

Another clear limitation is the presence of reducible Lewis basic functionalities that are not sterically demanding, as exemplified by nitrile **68**. In this case, hydrosilylation of the nitrile group is the predominant reaction.

Finally, several additional points^49^ about this method are noteworthy. (i) The developed catalysts can be easily synthesized in the laboratory, even on a 100 g scale, using commercially available and inexpensive starting materials. (ii) The reductions can be performed without reliance on the Schlenk technique. (iii) Water deactivates these catalysts by forming a dative adduct; however, they can be fully regenerated by adding TESH (**3**) to form silanols and siloxanes (TESOH and (TES)_2_O) and H_2_. (iv) Substrates were used as received from commercial vendors without further purification. (v) The utilized reducing agents (TESH and TMDS) are inexpensive and easily available in bulk.

### Synthetic Applications

**Figure 3:**
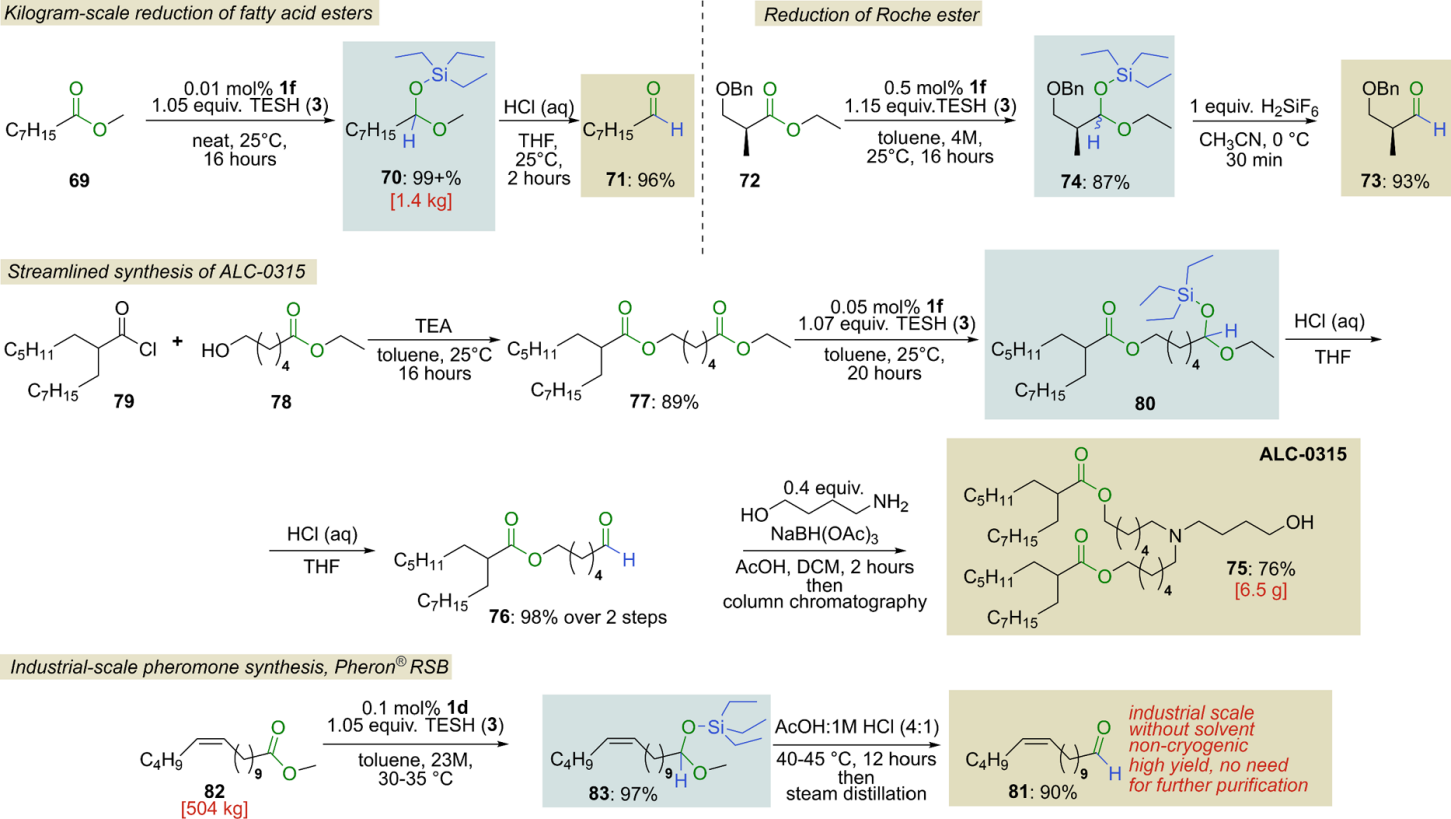
Selected applications of the catalytic hydrosilylation methodology.

To further demonstrate the synthetic potential of this catalytic method, we present four representative examples. First, by evaluating the reduction of methyl octanoate (**69**) in batch, we investigated the scalability of this process (Figure 3). A kilogram-scale reaction was carried out without solvent in a conventional laboratory environment using only 0.01 mol % of catalyst **1f**. As the process is exothermic, the reducing agent **3** was added at a specific rate to maintain the reaction temperature between 30 and 35 °C.^49^ Gratifyingly, full conversion and selectivity were achieved, and after a simple workup, acetal **70** was isolated in 99% yield. The purity of acetal **70** was high enough that it could be transformed to aldehyde **71** without any purification. A further opportunity to challenge the utility of this catalytic method was the reduction of benzylated Roche ester **72**. The Roche ester is a central chiral pool in polyketide natural product synthesis, and its conversion to the corresponding **73** aldehyde is a formidable synthetic challenge.^50,51^ Most importantly, the direct reduction of Roche ester derivatives **72** with DIBALH is not scalable, so a poor-redox economic route is needed, i.e., the full reduction of **72** to alcohol, followed by oxidation. In addition, aldehyde **73** was reported to be unstable during prolonged storage, as rapid racemization of the chiral center occurred even at 0 °C. Therefore, in polyketide synthesis, crude **73** is immediately subjected to aldol reactions. The use of our hydrosilylation method holds a great appeal to overcome previous synthetic hurdles. As shown in Figure 3, catalyst **1f** was a competent catalyst for the partial reduction of the benzylated Roche ester **72** to generate acetal derivative **74** without racemization of the stereocenter. The acetal **74** formed is stable and can be purified by column chromatography, and racemization of the stereogenic center was not observed upon storage (1 year at −20 °C), making it a valuable synthetic precursor for the on-demand formation of **73** using 33% aq. H_2_SiF_6_ reagent. Surprisingly, contrary to previous observations, Roche aldehyde **73** prepared by our methodology showed no signs of racemization even after days of storage at room temperature.

The third example was performed to demonstrate the utility of this methodology in the synthesis of lipids, i.e., the synthesis of ionizable aminolipids used for cellular delivery of mRNA.^52,53^ Due to its high selectivity in the partial reduction of esters, this process can provide rapid and scalable access to the key lipid aldehyde intermediates needed for aminolipid synthesis without the need for cumbersome, time-consuming, and costly purification steps. To illustrate this inherent synthetic potential, we sought to develop a streamlined and efficient route to the ionizable lipid ALC-0315 (**75**) which is the critical component of the lipid matrix of the prophylactic SARS-CoV-2 mRNA vaccine produced by Pfizer/BioNTech.^54^ The scalable synthesis of this lipid has involved several difficulties and was recognized as one of the bottlenecks in the Comirnaty vaccine production campaign.^55,56^ Accordingly, we envisioned a concise synthetic route to the key aldehyde intermediate **76** that exploited not only the high selectivity of catalyst **1f** in ester reduction but also its reduced activity toward sterically crowded ester groups. More specifically, we reasoned that catalyst **1f** could siteselectively reduce the terminal ester functionality in **77** while leaving the internal, more sterically demanding ester functionality intact. Thus, an additional advantage of this alternative route is that it avoids the synthesis of a monoester derivative from a diol, thus eliminating the burden of tedious chromatography. Figure 3 shows the successful implementation of our streamlined synthetic route. Using monovalent methyl 6-hydroxycaproate **78** and acyl chloride **79**, we prepared the diester **77** on a multidecagram scale. We then subjected diester **77** to partial reduction to generate monoacetal **80**. Gratifyingly, catalyst **1f** delivered the desired product **80** on a 50 g scale with excellent selectivity and efficiency; therefore, no purification was needed after hydrolyzing the acetal to the key aldehyde intermediate **76**. Finally, the reductive amination of aldehyde **76** was elicited using freshly prepared NaBH(OAc)_3_. Many conditions were screened to maximize the conversion and selectivity of the reaction; thus, ALC-0315 (**75**) was synthesized with very high purity after a single and simple chromatography and with a significantly improved overall isolated yield.^49^ The final example was performed to demonstrate the potential of our method in pheromone synthesis. Utilizing insect pheromones to disrupt mating is rising as a sustainable alternative to pesticides for pest control because this approach is pest specific and nontoxic; in addition, reports of insect resistance are limited.^57−59^ However, the growth of the insect pheromone market has been limited by the availability of low-cost, robust, and scalable pheromone synthesis routes.^60−62^ In particular, the synthesis of pheromone aldehydes on an industrial scale presents a formidable challenge, as the presence of alcohol impurities in the product (either as a starting material or as an overreduction side product) can be detrimental to the product’s performance in mating disruption.^60,63,64^ Thus, relying on a reductive approach to access these aldehydes, minimizing overreduction to alcohol (e.g., below 2%) is a major burden, as purification is either prohibitively costly or tedious. In this example, the straight-chain lepidopteran pheromone (*Z*)-hexadec-11-en-1-al (**81**), the key pheromone component of yellow and striped stem borers in rice (*Scirpophaga incertulas* and *Chilo suppressalis*) was synthesized from desaturated methyl (*Z*)-hexadec-11-enoate (**82**) using 1.1 equiv. TESH (**3**) as the reducing agent and 0.1 mol % of catalyst **1d**. The translation of our laboratory reduction protocol to an industrial-scale (504 kg) manufacturing was smooth and fast affording mixed acetal **83** in excellent, near-perfect yield.^65^ Then, without further purification, the crude reaction mixture of the mixed silyl acetal **83** was hydrolyzed to generate the desired aldehyde **81** in high yield and with minimal impurities. Overall, the use of the borane-catalyzed route provides a unique opportunity to access a key component of PheronRSB, a new mating disruption solution, at an industrial scale with minimal impurities.

Finally, we note that in the majority of the above presented examples, we stopped at the acetal stage as the selective partial reduction to acetals is a more challenging step than their hydrolysis to aldehydes. These mixed acetals serve as stable and protected precursors to aldehydes, facilitating the isolation and long-term storage of these compounds. Furthermore, these compounds can be easily hydrolyzed to acetal with conventional methodologies.^26,30^ Nevertheless, an alternative hydrolytic method employing fluorspar and orthogonal further synthetic elaboration was also investigated for challenging substrates (e.g., **49**, **52**).^49^

### Mechanistic Studies

**Figure 4:**
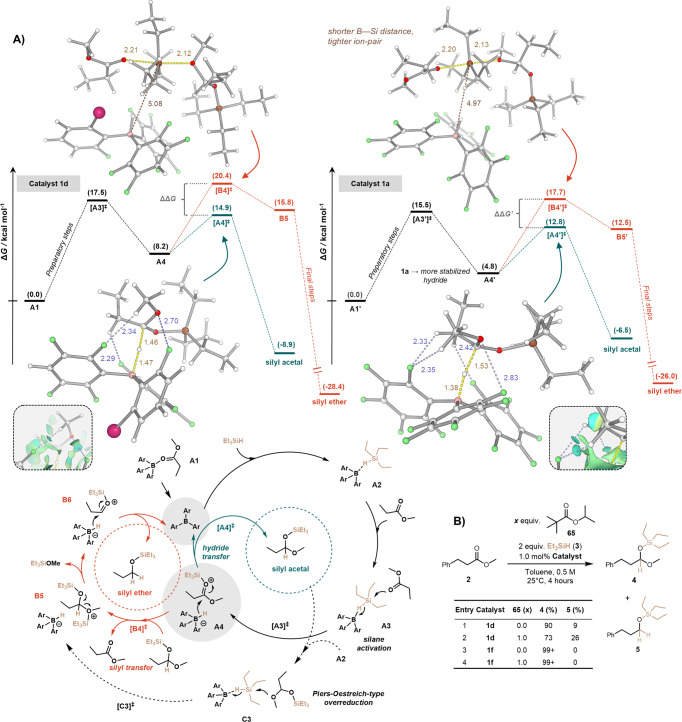
Mechanistic studies. (A) Computed free energy profiles of mechanistic routes involving **1d** and **1a** as a catalyst on the LNO–CCSD(T)/CBS//B97-3c level, for rate- and selectivity-determining states.^49^ Silane activation steps are shown in black, hydride transfer steps in teal, and silyl transfer steps in orange color. Catalytic cycles and Lewis structures are depicted for the key intermediates of the mechanism of silane activation (black arrows), hydride transfer (teal arrows), silyl transfer (orange arrows), and alternative acetal overreduction (dashed arrows). Reaction arrows indicate the progress of the reaction, and equilibrium relations are omitted for clarity. 3D structures of selectivity determining transition states are shown with noncovalent interaction (NCI) plots. Characteristic atom–atom distances are shown in ångströms. Atoms are shown in the following coloring: pink-bromine, brown-silicon, red-oxygen, green-fluorine, gray-carbon, and white-hydrogen. (B) Overreduction experiments supporting the silyl transfer mechanism.

Finally, mechanistic studies were performed to gain further insight into the mechanism underlying the high selectivity of fiddler crab-type boranes for acetal formation and the Janus-type behavior of these catalysts. The difficulty of this investigation was considerably increased by the expected mechanistic complexity of the catalytic cycles of hydrosilylation and the fleeting nature of the catalytic species. Comparative local natural orbital (LNO) coupled-cluster calculations (CCSD(T)/CBS),^49,66−68^ were performed for the underperforming **1a** and the more selective **1d** catalysts in combination with experimental studies (Figure 4). First, we established that the resting state of borane catalyst in the reaction is in the form of a borane-ester complex through slight stabilization. **1d** was found to be less sensitive to substrate inhibition, as the formation energy of its ester complex (**A1**, Δ*G* = −2.8 kcal/mol) is smaller than that of the **1a**-ester complex (**A1′**, Δ*G* = −5.3 kcal/mol). The inability of the catalyst **1d** to form a strong adduct with the ester enables an FLP scenario to be operative. Further computational analysis confirmed that the most plausible mechanism for the **1d**-promoted catalytic hydrosilylation is the Piers–Oestreich mechanism.^28,69^ Thus, the partial reduction proceeded through a synergistic Lewis acid–base Si–H activation (Figure 4A, **[A3]**^**⧧**^), which involved reversible η^1^ coordination in **A2** (Figure 4A) by the **1d** catalyst, and the attack of the carbonyl group via the rate-determining TS (**[A3]**^**⧧**^, Δ*G* = 17.5 kcal/mol; Figure 4A). This step results in a transient silylium-ester-borohydride ion pair **A4** (Δ*G* = 8.2 kcal/mol), which leads to the reduced product (via **[A4]**^**⧧**^, Δ*G* = 14.9 kcal/mol; Figure 4A, teal) after a hydride transfer step (for the derived rate laws and preliminary kinetic studies see the SI). We also computationally explored several possible overreduction pathways and revealed an unusual mechanistic scenario to explain the hydrosilylation of the acetal product. More specifically, the **A4** silylium-ester intermediate may be a branching point for competing overreduction, as the intermediate can undergo an S_N_2-type silylium cation transfer to a silyl acetal product (via the rate-determining TS **[B4]**^**⧧**^, Δ*G* = 20.4 kcal/mol). After this step, the reaction proceeds in a downhill manner via the elimination of a Et_3_SiOMe molecule and a subsequent hydride-transfer to give the silyl ether product. This mechanism is slightly more favorable than Si–H activation by the silyl acetal itself (**[C3]**^**⧧**^, Δ*G* = 21.2 kcal/mol; Figure 4A, dashed arrow), and is available only at partial conversion. Consequently, the selectivity determining barriers^70^ are the ones corresponding to **[A4]**^**⧧**^ and **[B4]**^**⧧**^ transition states and the difference between them is ΔΔ*G* = 5.5 kcal/mol, favoring the hydride transfer. Considering the **1a**-catalyzed reactions, the same silylium transfer step is responsible for overreduction, and the ΔΔ*G* between the selectivity determining barriers is slightly lower (4.9 kcal/mol). Furthermore, the more stabilized borohydride formed by **1a** steers the **A4′** ion pair lower in energy and less reactive (Δ*G*_A4′→[A4′]⧧_ = 8.0 kcal/mol). The decreased selectivity of **1a** is more pronounced after the substrate is fully converted and overreduction can proceed only through the Piers–Oestreich-type Si–H activation via **[C3′]**^**⧧**^ (Δ*G* = 14.0 kcal/mol) for **1a** and via **[C3]**^**⧧**^ (Δ*G* = 18.4 kcal/mol) for **1d**.

Further evidence supporting this intriguing silyl transfer pathway was sought experimentally in addition to the preliminary mechanistic studies using excess of reducing agent **3.**(49) Upon catalytic reduction of the ester to an acetal (Figure 4B), the nonreactive, spectator pivalate ester **65** was added to the reaction mixture as a “dummy substrate” to study its effect. Pivalate **65** enhanced the ratio of overreduced side product **5**, which can be explained by its ability to transfer the silylium cation to the acetal product. Importantly, this pivalate “enhancement” phenomenon was not observed for the more selective **1f** catalyst.

Additionally, extensive conformational analysis^71^ has shown that the bromine substituent of **1d** results in a changing conformational preference during the reaction. On one hand, for sterically crowded states **[A3]**^**⧧**^, **A4**, and **[A4]**^**⧧**^, bromine prefers the backside orientation, allowing tight packing between fluorines and the coordinating molecule on the front side (Figure 4A). The role of these *ortho*-fluorines can be rationalized via noncovalent interaction analysis (NCI),^72^ which revealed stabilizing interactions with close-lying hydrogen and oxygen atoms (note the highlighted distances in Figure 4A). On the other hand, in **[B4]**^**⧧**^, the borohydride is a spectator anion and bromine rotates to the front side leading to a slightly more separated ion pair than in **[B4′]**^**⧧**^ (B–Si distance 5.08 vs 4.97 Å, respectively; Figure 4A), with increased front strain upon hydride transfer.

## Conclusions

Overall, the above-described attributes clearly distinguish these fiddler crab-type boranes from all other catalysts and stoichiometric reagents previously used for the partial reduction of esters. This metal-free catalytic reaction can be conducted and easily scaled up at ambient temperature, without a solvent, using low catalyst loads and commercially available and inexpensive reducing agents. In addition to their high catalytic activity and selectivity, the applied catalysts are tolerant to various functional groups, and most importantly, exhibit exclusive selectivity which often obviates the need for chromatography to separate side products. Accordingly, this metal-free method not only approximates, but exceeds the activity/selectivity bar set by firmly established transition metal catalysts. Mechanistically, the high selectivity results from the following competing reaction steps involving the silylium ester intermediate: hydride transfer, which results in the desired product, or silylium transfer, which leads to a downhill path of overreduction. Our state-of-the-art coupled cluster calculations and the experimental evidence suggest that this alternative silylium transfer is favored over a Piers–Oestreich-type overreduction mechanism. Accordingly, this method extends beyond being a direct and selective ester-reducing protocol, providing new opportunities in the emerging field of lipid and pheromone synthesis.

## Supporting Information Available

Materials and methods; mechanistic and theoretical investigations; NMR spectra; Figures S1–S18; Tables S1–S11; and References (73−92) (PDF)
