# Supplementary material for: Selective Reduction of Esters to Access Aldehydes Using Fiddler Crab-Type Boranes
Source: J Am Chem Soc. 2024 Dec 26;147(1):1112–22. doi: 10.1021/jacs.4c14596 (PMC11726553; doi:10.1021/jacs.4c14596)

Supporting Information

Selective Reduction of Esters to Access Aldehydes Using Fiddler Crab-Type Boranes

Ádám Dudás[a,b], Ádám Gyömöre[a], Bence Balázs Mészáros[a,b], Stefánia Gondár[a], Renáta Adamik[a], Dániel Fegyverneki[a], Dávid Papp[b,c], Konrad Bernhard Otte[d], Sergio Ayala Jr.[d], János Daru[e], József Répási[f], Tibor Soós*[a]

[a] Organocatalysis Research Group, Institute of Organic Chemistry
HUN-REN Research Centre for Natural Sciences
Magyar tudósok körútja 2, Budapest, H-1117, Hungary

[b] Hevesy György PhD School of Chemistry
Eötvös Loránd University
Pázmány Péter sétány 1/A, Budapest, H-1117, Hungary

[c] MTA-ELTE Lendület Ion Mobility Mass Spectrometry Research Group,
Eötvös Loránd University
Pázmány Péter sétány 1/A, Budapest, H-1117, Hungary.

[d] Provivi, Inc., Santa Monica, CA 90404, USA

[e] Department of Organic Chemistry
Eötvös Loránd University
Pázmány Péter sétány 1/A, Budapest, H-1117, Hungary.

[f] Aldexchem Ltd., Érd, H-2030, Hungary.

*Corresponding author. Email: soos.tibor@ttk.hu

The PDF file includes:

Materials and Methods
Mechanistic and Theoretical Investigations
NMR Spectra
Figs. S1 to S18
Tables S1 to S11
References (73-92)

Table of Contents

1. Materials and Methods 3
  1.1 General Information 3
  1.2 Synthesis of Fiddler Crab-Type Boranes 5
    1.2.1 General procedure A for the synthesis of aryl trifluoroborate salts 6
    1.2.2 General procedure B1 for the synthesis of fiddler crab-type boranes 8
    1.2.3 General procedure B2 for the synthesis of fiddler crab-type boranes 10
    1.2.4 Prepared borane catalysts 12
    1.2.5 Large-scale laboratory synthesis of **1d** 17
    1.2.6 Relative Lewis acidity of the prepared catalysts according to the Gutmann–Beckett method 19
  1.3 Optimalization of the Hydrosilylation Reaction 20
    1.3.1 General procedure C for optimalization of the hydrosilylation reaction 20
    1.3.2 Catalyst screening 21
    1.3.3 Solvent screening 23
    1.3.4 The effect of the excess of reducing agent 24
    1.3.5 Screening the catalyst load and concentration 25
    1.3.6 Reaction temperature optimalization 25
    1.3.7 Screening of silane reducing agents 27
    1.3.8 NMR study of the hydrosilylation reaction 29
    1.3.9 Kinetic models and preliminary studies 32
  1.4 The Partial Reduction of Esters Using Fiddler Crab-Type Boranes 36
    1.4.1 General procedure D for the partial reduction of esters via hydrosilylation 36
    1.4.2 Scope of the partial reduction of esters using fiddler crab-type boranes 38
    1.4.3 Limitations of this methodology 69
    1.4.4 Synthesis of starting materials 70
  1.5 Applications 73
    1.5.1 Kilogram-scale laboratory reduction of fatty acid esters 73
    1.5.2 Reduction of Roche ester 75
    1.5.3 Streamlined synthesis of ALC-0315 80
    1.5.4 Industrial-scale pheromone synthesis, Pheron®RSB 85
    1.5.5 Notes on the hydrolysis and application of the formed mixed acetals 87
2. Mechanistic and Theoretical Investigations 91
  2.1 Computational methods 91
  2.2 Results on the double-hybrid level 92
    2.2.1 Borane complexes 92
    2.2.2 Mechanism of the reduction 93
    2.2.3 Mechanism of the overreduction 94
  2.3 LNO-CCSD(T) results 98
    2.3.1 Comparison of **1a** and **1d** catalyst selectivity 98
    2.3.2 Energetics for key stationary points 99
  2.4 Cartesian coordinates 100
  2.5 Experiments to support the silyl transfer pathway 120
3. NMR Spectra 122

1. Materials and Methods

1.1 General Information

All commercially available substances and reagents (purchased from these commercial suppliers: Acros Organics, Fluorochem, Merck, Sigma-Aldrich and VWR) were used as received, unless otherwise noted. All reactions were carried out using oven-dried glassware (dried at 140°C for at least 8 hours), under an inert atmosphere of nitrogen or argon, and using anhydrous solvents, unless otherwise noted. The solvents were dried prior to use by freshly distilling them from sodium/benzophenone (in the case of tetrahydrofuran (THF), diethyl ether (DEE), toluene ($PhCH_3$), hexanes (C_6H_{14}) and *n*-pentane (C_5H_{12})) or from calcium hydride (in the case of dichloromethane (DCM, stabilized with amylene), acetonitrile (CH_3CN), methanol (MeOH), ethanol (EtOH)) under an inert atmosphere of nitrogen, or by storing them on activated 4Å molecular sieves (4Å MS) for at least 2 weeks (in the case of dimethylformamide (DMF) and dimethyl sulfoxide (DMSO)). The inert gases used had a purity of at least 4.6, with a maximum water concentration of 5 ppm (V/V).

All air- and moisture-sensitive manipulations were carried out in a GS Glovebox (operated in overpressure mode using nitrogen inert gas) or using a 5-port Schlenk line (with nitrogen as an inert gas and a Vacuubrand RZ 6 rotary vane pump used as a vacuum source) employing standard Schlenk and cannula techniques. Prepared compounds were long-term stored under an atmosphere of argon, at -20°C (in the case of the acetals), or in the glovebox at room temperature (25°C, nitrogen atmosphere; in the case of the borane catalysts).

Flash chromatographic purifications were performed using Teledyne ISCO CombiFlash systems on silica gel columns (RediSep Gold Normal Phase Silica Gel columns, 20-40 microns), with the indicated eluents. Compound **63** was purified on a basic alumina column (RediSep Alumina, Basic, 40-60 microns, 80g). Thin-layer chromatographic analyses (TLC) were performed on silica plates (Kieselgel 60 F_{254}, Merck) using the indicated eluents. For TLC analysis of compound **63** neutral alumina plates were used (Aluminium oxide 60 F_{254}, Merck). Compounds were visualized using UV light (254 nm or 365 nm) or TLC stain solutions (e.g., $KMnO_4$ or 2,4-dinitrophenylhydrazine (DNPH)).

Exact mass measurements (HRMS) were performed on a high-resolution Waters ACQUITY RDa Detector (Waters Corp., Wilmslow, U.K.) equipped with an electrospray ionization source. Samples were dissolved in methanol. Flow injection analysis was performed using a 50 µL/min eluent flow. 10-10 µL sample solutions were injected by a Waters ACQUITY UPLC H-Class PLUS system. Leucin-Enkephalin peptide was used for single Lock Mass calibration correction. Borane compounds were detected as their hydroxy adducts, while acetal compounds were usually detected as their sodium adducts.

^{1}H-, ^{13}C-, ^{10}B-NMR, and ^{31}P-NMR spectra were recorded using a Varian 500 MHz INOVA spectrometer, while ^{19}F-NMR spectra (and a few ^{1}H- and ^{13}C-NMR spectra) were recorded using a Varian 300 MHz INOVA spectrometer. The ^{1}H-NMR for compound **81** was recorded on a Bruker Avance 500 MHz spectrometer. For ^{1}H and ^{13}C measurements, chemical shifts were referenced to the residual solvent signals ($CDCl_3$: δ = 7.26 ppm for ^{1}H, δ = 77.0 ppm for ^{13}C; DMSO-d^{6}: δ = 2.50 ppm for ^{1}H, δ = 39.5 ppm for ^{13}C; C_6D_6: δ = 7.16 ppm for ^{1}H, δ = 128.1 ppm for ^{13}C). ^{19}F-NMR spectra were referenced externally to benzotrifluoride at δ = -63.7 ppm, ^{10}B-NMR spectra were referenced externally to $BF_3 \cdot Et_2O$ at δ = 0.0 ppm, while ^{31}P-NMR spectra were referenced externally to H_3PO_4 (85%) at δ = 0.0 ppm. Data are reported as follows: chemical shifts (δ, ppm), multiplicity (s = singlet, d = doublet, t = triplet, q = quartet, p = pentet, br = broad, m = multiplet), and coupling constants (*J*, Hz). For spectra containing mixtures of diastereomers, the peaks of the minor diastereomer were marked with an asterisk (*). All spectra were recorded using the standard Varian/VnmrJ pulse sequences and settings at 30°C, ^{1}H decoupling was used for ^{13}C-NMR and ^{31}P-NMR measurements. For the

measurement of moisture-sensitive samples (i.e., borane catalysts) anhydrous NMR solvents (dried over activated 4Å molecular sieves for at least 1 week) were used and the NMR tubes were oven-dried at 140°C for at least 16 hours.

Gas chromatography–mass spectrometry (GC-MS) analyses were performed on a Shimadzu GC-2010 instrument equipped with a GCMS-QP2010 Ultra detector, using EI ionization. The analytical method used a Zebron ZB-5MSi column (30 m, ID: 0.25 mm, df: 0.25 μm), helium as a carrier gas, and an oven temperature profile as follows: 0 min.: 80°C; 11 min.: 250°C; 16 min.: 250°C. Further parameters: injector temperature: 250°C; gas flow rate: 27.7 mL/min.; split ratio: 1/20, detector temperature: 280°C.

High-performance liquid chromatography mass spectrometry (HPLC-MS) analyses were performed on a Shimadzu LC-MS-2020 instrument, using ESI ionization. The analytical method used a Phenomenex Kinetex EVO C18 column (2.6 μm, 100 Å, 50x2.1 mm) and the following eluents: eluent A (95 V/V% H_2O, 5 V/V% CH_3CN, 0.1 V/V% HCOOH) and eluent B (95 V/V% CH_3CN, 5 V/V% H_2O, 0.1 V/V% HCOOH). Gradient elution profile: 0 min.: 0 V/V%B; 8 min.: 100 V/V%B; 8.5 min.: 100 V/V%B; 9 min.: 0 V/V%B; 10 min.: 0 V/V%B. Further parameters: column temperature: 40°C; flow rate: 1 mL/min.; split ratio: 1/20.

The enantiomeric excesses (ee) of products were determined by chiral stationary phase HPLC analysis using a Jasco HPLC system (equipped with PDA detector) and Daicel Chiralpak ID (5 μm , 4.6 mm x 250 mm) column. We used isocratic elution, with an eluent comprising of 99.5 V/V% *n*-hexane and 0.5 V/V% 2-propanol. Further parameters: column temperature: 15°C; flow rate: 1 mL/minute.

1.2 Synthesis of Fiddler Crab-Type Boranes

The synthesis of novel fiddler crab-type boranes was achieved using the synthetic procedure previously developed in our research group[38], with slight modifications. The general synthetic plan is outlined in Fig. S1 and starts with the synthesis of an aryl trifluoroborate salt from the respective haloarene through an arylboronic acid intermediate (Fig. S1, General procedure A). The use of trifluoroborate salts for the synthesis of boranes (instead of the commonly employed direct use of boronic acid derivatives) is advantageous because higher yields and selectivity can be achieved. Also, trifluoroborate salts can be easily purified and are bench stable (they can be stored at 25°C on air for one year, without degradation of their quality). The aryl trifluoroborate salt is in turn reacted with 2 equivalents of a Grignard reagent either prepared directly from the corresponding aryl bromide substrate (Fig. S1, General procedure B1), or via the ortho lithiation of the respective fluoroarene followed by transmetalation with $MgBr_2·Et_2O$ (Fig. S1, General procedure B2), to finally yield the respective fiddler crab-type borane. Although General procedure B2 has more reaction steps and uses multiple reagents, it still might be advantageous compared to B1 during large-scale synthesis, as the price of the corresponding fluoroarene is usually significantly lower than the price of the aryl bromide starting material.

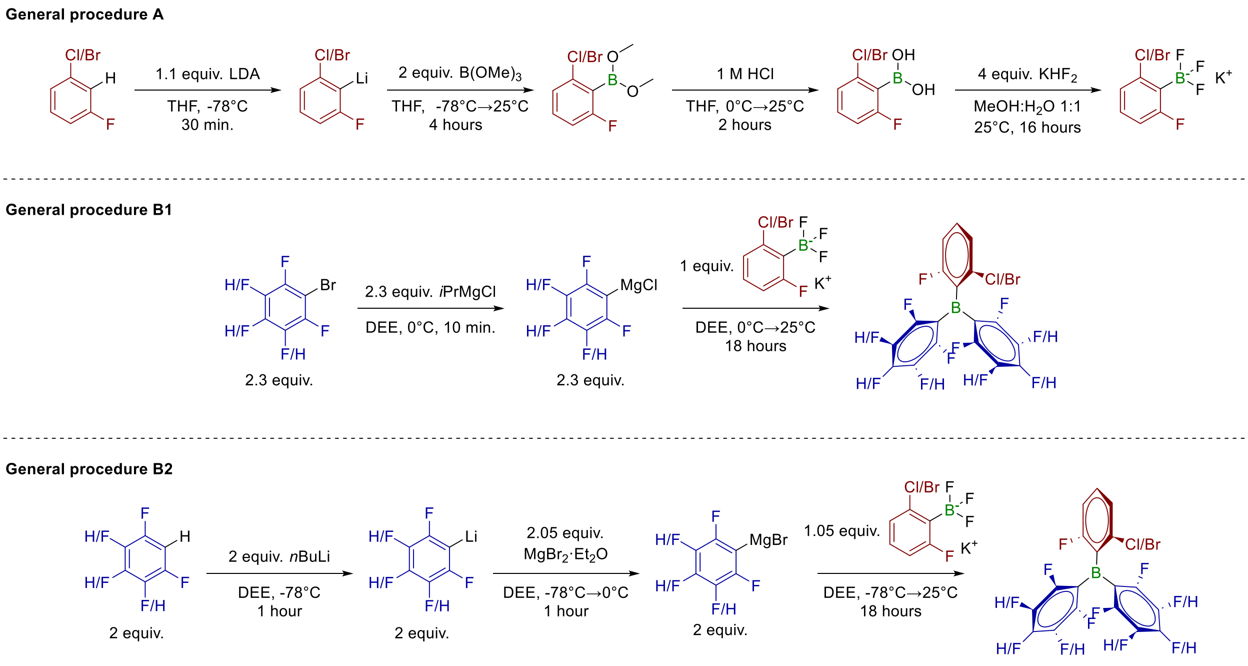

Fig. S1. General synthetic procedures for the synthesis of fiddler crab-type boranes

General procedure A: Synthesis of aryl trifluoroborate salts; General procedure B1: Synthesis of fiddler crab-type boranes using aryl bromides; General procedure B2: Synthesis of fiddler crab-type boranes using fluoroarenes. Abbreviations: LDA = lithium diisopropylamide, THF = tetrahydrofuran, DEE = diethyl ether

1.2.1 General procedure A for the synthesis of aryl trifluoroborate salts

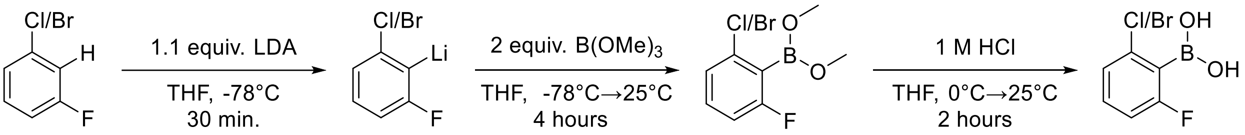

Cl: **S1**
Br: **S2**

In a 500 mL 3-necked flask, equipped with a condenser, nitrogen purge inlet and an immersion probe digital thermometer, diisopropylamine (8.90 g, 13 mL, 1.1 equiv., 88.0 mmol) was dissolved in tetrahydrofuran (THF, 200 mL, anhydrous) and was cooled to -78°C (using an acetone/dry ice bath). The solution of *n*-butyllithium (5.64 g, 35.2 mL, 1.1 equiv., 88.0 mmol, 2.5 M in hexanes) was added dropwise, keeping the reaction temperature below -60°C. Following addition, the reaction mixture was stirred for 30 min. at -78°C. Then, 1-chloro-3-fluorobenzene (10.4 g, 8.57 mL, 1 equiv., 80.0 mmol) or 1-bromo-3-fluorobenzene (14.0 g, 8.93 mL, 1 equiv., 80.0 mmol) was added dropwise within 5 min., keeping the reaction temperature below -70°C. The mixture was stirred for an additional 30 min. at -78°C [Note 1.]. Then, trimethyl borate (16.6 g, 17.8 mL, 2 equiv., 160 mmol) was added dropwise within 10 min. and the reaction temperature was maintained below -70°C. The reaction mixture was further stirred for 30 min. at -78 °C, then left to warm up to 25°C (by removing the acetone/dry ice bath) and stirred for another 4 hours. Afterwards, the reaction mixture was cooled down to 0°C (using an ice bath) and 250 mL 1M HCl solution (precooled to 0°C) was added dropwise, keeping the temperature below 6°C. The reaction was left to warm up to 25°C (by removing the ice bath) and stirred for another 2 hours. Then, 160 mL of diethyl ether was added, and the phases were separated. The aqueous phase was washed with another 40 mL of diethyl ether. The combined organic phase was washed with 2x160 mL brine and dried using Na_2SO_4. Finally, the solvents were evaporated on a rotary evaporator yielding a crude, white crystalline product, which can be used for the next synthetic step without further purification.

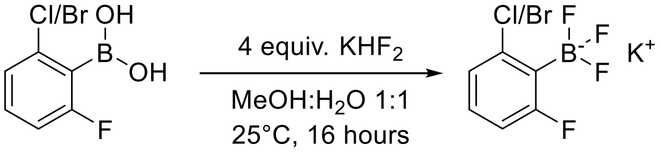

Cl: **S1** Cl: **S3**
Br: **S2** Br: **S4**

In a white 1000 mL polypropylene container, (2-chloro-6-fluorophenyl)boronic acid (**S1**, 13.9 g, 1 equiv., 80.0 mmol) or (2-bromo-6-fluorophenyl)boronic acid (**S2**, 17.5 g, 1 equiv., 80.0 mmol) was measured in and dissolved in methanol (90 mL, technical grade). Then, potassium hydrogen fluoride (25.0 g, 4 equiv., 320 mmol) dissolved in water (90 mL, RO purified) was added in one portion. The resulting suspension was intensely stirred for 16 hours. Afterwards, 500 mL of acetone was added, and the reaction mixture was stirred for further 30 minutes. The reaction mixture was filtered through pleated filter paper and the solvents were evaporated under reduced pressure on a rotary evaporator (water bath temperature: 60°C). After all the solvents were evaporated, an additional 400 mL acetone was added to the crude reaction mixture and the solvents were evaporated once again to remove traces of water. Finally, 100 mL toluene (technical grade) was added and evaporated the same way. The obtained white powder was dissolved once again in 100 mL acetone and filtered through pleated filter paper [Note 2.]. The filtrate was evaporated, and the obtained white powder was triturated with 100 mL of hexanes (technical grade) and filtered out. The filter cake was washed with 2x50 mL cold diethyl ether (technical grade) on the filter, dried under reduced pressure on a rotary

evaporator (water bath temperature: 60°C) and further dried in a vacuum desiccator for 16 hours (using P_4O_{10} as desiccant). The product is a white, crystalline solid.

[Note 1.] The progress of the lithiation can be monitored using GC-MS by quenching a small sample of the reaction with CH_3OD.
[Note 2.] The consecutive solvent additions and evaporations are needed for the removal of the residual water content, which can solubilize some inorganic salts during the first filtration. After most of the water has been removed, all inorganics can be removed too during the second filtration step.

Compound S3: potassium (2-chloro-6-fluorophenyl)trifluoroborate

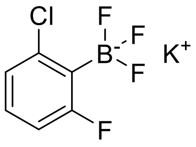

S3

Compound **S3** was prepared according to General procedure A on an 80 mmol scale, yielding 14.7 g (62.2 mmol) of pure product, which corresponds to an overall yield of 78%. The NMR spectral data of the final product is in accordance with literature data[40].

Physical state: white powder

^{1}H-NMR (500 MHz, DMSO-d^6) δ = 7.09 (td, J = 8.0, 6.2 Hz, 1H), 7.00 (d, J = 7.8 Hz, 1H), 6.82 (t, J = 8.7 Hz, 1H).

^{13}C-NMR (126 MHz, DMSO-d^6) δ = 166.0 (d, J = 243.0 Hz), 138.9 (d, J = 14.8 Hz), 134.2 – 131.3 (br m), 128.0 (d, J = 10.0 Hz), 125.0 (d, J = 3.4 Hz), 113.2 (d, J = 27.9 Hz).

^{19}F-NMR (282 MHz, DMSO-d^6) δ = -102.2 – -102.5 (m), -132.2 – -132.9 (m).

Compound S4: potassium (2-bromo-6-fluorophenyl)trifluoroborate

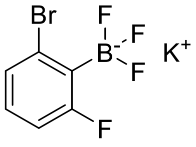

S4

Compound **S4** was prepared according to General procedure A on an 80 mmol scale, yielding 20.4 g (72.8 mmol) of pure product, which corresponds to an overall yield of 91%. (See NMR spectra)

Physical state: white powder

^{1}H-NMR (300 MHz, DMSO-d^6) δ = 7.18 (dq, J = 7.8, 0.6 Hz, 1H), 6.99 (td, J = 8.0, 6.1 Hz, 1H), 6.85 (dddt, J = 9.4, 8.1, 1.2, 0.6 Hz, 1H).

^{13}C-NMR (75 MHz, DMSO-d^6, partial) δ = 165.8 (d, J = 244.8 Hz), 128.4 (d, J = 3.2 Hz), 128.4 (d, J = 9.6 Hz), 128.0 (d, J = 14.0 Hz), 113.7 (d, J = 27.9 Hz).

^{19}F-NMR (282 MHz, DMSO-d^6) δ = -96.2 (tdd, J = 16.1, 12.6, 6.8 Hz), -127.6 – -128.6 (m).

1.2.2 General procedure B1 for the synthesis of fiddler crab-type boranes

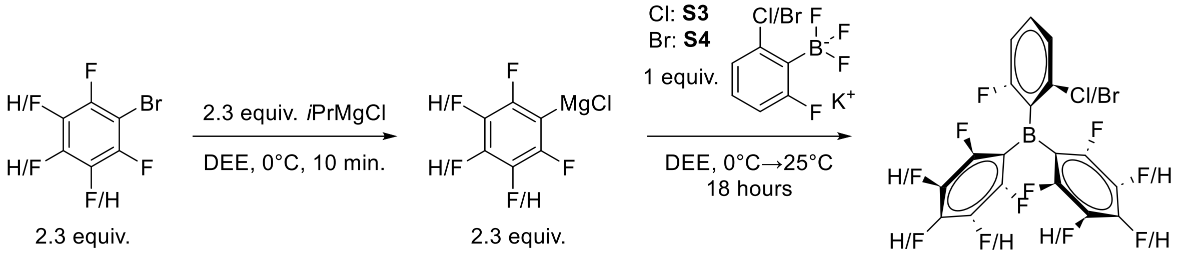

In a 100 mL 3-necked flask, equipped with a reflux condenser and a nitrogen gas inlet, magnesium turnings (1.61 g, 2.3 equiv., 66.3 mmol) were measured in and activated with iodine [Note 1.]. Then, 20 mL of anhydrous diethyl ether (DEE) [Note 2.] was added, followed by the dropwise addition of 2-chloropropane (5.21 g, 6.04 mL, 2.3 equiv., 66.3 mmol). After the Grignard reaction started (the solution started to warm up and reflux), 30 mL of diethyl ether was added to dilute the reaction, and dropwise addition of 2-chloropropane was continued to maintain reflux. After the addition is finished and the boiling of the reaction subsides, the flask is heated to maintain reflux for 1 hour (or until most of the magnesium turnings disappear). In another 250 mL 3-necked flask, equipped with a nitrogen gas inlet and an immersion probe digital thermometer, the respective aryl bromide substrate (2.3 equiv., 66.3 mmol) was measured in and dissolved in 90 mL of anhydrous diethyl ether, after which it was cooled to 0°C (using an ice bath). The previously prepared Grignard solution was added dropwise, via a syringe, within 45 min., while keeping the reaction temperature below 5°C. [Note 3.] After completion of the addition, the reaction mixture was stirred for 1 hour. The final steps of the reaction were carried out using Schlenk technique. In a 500 mL Schlenk flask, the aryl trifluoroborate salt **S3** or **S4** (1 equiv., 28.8 mmol) was measured in. Then the flask was connected to the Schlenk line via 3 vacuum/nitrogen inert gas cycles. Then the trifluoroborate salt was suspended in 20 mL anhydrous diethyl ether and cooled down to 0°C (using an ice bath). The cold (0°C) Grignard solution was added via cannula transfer within 20 min., while keeping the temperature of the reaction mixture below 4°C (measured using an immersion probe digital thermometer). The reaction mixture was left to warm up to 25°C (by removing the ice bath) and was stirred for an additional 18 hours [Note 4.]. Afterwards, the solvent was evaporated under reduced pressure (using the Schlenk line and a 50°C water bath). Next, 90 mL anhydrous toluene was added, and the suspension was sonicated for 10 minutes. The resulting precipitate was filtered off (using inert filtration, through a Celite layer [Note 5.]) and washed with 2x20 mL anhydrous toluene. The combined filtrate was then evaporated at reduced pressure (using the Schlenk line and a 70°C water bath), resulting in an off-white solid. Then, 10 mL of anhydrous *n*-pentane was added, and the resulting suspension was sonicated for 10 minutes. Finally, the solids were filtered (using inert filtration [Note 6.]), and dried under reduced pressure, to give the product as a white crystalline powder [Note 7.].

[Note 1.] A small iodine crystal was added to the magnesium turnings, then the flask was heated using a heat gun until the violet iodine vapors appeared, after which the flask was left to cool back down to room temperature.
[Note 2.] Diethyl ether cannot be substituted with tetrahydrofuran (THF) in this reaction because it would coordinate strongly to the borane, making purification cumbersome.
[Note 3.] While the reaction also works with commercially available isopropylmagnesium chloride solution (in diethyl ether), the freshly prepared reagent yielded a higher purity product. Also, the progress of the reaction can be monitored using GC-MS by quenching a small sample of the reaction mixture with methanol.
[Note 4.] The progress of the reaction can be monitored using HPLC-MS.

[Note 5.] The easiest way to carry out this step is to use a Schlenk filter equipped with a glass frit. The Celite layer is added before the whole filter in dried in a 140°C oven for at least 8 hours. Before the filtration step, the filtering apparatus is assembled together by connecting the filter to another oven-dried 250 mL Schlenk flask while they are still hot and is left to cool down under vacuum. The suspension to be filtered is transferred to this apparatus via a cannula.
[Note 6.] This step can also be conducted using a Schlenk filtering apparatus, but the cannula filtration technique is preferred. A short and narrow oven-dried glass tube (that fits through the Schlenk flask's neck and joint) is connected to the end of a cannula and secured using PTFE tape. Then, a filter paper is attached to the other end of the glass tube and secured using PTFE tape, forming a plain filtering surface. Finally, the other end of the cannula is routed through a rubber seal, so it can be fitted onto the Schlenk flask. This filtering "probe" can be immersed into the suspension to remove the liquids by connecting the other end of the cannula to an empty flask with vacuum. This technique is preferred because it leaves the final product inside a Schlenk flask that is easily transferable to a glovebox. Also, after filtering the product, the Schlenk flask can be sealed again, and the solids can be dried in vacuum to remove any solvent residues.
[Note 7.] After filtration, the Schlenk flask or the Schlenk filter containing the catalyst was transferred into a glovebox under vacuum. All subsequent manipulations involving the solid catalyst in its pure form were carried out inside the glovebox.

1.2.3 General procedure B2 for the synthesis of fiddler crab-type boranes

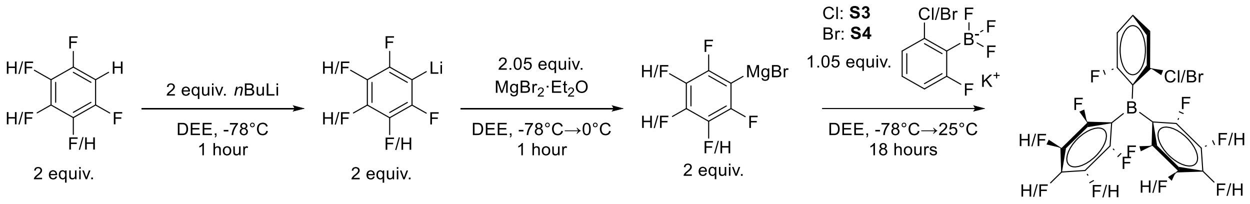

In a 250 mL 3-necked flask, equipped with a nitrogen gas inlet and an immersion probe digital thermometer, the respective fluoroarene (2.0 equiv., 61.0 mmol) was measured in and dissolved in 80 mL anhydrous diethyl ether (DEE) [Note 1.]. The solution was cooled to -78°C (using an acetone/dry ice bath), after which *n*-butyllithium (3.91 g, 24.4 mL, 2.5 M in hexanes, 2.0 equiv., 61.0 mmol) was added dropwise over 1 hour, keeping the temperature below -70°C. After completion of the addition, the mixture was maintained at -78°C for further 1 hour [Note 2.]. Then a previously prepared $MgBr_2$ solution (11.5 g, 2.05 equiv., 62.6 mmol) in 50 mL anhydrous diethyl ether [Note 3.] was added dropwise, keeping the temperature below -70°C. After completing the addition, the reaction mixture was stirred for another 1 hour at 0°C (using an ice bath). In a 500 mL Schlenk flask, the aryl trifluoroborate salt **S3** or **S4** (1.05 equiv., 32 mmol) was administered. Then the flask was connected to the Schlenk line via 3 vacuum/nitrogen inert gas cycles and the solids were suspended in 30 mL anhydrous diethyl ether. The suspension was cooled to -78°C (using an acetone/dry ice bath) and the cold Grignard reagent (0°C) was added via cannula transfer over 20 minutes. The reaction temperature was monitored (using an immersion probe digital thermometer) and kept below -70°C. The reaction mixture was stirred for another 2 hours at a temperature of -78°C, after which it was left to warm up in the acetone/dry ice cooling bath overnight and stirred for a total of 18 hours [Note 4.]. Next day, 8.0 mL trimethylsilyl chloride (TMSCl, 6.8 g, 2.05 equiv., 63 mmol) was added to the reaction mixture and it was stirred for another 2 hours [Note 5.]. Then, 50 mL anhydrous toluene was added to the mixture and the diethyl ether was evaporated under reduced pressure (using the Schlenk line) [Note 6.]. The resulting precipitate was filtered off (using inert filtration, through a Celite layer [Note 7.]) and washed with 2x20 mL anhydrous toluene. The resulting filtrate was concentrated to about 10-20 mL solvent volume under reduced pressure (using the Schlenk line and a 70°C water bath), after which 40 mL anhydrous hexanes was added, resulting in the precipitation of an off-white solid. The resulting suspension was cooled to -78°C (using an acetone/dry ice bath) and the solid was filtered using the cannula filtering technique [Note 8.]. The solids were washed with additional 15 mL anhydrous hexanes. Finally, the obtained solid was dried for 2 hours under reduced pressure (using the Schlenk line and a 60°C water bath), to give the product as a white crystalline powder [Note 9.].

[Note 1.] Diethyl ether cannot be substituted with tetrahydrofuran (THF) in this reaction because it would coordinate strongly to the borane, making purification cumbersome.
[Note 2.] The progress of the lithiation can be monitored using GC-MS by quenching a small sample of the reaction with CH_3OD.
[Note 3.] The $MgBr_2$ solution was freshly prepared: to activated magnesium turnings (1.52 g, 2.05 equiv., 62.6 mmol) 20 mL anhydrous diethyl ether was added, followed by the dropwise addition of 1,2-dibromoethane (11.8 g, 5.39 mL, 2.05 equiv., 62.6 mmol). After the Grignard reaction started (the solution started to warm up and reflux), 30 mL of diethyl ether was added to dilute the reaction, and dropwise addition of 1,2-dibromoethane was continued to maintain reflux. After the addition is finished and the boiling of the reaction subsides, the flask is heated to maintain reflux for 1 hour (or until most of the magnesium turnings disappear). Alternatively, previously prepared $MgBr_2{\cdot}Et_2O$ can be added to the reaction mixture directly in one portion.
[Note 4.] The progress of the reaction can be monitored using HPLC-MS.

[Note 5.] TMSCl is used to quench any remaining organometallic reagent. This step is crucial for the subsequent filtration step, as the suspension to be filtered won't be as gelatinous and won't clog the apparatus.
[Note 6.] Removal of diethyl ether is important, because it can solubilize $MgBr_2$ to some extent, which can lead to inorganic impurities in the final product. To facilitate this step, mark the 50 mL liquid level on the Schlenk flask before use and remove solvents until the liquid level of the reaction mixture reaches this mark. Alternatively, removal of some toluene also ensures efficient removal of residual diethyl ether.
[Note 7.] The easiest way to carry out this step is to use a Schlenk filter equipped with a glass frit. The Celite layer is added before the whole filter in dried in a 140°C oven for at least 8 hours. Before the filtration step, the filtering apparatus is assembled together by connecting the filter to another oven-dried 250 mL Schlenk flask while they are still hot and is left to cool down under vacuum. The suspension to be filtered is transferred to this apparatus via a cannula.
[Note 8.] This step can also be conducted using a Schlenk filtering apparatus, but the cannula filtration technique is preferred. A short and narrow oven-dried glass tube (that fits through the Shlenk flask's neck and joint) is connected to the end of a cannula and secured using PTFE tape. Then, a filter paper is attached to the other end of the glass tube and secured using PTFE tape, forming a plain filtering surface. Finally, the other end of the cannula is routed through a rubber seal, so it can be fitted onto the Schlenk flask. This filtering "probe" can be immersed into the suspension to remove the liquids by connecting the other end of the cannula to an empty flask with vacuum. This technique is preferred because it leaves the final product inside a Schlenk flask that is easily transferable to a glovebox. Also, after filtering the product, the Schlenk flask can be sealed again, and the solids can be dried in vacuum to remove any solvent residues.
[Note 9.] After filtration, the Schlenk flask or the Schlenk filter containing the catalyst was transferred into a glovebox under vacuum. All subsequent manipulations involving the solid catalyst in its pure form were carried out inside the glovebox.

1.2.4 Prepared borane catalysts

Compound 1b: (2,6-difluorophenyl)bis(2,3,5,6-tetrafluorophenyl)borane

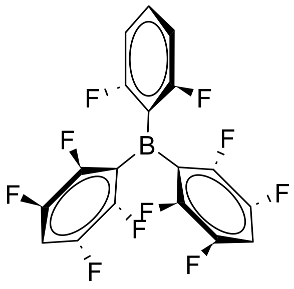

1b

Compound **1b** was prepared according to General procedure B2, starting form potassium (2,6-difluorophenyl)trifluoroborate on a 2.00 mmol scale, yielding 333 mg (789 µmol) of pure product, which corresponds to an overall yield of 40%. (See NMR spectra)

Physical state: white powder

^{1}H-NMR (500 MHz, C_6D_6) δ = 6.68 (tt, J = 8.4, 6.6 Hz, 1H), 6.35 (t, J = 8.3 Hz, 2H), 6.25 (tt, J = 9.4, 7.5 Hz, 2H).

^{13}C-NMR (126 MHz, C_6D_6) δ = 166.1 (dd, J = 253.5, 10.3 Hz), 147.4 (dtd, J = 243.2, 8.8, 5.7 Hz), 146.1 (dddd, J = 250.1, 16.6, 8.9, 3.8 Hz), 137.7 – 137.1 (m), 121.0 (br s), 117.1 (br s), 111.9 – 111.5 (m), 110.5 – 110.0 (m).

^{19}F-NMR (282 MHz, C_6D_6) δ = -97.6 (s), -130.5 – -132.0 (m), -137.3 – -139.2 (m).

^{10}B-NMR (54 MHz, C_6D_6) δ = 59.0.

HRMS (ESI-/Q-TOF): Calculated for $[M+OH]^-$ = $[C_{18}H_6OBF_{10}]^-$: m/z 438.03938; Found: m/z 438.03998

Compound 1c: (2,6-dibromophenyl)bis(2,3,5,6-tetrafluorophenyl)borane

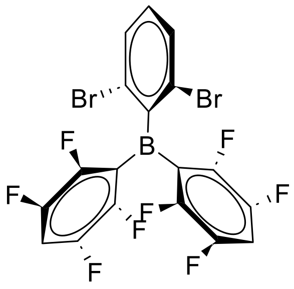

1c

Compound **1c** was prepared according to General procedure B2, starting from potassium (2,6-dibromophenyl)trifluoroborate on a 3.00 mmol scale, yielding 939 mg (1.73 mmol) of pure product, which corresponds to an overall yield of 58%. (See NMR spectra)

Physical state: white powder

^{1}H-NMR (500 MHz, C_6D_6) δ = 7.01 (d, J = 8.1 Hz, 2H), 6.38 (t, J = 8.1 Hz, 1H), 6.25 (tt, J = 9.3, 7.4 Hz, 2H).

^{13}C-NMR (126 MHz, C_6D_6) δ = 148.8 (d, J = 255.2 Hz), 146.0 (d, J = 247.0 Hz), 145.5 (br s) 131.8, 130.2, 123.1, 119.0 (br s), 111.7 (t, J = 22.6 Hz).

^{19}F-NMR (282 MHz, C_6D_6) δ = -128.5 (ddd, J = 20.9, 13.1, 6.9 Hz), -138.1 – -138.3 (m).

^{10}B-NMR (54 MHz, C_6D_6) δ = 60.4.

HRMS (ESI-/Q-TOF): Calculated for $[M+OH]^-$ = $[C_{18}H_6OBBr_2F_8]^-$: m/z 557.87925; Found: m/z 557.87980

Compound 1d: (2-bromo-6-fluorophenyl)bis(2,3,5,6-tetrafluorophenyl)borane

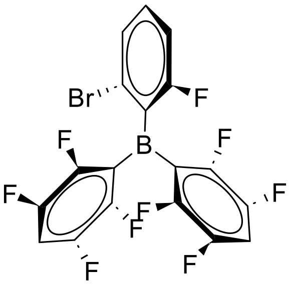

1d

Compound **1d** was prepared according to General procedure B2, starting from **S4** on a 30.5 mmol scale, yielding 9.36 g (19.4 mmol) of pure product, which corresponds to an overall yield of 64%. (See NMR spectra)

Physical state: white powder

^{1}H-NMR (500 MHz, C_6D_6) δ = 6.93 (dd, J = 6.6, 2.1 Hz, 1H), 6.59 – 6.50 (m, 2H), 6.24 (tt, J = 9.4, 7.5 Hz, 2H).

^{13}C-NMR (126 MHz, C_6D_6) δ = 162.8 (d, J = 245.9 Hz), 148.4 (dddd, J = 251.4, 12.8, 8.6, 3.7 Hz), 146.2 (dm, J = 249.6 Hz), 133.2 (d, J = 8.9 Hz), 132.4 – 131.6 (br m), 128.3 (d, J = 3.2 Hz), 123.2 (d, J = 9.3 Hz), 120.5 – 119.1 (br m), 114.1 (d, J = 23.1 Hz), 111.8 (tt J = 22.7, 2.0 Hz).

^{19}F-NMR (282 MHz, C_6D_6) δ = -102.2 – -102.4 (m), -129.2 (ddd, J = 22.1, 14.3, 7.7 Hz), -137.9 – -138.5 (m).

^{10}B-NMR (54 MHz, C_6D_6) δ = 64.4.

HRMS (ESI-/Q-TOF): Calculated for $[M+OH]^-$ = $[C_{18}H_6OBBrF_9]^-$: m/z 497.95932; Found: m/z 497.95986

Compound 1e: (2-bromo-6-fluorophenyl)bis(perfluorophenyl)borane

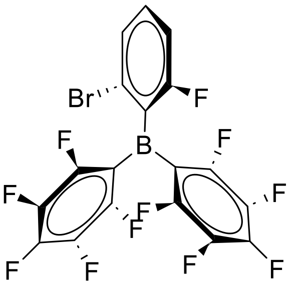

1e

Compound **1e** was prepared according to General procedure B1, starting from **S4** on a 18.0 mmol scale, yielding 3.13 g (6.03 mmol) of pure product, which corresponds to an overall yield of 34%. (See NMR spectra)

Physical state: white powder

^{1}H-NMR (500 MHz, C_6D_6) δ = 7.03 (d, J = 8.1 Hz, 2H), 6.40 (t, J = 8.1 Hz, 1H).

^{13}C-NMR (75 MHz, C_6D_6) δ = 162.7 (d, J = 245.5 Hz), 149.3 (dm, J = 252.3 Hz), 145.3 (dm, J = 261.5 Hz), 137.5 (dm, J = 253.1 Hz), 133.2 (d, J = 9.1 Hz), 132.2 – 131.5 (br m), 128.3 (d, J = 3.1 Hz), 123.2 (d, J = 9.5 Hz), 114.1 (d, J = 23.1 Hz), 114.2 – 113.3 (br m).

^{19}F-NMR (282 MHz, C_6D_6) δ = -102.4 (tt, J = 7.4, 2.3 Hz), -127.9 – -128.1 (m), -142.9 (tt, J = 20.9, 6.9 Hz), -160.6 – -160.9 (m).

^{10}B-NMR (54 MHz, C_6D_6) δ = 62.3.

HRMS (ESI-/Q-TOF): Calculated for $[M+OH]^-$ = $[C_{18}H_4OBBrF_{11}]^-$: m/z 533.94047; Found: m/z 533.94100

Compound 1f: (2-bromo-6-fluorophenyl)bis(2,3,6-trifluorophenyl)borane

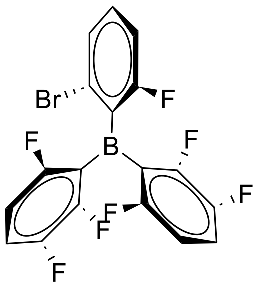

1f

Compound **1f** was prepared according to General procedure B2, starting from **S4** on a 84.8 mmol scale, yielding 23.9 g (53.5 mmol) of pure product, which corresponds to an overall yield of 63%. (See NMR spectra)

Physical state: white powder

^{1}H-NMR (500 MHz, C_6D_6) δ = 6.98 (dd, J = 7.6, 1.3 Hz, 1H), 6.63 – 6.53 (m, 2H), 6.48 (qd, J = 9.2, 5.1 Hz, 2H), 6.14 (tdd, J = 9.0, 3.3, 1.9 Hz, 2H).

^{13}C-NMR (126 MHz, C_6D_6) δ = 162.9 (d, J = 245.2 Hz), 160.7 (ddd, J = 249.9, 8.6, 2.5 Hz), 153.0 (ddd, J = 254.4, 13.3, 11.4 Hz), 147.3 (ddd, J = 246.0, 14.7, 3.6 Hz), 133.5 – 132.9 (br m), 132.5 (d, J = 8.9 Hz), 128.2 (d, J = 3.1 Hz), 123.6 (d, J = 9.6 Hz), 122.8 (ddd, J = 19.6, 11.3, 2.5 Hz), 119.8 – 119.2 (br m), 113.9 (d, J = 23.3 Hz), 111.5 (ddd, J = 27.6, 5.8, 4.0 Hz).

^{19}F-NMR (282 MHz, C_6D_6) δ = -102.4 (ddt, J = 8.8, 6.9, 2.0 Hz), -103.5 (dddt, J = 14.0, 8.5, 5.2, 1.7 Hz), -123.0 (ddt, J = 21.9, 9.1, 1.7 Hz), -142.6 (dddd, J = 21.9, 15.8, 9.4, 3.3 Hz).

^{10}B-NMR (54 MHz, C_6D_6) δ = 64.2.

HRMS (ESI-/Q-TOF): Calculated for $[M+OH]^-$ = $[C_{18}H_8OBBrF_7]^-$: m/z 461.97816; Found: m/z 461.97884

Compound 1g: (2-bromo-6-fluorophenyl)bis(2,4,6-trifluorophenyl)borane

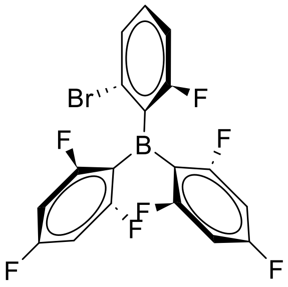

1g

Compound **1g** was prepared according to General procedure B1, starting from **S4** on a 71.2 mmol scale, yielding 22.2 g (49.8 mmol) of pure product, which corresponds to an overall yield of 70%. (See NMR spectra)

Physical state: white powder

^{1}H-NMR (500 MHz, C_6D_6) δ = 7.03 (dd, J = 7.8, 1.0 Hz, 1H), 6.67 – 6.56 (m, 2H), 6.12 – 6.05 (m, 4H).

^{13}C-NMR (126 MHz, C_6D_6) δ = 167.4 (dt, J = 256.4, 17.3 Hz), 167.1 (ddd, J = 254.3, 14.6, 14.3 Hz), 162.8 (d, J = 244.4 Hz), 134.1 – 133.7 (br m), 131.9 (d, J = 8.9 Hz), 128.1 (d, J = 3.0 Hz), 123.7 (d, J = 10.0 Hz), 114.8 – 114.4 (br m), 113.8 (d, J = 23.5 Hz), 100.6 (ddd, J = 30.2, 24.9, 3.2 Hz).

^{19}F-NMR (282 MHz, C_6D_6) δ = -94.4 (ddt, J = 11.7, 9.7, 2.1 Hz), -98.7 (tt, J = 11.6, 8.9 Hz), -102.8 (ddt, J = 8.5, 6.5, 1.9 Hz).

^{10}B-NMR (54 MHz, C_6D_6) δ = 62.4.

HRMS (ESI-/Q-TOF): Calculated for $[M+OH]^-$ = $[C_{18}H_8OBBrF_7]^-$: m/z 461.97816; Found: m/z 461.97885

Compound 1h: (2-bromo-6-fluorophenyl)bis(2,6-difluorophenyl)borane

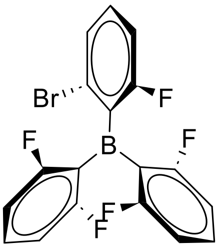

1h

Compound **1h** was prepared according to General procedure B1, starting from **S4** on a 17.8 mmol scale, yielding 2.92 g (7.10 mmol) of pure product, which corresponds to an overall yield of 40%. (See NMR spectra)

Physical state: white powder

^{1}H-NMR (500 MHz, C_6D_6) δ = 7.04 (dd, J = 7.9, 1.0 Hz, 1H), 6.72 (tt, J = 8.3, 6.5 Hz, 2H), 6.66 – 6.53 (m, 2H), 6.43 (t, J = 8.1 Hz, 4H).

^{13}C-NMR (126 MHz, C_6D_6, partial) δ = 166.2 (dd, J = 253.4, 11.2 Hz), 162.9 (d, J = 244.7 Hz), 136.1 (t, J = 11.7 Hz), 131.8 (d, J = 9.1 Hz), 128.1 (d, J = 3.3 Hz), 123.8 (d, J = 11.1 Hz), 113.8 (d, J = 23.4 Hz), 111.6 – 111.4 (m).

^{19}F-NMR (282 MHz, C_6D_6) δ = -97.7 (t, J = 7.1 Hz), -102.6 (t, J = 7.8 Hz).

^{10}B-NMR (54 MHz, C_6D_6) δ = 64.8.

HRMS (ESI-/Q-TOF): Calculated for $[M+OH]^-$ = $[C_{18}H_{10}OBBrF_5]^-$: m/z 425.99700; Found: m/z 425.99760

Compound S5: (2-chloro-6-fluorophenyl)bis(2,3,5,6-tetrafluorophenyl)borane

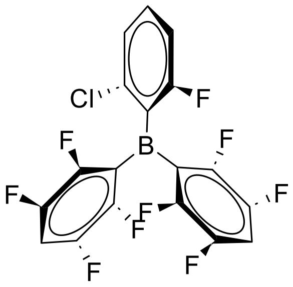

S5

Compound **S5** was prepared according to General procedure B1, starting from **S3** on a 13.7 mmol scale, yielding 2.15 g (4.89 mmol) of pure product, which corresponds to an overall yield of 36%. (See NMR spectra)

Physical state: white powder

^{1}H-NMR (500 MHz, C_6D_6) δ = 6.76 (d, J = 8.0 Hz, 1H), 6.61 (td, J = 8.2, 6.4 Hz, 1H), 6.50 (t, J = 8.6 Hz, 1H), 6.23 (ddd, J = 16.9, 9.4, 7.5 Hz, 2H).

^{13}C-NMR (75 MHz, C_6D_6) δ = 163.3 (d, J = 246.1 Hz), 148.2 (dddd, J = 251.0, 12.9, 8.7, 3.7 Hz), 146.1 (dm, J = 250.1 Hz), 136.1 (d, J = 9.8 Hz), 133.5 (d, J = 9.6 Hz), 129.8 – 129.0 (br m), 125.5 (d, J = 3.1 Hz), 120.4 – 119.4 (br m), 113.8 (d, J = 23.5 Hz), 111.6 (tt, J = 22.7, 2.0 Hz)

^{19}F-NMR (282 MHz, C_6D_6) δ = -101.9 (ddt, J = 8.4, 6.4, 1.7 Hz), -129.5 – -129.7 (m), -138.0 – -138.2 (m).

^{10}B-NMR (54 MHz, C_6D_6) δ = 64.6.

HRMS (ESI-/Q-TOF): Calculated for $[M+OH]^-$ = $[C_{18}H_6OBClF_9]^-$: m/z 454.00983; Found: m/z 454.01010

Compound S6: (2,6-dibromophenyl)bis(perfluorophenyl)borane

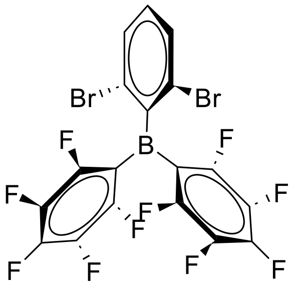

S6

Compound **S6** was prepared according to General procedure B1, starting from potassium (2,6-dibromophenyl)trifluoroborate on a 52.0 mmol scale, yielding 23.0 g (39.6 mmol) of pure product, which corresponds to an overall yield of 76%. (See NMR spectra)

Physical state: white powder

^{1}H-NMR (500 MHz, C_6D_6) δ = 7.03 (d, J = 8.1 Hz, 2H), 6.40 (t, J = 8.1 Hz, 1H)

^{13}C-NMR (126 MHz, C_6D_6) δ = 149.6 (d, J = 251.2 Hz), 145.3 (br s), 145.3 (d, J = 261.8 Hz), 137.7 (d, J = 252.1 Hz), 132.0, 130.3, 123.0, 112.8 (br s).

^{19}F-NMR (282 MHz, C_6D_6) δ = -127.4 (dt, J = 20.7, 6.4 Hz), -143.0 (tt, J = 20.9, 7.2 Hz), -160.6 – -161.0 (m).

^{10}B-NMR (54 MHz, C_6D_6) δ = 60.6.

HRMS (ESI-/Q-TOF): Calculated for $[M+OH]^-$ = $[C_{18}H_4OBBr_2F_{10}]^-$: m/z 593.86041; Found: m/z 593.86098

1.2.5 Large-scale laboratory synthesis of **1d**

As a demonstration of the scalability of the synthesis of fiddler crab-type boranes (General procedure B2), a large-scale laboratory synthesis was demonstrated on a 286 mmol scale. It is noteworthy, that the yield and selectivity of the synthesis improved in larger-scale reactions, even though the inertness of the reaction was harder to maintain on this scale.

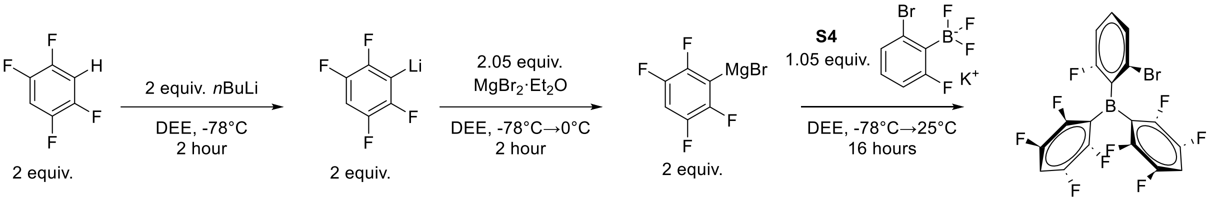

1d: 76%
[105 g]

In a preheated 1000 mL 4-necked flask, equipped with mechanical stirring, a nitrogen gas inlet and an immersion probe digital thermometer, the respective 1,2,4,5-tetrafluorobenzene (85.8 g, 63.8 mL, 2.0 equiv., 571 mmol) was measured in and dissolved in 250 mL anhydrous diethyl ether (DEE) [Note 1.]. The solution was cooled to -78°C (using an acetone/dry ice bath), after which *n*-butyllithium (36.6 g, 229 mL, 2.5 M in hexanes, 2.0 equiv., 571 mmol) was added dropwise (using a pressure compensated dropping funnel) over 2 hours, keeping the temperature below -70°C [Note 2.]. After completion of the addition, the mixture was maintained at -78°C for further 2 hours [Note 3.]. Then solid $MgBr_2 \cdot Et_2O$ (151 g, 2.05 equiv., 586 mmol) [Note 4.] was added portionwise, maintaining the temperature below -70°C. After completing the addition, the reaction mixture was stirred for another 2 hours at 0°C (using an ice bath) [Note 5.]. After that, the suspension was cooled to -78°C (using an acetone/dry ice bath) and the aryl trifluoroborate salt **S4** (84.3 g, 1.05 equiv., 300 mmol) was added to it in one portion. The reaction mixture was stirred for another 15 min. at a temperature of -78°C, after which it was left to warm up in the acetone/dry ice cooling bath overnight and stirred for a total of 16 hours [Note 6.]. Next day, 20 mL trimethylsilyl chloride (TMSCl, 17 g, 0.55 equiv., 0.16 mol) was added to the reaction mixture and it was stirred for another 2 hours [Note 7.]. Next, the reaction was transferred to a 2000 mL flask, followed by the addition of 500 mL anhydrous toluene. After this, the diethyl ether was evaporated under reduced pressure (using a rotary evaporator) [Note 8.]. Then 60 mL anhydrous hexanes was added to the flask, to facilitate the precipitation of $MgBr_2$.The resulting precipitate was filtered off (using inert filtration, through a Celite layer [Note 9.]) and washed with additional 2x50 mL anhydrous toluene. The resulting filtrate was concentrated to about 100 mL solvent volume under reduced pressure (using a rotary evaporator once again), after which 400 mL anhydrous hexanes was added, resulting in the precipitation of an off-white solid. The resulting suspension was cooled to -78°C (using an acetone/dry ice bath; sometimes the precipitation only starts after cooling the reaction mixture) and the solid was filtered using the cannula filtering technique [Note 10.]. The solids were washed with additional 2x100 mL anhydrous hexanes. Finally, the obtained solid was dried for 2 hours under reduced pressure (using the vacuum line connected straight to a Vacuubrand RZ 6 rotary vane pump, and a 60°C water bath), to give 105 g (217 mmol) of **1d** as a white crystalline powder, equaling to a yield of 76%. [Note 11.] (See compound data and NMR spectra)

[Note 1.] Diethyl ether cannot be substituted with tetrahydrofuran (THF) in this reaction because it would coordinate strongly to the borane, making purification cumbersome.
[Note 2.] A white precipitate started to appear around the middle of the addition sequence.
[Note 3.] The progress of the lithiation can be monitored using GC-MS by quenching a small sample of the reaction with CH_3OD.

[Note 4.] $MgBr_2 \cdot Et_2O$ was freshly prepared: to activated magnesium turnings (14.2 g, 2.05 equiv., 586 mmol) 100 mL anhydrous diethyl ether was added, followed by the dropwise addition of 1,2-dibromoethane (110 g, 50.5 mL, 2.05 equiv., 686 mmol). After the Grignard reaction started (the solution started to warm up and reflux), 150 mL of diethyl ether was added to dilute the reaction, and dropwise addition of 1,2-dibromoethane was continued to maintain reflux. After the addition is finished and the boiling of the reaction subsides, the flask is heated to maintain reflux for 1 hour (or until most of the magnesium turnings disappear). After completion of the reaction, the resulting solution is evaporated to dryness under reduced pressure on a rotary evaporator to give the $MgBr_2 \cdot Et_2O$.
[Note 5.] During the addition, the suspension solidified almost completely. Additional 50 mL anhydrous diethyl ether may be added to the mixture if the mechanical stirrer struggles with the suspension.
[Note 6.] The progress of the reaction can be monitored using HPLC-MS.
[Note 7.] TMSCl is used to quench any remaining organometallic reagent. This step is crucial for the subsequent filtration step, as the suspension to be filtered won't be as gelatinous and won't clog the apparatus.
[Note 8.] Removal of diethyl ether is important, because it can solubilize $MgBr_2$ to some extent, which can lead to inorganic impurities in the final product. To facilitate this step, mark the 500 mL liquid level on the Schlenk flask before use and remove solvents until the liquid level of the reaction mixture reaches this mark. Alternatively, removal of some toluene also ensures efficient removal of residual diethyl ether. To keep the inertness of the reaction, the vent inlet of the rotary evaporator was connected to nitrogen gas.
[Note 9.] The easiest way to carry out this step is to use a large Schlenk filter equipped with a glass frit, but alternatively the suspension can be filtered on a regular glass frit filter too under a stream of nitrogen (an upside-down funnel is placed above the filter to direct the flow of nitrogen onto the filter). Besides the glass filter, the Celite used for filtration was also oven-dried at 140°C for at least 8 hours.
[Note 10.] This step can also be conducted using a Schlenk filtering apparatus, but the cannula filtration technique is preferred. A short and narrow oven-dried glass tube (that fits through the Shlenk flask's neck and joint) is connected to the end of a cannula and secured using PTFE tape. Then, a filter paper is attached to the other end of the glass tube and secured using PTFE tape, forming a plain filtering surface. Finally, the other end of the cannula is routed through a rubber seal, so it can be fitted onto the Schlenk flask. This filtering "probe" can be immersed into the suspension to remove the liquids by connecting the other end of the cannula to an empty flask with vacuum. This technique is preferred because it leaves the final product inside a Schlenk flask that is easily transferable to a glovebox. Also, after filtering the product, the Schlenk flask can be sealed again, and the solids can be dried in vacuum to remove any solvent residues.
[Note 11.] After filtration, the flask containing the catalyst was transferred into a glovebox under vacuum. All subsequent manipulations involving the solid catalyst in its pure form were carried out inside the glovebox.

1.2.6 Relative Lewis acidity of the prepared catalysts according to the Gutmann–Beckett method

The relative Lewis acidity (RLA) of the prepared catalysts was determined using ^{31}P-NMR according to the Gutmann-Beckett method[73].

In a glovebox, an NMR tube was charged with the respective borane (0.15 mmol) and Et_3PO (20.1 mg, 0.15 mmol, 1 equiv.) followed by the addition of dry C_6D_6 (0.75 mL). The NMR tube was sealed, and their ^{31}P-NMR spectrum was recorded on a Varian 500 MHz INOVA spectrometer. Using similar conditions, the ^{31}P-NMR spectrum of the pure Et_3PO (0.15 mmol in 0.75 mL C_6D_6) was also recorded. The spectra were externally referenced to H_3PO_4 (85%). The $\delta(^{31}P)$ NMR shifts were determined and the $\Delta\delta(^{31}P)_{adduct}$ values were calculated according to the formula $\Delta\delta(^{31}P)_{adduct} = \delta(^{31}P)_{adduct} - \delta(^{31}P)_{Et3PO}$. Relative Lewis acidity was determined relative to **1a** according to the formula $\Delta\delta(^{31}P)_{adduct}/\Delta\delta(^{31}P)_{\mathbf{1a}\text{-adduct}}$. The results are summarized in Table S1.

Table S1. Relative Lewis acidity according to the Gutmann-Beckett method[73]

Species	$\delta(^{31}P)$ (ppm)	$\Delta\delta(^{31}P)_{adduct}$ (ppm)	Relative Lewis acidity (%)
Et_3PO	45.46	-	-
**1a**-Et_3PO	75.12	29.66	100
**1b**-Et_3PO	72.98	27.52	93
**1c**-Et_3PO	72.48	27.02	91
**1d**-Et_3PO	72.91	27.45	93
**1e**-Et_3PO	73.54	28.08	95
**1f**-Et_3PO	71.39	25.93	87
**1g**-Et_3PO	70.15	24.69	83
**1h**-Et_3PO	69.84	24.38	82

1.3 Optimalization of the Hydrosilylation Reaction

The borane catalyst, silane employed and reaction conditions were optimized to maximize yield in the partial reduction of the esters via the FLP catalyzed hydrosilylation reaction. Moreover, throughout the optimalization, a great attention was paid to the selectivity of the reaction to minimize overreduction. As a model substrate, methyl 3-phenylpropanoate (**2**) was chosen, and the reactions were monitored using ^{1}H q-NMR.

<u>1.3.1 General procedure C for optimalization of the hydrosilylation reaction</u>

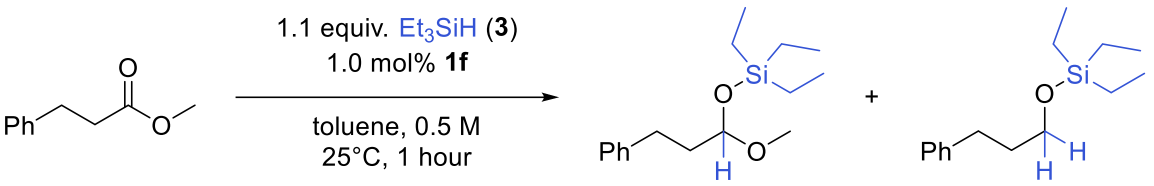

2 **4** **5**

The optimalization reactions were conducted in a glovebox on a 0.5 mmol scale, unless noted otherwise. In a 4 mL screw-capped vial, equipped with a magnetic stirrer, methyl 3-phenylpropanoate (**2**, 82.1 mg, 78.7 µL, 1.0 equiv., 0.500 mmol) was measured in, followed by the addition of 400 µL anhydrous toluene [Note 1.]. After that, a stock solution of (2-bromo-6-fluorophenyl)bis(2,3,6-trifluorophenyl)borane (**1f**, 2.32 mg, 100 µL, 0.05 M in anhydrous toluene, 0.01 equiv., 5.00 µmol) was added, followed by the addition of a stock solution of hexamethylbenzene (**S7**, 10.1 mg, 500 µL, 0.125 M in anhydrous toluene, 0.125 equiv., 62.5 µmol) which is used as an internal standard. Finally, triethylsilane (**3**, 64.0 mg, 87.8 µL, 1.1 equiv., 550 µmol) was added. The reaction was stirred for 1 hour, after which a small sample was taken from the reaction mixture (15 µL) and diluted with 670 µL C_6D_6 for ^{1}H q-NMR analysis. Based on the ^{1}H q-NMR spectra, the conversion (%) of the substrate as well as the yield (%) of the desired product **4** and the yield (%) of the overreduced side product **5** were calculated.

[Note 1.] Alternatively, C_6D_6 can be used, if the use of toluene is unsuitable due to spectral overlaps in the ^{1}H q-NMR spectra.

1.3.2 Catalyst screening

A large variety of borane catalysts were screened for the selective hydrosilylation of esters, a selection of which is seen in Fig. S2. This included commercially available catalysts, such as the tris(pentafluorophenyl)borane (BCF, **1a**), boranes previously prepared in our research group (**S8**[35], **S9**[40], **S10**[74], **S11**[40]), boranes previously known to art (**1b**[75], **S12**[76]), novel boranes (**1c**, **S6**), and the fiddler crab-type boranes (**1d**, **1e**, **1f**, **1g**, **1h, S5**). During this screening, to evaluate the robustness of our procedure and highlight the difference in selectivity between the catalysts, a slight excess of TESH (**3**) reducing agent was used (1.1 equiv.) with a comparably high catalyst loading of 1.0 mol%. The results are summarized in Table S2. The fiddler crab-type boranes, especially **1d** and **1f** outperformed the other catalysts in respect to both yield and selectivity (Entries 4 and 6). It's noteworthy that in the case of catalysts **1a**, **1b**, **1e**, **S5**, **S12** (Entries 1, 2, 5, 9, and 15) there is a significant difference between conversion and the sum of the yields **4** and **5**, meaning that there are further reductive side reactions happening besides the overreduction to **5**.

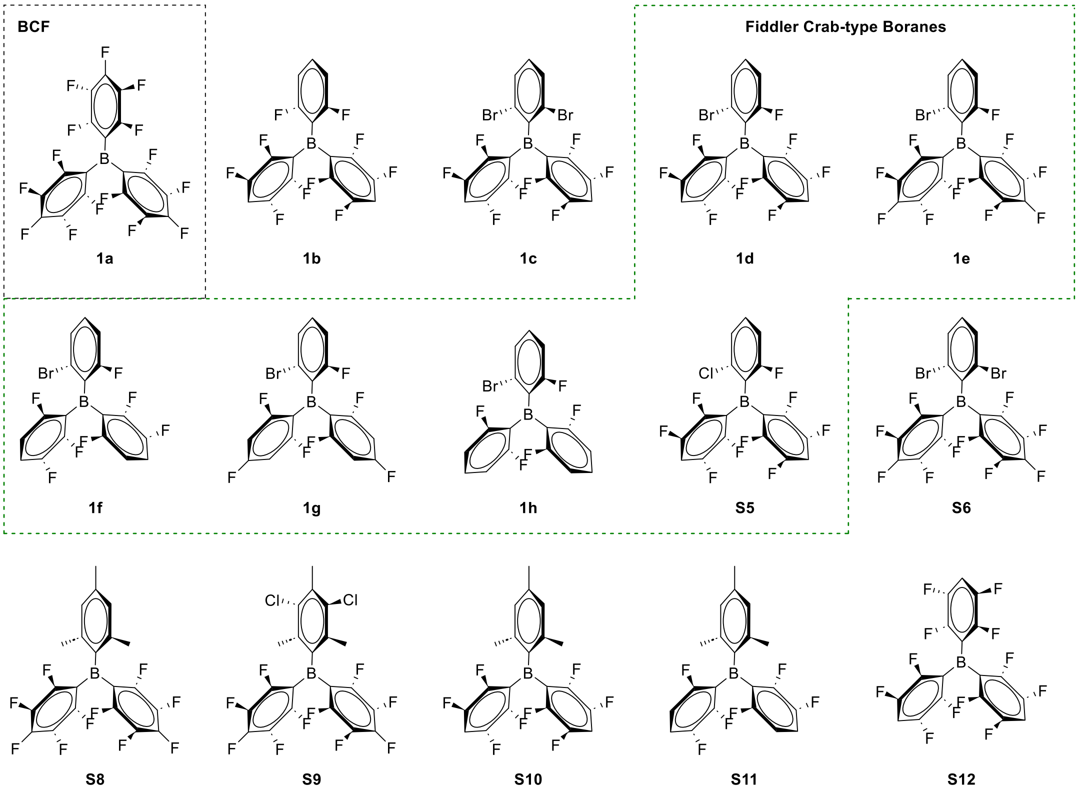

Fig. S2. Catalysts screened for the partial hydrosilylation of esters

Table S2. Catalyst screening

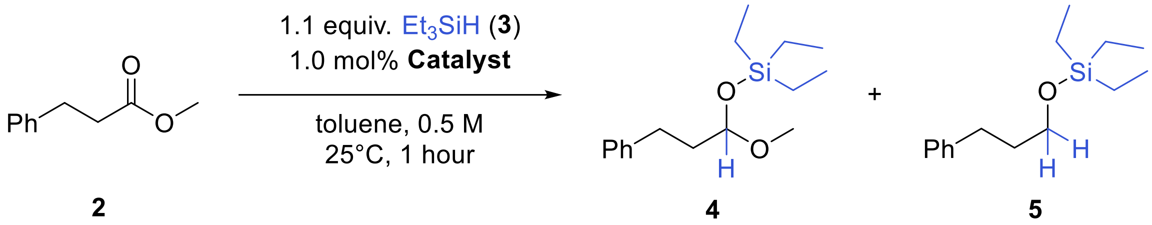

Entry	Catalyst	Conversion (%)	Yield of 4 (%)	Yield of 5 (%)
1	**1a**	90	59	9.3
2	**1b**	99+	88	5.6
3	**1c**	9	8	0.0
4	**1d**	99+	98	0.9
5	**1e**	99+	84	4.2
6	**1f**	84	84	0.0
7	**1g**	5	5	0.0
8	**1h**	1	1	0.0
9	**S5**	99+	88	1.1
10	**S6**	16	8	0.4
11	**S8**	10	3	0.0
12	**S9**	11	0	0.0
13	**S10**	6	0	0.0
14	**S11**	6	1	0.0
15	**S12**	99+	82	2.7

Experiments were executed according to the General procedure C. Conversion and yields were determined by ^{1}H q-NMR, using hexamethylbenzene (**S7**) as an internal standard.

1.3.3 Solvent screening

The FLP-catalyzed hydrosilylation reaction is incompatible with protic solvents both due to their side reaction with the silane reducing agents, and due to their (or their conjugate base's) increased affinity to form dative adducts with the borane catalysts. So, a variety of aprotic solvents (both polar and apolar) were screened, as seen in Table S3. Toluene, chloroform, hexane and diethyl ether were suitable solvents for the reaction. Acetonitrile inhibited the catalyst completely, while tetrahydrofuran and acetone were both reduced during the reaction, with no useful conversion of the starting material **2**. Interestingly, the use of dichloromethane led to significant overreduction,

Table S3. Solvent screening

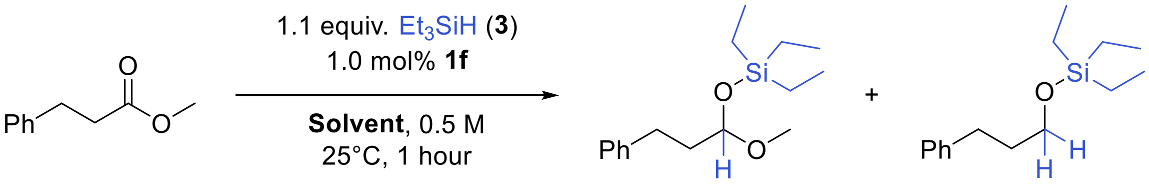

2 **4** **5**

Entry	Solvent	Conversion (%)	Yield of 4 (%)	Yield of 5 (%)
1	toluene	84	84	0.0
2	chloroform	80	78	0.0
3	dichloromethane	84	57	24
4	acetonitrile[a]	0	0	0.0
5	tetrahydrofuran[b]	0	0	0.0
6	acetone[b]	0	0	0.0
7	hexane	65	65	0.0
8	diethyl ether	99+	97	0.0

Experiments were executed according to the General procedure C. Conversion and yields were determined by ^{1}H q-NMR, using hexamethylbenzene (**S7**) as an internal standard. For Entries 1-6 deuterated solvents were used for easier evaluation of the results. For entries 7-8 the solvents were removed under reduced pressure prior to the NMR analysis. [a]Acetonitrile forms a stabile dative adduct with the borane. [b]The solvent reacted during the reaction.

1.3.4 The effect of the excess of reducing agent

The effect of the excess of reducing agent on the performance of our methodology was evaluated. The results, summarized in Table S4, clearly show the robustness of the procedure to excess of reducing agent. Additionally, to achieve complete conversion, a slight excess of TESH was needed (1.1-1.2 equiv.).

Table S4. The effect of the excess of reducing agent

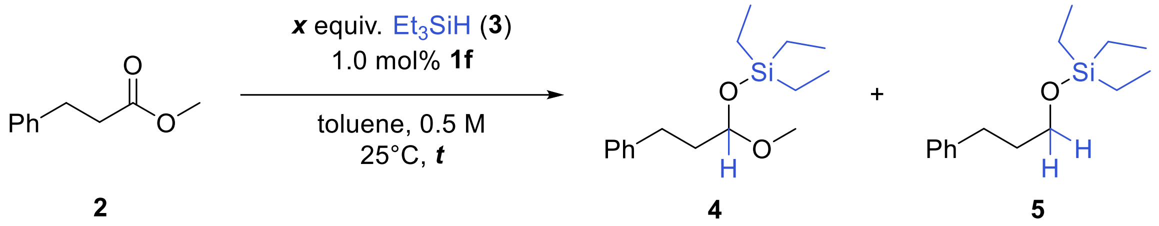

Entry	Et_3SiH equiv. (*x*)	Reaction time (*t* / hour)	Conversion (%)	Yield of 4 (%)	Yield of 5 (%)
1	0.9	1	71	70	0.0
2	1.0	1	74	74	0.0
3	1.1	1	84	84	0.0
4	1.2	1	85	84	0.0
5	1.5	1	92	91	0.0
6	3.0	1	99+	99+	0.0
7	0.9	24	89	89	0.0
8	1.0	24	96	96	0.0
9	1.1	24	99+	98	0.0
10	1.2	24	99+	99	0.0
11	1.5	24	99+	98	0.0
12	3.0	24	99+	99	0.6

Experiments were executed according to the General procedure C. Conversion and yields were determined by ^1H q-NMR, using hexamethylbenzene (**S7**) as an internal standard.

1.3.5 Screening the catalyst load and concentration

Various catalyst loadings and reaction concentrations were evaluated, as seen in Table S5. By increasing the reaction concentration, lower catalyst loadings can be employed.

Table S5. Screening the catalyst load and concentration

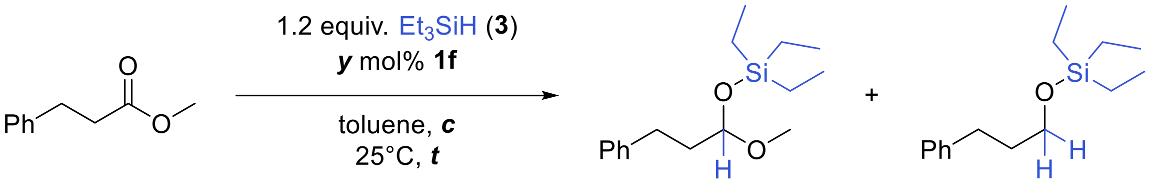

Entry	Catalyst load (*y* / mol%)	Concentration (*c* / M)	Reaction time (*t* / hour)	Conversion (%)	Yield of 4 (%)	Yield of 5 (%)
1	1.0	0.5	1	85	84	0.0
2	0.5	0.5	1	68	67	0.0
3	0.1	0.5	1	21	19	0.0
4	0.05	0.5	1	14	13	0.0
5	0.025	0.5	1	7	6	0.0
6	1.0	0.5	24	99+	99+	0.0
7	0.5	0.5	24	99+	99+	0.0
8	0.1	0.5	24	84	82	0.0
9	0.05	0.5	24	76	75	0.0
10	0.025	0.5	24	52	51	0.0
11	1.0	neat[a]	1	99+	99+	0.0
12	0.1	0.25	4	23	22	0.0
13	0.1	1	4	80	79	0.0
14	0.1	neat[a]	4	99+	99+	0.0
15	0.01	neat[a]	8	99+	99+	0.0

Experiments were executed according to the General procedure C. Conversion and yields were determined by ^{1}H q-NMR, using hexamethylbenzene (**S7**) as an internal standard. [a]In neat reactions, some solvent was still present because the catalyst was added as a solution.

1.3.6 Reaction temperature optimalization

The hydrosilylation reaction was conducted at different reaction temperatures, as seen in Table S6. There was no significant temperature dependence observed, although at higher temperatures the selectivity of the reaction started to deteriorate.

Table S6. Reaction temperature optimalization

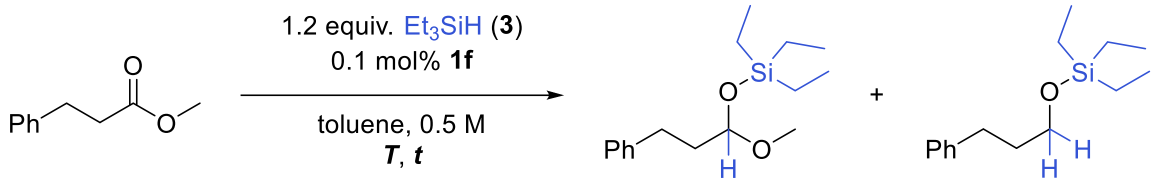

2 **4** **5**

Entry	Reaction temperature (*T* / °C)	Reaction time (*t* / hour)	Conversion (%)	Yield of 4 (%)	Yield of 5 (%)
1	0	4	60	58	0.0
2	25	4	58	54	0.0
3	40	4	54	48	0.0
4	60	4	41	33	0.0
5	0	24	98	98	0.0
6	25	24	84	83	0.0
7	40	24	88	85	0.0
8	60	24	78	68	0.0

Experiments were executed according to the General procedure C. Conversion and yields were determined by ^{1}H q-NMR, using hexamethylbenzene (**S7**) as an internal standard. The reactions were set up in a glovebox, after which they were immediately sealed and removed from the glovebox to be immersed in a temperature-controlled cooling/heating bath.

1.3.7 Screening of silane reducing agents

A variety of commercially available silanes (Fig. S3) were evaluated in the FLP catalyzed partial reduction of esters. These included the most used reducing agents, such as triethylsilane (TESH, **3**), 1,1,3,3-tetramethyldisiloxane (TMDS, **S13**), phenylsilane (**S14**), triethoxysilane (**S20**), triphenylsilane (**S21**), and polymethylhydrosiloxane (PMHS, **S22**). The results are summarized in Table S7. Out of these, only the use TESH (Entry 1) resulted in the selective reduction of the model substrate. Although acetal formation was observed using TMDS (Entries 1 and 2), and **S16** (Entry 6), their selectivity was inferior. Even so, by utilizing other catalysts, higher selectivity can be achieved in that case.

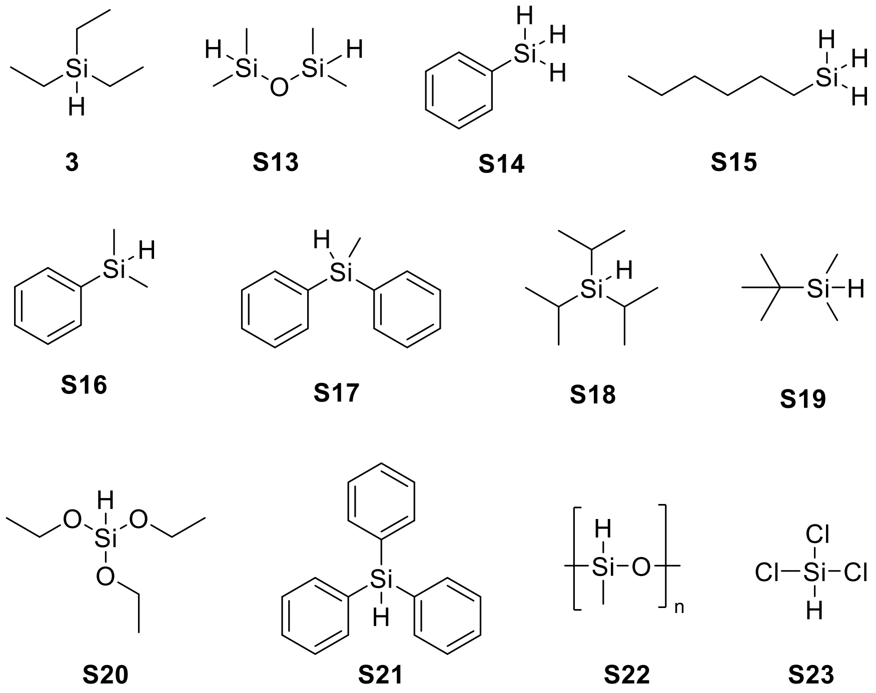

Fig. S3. Silanes screened for the partial hydrosilylation of esters

Table S7. Screening of silane reducing agents

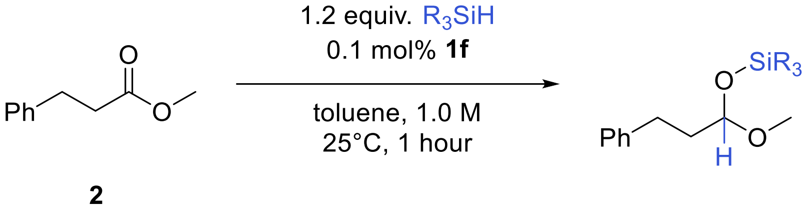

Entry	Silane	Observation
1	**3**	Selective reaction, 49 mol% acetal after 1 hour
2	**S13**	Full conversion, complex reaction mixture
3	**S13** (0.5 equiv.)	71% conversion, 40 % acetal, 22% overreduced side product
4	**S14**	Complex reaction mixture
5	**S15**	Complex reaction mixture
6	**S16**	81% conversion, 69% acetal, 11% overreduced side product
7	**S17**	No reaction observed
8	**S18**	No reaction observed
9	**S19**	No reaction observed
10	**S20**	No reaction observed
11	**S21**	No reaction observed
12	**S22**	No reaction observed
12	**S23**	No reaction observed

Experiments were executed according to the General procedure C. Conversion and yields were determined by ^{1}H q-NMR, using hexamethylbenzene (**S7**) as an internal standard. Due to several spectral overlaps and the complex reaction mixtures, these results are only indicative.

1.3.8 NMR study of the hydrosilylation reaction

An NMR study of the optimized hydrosilylation reaction was conducted to highlight the selectivity and efficiency of this new methodology.

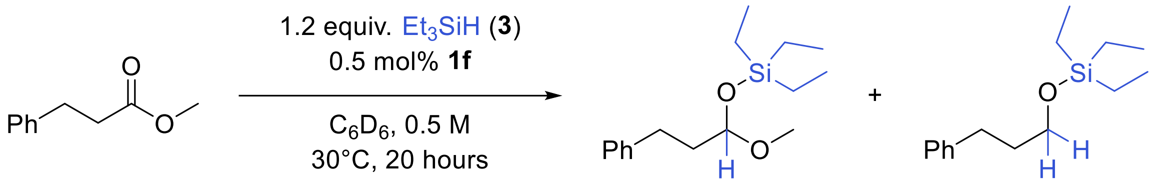

2 **4** **5**

A test reaction was set up in an NMR tube according to the General procedure C on a 0.375 mmol scale, using benzene-d[6] as a solvent, without any catalyst added. A starting ^{1}H spectrum (t = 0 min) was recorded using this sample on a Varian 500 MHz INOVA spectrometer. Next, the stock solution of the catalyst was added to the NMR sample (0.5 mol% of catalyst **1f**), it was mixed by shaking, and inserted back into the spectrometer. The reaction was monitored through 20 hours by measuring an ^{1}H-NMR spectrum every 1 hour. All throughout the experiment, the sample was kept at a temperature of 30°C and no mixing/stirring was applied.

^{1}H-NMR (500 MHz, C_6D_6) spectra of the hydrosilylation reaction (t = 1 hour steps, stacked)

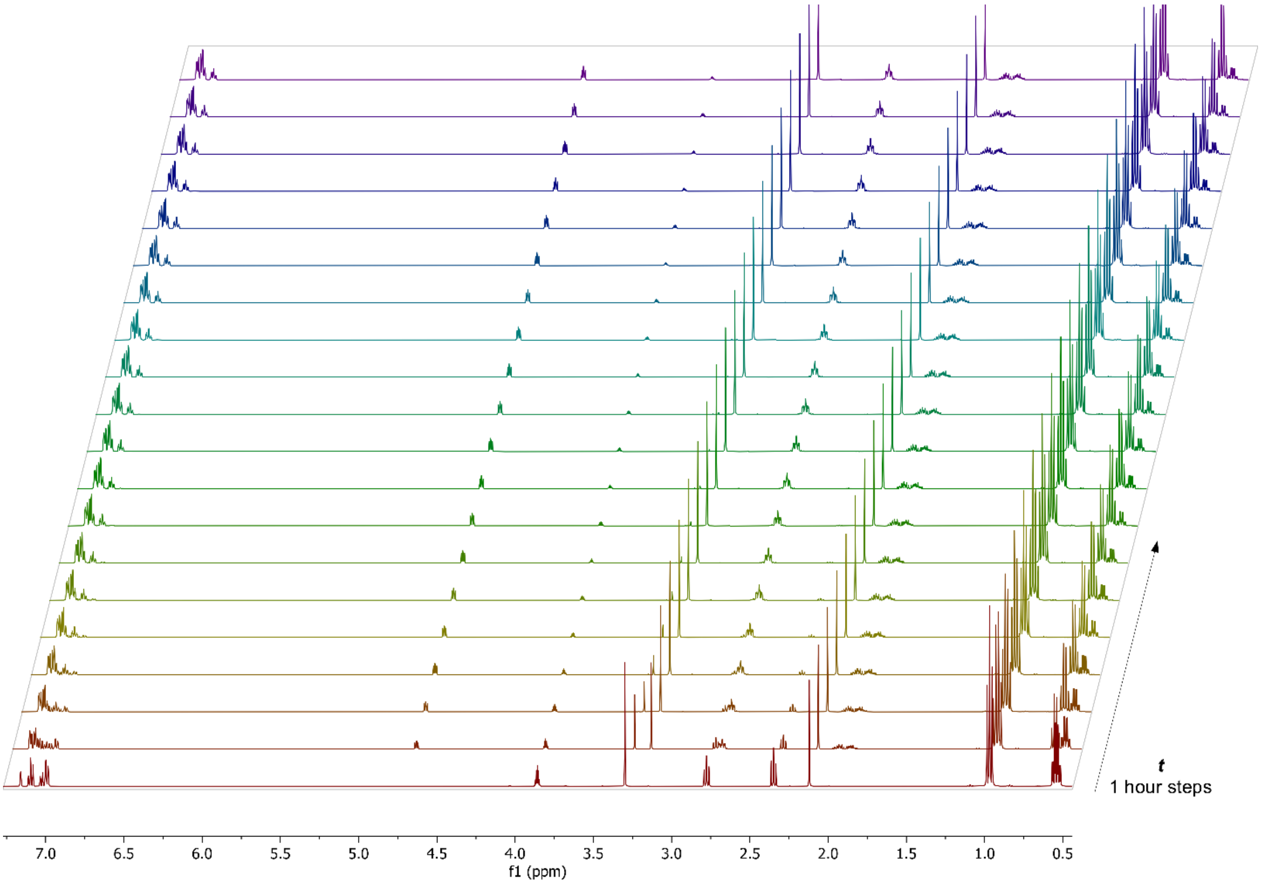

^{1}H-NMR (500 MHz, C_6D_6) spectra of the hydrosilylation reaction (t = 1 hour steps, superimposed)

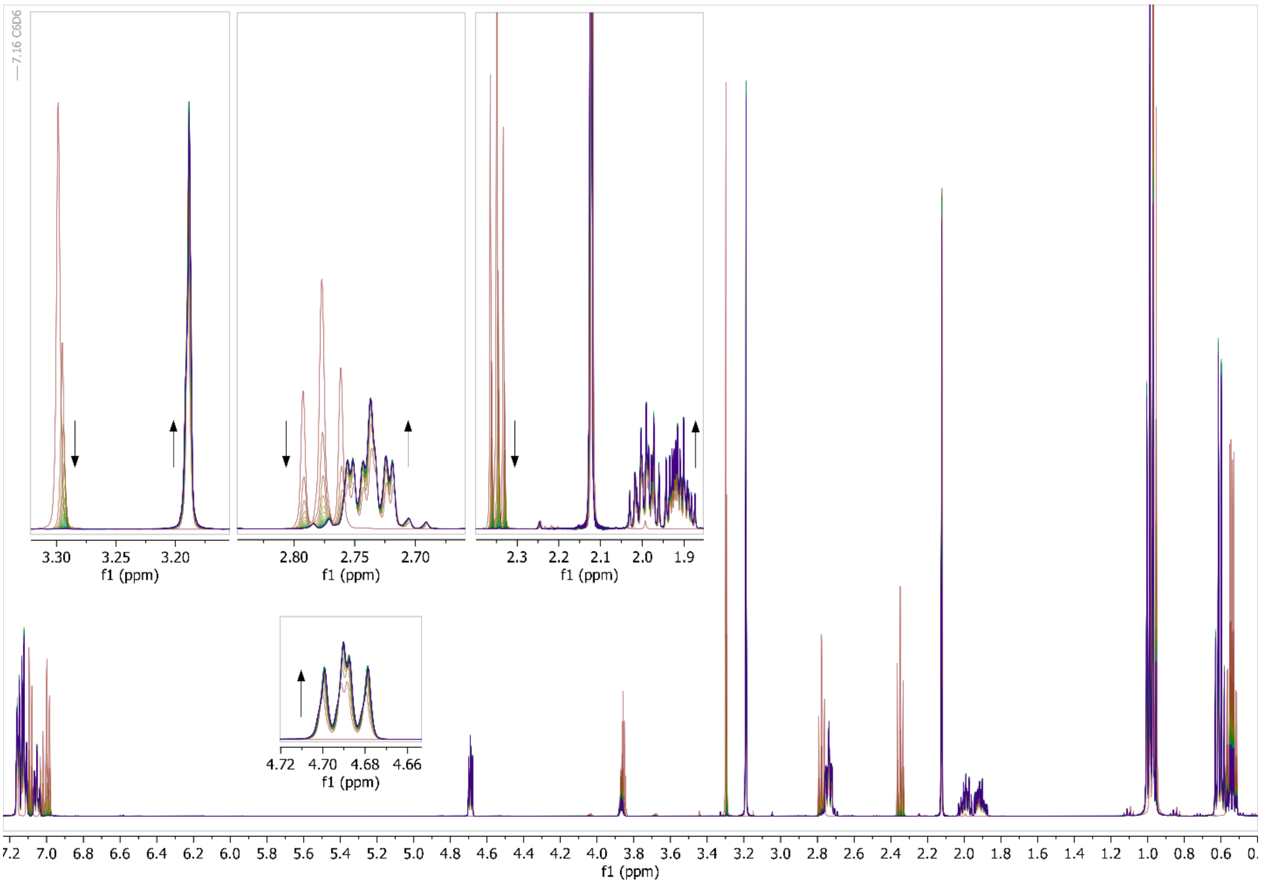

As can be seen from the ^{1}H-NMR spectra, the reaction achieved complete conversion within 16 hours, and a yield of 99%. Although an excess of TESH (**3**) was present, no detectable overreduction occurred.

In a similar experiment, 0.1 mol% of the less selective **1d** catalyst was used together with 2 equiv. of TESH (**3**) to induce a measurable amount of overreduction.

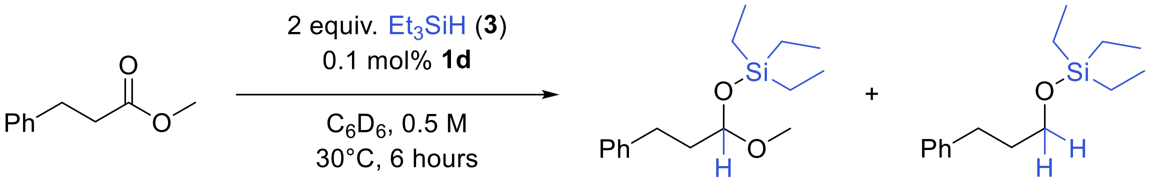

2 **4** **5**

A test reaction was set up in an NMR tube according to the General procedure C on a 0.375 mmol scale, using benzene-d[6] as a solvent, without any catalyst added. A starting ^{1}H spectrum (t = 0 min) was recorded using this sample on a Varian 500 MHz INOVA spectrometer. Next, the stock solution of the catalyst was added to the NMR sample (0.1 mol% of catalyst **1d**), it was mixed by shaking, and inserted back into the spectrometer. The reaction was monitored through 6 hours by measuring an ^{1}H-NMR spectrum every 2 minutes. All throughout the experiment, the sample was kept at a temperature of 30°C and no mixing/stirring was applied. By integrating the respective peaks and using hexamethylbenzene (**S7**) as an internal standard, the x mole fraction was determined for the ester starting material (**2**), acetal product (**4**), and the overreduced silyl ether product (**5**). The results of these preliminary kinetic experiments are shown in Fig. S4. The mole fraction of the silyl ether product has been magnified by a factor of 5x for better visibility.

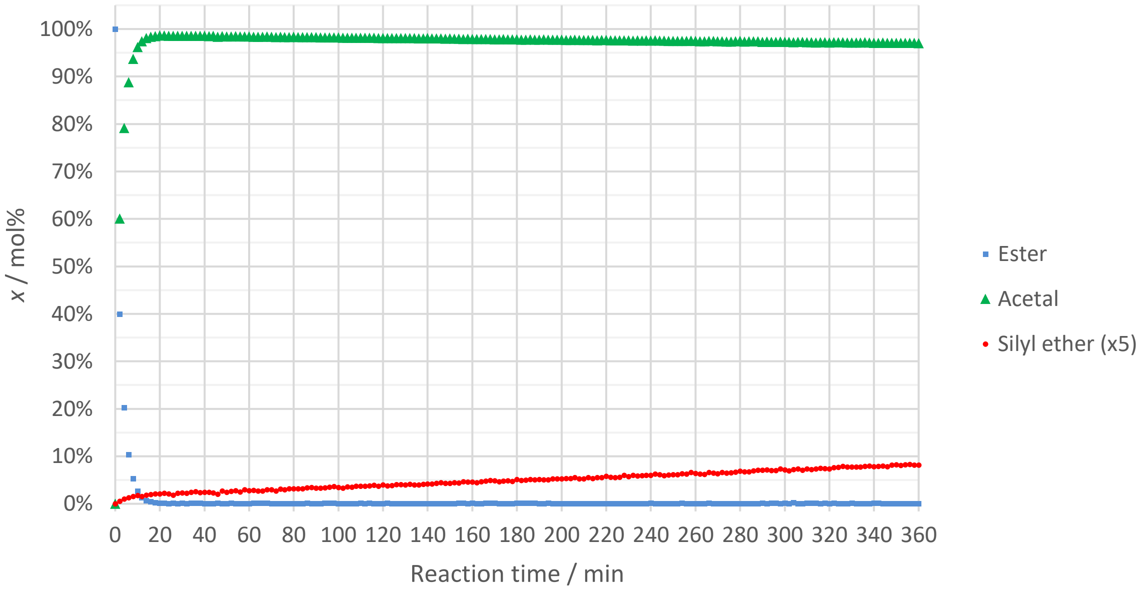

Fig. S4. Preliminary kinetic study of the overreduction using catalyst 1d

From these results it is evident that overreduction of the formed acetal starts almost immediately and continues throughout the monitored reaction time. Already after 2 minutes (at 60% conversion of ester **2**) there is 0.1 mol% of silyl ether (**5**) present. The amount of overreduced product continues to rise to 0.4 mol% as full conversion of the ester is reached at 16 minutes. Interestingly, after this point, the rate of overreduction significantly slows down, taking another 2 hours to once again double the amount of silyl ether present (to 0.8 mol%). This also supports our hypothesis, that overreduction happens faster through the silyl-transfer pathway while there is still ester starting material present in the system.

1.3.9 Kinetic models and preliminary studies

Based on previous mechanistic and kinetic studies by Piers[26,27], the Lewis acid catalyzed hydrosilylation reaction proceeds through pre-complexes. This pre-complex can either form between the borane and the silane (**model A**, the one favored in the case of catalyst **1a** as demonstrated by Piers), or between the borane and ester (**model B**). These two models and their effect on the reaction rate are demonstrated below.

Model A: Si–H activation via an Si-H···B encounter pre-complex

Symbols and approximations:

[B] = unbound, free borane catalyst concentration

[E] = ester concentration

[S] = triethyl silane concentration

K_d = dissociation constant for the inhibitory adduct

[EB] = borane-ester dative adduct concentration

[SB] = borane-silane encounter pre-complex concentration

[ES] = product concentration

$[B]_T$ = total catalyst concentration

$$[\mathrm{B}]_\mathrm{T} = [\mathrm{B}] + [\mathrm{EB}] + [\mathrm{SB}];\ \text{as } [\mathrm{SB}] \ll [\mathrm{EB}] \text{ or } [\mathrm{B}], \text{thus } [\mathrm{B}]_\mathrm{T} \approx [\mathrm{B}] + [\mathrm{EB}]$$

$[E]_T$ = total ester concentration

$$[\mathrm{E}]_\mathrm{T} = [\mathrm{E}] + [\mathrm{EB}];\ \text{as } [\mathrm{EB}] \ll [\mathrm{E}], \text{thus } [\mathrm{E}]_\mathrm{T} \approx [\mathrm{E}]$$

Mechanistic model:

i. Formation of an inhibitory adduct:

$$\mathrm{E} + \mathrm{B} \underset{k_{-1}}{\overset{k_1}{\rightleftharpoons}} \mathrm{EB}$$

$$K_\mathrm{d} = \frac{k_{-1}}{k_1} = \frac{[\mathrm{E}]_\mathrm{T}[\mathrm{B}]}{[\mathrm{EB}]} \Longrightarrow [\mathrm{B}] = [\mathrm{B}]_\mathrm{T} - [\mathrm{EB}] = \frac{K_\mathrm{d}[\mathrm{EB}]}{[\mathrm{E}]_\mathrm{T}} \Longrightarrow [\mathrm{EB}] = \frac{[\mathrm{B}]_\mathrm{T}[\mathrm{E}]_\mathrm{T}}{K_\mathrm{d} + [\mathrm{E}]_\mathrm{T}}$$

ii. Formation of a silane-borane encounter pre-complex (FLP):

$$\mathrm{S} + \mathrm{B} \underset{k_{-2}}{\overset{k_2}{\rightleftharpoons}} \mathrm{SB}$$

iii. Rate determining step:

$$\mathrm{SB} + \mathrm{E} \xrightarrow{k_3} \mathrm{ES} + \mathrm{B}$$

iv. Steady state assumption:

$$\frac{d[\mathrm{SB}]}{dt} \approx 0 \Longrightarrow k_2[\mathrm{S}][\mathrm{B}] = k_{-2}[\mathrm{SB}] + k_3[\mathrm{SB}][\mathrm{E}]_\mathrm{T} \Longrightarrow [\mathrm{SB}] = \frac{k_2[\mathrm{S}][\mathrm{B}]}{k_{-2} + k_3[\mathrm{E}]_\mathrm{T}}$$

Equations:

$$v_\mathrm{cat} = \frac{d[\mathrm{ES}]}{dt} = k_3[\mathrm{SB}][\mathrm{E}]_\mathrm{T} = k_3\frac{k_2[\mathrm{S}][\mathrm{B}]}{k_{-2} + k_3[\mathrm{E}]_\mathrm{T}}[\mathrm{E}]_\mathrm{T} = k_3k_2[\mathrm{S}][\mathrm{E}]_\mathrm{T}\frac{\frac{K_\mathrm{d}[\mathrm{EB}]}{[\mathrm{E}]_\mathrm{T}}}{k_{-2} + k_3[\mathrm{E}]_\mathrm{T}} =$$

$$= k_3k_2K_\mathrm{d}[\mathrm{S}]\frac{\frac{[\mathrm{B}]_\mathrm{T}[\mathrm{E}]_\mathrm{T}}{K_\mathrm{d} + [\mathrm{E}]_\mathrm{T}}}{k_{-2} + k_3[\mathrm{E}]_\mathrm{T}}$$

Two extreme cases:

$$a: K_\mathrm{d} \ll [\mathrm{E}]_\mathrm{T} \Rightarrow v_\mathrm{cat} \approx k_3 k_2 K_\mathrm{d} [\mathrm{S}] \frac{[\mathrm{B}]_\mathrm{T}}{k_{-2} + k_3 [\mathrm{E}]_\mathrm{T}}$$

$$b: K_\mathrm{d} \gg [\mathrm{E}]_\mathrm{T} \Rightarrow v_\mathrm{cat} \approx k_3 k_2 [\mathrm{S}] \frac{[\mathrm{B}]_\mathrm{T}[\mathrm{E}]_\mathrm{T}}{k_{-2} + k_3 [\mathrm{E}]_\mathrm{T}}$$

Model B: Si–H activation via an Ester···B FLP encounter pre-complex

Symbols and approximations:

[B] = unbound, free borane catalyst concentration

[E] = ester concentration

[S] = triethyl silane concentration

K_d = dissociation constant for the inhibitory adduct

[EB] = borane-ester dative adduct concentration

[EB'] = borane-ester encounter pre-complex (FLP) concentration

[ES] = product concentration

$[\mathrm{B}]_\mathrm{T}$ = total catalyst concentration

$$[\mathrm{B}]_\mathrm{T} = [\mathrm{B}] + [\mathrm{EB}] + [\mathrm{EB'}];\ \text{as } [\mathrm{EB'}] \ll [\mathrm{EB}] \text{ or } [\mathrm{B}], \text{thus } [\mathrm{B}]_\mathrm{T} \approx [\mathrm{B}] + [\mathrm{EB}]$$

$[\mathrm{E}]_\mathrm{T}$ = total ester concentration

$$[\mathrm{E}]_\mathrm{T} = [\mathrm{E}] + [\mathrm{EB}] + [\mathrm{EB'}];\ \text{as } [\mathrm{EB'}] \text{ and } [\mathrm{EB}] \ll [\mathrm{E}], \text{thus } [\mathrm{E}]_\mathrm{T} \approx [\mathrm{E}]$$

Mechanistic model:

i. Formation of an inhibitory adduct:

$$\mathrm{E} + \mathrm{B} \underset{k_{-1}}{\overset{k_1}{\rightleftharpoons}} \mathrm{EB}$$

$$K_\mathrm{d} = \frac{k_{-1}}{k_1} = \frac{[\mathrm{E}]_\mathrm{T}[\mathrm{B}]}{[\mathrm{EB}]} \Rightarrow [\mathrm{B}] = [\mathrm{B}]_\mathrm{T} - [\mathrm{EB}] = \frac{K_\mathrm{d}[\mathrm{EB}]}{[\mathrm{E}]_\mathrm{T}} \Rightarrow [\mathrm{EB}] = \frac{[\mathrm{B}]_\mathrm{T}[\mathrm{E}]_\mathrm{T}}{K_\mathrm{d} + [\mathrm{E}]_\mathrm{T}}$$

ii. Formation of an ester-borane encounter pre-complex (FLP):

$$\mathrm{E} + \mathrm{B} \underset{k_{-2}}{\overset{k_2}{\rightleftharpoons}} \mathrm{EB'}$$

iii. Rate determining step:

$$\mathrm{EB'} + \mathrm{S} \xrightarrow{k_3} \mathrm{ES} + \mathrm{B}$$

iv. Steady state assumption:

$$\frac{d[\mathrm{EB'}]}{dt} \approx 0 \Rightarrow k_2[\mathrm{E}]_\mathrm{T}[\mathrm{B}] = k_{-2}[\mathrm{EB'}] + k_3[\mathrm{EB'}][\mathrm{S}] \Rightarrow [\mathrm{EB'}] = \frac{k_2[\mathrm{E}]_\mathrm{T}[\mathrm{B}]}{k_{-2} + k_3[\mathrm{S}]}$$

Equations:

$$v_\mathrm{cat} = \frac{d[\mathrm{ES}]}{dt} = k_3[\mathrm{EB'}][\mathrm{S}] = k_3 \frac{k_2[\mathrm{E}]_\mathrm{T}[\mathrm{B}]}{k_{-2} + k_3[\mathrm{S}]}[\mathrm{S}] = k_3 k_2 [\mathrm{S}][\mathrm{E}]_\mathrm{T} \frac{\frac{K_\mathrm{d}[\mathrm{EB}]}{[\mathrm{E}]_\mathrm{T}}}{k_{-2} + k_3[\mathrm{S}]} =$$

$$= k_3 k_2 K_\mathrm{d} [\mathrm{S}] \frac{\frac{[\mathrm{B}]_\mathrm{T}[\mathrm{E}]_\mathrm{T}}{K_\mathrm{d} + [\mathrm{E}]_\mathrm{T}}}{k_{-2} + k_3[\mathrm{S}]}$$

Two extreme cases:

$$a: K_\mathrm{d} \ll [\mathrm{E}]_\mathrm{T} \Rightarrow v_\mathrm{cat} \approx k_3 k_2 K_\mathrm{d} [\mathrm{S}] \frac{[\mathrm{B}]_\mathrm{T}}{k_{-2} + k_3 [\mathrm{S}]}$$

$$b: K_d \gg [E]_T \Rightarrow v_{cat} \approx k_3 k_2 [S] \frac{[B]_T [E]_T}{k_{-2} + k_3[S]}$$

As can be seen from these equations, for very high or very low K_d values certain simplifications can be made. For example, in the case of model A and a low K_d (strong inhibitory adduct formation between borane and ester) the TOF of the catalyst becomes inversely proportional to the ester concentration, as was the case in the **1a** catalyzed hydrosilylation presented by Piers[26,27]. In the case of model B and a low K_d, the TOF of the catalysts should not change with the ester concentration. On the other hand, in the case of model A and high K_d values (weak inhibitory adduct formation between borane and ester), the catalyst TOF should be either proportional to $[E]_T$, or not be affected by it, depending on the relation of k_{-2} and $k_3[E]_T$. In the case of model B and high K_d, the catalyst TOF should be proportional to $[E]_T$.

To investigate these cases for our catalytic system, preliminary NMR kinetic studies were conducted similarly to the ones presented in section 1.3.8, using 0.1 mol% of catalyst **1f**.

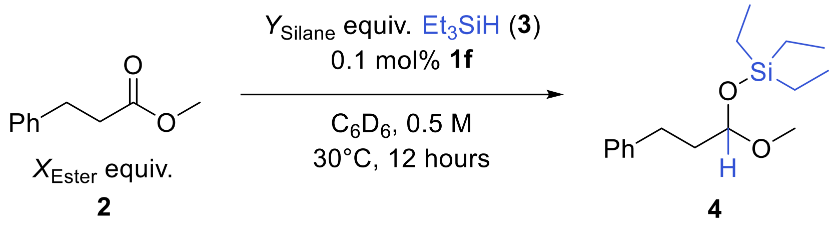

A test reaction was set up in an NMR tube according to the General procedure C on a 0.375 mmol scale, using benzene-d[6] as a solvent, without any catalyst added. A starting ^1H spectrum (t = 0 min) was recorded using this sample on a Varian 500 MHz INOVA spectrometer. Next, the stock solution of the catalyst was added to the NMR sample (0.1 mol% of catalyst **1f**), it was mixed by shaking, and inserted back into the spectrometer. The reaction was monitored through 12 hours by measuring an ^1H-NMR spectrum every 2 minutes. All throughout the experiment, the sample was kept at a temperature of 30°C and no mixing/stirring was applied. By integrating the respective peaks and using hexamethylbenzene (**S7**) as an internal standard, the x_{Acetal} yield was determined for the acetal product (**4**). No overreduction or other side reactions were observed. The experiment was repeated using X_{Ester}:Y_{Silane} equivalent ratios of 3:1, 1:1, and 1:3. The results of these preliminary kinetic experiments are shown in Fig. S5.

The obtained results show, that the initial rate of the reaction (and the TOF of the catalyst) increases with higher silane concentrations. Even more noteworthy is the fact that these rates also increase with higher ester concentration. This means, that in the case of the fiddler crab-type catalysts substrate inhibition is not an issue, allowing the use of higher ester concentrations and enabling even neat, solvent-free reaction conditions. Subsequently, the value of K_d must be relatively high for **1f**. A crude estimate based on NMR titration experiments with **2** put it in the order of $K_d \approx 5$ mol/dm^3. Thus, none of the above extreme cases can be used to simplify the rate law.

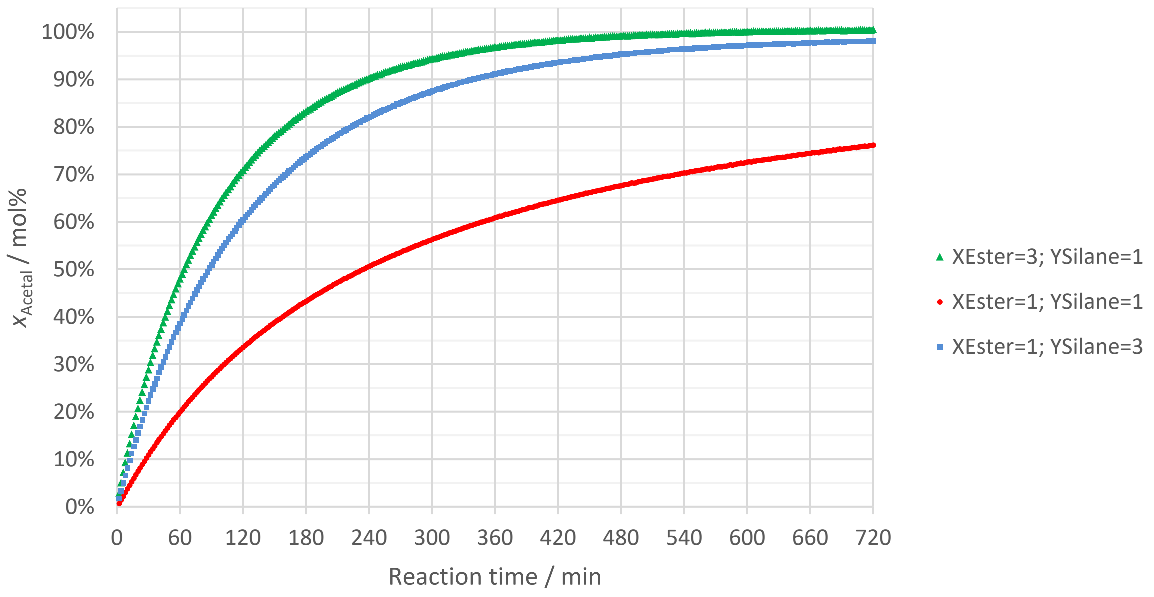

Fig. S5. Preliminary kinetic study of the hydrosilylation reaction using catalyst 1f

1.4 The Partial Reduction of Esters Using Fiddler Crab-Type Boranes

A variety of esters and lactones were reduced selectively using the novel fiddler crab-type boranes. Most of these reactions were promoted by the **1f** or **1d** boranes in the 0.05-1.0 mol% catalyst load range. In specific cases, a more Lewis acidic borane (i.e., **1e**) was used if the Lewis basicity of the substrate was significantly lower (for example in the case of ethyl trifluoroacetate). Conversely, for significantly more Lewis basic substrates (like lactones) a less Lewis acidic borane (i.e., **1h**) was used. In addition, a less Lewis acidic borane (i.e., **1g**) was needed for reactions that used TMDS as a reducing agent. Moreover, the catalyst load and/or the concentration of the reaction was fine-tuned for some substrates to ensure high selectivity and reasonable reaction rates.

1.4.1 General procedure D for the partial reduction of esters via hydrosilylation

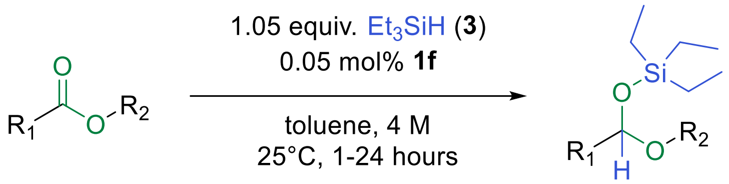

In a 4 mL oven-dried screw-capped vial equipped with a rubber seal and a magnetic stirrer, the ester substrate (5.0 mmol) is administered. The vial is capped, flushed with nitrogen or argon gas, and a nitrogen- or argon-filled balloon is connected to it [Note 1.]. Afterwards, 1.2 mL anhydrous toluene is added to the vial, followed by the addition of the **1f** catalyst solution (1.12 mg, 50.0 µL, 0.05 M in anhydrous toluene, 0.0005 equiv., 2.50 µmol) [Note 2.]. Finally, triethylsilane (**3**, 610 mg, 839 µL, 1.05 equiv., 5.25 mmol) is added [Note 3.], and the reaction mixture is stirred until complete conversion of the ester [Note 4.] is observed using TLC or NMR analysis [Note 5.]. After the reaction is complete, it is quenched by the addition of a few drops of acetonitrile or triethylamine [Note 6.]. Next, the mixture is filtered through a layer of silica gel, which is in turn washed with hexanes : ethyl acetate 10:1 (V/V) [Note 7.]. Finally, the reaction mixture is concentrated under reduced pressure using a rotary evaporator to give the crude silyl acetal product [Note 8.]. Optionally, the crude product can be further purified using flash chromatography on silica columns.

[Note 1.] The reaction is robust enough, that it doesn't need to be run in a glovebox (if there is a stock solution of the catalyst already prepared). It is usually sufficient to connect to the reaction a balloon filled with an inert gas to maintain some overpressure. If there is any residual water present in the reaction mixture, the catalyst will convert it to silanol using the excess silane, while hydrogen gas evolves. In these small-batch reactions this hydrogen evolution isn't a concern, but it should be taken in account during scale-up.
[Note 2.] This stock solution of the catalyst should be prepared in a glovebox, as the pure catalyst powder is hygroscopic and can decompose in a few minutes if exposed to moisture. Once the solution is prepared, it can be brought out from the glovebox in a vial equipped with a rubber seal and can be handled using standard inert syringe techniques. This stock solution can usually be used for 1-2 weeks after removal from the glovebox.
[Note 3.] In these small-batch reactions, the order of addition of these reagents is not important. Additionally, TESH can be added in one portion. Although the reaction is exotermic, it can quickly cool down to room temperature thanks to the larger surface/volume ration of these small-scale reactions. No difference in yield or selectivity was observed between these different addition modes. On the other hand, in large-scale reactions, a dropwise, controlled addition of the silane is required to control the temperature of the reaction and maintain high selectivity.

[Note 4.] In some cases, the reaction doesn't start right away, and a prolonged "induction period" is observed. This usually happens, when the moisture (or other protic solvent) content of the reaction mixture is higher, as they form stable adducts with the borane catalyst. In these cases, the free borane concentration is probably too low in the reaction mixture and additional time is needed for the catalyst to convert the water to silanol using the excess silane. Once enough catalyst becomes available, the reaction starts almost instantaneously, as indicated by the rapid evolution of heat and hydrogen gas. This should be taken into consideration during scale-up to prevent a runaway reaction.

[Note 5.] The reaction can be monitored by TLC, GC-MS or NMR analysis. The latter is usually preferred, as it can also indicate if a larger excess of silane is needed for complete conversion.

[Note 6.] Both acetonitrile and triethylamine quench the borane catalysts as they form stable adducts with them. This quenching step is required, because free boranes form a strong Brønsted acid with trace amounts of water during work-up and induce the hydrolysis of the acetal products (This was also observed in NMR samples too, if the moisture content of the NMR solvent was high.). In larger-scale reactions, the quenching of the excess silane is necessary before work-up. This can be achieved by adding ethyl acetate to the reaction mixture.

[Note 7.] During this filtration step, the catalyst is also removed from the reaction mixture. This way the crude silyl acetal product will be stable.

[Note 8.] During this final evaporation step, all solvents and the excess silane can be removed. The resulting crude product usually has a high purity (95+%) and can be used in further synthetic reactions without the need for any extra purification steps. The main contaminants of these crude products are triethylsilanol and hexaethyldisiloxane (resulting from excess moisture present in the reaction), which are mostly inert in subsequent reactions.

1.4.2 Scope of the partial reduction of esters using fiddler crab-type boranes

Compound 4: triethyl(1-methoxy-3-phenylpropoxy)silane

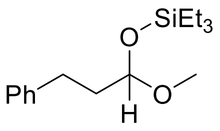

4

Compound **4** was prepared according to General procedure D, starting from methyl 3-phenylpropanoate (**2**) on a 5.00 mmol scale, yielding 1.40 g (4.98 mmol) product, which corresponds to a yield of 99+%. (See NMR spectra)

Physical state: clear liquid

^{1}H-NMR (500 MHz, $CDCl_3$) δ = 7.28 (t, *J* = 7.6 Hz, 2H), 7.20 – 7.16 (m, 3H), 4.71 (dd, *J* = 6.1, 4.1 Hz, 1H), 3.36 (s, 3H), 2.78 – 2.63 (m, 2H), 1.99 – 1.81 (m, 2H), 0.98 (t, *J* = 7.9 Hz, 9H), 0.64 (q, *J* = 8.0 Hz, 6H).

^{13}C-NMR (126 MHz, $CDCl_3$) δ = 142.0, 128.4, 128.3, 125.7, 98.5, 53.7, 38.9, 30.6, 6.8, 5.1.

HRMS (ESI-/Q-TOF): Calculated for $[M+Na]^+$ = $[C_{16}H_{28}NaO_2Si]^+$: m/z 303.1751; Found: m/z 303.1747

TLC: Rf: 0.61 using hexanes : ethyl acetate 20:1 (V/V) as an eluent; Visualization: $KMnO_4$

Compound 7: (1-ethoxy-3-phenylpropoxy)triethylsilane

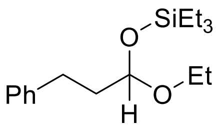

7

Compound **7** was prepared according to General procedure D, starting from ethyl 3-phenylpropanoate on a 5.00 mmol scale, yielding 1.47 g (5.00 mmol) product, which corresponds to a yield of 99+%. (See NMR spectra)

Physical state: clear liquid

^{1}H-NMR (500 MHz, $CDCl_3$) δ = 7.28 (t, *J* = 7.7 Hz, 2H), 7.20 – 7.16 (m, 3H), 4.78 (dd, *J* = 6.3, 4.1 Hz, 1H), 3.77 – 3.67 (m, 1H), 3.47 – 3.36 (m, 1H), 2.77 – 2.64 (m, 2H), 1.99 – 1.92 (m, 1H), 1.90 – 1.83 (m, 1H), 1.22 (td, *J* = 7.1, 0.7 Hz, 3H), 0.97 (t, *J* = 7.9 Hz, 9H), 0.67 – 0.59 (m, 6H).

^{13}C-NMR (126 MHz, $CDCl_3$) δ = 142.1, 128.4, 128.3, 125.7, 97.4, 61.9, 39.2, 30.7, 15.2, 6.8, 5.1.

HRMS (ESI-/Q-TOF): Calculated for $[M+Na]^+$ = $[C_{17}H_{30}NaO_2Si]^+$: m/z 317.1907; Found: m/z 317.1906

TLC: Rf: 0.57 using hexanes : ethyl acetate 20:1 (V/V) as an eluent; Visualization: $KMnO_4$

Compound 8: triethyl(1-isopropoxy-3-phenylpropoxy)silane

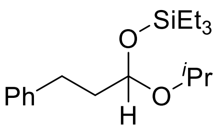

8

Compound **8** was prepared according to General procedure D, starting from isopropyl 3-phenylpropanoate on a 5.00 mmol scale, yielding 1.53 g (4.95 mmol) product, which corresponds to a yield of 99%. (See NMR spectra)

Physical state: clear liquid

^{1}H-NMR (500 MHz, C_6D_6) δ = 7.17 – 7.13 (m, 4H), 7.09 – 7.03 (m, 1H), 4.89 (dd, J = 6.3, 3.7 Hz, 1H), 3.84 (p, J = 6.1 Hz, 1H), 2.84 (ddd, J = 13.8, 9.9, 5.5 Hz, 1H), 2.76 (ddd, J = 3.8, 9.7, 6.7 Hz, 1H), 2.11 – 2.01 (m, 1H), 1.95 (dddd, J = 13.5, 10.1, 6.6, 3.7 Hz, 1H), 1.21 (d, J = 6.2 Hz, 3H), 1.05 (d, J = 6.1 Hz, 3H), 1.01 (t, J = 8.0 Hz, 9H), 0.63 (q, J = 7.8 Hz, 6H).

^{13}C-NMR (126 MHz, C_6D_6) δ = 142.6, 128.8, 128.7, 126.1, 95.7, 67.9, 40.6, 31.2, 23.9, 22.2, 7.2, 5.8.

HRMS (ESI-/Q-TOF): Calculated for $[M+Na]^+$ = $[C_{18}H_{32}NaO_2Si]^+$: m/z 331.20638; Found: m/z 331.20623

TLC: Rf: 0.61 using hexanes : ethyl acetate 20:1 (V/V) as an eluent; Visualization: $KMnO_4$

Compound 9: (1-(benzyloxy)-3-phenylpropoxy)triethylsilane

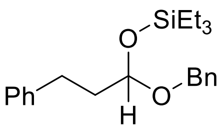

9

Compound **9** was prepared according to General procedure D, starting from benzyl 3-phenylpropanoate on a 2.00 mmol scale, using 0.75 mol% **1d** as a catalyst, yielding 672 mg (1.88 mmol) product, which corresponds to a yield of 94%. (See NMR spectra)

Physical state: clear liquid

^{1}H-NMR (500 MHz, $CDCl_3$) δ = 7.42 – 7.36 (m, 4H), 7.35 – 7.27 (m, 3H), 7.23 – 7.18 (m, 3H), 4.96 (dd, J = 6.4, 3.9 Hz, 1H), 4.79 (d, J = 11.8 Hz, 1H), 4.52 (d, J = 11.8 Hz, 1H), 2.83 (ddd, J = 15.2, 10.2, 5.4 Hz, 1H), 2.73 (ddd, J = 14.0, 10.1, 6.5 Hz, 1H), 2.08 (dddd, J = 13.6, 10.1, 6.4, 5.4 Hz, 1H), 1.97 (dddd, J = 13.8, 10.2, 6.5, 3.8 Hz, 1H), 1.02 (t, J = 8.0 Hz, 9H), 0.69 (q, J = 7.9 Hz, 6H).

^{13}C-NMR (126 MHz, $CDCl_3$) δ = 141.9, 138.5, 128.4, 128.32, 128.29, 127.6, 127.4, 125.7, 97.0, 68.1, 39.3, 30.7, 6.8, 5.2.

HRMS (ESI-/Q-TOF): Calculated for $[M+Na]^+$ = $[C_{22}H_{32}NaO_2Si]^+$: m/z 379.20638; Found: m/z 379.20703

TLC: Rf: 0.39 using hexanes : ethyl acetate 20:1 (V/V) as an eluent; Visualization: $KMnO_4$

Compound 10: 3,3,7,7-tetraethyl-5-phenethyl-4,6-dioxa-3,7-disilanonane

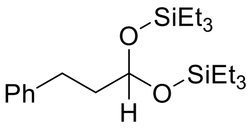

10

Compound **10** was prepared according to General procedure D, starting from *tert*-butyl 3-phenylpropanoate on a 5.00 mmol scale, using 0.1 mol% **1d** as a catalyst and 2.1 equiv. TESH (**3**), yielding 1.83 g (4.81 mmol) product, which corresponds to a yield of 96%. (See NMR spectra)

Physical state: clear liquid

^{1}H-NMR (500 MHz, $CDCl_3$) δ = 7.30 (t, J = 7.5 Hz, 2H), 7.24 – 7.17 (m, 3H), 5.24 (t, J = 4.8 Hz, 1H), 2.78 – 2.70 (m, 2H), 1.95 – 1.86 (m, 2H), 1.01 (t, J = 7.9 Hz, 18H), 0.67 (q, J = 8.0 Hz, 12H).

^{13}C-NMR (126 MHz, $CDCl_3$) δ = 142.2, 128.35, 128.32, 125.7, 92.6, 42.5, 30.8, 6.9, 5.3.

HRMS (ESI-/Q-TOF): Calculated for $[M+Na]^+$ = $[C_{21}H_{40}NaO_2Si_2]^+$: m/z 403.24590; Found: m/z 403.24590

TLC: Rf: 0.24 using hexanes : ethyl acetate 100:1 (V/V) as an eluent; Visualization: $KMnO_4$

Compound 11: triethyl(methoxy(phenyl)methoxy)silane

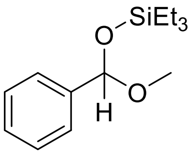

11

Compound **11** was prepared according to General procedure D, starting from methyl benzoate on a 5.00 mmol scale, using 0.05 mol% **1d** as a catalyst, yielding 1.10 g (4.36 mmol) product, which corresponds to a yield of 87%. (See NMR spectra)

Physical state: clear liquid

^{1}H-NMR (500 MHz, C_6D_6) δ = 7.55 (d, J = 7.3 Hz, 2H), 7.19 (t, J = 7.6 Hz, 2H), 7.14 – 7.09 (td, J = 7.4, 1.5 Hz, 1H), 5.80 (s, 1H), 3.14 (s, 3H), 0.98 (t, J = 8.0 Hz, 9H), 0.63 (q, J = 7.9 Hz, 6H).

^{13}C-NMR (126 MHz, C_6D_6) δ = 141.7, 128.5, 128.4, 126.9, 98.0, 51.6, 7.0, 5.4.

HRMS (ESI-/Q-TOF): Calculated for $[M+Na]^+$ = $[C_{14}H_{24}NaO_2Si]^+$: m/z 275.14378; Found: m/z 275.14322

TLC: Rf: 0.59 using hexanes : ethyl acetate 20:1 (V/V) as an eluent; Visualization: $KMnO_4$

Compound 12: triethyl(methoxy(p-tolyl)methoxy)silane

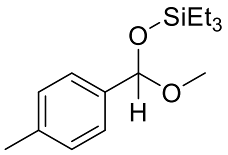

12

Compound **12** was prepared according to General procedure D, starting from methyl 4-methylbenzoate on a 5.00 mmol scale, using 0.1 mol% **1f** as a catalyst, yielding 1.29 g (4.84 mmol) product, which corresponds to a yield of 97%. (See NMR spectra)

Physical state: clear liquid

^{1}H-NMR (500 MHz, C_6D_6) δ = 7.49 (d, J = 8.0 Hz, 2H), 7.03 (d, J = 7.8 Hz, 2H), 5.83 (s, 1H), 3.18 (s, 3H), 2.11 (s, 3H), 1.00 (t, J = 8.0 Hz, 9H), 0.65 (q, J = 7.9 Hz, 6H).

^{13}C-NMR (126 MHz, C_6D_6) δ =138.9, 137.9, 129.1, 126.9, 98.1, 51.6, 21.2, 7.0, 5.4.

HRMS (ESI-/Q-TOF): Calculated for $[M+Na]^+$ = $[C_{15}H_{26}NaO_2Si]^+$: m/z 289.1594; Found: m/z 289.1597

TLC: Rf: 0.46 using hexanes : ethyl acetate 20:1 (V/V) as an eluent; Visualization: $KMnO_4$

Compound 13: triethyl(methoxy(m-tolyl)methoxy)silane

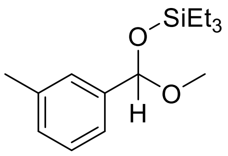

13

Compound **13** was prepared according to General procedure D, starting from methyl 3-methylbenzoate on a 5.00 mmol scale, using 0.1 mol% **1f** as a catalyst, yielding 1.21 g (4.54 mmol) product, which corresponds to a yield of 90%. (See NMR spectra)

Physical state: clear liquid

^{1}H-NMR (500 MHz, C_6D_6) δ = 7.41 (d, J = 7.2 Hz, 2H), 7.15 (t, J = 8.0 Hz, 1H), 6.97 (d, J = 7.5 Hz, 1H), 5.81 (s, 1H), 3.18 (s, 3H), 2.15 (s, 3H), 1.00 (t, J = 8.0 Hz, 9H), 0.66 (q, J = 7.9 Hz, 6H).

^{13}C-NMR (126 MHz, C_6D_6) δ = 141.7, 137.8, 129.2, 128.3, 127.7, 124.1, 98.2, 51.6, 21.4, 7.0, 5.4.

HRMS (ESI-/Q-TOF): Calculated for $[M+Na]^+$ = $[C_{15}H_{26}NaO_2Si]^+$: m/z 289.15943; Found: m/z 289.15937

TLC: Rf: 0.45 using hexanes : ethyl acetate 20:1 (V/V) as an eluent; Visualization: $KMnO_4$

Compound 14: triethyl(methoxy(o-tolyl)methoxy)silane

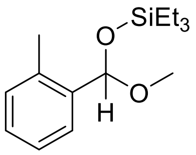

14

Compound **14** was prepared according to General procedure D, starting from methyl 2-methylbenzoate on a 5.00 mmol scale, using 0.15 mol% **1f** as a catalyst, yielding 1.19 g (4.47 mmol) product, which corresponds to a yield of 89%. (See NMR spectra)

Physical state: clear liquid

^{1}H-NMR (500 MHz, C_6D_6) δ = 7.70 (dd, J = 7.3, 1.8 Hz, 1H), 7.14 – 7.06 (m, 2H), 7.02 (dd, J = 6.6, 0.8 Hz, 1H), 5.92 (s, 1H), 3.13 (s, 3H), 2.39 (s, 3H), 0.98 (t, J = 7.9 Hz, 9H), 0.64 (q, J = 8.1 Hz, 6H).

^{13}C-NMR (126 MHz, C_6D_6) δ = 139.1, 136.3, 130.9, 128.5, 127.0, 125.7, 97.0, 51.8, 19.1, 7.0, 5.4.

HRMS (ESI-/Q-TOF): Calculated for $[M+Na]^+$ = $[C_{15}H_{26}NaO_2Si]^+$: m/z 289.15943; Found: m/z 289.15919

TLC: Rf: 0.45 using hexanes : ethyl acetate 20:1 (V/V) as an eluent; Visualization: $KMnO_4$

Compound 15: triethyl(methoxy(4-methoxyphenyl)methoxy)silane

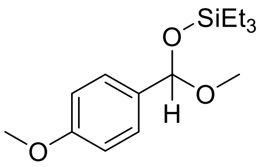

15

Compound **15** was prepared according to General procedure D, starting from methyl 4-methoxybenzoate on a 5.00 mmol scale, using 0.15 mol% **1h** as a catalyst, yielding 1.25 g (4.43 mmol) product, which corresponds to a yield of 88%. (See NMR spectra)

Physical state: clear liquid

^{1}H-NMR (500 MHz, C_6D_6) δ = 7.47 (d, J = 8.6 Hz, 2H), 6.80 (d, J = 8.7 Hz, 2H), 5.81 (s, 1H), 3.32 (s, 3H), 3.18 (s, 3H), 1.00 (t, J = 8.0 Hz, 9H), 0.65 (q, J = 7.9 Hz, 6H).

^{13}C-NMR (126 MHz, C_6D_6) δ = 160.2, 134.0, 128.1, 113.8, 97.9, 54.8, 51.5, 7.0, 5.4.

HRMS (ESI-/Q-TOF): Calculated for $[M+Na]^+$ = $[C_{15}H_{26}NaO_3Si]^+$: m/z 305.15434; Found: m/z 305.15531

TLC: Rf: 0.36 using hexanes : ethyl acetate 20:1 (V/V) as an eluent; Visualization: $KMnO_4$

Compound 16: triethyl(methoxy(3-(trifluoromethyl)phenyl)methoxy)silane

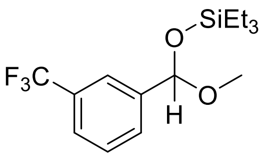

16

Compound **16** was prepared according to General procedure D, starting from methyl 3-(trifluoromethyl)benzoate on a 5.00 mmol scale, using 0.05 mol% **1d** as a catalyst, yielding 1.52 g (4.74 mmol) product, which corresponds to a yield of 95%. (See NMR spectra)

Physical state: clear liquid

^{1}H-NMR (500 MHz, C_6D_6) δ = 7.91 (s, 1H), 7.52 (d, J = 7.7 Hz, 1H), 7.31 (d, J = 7.7 Hz, 1H), 6.97 (t, J = 7.8 Hz, 1H), 5.66 (s, 1H), 3.04 (s, 3H), 0.94 (t, J = 8.0 Hz, 9H), 0.58 (q, J = 8.0 Hz, 6H).

^{13}C-NMR (126 MHz, C_6D_6) δ = 142.8, 130.8 (q, J = 32.2 Hz), 130.2, 128.9, 125.3 (q, J = 3.8 Hz), 125.0 (q, J = 272.0 Hz), 123.8 (q, J = 3.8 Hz), 97.1, 51.7, 6.9, 5.3.

^{19}F-NMR (282 MHz, C_6D_6) δ = -62.4.

HRMS (ESI-/Q-TOF): Calculated for $[M+Na]^+$ = $[C_{15}H_{23}F_3NaO_2Si]^+$: m/z 343.1312; Found: m/z 343.1307

TLC: Rf: 0.60 using hexanes as an eluent; Visualization: $KMnO_4$

Compound 17: ((1-ethoxydecyl)oxy)triethylsilane

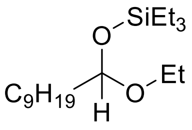

17

Compound **17** was prepared according to General procedure D, starting from ethyl decanoate on a 200 mmol scale, using 0.1 mol% **1f** as a catalyst, yielding 62.0 g (196 mmol) product, which corresponds to a yield of 98%. (See NMR spectra)

Physical state: clear oil

^{1}H-NMR (500 MHz, $CDCl_3$) δ = 4.75 (dd, J = 6.2, 4.4 Hz, 1H), 3.69 (dq, J = 9.1, 7.1 Hz, 1H), 3.41 (dq, J = 9.1, 7.0 Hz, 1H), 1.66 – 1.48 (m, 2H), 1.40 – 1.22 (m, 14H), 1.19 (t, J = 7.0 Hz, 3H), 0.98 (t, J = 8.0 Hz, 9H), 0.88 (t, J = 6.9 Hz, 3H), 0.68 – 0.60 (m, 6H).

^{13}C-NMR (126 MHz, $CDCl_3$) δ = 98.2, 61.6, 37.7, 31.9, 29.6, 29.53, 29.51, 29.3, 24.5, 22.7, 15.2, 14.1, 6.8, 5.1.

HRMS (ESI-/Q-TOF): Calculated for $[M+Na]^+$ = $[C_{18}H_{40}NaO_2Si]^+$: m/z 339.26898; Found: m/z 339.26999

TLC: Rf: 0.59 using hexanes : ethyl acetate 20:1 (V/V) as an eluent; Visualization: $KMnO_4$

Compound 18: 3,3,13,13-tetraethyl-5,11-dinonyl-8-((1-((triethylsilyl)oxy)decyl)oxy)-4,6,10,12-tetraoxa-3,13-disilapentadecane

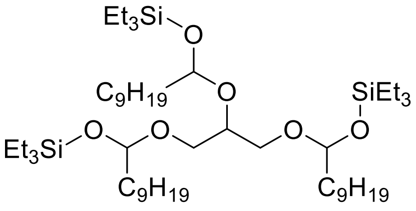

18

Compound **18** was prepared according to General procedure D, starting from propane-1,2,3-triyl tris(decanoate) on a 5.00 mmol scale, using 0.15 mol% **1f** as a catalyst and 3.3 equiv. TESH (**3**), yielding 4.50 g (4.98 mmol) product, which corresponds to a yield of 99+%. (See NMR spectra)

Physical state: pale yellow oil

Mixture of diastereomers

^{1}H-NMR (500 MHz, $CDCl_3$) δ = 5.01 – 4.94 (m, 1H), 4.81 – 4.71 (m, 2H), 3.88 – 3.57 (m, 3H), 3.52 – 3.26 (m, 2H), 1.64 – 1.45 (m, 6H), 1.41 – 1.20 (m, 42H), 1.01 – 0.93 (m, 27H), 0.88 (t, J = 6.8 Hz, 9H), 0.68 – 0.59 (m, 18H).

^{13}C-NMR (126 MHz, $CDCl_3$) δ = 98.92, 98.86, 98.82, 98.79, 98.74, 98.54, 98.42, 98.39, 98.21, 98.12, 97.99, 77.25, 77.00, 76.74, 74.61, 74.40, 74.21, 74.18, 68.60, 68.29, 67.09, 67.01, 66.90, 66.64, 38.37, 38.32, 38.25, 37.88, 37.87, 37.70, 37.61, 37.57, 37.56, 31.93, 31.92, 29.73, 29.71, 29.68, 29.68, 29.65, 29.64, 29.62, 29.38, 29.36, 24.51, 24.46, 24.44, 24.42, 24.39, 22.69, 14.07, 6.85, 6.81, 5.26, 5.25, 5.24, 5.18, 5.16, 5.15.

HRMS (ESI-/Q-TOF): Calculated for $[M+Na]^+$ = $[C_{51}H_{110}NaO_6Si_3]^+$: m/z 925.75024; Found: m/z 925.74932

TLC: Rf: 0.69 using hexanes : ethyl acetate 20:1 (V/V) as an eluent; Visualization: $KMnO_4$

Compound 19: (3-bromo-1-ethoxypropoxy)triethylsilane

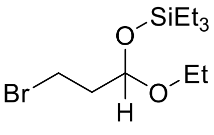

19

Compound **19** was prepared according to General procedure D, starting from ethyl 3-bromopropanoate on a 5.00 mmol scale, using 0.05 mol% **1d** as a catalyst, yielding 1.47 g (4.94 mmol) product, which corresponds to a yield of 99%. (See NMR spectra)

Physical state: clear liquid

^{1}H-NMR (500 MHz, $CDCl_3$) δ = 4.95 (dd, J = 6.1, 4.1 Hz, 1H), 3.71 (dq, J = 9.3, 7.1 Hz, 1H), 3.50 – 3.39 (m, 3H), 2.19 – 2.04 (m, 2H), 1.20 (t, J = 7.0 Hz, 3H), 0.98 (t, J = 7.9 Hz, 9H), 0.66 (q, J = 8.1 Hz, 6H).

^{13}C-NMR (126 MHz, $CDCl_3$) δ = 96.1, 62.3, 40.6, 28.9, 15.2, 6.7, 5.0.

HRMS (ESI-/Q-TOF): Calculated for $[M+Na]^+$ = $[C_{11}H_{25}BrNaO_2Si]^+$: m/z 319.06994; Found: m/z 319.06966

TLC: Rf: 0.31 using hexanes : ethyl acetate 20:1 (V/V) as an eluent; Visualization: $KMnO_4$

Compound 20: (4-bromo-1-ethoxybutoxy)triethylsilane

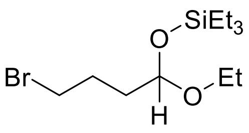
20

Compound **20** was prepared according to General procedure D, starting from ethyl 4-bromobutanoate on a 5.00 mmol scale, using 0.05 mol% **1d** as a catalyst, yielding 1.49 g (4.79 mmol) product, which corresponds to a yield of 96%. (See NMR spectra)

Physical state: clear liquid

^{1}H-NMR (500 MHz, $CDCl_3$) δ = 4.81 (dd, J = 5.6, 4.5 Hz, 1H), 3.69 (dq, J = 9.2, 7.1 Hz, 1H), 3.46 – 3.36 (m, 3H), 2.04 – 1.89 (m, 2H), 1.79 – 1.68 (m, 2H), 1.19 (t, J = 7.0 Hz, 3H), 0.98 (t, J = 7.9 Hz, 9H), 0.64 (q, J = 7.9 Hz, 6H).

^{13}C-NMR (126 MHz, $CDCl_3$) δ = 97.1, 61.9, 36.0, 33.9, 27.7, 15.2, 6.8, 5.1.

HRMS (ESI-/Q-TOF): Calculated for $[M+Na]^+$ = $[C_{12}H_{27}BrNaO_2Si]^+$: m/z 333.08559; Found: m/z 333.08503

TLC: Rf: 0.46 using hexanes : ethyl acetate 20:1 (V/V) as an eluent; Visualization: $KMnO_4$

Compound 21: ((5-bromo-1-ethoxypentyl)oxy)triethylsilane

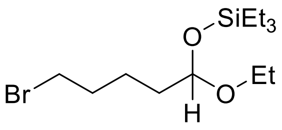
21

Compound **21** was prepared according to General procedure D, starting from ethyl 5-bromopentanoate on a 5.00 mmol scale, using 0.05 mol% **1d** as a catalyst, yielding 1.61 g (4.95 mmol) product, which corresponds to a yield of 99%. (See NMR spectra)

Physical state: clear liquid

^{1}H-NMR (500 MHz, $CDCl_3$) δ = 4.76 (dd, J = 6.1, 3.8 Hz, 1H), 3.69 (dq, J = 9.2, 7.0 Hz, 1H), 3.45 – 3.34 (m, 3H), 1.91 – 1.82 (m, 2H), 1.67 – 1.45 (m, 4H), 1.18 (t, J = 7.0 Hz, 3H), 0.97 (t, J = 8.0 Hz, 9H), 0.63 (q, J = 7.9 Hz, 6H).

^{13}C-NMR (126 MHz, $CDCl_3$) δ = 97.7, 61.8, 36.7, 33.6, 32.7, 23.1, 15.2, 6.8, 5.1.

HRMS (ESI-/Q-TOF): Calculated for $[M+Na]^+$ = $[C_{13}H_{29}BrNaO_2Si]^+$: m/z 347.10124; Found: m/z 347.10155

TLC: Rf: 0.45 using hexanes : ethyl acetate 20:1 (V/V) as an eluent; Visualization: $KMnO_4$

Compound 22: ((6-bromo-1-ethoxyhexyl)oxy)triethylsilane

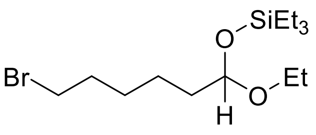

22

Compound **22** was prepared according to General procedure D, starting from ethyl 6-bromohexanoate on a 5.00 mmol scale, using 0.05 mol% **1d** as a catalyst, yielding 1.63 g (4.80 mmol) product, which corresponds to a yield of 96%. (See NMR spectra)

Physical state: clear oil

^{1}H-NMR (500 MHz, $CDCl_3$) δ = 4.76 (dd, J = 6.1, 4.2 Hz, 1H), 3.69 (dq, J = 9.3, 7.1 Hz, 1H), 3.44 – 3.36 (m, 3H), 1.86 (p, J = 6.9 Hz, 2H), 1.67 – 1.50 (m, 2H), 1.49 – 1.34 (m, 4H), 1.19 (t, J = 7.1 Hz, 3H), 0.97 (t, J = 8.0 Hz, 9H), 0.63 (q, J = 8.0 Hz, 6H).

^{13}C-NMR (126 MHz, $CDCl_3$) δ = 97.8, 61.8, 37.4, 33.8, 32.8, 28.0, 23.6, 15.2, 6.8, 5.1.

HRMS (ESI-/Q-TOF): Calculated for $[M+Na]^+$ = $[C_{14}H_{31}BrNaO_2Si]^+$: m/z 361.11689; Found: m/z 361.11642

TLC: Rf: 0.47 using hexanes : ethyl acetate 20:1 (V/V) as an eluent; Visualization: $KMnO_4$

Compound 23: (4-bromo-1-isopropoxybutoxy)triethylsilane

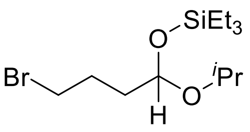

23

Compound **23** was prepared according to General procedure D, starting from isopropyl 4-bromobutanoate on a 2.50 mmol scale, using 0.05 mol% **1d** as a catalyst, yielding 775 mg (2.38 mmol) product, which corresponds to a yield of 95%. (See NMR spectra)

Physical state: clear oil

^{1}H-NMR (500 MHz, $CDCl_3$) δ = 4.86 (t, J = 5.1 Hz, 1H), 3.86 – 3.79 (m, 1H), 3.49 – 3.36 (m, 2H), 2.06 – 1.81 (m, 2H), 1.69 (td, J = 7.6, 5.0 Hz, 2H), 1.18 (d, J = 6.2 Hz, 3H), 1.10 (d, J = 6.1 Hz, 3H), 0.97 (t, J = 7.9 Hz, 9H), 0.63 (q, J = 8.2 Hz, 6H).

^{13}C-NMR (126 MHz, $CDCl_3$) δ = 95.1, 68.0, 36.7, 34.0, 27.8, 23.5, 21.9, 6.8, 5.1.

HRMS (ESI-/Q-TOF): Calculated for $[M+Na]^+$ = $[C_{13}H_{29}BrNaO_2Si]^+$: m/z 347.10124; Found: m/z 347.10120

TLC: Rf: 0.52 using hexanes : ethyl acetate 20:1 (V/V) as an eluent; Visualization: $KMnO_4$

Compound 24: (2-bromo-1-ethoxyethoxy)triethylsilane

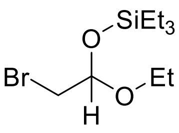

24

Compound **24** was prepared according to General procedure D, starting from ethyl 2-bromoacetate on a 5.00 mmol scale, using 0.05 mol% **1d** as a catalyst, yielding 1.35 g (4.78 mmol) product, which corresponds to a yield of 96%. (See NMR spectra)

Physical state: clear liquid

^{1}H-NMR (500 MHz, $CDCl_3$) δ = 4.93 (dd, J = 6.0, 4.3 Hz, 1H), 3.71 (dq, J = 9.2, 7.0 Hz, 1H), 3.52 (dq, J = 9.2, 7.0 Hz, 1H), 3.36 (dd, J = 10.4, 6.0 Hz, 1H), 3.27 (dd, J = 10.4, 4.2 Hz, 1H), 1.23 (t, J = 7.1 Hz, 3H), 0.99 (t, J = 8.0 Hz, 9H), 0.67 (q, J = 7.7 Hz, 6H).

^{13}C-NMR (126 MHz, $CDCl_3$) δ = 96.5, 62.3, 34.9, 15.1, 6.7, 5.0.

HRMS (ESI-/Q-TOF): Calculated for $[M+Na]^+$ = $[C_{10}H_{23}BrNaO_2Si]^+$: m/z 305.05429; Found: m/z 305.05410

TLC: Rf: 0.69 using hexanes : ethyl acetate 20:1 (V/V) as an eluent; Visualization: $KMnO_4$

Compound 25: (3-chloro-1-ethoxypropoxy)triethylsilane

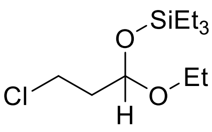

25

Compound **25** was prepared according to General procedure D, starting from ethyl 3-chloropropanoate on a 5.00 mmol scale, using 0.05 mol% **1d** as a catalyst, yielding 1.15 g (4.55 mmol) product, which corresponds to a yield of 91%. (See NMR spectra)

Physical state: clear liquid

^{1}H-NMR (500 MHz, $CDCl_3$) δ = 4.97 (dd, J = 6.1, 4.3 Hz, 1H), 3.71 (dq, J = 9.2, 7.1 Hz, 1H), 3.66 – 3.56 (m, 2H), 3.44 (dq, J = 9.2, 7.1 Hz, 1H), 2.11 – 1.95 (m, 2H), 1.20 (t, J = 7.1 Hz, 3H), 0.98 (t, J = 7.9 Hz, 9H), 0.65 (q, J = 7.9 Hz, 6H).

^{13}C-NMR (126 MHz, $CDCl_3$) δ = 95.3, 62.3, 40.8, 40.5, 15.2, 6.7, 5.0.

HRMS (ESI-/Q-TOF): Calculated for $[M+Na]^+$ = $[C_{11}H_{25}ClNaO_2Si]^+$: m/z 275.12045; Found: m/z 275.12001

TLC: Rf: 0.53 using hexanes : ethyl acetate 20:1 (V/V) as an eluent; Visualization: $KMnO_4$

Compound 26: (2-bromo-1-methoxypropoxy)triethylsilane

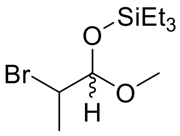

26

Compound **26** was prepared according to General procedure D, starting from methyl 2-bromopropanoate on a 1.50 mmol scale, using 0.05 mol% **1d** as a catalyst, yielding 401 mg (1.42 mmol) product, which corresponds to a yield of 95%. (dr: 71:29)(See NMR spectra)

Physical state: clear liquid

Major diastereomer:

^{1}H-NMR (500 MHz, $CDCl_3$) δ = 4.71 (d, J = 4.6 Hz, 1H), 4.02 (qd, J = 6.8, 4.6 Hz, 1H), 3.38 (s, 3H), 1.64 (d, J = 6.8 Hz, 3H), 0.99 (t, J = 7.9 Hz, 9H), 0.69 (q, J = 8.2 Hz, 6H).

^{13}C-NMR (126 MHz, $CDCl_3$) δ = 100.3, 54.1, 50.5, 20.1, 6.7, 5.0.

Minor diastereomer:

^{1}H-NMR (500 MHz, $CDCl_3$) δ = 4.76 (d, J = 3.8 Hz, 1H), 3.93 (qd, J = 6.9, 3.7 Hz, 1H), 3.40 (s, 3H), 1.65 (d, J = 6.8 Hz, 3H), 0.99 (t, J = 7.9 Hz, 9H), 0.68 (q, J = 8.1 Hz, 6H).

^{13}C-NMR (126 MHz, $CDCl_3$) δ = 99.9, 55.1, 50.9, 18.5, 6.7, 4.9.

HRMS (ESI-/Q-TOF): Calculated for $[M+Na]^+$ = $[C_{10}H_{23}BrNaO_2Si]^+$: m/z 305.05429; Found: m/z 305.05412

TLC: Rf: 0.60 using hexanes : ethyl acetate 20:1 (V/V) as an eluent; Visualization: $KMnO_4$

Compound 27: ((2-bromo-1-ethoxyoctyl)oxy)triethylsilane

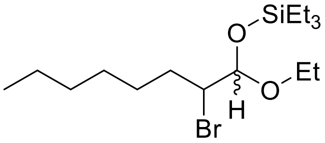

27

Compound **27** was prepared according to General procedure D, starting from ethyl 2-bromooctanoate on a 27.1 mmol scale, using 0.1 mol% **1d** as a catalyst, yielding 9.92 g (27.0 mmol) product, which corresponds to a yield of 99+%. (dr: 76:24)(See NMR spectra)

Physical state: clear oil

Major diastereomer:

^{1}H-NMR (500 MHz, $CDCl_3$) δ = 4.82 (d, J = 4.5 Hz, 1H), 3.89 (ddd, J = 10.2, 4.5, 3.2 Hz, 1H), 3.75 – 3.66 (m, 1H), 3.56 – 3.48 (m, 1H), 1.99 – 1.91 (m, 1H), 1.79 – 1.70 (m, 1H), 1.64 – 1.53 (m, 1H), 1.42 – 1.26 (m, 7H), 1.25 – 1.18 (m, 3H), 1.03 – 0.97 (m, 9H), 0.91 – 0.85 (m, 3H), 0.72 – 0.64 (m, 6H).

^{13}C-NMR (126 MHz, $CDCl_3$) δ = 98.9, 62.6, 58.6, 32.6, 31.6, 28.7, 27.4, 22.6, 15.1, 14.0, 6.8, 5.1.

Minor diastereomer:

^{1}H-NMR (500 MHz, $CDCl_3$) δ = 4.88 (d, J = 3.9 Hz, 1H), 3.82 (ddd, J = 10.5, 3.9, 2.9 Hz, 1H), 3.75 – 3.66 (m, 1H), 3.58 – 3.47 (m, 1H), 2.06 – 1.97 (m, 1H), 1.82 – 1.74 (m, 1H), 1.59 – 1.53 (m, 1H), 1.42 – 1.25 (m, 7H), 1.25 – 1.21 (m, 3H), 1.03 – 0.97 (m, 9H), 0.91 – 0.85 (m, 3H), 0.71 – 0.63 (m, 6H).

^{13}C-NMR (126 MHz, $CDCl_3$) δ = 98.8, 63.4, 58.9, 31.6, 31.2, 28.6, 27.6, 22.6, 15.1, 14.0, 6.7, 5.1.

HRMS (ESI-/Q-TOF): Calculated for $[M+Na]^+$ = $[C_{16}H_{35}BrNaO_2Si]^+$: m/z 389.14819; Found: m/z 389.14949

TLC: Rf: 0.39 using hexanes : ethyl acetate 100:1 (V/V) as an eluent; Visualization: $KMnO_4$

Compound 28: (4-bromo-1-ethoxy-2-methylbutoxy)triethylsilane

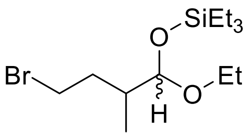

28

Compound **28** was prepared according to General procedure D, starting from ethyl 4-bromo-2-methylbutanoate on a 5.00 mmol scale, using 0.5 mol% **1f** as a catalyst, yielding 1.48 g (4.55 mmol) product, which corresponds to a yield of 91%. (dr: 59:41)(See NMR spectra)

Physical state: clear liquid

Major diastereomer:

^{1}H-NMR (500 MHz, $CDCl_3$) δ = 4.61 (d, J = 3.7 Hz, 1H), 3.72 – 3.62 (m, 1H), 3.59 – 3.49 (m, 1H), 3.47 – 3.34 (m, 2H), 2.06 (dtd, J = 14.0, 7.7, 5.1 Hz, 1H), 1.89 – 1.80 (m, 1H), 1.77 – 1.67 (m, 1H), 1.18 (t, J = 7.0 Hz, 3H), 0.98 (t, J = 8.0 Hz, 9H), 0.93 (d, J = 6.9 Hz, 3H), 0.65 (q, J = 7.9 Hz, 6H).

^{13}C-NMR (126 MHz, $CDCl_3$) δ = 100.9, 62.5, 38.2, 35.1, 32.7, 15.33, 13.3, 6.98, 5.24.

Minor diastereomer:

^{1}H-NMR (500 MHz, $CDCl_3$) δ = 4.59 (d, J = 4.1 Hz, 1H), 3.72 – 3.62 (m, 1H), 3.59 – 3.49 (m, 1H), 3.47 – 3.34 (m, 2H), 2.15 (dtd, J = 14.2, 8.1, 4.6 Hz, 1H), 1.89 – 1.80 (m, 1H), 1.77 – 1.67 (m, 1H), 1.18 (t, J = 7.0 Hz, 3H), 0.98 (t, J = 8.0 Hz, 9H), 0.91 (d, J = 6.9 Hz, 3H), 0.65 (q, J = 7.9 Hz, 6H).

^{13}C-NMR (126 MHz, $CDCl_3$) δ = 100.3, 62.6, 38.4, 34.0, 32.9, 15.31, 14.6, 7.00, 5.28.

HRMS (ESI-/Q-TOF): Calculated for $[M+Na]^+$ = $[C_{13}H_{29}BrNaO_2Si]^+$: m/z 347.10124; Found: m/z 347.10234

TLC: Rf: 0.57 using hexanes : ethyl acetate 20:1 (V/V) as an eluent; Visualization: $KMnO_4$

Compound 29: (4-bromo-1-ethoxy-2-fluorobutoxy)triethylsilane

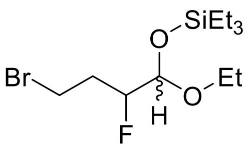

29

Compound **29** was prepared according to General procedure D, starting from ethyl 4-bromo-2-fluorobutanoate on a 2.50 mmol scale, using 1.0 mol% **1f** as a catalyst, yielding 740 mg (2.25 mmol) product, which corresponds to a yield of 90%. (See NMR spectra)

Physical state: clear liquid

^{1}H-NMR (500 MHz, $CDCl_3$) δ = 4.88 (dd, J = 6.9, 3.7 Hz, 1H), 4.54 (ddt, J = 48.2, 9.3, 3.5 Hz, 1H), 3.74 (dq, J = 9.2, 7.1 Hz, 1H), 3.60 – 3.45 (m, 3H), 2.31 – 2.11 (m, 2H), 1.21 (t, J = 7.0 Hz, 3H), 0.97 (t, J = 7.9 Hz, 9H), 0.66 (q, J = 7.7 Hz, 6H).

^{13}C-NMR (126 MHz, $CDCl_3$) δ = 96.2 (d, J = 27.8 Hz), 91.4 (d, J = 174.3 Hz), 63.0, 32.1 (d, J = 20.2 Hz), 28.9 (d, J = 4.4 Hz), 15.2, 6.7, 5.0.

^{19}F-NMR (282 MHz, $CDCl_3$) δ = -194.80 (dddd, J = 48.9, 31.4, 17.4, 6.9 Hz).

HRMS (ESI-/Q-TOF): Calculated for $[M+Na]^+$ = $[C_{12}H_{26}BrFNaO_2Si]^+$: m/z 351.07617; Found: m/z 351.07656

TLC: Rf: 0.58 using hexanes : ethyl acetate 20:1 (V/V) as an eluent; Visualization: $KMnO_4$

Compound 30: 4-bromo-1-ethoxy-2-methoxybutoxy)triethylsilane

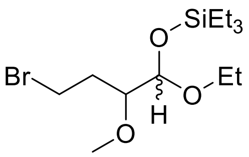

30

Compound **30** was prepared according to General procedure D, starting from ethyl 4-bromo-2-methoxybutanoate (**S24**) on a 1.00 mmol scale, using 1.0 mol% **1f** as a catalyst, yielding 338 mg (990 µmol) product, which corresponds to a yield of 99%. (dr: 61:39)(See NMR spectra)

Physical state: clear liquid

Major diastereomer:

^{1}H-NMR (500 MHz, $CDCl_3$) δ = 4.74 (d, J = 4.0 Hz, 1H), 3.78 – 3.68 (m, 1H), 3.59 – 3.51 (m, 2H), 3.48 (s, 3H), 3.51 – 3.46 (m, 1H), 3.33 – 3.25 (m, 1H), 2.21 – 1.95 (m, 2H), 1.24 – 1.18 (m, 3H), 1.02 – 0.95 (m, 9H), 0.73 – 0.63 (m, 6H).

^{13}C-NMR (126 MHz, $CDCl_3$) δ = 98.1, 81.3, 63.2, 59.2, 33.0, 30.8, 15.3, 6.79, 5.2.

Minor diastereomer:

^{1}H-NMR (500 MHz, $CDCl_3$) δ = 4.81 (d, J = 4.1 Hz, 1H), 3.78 – 3.68 (m, 1H), 3.59 – 3.51 (m, 2H), 3.47 (s, 3H), 3.51 – 3.46 (m, 1H), 3.33 – 3.25 (m, 1H), 2.21 – 1.95 (m, 2H), 1.24 – 1.18 (m, 3H), 1.02 – 0.95 (m, 9H), 0.73 – 0.63 (m, 6H).

^{13}C-NMR (126 MHz, $CDCl_3$) δ = 97.0, 81.6, 62.9, 58.8, 32.2, 30.9, 15.2, 6.78, 5.0.

HRMS (ESI-/Q-TOF): Calculated for $[M+Na]^+$ = $[C_{13}H_{29}BrNaO_3Si]^+$: m/z 363.09615; Found: m/z 363.09575

TLC: Rf: 0.55 and 0.62 using hexanes : ethyl acetate 10:1 (V/V) as an eluent; Visualization: $KMnO_4$

Compound 31: (4-bromo-1-ethoxy-2,2-difluorobutoxy)triethylsilane

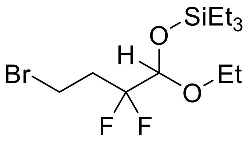

31

Compound **31** was prepared according to General procedure D, starting from ethyl 4-bromo-2,2-difluorobutanoate on a 2.50 mmol scale, using 2.0 mol% **1d** as a catalyst, yielding 448 mg (1.29 mmol) product, which corresponds to a yield of 52%. (See NMR spectra)

Physical state: clear liquid

^{1}H-NMR (500 MHz, $CDCl_3$) δ = 4.72 (dd, J = 4.6, 3.7 Hz, 1H), 3.75 (dq, J = 9.1, 7.1 Hz, 1H), 3.57 – 3.47 (m, 3H), 2.61 – 2.49 (m, 2H), 1.22 (t, J = 7.1 Hz, 3H), 0.98 (t, J = 8.0 Hz, 9H), 0.66 (q, J = 8.3 Hz, 6H).

^{13}C-NMR (126 MHz, $CDCl_3$) δ = 120.7 (t, J = 246.6 Hz), 95.8 (t, J = 35.1 Hz), 64.5, 34.5 (t, J = 23.3 Hz), 23.7 (t, J = 6.0 Hz), 15.1, 6.6, 4.8.

^{19}F-NMR (282 MHz, $CDCl_3$) δ = -109.4 – -110.7 (m), -110.8 – -112.0 (m).

HRMS (ESI-/Q-TOF): Calculated for $[M+Na]^+$ = $[C_{12}H_{25}BrF_2NaO_2Si]^+$: m/z 369.06675; Found: m/z 369.06704

TLC: Rf: 0.17 using hexanes as an eluent; Visualization: $KMnO_4$

Compound 32: (1-ethoxy-2,2,2-trifluoroethoxy)triethylsilane

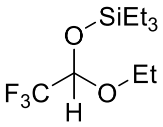

32

Compound **32** was prepared according to General procedure D, starting from ethyl 2,2,2-trifluoroacetate on a 5.00 mmol scale, using 0.25 mol% **1e** as a catalyst, yielding 1.10 g (4.26 mmol) product, which corresponds to a yield of 85%. (See NMR spectra)

Physical state: clear liquid

^{1}H-NMR (500 MHz, $CDCl_3$) δ = 4.91 (qd, J = 3.8, 0.8 Hz, 1H), 3.78 (dq, J = 9.4, 7.1 Hz, 1H), 3.66 (dq, J = 9.3, 7.0 Hz, 1H), 1.26 (td, J = 7.1, 0.7 Hz, 3H), 0.99 (t, J = 8.0 Hz, 9H), 0.69 (q, J = 8.1 Hz, 6H).

^{13}C-NMR (126 MHz, $CDCl_3$) δ = 121.9 (q, J = 284.7 Hz), 92.5 (q, J = 35.7 Hz), 63.8, 15.0, 6.4, 4.8.

^{19}F-NMR (282 MHz, $CDCl_3$) δ = -82.9 (d, J = 3.7 Hz).

HRMS (ESI-/Q-TOF): Calculated for $[M+Na]^+$ = $[C_{10}H_{21}F_3NaO_2Si]^+$: m/z 281.11551; Found: m/z 281.11537

TLC: Rf: 0.27 using hexanes as an eluent; Visualization: $KMnO_4$

Compound 33: (2-(2-chloroethoxy)-1-ethoxyethoxy)triethylsilane

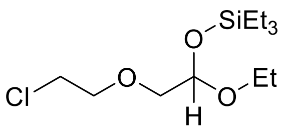

33

Compound **33** was prepared according to General procedure D, starting from ethyl 2-(2-chloroethoxy)acetate on a 10.0 mmol scale, using 0.1 mol% **1f** as a catalyst, yielding 2.70 g (9.54 mmol) product, which corresponds to a yield of 95%. (See NMR spectra)

Physical state: pale yellow liquid

^{1}H-NMR (500 MHz, $CDCl_3$) δ = 4.89 (dd, J = 5.5, 4.5 Hz, 1H), 3.83 – 3.70 (m, 3H), 3.61 (t, J = 6.0 Hz, 2H), 3.53 – 3.43 (m, 3H), 1.22 (t, J = 7.0 Hz, 3H), 0.97 (t, J = 8.0 Hz, 9H), 0.68 – 0.62 (m, 6H).

^{13}C-NMR (126 MHz, $CDCl_3$) δ = 96.2, 74.5, 71.6, 62.5, 42.6, 15.2, 6.7, 5.0.

HRMS (ESI-/Q-TOF): Calculated for $[M+Na]^+$ = $[C_{12}H_{27}ClNaO_3Si]^+$: m/z 305.13102; Found: m/z 305.13249

TLC: Rf: 0.55 using hexanes : ethyl acetate 10:1 (V/V) as an eluent; Visualization: $KMnO_4$

Compound 34: (2-(2-bromoethoxy)-1-ethoxyethoxy)triethylsilane

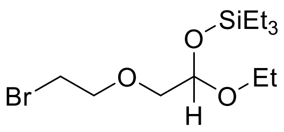

34

Compound **34** was prepared according to General procedure D, starting from ethyl 2-(2-bromoethoxy)acetate on a 10.0 mmol scale, using 0.5 mol% **1f** as a catalyst, yielding 3.21 g (9.81 mmol) product, which corresponds to a yield of 98%. (See NMR spectra)

Physical state: clear liquid

^{1}H-NMR (500 MHz, $CDCl_3$) δ = 4.89 (dd, J = 5.6, 4.4 Hz, 1H), 3.89 – 3.71 (m, 4H), 3.53 – 3.42 (m, 4H), 1.22 (t, J = 7.1 Hz, 3H), 0.98 (t, J = 7.9 Hz, 9H), 0.65 (q, J = 7.8 Hz, 6H).

^{13}C-NMR (126 MHz, $CDCl_3$) δ = 96.2, 74.4, 71.5, 62.5, 30.2, 15.2, 6.7, 5.1.

HRMS (ESI-/Q-TOF): Calculated for $[M+Na]^+$ = $[C_{12}H_{27}BrNaO_3Si]^+$: m/z 349.08050 Found: m/z 349.08008

TLC: Rf: 0.52 using hexanes : ethyl acetate 10:1 (V/V) as an eluent; Visualization: $KMnO_4$

Compound 35: 5-ethoxy-3,3,12,12-tetraethyl-4,11-dioxa-3,12-disilatetradecane

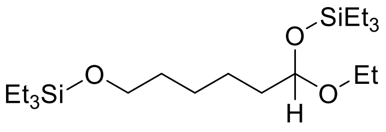

35

Compound **35** was prepared according to General procedure D, starting from ethyl 6-hydroxyhexanoate on a 5.00 mmol scale, using 0.25 mol% **1f** as a catalyst and 2.1 equiv. TESH (**3**), yielding 1.85 g (4.73 mmol) product, which corresponds to a yield of 95%. (See NMR spectra)

Physical state: clear oil

^{1}H-NMR (500 MHz, $CDCl_3$) δ = 4.75 (dd, J = 6.2, 4.3 Hz, 1H), 3.69 (dq, J = 9.3, 7.1 Hz, 1H), 3.59 (t, J = 6.7 Hz, 2H), 3.40 (dq, J = 9.3, 7.1 Hz, 1H), 1.66 – 1.58 (m, 1H), 1.57 – 1.48 (m, 3H), 1.42 – 1.29 (m, 4H), 1.18 (t, J = 7.0 Hz, 3H), 1.00 – 0.92 (m, 18H), 0.69 – 0.55 (m, 12H).

^{13}C-NMR (126 MHz, $CDCl_3$) δ = 98.1, 62.9, 61.7, 37.7, 32.9, 25.8, 24.3, 15.2, 6.8, 6.7, 5.1, 4.5.

HRMS (ESI-/Q-TOF): Calculated for $[M+Na]^+$ = $[C_{20}H_{46}NaO_3Si_2]^+$: m/z 413.28777 Found: m/z 413.28847

TLC: Rf: 0.48 using hexanes : ethyl acetate 20:1 (V/V) as an eluent; Visualization: $KMnO_4$

Compound 36: triethyl((tetrahydrofuran-2-yl)oxy)silane

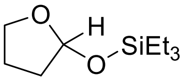

36

Compound **36** was prepared according to General procedure D, starting from gamma-butyrolactone on a 5.00 mmol scale, using 0.1 mol% **1h** as a catalyst, yielding 912 mg (4.51 mmol) product, which corresponds to a yield of 90%. (See NMR spectra)

Physical state: clear liquid

^{1}H-NMR (500 MHz, $CDCl_3$) δ = 5.49 (t, J = 2.7 Hz, 1H), 3.98 (td, J = 8.2, 4.8 Hz, 1H), 3.81 – 3.74 (m, 1H), 2.07 – 1.98 (m, 1H), 1.87 – 1.76 (m, 3H), 0.95 (t, J = 7.9 Hz, 9H), 0.62 (q, J = 7.9 Hz, 6H).

^{13}C-NMR (126 MHz, $CDCl_3$) δ = 98.6, 67.2, 35.0, 23.3, 6.7, 4.8.

HRMS (ESI-/Q-TOF): Calculated for $[M+H]^+$ = $[C_{10}H_{23}O_2Si]^+$: m/z 203.14618 Found: m/z 203.14627

TLC: Rf: 0.71 using hexanes : ethyl acetate 20:1 (V/V) as an eluent; Visualization: $KMnO_4$

Compound 37: triethyl((tetrahydro-2H-pyran-2-yl)oxy)silane

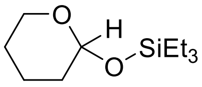

37

Compound **37** was prepared according to General procedure D, starting from tetrahydro-2H-pyran-2-one on a 5.00 mmol scale, using 0.1 mol% **1h** as a catalyst, yielding 1.03 g (4.76 mmol) product, which corresponds to a yield of 95%. (See NMR spectra)

Physical state: clear liquid

^{1}H-NMR (500 MHz, $CDCl_3$) δ = 4.93 – 4.84 (m, 1H), 3.95 (ddd, J = 11.5, 5.6, 3.7 Hz, 1H), 3.45 (dt, J = 11.2, 5.0 Hz, 1H), 1.88 – 1.80 (m, 1H), 1.71 – 1.65 (m, 1H), 1.52 – 1.45 (m, 4H), 0.95 (t, J = 8.0 Hz, 9H), 0.62 (q, J = 8.0 Hz, 6H).

^{13}C-NMR (126 MHz, $CDCl_3$) δ = 94.2, 63.1, 33.5, 25.4, 20.0, 6.6, 4.8.

HRMS (ESI-/Q-TOF): Calculated for $[M+Na]^+$ = $[C_{11}H_{24}NaO_2Si]^+$: m/z 239.14378 Found: m/z 239.14315

TLC: Rf: 0.53 using hexanes : ethyl acetate 20:1 (V/V) as an eluent; Visualization: $KMnO_4$

Compound 38: triethyl((tetrahydro-2H-pyran-2-yl)oxy)silane

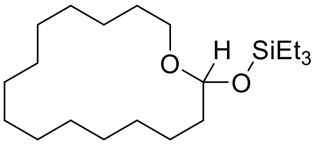

38

Compound **38** was prepared according to General procedure D, starting from oxacyclohexadecan-2-one on a 5.00 mmol scale, yielding 1.76 g (4.93 mmol) product, which corresponds to a yield of 99%. (See NMR spectra)

Physical state: clear oil

^{1}H-NMR (500 MHz, $CDCl_3$) δ = 4.73 (dd, J = 8.1, 2.4 Hz, 1H), 3.82 – 3.76 (m, 1H), 3.24 (td, J = 8.9, 3.3 Hz, 1H), 1.69 – 1.60 (m, 1H), 1.57 (tq, J = 9.2, 4.7 Hz, 1H), 1.53 – 1.23 (m, 24H), 0.97 (t, J = 8.0 Hz, 9H), 0.63 (q, J = 8.0 Hz, 6H).

^{13}C-NMR (126 MHz, $CDCl_3$) δ = 98.5, 66.9, 37.9, 29.2, 27.5, 27.3 (3C), 26.7, 26.1 (2C), 26.0, 25.9, 25.2, 23.5, 6.8, 5.1.

HRMS (ESI-/Q-TOF): Calculated for $[M+H]^+$ = $[C_{21}H_{45}O_2Si]^+$: m/z 357.31833 Found: m/z 357.31779

TLC: Rf: 0.35 using hexanes : ethyl acetate 100:1 (V/V) as an eluent; Visualization: $KMnO_4$

Compound 39: triethyl((oxacycloheptadec-8-en-2-yl)oxy)silane

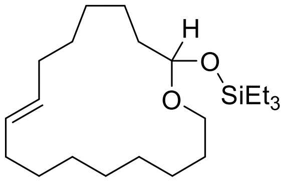

39

Compound **39** was prepared according to General procedure D, starting from oxacycloheptadec-8-en-2-one on a 5.00 mmol scale, using 0.25 mol% **1f** as a catalyst, yielding 1.83 g (4.96 mmol) product, which corresponds to a yield of 99%. (See NMR spectra)

Physical state: clear oil

^{1}H-NMR (500 MHz, $CDCl_3$) δ = 5.37 (dt, J = 15.1, 6.5 Hz, 1H), 5.30 (dt, J = 15.2, 6.4 Hz, 1H), 4.73 (dd, J = 8.4, 2.3 Hz, 1H), 3.65 – 3.59 (m, 1H), 3.42 – 3.35 (m, 1H), 2.10 – 1.93 (m, 4H), 1.72 – 1.63 (m, 1H), 1.63 – 1.53 (m, 1H), 1.53 – 1.19 (m, 18H), 0.96 (t, J = 8.0 Hz, 9H), 0.62 (q, J = 7.9 Hz, 6H).

^{13}C-NMR (126 MHz, $CDCl_3$) δ = 131.2, 130.6, 98.4, 65.7, 37.7, 32.0, 31.8, 29.6 (2C), 28.7, 28.5, 28.3, 27.8, 26.7, 26.3, 23.6, 6.8, 5.1.

HRMS (ESI-/Q-TOF): Calculated for $[M+H]^+$ = $[C_{22}H_{45}O_2Si]^+$: m/z 369.31833 Found: m/z 369.31815

TLC: Rf: 0.31 using hexanes : ethyl acetate 100:1 (V/V) as an eluent; Visualization: $KMnO_4$

Compound 40: triethyl(((Z)-1-(((Z)-icos-11-en-1-yl)oxy)icos-11-en-1-yl)oxy)silane

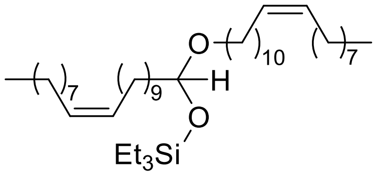

40

Compound **40** was prepared according to General procedure D, starting from natural Jojoba oil on a 5.00 mmol scale, yielding 3.45 g (4.89 mmol) product, which corresponds to a yield of 98%. Jojoba oil is comprised almost entirely of wax esters; its main fatty-acid component is 11-eicosenoic acid (approx. 77%), while its main fatty alcohol components are 11-eicosenol (approx. 44%) and 12-docosenol (approx. 45%). Thus, 11-eicosenyl-11-eicosenoate (the ester leading to compound **40**) is just one of the relevant components of Jojoba oil besides a plethora of mono-esters. Even so, when we compared the NMR spectra of pure Jojoba oil and the NMR spectra of the reduced oil, we could see that complete conversion of practically all of the ester components of Jojoba oil was achieved, as showed by the disappearance of the triplet at 4.05 ppm (O-CH_2- peak) and the appearance of the acetalic peak at 4.73 ppm. (See NMR spectra)

Physical state: clear oil

^{1}H-NMR (500 MHz, $CDCl_3$) δ = 5.35 (t, J = 4.8 Hz, 4H), 4.73 (dd, J = 6.3, 4.3 Hz, 1H), 3.66 – 3.58 (m, 1H), 3.35 – 3.26 (m, 1H), 2.05 – 1.98 (m, 8H), 1.65 – 1.47 (m, 2H), 1.39 – 1.22 (m, 54H), 0.98 (t, J = 7.9 Hz, 9H), 0.88 (t, J = 6.8 Hz, 6H), 0.64 (q, J = 7.9 Hz, 6H).

^{13}C-NMR (126 MHz, $CDCl_3$) δ = 129.90, 129.88, 98.35, 66.51, 37.68, 31.91, 29.80, 29.79, 29.78, 29.70, 29.69, 29.68, 29.67, 29.64, 29.62, 29.61, 29.60, 29.58, 29.57, 29.55, 29.53, 29.52, 29.49, 29.33, 29.32, 27.22, 26.26, 24.55, 22.68, 14.09, 6.82, 5.17.

HRMS (ESI-/Q-TOF): Calculated for $[M+Na]^+$ = $[C_{46}H_{92}NaO_2Si]^+$: m/z 727.67588 Found: m/z 727.67410

TLC: Rf: 0.27 using hexanes : ethyl acetate 100:1 (V/V) as an eluent; Visualization: $KMnO_4$

Compound 41: 3,3,13,13-tetraethyl-5,11-di((8Z,10E,12E)-heptadeca-8,10,12-trien-1-yl)-8-(((9Z,11E,13E)-1-((triethylsilyl)oxy)octadeca-9,11,13-trien-1-yl)oxy)-4,6,10,12-tetraoxa-3,13-disilapentadecane

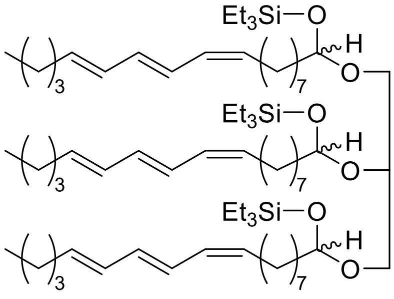

41

Compound **41** was prepared according to General procedure D, starting from natural Tung oil on a 5.00 mmol scale, using 0.75 mol% **1f** as a catalyst and 3.3 equiv. TESH (**3**), yielding 5.60 g (4.58 mmol) product, which corresponds to a yield of 92%. The main fatty-acid component of Tung oil is α-eleostearic acid (approx. 82%), but several other fatty-acids are also present. Thus, the triglyceride leading to compound **41** is just one of the relevant components of Tung oil besides several other lipids. Even so, when we compared the NMR spectra of pure Tung oil and the NMR spectra of the reduced oil, we could see that complete conversion of practically all of the ester components of Tung oil was achieved, as showed by the disappearance of the peaks at 4.29 and 4.14 ppm (O-CH_2-peaks), and the appearance of the acetalic peaks between 5.05 – 4.70 ppm. (See NMR spectra)

Physical state: pale yellow oil

^{1}H-NMR (500 MHz, $CDCl_3$) δ = 6.38 (dd, J = 14.2, 11.2 Hz), 6.21 – 6.06 (m), 5.99 (t, J = 11.0 Hz), 5.70 (dt, J = 14.2, 7.1 Hz), 5.43 – 5.32 (m), 4.98 (dq, J = 10.2, 4.3 Hz), 4.76 (m), 3.88 – 3.75 (m), 3.75 – 3.58 (m), 3.52 – 3.26 (m), 2.17 (q, J = 7.5 Hz), 2.10 (q, J = 7.2 Hz), 2.07 – 1.97 (m), 1.65 – 1.45 (m), 1.41 – 1.25 (m), 1.01 – 0.94 (m), 0.93 – 0.87 (m), 0.70 – 0.59 (m).

^{13}C-NMR (126 MHz, $CDCl_3$) δ = 135.07, 135.05, 135.04, 132.77, 131.91, 131.88, 131.85, 130.59, 128.65, 125.99, 98.86, 98.79, 98.77, 98.76, 98.72, 98.66, 98.54, 98.52, 98.50, 98.36, 98.34, 98.32, 98.16, 98.07, 97.94, 74.59, 74.37, 74.19, 74.15, 68.53, 68.23, 67.09, 67.07, 67.01, 66.93, 66.84, 66.61, 38.22, 37.82, 37.68, 37.59, 37.52, 32.48, 31.92, 31.90, 31.47, 29.78, 29.76, 29.71, 29.63, 29.60, 29.56, 29.52, 29.34, 29.32, 27.89, 27.87, 27.27, 27.19, 25.62, 24.47, 24.42, 24.40, 22.67, 22.56, 22.21, 13.88, 6.86, 6.83, 6.78, 6.76, 6.43, 5.32, 5.24, 5.18, 5.15, 5.11.

HRMS (ESI-/Q-TOF): Calculated for $[M+Na]^+$ = $[C_{75}H_{140}NaO_6Si_3]^+$: m/z 1243.98499 Found: m/z 1243.98335

TLC: Rf: 0.69 using hexanes : ethyl acetate 20:1 (V/V) as an eluent; Visualization: $KMnO_4$

Compound 42: (1-(but-3-yn-1-yloxy)-3-phenylpropoxy)triethylsilane

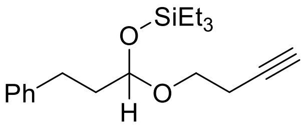

42

Compound **42** was prepared according to General procedure D, starting from but-3-yn-1-yl 3-phenylpropanoate on a 5.00 mmol scale, using 0.5 mol% **1f** as a catalyst, yielding 1.55 g (4.87 mmol) product, which corresponds to a yield of 97%. (See NMR spectra)

Physical state: clear liquid

^{1}H-NMR (500 MHz, $CDCl_3$) δ = 7.31 – 7.26 (m, 2H), 7.22 – 7.16 (m, 3H), 4.83 (dd, J = 6.4, 4.1 Hz, 1H), 3.77 (dt, J = 9.2, 6.9 Hz, 1H), 3.53 (dt, J = 9.2, 7.2 Hz, 1H), 2.80 – 2.65 (m, 2H), 2.51 – 2.46 (m, 2H), 2.02 – 1.93 (m, 2H), 1.92 – 1.84 (m, 1H), 0.97 (t, J = 7.9 Hz, 9H), 0.64 (q, J = 7.9 Hz, 6H).

^{13}C-NMR (126 MHz, $CDCl_3$) δ = 141.9, 128.4, 128.3, 125.8, 97.5, 81.4, 69.2, 64.4, 39.0, 30.7, 20.0, 6.8, 5.1.

HRMS (ESI-/Q-TOF): Calculated for $[M+Na]^+$ = $[C_{19}H_{30}NaO_2Si]^+$: m/z 341.19073; Found: m/z 341.19096

TLC: Rf: 0.49 using hexanes : ethyl acetate 20:1 (V/V) as an eluent; Visualization: $KMnO_4$

Compound 43: triethyl(furan-2-yl(methoxy)methoxy)silane

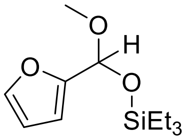

43

Compound **43** was prepared according to General procedure D, starting from methyl furan-2-carboxylate on a 5.00 mmol scale, using 0.5 mol% **1g** as a catalyst, yielding 990 mg (4.08 mmol) product, which corresponds to a yield of 82%. (See NMR spectra)

Physical state: clear liquid

^{1}H-NMR (500 MHz, C_6D_6) δ = 7.09 (dd, J = 1.8, 0.9 Hz, 1H), 6.40 (dt, J = 3.3, 0.9 Hz, 1H), 6.07 (dd, J = 3.2, 1.8 Hz, 1H), 5.86 (s, 1H), 3.18 (s, 3H), 0.98 (t, J = 8.0 Hz, 9H), 0.61 (q, J = 7.9 Hz, 6H).

^{13}C-NMR (126 MHz, C_6D_6) δ = 154.5, 142.2, 110.3, 107.8, 92.8, 51.7, 6.9, 5.3.

HRMS (ESI-/Q-TOF): Calculated for $[M+Na]^+$ = $[C_{12}H_{22}NaO_3Si]^+$: m/z 265.12304; Found: m/z 265.12323

TLC: Rf: 0.44 using hexanes : ethyl acetate 20:1 (V/V) as an eluent; Visualization: $KMnO_4$

Compound 44: (1-ethoxy-2-(thiophen-2-yl)ethoxy)triethylsilane

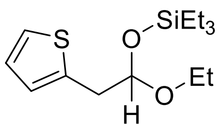

44

Compound **44** was prepared according to General procedure D, starting from ethyl 2-(thiophen-2-yl)acetate on a 2.00 mmol scale, yielding 546 mg (1.91 mmol) product, which corresponds to a yield of 95%. (See NMR spectra)

Physical state: pale yellow oil

^{1}H-NMR (500 MHz, $CDCl_3$) δ = 7.15 (dd, J = 5.1, 1.2 Hz, 1H), 6.93 (dd, J = 5.1, 3.5 Hz, 1H), 6.86 (dd, J = 3.5, 1.1 Hz, 1H), 4.92 (dd, J = 6.3, 4.3 Hz, 1H), 3.74 (dq, J = 9.0, 7.0 Hz, 1H), 3.45 (dq, J = 9.1, 7.0 Hz, 1H), 3.15 (ddd, J = 14.7, 6.2, 1.0 Hz, 1H), 3.06 (dd, J = 14.7, 4.3 Hz, 1H), 1.22 (t, J = 7.0 Hz, 3H), 0.97 (t, J = 8.0 Hz, 9H), 0.63 (q, J = 7.8 Hz, 6H).

^{13}C-NMR (126 MHz, $CDCl_3$) δ = 139.2, 126.4, 125.9, 124.1, 98.1, 62.2, 38.6, 15.1, 6.7, 5.0.

HRMS (ESI-/Q-TOF): Calculated for $[M+Na]^+$ = $[C_{14}H_{26}NaO_2SSi]^+$: m/z 309.13150; Found: m/z 309.13215

TLC: Rf: 0.51 using hexanes : ethyl acetate 20:1 (V/V) as an eluent; Visualization: $KMnO_4$

Compound 45: ((5-(1,2-dithiolan-3-yl)-1-methoxypentyl)oxy)triethylsilane

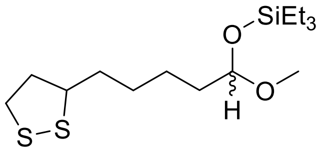

45

Compound **45** was prepared according to General procedure D, starting from methyl 5-(1,2-dithiolan-3-yl)pentanoate on a 5.00 mmol scale, using 0.1 mol% **1f** as a catalyst, yielding 1.54 g (4.57 mmol) product, which corresponds to a yield of 92%. (See NMR spectra)

Physical state: pale yellow oil

Diastereomer 1

^{1}H-NMR (500 MHz, $CDCl_3$) δ = 4.72 – 4.62 (m, 1H), 3.60 – 3.51 (m, 1H), 3.31 (s, 3H), 3.22 – 3.13 (m, 1H), 3.13 – 3.06 (m, 1H), 2.50 – 2.37 (m, 1H), 1.94 – 1.84 (m, 1H), 1.73 – 1.63 (m, 2H), 1.63 – 1.49 (m, 2H), 1.49 – 1.31 (m, 4H), 1.00 – 0.93 (m, 9H), 0.63 (q, J = 8.0 Hz, 6H).

^{13}C-NMR (126 MHz, $CDCl_3$) δ = 98.9, 56.5, 53.6, 40.2, 38.4, 37.1, 34.9, 29.2, 24.1, 6.7, 5.0.

Diastereomer 2

^{1}H-NMR (500 MHz, $CDCl_3$) δ = 4.72 – 4.62 (m, 1H), 3.60 – 3.51 (m, 1H), 3.31 (s, 3H), 3.22 – 3.13 (m, 1H), 3.13 – 3.06 (m, 1H), 2.50 – 2.37 (m, 1H), 1.94 – 1.84 (m, 1H), 1.73 – 1.63 (m, 2H), 1.63 – 1.49 (m, 2H), 1.49 – 1.31 (m, 4H), 1.00 – 0.93 (m, 9H), 0.63 (q, J = 8.0 Hz, 6H).

^{13}C-NMR (126 MHz, $CDCl_3$) δ = 98.9, 56.5, 53.6, 40.2, 38.4, 37.0, 34.8, 29.1, 24.0, 6.7, 5.0.

HRMS (ESI-/Q-TOF): Calculated for $[M+Na]^+$ = $[C_{15}H_{32}NaO_2S_2Si]^+$: m/z 359.15052; Found: m/z 359.15003

TLC: Rf: 0.63 using hexanes : ethyl acetate 10:1 (V/V) as an eluent; Visualization: $KMnO_4$

Compound 46: 3-(2-methoxy-2-((triethylsilyl)oxy)ethyl)-1*H*-indole

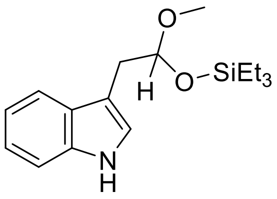

46

Compound **46** was prepared according to General procedure D, starting from methyl 2-(1*H*-indol-3-yl)acetate on a 5.00 mmol scale, using 0.25 mol% **1h** as a catalyst, yielding 1.45 g (4.75 mmol) product, which corresponds to a yield of 95%. (See NMR spectra)

Physical state: pale yellow oil

^{1}H-NMR (500 MHz, $CDCl_3$) δ = 7.99 (bs, 1H), 7.65 – 7.62 (m, 1H), 7.35 (dt, *J* = 8.2, 1.0 Hz, 1H), 7.19 (ddd, *J* = 8.1, 6.9, 1.2 Hz, 1H), 7.12 (ddd, *J* = 8.0, 7.0, 1.1 Hz, 1H), 7.08 (d, *J* = 2.3 Hz, 1H), 4.98 (dd, *J* = 6.5, 4.3 Hz, 1H), 3.36 (s, 3H), 3.10 (ddd, *J* = 14.8, 6.5, 1.0 Hz, 1H), 3.01 (ddd, *J* = 14.8, 4.3, 0.8 Hz, 1H), 0.96 (t, *J* = 8.0 Hz, 9H), 0.62 (q, *J* = 7.9 Hz, 6H).

^{13}C-NMR (126 MHz, $CDCl_3$) δ = 136.1, 127.9, 122.8, 121.8, 119.3, 119.0, 111.5, 111.0, 99.2, 53.8, 33.6, 6.7, 5.0.

HRMS (ESI-/Q-TOF): Calculated for $[M+Na]^+$ = $[C_{17}H_{27}NNaO_2Si]^+$: m/z 328.1703; Found: m/z 328.1699

TLC: Rf: 0.46 using hexanes : ethyl acetate 5:1 (V/V) as an eluent; Visualization: $KMnO_4$

Compound 47: 2-(ethoxy((triethylsilyl)oxy)methyl)benzo[*d*]thiazole

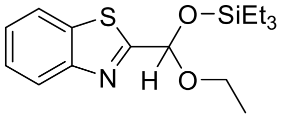

47

Compound **47** was prepared according to General procedure D, starting from ethyl benzo[*d*]thiazole-2-carboxylate on a 2.50 mmol scale, using 0.25 mol% catalyst, yielding 790 mg (2.44 mmol) product, which corresponds to a yield of 98%. (See NMR spectra)

Physical state: pale yellow oil

^{1}H-NMR (500 MHz, $CDCl_3$) δ = 8.04 (dd, *J* = 8.0, 1.2 Hz, 1H), 7.90 (dd, *J* = 8.0, 1.3 Hz, 1H), 7.47 (ddd, *J* = 8.3, 7.2, 1.3 Hz, 1H), 7.39 (td, *J* = 7.6, 7.2, 1.2 Hz, 1H), 6.06 (s, 1H), 3.70 (qd, *J* = 7.1, 1.6 Hz, 2H), 1.25 (t, *J* = 7.1 Hz, 3H), 0.99 (t, *J* = 7.9 Hz, 9H), 0.72 (q, *J* = 8.0 Hz, 6H).

^{13}C-NMR (126 MHz, $CDCl_3$) δ = 172.8, 153.1, 135.1, 125.9, 125.3, 123.5, 121.9, 94.6, 61.6, 15.1, 6.6, 4.9.

HRMS (ESI-/Q-TOF): Calculated for $[M+H]^+$ = $[C_{16}H_{26}NO_2SSi]^+$: m/z 324.1448; Found: m/z 324.1443

TLC: Rf: 0.37 using hexanes : ethyl acetate 20:1 (V/V) as an eluent; Visualization: $KMnO_4$

Compound 48: 3-bromo-5-(ethoxy((triethylsilyl)oxy)methyl)isoxazole

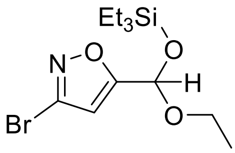

48

Compound **48** was prepared according to General procedure D, starting from ethyl 3-bromoisoxazole-5-carboxylate on a 2.50 mmol scale, using 0.50 mol% catalyst, yielding 810 mg (2.41 mmol) product, which corresponds to a yield of 96%. (See NMR spectra)

Physical state: pale yellow oil

^{1}H-NMR (500 MHz, $CDCl_3$) δ = 6.38 (d, J = 0.7 Hz, 1H), 5.86 (d, J = 0.8 Hz, 1H), 3.66 – 3.54 (m, 2H), 1.22 (t, J = 7.1 Hz, 3H), 0.97 (t, J = 7.9 Hz, 9H), 0.68 (q, J = 7.8 Hz, 6H).

^{13}C-NMR (126 MHz, $CDCl_3$) δ = 173.2, 140.1, 105.7, 90.1, 61.4, 15.0, 6.5, 4.7.

HRMS (ESI-/Q-TOF): Calculated for $[M+H]^+$ = $[C_{12}H_{23}BrNO_3Si]^+$: m/z 336.063; Found: m/z 336.062

TLC: Rf: 0.54 using hexanes : ethyl acetate 20:1 (V/V) as an eluent; Visualization: $KMnO_4$

Compound 49: 2-(ethoxy((triethylsilyl)oxy)methyl)pyridine

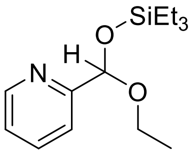

49

Compound **49** was prepared according to General procedure D, starting from ethyl picolinate on a 2.50 mmol scale, using 2.0 mol% **1d** as a catalyst and 1.2 equiv. TESH (**3**), yielding after purification (column chromatography on silica gel, with gradient elution using dichloromethane and methanol as eluents) 480 mg (1.79 mmol) product, which corresponds to a yield of 72%. (See NMR spectra)

Physical state: pale orange oil

^{1}H-NMR (500 MHz, $CDCl_3$) δ = 8.54 (ddd, J = 4.9, 1.8, 1.0 Hz, 1H), 7.72 (td, J = 7.7, 1.7 Hz, 1H), 7.63 (dt, J = 7.8, 1.2 Hz, 1H), 7.22 (ddd, J = 7.5, 4.9, 1.3 Hz, 1H), 5.76 (s, 1H), 3.73 (dq, J = 9.1, 7.1 Hz, 1H), 3.58 (dq, J = 9.2, 7.1 Hz, 1H), 1.23 (t, J = 7.1 Hz, 3H), 0.92 (t, J = 7.9 Hz, 9H), 0.67 – 0.59 (m, 6H).

^{13}C-NMR (126 MHz, $CDCl_3$) δ = 160.4, 148.3, 136.8, 123.3, 120.5, 98.1, 62.2, 15.1, 6.6, 4.8.

HRMS (ESI-/Q-TOF): Calculated for $[M+H]^+$ = $[C_{14}H_{26}NO_2Si]^+$: m/z 268.1727; Found: m/z 268.1728

TLC: Rf: 0.38 using hexanes : ethyl acetate 7:1 (V/V) as an eluent; Visualization: $KMnO_4$

Compound 50: 6-bromo-4-(ethoxy((triethylsilyl)oxy)methyl)-2-methylquinoline

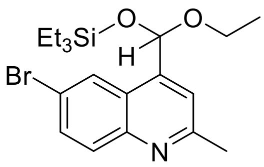

50

Compound **50** was prepared according to General procedure D, starting from ethyl 6-bromo-2-methylquinoline-4-carboxylate on a 2.50 mmol scale, using 0.50 mol% **1d** as a catalyst, yielding 982 mg (2.39 mmol) product, which corresponds to a yield of 96%. (See NMR spectra)

Physical state: pale yellow oil

^{1}H-NMR (500 MHz, $CDCl_3$) δ = 8.44 (d, J = 2.2 Hz, 1H), 7.89 (d, J = 8.9 Hz, 1H), 7.73 (dd, J = 8.9, 2.2 Hz, 1H), 7.47 (s, 1H), 6.18 (d, J = 0.8 Hz, 1H), 3.58 (dq, J = 9.2, 7.1 Hz, 1H), 3.50 (dq, J = 9.2, 7.1 Hz, 1H), 2.73 (s, 3H), 1.19 (t, J = 7.1 Hz, 3H), 0.95 (t, J = 7.9 Hz, 9H), 0.71 – 0.63 (m, 6H).

^{13}C-NMR (126 MHz, $CDCl_3$) δ = 159.2, 147.1, 144.7, 132.5, 130.8, 127.0, 125.2, 120.1, 119.7, 94.7, 60.9, 25.5, 15.2, 6.6, 4.9.

HRMS (ESI-/Q-TOF): Calculated for $[M+H]^+$ = $[C_{19}H_{29}BrNO_2Si]^+$: m/z 410.1145; Found: m/z 410.1142

TLC: Rf: 0.32 using hexanes : ethyl acetate 10:1 (V/V) as an eluent; Visualization: $KMnO_4$

Compound 51: 1-benzhydryl-3-(methoxy((triethylsilyl)oxy)methyl)azetidine

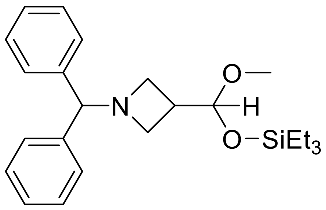

51

Compound **51** was prepared according to General procedure D, starting from methyl 1-benzhydrylazetidine-3-carboxylate on a 5.00 mmol scale, using 0.50 mol% catalyst, yielding 1.92 g (4.82 mmol) product, which corresponds to a yield of 96%. (See NMR spectra)

Physical state: pale yellow oil

^{1}H-NMR (500 MHz, $CDCl_3$) δ = 7.41 – 7.37 (m, 4H), 7.29 – 7.23 (m, 4H), 7.20 – 7.15 (m, 2H), 4.85 (d, J = 6.4 Hz, 1H), 4.33 (s, 1H), 3.29 (s, 3H), 3.25 (dt, J = 11.2, 7.7 Hz, 2H), 2.95 (dt, J = 18.6, 7.2 Hz, 2H), 2.74 – 2.64 (m, 1H), 0.97 (t, J = 7.9 Hz, 9H), 0.63 (q, J = 7.9 Hz, 6H).

^{13}C-NMR (126 MHz, $CDCl_3$) δ = 142.33, 142.31, 128.4, 127.54, 127.51, 126.99, 126.97, 100.2, 77.9, 55.2, 54.8, 53.3, 35.4, 6.8, 5.2.

HRMS (ESI-/Q-TOF): Calculated for $[M+H]^+$ = $[C_{24}H_{36}NO_2Si]^+$: m/z 398.2510; Found: m/z 398.2506

TLC: Rf: 0.73 using hexanes : ethyl acetate 3:1 (V/V) as an eluent; Visualization: $KMnO_4$

Compound 52: triethylsilyl (3-methoxy-3-((triethylsilyl)oxy)propyl)carbamate

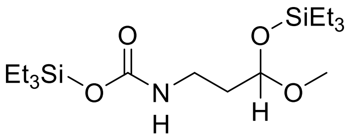

52

Compound **52** was prepared according to General procedure D, starting from methyl 3-((tert-butoxycarbonyl)amino)propanoate on a 2.00 mmol scale, using 1.0 mol% **1h** as a catalyst and 2.1 equiv. TESH (**3**), yielding 695 mg (1.84 mmol) product, which corresponds to a yield of 92%. (See NMR spectra)

Physical state: clear oil

^{1}H-NMR (500 MHz, C_6D_6) δ = 4.92 (t, J = 5.9 Hz, 1H), 4.59 (t, J = 4.7 Hz, 1H), 3.27 (tq, J = 13.6, 7.1 Hz, 2H), 3.06 (s, 3H), 1.72 – 1.58 (m, 2H), 1.05 (t, J = 8.0 Hz, 9H), 0.96 (t, J = 7.9 Hz, 9H), 0.89 – 0.82 (m, 6H), 0.56 (q, J = 7.9 Hz, 6H).

^{13}C-NMR (126 MHz, C_6D_6) δ = 155.2, 98.4, 53.6, 37.1, 36.9, 7.0, 6.9, 5.4, 5.3.

HRMS (ESI-/Q-TOF): Calculated for $[M+Na]^+$ = $[C_{17}H_{39}NNaO_4Si_2]^+$: m/z 400.2310; Found: m/z 400.2297

TLC: Rf: 0.58 using dichloromethane : 2-propanol 5:1 + 1% triethylamine (V/V) as an eluent; Visualization: $KMnO_4$

Compound 53: 2,4,6-trichloro-*N*-(4-ethoxy-4-((triethylsilyl)oxy)butyl)benzamide

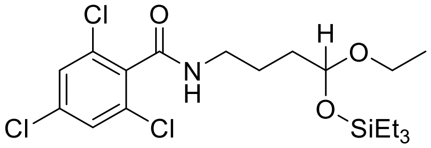

53

Compound **53** was prepared according to General procedure D, starting from ethyl 4-(2,4,6-trichlorobenzamido)butanoate (**S25**) on a 2.50 mmol scale, using 0.50 mol% **1g** as a catalyst and 1.5 equiv. TESH (**3**), yielding 1.02 g (2.24 mmol) product, which corresponds to a yield of 90%. (See NMR spectra)

Physical state: white solid, melting point: 49.0–49.6°C

^{1}H-NMR (500 MHz, $CDCl_3$) δ = 7.33 (s, 2H), 6.25 – 6.18 (m, 1H), 4.83 – 4.79 (m, 1H), 3.68 (dq, J = 9.2, 7.1 Hz, 1H), 3.49 (p, J = 7.1, 6.7 Hz, 2H), 3.37 (dq, J = 9.2, 7.0 Hz, 1H), 1.78 – 1.68 (m, 4H), 1.10 (t, J = 7.0 Hz, 3H), 0.96 (t, J = 7.9 Hz, 9H), 0.62 (q, J = 8.0 Hz, 6H).

^{13}C-NMR (126 MHz, $CDCl_3$) δ = 163.5, 135.6, 135.0, 133.0, 128.1, 97.4, 62.1, 39.8, 34.8, 23.6, 15.0, 6.7, 5.0.

HRMS (ESI-/Q-TOF): Calculated for $[M+Na]^+$ = $[C_{19}H_{30}Cl_3NNaO_3Si]^+$: m/z 476.0953; Found: m/z 476.0949

TLC: Rf: 0.38 using hexanes : ethyl acetate 5:1 (V/V) as an eluent; Visualization: $KMnO_4$

Compound 54: 2-(4-ethoxy-4-((triethylsilyl)oxy)butyl)isoindoline-1,3-dione

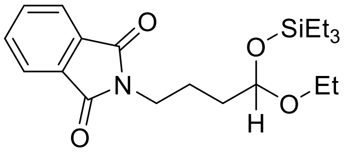

54

Compound **54** was prepared according to General procedure D, starting from ethyl 4-(1,3-dioxoisoindolin-2-yl)butanoate on a 5.00 mmol scale, using 1.0 mol% **1g** as a catalyst. The crude product was purified by flash chromatography, yielding 835 mg (2.21 mmol) product, which corresponds to a yield of 44%. (See NMR spectra)

Physical state: clear oil

^{1}H-NMR (300 MHz, $CDCl_3$) δ = 7.80 (dt, J = 7.0, 3.5 Hz, 2H), 7.67 (dd, J = 5.5, 3.1 Hz, 2H), 4.76 (dd, J = 5.6, 4.4 Hz, 1H), 3.76 – 3.57 (m, 3H), 3.36 (dq, J = 9.1, 7.0 Hz, 1H), 1.81 – 1.66 (m, 2H), 1.66 – 1.51 (m, 2H), 1.14 (t, J = 7.1 Hz, 3H), 0.98 – 0.86 (m, 9H), 0.58 (q, J = 7.7 Hz, 6H).

^{13}C-NMR (75 MHz, $CDCl_3$) δ = 168.3, 133.8, 132.1, 123.0, 97.3, 61.8, 37.7, 34.8, 23.6, 15.1, 6.7, 5.0.

HRMS (ESI-/Q-TOF): Calculated for $[M+Na]^+$ = $[C_{20}H_{31}NNaO_4Si]^+$: m/z 400.19145; Found: m/z 400.19115

TLC: Rf: 0.40 using hexanes : ethyl acetate 10:1 (V/V) as an eluent; Visualization: $KMnO_4$

Compound 55: 4,5,6,7-tetrachloro-2-(4-ethoxy-4-((triethylsilyl)oxy)butyl)isoindoline-1,3-dione

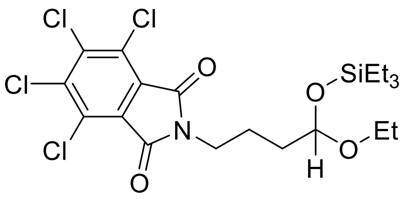

55

Compound **55** was prepared according to General procedure D, starting from ethyl 4-(4,5,6,7-tetrachloro-1,3-dioxoisoindolin-2-yl)butanoate (**S26**) on a 5.00 mmol scale, using 1.0 mol% **1g** as a catalyst, yielding 2.30 g (4.46 mmol) product, which corresponds to a yield of 89%. (See NMR spectra)

Physical state: white solid, melting point: 51.4–52.0°C

^{1}H-NMR (500 MHz, $CDCl_3$) δ = 4.79 (dd, J = 5.9, 4.2 Hz, 1H), 3.79 – 3.64 (m, 3H), 3.39 (dq, J = 9.3, 7.1 Hz, 1H), 1.82 – 1.72 (m, 2H), 1.68 – 1.57 (m, 2H), 1.17 (t, J = 7.0 Hz, 3H), 0.95 (t, J = 8.0 Hz, 9H), 0.62 (q, J = 8.0 Hz, 6H).

^{13}C-NMR (126 MHz, $CDCl_3$) δ = 163.5, 140.0, 129.6, 127.7, 97.2, 62.0, 38.7, 34.8, 23.3, 15.2, 6.8, 5.1.

HRMS (ESI-/Q-TOF): Calculated for $[M+Na]^+$ = $[C_{20}H_{27}Cl_4NNaO_4Si]^+$: m/z 536.03557; Found: m/z 536.03541

TLC: Rf: 0.49 using hexanes : ethyl acetate 10:1 (V/V) as an eluent; Visualization: $KMnO_4$

Compound 56: *N*,*N*-diisopropyl-6-methoxy-6-((triethylsilyl)oxy)hexanamide

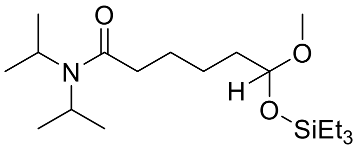

56

Compound **56** was prepared according to General procedure D, starting from methyl 6-(diisopropylamino)-6-oxohexanoate on a 5.00 mmol scale, using 1.0 mol% **1g** as a catalyst and 1.2 equiv. TESH (**3**), yielding 1.62 g (4.50 mmol) product, which corresponds to a yield of 90%. (See NMR spectra)

Physical state: clear oil

^{1}H-NMR (500 MHz, $CDCl_3$) δ = 4.69 (dd, *J* = 6.1, 4.2 Hz, 1H), 3.99 – 3.90 (m, 1H), 3.47 (bs, 1H), 3.31 (s, 3H), 2.26 (t, *J* = 7.7 Hz, 2H), 1.68 – 1.52 (m, 4H), 1.44 – 1.32 (m, 8H), 1.18 (d, *J* = 6.8 Hz, 6H), 0.97 (t, *J* = 7.9 Hz, 9H), 0.63 (q, *J* = 7.9 Hz, 6H).

^{13}C-NMR (126 MHz, $CDCl_3$) δ = 171.8, 99.0, 53.6, 48.2, 45.5, 37.2, 35.3, 25.3, 24.3, 21.0, 20.7, 6.8, 5.1.

HRMS (ESI-/Q-TOF): Calculated for $[M+Na]^+$ = $[C_{19}H_{41}NNaO_3Si]^+$: m/z 382.2748; Found: m/z 382.2745

TLC: Rf: 0.59 using hexanes : ethyl acetate 3:1 (V/V) as an eluent; Visualization: $KMnO_4$

Compound 57: (ethoxy(4-nitrophenyl)methoxy)triethylsilane

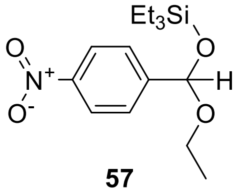

57

Compound **57** was prepared according to General procedure D, starting from ethyl 4-nitrobenzoate on a 5.00 mmol scale, using 2.0 mol% **1g** as a catalyst and 2.1 equiv. TESH (**3**), yielding after purification (column chromatography on silica gel, with gradient elution using hexanes and ethyl acetate as eluents) 790 mg (2.54 mmol) product, which corresponds to a yield of 51%. (See NMR spectra)

Physical state: pale orange oil

^{1}H-NMR (500 MHz, $CDCl_3$) δ = 8.21 (d, *J* = 8.7 Hz, 2H), 7.63 (d, *J* = 8.6 Hz, 2H), 5.84 (s, 1H), 3.53 (qd, *J* = 7.1, 2.9 Hz, 2H), 1.20 (t, *J* = 7.1 Hz, 3H), 0.95 (t, *J* = 7.9 Hz, 9H), 0.65 (q, *J* = 7.7 Hz, 6H).

^{13}C-NMR (126 MHz, $CDCl_3$) δ = 148.4, 147.9, 127.3, 123.5, 95.8, 60.8, 15.1, 6.6, 4.9.

HRMS (ESI-/Q-TOF): Calculated for $[M+Na]^+$ = $[C_{15}H_{25}NNaO_4Si]^+$: m/z 334.1445; Found: m/z 334.1437

TLC: Rf: 0.75 using hexanes : ethyl acetate 10:1 (V/V) as an eluent; Visualization: $KMnO_4$

Compound 58: (ethoxy(3-nitrophenyl)methoxy)triethylsilane

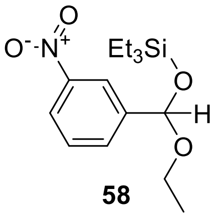

58

Compound **58** was prepared according to General procedure D, starting from ethyl 3-nitrobenzoate on a 5.00 mmol scale, using 4.0 mol% **1g** as a catalyst and 2.0 equiv. TESH (**3**), yielding after purification (column chromatography on silica gel, with gradient elution using hexanes and ethyl acetate as eluents) 622 mg (2.00 mmol) product, which corresponds to a yield of 40%. (See NMR spectra)

Physical state: pale orange oil

¹H-NMR (500 MHz, $CDCl_3$) δ = 8.33 (t, J = 2.0 Hz, 1H), 8.17 (ddd, J = 8.2, 2.4, 1.1 Hz, 1H), 7.80 (d, J = 7.7 Hz, 1H), 7.53 (t, J = 7.9 Hz, 1H), 5.85 (s, 1H), 3.60 – 3.49 (m, 2H), 1.21 (t, J = 7.0 Hz, 3H), 0.96 (t, J = 7.9 Hz, 9H), 0.66 (q, J = 7.7 Hz, 6H).

¹³C-NMR (126 MHz, $CDCl_3$) δ = 148.3, 143.8, 132.4, 129.2, 123.2, 121.5, 95.7, 60.9, 15.1, 6.6, 4.9.

HRMS (ESI-/Q-TOF): Calculated for $[M+Na]^+$ = $[C_{15}H_{25}NNaO_4Si]^+$: m/z 334.1445; Found: m/z 334.1441

TLC: Rf: 0.42 using hexanes : ethyl acetate 20:1 (V/V) as an eluent; Visualization: $KMnO_4$

Compound 59: (1-ethoxy-2-(2-nitrophenyl)ethoxy)triethylsilane

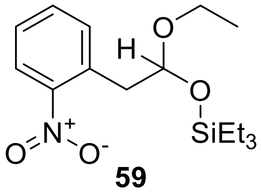

59

Compound **59** was prepared according to General procedure D, starting from ethyl 2-(2-nitrophenyl)acetate on a 5.00 mmol scale, using 2.0 mol% **1g** as a catalyst, yielding 1.61 g (4.94 mmol) product, which corresponds to a yield of 99%. (See NMR spectra)

Physical state: pale yellow oil

¹H-NMR (500 MHz, $CDCl_3$) δ = 7.88 (dd, J = 8.2, 1.3 Hz, 1H), 7.50 (td, J = 7.5, 1.3 Hz, 1H), 7.43 (dd, J = 7.7, 1.5 Hz, 1H), 7.36 (ddd, J = 8.6, 7.4, 1.5 Hz, 1H), 5.02 (dd, J = 5.7, 4.9 Hz, 1H), 3.68 (dq, J = 9.2, 7.0 Hz, 1H), 3.36 (dq, J = 9.2, 7.0 Hz, 1H), 3.24 – 3.14 (m, 2H), 1.11 (t, J = 7.0 Hz, 3H), 0.92 (t, J = 7.9 Hz, 9H), 0.59 (q, J = 7.8 Hz, 6H).

¹³C-NMR (126 MHz, $CDCl_3$) δ = 150.0, 134.4, 132.3, 132.1, 127.4, 124.3, 97.2, 62.4, 41.1, 15.0, 6.6, 4.9.

HRMS (ESI-/Q-TOF): Calculated for $[M+Na]^+$ = $[C_{16}H_{27}NNaO_4Si]^+$: m/z 348.1602; Found: m/z 348.1603

TLC: Rf: 0.67 using hexanes : ethyl acetate 10:1 (V/V) as an eluent; Visualization: $KMnO_4$

Compound 60: (1-ethoxy-3-nitropropoxy)triethylsilane

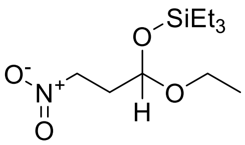

60

Compound **60** was prepared according to General procedure D, starting from ethyl 3-nitropropanoate on a 5.00 mmol scale, using 2.0 mol% **1g** as a catalyst, yielding 1.15 g (4.36 mmol) product, which corresponds to a yield of 87%. (See NMR spectra)

Physical state: pale yellow oil

^{1}H-NMR (500 MHz, $CDCl_3$) δ = 4.92 (s, 1H), 4.56 – 4.44 (m, 2H), 3.68 (p, J = 7.6 Hz, 1H), 3.38 (p, J = 7.7 Hz, 1H), 2.35 – 2.17 (m, 2H), 1.18 (t, J = 7.2 Hz, 3H), 0.97 (t, J = 6.9 Hz, 9H), 0.69 – 0.60 (m, 6H).

^{13}C-NMR (126 MHz, $CDCl_3$) δ = 94.5, 71.1, 62.5, 34.8, 15.0, 6.7, 4.9.

HRMS (ESI-/Q-TOF): Calculated for $[M+Na]^+$ = $[C_{11}H_{25}NNaO_4Si]^+$: m/z 286.1445; Found: m/z 286.1448

TLC: Rf: 0.59 using hexanes : ethyl acetate 10:1 (V/V) as an eluent; Visualization: $KMnO_4$

Compound 61: 4,10-bis(3-bromopropyl)-6,6,8,8-tetramethyl-3,5,7,9,11-pentaoxa-6,8-disilatridecane

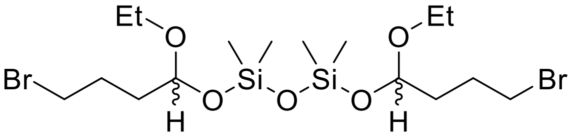

61

Compound **61** was prepared according to General procedure D, starting from 6.60 mmol ethyl 4-bromobutanoate, using 0.1 mol% **1g** as a catalyst and 0.55 equiv. TMDS (**S13**, instead of TESH) as a reducing agent, yielding 1.64 g (3.13 mmol) product, which corresponds to a yield of 95%. (See NMR spectra)

Physical state: clear oil

^{1}H-NMR (500 MHz, $CDCl_3$) δ = 4.88 (t, J = 5.1 Hz, 2H), 3.77 – 3.68 (m, 2H), 3.48 – 3.35 (m, 6H), 2.02 – 1.91 (m, 4H), 1.81 – 1.71 (m, 4H), 1.20 (t, J = 7.0 Hz, 6H), 0.20 – 0.13 (m, 12H).

^{13}C-NMR (126 MHz, $CDCl_3$) δ = 96.8, 62.5, 36.1, 33.8, 27.7, 15.1, 0.07, 0.06, -0.17, -0.18.

HRMS (ESI-/Q-TOF): Calculated for $[M+Na]^+$ = $[C_{16}H_{36}Br_2NaO_5Si_2]^+$: m/z 545.03602; Found: m/z 545.03592

TLC: Rf: 0.48 using hexanes : ethyl acetate 10:1 (V/V) as an eluent; Visualization: $KMnO_4$

Compound 63: (((3*S*,3a*S*,5a*S*,9b*S*)-3,5a,9-trimethyl-2,3,3a,4,5,5a,6,9b-octahydronaphtho[1,2-*b*]furan-2,8-diyl)bis(oxy))bis(triethylsilane)

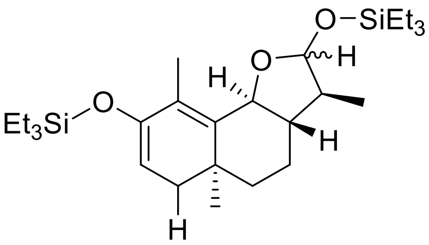

63

Compound **63** was prepared according to General procedure D, starting from α-Santonin on a 2.50 mmol scale, using 0.25 mol% **1h** as a catalyst and 2.1 equiv. TESH (**3**), yielding after purification (column chromatography on basic aluminium oxide, with gradient elution using hexanes and ethyl acetate as eluents) 908 mg (1.90 mmol) product, which corresponds to a yield of 76% (single diastereomer observed). (See NMR spectra)

Physical state: pale yellow oil

^{1}H-NMR (500 MHz, $CDCl_3$) δ = 5.38 (d, J = 5.2 Hz, 1H), 4.80 (dd, J = 7.0, 2.5 Hz, 1H), 3.99 (d, J = 11.1 Hz, 1H), 2.19 (d, J = 16.2 Hz, 1H), 2.03 (d, J = 2.2 Hz, 3H), 1.84 (dd, J = 16.1, 7.0 Hz, 1H), 1.80 – 1.73 (m, 1H), 1.70 – 1.53 (m, 3H), 1.34 (td, J = 13.3, 4.1 Hz, 1H), 1.25 – 1.16 (m, 1H), 1.02 – 0.93 (m, 24H), 0.70 (q, J = 7.8 Hz, 6H), 0.63 (qd, J = 7.9, 1.3 Hz, 6H).

^{13}C-NMR (126 MHz, $CDCl_3$) δ = 149.7, 137.9, 124.4, 99.5, 98.7, 83.6, 48.3, 43.5, 43.0, 39.7, 37.0, 24.1, 23.5, 13.2, 11.0, 6.8, 5.1, 4.9.

HRMS (ESI-/Q-TOF): Calculated for $[M+H]^+$ = $[C_{27}H_{51}O_3Si_2]^+$: m/z 479.3371; Found: m/z 479.3361

TLC: Rf: 0.64 using hexanes : ethyl acetate 20:1 (V/V) as an eluent, on neutral aluminium oxide TLC plate; Visualization: $KMnO_4$

Compound 64: (3*S*,3a*S*,5a*S*,9b*S*)-3,5a,9-trimethyl-2-((triethylsilyl)oxy)-2,3,3a,5,5a,6,7,9b-octahydronaphtho[1,2-*b*]furan-8(4*H*)-one

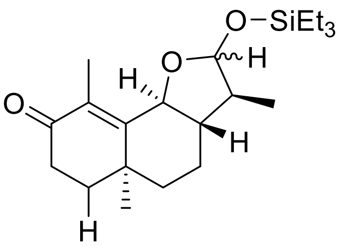

64

Compound **64** was prepared according to General procedure D, starting from α-Santonin on a 2.50 mmol scale, using 0.25 mol% **1h** as a catalyst and 2.1 equiv. TESH (**3**), yielding after purification (column chromatography on silica gel, with gradient elution using hexanes and ethyl acetate as eluents) 695 mg (1.91 mmol) product, which corresponds to a yield of 76% (single diastereomer observed). (See NMR spectra)

Physical state: pale yellow oil

^{1}H-NMR (500 MHz, $CDCl_3$) δ = 5.38 (d, J = 4.4 Hz, 1H), 4.22 (d, J = 9.4 Hz, 1H), 2.55 – 2.46 (m, 1H), 2.42 – 2.35 (m, 1H), 2.07 (d, J = 1.6 Hz, 3H), 1.91 – 1.83 (m, 2H), 1.78 – 1.71 (m, 3H), 1.68 – 1.62 (m, 1H), 1.48 (td, J = 13.2, 3.9 Hz, 1H), 1.44 – 1.35 (m, 1H), 1.24 (s, 3H), 0.99 – 0.93 (m, 12H), 0.62 (q, J = 8.2 Hz, 6H).

^{13}C-NMR (126 MHz, $CDCl_3$) δ = 199.8, 157.7, 127.7, 99.7, 83.8, 49.9, 44.1, 42.7, 38.4, 38.1, 33.8, 24.5, 24.4, 11.5, 11.0, 6.7, 4.8.

HRMS (ESI-/Q-TOF): Calculated for $[M+H]^+$ = $[C_{21}H_{37}O_3Si]^+$: m/z 365.2507; Found: m/z 365.2499

TLC: Rf: 0.38 using hexanes : ethyl acetate 7:1 (V/V) as an eluent; Visualization: $KMnO_4$

Compound 66: 1,3-bis(methoxy((triethylsilyl)oxy)methyl)bicyclo[1.1.1]pentane

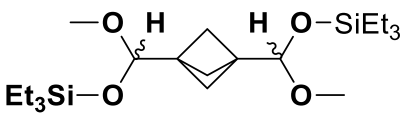

66

Compound **66** was prepared according to General procedure D, starting from dimethyl bicyclo[1.1.1]pentane-1,3-dicarboxylate on a 2.50 mmol scale, using 0.25 mol% catalyst and 2.1 equiv. TESH (**3**), yielding 989 mg (2.37 mmol) product, which corresponds to a yield of 95%. (See NMR spectra)

Physical state: clear oil

Mixture of diastereomers

^{1}H-NMR (500 MHz, $CDCl_3$) δ = 4.62 (s, 2H), 3.34 (s, 6H), 1.71 – 1.67 (m, 6H), 0.98 (t, J = 8.0 Hz, 18H), 0.64 (q, J = 8.0 Hz, 12H).

^{13}C-NMR (126 MHz, $CDCl_3$) δ = 96.84, 96.81, 54.6, 54.5, 46.65, 46.62, 41.4, 6.8, 5.1.

HRMS (ESI-/Q-TOF): Calculated for $[M+Na]^+$ = $[C_{21}H_{44}NaO_4Si_2]^+$: m/z 439.2670; Found: m/z 439.2664

TLC: Rf: 0.60 using hexanes : ethyl acetate 10:1 (V/V) as an eluent; Visualization: $KMnO_4$

Compound 67: 1,4-bis(methoxy((triethylsilyl)oxy)methyl)cubane

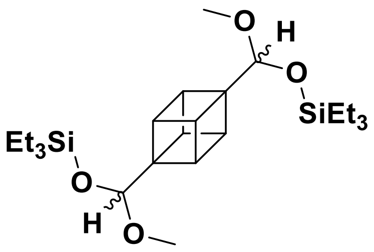

67

Compound **67** was prepared according to General procedure D, starting from cubane-1,4-dicarboxylic acid dimethyl ester on a 2.50 mmol scale, using 0.25 mol% catalyst and 2.1 equiv. TESH (**3**), yielding 1.11 g (2.45 mmol) product, which corresponds to a yield of 98%. (See NMR spectra)

Physical state: clear oil

Mixture of diastereomers

^{1}H-NMR (500 MHz, $CDCl_3$) δ = 4.81 (s, 2H), 3.85 (s, 6H), 3.34 (s, 6H), 0.97 (t, J = 8.0 Hz, 18H), 0.64 (q, J = 7.9 Hz, 12H).

^{13}C-NMR (126 MHz, $CDCl_3$) δ = 97.69, 97.65, 60.37, 60.35, 54.0, 53.9, 43.80, 43.79, 6.8, 5.2.

HRMS (ESI-/Q-TOF): Calculated for $[M+Na]^+$ = $[C_{24}H_{44}NaO_4Si_2]^+$: m/z 475.2670; Found: m/z 475.2661

TLC: Rf: 0.59 using hexanes : ethyl acetate 10:1 (V/V) as an eluent; Visualization: $KMnO_4$

1.4.3 Limitations of this methodology

This methodology for the partial reduction of esters has limitations in the substrate scope (Fig. S6). This means, that using the General procedure D, we were not able to reduce their ester functionalities and convert them into silyl acetal moieties effectively.

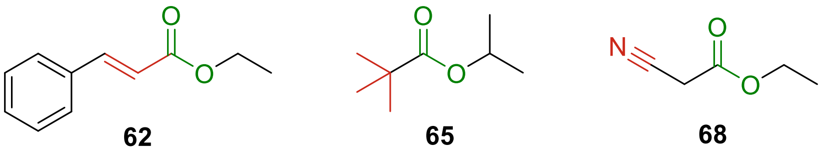

62 α,β-unsaturated esters

65 sterically hindered α-position

68 nitrile group

Fig. S6. Limitations of the novel methodology

In the case of substrate **62**, the α,β-unsaturated ester functionality reacted in a 1,4-addition (instead of the 1,2-addition), resulting in the formation of a silyl ketene acetal. In the case of substrate **65**, the large steric hindrance of the α-position resulted in no observable conversion. Finally, in case of substrate **68**, the significantly more Lewis basic (and sterically nondemanding) nitrile group resulted in no observable conversion of the ester group. On the other hand, reduction of the nitrile group occurred at higher catalyst loadings.

1.4.4 Synthesis of starting materials

Most of the ester substrates used in this study are commercially available compounds that were used as received from vendors, without any additional purification. A few esters were synthesized from their respective carboxylic acids via literature procedures, usually using Fisher or Steglich esterification. These compounds were purified via vacuum distillation or flash chromatography.

The following substrates are novel compounds that were previously not reported in the literature. Their synthesis is described herein.

Synthesis of **S24**

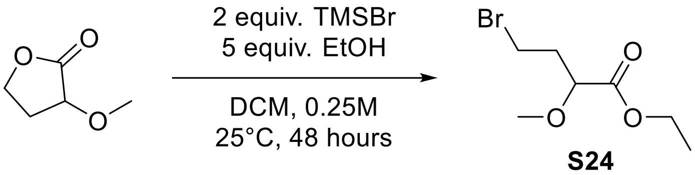

S24

In a 25 mL flask, equipped with a rubber septa and nitrogen gas inlet, 446 mg (1 equiv., 4.00 mmol) lactone was measured in, followed by the addition of 15 mL dichloromethane (DCM). Next, ethanol (921 mg, 1.17 mL, 5 equiv., 20.0 mmol) was added and the reaction mixture was cooled to 0°C (using an ice bath). Finally, bromotrimethylsilane (TMSBr, 1.22 g, 1.06 mL, 2 equiv., 8.00 mmol) was added dropwise. After completion of the addition, the reaction mixture was stirred at room temperature for 48 hours. After complete conversion has been achieved (based on TLC analysis), the reaction was quenched by the addition of 10 mL of water. The phases were separated, and the aqueous phase was further washed with 2x10 mL DCM. The combined organic phase was washed with 20% (*m*/*m*) sodium thiosulfate, dried using sodium sulfate, filtered through a layer of silica gel, and finally evaporated under reduced pressure using a rotary evaporator. This resulted in 470 mg (2.09 mmol) of **S24** corresponding to a yield of 52%. The ester was used without further purification.

Compound S24: ethyl 4-bromo-2-methoxybutanoate (See NMR spectra)

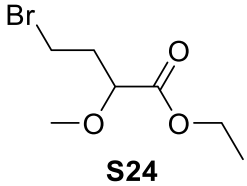

S24

Physical state: clear liquid

^{1}H-NMR (500 MHz, $CDCl_3$) δ = 4.23 (qd, J = 7.1, 3.1 Hz, 2H), 3.96 – 3.92 (m, 1H), 3.58 – 3.46 (m, 2H), 3.43 (s, 3H), 2.25 – 2.19 (m, 2H), 1.29 (t, J = 7.1 Hz, 3H).

^{13}C-NMR (126 MHz, $CDCl_3$) δ = 172.0, 78.1, 61.1, 58.5, 35.7, 28.9, 14.2.

HRMS (ESI-/Q-TOF): Calculated for $[M+Na]^+$ = $[C_7H_{13}BrNaO_3]^+$: m/z 246.99403; Found: m/z 246.99311

TLC: Rf: 0.20 using hexanes : ethyl acetate 20:1 (V/V) as an eluent; Visualization: $KMnO_4$

Synthesis of **S25**

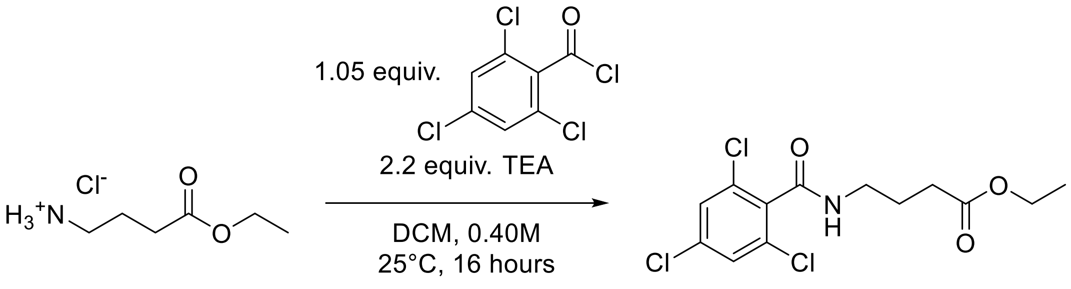

In a 50 mL flask, equipped with a magnetic stirrer and a $CaCl_2$ filled drying tube, ethyl 4-aminobutanoate hydrochloride (2.00 g, 1 equiv., 11.9 mmol) was suspended in 30 mL DCM (stabilized with amylene). Next, the mixture was cooled to 0°C, and triethylamine (2.66 g, 3.66 mL, 2.2 equiv., 26.2 mmol) was added dropwise. After 10 minutes, 2,4,6-trichlorobenzoyl chloride (3.06 g, 1.96 mL, 1.05 equiv., 12.5 mmol) was added dropwise, keeping the reaction temperature below 6°C. After completion of the addition, the reaction mixture was stirred at room temperature for 16 hours. After complete conversion was achieved (as shown by TLC analysis), the reaction mixture was filtered over a thin layer of Celite, which in turn was washed with 20 mL DCM. The combined organic phase was washed with 1M aqueous hydrochloric acid, saturated aqueous sodium bicarbonate, and brine, dried using sodium sulfate, filtered, and finally evaporated under reduced pressure using a rotary evaporator. The crude product was purified using flash chromatography, which resulted in 3.7 g (11 mmol) of **S25** corresponding to a yield of 92%.

Compound S25: ethyl 4-(2,4,6-trichlorobenzamido)butanoate (See NMR spectra)

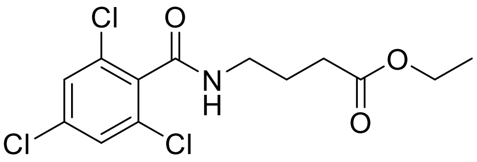

S25

Physical state: white solid, melting point: 76.0–76.8°C

^{1}H-NMR (500 MHz, $CDCl_3$) δ 7.33 (s, 2H), 6.08 (bs, 1H), 4.13 (q, J = 7.1 Hz, 2H), 3.53 (td, J = 6.8, 5.8 Hz, 2H), 2.46 (t, J = 7.1 Hz, 2H), 1.97 (p, J = 7.0 Hz, 2H), 1.26 (t, J = 7.1 Hz, 3H).

^{13}C-NMR (126 MHz, $CDCl_3$) δ = 173.4, 163.8, 135.8, 134.7, 132.9, 128.1, 60.7, 39.5, 31.7, 24.2, 14.2.

HRMS (ESI-/Q-TOF): Calculated for $[M+H]^+$ = $[C_{13}H_{15}Cl_3NO_3]^+$: m/z 338.0112; Found: m/z 338,0108

TLC: Rf: 0.27 using hexanes : ethyl acetate 3:1 (V/V) as an eluent; Visualization: $KMnO_4$

Synthesis of **S26**

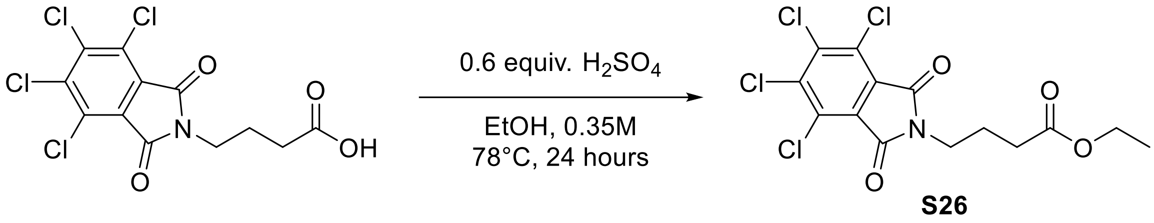

In a 100 mL flask, equipped with a magnetic stirrer and a reflux condenser, 7.4 g (1 equiv., 20 mmol) of 4-(4,5,6,7-tetrachloro-1,3-dioxoisoindolin-2-yl)butanoic acid was dissolved in 58 mL (46 g, 50 equiv., 1.0 mol) anhydrous ethanol. Next, while stirring, 0.64 mL (1.2 g, 0.6 equiv., 12 mmol) sulfuric acid (98%) was added dropwise. The reaction mixture was heated to reflux and stirred for 24 hours. After complete conversion has been achieved (as shown by TLC analysis), the reaction mixture was concentrated under reduced pressure (using a rotary evaporator) to about half the volume. Then, 100 mL ethyl acetate and 50 mL water were added, and the phases were separated. The aqueous phase was further extracted with 2x25 mL ethyl acetate. The combined organic phase was washed with saturated aqueous sodium bicarbonate and brine, dried using sodium sulfate, filtered, and finally evaporated under reduced pressure using a rotary evaporator. The crude product was purified using flash chromatography. This resulted in 3.5 g (8.8 mmol) of **S26** corresponding to a yield of 44%.

Compound S26: ethyl 4-(4,5,6,7-tetrachloro-1,3-dioxoisoindolin-2-yl)butanoate (See NMR spectra)

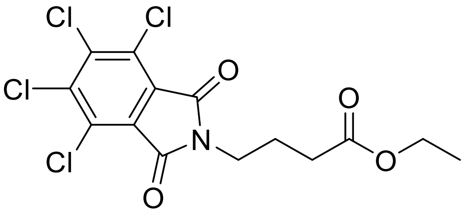

S26

Physical state: white solid, melting point: 117.5–118.0°C

^{1}H-NMR (500 MHz, $CDCl_3$) δ = 4.11 (q, J = 7.1 Hz, 2H), 3.77 (t, J = 6.8 Hz, 2H), 2.37 (t, J = 7.3 Hz, 2H), 2.02 (p, J = 7.1 Hz, 2H), 1.25 (t, J = 7.1 Hz, 3H).

^{13}C-NMR (126 MHz, $CDCl_3$) δ = 172.4, 163.6, 140.1, 129.7, 127.6, 60.6, 38.2, 31.6, 23.5, 14.2.

HRMS (ESI-/Q-TOF): Calculated for $[M+Na]^+$ = $[C_{14}H_{11}Cl_4NNaO_4]^+$: m/z 419.93344; Found: m/z 419.93315

TLC: Rf: 0.35 using hexanes : ethyl acetate 10:1 (V/V) as an eluent; Visualization: $KMnO_4$

1.5 Applications

1.5.1 Kilogram-scale laboratory reduction of fatty acid esters

To highlight the scalability of the fiddler crab-type borane catalyzed partial hydrosilylation reaction, a kilogram-scale reaction is presented for the reduction of fatty acid esters under laboratory conditions. The reaction was conducted following General procedure D, using standard laboratory setup, at ambient temperature, without the need for solvent.

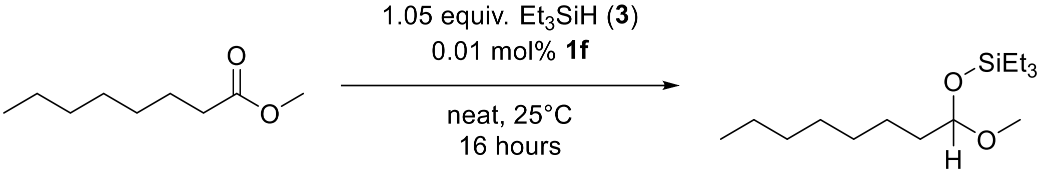

69 **70**: 99+% [1.4 kg]

In an oven-dried 2000 mL 3-necked flask, equipped with a magnetic stirrer, an immersion probe digital thermometer, a nitrogen inlet with a bubbler, and a pressure-compensated addition funnel, methyl octanoate (**69**, 791 g, 902 mL, 1 equiv, 5.00 mol) was measured in. After that, a stock solution of the **1f** borane catalyst (223 mg, 10.0 mL, 0.05 M in anhydrous toluene, 0.0001 equiv., 500 μmol) was added in one portion. Finally, the addition funnel was filled with triethylsilane (**3**, 610 g, 839 mL, 1.05 equiv., 5.25 mol), and the first 300 mL of TESH was added to the reaction mixture in one portion. After a short induction period (usually about 30 min. [Note 1.]), gas started to evolve in the reaction mixture and the temperature started to rise. (If needed, a cold water bath can be used to keep the reaction temperature below 40°C.) After the temperature starts to lower, dropwise addition of the remaining TESH was continued to maintain the reaction temperature between 30-35°C. After the addition was complete (usually in about 1 hour), stirring of the reaction mixture was continued at room temperature until TLC analysis showed the complete conversion of the ester [Note 2.]. After completion of the reaction, 5 mL of acetonitrile was added to quench the borane catalyst, followed by the filtration of the mixture through a silica gel layer. The silica gel was washed with 200 mL of hexanes : ethyl acetate 10:1 (V/V), after which the combined organic phase was concentrated under reduced pressure using a rotary evaporator (with a 40°C water bath) to give 1.37 kg (4.99 mol) of **70** as a clear liquid (Yield: 99+%).

[Note 1.] If the induction period is too long, additional 100 mL of TESH can be added to the reaction mixture.
[Note 2.] If complete conversion isn't reached within a day, additional TESH can be added to the reaction. NMR analysis of the crude reaction mixture can indicate the exact amount needed.

Compound 70: triethyl((1-methoxyoctyl)oxy)silane (See NMR spectra)

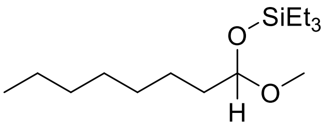

70

Physical state: clear liquid

^{1}H-NMR (500 MHz, $CDCl_3$) δ = 4.68 (dd, J = 6.1, 4.4 Hz, 1H), 3.32 (s, 3H), 1.65 – 1.49 (m, 2H), 1.40 – 1.21 (m, 10H), 0.98 (t, J = 8.0 Hz, 9H), 0.91 – 0.85 (m, 3H), 0.65 (q, J = 7.9 Hz, 6H).

^{13}C-NMR (126 MHz, $CDCl_3$) δ = 99.3, 53.6, 37.3, 31.8, 29.5, 29.3, 24.4, 22.6, 14.1, 6.8, 5.1.

HRMS (ESI-/Q-TOF): Calculated for $[M+Na]^+$ = $[C_{15}H_{34}NaO_2Si]^+$: m/z 297.22203; Found: m/z 297.22324

TLC: Rf: 0.58 using hexanes : ethyl acetate 20:1 (V/V) as an eluent; Visualization: $KMnO_4$

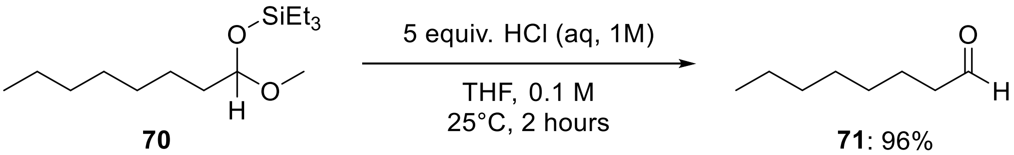

The acidic hydrolysis of acetal **70** to octanal (**71**) is demonstrated. In a 250 mL flask, equipped with a stirring bar, 2.75 g of **70** (1 equiv., 10.0 mmol) was diluted with tetrahydrofuran (THF, 100 mL, technical grade). Next, 50 mL of aqueous hydrochloric acid (1.82 g, 1 M, 5 equiv., 50.00 mmol) was slowly added. The resulting biphasic system was intensively stirred for 2 hours at room temperature, until complete conversion of the silyl acetal was achieved (the reaction was monitored by TLC). Afterwards, 50 mL brine was added to the reaction mixture followed by the separation of the phases. The aqueous phase was further extracted with 2x25 mL diethyl ether. The combined organic phases were washed with sat. aqueous $NaHCO_3$ and brine. The organic phase was dried using sodium sulfate, filtered, and concentrated under reduced pressure using a rotary evaporator [Note 1.]. The obtained crude product was further purified by flash chromatography on silica gel to remove the triethylsilanol and hexaethyldisiloxane by-products. This resulted in 1.24 g (9.64 mmol) of pure aldehyde **71** with a yield of 96%. The NMR spectral data of the final product is in accordance with literature data.

[Note 1.] Due to the volatility of octanal, care must be taken during evaporation. Also, the temperature of the water bath was lowered to 30°C.

1.5.2 Reduction of Roche ester

The benzylated Roche ester **72** ((*S*)-enantiomer) was selectively reduced to acetal **74** by using the General procedure D with the fiddler crab-type borane **1f**.

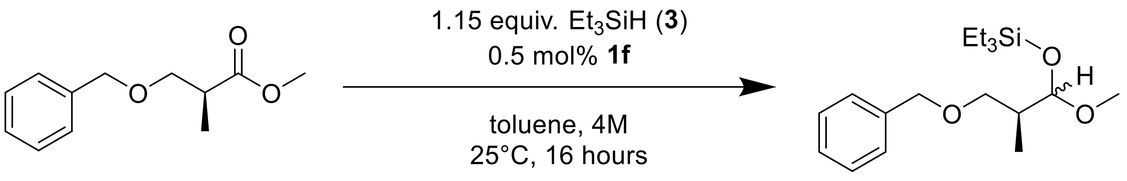

72 **74**: 87%

In a 4 mL oven-dried screw-capped vial equipped with a rubber seal and a magnetic stirrer, 1.04 g (1 equiv., 5.00 mmol) of (*S*)-Roche ester **72** was measured in. The vial was capped, flushed with argon gas, and an argon-filled balloon was connected to it. Afterwards, 0.75 mL anhydrous toluene was added to the vial, followed by the addition of the **1f** catalyst solution (11.2 mg, 500 µL, 0.05 M in anhydrous toluene, 0.005 equiv., 25.0 µmol). Finally, triethylsilane (**3**, 669 mg, 918 µL, 1.15 equiv., 5.75 mmol) was added, and the reaction mixture was stirred for 16 hours at room temperature, or until complete conversion of the ester was observed using TLC analysis. After the reaction was complete, it was quenched by the addition of a few drops of acetonitrile. Next, the mixture was filtered through a layer of silica gel, which in turn was washed with hexanes : ethyl acetate 10:1 (V/V). Finally, the reaction mixture was concentrated under reduced pressure using a rotary evaporator to obtain the crude silyl acetal product. This in turn was further purified using flash chromatography on silica gel to obtain 1.41 g (4.35 mmol) of pure acetal **74**, which corresponds to a yield of 87%.

The (*R*)-enantiomer of this acetal (**S27**) was synthesized in a completely analogous reaction, starting from the respective (*R*)-Roche ester.

Compound 74: ((2*S*)-3-(benzyloxy)-1-methoxy-2-methylpropoxy)triethylsilane (See NMR spectra)

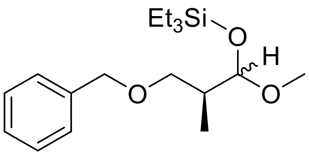

74

Physical state: clear liquid

dr: 56:44

Major diastereomer

^{1}H-NMR (500 MHz, $CDCl_3$) δ = 7.34 – 7.33 (m, 4H), 7.29 – 7.27 (m, 1H), 4.75 (d, J = 4.2 Hz, 1H), 4.54 – 4.45 (m, 2H), 3.50 (dd, J = 9.1, 6.4 Hz, 1H), 3.36 – 3.33 (m, 1H), 3.32 (s, 3H), 2.05 – 1.95 (m, 1H), 0.97 (d, J = 2.6 Hz, 3H), 0.99 – 0.96 (m, 9H), 0.66 (q, J = 8.0 Hz, 6H).

^{13}C-NMR (126 MHz, $CDCl_3$) δ = 138.79, 128.3, 127.5, 127.38, 99.3, 73.06, 72.4, 54.28, 40.4, 10.6, 6.79, 5.0.

Minor diastereomer

^{1}H-NMR (500 MHz, $CDCl_3$) δ = 7.34 – 7.33 (m, 4H), 7.29 – 7.27 (m, 1H), 4.67 (d, J = 4.3 Hz, 1H), 4.54 – 4.45 (m, 2H), 3.56 (dd, J = 9.1, 6.4 Hz, 1H), 3.36 – 3.33 (m, 1H), 3.31 (s, 3H), 2.05 – 1.95 (m, 1H), 1.00 (d, J = 2.4 Hz, 3H), 0.99 – 0.96 (m, 9H), 0.65 (q, J = 7.8 Hz, 6H).

^{13}C-NMR (126 MHz, $CDCl_3$) δ = = 138.75, 128.3, 127.6, 127.40, 100.4, 73.13, 71.7, 54.29, 39.8, 12.1, 6.81, 5.1.

HRMS (ESI-/Q-TOF): Calculated for $[M+Na]^+$ = $[C_{18}H_{32}NaO_3Si]^+$: m/z 347.20129; Found: m/z 347.20080

TLC: Rf: 0.45 using hexanes : ethyl acetate 20:1 (V/V) as an eluent; Visualization: $KMnO_4$

Compound S27: ((2*R*)-3-(benzyloxy)-1-methoxy-2-methylpropoxy)triethylsilane (See NMR spectra)

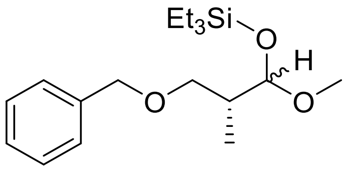

S27

Physical state: clear liquid

dr: 56:44

Major diastereomer

^{1}H-NMR (500 MHz, $CDCl_3$) δ = 7.34 – 7.33 (m, 4H), 7.29 – 7.27 (m, 1H), 4.75 (d, J = 4.2 Hz, 1H), 4.54 – 4.45 (m, 2H), 3.50 (dd, J = 9.1, 6.4 Hz, 1H), 3.36 – 3.33 (m, 1H), 3.32 (s, 3H), 2.05 – 1.95 (m, 1H), 0.97 (d, J = 2.6 Hz, 3H), 0.99 – 0.96 (m, 9H), 0.66 (q, J = 8.0 Hz, 6H).

^{13}C-NMR (126 MHz, $CDCl_3$) δ = 138.79, 128.3, 127.5, 127.38, 99.3, 73.06, 72.4, 54.28, 40.4, 10.6, 6.79, 5.0.

Minor diastereomer

^{1}H-NMR (500 MHz, $CDCl_3$) δ = 7.34 – 7.33 (m, 4H), 7.29 – 7.27 (m, 1H), 4.67 (d, J = 4.3 Hz, 1H), 4.54 – 4.45 (m, 2H), 3.56 (dd, J = 9.1, 6.4 Hz, 1H), 3.36 – 3.33 (m, 1H), 3.31 (s, 3H), 2.05 – 1.95 (m, 1H), 1.00 (d, J = 2.4 Hz, 3H), 0.99 – 0.96 (m, 9H), 0.65 (q, J = 7.8 Hz, 6H).

^{13}C-NMR (126 MHz, $CDCl_3$) δ = 138.75, 128.3, 127.6, 127.40, 100.4, 73.13, 71.7, 54.29, 39.8, 12.1, 6.81, 5.1.

HRMS (ESI-/Q-TOF): Calculated for $[M+Na]^+$ = $[C_{18}H_{32}NaO_3Si]^+$: m/z 347.20129; Found: m/z 347.20080

TLC: Rf: 0.45 using hexanes : ethyl acetate 20:1 (V/V) as an eluent; Visualization: $KMnO_4$

The acetals **74** and **S27** were in turn hydrolyzed to form the respective Roche aldehyde. This was achieved under mild conditions using hexafluorosilicic acid. This reagent, besides acting as an acid to facilitate hydrolysis, can also transform the triethylsilanol by-product into the more volatile triethylfluorosilane[77] which in turn can be removed, together with the solvents, under reduced pressure.

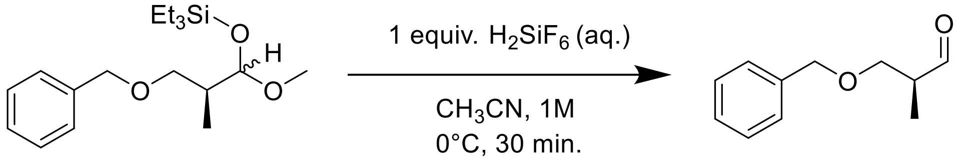

74 **73**: 93%

In a 4 mL oven-dried screw-capped vial equipped with a rubber seal and a magnetic stirrer, 325 mg (1 equiv., 1.00 mmol) of Roche acetal **74** was measured in. The vial was capped, flushed with argon gas, and an argon-filled balloon was connected to it. Then, 1.0 mL acetonitrile (technical grade) was added to the vial and the reaction mixture was cooled to 0°C (using an ice bath). This was followed by the dropwise addition of the hexafluorosilicic acid solution (437 mg, 348 µL, 33 *m*/*m*% in water, 1.0 equiv, 1.00 mmol). Afterwards, the reaction mixture was intensively stirred for a further 30 min. at 0°C. Then, the mixture was left to warm up to room temperature and it was diluted with 25 mL diethyl ether. The organic phase was separated and washed with water (RO purified) and brine. Next, the organic phase was dried on sodium sulfate, filtered, and concentrated under reduced pressure using a rotary evaporator to obtain the crude aldehyde product. This in turn was further purified using flash chromatography on silica gel to obtain 165 mg (926 µmol) of pure aldehyde **73**, which corresponds to a yield of 93%.

The (*R*)-enantiomer of this aldehyde (**S28**) was synthesized in a completely analogous reaction, starting from **S27**. The NMR spectral data of the aldehydes **73** and **S28** is in accordance with literature data.

The enantiomeric excesses (ee) of compounds **73** and **S28** were determined using chiral HPLC analysis. No degradation in enantiopurity was observed, as both compounds had an ee above 99%, highlighting the practicality of this methodology. (Although using the chiral stationary phases available to us, we were not able to determine the enantiomeric excesses of the acetal compounds **74** and **S27** directly, this result corroborates that the hydrosilylation reaction preserved the enantiopurity of the starting materials.) It is also noteworthy that a fast racemization of the aldehyde was often reported in the literature (within an hour, even upon storage at 0°C). Contrary to this observation, the product prepared according to our methodology didn't show any signs of racemization even after days of storage at room temperature. Additionally, the intermediary acetal compounds were stable (did not racemize) for 1 year at -20°C.

Chiral HPLC chromatogram of **73**

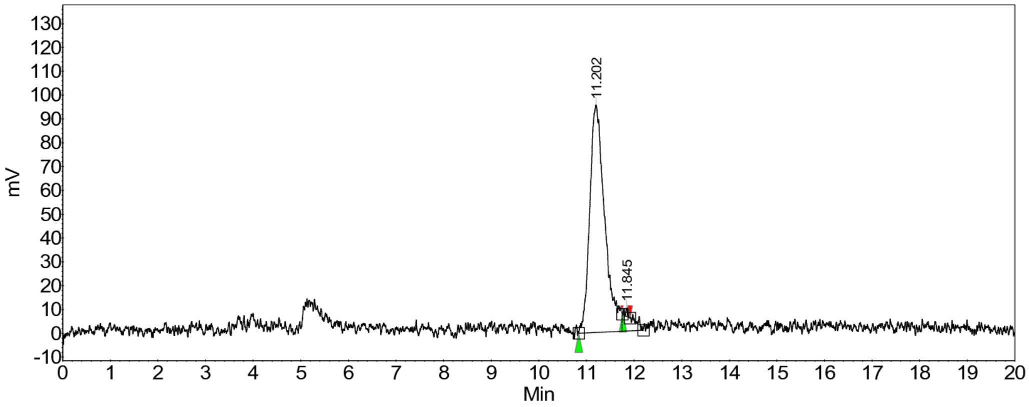

Index	Name	Time [Min]	Quantity [% Area]	Height [mV]	Area [mV.Min]	Area % [%]
1	(S)-Enantiomer	11.202	99.45	95.6	34.7	99.446
2	(R)-Enantiomer	11.845	0.55	3.8	0.2	0.554
Total			100.00	99.4	34.9	100.000

Chiral HPLC chromatogram of **S28**

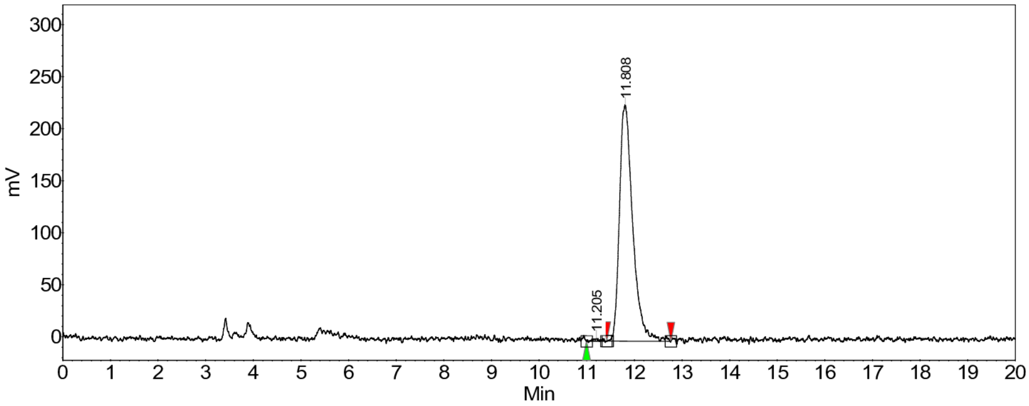

Index	Name	Time [Min]	Quantity [% Area]	Height [mV]	Area [mV.Min]	Area % [%]
2	(S)-Enantiomer	11.205	0.51	2.8	0.4	0.515
1	(R) Enantiomer	11.808	99.49	227.2	72.9	99.485
Total			100.00	229.9	73.3	100.000

Chiral HPLC chromatogram of **73** + **S28** (rac)

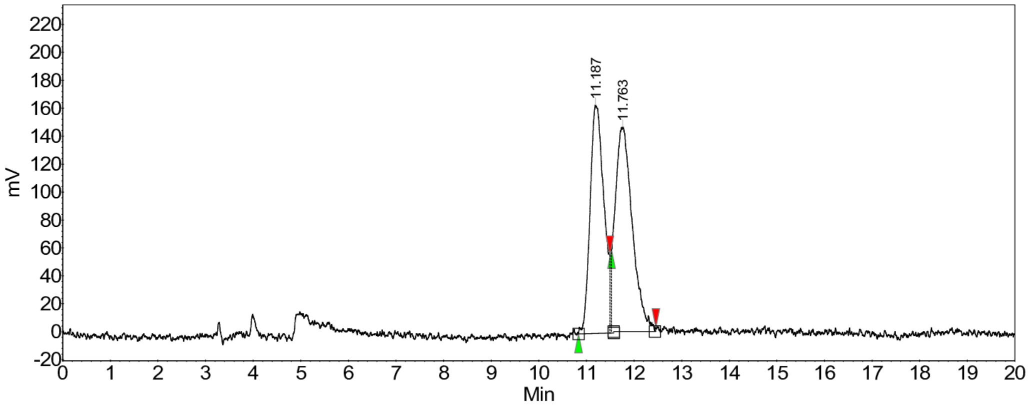

Index	Name	Time [Min]	Quantity [% Area]	Height [mV]	Area [mV.Min]	Area % [%]
1	(S) enantiomer	11.187	47.39	163.7	54.7	47.391
2	(R)-enantiomer	11.763	52.61	146.5	60.7	52.609
Total			100.00	310.2	115.4	100.000

1.5.3 Streamlined synthesis of ALC-0315

A highly efficient and practical synthetic pathway is presented for the synthesis of the cationic lipid ALC-0315.

Step 1: Synthesis of the diester **77**

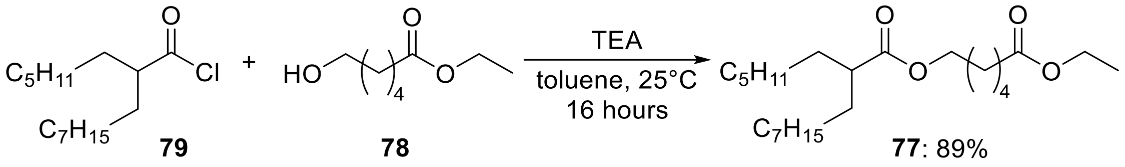

In an oven-dried 2-necked 500 mL flask, equipped with a stirring bar, condenser and addition funnel, under inert conditions (nitrogen gas), ethyl 6-hydroxyhexanoate (**78**, 26.4 g, 26.8 mL, 1 equiv., 165 mmol) was dissolved in 100 mL anhydrous toluene. Next, triethylamine (25.0 g, 34.5 mL, 1.5 equiv., 248 mmol) was added, followed by the dropwise addition of 2-hexyldecanoyl chloride (**79**, 47.8 g, 1.05 equiv., 174 mmol) [Note 1.] dissolved in 60 mL anhydrous toluene. (Precipitation of TEA·HCl began immediately.) The reaction was monitored by TLC and stirring was continued overnight for 16 hours at room temperature.

Next, the reaction mixture was filtered through a Celite layer and washed with additional toluene. The resulting clear solution was washed with 1M aqueous HCl, followed by washing with sat. $NaHCO_3$ (aq) and sat. NaCl (aq). The organic phase was dried on MgSO4, filtered and concentrated under reduced pressure, resulting in 73.2 g of crude product. This ester was purified using column chromatography on silica gel, with gradient elution using hexanes and ethyl acetate, affording 58.5 g 6-ethoxy-6-oxohexyl 2-hexyldecanoate (**77**, 147 mmol, 89 % yield).

[Note 1.] The acyl chloride **79** was freshly prepared by reacting 2-hexyldecanoic acid (44.6 g, 51.0 mL, 1 equiv., 174 mmol) dissolved in 150 mL anhydrous toluene with thionyl chloride (51.7 g, 31.7 mL, 2.5 equiv., 435 mmol) in the presence of a few drops of DMF. After complete conversion is achieved, the solvents, together with the excess thionyl chloride, were removed under reduced pressure to give the acyl chloride, which was immediately used up in the next reaction.

Compound 77: 6-ethoxy-6-oxohexyl 2-hexyldecanoate (See NMR spectra)

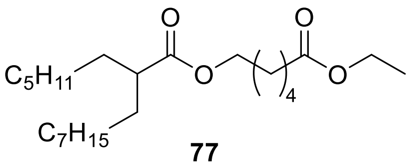

77

Physical state: yellowish oil

^{1}H-NMR (500 MHz, $CDCl_3$) δ = 4.12 (q, J = 7.2 Hz, 2H), 4.06 (t, J = 6.6 Hz, 2H), 2.33 – 2.26 (m, 3H), 1.64 (dt, J = 14.8, 7.5 Hz, 4H), 1.57 (dd, J = 8.6, 5.6 Hz, 2H), 1.46 – 1.35 (m, 4H), 1.30 – 1.18 (m, 23H), 0.90 – 0.83 (m, 6H).

^{13}C-NMR (126 MHz, $CDCl_3$) δ = 176.6, 173.4, 63.8, 60.2, 45.8, 34.2, 32.5 (2C), 31.8, 31.7, 29.5, 29.4, 29.22, 29.19, 28.4, 27.44, 27.40, 25.6, 24.6, 22.62, 22.55, 14.2, 14.04, 14.00.

HRMS (ESI-/Q-TOF): Calculated for $[M+Na]^+$ = $[C_{24}H_{46}NaO_4]^+$: m/z 421.32883; Found: m/z 421.33046

TLC: Rf: 0.34 using hexanes : ethyl acetate 20:1 (V/V) as an eluent; Visualization: $KMnO_4$

Step 2: Synthesis of acetal **80**

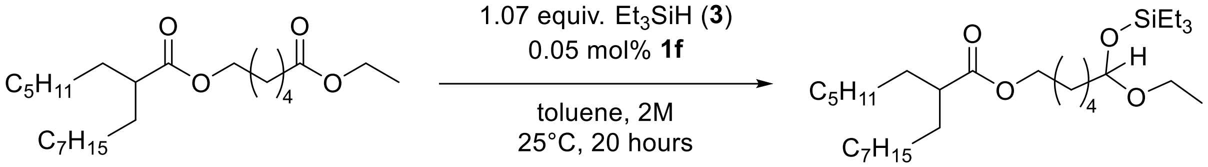

77 **80**: 98%

In an oven-dried 100 mL flask equipped with a stirring bar, rubber septa, thermometer and nitrogen inlet, under inert conditions (nitrogen gas), 6-ethoxy-6-oxohexyl 2-hexyldecanoate (**77**, 31.9 g, 1 equiv., 80.0 mmol) was dissolved in toluene (40 mL, anhydrous). Next, a stock solution of the **1f** catalyst (17.9 mg, 800 µL, 0.05 M in anhydrous toluene, 0.0005 equiv., 40.0 µmol) was added. Finally, triethylsilane (**3**, 9.95 g, 13.7 mL, 1.07 equiv., 85.6 mmol) was added dropwise, keeping the temperature of the reaction between 24°C and 28°C. The reaction was further stirred at room temperature overnight for a total of 20 hours. Monitoring of the reaction is done by TLC and NMR analysis.

This reaction mixture can be used in the next synthetic step as it is. Alternatively, work-up of the reaction is continued according to the General procedure D. After the reaction was complete, it was quenched by the addition of a few drops of acetonitrile. Next, the mixture was filtered through a layer of silica gel, which in turn was washed with hexanes : ethyl acetate 10:1 (V/V). Finally, the reaction mixture was concentrated under reduced pressure using a rotary evaporator to obtain 40.5 g (78.7 mmol) of silyl acetal **80**, which corresponds to a yield of 98%. The crude acetal is pure enough for the next synthetic step.

Compound 80: 6-ethoxy-6-((triethylsilyl)oxy)hexyl 2-hexyldecanoate (See NMR spectra)

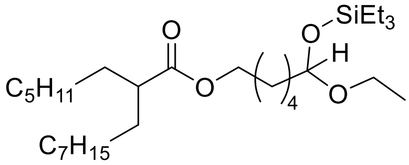

80

Physical state: pale yellow oil

^{1}H-NMR (500 MHz, $CDCl_3$) δ = 4.75 (dd, J = 6.1, 4.3 Hz, 1H), 4.06 (t, J = 6.7 Hz, 2H), 3.73 – 3.65 (m, 1H), 3.40 (dq, J = 9.2, 7.0 Hz, 1H), 2.30 (tt, J = 8.9, 5.3 Hz, 1H), 1.66 – 1.49 (m, 6H), 1.47 – 1.33 (m, 6H), 1.31 – 1.20 (m, 20H), 1.18 (t, J = 7.1 Hz, 3H), 0.97 (t, J = 7.9 Hz, 9H), 0.87 (td, J = 7.0, 1.3 Hz, 6H), 0.63 (q, J = 7.9 Hz, 6H).

^{13}C-NMR (126 MHz, $CDCl_3$) δ = 176.6, 98.0, 64.0, 61.7, 45.8, 37.6, 32.5 (2C), 31.8, 31.7, 29.6, 29.4, 29.24, 29.21, 28.8, 27.45, 27.41, 25.9, 24.1, 22.64, 22.57, 15.2, 14.1, 14.0, 6.8, 5.1.

HRMS (ESI-/Q-TOF): Calculated for $[M+Na]^+$ = $[C_{30}H_{62}NaO_4Si]^+$: m/z 537.43096; Found: m/z 537.43356

TLC: Rf: 0.45 using hexanes : ethyl acetate 20:1 (V/V) as an eluent; Visualization: $KMnO_4$

Step 3: Synthesis of aldehyde **76**

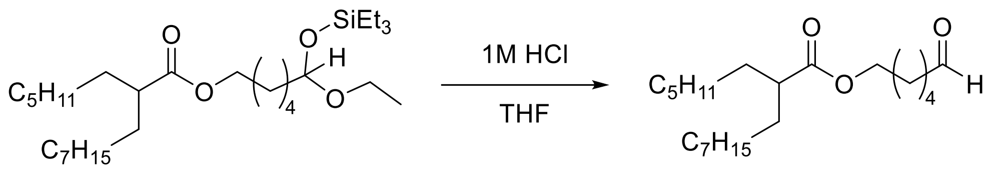

80 **76**: 99+%

In a 1000 mL Erlenmeyer flask, equipped with a stirring bar, 30.9 g of the acetal **80** (1 equiv., 60.0 mmol) was dissolved in tetrahydrofuran (600 mL, technical grade). [Note 1.] Next, an aqueous solution of hydrochloric acid (10.9 g, 300 mL, 1 M, 5 equiv., 300 mmol) was slowly added. The resulting biphasic system was stirred intensively for 3 hours at room temperature. The reaction was monitored by TLC.

Afterwards, sat. NaCl (aq) was added, and the phases were separated. The aqueous phase was further extracted with 2x100 mL toluene. The organic phases were combined and washed with sat. $NaHCO_3$ (aq) and sat. NaCl (aq). Next, solvents were removed under reduced pressure on a rotary evaporator. The resulting crude product was mixed with approx. 1.5 L of distilled water, which was once again removed under reduced pressure on a rotary evaporator (with a 50°C bath temperature). [Note 2.]

Next, the oily residue left behind was dissolved in *n*-heptane, washed with sat. NaCl (aq), dried on $MgSO_4$, filtered, and concentrated under reduced pressure, followed by dissolution in anhydrous ethanol and once again concentration under reduced pressure to remove any residual hydrocarbonic solvents. This afforded 21.2 g (59.8 mmol) of the aldehyde **76**, corresponding to a yield of 99+%. The isolated aldehyde was used in the next reaction step without the need of further purification.

[Note 1.] Alternatively, the final reaction mixture of step 2 can be used directly, without the need of isolating the acetal.

[Note 2.] This is basically a steam distilling step, which removes the silanol and siloxane by-products.

Compound 76: 6-oxohexyl 2-hexyldecanoate (See NMR spectra)

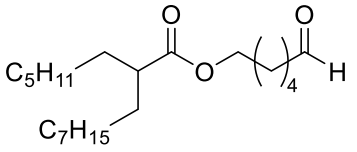

76

Physical state: clear oily liquid

^{1}H-NMR (500 MHz, $CDCl_3$) δ = 9.77 (t, J = 1.7 Hz, 1H), 4.07 (t, J = 6.6 Hz, 2H), 2.44 (td, J = 7.3, 1.7 Hz, 2H), 2.31 (tt, J = 8.8, 5.3 Hz, 1H), 1.71 – 1.62 (m, 4H), 1.59 (q, J = 8.0 Hz, 2H), 1.47 – 1.37 (m, 4H), 1.31 – 1.22 (m, 20H), 0.90 – 0.86 (m, 6H).

^{13}C-NMR (126 MHz, $CDCl_3$) δ = 202.1, 176.6, 63.6, 45.8, 43.7, 32.5 (2C), 31.8, 31.7, 29.5, 29.4, 29.20, 29.17, 28.5, 27.43, 27.39, 25.6, 22.6, 22.5, 21.6, 14.03, 13.99.

TLC: Rf: 0.21 using hexanes : ethyl acetate 20:1 (V/V) as an eluent; Visualization: $KMnO_4$

Step 4: Synthesis of ALC-0315 (**75**)

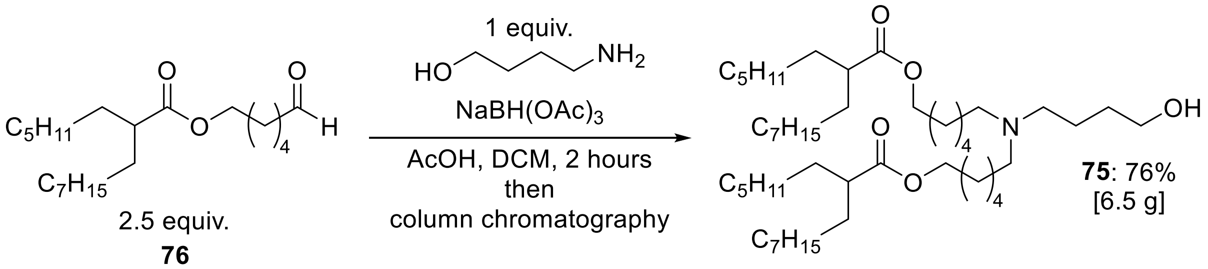

In an oven-dried 100 mL flask, equipped with a stirring bar, rubber septa, immersion probe digital thermometer and nitrogen inlet, under inert conditions (nitrogen gas), powdered $NaBH_4$ (1.06 g, 2.5 equiv., 28.0 mmol) was suspended in anhydrous toluene (40 mL). Next, acetic acid (5.05 g, 4.81 mL, 7.5 equiv., 84.1 mmol) was added dropwise, keeping the reaction temperature below 30°C. The resulting suspension was stirred for 24 hours (in situ formation of triacetoxyborohydride) [Note 1.].

Next, 4-aminobutan-1-ol (1.00 g, 1.03 mL, 1 equiv., 11.2 mmol) was added, followed by the addition of acetic acid (1.68 g, 1.60 mL, 2.5 equiv., 28.0 mmol). Finally, 6-oxohexyl 2-hexyldecanoate (**76**, 9.94 g, 2.5 equiv., 28.0 mmol), dissolved in 10 mL anhydrous toluene, was added dropwise, keeping the reaction temperature below 30°C. The resulting suspension was stirred for 8 hours. Monitoring of the reaction is done using HPLC-MS.

The reaction was quenched by the addition of distilled water. Next, the reaction mixture was diluted with an additional 100 mL toluene and the phases were separated. The organic phase was washed with sat. NaHCO3 (aq) and sat. NaCl (aq), dried on MgSO4, filtered, and concentrated under reduced pressure using a rotary evaporator. The resulting crude mixture was purified by column chromatography on silica gel, using hexanes and 2-propanol as eluents (with gradient elution). For better chromatographic performance, 1 mL of acetic acid was added to the crude mixture, after which it was dissolved in hexanes and the resulting solution was evaporated onto the surface of silica gel. The obtained silica gel containing the crude product was placed on top of the chromatographic column (in a separate sample loading cartridge) and chromatography was carried out as usual. [Note 2.] After chromatography, the fractions containing the product were concentrated in vacuo, the resulting oil was dissolved in *n*-heptane, washed with sat. $NaHCO_3$ (aq) and sat. NaCl (aq), dried on $MgSO_4$, filtered, and concentrated under reduced pressure using a rotary evaporator. This was followed by dissolution in

anhydrous ethanol and once again concentration under reduced pressure to remove any residual hydrocarbonic solvents. Finally, this yielded 6.54 g of ALC-0315 (**75**, 8.54 mmol), which corresponds to a yield of 76 %. The purity of the final product is 98% according to ^{1}H q-NMR measurements and 99% according to HPLC-MS.

[Note 1.] This step is particularly important to ensure a finely dispersed triacetoxyborohydride suspension, which is crucial for the selectivity and efficiency of the reaction. The use of commercially available triacetoxyborohydride resulted in lower yields and selectivity.
[Note 2.] Using this technique, the cationic lipid probably forms an acetic acid salt which greatly improves the efficiency of the chromatography, as significantly better separation is observed. On the other hand, as the acetic acid coelutes with the lipid, it must be removed via a basic extraction step after chromatography.

Compound 75: ALC-0315 (See NMR spectra)

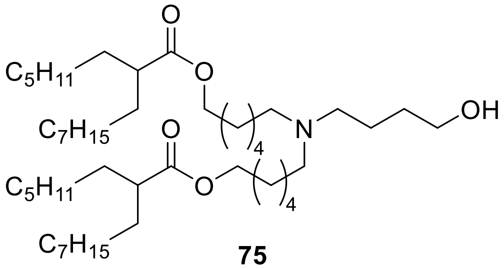

75

Physical state: clear oil

^{1}H-NMR (500 MHz, $CDCl_3$) δ = 6.23 (br s, 1H), 4.06 (t, J = 6.7 Hz, 4H), 3.55 (t, J = 4.8 Hz, 2H), 2.47 – 2.38 (m, 6H), 2.31 (tt, J = 8.8, 5.3 Hz, 2H), 1.67 – 1.55 (m, 13H), 1.53 – 1.33 (m, 12H), 1.34 – 1.20 (m, 43H), 0.88 (td, J = 7.0, 1.1 Hz, 12H).

^{13}C-NMR (126 MHz, $CDCl_3$) δ = 176.6 (2C), 64.0(2C), 62.7, 54.7(2C), 53.7, 45.8(2C), 32.7, 32.5(4C), 31.8(2C), 31.7(2C), 29.5(2C), 29.4(2C), 29.23(2C), 29.20(2C), 28.7(2C), 27.44(2C), 27.40(2C), 27.3(2C), 26.4, 26.0(2C), 25.9(2C), 22.64(2C), 22.56(2C), 14.1(2C), 14.0(2C).

TLC: Rf: 0.29 using hexanes : 2-propanol 10:1 (V/V) as an eluent; Visualization: $KMnO_4$

1.5.4 Industrial-scale pheromone synthesis, Pheron®RSB

To highlight the practicality and industrial relevance of the developed methodology, an industrial-scale synthesis is presented for the straight chain lepidopteran pheromone (*Z*)-hexadec-11-en-1-al (**81**), a key component of Pheron®RSB .

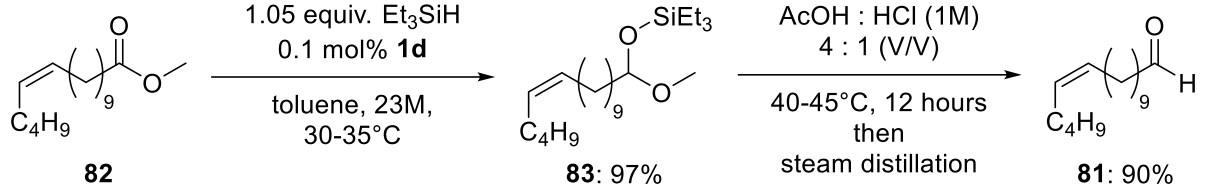

Multiple batches of reductions and hydrolyses were performed to achieve up to 930 kg of aldehyde **81**. The largest scale reduction reaction was performed using 504 kg of unsaturated fatty acid methyl ester **82** (produced via fermentation, for more details see: WO/2017/087846).

Reduction of methyl (*Z*)-hexadec-11-enoate (**82**, Z11-16ME): Borane catalyst **1d** (907 g, 0.1 mol%, 1.88 mol) was dissolved in anhydrous toluene (83 L) and added into a jacketed reactor, equipped with thermometer, dropping funnel, and bubbler. To this solution was slowly added a mixture of methyl (*Z*)-hexadec-11-enoate (**82**, 504 kg, 1 equiv. 1.88 kmol) and triethylsilane (**3**, 229 kg, 315 L, 1.05 equiv., 1.97 kmol) over a period and the temperature was maintained between 30°C and 35°C. Gas formation was limited to a manageable extent. After completion of the addition, the reaction was stirred at room temperature and monitored by TLC and GC-FID (stop criteria: <1.5 area% **82**). The yield of the reduction step was 97%, while the quantity of overreduced product was <1.5 area% based on GC-MS analysis. The reaction was quenched with 18.3 L ethyl acetate and stirred for an additional 45 min.

Hydrolysis: To part of the crude silyl acetal solution (484 kg of solution containing 1.09 kmol of silyl acetal **83**) was added a mixture of 300 L acetic acid and 75 L of 1M HCl. The mixture was stirred at 40-45°C for 12 hours. The acidic aqueous phase was drained and the organic phase was washed with water. After separating the phases, the residual solvents were removed from the crude aldehyde via azeotropic drying using methyl *t*-butyl ether. The product mixture was purified via counter current steam distillation to yield 234 kg (981 mol) of aldehyde **81** (90% yield). The purity of the final product was evaluated using GC-MS (Fig. S7) and NMR (Fig. S8).

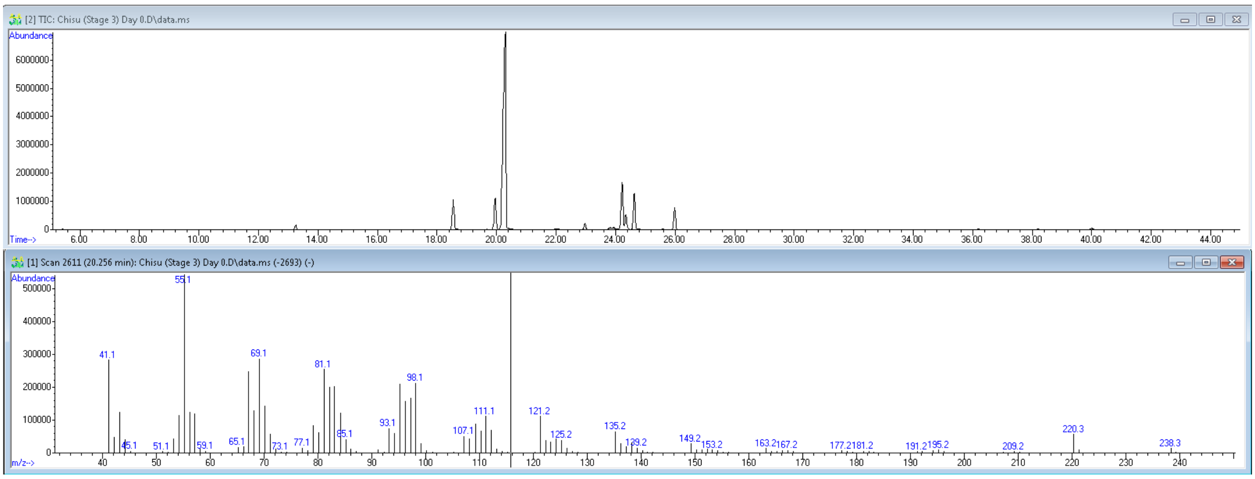

Fig. S7. GC-MS analysis of the final product

Representative gas chromatogram (top) and GCMS (bottom) of (Z)-hexadec-11-en-1-al (**81**, chemical formula: C16H30O; m/z: 238.3).

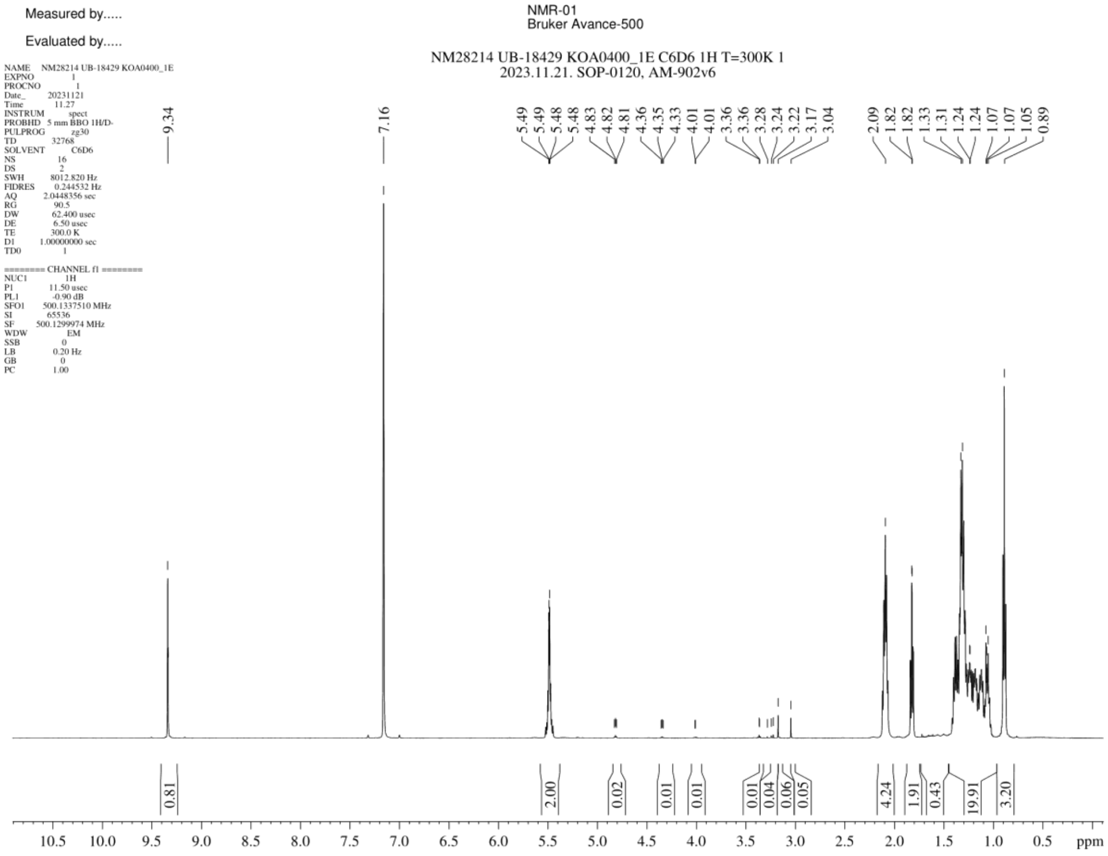

Fig. S8. ^{1}H-NMR analysis of the final product 81

1.5.5 Notes on the hydrolysis and application of the formed mixed acetals

The mixed acetals formed in the selective hydrosilylation of esters act as stable/protected precursors to aldehydes. This enables higher isolated yields and better stability during long-term storage.

When the aldehyde function is needed, the formed acetals can be easily hydrolyzed to afford aldehydes (even in a one pot manner after the reductive step) usually via conventional methods. The acidic hydrolysis of the acetal functional group is pretty well known in organic synthesis and mixed silyl acetals were also readily hydrolyzed under acetic conditions in the presented prior arts.[26,30] In these works, most of the functional groups also present in our scope were tolerated during acidic workup procedures (i.e., double bond, triple bond, halogen, amine, nitro, hydroxide, ether, heteroaromatic). Even more so, the presence of the silyl group in these acetals also enables hydrolysis via the use of traditional desilylation techniques, like the use of fluorine containing reagents. Indeed, these acetals are readily hydrolyzed by TBAF, TEA-HF or Pyridine-HF. To highlight some of these possibilities, we presented several hydrolysis methods in the application section, like the traditional use of HCl or acetic acid (Compounds **71**, **76**, **81**), or a novel, milder method that uses hexafluorosilicic acid, which also helps with the removal of the silicon containing by-product via the formation of volatile triethylfluorosilane (Compound **73**).

In the case of substrates containing basic functionalities, the use of an acidic work-up might not be ideal. Herein we demonstrate on the example of the pyridine containing compound **49** that the mixed acetal functional group can be hydrolyzed easily using a scalable and affordable novel technique that employs fluorspar.[78]

Synthesis of aldehyde **S29**

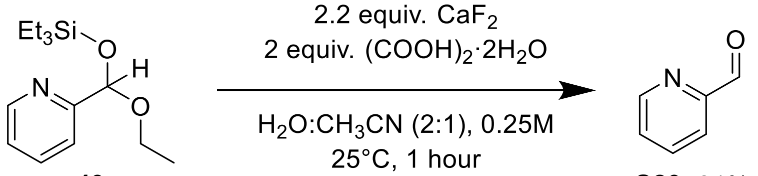

49 **S29**: 81%

In a 10 mL PP centrifuge (Falcon) tube equipped with a magnetic stirrer, calcium fluoride (64 mg, 2.2 equiv., 0.82 mmol) and oxalic acid dihydrate (94 mg, 2 equiv., 0.75 mmol) were measured in and suspended in 1 mL H_2O. Next, while stirring, a solution of acetal **49** (100 mg, 1 equiv., 374 μmol) in 0.5 mL CH_3CN was added dropwise. The reaction vessel was flushed with Ar gas and closed. Stirring was continued for 1 hour at room temperature (25°C), after which TLC analysis showed the complete conversion of the acetal. Next, saturated $NaHCO_{3(aq)}$ was added to the reaction mixture until pH=10 was reached. Then the mixture was extracted with 3x15 mL DCM. The combined organic phase was washed with 20 mL sat. $NaHCO_{3(aq)}$, dried on $MgSO_4$, filtered and concentrated under reduced pressure, resulting in 102 mg of crude product. This was subsequently purified using column chromatography on silica gel, with gradient elution using hexanes and ethyl acetate, affording 65 mg of picolinaldehyde (**S29**, 748 μmol, 81% yield) as a clear liquid.

Compound S29: picolinaldehyde

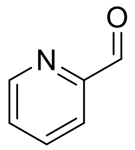

S29

The NMR spectral data of the final product is in accordance with literature data[79].

Physical state: clear liquid

^{1}H-NMR (500 MHz, $CDCl_3$) δ = 10.09 (s, 1H), 8.80 (dd, J = 4.9, 1.5 Hz, 1H), 7.97 (dd, J = 7.8, 1.2 Hz, 1H), 7.88 (td, J = 7.7, 1.6 Hz, 1H), 7.55 – 7.50 (m, 1H).

^{13}C-NMR (126 MHz, $CDCl_3$) δ = 193.4, 152.9, 150.3, 137.3, 128.0, 121.9.

Another noteworthy example is the hydrolysis of the bifunctional substrate **52**, which might also pose a challenge. In this case, the special reactivity of the triethylsilyl carbamate group[80] can be exploited to provide orthogonal functionalization opportunities. Herein we present that the free amine-acetal (**S30**) can be obtained through a process of alcoholic solvolysis. Alternatively, the use of acetic anhydride enables the synthesis of a protected amino-acetal compound (**S31**), which can then be subjected to hydrolysis of the acetal group under mild conditions. In this manner, these substrates can serve as valuable precursors for divergent synthesis.

Synthesis of amino-acetal **S30**

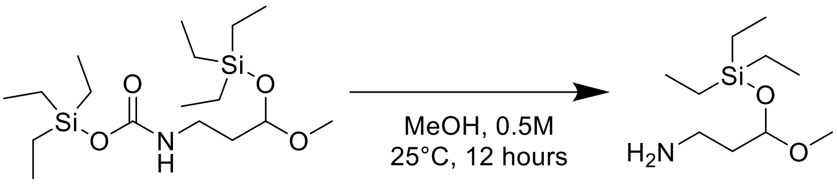

52 **S30**: 63%

In a 4 mL oven-dried screw-capped vial equipped with a rubber seal and a magnetic stirrer, 944 mg (1 equiv., 2.50 mmol) of acetal **52** was measured in. (The reaction can also be conducted in a one-pot manner using the crude reaction mixture from the synthesis of compound **52** directly.) The vial was capped, flushed with argon gas, and an argon-filled balloon was connected to it. Afterwards, 5 mL technical grade MeOH (0.5M) was added to the vial, and the reaction mixture was stirred for 12 hours at room temperature, or until complete conversion of the starting material was observed using TLC analysis. After the reaction was complete, the solvent was removed under reduced pressure using a rotary evaporator to obtain the crude amino-acetal product. This was subsequently purified using column chromatography on silica gel, with gradient elution using methanol and dichloromethane, affording 345 mg of the amino-acetal (**S30**, 1.57 mmol, 63% yield) as a clear liquid.

Compound S30: 3-methoxy-3-((triethylsilyl)oxy)propan-1-amine (See NMR spectra)

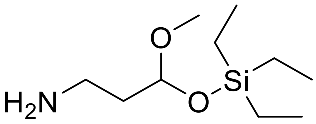

S30

Physical state: clear liquid

^{1}H-NMR (500 MHz, $CDCl_3$) δ = 4.76 (t, J = 5.0 Hz, 1H), 3.28 (s, 3H), 2.85 – 2.71 (m, 4H), 1.72 (td, J = 6.8, 5.0 Hz, 2H), 0.93 (t, J = 8.0 Hz, 9H), 0.60 (q, J = 7.9 Hz, 6H).

^{13}C-NMR (126 MHz, $CDCl_3$) δ = 97.9, 53.7, 39.7, 37.2, 6.6, 4.9.

HRMS (ESI-/Q-TOF): Calculated for $[M+Na]^+$ = $[C_{10}H_{25}NNaO_2Si]^+$: m/z 242.1547; Found: m/z 242.1546

Synthesis of amino-acetal **S31**

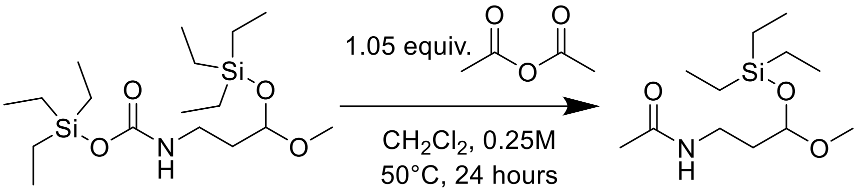

52 **S31**: 56%

In a 4 mL oven-dried screw-capped vial equipped with a rubber seal and a magnetic stirrer, 254 mg (1 equiv., 1.25 mmol) of acetal **52** was measured in. (The reaction can also be conducted in a one-pot manner using the crude reaction mixture from the synthesis of compound **52** directly.) The vial was capped, flushed with argon gas, and an argon-filled balloon was connected to it. Afterwards, 5 mL technical grade CH_2Cl_2 (0.25M) was added to the vial, followed by the addition of 124 µL acetic anhydride (134 mg, 1.05 equiv., 1.31 mmol). The reaction mixture was heated to 50°C and stirring was continued until 24 or until complete conversion of the starting material was observed using TLC analysis. After the reaction was complete, the solvents were removed under reduced pressure using a rotary evaporator to obtain the crude amino-acetal product. This was subsequently purified using column chromatography on silica gel, with gradient elution using hexanes and ethyl acetate, affording 183 mg of the amino-acetal (**S31**, 700 µmol, 56% yield) as a clear liquid.

Compound S31: *N*-(3-methoxy-3-((triethylsilyl)oxy)propyl)acetamide (See NMR spectra)

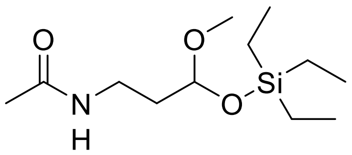

S31

Physical state: clear liquid

^{1}H-NMR (500 MHz, $CDCl_3$) δ = 6.16 (br s, 1H), 4.79 (dd, J = 5.4, 3.7 Hz, 1H), 3.48 – 3.24 (m, 5H), 1.93 (s, 3H), 1.77 (dtdd, J = 16.8, 14.3, 8.3, 5.7 Hz, 2H), 0.97 (t, J = 7.9 Hz, 9H), 0.64 (q, J = 8.0 Hz, 6H).

^{13}C-NMR (126 MHz, $CDCl_3$) δ = 169.7, 98.5, 54.2, 36.0, 35.0, 23.3, 6.7, 4.9.

HRMS (ESI-/Q-TOF): Calculated for $[M+Na]^+$ = $[C_{12}H_{27}NNaO_3Si]^+$: m/z 284.1652; Found: m/z 284.1650

2. Mechanistic and Theoretical Investigations

2.1 Computational methods

Critical points have been optimized at the B97-3c level[81] including toluene CPCM solvent model[82], using the Orca 5.0.3[83] program package. Local minima and saddle points of the applied PES have been identified by all positive or a single negative Hessian eigenvalue respectively using the same level of theory. Thermal and entropic corrections were evaluated using the ideal gas-rigid rotor-harmonic oscillator (RRHO) approximations at 298.15 K and c = 1 mol/dm^3 concentration. All elementary reactions were confirmed by IRC calculations. The final high-accuracy single-point energies have been calculated using the range-separated double-hybrid ωB97X-2 functional[84] on the def2-QZVPP basis set[85]. To reduce the computational cost of the computations, while maintaining the required accuracy, we have employed the Domain-Based Local Pair Natural Orbital (DLPNO) treatment with tightPNO settings for all double hybrid calculations.[86]

To assess the conformational flexibility of key intermediates and key transition states, we have utilized the Crest 2.11.2 program[87] with the GFN2-xTB semiempirical method for conformational analysis. Initial structures have been provided from previous DFT-optimized geometries. In the case of transition states, restraints have been applied for atoms in the reaction center. Then, the resulting conformer ensembles were refined using the Censo 1.1.2 energetic sorting program[87] in combination with Orca 5.0.3 for quantum chemical computations. In the censo protocol, we have used GFN2-xTB for initial prescreening, B97 3c for geometry optimization (in the case of transition states we applied constrained optimizations), and the meta-GGA B97M-V functional[88] on the def2-TZVPP basis set[85] for single-point energies. Based on the meta-GGA energetic ranking, the lowest energy conformer has been selected and calculated on the high-accuracy level (DLPNO-ωB97X-2/def2-QZVPP // B97-3c CPCM(toluene)).

High-accuracy electronic energies have been provided via Local Natural Orbital Coupled-Cluster calculations (LNO-CCSD(T))[66-68] using the MRCC program package.[89,90] To approach the CBS limit, def2-TZVPP/def2-QZVPP energy extrapolation has been done according to Neese et al.[91] Throughout the calculations, the local correlation threshold was set to normal.

Solvent effects have been accounted for using the ωB97M-V functional[92] on the def2-TZVPP basis set including the toluene CPCM solvent model. Solvation energies have been calculated using the difference of gas phase and solvation-included single-point energies.

Noncovalent interaction analysis has been performed by the Nciplot program[72] using the ωB97M-V/def2-TZVPP electron densities.

2.2 Results on the double-hybrid level

2.2.1 Borane complexes

Comparing the two catalysts, the formation of a **1a**-ester complex is exergonic by -3.7 kcal/mol, while the **1d**-ester is less exergonic to form (-1.2 kcal/mol), these are the resting states in the mechanism of catalytic hydrosilylation. Notably, the boron center shows sp^3 character ($O_{carbonyl}$-B-C angle 101-111°) in these complexes, and one of the aryl rings is in an axial position (Fig. S9).

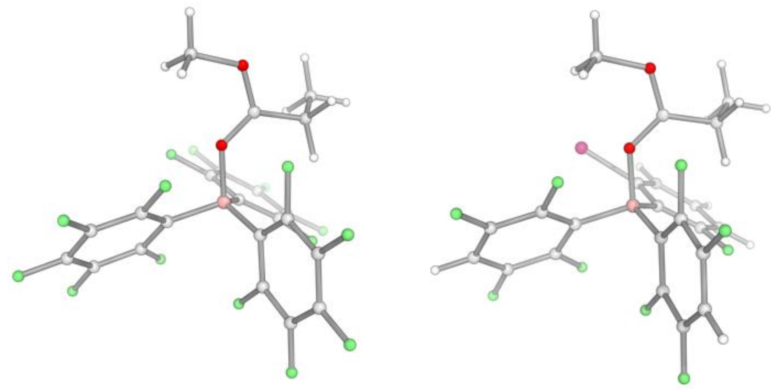

	A1'	A1
Δ*G* of formation	-3.7 kcal/mol	-1.2 kcal/mol

Fig. S9. Comparison of borane-ester adducts, calculated on the DLPNO-ωB97X-2/def2-QZVPP // B97-3c level

Complexation with triethylsilane (**3**) is endergonic with both catalysts, but again, there is a more favorable association in the case of **1a**. From a structural point of view, all rings are arranged in a symmetrical propellane-like structure (Fig. S10), and the boron center suffers only a weak pyramidalization (H_{silane}-B-C angle 98-100°).

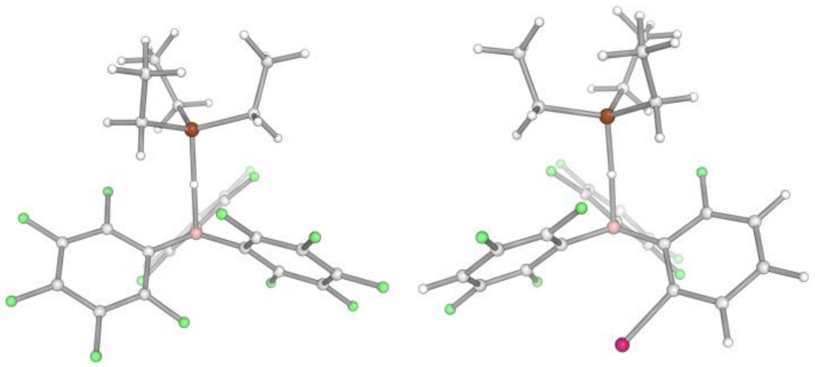

	A2'	A2
Δ*G* of formation	4.1 kcal/mol	6.3 kcal/mol

Fig. S10. Comparison of borane-silane adducts, calculated on the DLPNO-ωB97X-2/def2-QZVPP // B97-3c level

In the calculation of free energy profiles of different mechanisms, the borane-ester adducts were used as a reference point, except when modeling a full-conversion scenario.

2.2.2 Mechanism of the reduction

First, we aimed to validate the Piers–Oestreich-type mechanism of hydrosilylation using **1a** as the catalyst (Fig. S11, path C). As in principle, the borane could also activate the carbonyl, two alternative routes have been considered (Fig. S11, path A and B), or it could activate the Si–H bond (Fig. S11, path C).

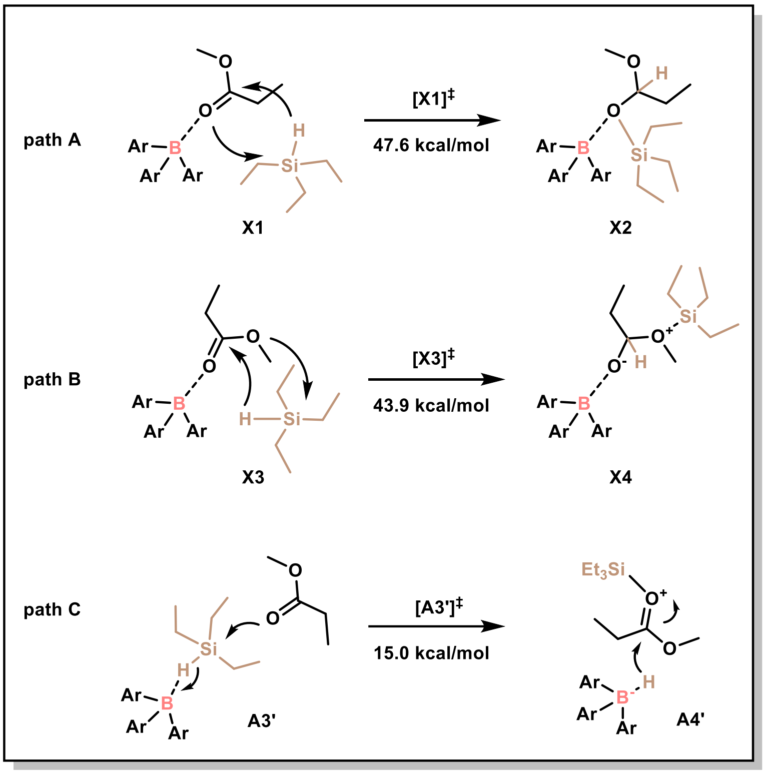

Fig. S11. Possible mechanistic routes of reduction, calculated on the DLPNO-ωB97X-2/def2-QZVPP // B97-3c level

Carbonyl activation can take place in two ways: the silylium cation is either capped by the carbonyl oxygen (path A) or by the methoxy group (path B). In both routes, prohibitively high activation energy has been found (47.6 and 43.9 kcal/mol, respectively). The activation of Si–H, however, is much more favorable (Fig. S12): after the association of the ternary complex A3' the Si–H scission requires a small activation energy (15.0 kcal/mol) via [A3']‡. The resulting ion pair (A4', 4.4 kcal) can undergo a hydride transfer reaction having a barrier of 13.4 kcal/mol via [A4']‡ to give the silyl-acetal product and the free borane (-6.4 kcal).

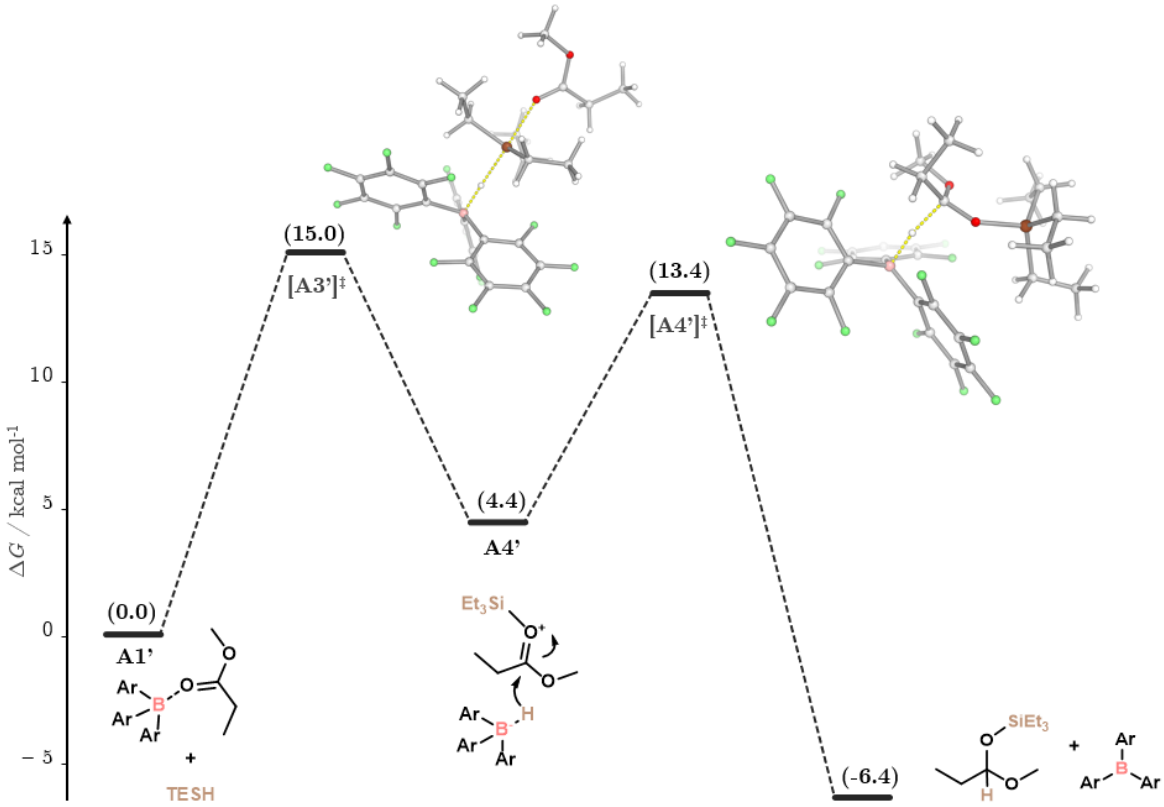

Fig. S12. Free energy diagram of reduction, calculated on the DLPNO-ωB97X-2/def2-QZVPP // B97-3c level

Therefore, our results are in good accordance with the experimental mechanistic investigations of Piers[26], and Oestreich[28] and the computational studies of Sakata[69] for similar hydrosilylation reactions, concluding that the borane activates the silane and the attack of carbonyl results in Si–H bond cleavage.

2.2.3 Mechanism of the overreduction

Theoretically, there could be several routes leading to the over-reduced silyl-ether side products. Here, we have rationalized three alternative mechanisms (Fig. S13) below and compared them using **1a** as the model catalyst to establish the most probable one. Similar to the first hydride reduction, the Piers–Oestreich-type Si–H cleavage can take place (Fig. S14). In this reaction, silane activation requires a higher activation energy of 19.4 kcal/mol (via [C3']‡). Further steps from X6 are the same as in path C and will be discussed later.

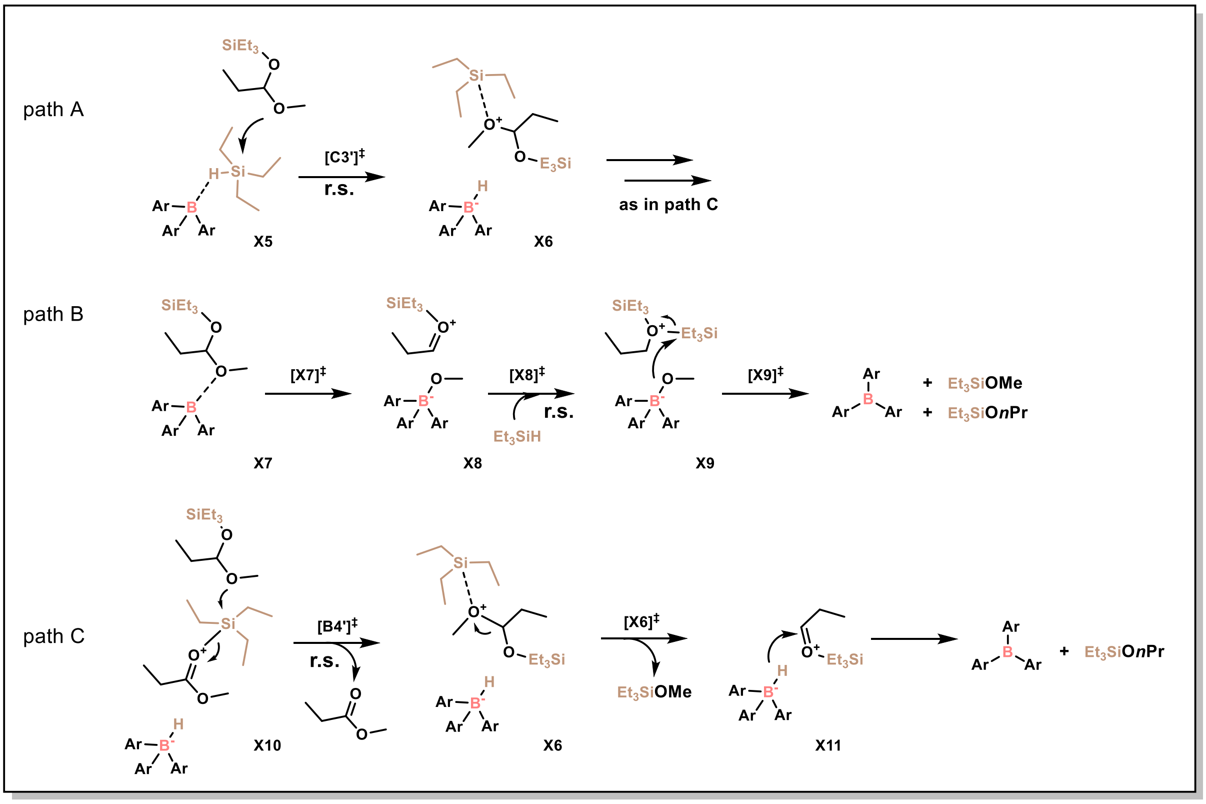

Fig. S13. Possible mechanistic routes of overreduction

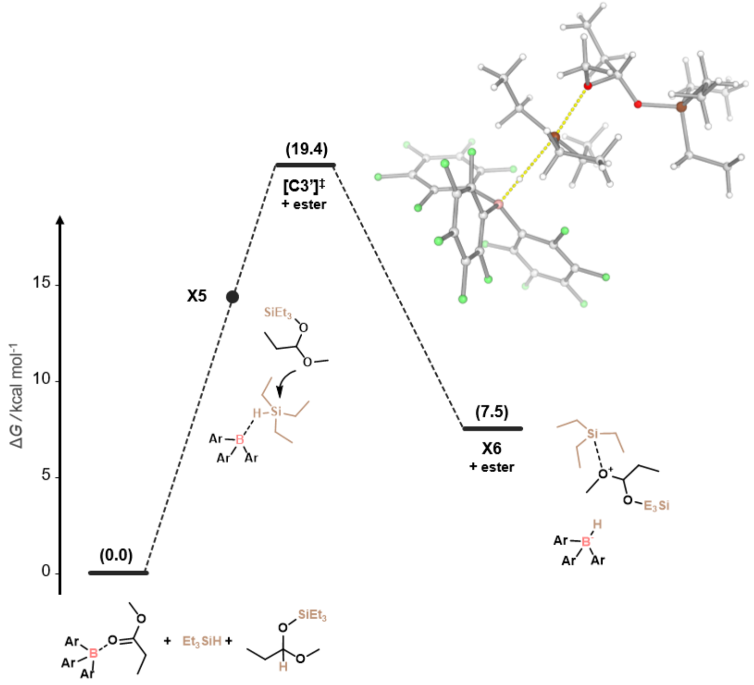

Fig. S14. Free energy diagram of overreduction, path A, calculated on the DLPNO-ωB97X-2/def2-QZVPP // B97-3c level

Next, we also considered a mechanistic route, where **1a** activates the methoxy group of the silyl-acetal in path B (Fig. S13). First, borane activation leads to methoxy cleavage on the silyl acetal via [X7]‡ (14.8 kcal/mol) in an S_N1-type step (Fig. S15). This leads to a highly reactive carbocation X8, which can react with a second triethylsilane molecule after association,

however, this step requires an activation energy of 25.1 kcal/mol (via [X8]‡). This is followed by the association of methoxy and a silylium cation to give methyl-silyl and propyl-silyl ether via [X9]‡ (5.8 kcal/mol).

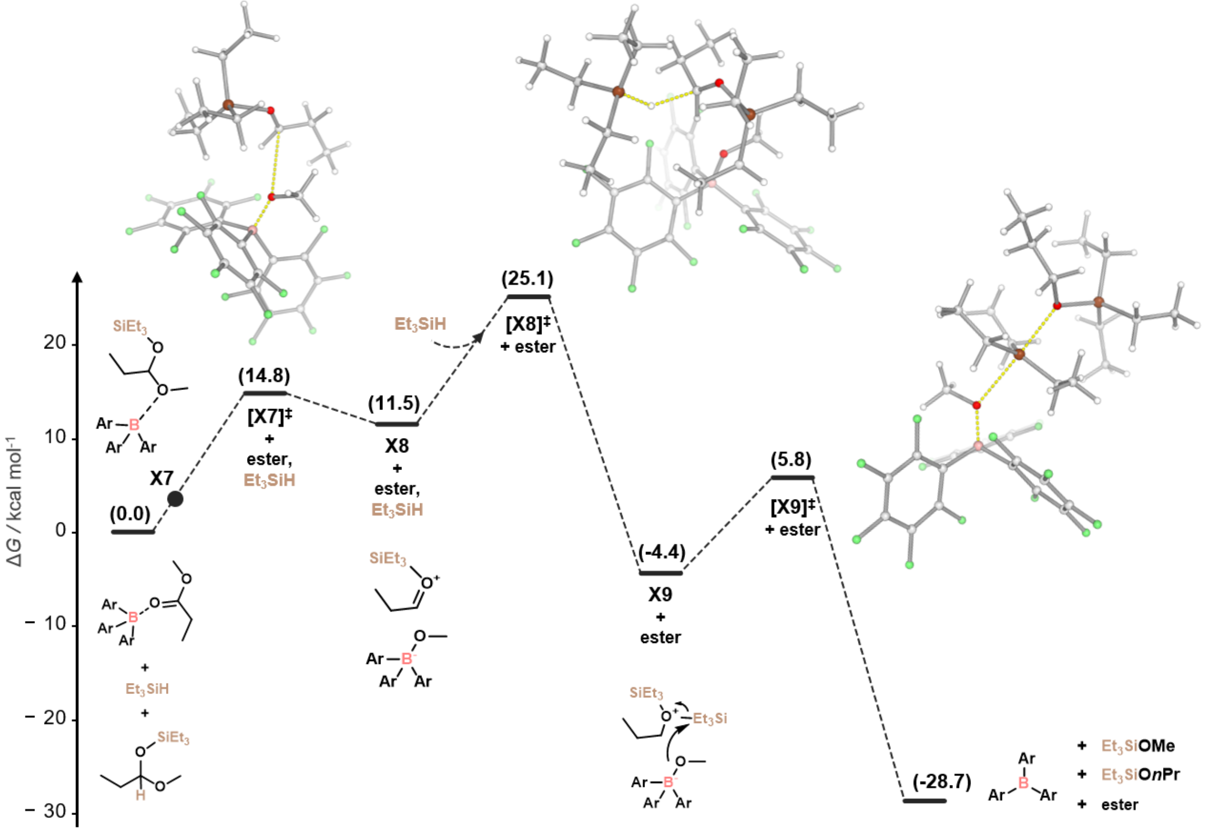

Fig. S15. Free energy diagram of over-reduction, path B, calculated on the DLPNO-ωB97X-2/def2-QZVPP // B97-3c level

The most favorable path was found to be a silyl transfer path having a barrier of 17.3 kcal/mol (Fig. S13, path C and Fig. S16). For this reaction to happen, ester molecules still have to be available in the reaction mixture. Upon collision of the previously formed A4' ion pair with a silyl-acetal, the silylium ion gets transferred via [B4']‡ (17.3 kcal/mol), and dissociation of the ester leads to another ion pair X6 (11.6 kcal/mol). Then, an S_N1-type elimination follows and a TESOMe molecule dissociates leaving behind a highly reactive oxonium cation X11 (5.9 kcal/mol). Along the dissociation step, no transition state was found, the low dissociation barrier can be estimated using the trimolecular coordination complex [X6]‡ (12.0 kcal/mol). X11 collapses through a downhill reaction to give the final products.

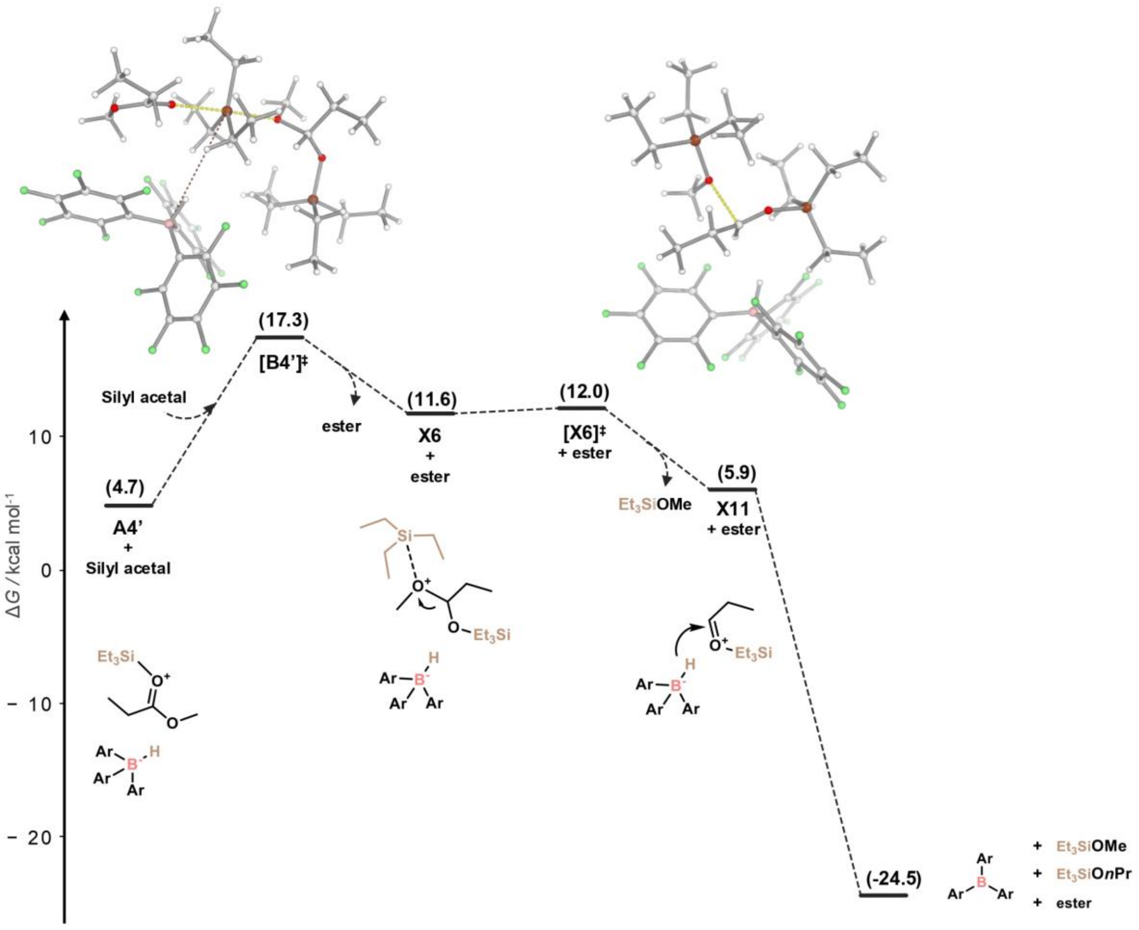

Fig. S16. Free energy diagram of overreduction, path C, calculated on the DLPNO-ωB97X-2/def2-QZVPP // B97-3c level

By mapping the minimum energy path of this latter step, it turns out, it has a negligible barrier both on the GGA and the hybrid meta-GGA levels (Fig. S17).

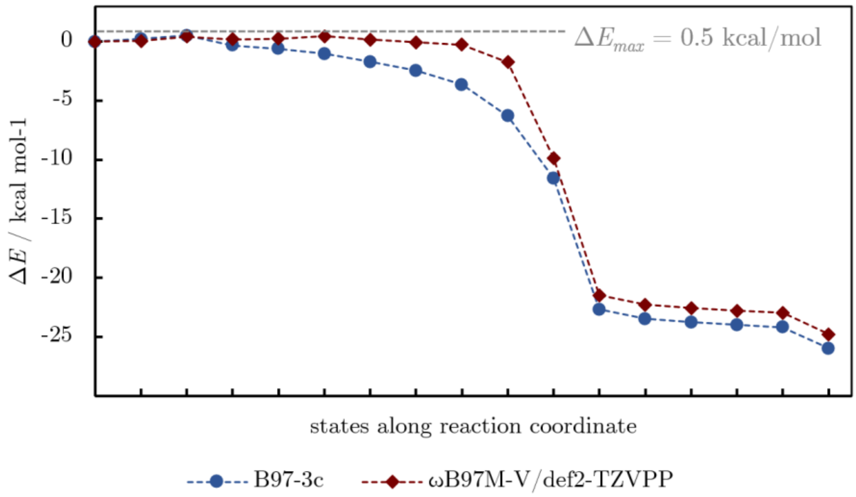

Fig. S17. Minimum energy path of the reaction of X11, calculated on the B97-3c // B97-3c (blue) and ωB97M-V/def2-TZVPP // B97-3c (red) level

These results show that silyl transfer is more favorable than direct Si–H cleavage in the case of a silyl-acetal molecule. Although the transition state of the silyl transfer step is formally tetramolecular, it includes the tight ion pair of the silyl-ester cation and the borohydride anion, therefore dissociation is less likely.

To conclude, in a substrate-competitive scenario, where both ester and silyl-acetal molecules are available, the silyl transfer mechanism is the more probable one. Also, after the depletion of ester, the Piers–Oestreich-type cleavage becomes the best available route to silyl acetal overreduction.

We have also examined Path A and C for the **1d** catalyzed reaction, where the same conclusions can be drawn based on the activation energies (path A: 21.3 kcal/mol, path C: 22.7 kcal/mol). Finally, we have compared the selectivities of catalysts based on the hydride transfer and silyl transfer steps.

2.3 LNO-CCSD(T) results

2.3.1 Comparison of **1a** and **1d** catalyst selectivity

After the most probable mechanistic routes were selected based on the double hybrid level of theory, conformational analysis was performed to find the most stable conformers, and LNO-CCSD(T) energies were calculated for the key states for the final comparison of catalysts. For a detailed discussion, see the manuscript.

We compared the sensitivity of the selectivity determining barrier differences with respect to method and basis size, which is shown in Table S8. These results show that the double-hybrid ωB97X-2 functional is in excellent agreement with the LNO-CCSD(T) method.

Table S8: Sensitivity of the differences in selectivity determining barriers with respect to method and basis set

	DLPNO-ωB97X-2 / def2-QZVPP	LNO-CCSD(T) / def2-TZVPP	LNO-CCSD(T) / def2-QZVPP	LNO-CCSD(T) / CBS
**1a** - $\Delta\Delta G$ / kcal mol^{-1}	3.9	4.2	4.8	4.9
**1d** - $\Delta\Delta G$ / kcal mol^{-1}	4.4	5.1	5.6	5.5
$\Delta\Delta\Delta G$ / kcal mol^{-1}	0.5	0.9	0.8	0.6

2.3.2 Energetics for key stationary points

Table S9: Energetics in Hartrees for key stationary points.

$G_{\text{final}} = E_{\text{CC/CBS}} + (G_{\text{GGA}} - E_{\text{GGA}}) + \Delta E_{\text{solv, hybrid}}$

	$E_{\text{CC/TZ}}$	$E_{\text{CC/QZ}}$	$E_{\text{CC/CBS}}$	$G_{\text{GGA}} - E_{\text{GGA}}$	$\Delta E_{\text{solv, hybrid}}$	G_{final}
**1a**	-2205.413008	-2206.015468	-2206.407663	0.096357	-0.004716	-2206.316022
**1d**	-4182.655309	-4183.159502	-4183.464477	0.138202	-0.006602	-4183.332877
**methyl-propionate**	-307.225025	-307.312585	-307.368952	0.088753	-0.004649	-307.284847
**TESH**	-526.910156	-526.996365	-527.047994	0.170423	-0.001143	-526.878714
**silyl-acetal**	-834.178783	-834.353773	-834.462624	0.284504	-0.004369	-834.182489
**A1**	-4489.913066	-4490.503661	-4490.864673	0.253308	-0.010778	-4490.622144
**[A3]‡**	-5016.815086	-5017.491210	-5017.903669	0.446495	-0.015806	-5017.472980
**A4**	-5016.820633	-5017.498740	-5017.912277	0.444226	-0.019713	-5017.487764
**[A4]‡**	-5016.822171	-5017.499002	-5017.911900	0.445167	-0.010359	-5017.477091
**[B4]‡**	-5851.015538	-5851.866657	-5852.388550	0.756157	-0.018421	-5851.650814
**B5**	-5851.008737	-5851.860649	-5852.382819	0.748484	-0.023827	-5851.658162
**[C3]‡**	-5543.767500	-5544.530559	-5544.995276	0.646380	-0.015792	-5544.364687
**A1’**	-2512.673456	-2513.362197	-2513.810327	0.211063	-0.010113	-2513.609377
**[A3’]‡**	-3039.580573	-3040.354911	-3040.854664	0.404006	-0.012700	-3040.463357
**A4’**	-3039.586201	-3040.362715	-3040.863695	0.401786	-0.018551	-3040.480460
**[A4’]‡**	-3039.584747	-3040.359875	-3040.860080	0.402066	-0.009689	-3040.467703
**[B4’]‡**	-3873.778074	-3874.727177	-3875.336062	0.713290	-0.019535	-3874.642308
**B5’**	-3873.779837	-3874.729091	-3875.338077	0.708181	-0.020736	-3874.650631
**[C3’]‡**	-3566.533574	-3567.394508	-3567.946268	0.603907	-0.012596	-3567.354957
**methyl-silyl-ether**	-641.324202	-641.444051	-641.517302	0.201249	-0.002598	-641.318651
**propyl-silyl-ether**	-719.809887	-719.952237	-720.040007	0.254887	-0.002662	-719.787783

Abbreviations: CC/TZ: LNO-CCSD(T) / def2-TZVPP, CC/QZ: LNO-CCSD(T) / def2-QZVPP, CC/CBS: LNO-CCSD(T) / CBS, GGA: B97-3c, hybrid: ωB97M-V/def2-TZVPP.

2.4 Cartesian coordinates

Table S10. Cartesian coordinates (in Å) of optimized structures

1d

34

B	0.245252	0.141191	-0.261634
C	-0.222434	1.612038	-0.511677
C	-1.170929	2.268855	0.272586
C	-1.582296	3.567439	0.031649
C	-1.043273	4.248745	-1.050892
C	-0.095954	3.647374	-1.864855
C	0.292370	2.357565	-1.568803
C	-0.802705	-1.015599	-0.275799
C	-1.864508	-1.034374	-1.180424
C	-2.813253	-2.043987	-1.173727
C	-2.745910	-3.065766	-0.248572
C	-1.707622	-3.066693	0.662071
C	-0.749934	-2.068098	0.638448
C	1.761853	-0.151499	-0.037694
C	2.563969	0.692587	0.731309
C	3.914395	0.446847	0.920283
C	4.520305	-0.641311	0.325725
C	3.751459	-1.485307	-0.450785
C	2.397658	-1.249060	-0.619228
F	0.224586	-2.108612	1.564539
F	-1.977940	-0.074064	-2.114135
Br	-1.875138	1.378047	1.817516
F	1.208694	1.766553	-2.377582
F	1.703852	-2.097536	-1.398638
F	2.028163	1.759321	1.348517
F	-3.808943	-2.024956	-2.076106
F	4.639534	1.278423	1.687349
F	4.320547	-2.546435	-1.047377
F	-1.625024	-4.046233	1.578572
H	-2.305623	4.041958	0.676943
H	-1.363357	5.260364	-1.257174
H	0.334465	4.159751	-2.712245
H	-3.488482	-3.849188	-0.237874
H	5.574154	-0.829094	0.465245

1a

34

C	-1.513249	-1.551224	0.853119
C	-1.348716	-0.417323	0.057379
C	-2.461729	-0.020602	-0.684156
C	-3.663077	-0.706134	-0.653989
C	-3.785840	-1.822622	0.161689
C	-2.707398	-2.246168	0.926140
B	0.000035	0.362320	0.001255
C	1.348754	-0.417273	-0.056307
C	1.512576	-1.550873	-0.852614
C	2.706614	-2.245873	-0.926868
C	3.785705	-1.822681	-0.163139
C	3.663677	-0.706490	0.653057
C	2.462401	-0.020884	0.684461
F	0.501996	-1.988966	-1.620679
F	2.834084	-3.313533	-1.720005
F	4.702130	-0.311311	1.395099
F	2.387415	1.043141	1.500217
F	-2.386005	1.043768	-1.499396
F	-4.700907	-0.310595	-1.396712
F	-2.835591	-3.314103	1.718788
F	-0.503361	-1.989651	1.621903
C	-0.000022	1.921100	0.000467
C	-0.855503	2.659921	0.818368
C	-0.860048	4.043274	0.839415
C	0.000010	4.736709	-0.000916
C	0.860053	4.042431	-0.840564
C	0.855462	2.659101	-0.818161
F	-1.694454	2.034056	1.659424
F	1.694443	2.032364	-1.658540
F	-1.676838	4.717074	1.654249
F	0.000032	6.067562	-0.001562
F	1.676881	4.715410	-1.656038
F	4.937664	-2.487258	-0.213495
F	-4.937887	-2.487135	0.210872

silyl-acetal

37

C	2.552826	1.041242	-0.414622
C	1.621079	0.127216	0.352110
O	2.169861	-0.076658	1.629370
O	0.341784	0.717009	0.465161
C	1.494424	-1.065077	2.392251
C	3.911330	0.426862	-0.698161
Si	-1.058474	0.201343	-0.304598
C	-2.391958	1.323229	0.389747
C	-2.594888	1.198027	1.897077
C	-1.409197	-1.603939	0.100636
C	-2.714211	-2.108859	-0.511909
C	-0.932262	0.433555	-2.170348
C	-0.052073	-0.568555	-2.912418
H	2.053586	1.303403	-1.347122
H	2.653720	1.964598	0.157566
H	1.537156	-0.847228	-0.152855
H	0.478124	-0.756198	2.639768
H	1.452272	-2.017999	1.855691
H	2.060400	-1.198680	3.309679
H	4.442205	0.199687	0.223573
H	3.814156	-0.500739	-1.264027
H	4.529654	1.105756	-1.283564
H	-3.326989	1.114388	-0.136016
H	-2.132190	2.352776	0.130640
H	-1.674727	1.414366	2.438047
H	-2.906591	0.191568	2.176079
H	-3.358883	1.886013	2.260877
H	-1.437749	-1.717375	1.185837
H	-0.575903	-2.223039	-0.238264
H	-2.705168	-2.031903	-1.598910
H	-3.570639	-1.536591	-0.155838
H	-2.898566	-3.154547	-0.263775
H	-0.583242	1.452533	-2.356504
H	-1.947572	0.396534	-2.575103
H	0.980796	-0.536591	-2.568139
H	-0.037439	-0.374082	-3.985463
H	-0.405129	-1.589786	-2.773581

methyl-propionate

14

C	1.231780	-0.732999	0.147737
C	-0.028116	0.059102	-0.074851
O	-1.120039	-0.690924	0.185102
O	-0.085186	1.215569	-0.436172
C	-2.390230	-0.036495	0.010521
C	2.497018	0.043309	-0.151881
H	1.219831	-1.084999	1.181060
H	1.167073	-1.635585	-0.462453
H	-3.138317	-0.783495	0.249431
H	-2.478068	0.814084	0.681712
H	-2.508917	0.302996	-1.015193
H	2.572336	0.931402	0.471588

H	3.371457	-0.577408	0.034099
H	2.525127	0.366764	-1.190119

TESH

23

Si	0.214012	-0.342827	-0.379775
H	0.604882	-0.897360	-1.714303
C	-0.915510	-1.589122	0.474364
C	-2.225338	-1.854215	-0.261622
C	-0.682193	1.291980	-0.669519
C	-1.138773	1.974716	0.616682
C	1.781125	-0.081250	0.637062
C	2.754592	0.929257	0.037978
H	-1.120608	-1.233135	1.487221
H	-0.359737	-2.521907	0.598346
H	-2.835280	-2.598682	0.251714
H	-2.047310	-2.221168	-1.272813
H	-2.825678	-0.948934	-0.348769
H	-1.539344	1.108914	-1.321328
H	-0.023219	1.955988	-1.233307
H	-1.655137	2.914092	0.415211
H	-0.296887	2.203131	1.270320
H	-1.824350	1.344526	1.183308
H	2.273289	-1.049336	0.759025
H	1.491597	0.229701	1.644117
H	3.066936	0.636293	-0.964747
H	3.656622	1.030914	0.642768
H	2.305218	1.918867	-0.041025

methyl-silyl-ether

27

C	-1.109906	1.349644	0.277074
C	-0.651129	2.750590	-0.120190
Si	0.025812	-0.029122	-0.296620
C	-0.558165	-1.702868	0.336887
C	-1.994679	-2.047834	-0.045728
C	1.797098	0.299620	0.264372
C	2.739861	-0.901713	0.272250
O	-0.096376	0.014098	-1.969613
H	-1.203031	1.278385	1.364442
H	-2.109695	1.158665	-0.118884
H	-1.348799	3.515972	0.221882
H	-0.565953	2.845781	-1.201889
H	0.324152	2.988161	0.304066
H	1.744261	0.726902	1.270156
H	2.206923	1.094343	-0.364075
H	2.386778	-1.680634	0.946757
H	3.743131	-0.622558	0.596823
H	2.834361	-1.351785	-0.715203
H	-0.442658	-1.712322	1.424606
H	0.120852	-2.476296	-0.030276
H	-2.702813	-1.332905	0.371978
H	-2.283464	-3.037173	0.311286
H	-2.129381	-2.038140	-1.127033
C	0.679903	-0.813867	-2.817069
H	0.297671	-0.719961	-3.832460
H	1.730585	-0.513567	-2.818284
H	0.623537	-1.867029	-2.527918

propyl-silyl-ether

33

C	-1.120886	1.436860	0.209393
C	-0.593450	2.712419	-0.441566
Si	-0.075557	-0.088341	-0.121637
C	-0.759669	-1.554045	0.843895
C	-2.198831	-1.919178	0.491337
C	1.720894	0.231983	0.358699
C	2.606910	-0.994825	0.562077
O	-0.227301	-0.334355	-1.771609
H	-1.197986	1.568693	1.292374
H	-2.137355	1.236163	-0.137020
H	-1.245205	3.565601	-0.249009
H	-0.514770	2.598538	-1.522164
H	0.397734	2.971818	-0.069885
H	1.697827	0.819810	1.281112
H	2.162270	0.894409	-0.390167
H	2.219054	-1.640436	1.348797
H	3.622349	-0.713873	0.844346
H	2.679660	-1.599360	-0.341000
H	-0.681799	-1.316134	1.909317
H	-0.109703	-2.417832	0.690572
H	-2.883419	-1.097493	0.699798
H	-2.548299	-2.783768	1.057100
H	-2.296620	-2.159013	-0.566804
C	0.701181	-1.008066	-2.607843
H	0.491529	-0.690291	-3.631871
H	1.723355	-0.686545	-2.381435
C	0.603405	-2.518198	-2.518287
C	1.562209	-3.208757	-3.473771
H	0.811396	-2.833723	-1.494545
H	-0.425164	-2.814369	-2.731466
H	1.476017	-4.291939	-3.405591
H	1.363444	-2.926014	-4.507902
H	2.597544	-2.945934	-3.253966

A1'

48

C	0.842342	-2.548820	0.274025
C	1.022316	-1.172103	0.371342
C	2.045998	-0.776840	1.224132
C	2.860477	-1.673517	1.902054
C	2.656697	-3.034163	1.752039
C	1.636903	-3.477256	0.926465
B	-0.010048	-0.163202	-0.399834
O	-0.040351	-0.744956	-1.873134
C	-0.625452	-0.319915	-2.895128
O	-0.446503	-0.953210	-4.018025
C	0.433057	-2.114139	-4.025247
F	2.309920	0.526193	1.443431
F	3.840570	-1.234962	2.704644
F	1.419973	-4.793129	0.781658
F	-0.161048	-3.053430	-0.478028
C	-1.454281	-0.353031	0.336977
C	-2.582738	-0.984641	-0.165400
C	-3.735311	-1.199123	0.579251
C	-3.783384	-0.788720	1.898896
C	-2.675125	-0.166714	2.452765
C	-1.549229	0.026838	1.673091
F	-2.629922	-1.440847	-1.441201
F	-0.500749	0.629958	2.267831
C	0.537055	1.360494	-0.589388
C	1.779542	1.557060	-1.187457
C	2.337004	2.803440	-1.413744
C	1.628092	3.937554	-1.049868
C	0.376742	3.797137	-0.476384
C	-0.144531	2.526950	-0.267662
F	2.516961	0.494925	-1.576552
F	3.544935	2.926683	-1.980294
F	-0.328648	4.887711	-0.147682
F	-1.391956	2.490313	0.243555
C	-1.515207	0.866064	-2.993810
C	-0.882161	1.999506	-3.802257
H	0.048973	2.335878	-3.355559
H	-2.430370	0.535095	-3.482718
H	-1.777484	1.196435	-1.996677
H	0.435793	-2.451305	-5.052938
H	-1.568755	2.841336	-3.827538
H	0.035289	-2.875300	-3.363431
H	-0.682188	1.688855	-4.823940
H	1.427322	-1.819857	-3.706722
F	-4.797292	-1.808560	0.032346
F	-4.883692	-0.988642	2.631340
F	-2.706355	0.235427	3.730150
F	2.141850	5.152007	-1.263834
F	3.432661	-3.910713	2.398369

A2'

57

C	1.630807	-0.786632	-1.522627
C	1.558342	-0.427385	-0.181679
C	2.718962	-0.644333	0.552865
C	3.858493	-1.221082	0.010378
C	3.869801	-1.589220	-1.324876
C	2.741036	-1.373968	-2.100720
B	0.228392	0.224437	0.422908
C	-0.325069	1.547855	-0.285420
C	-1.662325	1.910857	-0.174167
C	-2.207205	3.027456	-0.780904
C	-1.381240	3.866631	-1.512978
C	-0.034280	3.565093	-1.628376
C	0.468793	2.428007	-1.012454
F	-2.503512	1.145533	0.554091
F	-3.509955	3.313879	-0.657510
F	0.770189	4.380331	-2.321791
F	1.792335	2.219939	-1.141978
F	2.796215	-0.283119	1.847216
F	4.951086	-1.413495	0.759940
F	2.743917	-1.720765	-3.394611
F	0.570114	-0.570288	-2.329961
C	-0.000831	0.089647	2.000496
C	-0.167748	1.173762	2.855053
C	-0.375742	1.033646	4.219794
C	-0.405236	-0.232357	4.780985
C	-0.224383	-1.342013	3.969562
C	-0.009103	-1.156505	2.616383
F	-0.119323	2.435642	2.389016
F	0.194069	-2.268005	1.876773
Si	-1.827712	-1.789245	-0.574562
C	-2.869721	-2.165659	0.937884
C	-4.283777	-2.634611	0.592323
C	-0.793655	-3.231865	-1.178020
C	-1.545025	-4.563629	-1.148681
C	-2.737344	-0.869747	-1.931614
C	-3.358090	-1.798094	-2.976414
H	-0.759833	-0.740823	-0.050986
F	-0.535690	2.108813	5.001469
F	-0.604066	-0.383810	6.092605
F	-0.244431	-2.570359	4.503560
F	-1.880550	4.957378	-2.099342
F	4.958641	-2.144979	-1.861761
H	0.101155	-3.299059	-0.559415
H	-0.448803	-3.015697	-2.188938
H	-2.433551	-4.544761	-1.777696
H	-1.861258	-4.824954	-0.140424
H	-0.908980	-5.371329	-1.509426
H	-2.038597	-0.186072	-2.413469
H	-3.506602	-0.246514	-1.476173
H	-2.603492	-2.401763	-3.477462
H	-3.874912	-1.221913	-3.743222
H	-4.085352	-2.478753	-2.536829
H	-2.918412	-1.269485	1.556179
H	-2.357909	-2.919565	1.535609
H	-4.851038	-2.844342	1.498584
H	-4.277055	-3.545314	-0.004352
H	-4.832768	-1.879120	0.033107

A3'

71

C	2.993558	0.465641	-1.380038
C	3.013736	0.965215	-0.083486
C	4.242594	0.880418	0.561284
C	5.360625	0.296358	-0.017435
C	5.279719	-0.213480	-1.302671
C	4.079831	-0.133104	-1.991844
B	1.698883	1.627845	0.553708
C	1.062293	2.867562	-0.241404
C	-0.265449	3.226686	-0.041775
C	-0.887044	4.273217	-0.698641
C	-0.149596	5.045671	-1.582151
C	1.185818	4.746468	-1.795269
C	1.766841	3.681608	-1.121127
F	-1.018266	2.529010	0.834930
F	-2.178487	4.556525	-0.479987
F	1.906123	5.497354	-2.638810
F	3.077593	3.479610	-1.354675
F	4.413313	1.384266	1.798258
F	6.521342	0.232980	0.648021
F	3.990707	-0.616808	-3.238507
F	1.859629	0.551921	-2.107185
C	1.636350	1.693486	2.155308
C	1.520659	2.868633	2.889182
C	1.468411	2.891327	4.275749
C	1.555311	1.704365	4.984168
C	1.691432	0.509343	4.295265
C	1.747297	0.533933	2.913496
F	1.467545	4.066709	2.276499
F	1.914147	-0.652913	2.292564
Si	-0.376760	-0.551209	-0.000919
C	-1.305006	-0.576557	1.633989
C	-2.772237	-0.164529	1.579909
C	0.737862	-2.022128	-0.363474
C	0.639271	-3.194002	0.607878
C	-1.300666	0.179414	-1.469057
C	-1.145249	-0.540636	-2.804228
O	-2.470816	-2.782219	-0.542909
C	-3.300903	-3.057818	-1.389677
O	-3.168212	-4.092749	-2.235650
C	-1.973688	-4.886581	-2.082012
C	-4.593511	-2.323339	-1.589328
C	-5.610013	-2.707218	-0.515653
H	-4.375227	-1.258656	-1.528693
H	-4.988590	-2.534781	-2.579838
H	-1.089678	-4.275313	-2.240022
H	-1.935391	-5.326046	-1.089142
H	-2.039764	-5.660062	-2.837575
H	-5.225504	-2.497732	0.479990
H	-6.527354	-2.138602	-0.651194
H	-5.858500	-3.765545	-0.569994
H	0.727645	0.611738	0.284679
F	1.349835	4.050037	4.937240
F	1.508128	1.710482	6.319252
F	1.782422	-0.644759	4.969929
F	-0.722681	6.068555	-2.222560
F	6.347691	-0.777830	-1.873377
H	1.766683	-1.664244	-0.401977
H	0.504696	-2.353401	-1.375769
H	-0.368196	-3.599167	0.636263
H	0.913393	-2.905346	1.619538
H	1.312588	-3.996270	0.305310
H	-0.988738	1.217712	-1.581400
H	-2.352326	0.221667	-1.184771
H	-0.113913	-0.543486	-3.146256
H	-1.742572	-0.051116	-3.573282
H	-1.471906	-1.575773	-2.748137
H	-0.771620	0.063465	2.336433
H	-1.216431	-1.588133	2.030975
H	-3.218315	-0.215013	2.573187
H	-3.339069	-0.823750	0.928942
H	-2.896799	0.853620	1.219352

[A3']‡

71

C	0.853564	2.051650	-1.486479
C	1.344105	1.334018	-0.402694
C	2.346952	1.977404	0.311612
C	2.793167	3.258146	0.012352
C	2.248139	3.940958	-1.061010
C	1.263541	3.329725	-1.820479
B	0.754748	-0.139128	-0.074936
C	1.082391	-0.683601	1.415111
C	1.862346	-1.794692	1.709175
C	2.085353	-2.246997	3.003063
C	1.536684	-1.561331	4.073080
C	0.773033	-0.430909	3.830964
C	0.581523	-0.016233	2.525354
F	2.466839	-2.499529	0.730910
F	2.838917	-3.333407	3.229552
F	0.244633	0.251698	4.859572

F	-0.136260	1.117536	2.354700
F	2.962280	1.373641	1.349052
F	3.757707	3.836427	0.743761
F	0.734439	3.974686	-2.872431
F	-0.079995	1.492234	-2.288614
C	0.963867	-1.229247	-1.254879
C	1.880482	-1.126067	-2.293515
C	1.994548	-2.070956	-3.304589
C	1.186840	-3.194906	-3.287030
C	0.277362	-3.356764	-2.254498
C	0.202155	-2.390306	-1.267519
F	2.735228	-0.085003	-2.368137
F	-0.668010	-2.627158	-0.260063
Si	-2.358450	0.325265	0.008317
C	-2.101711	2.172921	0.226795
C	-2.660235	3.046775	-0.892113
C	-2.542672	-0.848377	1.463657
C	-3.103990	-0.292626	2.766655
C	-2.586837	-0.344461	-1.731221
C	-3.381301	-1.639540	-1.858729
O	-4.563472	0.580170	0.035420
C	-5.492897	1.218362	0.533528
O	-6.747653	0.941495	0.222999
C	-6.981109	-0.130032	-0.721508
C	-5.348887	2.364087	1.485569
C	-6.567604	2.698359	2.325557
H	-4.488283	2.152184	2.112567
H	-5.060395	3.223192	0.874872
H	-8.055171	-0.163490	-0.851998
H	-6.484785	0.085412	-1.662454
H	-6.614788	-1.068170	-0.316715
H	-6.871916	1.850993	2.936125
H	-6.331042	3.524188	2.992542
H	-7.413748	2.993076	1.711491
H	-0.530506	0.042223	-0.055986
F	2.889478	-1.914403	-4.291761
F	1.285953	-4.116630	-4.252526
F	-0.500907	-4.450119	-2.214569
F	1.743135	-1.982547	5.326628
F	2.668355	5.174923	-1.364321
H	-1.567592	-1.291931	1.645886
H	-3.168124	-1.669103	1.108477
H	-2.579448	0.601964	3.094513
H	-3.009767	-1.029744	3.563536
H	-4.160627	-0.051213	2.681648
H	-1.603794	-0.466570	-2.179045
H	-3.072106	0.449380	-2.301845
H	-3.474701	-1.927091	-2.905945
H	-4.384215	-1.535663	-1.454450
H	-2.900160	-2.463703	-1.339359
H	-1.036389	2.357551	0.334671
H	-2.533001	2.452146	1.187104
H	-2.146225	2.866551	-1.832192
H	-2.536273	4.102306	-0.651403
H	-3.719629	2.873067	-1.069113

A4'

71

C	-0.301008	0.109088	-2.249126
C	-0.092400	0.149276	-0.874642
C	1.228162	-0.055097	-0.496184
C	2.261575	-0.280711	-1.397453
C	1.995620	-0.312166	-2.753925
C	0.693835	-0.114570	-3.184947
B	-1.375538	0.262687	0.128551
C	-2.385818	1.512422	-0.168243
C	-3.689549	1.445159	0.306816
C	-4.636390	2.437996	0.113875
C	-4.281183	3.584266	-0.577567
C	-2.988024	3.709307	-1.056098
C	-2.073786	2.687985	-0.836700
F	-4.100878	0.359036	1.007564
F	-5.888741	2.306414	0.588931
F	-2.633324	4.823028	-1.719592
F	-0.830219	2.899615	-1.319235
F	1.590072	-0.074655	0.806673
F	3.518277	-0.484596	-0.965149
F	0.415169	-0.147557	-4.500698
F	-1.551478	0.276809	-2.745784
C	-1.008508	0.283718	1.717600
C	-0.498824	1.409667	2.350198
C	-0.238794	1.476002	3.709458
C	-0.487783	0.366301	4.502432
C	-0.988326	-0.783298	3.916797
C	-1.232416	-0.797881	2.551515
F	-0.203688	2.510342	1.626436
F	-1.708068	-1.969244	2.053505
Si	-1.320938	-4.643106	-0.757577
C	-1.218666	-5.948672	0.566984
C	-0.984528	-5.424127	1.981331
C	-1.623961	-5.371516	-2.450560
C	-2.867547	-6.248095	-2.579285
C	0.050177	-3.390025	-0.705731
C	1.421441	-4.037597	-0.905493
O	-2.809523	-3.788687	-0.260653
C	-3.571437	-2.919422	-0.782990
O	-4.591164	-2.504701	-0.117001
C	-4.820258	-2.996690	1.237139
C	-3.405010	-2.381787	-2.153388
C	-4.348487	-1.263368	-2.543133
H	-2.365794	-2.069771	-2.244428
H	-3.512910	-3.237092	-2.825036
H	-5.734854	-2.512750	1.549949
H	-4.931754	-4.075213	1.214989
H	-3.988290	-2.705581	1.865564
H	-4.224982	-0.403708	-1.892847
H	-4.126003	-0.950005	-3.559066
H	-5.386337	-1.583221	-2.503525
H	-2.019656	-0.746098	-0.086835
F	0.259856	2.590954	4.268806
F	-0.243131	0.405235	5.820382
F	-1.229988	-1.869022	4.676040
F	-5.178354	4.561162	-0.778322
F	2.978952	-0.536660	-3.638093
H	-0.732379	-5.964807	-2.673250
H	-1.625675	-4.577643	-3.198658
H	-3.782355	-5.695266	-2.366365
H	-2.834625	-7.092421	-1.893067
H	-2.961271	-6.650544	-3.586760
H	0.012366	-2.865804	0.247728
H	-0.115876	-2.629303	-1.467107
H	1.634276	-4.782336	-0.139720
H	2.207497	-3.286661	-0.854973
H	1.503974	-4.527217	-1.875140
H	-0.410448	-6.625483	0.276422
H	-2.127983	-6.552117	0.529763
H	-0.904472	-6.244574	2.693188
H	-1.798144	-4.781996	2.310931
H	-0.067179	-4.842216	2.048791

[A4']‡

71

C	0.221667	-2.471408	0.122974
C	1.165318	-1.453331	0.018692
C	2.487330	-1.887377	0.003127
C	2.852940	-3.219294	0.145826
C	1.873972	-4.187842	0.285486
C	0.540980	-3.808721	0.270786
B	0.694796	0.087361	-0.051765
C	-0.367740	0.481255	-1.196410
C	-1.070354	1.680538	-1.122649
C	-1.974926	2.103547	-2.080206
C	-2.208467	1.306371	-3.189154
C	-1.523403	0.110671	-3.318828
C	-0.615247	-0.268942	-2.340919
F	-0.851012	2.529004	-0.096186
F	-2.611828	3.277943	-1.959997
F	-1.732590	-0.659114	-4.396795
F	0.051502	-1.416324	-2.577402
F	3.504917	-1.020546	-0.168041
F	4.144314	-3.579593	0.136221
F	-0.418969	-4.739817	0.382362
F	-1.090745	-2.174179	0.056709
C	1.841741	1.196405	0.207197

C	2.239805	2.138199	-0.737915
C	3.204792	3.105276	-0.492693
C	3.842684	3.141812	0.735064
C	3.506812	2.203272	1.697043
C	2.543803	1.254319	1.405183
F	1.709784	2.149516	-1.977108
F	2.319282	0.326083	2.363920
O	-1.160253	-1.041068	2.654438
C	-0.078174	-1.475182	3.496164
C	-1.177290	0.198747	2.151811
O	-2.263637	0.459114	1.471318
Si	-3.855214	-0.212075	1.318402
C	-4.204276	-1.566678	2.566129
C	-3.772716	-2.982622	2.200917
C	-0.629521	1.348961	2.954470
C	-1.586225	1.720095	4.085292
C	-4.939531	1.269221	1.690293
C	-4.695722	2.485662	0.804110
C	-4.034993	-0.766512	-0.454883
C	-5.482192	-1.092392	-0.820916
H	0.348857	1.096668	3.344757
H	-0.500842	2.187007	2.278555
H	0.861524	-1.412357	2.960646
H	-0.034619	-0.892965	4.412227
H	-0.301934	-2.509360	3.728623
H	-1.162324	2.539487	4.661403
H	-2.547233	2.043780	3.694758
H	-1.757846	0.886700	4.764135
H	-0.095801	0.162988	1.076824
F	3.537947	3.994554	-1.438643
F	4.777356	4.063818	0.987001
F	4.133977	2.208598	2.883062
F	-3.084656	1.689998	-4.124042
F	2.208994	-5.474982	0.423006
H	-4.812579	1.532700	2.743231
H	-5.977331	0.937653	1.598743
H	-5.362923	3.308078	1.061853
H	-4.858662	2.253755	-0.247473
H	-3.674705	2.849715	0.899008
H	-3.397944	-1.631382	-0.631254
H	-3.653928	0.021736	-1.103350
H	-6.129260	-0.221945	-0.721841
H	-5.894333	-1.878131	-0.187824
H	-5.557745	-1.436541	-1.851930
H	-5.288696	-1.539656	2.711561
H	-3.780782	-1.271177	3.528004
H	-2.695896	-3.052660	2.078851
H	-4.227099	-3.309647	1.266969
H	-4.067181	-3.694795	2.971940

[B4']‡

108

C	4.952448	3.550766	-1.904474
C	3.550750	3.111706	-1.550713
O	3.436243	2.799456	-0.104605
O	2.634831	4.077635	-1.885962
C	3.671693	3.940564	0.743472
C	5.157918	3.569088	-3.409849
Si	1.064369	3.774679	-2.486396
C	-0.019923	3.101334	-1.116687
C	-0.230644	4.061475	0.049676
C	1.193273	2.567098	-3.917064
C	-0.139203	2.365337	-4.638766
C	0.503410	5.469968	-3.046398
C	1.315090	6.054169	-4.199051
H	5.130767	4.542868	-1.491459
H	5.659016	2.867245	-1.432842
H	3.325168	2.157051	-2.014063
H	3.184073	4.808563	0.314488
H	3.231106	3.727385	1.708298
H	4.736658	4.123186	0.857529
H	6.175708	3.864920	-3.655559
H	4.982931	2.584928	-3.844543
H	4.478805	4.270744	-3.888591
H	0.400785	2.157803	-0.764704
H	-0.984031	2.831389	-1.551643
H	-0.776148	3.587382	0.863514
H	-0.800439	4.937793	-0.255579
H	0.714328	4.419508	0.456998
H	1.558122	1.603754	-3.557027
H	1.948557	2.933764	-4.615714
H	-0.040246	1.665033	-5.467083
H	-0.521061	3.301133	-5.045975
H	-0.900372	1.965373	-3.970759
H	0.537244	6.141142	-2.185283
H	-0.551949	5.402225	-3.321673
H	1.256971	5.427609	-5.088408
H	2.367991	6.146156	-3.934935
H	0.960209	7.046529	-4.477853
C	-3.013274	-1.812224	-1.602101
C	-1.767565	-1.244967	-1.370926
C	-0.977461	-1.097048	-2.499180
C	-1.379282	-1.478720	-3.771905
C	-2.632765	-2.036823	-3.950689
C	-3.461679	-2.204533	-2.852359
B	-1.253303	-0.870895	0.133548
C	-1.234411	-2.277806	0.973495
C	-0.242885	-3.208664	0.688206
C	-0.133707	-4.442489	1.308139
C	-1.057055	-4.794603	2.277832
C	-2.072956	-3.908457	2.592635
C	-2.149844	-2.688639	1.933534
F	0.698400	-2.933478	-0.249693
F	0.854694	-5.298433	0.986827
F	-2.978105	-4.247069	3.527268
F	-3.184141	-1.897446	2.288061
F	0.258513	-0.550150	-2.414742
F	-0.566614	-1.316605	-4.833395
F	-4.683924	-2.739145	-3.018861
F	-3.871486	-1.982389	-0.572792
C	-2.040731	0.368059	0.848364
C	-2.893466	1.259637	0.209292
C	-3.466250	2.361728	0.831234
C	-3.186489	2.622124	2.160017
C	-2.329997	1.773015	2.840511
C	-1.781722	0.689681	2.176210
F	-3.209380	1.112468	-1.098567
F	-0.945123	-0.085815	2.910167
Si	3.340971	0.893904	0.840853
C	1.856100	1.432715	1.843747
C	2.103741	1.639721	3.334069
C	5.089275	1.160747	1.488155
C	5.763244	0.112451	2.362683
C	3.059084	-0.000454	-0.777673
C	4.302315	-0.314100	-1.603665
O	3.082872	-0.986903	1.953074
C	3.192844	-2.214499	1.905332
O	2.544972	-2.989040	2.752805
C	1.643614	-2.355025	3.693585
C	4.015507	-2.970008	0.912355
C	4.546413	-4.314821	1.377237
H	3.375980	-3.102664	0.036731
H	4.821708	-2.314872	0.597288
H	0.901217	-1.773885	3.159830
H	1.177467	-3.171433	4.229714
H	2.203004	-1.718825	4.371754
H	3.740539	-5.003457	1.610839
H	5.151165	-4.757484	0.589047
H	5.171033	-4.208897	2.262001
H	-0.101119	-0.502880	0.060073
F	-4.274929	3.192477	0.149723
F	-3.720744	3.688327	2.775370
F	-2.034614	2.018339	4.130674
F	-0.968049	-5.979535	2.902100
F	-3.041008	-2.411944	-5.172654
H	5.065409	2.103622	2.034974
H	5.703677	1.359305	0.607591
H	5.898773	-0.830836	1.840085
H	5.191317	-0.090651	3.264239
H	6.751190	0.456553	2.668777
H	2.343275	0.571724	-1.363744
H	2.521524	-0.915175	-0.545358
H	5.017259	-0.918235	-1.049550
H	4.831683	0.582919	-1.917760
H	4.036855	-0.866535	-2.503970
H	1.105637	0.658961	1.697460

H	1.435675	2.331172	1.401739
H	2.486698	0.737018	3.802279
H	1.178543	1.907054	3.842348
H	2.822699	2.435703	3.527415

B5'

108

C	-1.543926	-0.562617	-3.463644
C	-1.679729	-1.644274	-2.423904
O	-3.159951	-1.822541	-2.070043
O	-1.245023	-2.875609	-2.790950
C	-3.967498	-2.431665	-3.120592
C	-2.270581	0.726834	-3.137044
Si	0.297835	-3.423629	-3.278970
C	0.028975	-5.271442	-3.399409
C	-1.012101	-5.694267	-4.431568
C	1.566846	-2.956086	-1.986484
C	1.631864	-3.856976	-0.757304
C	0.739690	-2.684028	-4.947409
C	1.959833	-3.351213	-5.581828
H	-0.473883	-0.368717	-3.522245
H	-1.829450	-0.953544	-4.439231
H	-1.272150	-1.313778	-1.468154
H	-4.994860	-2.158040	-2.924667
H	-3.652511	-2.018248	-4.068839
H	-3.835289	-3.506404	-3.112000
H	-3.350311	0.599314	-3.171824
H	-2.003228	1.091590	-2.148560
H	-2.001258	1.497574	-3.854742
H	-0.248437	-5.640137	-2.410118
H	0.992360	-5.735654	-3.623817
H	-0.738527	-5.369564	-5.434917
H	-1.988962	-5.266568	-4.208298
H	-1.130219	-6.777468	-4.461945
H	1.388516	-1.922288	-1.685591
H	2.540563	-2.939957	-2.480537
H	1.929767	-4.870193	-1.023271
H	0.671879	-3.923039	-0.248871
H	2.353565	-3.480833	-0.032922
H	0.930642	-1.616535	-4.832979
H	-0.121587	-2.765826	-5.613771
H	1.792059	-4.412144	-5.763295
H	2.841695	-3.264406	-4.947929
H	2.207804	-2.892508	-6.538559
C	0.414918	-0.578309	1.789510
C	0.827730	0.732078	1.597149
C	1.180083	1.401148	2.760963
C	1.106998	0.829042	4.024261
C	0.686606	-0.482879	4.157915
C	0.335386	-1.197409	3.024813
B	0.837072	1.342908	0.085226
C	2.043580	0.769741	-0.853910
C	1.952235	0.932026	-2.231108
C	2.868873	0.420609	-3.134237
C	3.960005	-0.288945	-2.661109
C	4.110289	-0.460889	-1.296667
C	3.167092	0.074643	-0.429462
F	0.915459	1.625154	-2.760982
F	2.712516	0.595624	-4.460299
F	5.166007	-1.149781	-0.829307
F	3.406453	-0.120920	0.886849
F	1.636325	2.671997	2.719169
F	1.447631	1.527910	5.120165
F	-0.067745	-2.476362	3.139914
F	0.085968	-1.340894	0.718320
C	0.731373	2.966661	0.051097
C	1.803917	3.830753	-0.114644
C	1.673547	5.210259	-0.175837
C	0.418470	5.781577	-0.045101
C	-0.681425	4.961284	0.142055
C	-0.501356	3.587754	0.186884
F	3.063830	3.348450	-0.203889
F	-1.618991	2.846880	0.387844
Si	-3.588232	-2.044396	-0.302569
C	-3.270586	-0.371296	0.434430
C	-4.334836	0.673742	0.109050
C	-2.514295	-3.425987	0.330539
C	-2.805908	-4.805235	-0.252274
C	-5.398258	-2.500513	-0.308329
C	-5.872817	-2.697470	1.135672
O	-3.266422	-1.005335	3.798346
C	-2.869788	-0.486368	4.824551
O	-2.423360	0.779995	4.889695
C	-2.444745	1.532524	3.660795
C	-2.828857	-1.147502	6.173690
C	-2.942202	-2.657023	6.111024
H	-1.917937	-0.834192	6.681042
H	-3.649156	-0.720721	6.756881
H	-2.035834	2.504958	3.906802
H	-1.831085	1.050147	2.907295
H	-3.461999	1.631089	3.291357
H	-3.866543	-2.964607	5.627715
H	-2.114590	-3.088587	5.551795
H	-2.926662	-3.075701	7.115239
H	-0.178007	0.927947	-0.436637
F	2.747214	6.002006	-0.342707
F	0.270058	7.114232	-0.095084
F	-1.904514	5.507280	0.277091
F	4.851429	-0.813688	-3.516816
F	0.616602	-1.055417	5.369684
H	-2.654214	-3.432312	1.414909
H	-1.467408	-3.168383	0.188370
H	-2.621849	-4.830189	-1.324136
H	-3.837343	-5.110424	-0.082993
H	-2.165528	-5.560143	0.202095
H	-5.571572	-3.412010	-0.880120
H	-5.982565	-1.714466	-0.788201
H	-5.353309	-3.522486	1.619697
H	-6.937979	-2.920952	1.164367
H	-5.708153	-1.810184	1.744116
H	-2.282884	-0.013181	0.145136
H	-3.213025	-0.520244	1.513554
H	-4.447220	0.824986	-0.963139
H	-4.072857	1.635943	0.543603
H	-5.308575	0.391015	0.505270

[C3’]‡

71

C	0.221667	-2.471408	0.122974
C	1.165318	-1.453331	0.018692
C	2.487330	-1.887377	0.003127
C	2.852940	-3.219294	0.145826
C	1.873972	-4.187842	0.285486
C	0.540980	-3.808721	0.270786
B	0.694796	0.087361	-0.051765
C	-0.367740	0.481255	-1.196410
C	-1.070354	1.680538	-1.122649
C	-1.974926	2.103547	-2.080206
C	-2.208467	1.306371	-3.189154
C	-1.523403	0.110671	-3.318828
C	-0.615247	-0.268942	-2.340919
F	-0.851012	2.529004	-0.096186
F	-2.611828	3.277943	-1.959997
F	-1.732590	-0.659114	-4.396795
F	0.051502	-1.416324	-2.577402
F	3.504917	-1.020546	-0.168041
F	4.144314	-3.579593	0.136221
F	-0.418969	-4.739817	0.382362
F	-1.090745	-2.174179	0.056709
C	1.841741	1.196405	0.207197
C	2.239805	2.138199	-0.737915
C	3.204792	3.105276	-0.492693
C	3.842684	3.141812	0.735064
C	3.506812	2.203272	1.697043
C	2.543803	1.254319	1.405183
F	1.709784	2.149516	-1.977108
F	2.319282	0.326083	2.363920
O	-1.160253	-1.041068	2.654438
C	-0.078174	-1.475182	3.496164
C	-1.177290	0.198747	2.151811
O	-2.263637	0.459114	1.471318
Si	-3.855214	-0.212075	1.318402
C	-4.204276	-1.566678	2.566129
C	-3.772716	-2.982622	2.200917

C	-0.629521	1.348961	2.954470
C	-1.586225	1.720095	4.085292
C	-4.939531	1.269221	1.690293
C	-4.695722	2.485662	0.804110
C	-4.034993	-0.766512	-0.454883
C	-5.482192	-1.092392	-0.820916
H	0.348857	1.096668	3.344757
H	-0.500842	2.187007	2.278555
H	0.861524	-1.412357	2.960646
H	-0.034619	-0.892965	4.412227
H	-0.301934	-2.509360	3.728623
H	-1.162324	2.539487	4.661403
H	-2.547233	2.043780	3.694758
H	-1.757846	0.886700	4.764135
H	-0.095801	0.162988	1.076824
F	3.537947	3.994554	-1.438643
F	4.777356	4.063818	0.987001
F	4.133977	2.208598	2.883062
F	-3.084656	1.689998	-4.124042
F	2.208994	-5.474982	0.423006
H	-4.812579	1.532700	2.743231
H	-5.977331	0.937653	1.598743
H	-5.362923	3.308078	1.061853
H	-4.858662	2.253755	-0.247473
H	-3.674705	2.849715	0.899008
H	-3.397944	-1.631382	-0.631254
H	-3.653928	0.021736	-1.103350
H	-6.129260	-0.221945	-0.721841
H	-5.894333	-1.878131	-0.187824
H	-5.557745	-1.436541	-1.851930
H	-5.288696	-1.539656	2.711561
H	-3.780782	-1.271177	3.528004
H	-2.695896	-3.052660	2.078851
H	-4.227099	-3.309647	1.266969
H	-4.067181	-3.694795	2.971940

A1

48

C	0.813527	-2.204979	0.711911
C	0.218739	-1.373195	-0.231613
C	-0.196447	-2.011315	-1.397787
C	0.014209	-3.359713	-1.636272
C	0.629423	-4.154407	-0.692475
C	1.022677	-3.557931	0.486303
B	-0.106007	0.216658	-0.005853
C	0.825375	0.953113	1.112767
C	2.226992	0.927327	1.064607
C	3.053518	1.534955	1.996155
C	2.485243	2.235324	3.047446
C	1.110077	2.335972	3.136775
C	0.335577	1.711611	2.176892
Br	3.171360	0.035235	-0.363659
F	-1.004662	1.910646	2.297629
F	-0.851930	-1.319351	-2.360231
F	-0.403317	-3.903167	-2.799284
F	1.622101	-4.301134	1.439064
F	1.234192	-1.725428	1.899854
C	-1.733012	0.293206	0.166589
C	-2.650963	0.826436	-0.729343
C	-4.024500	0.737753	-0.542530
C	-4.553230	0.092973	0.552686
C	-3.667426	-0.458607	1.456235
C	-2.302330	-0.361094	1.257433
F	-2.251894	1.476348	-1.854218
F	-1.501884	-0.923862	2.187072
F	-4.137275	-1.098791	2.545497
F	-4.849881	1.289970	-1.455950
O	0.319778	0.878819	-1.381010
C	0.374988	2.082826	-1.711333
C	-0.014499	3.261021	-0.894049
C	1.195518	4.082109	-0.446301
O	0.825518	2.380200	-2.897734
C	1.241318	1.291785	-3.770277
H	1.868263	3.491752	0.168118
H	-0.665108	3.874109	-1.516016
H	-0.593310	2.923687	-0.043740
H	2.030316	0.722270	-3.292208
H	0.850084	4.926981	0.143527
H	1.598413	1.778708	-4.667833
H	1.748591	4.463483	-1.300474
H	0.391675	0.652493	-3.982397
H	4.125896	1.463437	1.900125
H	3.116955	2.710783	3.784651
H	0.628439	2.892956	3.926651
H	0.791269	-5.207159	-0.866729
H	-5.620012	0.021667	0.699403

A2

57

B	-0.089473	0.089944	0.284056
C	0.718789	-0.225766	1.629656
C	0.314520	-1.277879	2.451547
C	0.956857	-1.690930	3.598399
C	2.078720	-0.988337	4.004645
C	2.507086	0.109413	3.277090
C	1.823778	0.474324	2.125833
C	-1.630642	0.495082	0.437739
C	-2.531416	0.318953	-0.607418
C	-3.875806	0.625092	-0.503042
C	-4.382329	1.163845	0.662152
C	-3.509625	1.377990	1.707646
C	-2.163850	1.056248	1.594217
C	0.720539	0.705734	-0.950990
C	0.370218	1.885900	-1.599029
C	1.105640	2.393718	-2.659315
C	2.230710	1.742154	-3.120230
C	2.604142	0.572570	-2.490992
C	1.868953	0.082309	-1.427262
F	-1.381204	1.326682	2.657173
F	-2.095375	-0.176290	-1.788350
F	-0.814751	-1.960923	2.108575
Br	2.432480	2.103866	1.305850
F	2.308526	-1.052433	-0.837599
F	-0.686503	2.612938	-1.184528
F	-4.693059	0.406681	-1.552067
F	0.718987	3.545518	-3.240628
F	3.699116	-0.093287	-2.907508
F	-3.965435	1.914022	2.856029
Si	-0.421350	-2.722992	-0.858990
C	0.744345	-3.807342	0.134436
C	1.245478	-5.027913	-0.637476
C	0.079847	-2.515371	-2.655288
C	-0.558507	-3.547918	-3.584893
C	-2.237194	-3.127504	-0.615395
C	-2.518254	-4.628758	-0.547044
H	-0.252671	-1.279406	-0.240191
H	0.572964	-2.530883	4.157962
H	2.608236	-1.280630	4.900548
H	3.354722	0.688736	3.609471
H	-5.428411	1.414327	0.751008
H	2.801972	2.137118	-3.946404
H	0.234249	-4.119477	1.045176
H	1.589623	-3.199651	0.455993
H	1.795796	-4.741055	-1.531805
H	0.428710	-5.677490	-0.948741
H	1.917437	-5.624549	-0.020881
H	1.166257	-2.568993	-2.723212
H	-0.194401	-1.510864	-2.975937
H	-1.645123	-3.483777	-3.570806
H	-0.286004	-4.566353	-3.312614
H	-0.237269	-3.390332	-4.614231
H	-2.807140	-2.674179	-1.425855
H	-2.576673	-2.645038	0.300865
H	-3.582231	-4.815796	-0.403616
H	-1.991402	-5.099840	0.281030
H	-2.219456	-5.141672	-1.459921

A3

71

C	1.812457	-0.651065	-1.425843
C	1.744649	-0.154840	-0.128140
C	2.939586	-0.187704	0.584148

C	4.105588	-0.718199	0.049023
C	4.132081	-1.229021	-1.231174
C	2.964791	-1.191560	-1.966372
B	0.375859	0.423703	0.469811
C	-0.363397	1.581014	-0.353164
C	-1.723509	1.814423	-0.180031
C	-2.410838	2.798191	-0.867223
C	-1.751585	3.622728	-1.755371
C	-0.398049	3.427493	-1.935961
C	0.277821	2.430380	-1.249022
F	-2.425896	1.061244	0.696192
F	-3.732777	2.957389	-0.658529
F	0.281055	4.222663	-2.785282
F	1.607823	2.342848	-1.451432
F	3.015888	0.314427	1.832446
F	5.229086	-0.726083	0.792257
F	2.949169	-1.673207	-3.225048
F	0.712687	-0.615616	-2.212466
C	0.223065	0.424674	2.064579
C	0.085730	1.544372	2.891803
C	-0.093775	1.464704	4.265449
C	-0.118707	0.223042	4.878267
C	0.067894	-0.922810	4.123822
C	0.248749	-0.781413	2.765086
Br	0.254701	3.331597	2.200969
F	0.479119	-1.926071	2.061382
Si	-1.523744	-1.839018	-0.289643
C	-2.514786	-2.180656	1.268009
C	-3.947117	-2.637736	0.994092
C	-0.402300	-3.249120	-0.813361
C	-1.057992	-4.624204	-0.680959
C	-2.521982	-1.092131	-1.691556
C	-3.055248	-2.137923	-2.671534
O	-4.711210	-5.282176	-1.522395
C	-5.798410	-5.815962	-1.609062
O	-5.966598	-7.153112	-1.598554
C	-4.766672	-7.938623	-1.466795
C	-7.119001	-5.106299	-1.728066
C	-6.996648	-3.602117	-1.855031
H	-7.653170	-5.534862	-2.577296
H	-7.711287	-5.378547	-0.851762
H	-4.092887	-7.749356	-2.298335
H	-4.259406	-7.707908	-0.533559
H	-5.093096	-8.971967	-1.473522
H	-6.438724	-3.323357	-2.746404
H	-7.984878	-3.151205	-1.919927
H	-6.482323	-3.171719	-0.999279
H	-0.521471	-0.684459	0.124822
H	-0.195722	2.365551	4.850844
H	-0.261588	0.152832	5.947454
H	0.088199	-1.905885	4.570087
H	-2.277570	4.398131	-2.291055
H	5.038911	-1.641140	-1.646753
H	0.504275	-3.209143	-0.209860
H	-0.089063	-3.079438	-1.843077
H	-1.951387	-4.714277	-1.294490
H	-1.347397	-4.834616	0.347421
H	-0.368062	-5.409147	-0.990864
H	-1.895235	-0.373756	-2.219036
H	-3.345870	-0.519741	-1.265843
H	-2.244731	-2.671031	-3.165662
H	-3.654428	-1.664929	-3.449662
H	-3.681783	-2.879592	-2.181936
H	-2.523912	-1.276749	1.876326
H	-1.982677	-2.930387	1.853105
H	-4.473394	-2.834198	1.928377
H	-3.983284	-3.548366	0.400354
H	-4.513056	-1.877592	0.458133

[A3]‡

71

C	0.921580	-1.065699	-2.351112
C	1.377206	-1.220246	-1.047183
C	2.317089	-2.230442	-0.876923
C	2.725997	-3.054077	-1.917696
C	2.229450	-2.892013	-3.193415
C	1.316169	-1.878322	-3.398323
B	0.804181	-0.267457	0.135741
C	1.104740	1.313970	-0.055278
C	0.373656	2.247038	0.671022
C	0.546636	3.613379	0.546028
C	1.490834	4.124427	-0.319931
C	2.243741	3.225623	-1.046394
C	2.055716	1.857851	-0.911097
F	-0.549318	1.822804	1.567683
F	-0.209005	4.454941	1.286112
F	3.188524	3.685217	-1.894105
F	2.882346	1.063144	-1.625394
F	2.893698	-2.451063	0.324853
F	3.634397	-4.023837	-1.677505
F	0.813428	-1.669436	-4.635561
F	0.059211	-0.061308	-2.640963
C	1.042424	-0.847686	1.633875
C	1.852502	-0.344920	2.655771
C	1.911369	-0.881977	3.936309
C	1.163137	-2.006006	4.239712
C	0.389579	-2.597849	3.255266
C	0.373244	-2.014413	2.006328
Br	3.087241	1.107922	2.357896
F	-0.358863	-2.661751	1.053245
Si	-2.384531	-0.328593	-0.256756
C	-2.591835	-0.457960	1.605709
C	-3.307706	-1.715119	2.089642
C	-2.281577	-1.916292	-1.254638
C	-2.704811	-1.826739	-2.716623
C	-2.280604	1.311950	-1.166425
C	-3.024778	2.507118	-0.584338
O	-4.477435	-0.349686	-0.596137
C	-5.559038	0.063445	-0.163926
O	-6.671831	-0.144882	-0.839383
C	-6.574341	-0.863923	-2.092960
C	-5.761206	0.786860	1.128512
C	-7.073802	1.530779	1.285256
H	-5.652892	0.032733	1.911400
H	-4.915470	1.455952	1.255135
H	-5.953410	-0.310268	-2.789710
H	-6.154978	-1.850755	-1.925437
H	-7.591070	-0.934131	-2.457392
H	-7.927186	0.860160	1.239456
H	-7.090130	2.031891	2.250275
H	-7.195636	2.286753	0.512798
H	-0.474883	-0.330461	-0.024069
H	2.553749	-0.436351	4.680390
H	1.203511	-2.431171	5.232948
H	-0.178334	-3.498554	3.436417
H	1.638715	5.188429	-0.423038
H	2.549887	-3.529437	-4.003113
H	-2.900392	-2.647274	-0.730221
H	-1.264741	-2.292462	-1.186558
H	-3.733709	-1.493100	-2.819699
H	-2.624999	-2.802448	-3.196202
H	-2.077049	-1.139994	-3.277439
H	-2.632700	1.111736	-2.180045
H	-1.228047	1.555540	-1.273165
H	-2.752142	3.417811	-1.116884
H	-2.789698	2.668772	0.465376
H	-4.103446	2.399630	-0.676136
H	-3.108743	0.437786	1.947076
H	-1.608738	-0.403049	2.062889
H	-3.459407	-1.678559	3.168275
H	-2.725527	-2.606602	1.873738
H	-4.283513	-1.850229	1.626242

A4

71

C	-1.776714	-0.414301	3.134291
C	-1.728016	0.498417	2.084855
C	-2.416178	1.682570	2.324457
C	-3.120149	1.921107	3.496023
C	-3.161518	0.985964	4.509105
C	-2.470691	-0.191380	4.311100
B	-0.925893	0.065802	0.724027
C	-1.173017	1.025558	-0.575558
C	-2.470198	1.227656	-1.039142

C	-2.780036	1.979964	-2.156116
C	-1.783204	2.568280	-2.907483
C	-0.483548	2.369117	-2.495648
C	-0.191098	1.613893	-1.365984
F	-3.515583	0.668697	-0.374704
F	-4.073029	2.125289	-2.530695
F	0.527951	2.916222	-3.207221
F	1.122386	1.475676	-1.074141
F	-2.407852	2.687208	1.418927
F	-1.120048	-1.599108	3.031842
C	0.639796	-0.231280	1.069772
C	1.249459	-1.428878	0.707050
C	2.543099	-1.803690	1.016018
C	3.326109	-0.923918	1.744495
C	2.803153	0.300510	2.127599
C	1.497055	0.613178	1.779293
F	0.535193	-2.343797	-0.026464
Br	0.915485	2.380522	2.278720
H	3.405627	1.007163	2.678144
H	2.920392	-2.761144	0.686381
O	-2.696593	-3.612285	1.530215
C	-1.564534	-4.439427	1.933992
C	-2.852835	-3.326584	0.285438
C	-4.014922	-2.483651	-0.073898
C	-4.782720	-1.885674	1.086462
O	-2.049370	-3.823392	-0.560070
Si	-1.837588	-3.701987	-2.328089
C	-1.465926	-1.924855	-2.763136
C	-2.464470	-1.244907	-3.695821
C	-0.381782	-4.844257	-2.541550
C	-0.031238	-5.067139	-4.012241
C	-3.407632	-4.384136	-3.076895
C	-3.822313	-5.757382	-2.556305
H	-3.642070	-1.704602	-0.736578
H	-4.659032	-3.114063	-0.693042
H	-1.668451	-5.424952	1.492912
H	-0.645397	-3.965622	1.613701
H	-1.628777	-4.480557	3.011884
H	-4.144978	-1.244271	1.686360
H	-5.597424	-1.280394	0.698911
H	-5.203846	-2.655241	1.728069
H	-1.438232	-0.998430	0.415356
F	-3.767423	3.098021	3.655118
H	-3.703882	1.173267	5.423019
F	-2.475349	-1.141141	5.277216
H	-2.011279	3.155343	-3.783694
H	4.342773	-1.184452	2.004961
H	-3.227892	-4.432263	-4.154450
H	-4.218596	-3.664238	-2.962252
H	-4.038862	-5.736081	-1.488554
H	-3.040962	-6.499350	-2.713406
H	-4.718371	-6.115873	-3.060967
H	-0.471677	-1.920637	-3.215188
H	-1.352457	-1.352844	-1.842861
H	-2.586049	-1.789925	-4.631145
H	-2.130623	-0.239477	-3.942005
H	-3.449225	-1.153212	-3.240524
H	-0.592876	-5.796880	-2.052870
H	0.467202	-4.410461	-2.010719
H	0.850091	-5.698715	-4.113564
H	0.185350	-4.129364	-4.523535
H	-0.842703	-5.554234	-4.551732

[A4]‡

71

C	1.255236	0.338317	-2.943636
C	0.161758	0.430192	-2.074232
C	-1.046591	0.606010	-2.738691
C	-1.197908	0.736838	-4.105259
C	-0.069746	0.674478	-4.903727
C	1.168796	0.461575	-4.321915
B	0.341368	0.357615	-0.466284
C	0.472614	-1.104480	0.185811
C	0.379773	-2.286633	-0.554465
C	0.547462	-3.542694	0.010917
C	0.802674	-3.689939	1.356246
C	0.873568	-2.545094	2.121196
C	0.692454	-1.297888	1.551756
F	0.123283	-2.270042	-1.877104
F	0.450589	-4.636278	-0.771548
F	1.096791	-2.642922	3.447287
F	0.758532	-0.245530	2.390783
F	-2.191027	0.642036	-2.006811
Br	3.002601	-0.087680	-2.265600
C	1.324935	1.540176	0.033379
C	2.535897	1.381235	0.707046
C	3.339740	2.455080	1.059951
C	2.985474	3.749539	0.744237
C	1.808436	3.938159	0.052143
C	1.017256	2.858221	-0.299040
F	3.015208	0.159453	1.007385
F	-0.113198	3.134252	-0.991233
F	1.422350	5.185237	-0.288639
F	4.499604	2.224833	1.707570
Si	-3.244503	-1.409272	1.531428
C	-3.242712	-1.938990	-0.265121
C	-3.662942	-3.388648	-0.495854
C	-2.598146	-2.719506	2.704943
C	-3.628929	-3.755564	3.148203
C	-4.938725	-0.805757	2.059955
C	-5.028536	-0.237366	3.472187
O	-2.072092	-0.152834	1.767538
C	-1.963834	1.013232	1.150191
C	-1.312508	2.105010	1.960934
C	-2.117025	2.446110	3.209501
O	-3.085145	1.304432	0.458225
C	-3.264834	2.607616	-0.114651
H	-0.322591	1.766360	2.238068
H	-1.179896	2.983266	1.337575
H	-2.428000	2.870320	-0.749924
H	-4.166404	2.535321	-0.711180
H	-3.399673	3.354370	0.663806
H	-1.621316	3.250119	3.749519
H	-3.126855	2.775532	2.972941
H	-2.187071	1.587404	3.871684
H	-0.923437	0.783637	0.149091
H	-2.184588	0.877916	-4.521348
H	-0.153774	0.775288	-5.976807
H	2.056233	0.378420	-4.930577
H	0.929283	-4.666441	1.798007
H	3.613987	4.583001	1.018217
H	-5.610565	-1.663587	1.959074
H	-5.288931	-0.073171	1.332267
H	-4.680203	-0.949331	4.219416
H	-6.053332	0.029521	3.731300
H	-4.422442	0.659419	3.576370
H	-2.245455	-1.778259	-0.669100
H	-3.893571	-1.263562	-0.820948
H	-2.984179	-4.084942	-0.005661
H	-3.662468	-3.631100	-1.558370
H	-4.665916	-3.589008	-0.118875
H	-2.179758	-2.211961	3.576418
H	-1.756708	-3.219681	2.225920
H	-3.181630	-4.490779	3.817210
H	-4.047630	-4.300912	2.304317
H	-4.460437	-3.297777	3.681172

[B4]‡

108

C	-1.599818	3.034351	-0.781102
C	-1.291576	2.213352	0.296527
C	-0.453076	2.793950	1.241297
C	0.050933	4.077375	1.126476
C	-0.257135	4.861180	0.033857
C	-1.090036	4.317447	-0.920703
B	-1.784131	0.670449	0.491389
C	-2.232160	-0.039025	-0.905050
C	-1.254341	-0.388465	-1.825005
C	-1.515695	-1.104550	-2.978633
C	-2.804983	-1.482270	-3.294097
C	-3.806710	-1.116608	-2.418429
C	-3.522997	-0.402048	-1.262980
F	0.034572	-0.014266	-1.602572
F	-0.498934	-1.438278	-3.814255

F	-5.085864	-1.453778	-2.698218
F	-4.575643	-0.064013	-0.483100
F	-0.108369	2.099937	2.356715
F	0.861126	4.570323	2.094304
F	-1.418944	5.051877	-2.009158
F	-2.442856	2.612712	-1.754866
C	-2.844148	0.422101	1.700837
C	-3.607207	1.416348	2.313175
C	-4.464439	1.224846	3.381129
C	-4.619036	-0.054077	3.888365
C	-3.921699	-1.105515	3.315713
C	-3.067538	-0.839176	2.256837
F	-3.541901	2.689942	1.831567
Br	-2.181858	-2.397803	1.517259
C	0.571308	-4.447364	3.910710
O	0.274392	-3.062292	4.186704
C	0.752415	-2.157907	3.347282
O	1.477338	-2.498645	2.408006
C	0.343842	-0.759710	3.656231
C	-0.511216	-0.548774	4.887965
C	3.840706	-3.161509	1.241418
Si	2.582984	-1.922358	0.584527
C	1.175694	-2.673548	-0.392960
C	1.097558	-4.195653	-0.366997
C	4.675708	-2.778091	2.457799
C	2.465677	-0.103812	1.000157
C	3.447448	0.448175	2.026869
O	3.801293	-1.482098	-1.095725
C	3.314159	-0.843826	-2.329503
C	3.728967	-1.658130	-3.535721
C	3.259033	-3.100058	-3.508151
C	5.238089	-1.456168	-0.960805
O	3.815848	0.441338	-2.385455
Si	3.076771	1.851993	-2.982627
C	4.501293	2.786114	-3.768470
C	5.101752	2.110622	-4.997102
C	2.368371	2.827167	-1.549296
C	3.409098	3.283276	-0.530960
C	1.761942	1.407788	-4.244632
C	0.968980	2.622219	-4.726929
H	-3.023491	-2.035901	-4.194176
H	-4.041249	-2.112775	3.685352
H	-5.286996	-0.235470	4.719118
H	-5.006994	2.066236	3.787280
H	0.137460	5.860158	-0.069452
H	3.316005	-1.145785	-4.404155
H	4.812643	-1.597469	-3.639141
H	2.235478	-0.854431	-2.201075
H	5.672298	-2.348601	-1.404494
H	5.627500	-0.565403	-1.438533
H	5.474379	-1.417788	0.093575
H	3.584217	-3.623798	-4.404909
H	3.656004	-3.634283	-2.647100
H	2.173016	-3.163102	-3.466837
H	1.078895	0.672952	-3.821833
H	2.241340	0.917446	-5.094466
H	0.405668	3.079216	-3.915261
H	0.252569	2.344919	-5.499938
H	1.617990	3.390811	-5.147670
H	5.271479	2.928922	-3.006858
H	4.151531	3.790454	-4.021141
H	5.953622	2.667471	-5.388530
H	4.373833	2.024731	-5.803103
H	5.449175	1.103881	-4.765492
H	1.588407	2.239096	-1.063901
H	1.849868	3.696891	-1.958101
H	3.992261	2.447727	-0.146376
H	2.943880	3.773979	0.322646
H	4.111578	3.990590	-0.970116
H	1.257019	-0.168338	3.707144
H	-0.174383	-0.401187	2.766364
H	1.644640	-4.606315	3.871950
H	0.130749	-5.002013	4.729599
H	0.119780	-4.738772	2.967169
H	0.002290	-0.856367	5.796470
H	-0.753870	0.507527	4.975437
H	-1.445950	-1.096924	4.820284
H	-0.783017	0.067353	0.830211
H	1.216554	-2.334701	-1.422534
H	0.261405	-2.243679	0.012580
H	0.993878	-4.576137	0.646430
H	1.982436	-4.656396	-0.803859
H	0.234785	-4.541340	-0.935766
H	1.441306	0.080246	1.313108
H	2.577864	0.439491	0.065814
H	3.227724	1.493038	2.240370
H	4.474906	0.398326	1.670280
H	3.406823	-0.093434	2.969435
H	4.482081	-3.485880	0.424246
H	3.236462	-4.032987	1.491205
H	4.044037	-2.501316	3.298969
H	5.338123	-1.937290	2.262122
H	5.297904	-3.614272	2.776555

B5

108

C	3.863956	0.001895	1.360364
C	2.645614	0.275929	0.751737
C	2.102729	1.509221	1.083244
C	2.690428	2.390110	1.973477
C	3.889041	2.080041	2.581706
C	4.467932	0.869121	2.259790
B	1.821107	-0.727055	-0.240411
C	2.172373	-2.291161	0.058698
C	1.761532	-2.862750	1.257957
C	2.015664	-4.180717	1.597223
C	2.689758	-5.014247	0.727998
C	3.108976	-4.477713	-0.471528
C	2.867061	-3.147967	-0.784597
F	1.089733	-2.112238	2.171013
F	1.611015	-4.659815	2.797465
F	3.784600	-5.258391	-1.346470
F	3.366742	-2.703713	-1.962546
F	0.935064	1.907638	0.511359
F	2.083840	3.572893	2.250251
F	5.651731	0.532475	2.820872
F	4.535290	-1.138443	1.075103
C	1.879987	-0.318837	-1.816047
C	2.842682	0.509615	-2.392997
C	2.875049	0.900928	-3.719047
C	1.894616	0.429223	-4.575445
C	0.911866	-0.417053	-4.087416
C	0.932719	-0.758697	-2.743649
F	3.865250	0.975977	-1.620328
Br	-0.464027	-1.976576	-2.192550
C	-1.719979	-3.980092	0.791703
O	-1.602977	-4.243789	2.202978
C	-2.079777	-3.298065	3.026584
O	-2.666747	-2.305784	2.637614
C	-1.770853	-3.617882	4.461754
C	-2.562994	-2.790507	5.453059
C	-1.478373	1.954948	2.690874
Si	-2.882502	1.290230	1.664875
C	-2.436111	0.110941	0.305614
C	-3.637865	-0.474239	-0.427572
C	-0.763642	0.837350	3.453709
C	-4.305403	0.722898	2.726612
C	-4.712385	1.652257	3.865803
O	-3.500811	2.771332	0.770720
C	-2.565853	3.485265	-0.217123
C	-2.629377	4.965875	0.054807
C	-2.234328	5.365318	1.463175
C	-4.912103	3.079296	0.588562
O	-2.968350	3.136999	-1.460006
Si	-2.021027	2.872201	-2.867337
C	-0.209896	2.715485	-2.449533
C	0.538332	3.976706	-2.031355
C	-2.362365	4.352652	-3.965503
C	-1.682377	4.252296	-5.329422
C	-2.667653	1.288213	-3.619166
C	-4.158946	1.286425	-3.938053
H	2.891547	-6.043455	0.981949
H	0.146339	-0.807790	-4.740621
H	1.897639	0.712493	-5.619186
H	3.668047	1.548162	-4.064935
H	4.359949	2.758168	3.276554

H	-1.949672	5.418333	-0.667939
H	-3.622064	5.330539	-0.207468
H	-1.602494	3.059704	0.060519
H	-5.140009	4.000640	1.112119
H	-5.108880	3.166130	-0.472366
H	-5.483365	2.261160	1.001134
H	-1.230351	5.020982	1.704649
H	-2.246691	6.447959	1.565952
H	-2.914517	4.952960	2.205718
H	-2.416577	0.464830	-2.949308
H	-2.089103	1.101111	-4.527051
H	-4.479179	0.327385	-4.345192
H	-4.413327	2.052002	-4.670662
H	-4.758828	1.477212	-3.048794
H	0.249821	2.308863	-3.353156
H	-0.069561	1.927926	-1.710667
H	0.191891	4.367450	-1.075792
H	0.432335	4.773114	-2.767249
H	1.602459	3.773277	-1.919265
H	-2.033974	5.256250	-3.446708
H	-3.442544	4.457645	-4.088362
H	-2.030351	3.383027	-5.886663
H	-0.600626	4.161830	-5.233792
H	-1.880626	5.131008	-5.943034
H	-1.920231	-4.685871	4.617713
H	-0.698165	-3.454685	4.589149
H	-1.193619	-3.066848	0.534198
H	-1.257033	-4.825955	0.298077
H	-2.764210	-3.893728	0.502443
H	-2.283091	-3.053863	6.471113
H	-2.378421	-1.727749	5.315958
H	-3.633063	-2.958061	5.342963
H	0.658254	-0.593986	0.079915
H	-1.854042	-0.687783	0.762834
H	-1.752257	0.592509	-0.392445
H	-4.265527	-1.057808	0.242532
H	-3.311125	-1.135160	-1.228157
H	-4.257005	0.299182	-0.879958
H	-3.949466	-0.228948	3.128901
H	-5.164242	0.450736	2.112316
H	-5.518971	1.216941	4.454217
H	-5.059307	2.621105	3.507889
H	-3.881332	1.837129	4.543565
H	-1.879171	2.696156	3.384139
H	-0.759941	2.479153	2.066826
H	-1.439422	0.294344	4.112486
H	0.036954	1.246533	4.067692
H	-0.315871	0.115803	2.775852

[C3]‡

71

C	1.214242	0.307943	-2.948685
C	0.133179	0.424455	-2.067492
C	-1.075306	0.628756	-2.724621
C	-1.238407	0.755737	-4.090516
C	-0.119898	0.663295	-4.899707
C	1.118722	0.426762	-4.327245
B	0.294479	0.367196	-0.448640
C	0.479949	-1.110008	0.192588
C	0.395916	-2.296383	-0.538786
C	0.564109	-3.549660	0.033762
C	0.810895	-3.691298	1.381351
C	0.875252	-2.540651	2.138443
C	0.695881	-1.297959	1.558142
F	0.138853	-2.290187	-1.863561
F	0.476334	-4.648604	-0.744592
F	1.092588	-2.629148	3.467861
F	0.758523	-0.237116	2.392642
F	-2.217058	0.702223	-1.985010
Br	2.963015	-0.146261	-2.289561
C	1.329210	1.536831	0.014803
C	2.551674	1.374015	0.663665
C	3.369143	2.443680	0.999987
C	3.017084	3.740466	0.691357
C	1.825531	3.933785	0.025636
C	1.021856	2.857012	-0.306336
F	3.026931	0.149460	0.966617
F	-0.124654	3.141261	-0.974210
F	1.437830	5.184240	-0.307237
F	4.540830	2.207605	1.626541
Si	-3.273778	-1.431257	1.529972
C	-3.187650	-1.934067	-0.268757
C	-3.523280	-3.401372	-0.526788
C	-2.637790	-2.715840	2.730804
C	-3.660920	-3.779444	3.123872
C	-4.978312	-0.822812	2.010664
C	-5.091023	-0.218385	3.406304
O	-2.120710	-0.138209	1.810256
C	-2.047595	1.031257	1.246149
C	-1.318110	2.101189	1.996937
C	-2.070072	2.478014	3.273470
O	-3.105025	1.322034	0.501906
C	-3.276558	2.638996	-0.058454
H	-0.334398	1.726444	2.243926
H	-1.182046	2.967755	1.360039
H	-2.407646	2.920507	-0.638178
H	-4.141458	2.558329	-0.704378
H	-3.467904	3.360646	0.730495
H	-1.520841	3.261301	3.790730
H	-3.072020	2.849865	3.068750
H	-2.152642	1.623554	3.939653
H	-0.861838	0.759970	0.117611
H	-2.225388	0.918618	-4.498281
H	-0.211559	0.760985	-5.972549
H	1.998993	0.324395	-4.943403
H	0.938761	-4.664967	1.828836
H	3.655887	4.570622	0.951015
H	-5.644033	-1.686403	1.920666
H	-5.320631	-0.112059	1.258163
H	-4.758122	-0.912377	4.177045
H	-6.119485	0.057062	3.639488
H	-4.484686	0.680276	3.497457
H	-2.187687	-1.715050	-0.635891
H	-3.855128	-1.286406	-0.837562
H	-2.824781	-4.066314	-0.021631
H	-3.473775	-3.629033	-1.591065
H	-4.526425	-3.658127	-0.186655
H	-2.279748	-2.194053	3.620511
H	-1.758268	-3.189135	2.295058
H	-3.224944	-4.504749	3.810452
H	-4.026340	-4.332625	2.260473
H	-4.527058	-3.344673	3.620271

X1

71

Si	0.956536	-6.169188	-1.116757
H	0.538017	-4.732225	-1.021141
C	-0.084761	-6.999148	-2.455038
C	-1.559543	-7.189483	-2.114535
C	0.663455	-6.961302	0.569525
C	1.338348	-6.229452	1.725644
C	2.775843	-6.211152	-1.592265
C	3.077001	-5.549305	-2.934503
H	0.370421	-7.968266	-2.676704
H	0.013186	-6.415876	-3.373383
H	-2.108222	-7.646110	-2.938762
H	-2.049555	-6.240955	-1.891629
H	-1.691099	-7.830544	-1.243954
H	1.014627	-7.995711	0.522897
H	-0.411867	-7.027824	0.749994
H	2.418832	-6.185247	1.598669
H	1.145433	-6.717003	2.681609
H	0.982843	-5.202707	1.806709
H	3.094434	-7.257097	-1.609386
H	3.362834	-5.738259	-0.803727
H	2.573621	-6.060081	-3.754752
H	4.144705	-5.554897	-3.153845
H	2.750528	-4.509676	-2.948200
C	2.389550	-1.860328	-0.249510
C	1.457732	-1.383586	0.667102
C	1.578542	-1.908483	1.947010
C	2.591699	-2.781426	2.318894
C	3.525272	-3.185306	1.381294
C	3.422966	-2.719714	0.081259

B	0.299184	-0.354186	0.160549
O	-0.525510	-1.209191	-0.880507
C	-1.163908	-2.262199	-0.654081
O	-1.153395	-2.737166	0.557054
C	-1.937866	-3.898081	0.920326
F	0.687885	-1.608034	2.913680
F	2.660789	-3.257289	3.571393
F	4.321469	-3.104573	-0.836569
F	2.330052	-1.470822	-1.541112
C	-0.673344	0.291716	1.305739
C	-0.078674	1.080796	2.285996
C	-0.781855	1.763400	3.262523
C	-2.165563	1.689904	3.276375
C	-2.807046	0.933842	2.312733
C	-2.060132	0.266679	1.350478
F	1.263806	1.199718	2.319332
F	-2.790522	-0.424528	0.440331
C	0.837263	0.918105	-0.719049
C	-0.050235	1.628030	-1.522675
C	0.302802	2.747391	-2.258837
C	1.599816	3.226027	-2.183616
C	2.514254	2.570567	-1.378104
C	2.121473	1.447380	-0.663115
F	-1.344521	1.249891	-1.608532
F	-0.598368	3.380540	-3.025379
F	3.771737	3.029382	-1.294386
F	3.078492	0.884695	0.100640
C	-1.920519	-2.906665	-1.756156
C	-1.656023	-2.317880	-3.126324
H	-2.255304	-2.842351	-3.866233
H	-1.700580	-3.973069	-1.737175
H	-2.978692	-2.824357	-1.489520
H	-1.621772	-4.761247	0.345850
H	-0.608846	-2.421642	-3.398920
H	-2.992706	-3.692248	0.769293
H	-1.912183	-1.263312	-3.161727
H	-1.730124	-4.052461	1.970677
F	-0.144316	2.493721	4.187990
F	-2.870325	2.341733	4.208082
F	-4.147274	0.859425	2.308806
F	1.963054	4.309873	-2.878792
F	4.504640	-4.031062	1.721146

[X1]‡

71

Si	0.405702	-2.249053	-1.422704
H	-1.193907	-1.865635	-0.285360
C	0.585350	-1.206492	-2.940444
C	-0.660275	-1.016123	-3.797298
C	-0.645731	-3.763939	-1.765354
C	-0.785935	-4.792755	-0.653140
C	1.908195	-2.630294	-0.415273
C	3.067082	-1.652981	-0.550309
H	1.348372	-1.760626	-3.505181
H	1.051377	-0.261975	-2.683474
H	-0.426292	-0.428766	-4.682810
H	-1.443308	-0.492741	-3.255271
H	-1.066763	-1.967632	-4.133740
H	-0.130843	-4.210596	-2.627548
H	-1.621276	-3.459558	-2.143769
H	0.181220	-5.134611	-0.290410
H	-1.333849	-5.665949	-1.003626
H	-1.329917	-4.389012	0.197360
H	2.201556	-3.632787	-0.748974
H	1.610585	-2.747209	0.625072
H	3.415979	-1.576708	-1.578804
H	3.908036	-1.974096	0.061717
H	2.784924	-0.660539	-0.220614
C	2.110055	0.284353	2.367016
C	1.552753	1.293685	1.596198
C	2.043338	2.565271	1.886518
C	3.010723	2.831227	2.838579
C	3.540383	1.784734	3.575447
C	3.087653	0.502806	3.331173
B	0.324247	1.170920	0.488849
O	-0.073895	-0.275585	0.145873
C	-1.183565	-1.036179	0.511421
O	-0.913848	-1.670694	1.718486
C	-1.954578	-2.395512	2.357930
F	1.594856	3.636082	1.192347
F	3.446643	4.083653	3.051799
F	3.588622	-0.524939	4.039536
F	1.731354	-1.000556	2.229811
C	-0.868290	2.089419	1.209110
C	-1.366210	3.310701	0.757974
C	-2.305128	4.066950	1.448417
C	-2.767046	3.638615	2.678466
C	-2.260718	2.463902	3.203995
C	-1.324724	1.742605	2.481088
F	-0.939223	3.878001	-0.387950
F	-0.848744	0.644836	3.101433
C	0.796945	1.728798	-1.001224
C	-0.143293	1.899688	-2.013179
C	0.152516	2.323950	-3.295978
C	1.472230	2.555675	-3.645424
C	2.455518	2.366040	-2.693484
C	2.106022	1.960581	-1.409588
F	-1.451181	1.647846	-1.780256
F	-0.816642	2.492934	-4.210196
F	3.741326	2.571139	-3.019038
F	3.154299	1.791908	-0.575755
C	-2.563054	-0.387728	0.376241
C	-3.585462	-1.267471	-0.324866
H	-4.536149	-0.744895	-0.407369
H	-2.922849	-0.094486	1.359132
H	-2.433414	0.524990	-0.187241
H	-2.320069	-3.213827	1.734272
H	-3.767430	-2.202293	0.201067
H	-2.792672	-1.758733	2.637216
H	-3.258747	-1.512863	-1.335357
H	-1.515016	-2.810308	3.259078
F	-2.753556	5.226373	0.941221
F	-3.671121	4.355754	3.356605
F	-2.670658	2.041756	4.411962
F	1.788237	2.948150	-4.885373
F	4.479278	2.012803	4.503972

X2

71

Si	0.611879	-4.127620	-1.440002
H	-1.560999	-4.534280	0.022904
C	0.509862	-3.440009	-3.192680
C	0.698832	-4.505467	-4.273472
C	-0.292745	-5.786255	-1.424585
C	-0.072311	-6.734799	-0.250816
C	2.406371	-4.611396	-1.156725
C	3.556711	-3.646343	-1.385587
H	1.291867	-2.692326	-3.301614
H	-0.428651	-2.922483	-3.367360
H	0.774434	-4.036378	-5.254187
H	-0.137690	-5.199910	-4.310873
H	1.607267	-5.088745	-4.127683
H	0.122047	-6.260348	-2.317195
H	-1.353095	-5.680060	-1.650793
H	0.985826	-6.930825	-0.092862
H	-0.556898	-7.693756	-0.432889
H	-0.466634	-6.348503	0.684481
H	2.475852	-5.415649	-1.899393
H	2.519008	-5.113543	-0.201390
H	3.445311	-3.083131	-2.310612
H	4.500976	-4.187255	-1.449009
H	3.647646	-2.933977	-0.574739
C	2.184377	-2.943357	1.874296
C	1.759249	-1.764535	1.285026
C	2.395790	-0.624197	1.774396
C	3.412355	-0.644254	2.711693
C	3.830165	-1.857313	3.234705
C	3.206029	-3.015036	2.811637
B	0.523565	-1.526827	0.235260
O	-0.163674	-3.016883	-0.236721
C	-1.560473	-3.488616	0.300748
O	-1.641014	-3.339039	1.647146
C	-1.409972	-4.490174	2.463573
F	2.033682	0.596315	1.323668

F	3.996416	0.492142	3.117629
F	3.569513	-4.198034	3.332713
F	1.580791	-4.122228	1.606888
C	-0.558577	-0.594749	1.080723
C	-1.153625	0.590491	0.634852
C	-2.032276	1.358147	1.388373
C	-2.321804	0.998135	2.689109
C	-1.695061	-0.114112	3.217360
C	-0.820325	-0.851281	2.435622
F	-0.886006	1.120777	-0.573227
F	-0.198834	-1.836059	3.108965
C	1.019117	-0.877765	-1.198054
C	0.100704	-0.624765	-2.217708
C	0.403036	-0.042604	-3.433892
C	1.710366	0.313773	-3.709498
C	2.673324	0.080343	-2.747673
C	2.320525	-0.492131	-1.530617
F	-1.191661	-0.962251	-2.063618
F	-0.551108	0.150053	-4.356193
F	3.948058	0.414535	-2.992731
F	3.359570	-0.640203	-0.689492
C	-2.700654	-2.750040	-0.362828
C	-3.067689	-3.266746	-1.740602
H	-3.888086	-2.682937	-2.152554
H	-3.549648	-2.897723	0.304345
H	-2.504046	-1.685474	-0.366034
H	-0.377906	-4.818878	2.416356
H	-3.394188	-4.305253	-1.693849
H	-2.077633	-5.300729	2.170783
H	-2.245111	-3.206117	-2.444443
H	-1.642288	-4.185864	3.477877
F	-2.578217	2.468546	0.870453
F	-3.161668	1.726253	3.430389
F	-1.919759	-0.457960	4.494997
F	2.035187	0.862352	-4.882770
F	4.813108	-1.904570	4.140702

X3

71

Si	0.462141	-6.841565	-1.863882
H	-0.233854	-5.651728	-2.453364
C	1.433600	-7.687452	-3.237928
C	0.569799	-8.211049	-4.382229
C	-0.860650	-7.985934	-1.158291
C	-1.689244	-7.367543	-0.035851
C	1.611736	-6.226427	-0.500249
C	2.720470	-5.296858	-0.983586
H	2.009863	-8.505451	-2.797084
H	2.171986	-6.981326	-3.623470
H	1.175703	-8.658101	-5.170839
H	-0.016594	-7.412059	-4.836790
H	-0.130544	-8.972687	-4.041301
H	-0.372825	-8.898186	-0.804752
H	-1.517153	-8.299714	-1.972500
H	-1.069856	-7.111130	0.823662
H	-2.465466	-8.046764	0.317735
H	-2.185139	-6.454164	-0.367207
H	2.043345	-7.098503	-0.001639
H	1.017823	-5.719329	0.263261
H	3.378360	-5.793036	-1.695386
H	3.341947	-4.949306	-0.158708
H	2.317255	-4.414567	-1.481288
C	2.655183	-1.258001	-0.237730
C	1.830269	-0.782657	0.777297
C	2.236238	-1.116966	2.062473
C	3.405044	-1.815286	2.334032
C	4.212519	-2.230419	1.289926
C	3.834644	-1.946249	-0.011971
B	0.452549	-0.005721	0.381129
O	-0.374496	-1.109714	-0.394886
C	-0.756695	-2.215502	0.050452
O	-0.464149	-2.515560	1.282931
C	-0.969320	-3.719457	1.908052
F	1.489646	-0.796750	3.138427
F	3.748049	-2.111516	3.597030
F	4.604458	-2.350832	-1.032299
F	2.325864	-1.050699	-1.530964

C	-0.419034	0.636037	1.605672
C	0.178590	1.636709	2.366057
C	-0.465885	2.332201	3.373559
C	-1.793585	2.047308	3.650172
C	-2.439239	1.072965	2.911498
C	-1.752897	0.401403	1.908045
F	1.466645	1.963707	2.138871
F	-2.486441	-0.516785	1.231077
C	0.621722	1.196582	-0.715347
C	-0.489745	1.657741	-1.414673
C	-0.449319	2.704229	-2.321745
C	0.747395	3.365655	-2.539699
C	1.876633	2.961570	-1.849360
C	1.794561	1.903078	-0.955074
F	-1.700929	1.092437	-1.218382
F	-1.555331	3.094296	-2.973763
F	3.039210	3.599606	-2.049008
F	2.942072	1.590055	-0.321796
C	-1.510119	-3.140492	-0.830423
C	-1.747713	-2.622548	-2.232868
H	-2.290695	-3.370045	-2.805339
H	-0.961423	-4.084498	-0.855428
H	-2.452321	-3.371129	-0.327340
H	-0.742883	-4.593156	1.308242
H	-0.808538	-2.421781	-2.741090
H	-2.039919	-3.623318	2.060262
H	-2.330081	-1.705706	-2.223139
H	-0.457941	-3.772597	2.860109
F	0.174170	3.274897	4.079120
F	-2.441275	2.705864	4.617846
F	-3.727189	0.791100	3.163216
F	0.809881	4.384882	-3.404072
F	5.337612	-2.911959	1.533284

[X3]‡

71

Si	0.348248	-3.083696	-0.948109
H	-0.099069	-1.087160	-1.080641
C	-0.863100	-3.805223	-2.174136
C	-2.080911	-4.521260	-1.598907
C	0.351567	-3.963051	0.690159
C	1.262583	-3.439429	1.788786
C	2.015972	-2.682737	-1.677391
C	2.018837	-1.988211	-3.033926
H	-0.242045	-4.518192	-2.732434
H	-1.148836	-3.058494	-2.912186
H	-2.703212	-4.916040	-2.399960
H	-2.700424	-3.857650	-0.999164
H	-1.790697	-5.356741	-0.965483
H	0.637325	-4.988076	0.412810
H	-0.678197	-4.032755	1.038010
H	2.299244	-3.378444	1.464973
H	1.227705	-4.092055	2.659370
H	0.949941	-2.449264	2.103026
H	2.493693	-3.669747	-1.751789
H	2.615117	-2.133190	-0.956425
H	1.470039	-2.556364	-3.782911
H	3.038536	-1.865201	-3.393866
H	1.573538	-0.998260	-2.971985
C	2.195447	0.229495	0.628475
C	1.090015	0.732870	1.300403
C	1.045203	0.418702	2.651453
C	2.065836	-0.248248	3.315442
C	3.176401	-0.674161	2.609013
C	3.239058	-0.437344	1.246896
B	-0.002888	1.642224	0.480875
O	-0.757203	0.785087	-0.492465
C	-1.014626	-0.511414	-0.606425
O	-1.098755	-1.289154	0.588753
C	-2.207085	-1.059484	1.461581
F	-0.016700	0.754959	3.413817
F	1.972590	-0.517714	4.627178
F	4.302676	-0.867354	0.544667
F	2.322253	0.428642	-0.704894
C	-1.050907	2.496209	1.439433
C	-0.537404	3.382225	2.382312
C	-1.308645	4.230349	3.156637

C	-2.684318	4.232042	2.993501
C	-3.246917	3.383069	2.059694
C	-2.432493	2.548519	1.302527
F	0.795966	3.444830	2.586747
F	-3.094018	1.773368	0.414141
C	0.699440	2.848160	-0.430492
C	-0.058189	3.492671	-1.406886
C	0.412916	4.533887	-2.191967
C	1.697170	5.009033	-1.994292
C	2.480712	4.426959	-1.015150
C	1.971444	3.382480	-0.255006
F	-1.343520	3.143072	-1.628149
F	-0.369664	5.101600	-3.125301
F	3.727154	4.882290	-0.806457
F	2.817507	2.903879	0.682959
C	-2.177107	-0.715791	-1.565831
C	-1.869506	-0.240982	-2.972679
H	-2.706679	-0.445449	-3.637337
H	-2.450188	-1.767368	-1.559155
H	-3.024934	-0.162526	-1.169069
H	-3.159835	-1.184737	0.950477
H	-0.990692	-0.739809	-3.382341
H	-2.156247	-0.071249	1.907015
H	-1.678444	0.827836	-2.986853
H	-2.119993	-1.804406	2.246849
F	-0.744112	5.050142	4.057433
F	-3.455210	5.044021	3.728851
F	-4.579726	3.377504	1.886607
F	2.171538	6.018645	-2.736189
F	4.156824	-1.346337	3.225382

X4

71

Si	0.165756	-5.877171	-0.465713
H	0.909365	-2.809473	-1.568508
C	-0.856069	-6.735294	-1.791468
C	-2.356851	-6.830270	-1.530947
C	0.096399	-6.847406	1.140254
C	1.004638	-6.314240	2.244838
C	1.947378	-5.678449	-1.025219
C	2.200604	-5.388382	-2.501595
H	-0.432918	-7.738938	-1.900016
H	-0.678156	-6.249098	-2.752983
H	-2.859386	-7.421486	-2.296747
H	-2.822367	-5.845705	-1.528939
H	-2.573644	-7.295505	-0.569871
H	0.371597	-7.877112	0.894901
H	-0.935725	-6.901920	1.492847
H	2.047757	-6.293587	1.932411
H	0.947336	-6.928798	3.143179
H	0.737160	-5.299035	2.531698
H	2.463177	-6.601867	-0.748167
H	2.400837	-4.904783	-0.405115
H	1.829637	-6.191954	-3.135704
H	3.265276	-5.276812	-2.705454
H	1.717140	-4.471254	-2.835390
C	2.760287	-1.831213	-0.303810
C	1.680998	-1.533026	0.522197
C	1.709254	-2.165753	1.760245
C	2.766427	-2.955972	2.189063
C	3.833240	-3.191382	1.339582
C	3.829096	-2.625047	0.075644
B	0.467794	-0.567063	0.021052
O	-0.426888	-1.425327	-0.953123
C	-0.099269	-2.418244	-1.633168
O	-0.414465	-4.305682	-0.180642
C	-1.506900	-4.060520	0.703215
F	0.690794	-2.038716	2.632349
F	2.757203	-3.509965	3.408794
F	4.840413	-2.871130	-0.768665
F	2.807885	-1.341460	-1.565240
C	-0.522526	0.007621	1.191274
C	0.038585	0.784661	2.200536
C	-0.690012	1.405121	3.199486
C	-2.068788	1.271405	3.212968
C	-2.678080	0.516651	2.228118
C	-1.908252	-0.088886	1.241807
F	1.376617	0.951033	2.246673
F	-2.610133	-0.796510	0.329941
C	0.918681	0.756988	-0.843772
C	0.004435	1.405698	-1.671108
C	0.288337	2.565401	-2.375211
C	1.532638	3.155692	-2.239883
C	2.467143	2.567586	-1.406257
C	2.147088	1.401386	-0.725162
F	-1.249364	0.927289	-1.819626
F	-0.634613	3.129968	-3.168417
F	3.674540	3.132423	-1.262153
F	3.121453	0.917005	0.068676
C	-0.990930	-2.876316	-2.710078
C	-0.602865	-2.126535	-3.995669
H	-1.221159	-2.486804	-4.814055
H	-0.863615	-3.943940	-2.855655
H	-2.024605	-2.669119	-2.444967
H	-2.383743	-4.649336	0.437605
H	0.439130	-2.297996	-4.258551
H	-1.765736	-3.009522	0.637403
H	-0.760956	-1.057051	-3.887352
H	-1.229133	-4.281437	1.732318
F	-0.080522	2.129371	4.148861
F	-2.798456	1.862259	4.166003
F	-4.013082	0.379627	2.228596
F	1.826295	4.279101	-2.903292
F	4.845310	-3.973692	1.725858

X5

94

C	0.627288	-0.327698	-2.123622
C	0.675334	-0.263679	-0.735581
C	1.953600	-0.278563	-0.187918
C	3.103365	-0.395571	-0.955885
C	3.000117	-0.482683	-2.334339
C	1.746899	-0.454230	-2.925991
B	-0.664365	-0.127855	0.133014
C	-1.616149	1.121120	-0.182055
C	-2.961447	1.091010	0.166758
C	-3.846225	2.120117	-0.097404
C	-3.376247	3.271726	-0.709815
C	-2.036877	3.362961	-1.050770
C	-1.185536	2.301974	-0.776183
F	-3.464728	0.004014	0.789940
F	-5.139072	2.023388	0.241166
F	-1.575139	4.478972	-1.629301
F	0.107409	2.479181	-1.106896
F	2.139244	-0.162113	1.140394
F	4.312136	-0.410333	-0.379995
F	1.635335	-0.533021	-4.258633
F	-0.565139	-0.272017	-2.754632
C	-0.588548	-0.617622	1.655836
C	-0.833227	0.201915	2.751470
C	-0.765110	-0.250129	4.061486
C	-0.422207	-1.568537	4.313589
C	-0.153018	-2.417933	3.251552
C	-0.223024	-1.923041	1.961943
F	-1.139016	1.502701	2.586476
F	0.076476	-2.782017	0.964270
Si	-2.354051	-2.354023	-1.063476
C	-3.115296	-3.184635	0.441233
C	-4.636830	-3.305244	0.396672
C	-1.106005	-3.385266	-2.022758
C	-0.931512	-4.824806	-1.547110
C	-3.556365	-1.373872	-2.122305
C	-4.071875	-2.134275	-3.343020
O	-4.079321	-6.659026	-1.599797
C	-4.814186	-7.122230	-0.476943
C	-4.856659	-6.429913	-2.746448
O	-5.683643	-5.305803	-2.520511
Si	-7.179501	-4.974602	-3.215114
C	-6.951279	-4.576152	-5.043222
C	-8.150902	-3.928629	-5.731357
C	-3.909009	-6.197864	-3.905291
C	-3.025371	-7.391776	-4.217196
C	-7.784166	-3.500949	-2.216187
C	-9.277419	-3.197058	-2.311528

C	-8.337615	-6.447695	-3.043954
C	-8.598037	-6.906791	-1.612836
H	-1.450547	-1.198223	-0.428350
F	-1.013313	0.577012	5.084968
F	-0.351058	-2.018192	5.569261
F	0.183531	-3.693592	3.484431
F	-4.208119	4.284550	-0.966572
F	4.097413	-0.594411	-3.087213
H	-0.145179	-2.871411	-1.990863
H	-1.408793	-3.367457	-3.070681
H	-1.864952	-5.381157	-1.572817
H	-0.552101	-4.865879	-0.528565
H	-0.217666	-5.351417	-2.180480
H	-3.072420	-0.450067	-2.435717
H	-4.392330	-1.076312	-1.490502
H	-3.274661	-2.341114	-4.054649
H	-4.827299	-1.547906	-3.865946
H	-4.525835	-3.084842	-3.074066
H	-2.816545	-2.625603	1.328195
H	-2.660653	-4.169539	0.543945
H	-5.003570	-3.830249	1.278700
H	-4.976309	-3.857264	-0.475634
H	-5.117045	-2.329173	0.379631
H	-4.511766	-5.940437	-4.774464
H	-3.307348	-5.319893	-3.670552
H	-5.486206	-7.312727	-2.933784
H	-5.547602	-6.387221	-0.145970
H	-5.327284	-8.061421	-0.703505
H	-4.096167	-7.293386	0.319927
H	-3.622295	-8.279371	-4.430404
H	-2.404680	-7.195598	-5.090201
H	-2.368059	-7.626141	-3.383494
H	-7.203258	-2.624540	-2.512729
H	-7.513088	-3.689081	-1.175161
H	-9.880999	-4.041094	-1.980947
H	-9.583821	-2.963417	-3.329197
H	-9.548285	-2.343507	-1.689408
H	-7.943417	-7.275502	-3.639448
H	-9.280304	-6.175678	-3.526571
H	-7.682386	-7.243434	-1.131427
H	-9.308381	-7.733379	-1.576281
H	-9.004404	-6.102924	-1.000012
H	-6.692908	-5.505003	-5.559102
H	-6.075965	-3.930543	-5.141510
H	-8.362929	-2.943836	-5.318277
H	-9.055009	-4.527748	-5.623379
H	-7.976050	-3.798752	-6.799776

X6

94

Si	-3.000759	-4.904747	-2.044473
C	-4.023159	-4.771697	-0.483908
C	-3.935951	-3.410222	0.206131
C	-1.348148	-5.721334	-1.717223
C	-0.440401	-4.796580	-0.900677
C	-2.780743	-3.337882	-3.031895
C	-1.745978	-3.419162	-4.152546
O	-3.842106	-6.084750	-3.138230
C	-3.804903	-7.505845	-2.806856
C	-5.129399	-5.684245	-3.948470
O	-5.676379	-4.646274	-3.278694
Si	-7.358405	-4.325373	-3.085273
C	-7.981013	-3.754172	-4.754821
C	-9.385759	-3.154066	-4.756757
C	-4.699723	-5.367185	-5.350004
C	-3.953996	-6.479356	-6.061240
C	-7.361902	-2.980143	-1.779982
C	-8.726334	-2.721996	-1.141958
C	-8.210571	-5.887423	-2.509619
C	-7.715141	-6.451653	-1.182459
H	-0.868387	-5.984789	-2.661051
H	-1.484345	-6.652005	-1.166194
H	-0.865957	-4.577538	0.077402
H	-0.261197	-3.846650	-1.400951
H	0.529410	-5.262000	-0.732465
H	-2.478191	-2.574094	-2.310960
H	-3.747577	-3.010119	-3.407856
H	-0.747320	-3.606098	-3.763579
H	-1.706989	-2.485841	-4.712201
H	-1.966788	-4.213785	-4.862365
H	-3.694238	-5.558077	0.198907
H	-5.058633	-4.998528	-0.726511
H	-4.532000	-3.399794	1.117623
H	-4.306533	-2.613304	-0.435495
H	-2.914051	-3.156702	0.484144
H	-5.627709	-5.154632	-5.879193
H	-4.132419	-4.439351	-5.342684
H	-5.717703	-6.599748	-3.893649
H	-4.562554	-7.726198	-2.062871
H	-3.982420	-8.060876	-3.717041
H	-2.816694	-7.734644	-2.434361
H	-4.518319	-7.409753	-6.040320
H	-3.800195	-6.215985	-7.104626
H	-2.976639	-6.659491	-5.617193
H	-6.975687	-2.061926	-2.228887
H	-6.645338	-3.254278	-1.005001
H	-9.113583	-3.613355	-0.650940
H	-9.468214	-2.410440	-1.873899
H	-8.665652	-1.937070	-0.388292
H	-8.133746	-6.642899	-3.291661
H	-9.277966	-5.661311	-2.446022
H	-6.666433	-6.740059	-1.237033
H	-8.275623	-7.340976	-0.896188
H	-7.809443	-5.730028	-0.371702
H	-7.950293	-4.610185	-5.429522
H	-7.265156	-3.030284	-5.152270
H	-9.425878	-2.223026	-4.193734
H	-10.118201	-3.833929	-4.324668
H	-9.713925	-2.933831	-5.772054
C	-8.863459	-9.100384	-8.326121
C	-8.142006	-8.158641	-7.605106
C	-7.833172	-7.002246	-8.302658
C	-8.208269	-6.778162	-9.619984
C	-8.932628	-7.745858	-10.293867
C	-9.262006	-8.923194	-9.640616
B	-7.761085	-8.377475	-6.035728
C	-6.550390	-9.437643	-5.756757
C	-5.782665	-10.090170	-6.713062
C	-4.714790	-10.926025	-6.404899
C	-4.369705	-11.139961	-5.083247
C	-5.099808	-10.505707	-4.091118
C	-6.148170	-9.676010	-4.447045
F	-6.030472	-9.939863	-8.032121
F	-4.010050	-11.527904	-7.377640
F	-4.765047	-10.689723	-2.797997
F	-6.793522	-9.064672	-3.421878
F	-7.121554	-6.007329	-7.717830
F	-7.881686	-5.633903	-10.249043
F	-9.953125	-9.873374	-10.293584
F	-9.186780	-10.280598	-7.752848
C	-9.143243	-8.697078	-5.219823
C	-9.526280	-9.912000	-4.669471
C	-10.704219	-10.093324	-3.957462
C	-11.572663	-9.028418	-3.789409
C	-11.247555	-7.801111	-4.342361
C	-10.062461	-7.669630	-5.047229
F	-8.747408	-11.009000	-4.801283
F	-9.817913	-6.448288	-5.576857
H	-7.354524	-7.320984	-5.596063
F	-11.018389	-11.292105	-3.435329
F	-12.715724	-9.183701	-3.103732
F	-12.083423	-6.757165	-4.187570
F	-3.340637	-11.939393	-4.763900
F	-9.307361	-7.549036	-11.566877

[X6]‡

94

Si	-2.864264	-4.783130	-1.638857
C	-2.506229	-5.086430	0.190259
C	-1.723849	-3.983333	0.901101
C	-1.453352	-5.389639	-2.729778
C	-0.130334	-4.653253	-2.534562
C	-3.252461	-2.978818	-1.972178
C	-3.522953	-2.656042	-3.438349

O	-4.264820	-5.609161	-2.061323
C	-4.354839	-7.020587	-1.954725
C	-6.689483	-5.421843	-4.990136
O	-7.984390	-4.925907	-4.660203
Si	-8.430513	-4.616646	-3.070574
C	-10.097683	-3.758681	-3.221192
C	-11.011888	-3.882209	-2.005166
C	-6.047718	-4.578354	-6.068918
C	-4.713101	-5.148265	-6.518307
C	-7.137942	-3.491877	-2.287140
C	-7.597789	-2.680639	-1.078484
C	-8.556686	-6.267515	-2.162721
C	-8.417450	-6.222428	-0.644200
H	-1.778242	-5.303858	-3.769708
H	-1.309569	-6.458716	-2.553499
H	0.259048	-4.788204	-1.526197
H	-0.238705	-3.581055	-2.698006
H	0.635423	-5.009551	-3.224518
H	-2.421565	-2.373090	-1.603715
H	-4.113104	-2.700195	-1.359452
H	-2.653870	-2.867185	-4.060869
H	-3.776422	-1.604893	-3.580564
H	-4.349913	-3.245430	-3.826951
H	-1.979162	-6.038549	0.292243
H	-3.468969	-5.228078	0.688678
H	-1.539657	-4.233070	1.946673
H	-2.265306	-3.038517	0.884550
H	-0.754471	-3.805960	0.437062
H	-6.733953	-4.511189	-6.914596
H	-5.919576	-3.562637	-5.690393
H	-6.036955	-5.452547	-4.113197
H	-5.384730	-7.313106	-2.143246
H	-3.718108	-7.520446	-2.688546
H	-4.076674	-7.374830	-0.958970
H	-4.833878	-6.143060	-6.947244
H	-4.248526	-4.517855	-7.274834
H	-4.014508	-5.233764	-5.685725
H	-6.803953	-2.810315	-3.073739
H	-6.263366	-4.088030	-2.025462
H	-7.919825	-3.318491	-0.256953
H	-8.433861	-2.027713	-1.326735
H	-6.795816	-2.046180	-0.697716
H	-7.781498	-6.915129	-2.576881
H	-9.502549	-6.740852	-2.437642
H	-7.452685	-5.812161	-0.348837
H	-8.494673	-7.217941	-0.204607
H	-9.187189	-5.605306	-0.182944
H	-10.600271	-4.162803	-4.101353
H	-9.917756	-2.704954	-3.449089
H	-10.559021	-3.458576	-1.109796
H	-11.246234	-4.923306	-1.786107
H	-11.958967	-3.365063	-2.163424
C	-8.168366	-9.156528	-9.305747
C	-7.220613	-9.126826	-8.285500
C	-5.900538	-8.919493	-8.678365
C	-5.535775	-8.720583	-9.998609
C	-6.511384	-8.766257	-10.984207
C	-7.837107	-8.991174	-10.638574
B	-7.605482	-9.337847	-6.784619
C	-6.740217	-10.328650	-5.941054
C	-6.310235	-11.548989	-6.462664
C	-5.521460	-12.433284	-5.747012
C	-5.119571	-12.096701	-4.462381
C	-5.519925	-10.890075	-3.905407
C	-6.326693	-10.041589	-4.641427
F	-6.688176	-11.931116	-7.693575
F	-5.146879	-13.602789	-6.274118
F	-5.124518	-10.566252	-2.669873
F	-6.685026	-8.885396	-4.062608
F	-4.922854	-8.860178	-7.757183
F	-4.262141	-8.492829	-10.334418
F	-8.770870	-9.046610	-11.593157
F	-9.460917	-9.383202	-9.016569
C	-8.818978	-8.583749	-6.172094
C	-9.603597	-9.132994	-5.151347
C	-10.706237	-8.488198	-4.623760
C	-11.065765	-7.241309	-5.115885
C	-10.317389	-6.651128	-6.123155
C	-9.224403	-7.325932	-6.634314

F	-9.328313	-10.351148	-4.660624
F	-8.531356	-6.696429	-7.592544
H	-6.789663	-6.453097	-5.339210
F	-11.434747	-9.051423	-3.654896
F	-12.123637	-6.610408	-4.613413
F	-10.666604	-5.453513	-6.593543
F	-4.352801	-12.931173	-3.763528
F	-6.176508	-8.593550	-12.261688

[X7]‡

71

C	1.006074	1.609641	-1.757943
C	1.210750	1.692625	-0.384936
C	2.547546	1.804751	-0.018634
C	3.599070	1.752743	-0.925264
C	3.337115	1.620462	-2.276963
C	2.021438	1.554111	-2.698843
B	-0.114712	1.737149	0.619822
C	-0.759677	3.240622	0.293145
C	-1.980739	3.502513	-0.306845
C	-2.413503	4.776991	-0.652486
C	-1.605444	5.868347	-0.395566
C	-0.375406	5.662736	0.207786
C	0.009931	4.374185	0.533395
F	-2.857685	2.510699	-0.589461
F	-3.612605	4.960765	-1.235031
F	0.419130	6.713684	0.473200
F	1.213115	4.253412	1.139248
F	2.917012	1.974553	1.265951
F	4.871792	1.837459	-0.505476
F	1.745009	1.438676	-4.010459
F	-0.251411	1.583327	-2.265576
C	0.235599	1.556658	2.231553
C	-0.330939	2.295212	3.262267
C	-0.115928	2.030904	4.609671
C	0.675590	0.959239	4.978404
C	1.241384	0.172764	3.988729
C	0.998218	0.477571	2.661049
F	-1.160385	3.332709	3.012716
F	1.565424	-0.357915	1.759019
Si	0.330312	-3.070639	-0.420044
C	-0.077252	-3.100551	1.392689
C	1.013606	-3.789976	2.213142
C	0.386199	-4.767868	-1.196000
C	-0.735207	-5.712612	-0.773625
C	1.714881	-1.959292	-0.971474
C	2.128604	-2.093679	-2.435372
O	-1.208275	-2.451432	-1.142358
C	-1.442119	-1.434833	-1.837986
O	-0.972396	0.584834	0.240739
C	-2.036868	0.257407	1.106711
C	-2.732487	-1.310250	-2.512853
C	-3.232106	0.099704	-2.775854
H	-2.532186	-1.802823	-3.479703
H	-3.463272	-1.943865	-2.012063
H	-2.714263	-0.422440	0.588747
H	-1.690397	-0.244310	2.015240
H	-2.615550	1.130874	1.412100
H	-2.487348	0.699844	-3.289966
H	-4.120615	0.053550	-3.399571
H	-3.487835	0.599028	-1.849482
H	-0.660282	-0.697162	-1.995938
F	-0.686290	2.790628	5.559444
F	0.888936	0.681276	6.271652
F	2.008081	-0.878314	4.331526
F	-2.005283	7.105705	-0.722499
F	4.339980	1.559016	-3.163851
H	1.359413	-5.195302	-0.939077
H	0.401503	-4.651389	-2.281485
H	-1.715173	-5.311937	-1.028681
H	-0.725381	-5.892968	0.299723
H	-0.638968	-6.678779	-1.267153
H	2.563120	-2.191696	-0.322709
H	1.460220	-0.928979	-0.729131
H	2.443907	-3.107118	-2.676235
H	2.960647	-1.429775	-2.661739
H	1.320336	-1.834145	-3.118862

H	-1.031211	-3.614928	1.520824
H	-0.226685	-2.081197	1.739319
H	0.750310	-3.801470	3.269307
H	1.968277	-3.274988	2.126820
H	1.163349	-4.823740	1.903256

X8

71

C	-0.689548	0.239297	-2.028571
C	-0.505803	0.150712	-0.648894
C	0.830714	0.155635	-0.254707
C	1.892287	0.155066	-1.149659
C	1.647782	0.183033	-2.510855
C	0.338539	0.235848	-2.955719
B	-1.800857	0.223664	0.352524
C	-2.593743	1.603353	-0.033012
C	-3.895158	1.756744	-0.486004
C	-4.437255	2.981347	-0.855669
C	-3.667273	4.126545	-0.772668
C	-2.362232	4.027581	-0.317246
C	-1.864826	2.787899	0.041029
F	-4.734095	0.702714	-0.600557
F	-5.702818	3.061544	-1.293740
F	-1.606306	5.129781	-0.215731
F	-0.600033	2.766795	0.515536
F	1.185294	0.197550	1.041505
F	3.155957	0.141929	-0.705608
F	0.079303	0.279797	-4.269620
F	-1.936377	0.323771	-2.542469
C	-1.489853	0.069307	1.947284
C	-1.917044	0.931895	2.952263
C	-1.673756	0.710224	4.302546
C	-0.998780	-0.428839	4.702051
C	-0.574088	-1.331871	3.740708
C	-0.831892	-1.065834	2.409131
F	-2.624298	2.045068	2.678327
F	-0.401498	-1.988425	1.525867
Si	-1.252082	-4.516483	-0.484826
C	-1.129979	-5.192656	1.258283
C	-0.258496	-6.440913	1.376074
C	-1.816179	-5.872182	-1.653362
C	-3.153698	-6.505562	-1.282454
C	0.350946	-3.740999	-1.088983
C	0.522253	-3.600945	-2.599854
O	-2.505159	-3.359181	-0.406909
C	-2.592867	-2.145694	-1.015338
O	-2.757542	-1.072244	0.055221
C	-3.747628	-1.412427	1.059721
C	-3.765482	-2.060961	-1.964163
C	-3.501875	-2.836575	-3.243749
H	-4.650392	-2.458450	-1.469329
H	-3.957615	-1.019209	-2.194357
H	-4.618599	-1.818827	0.563753
H	-3.335642	-2.134511	1.754530
H	-4.018669	-0.501321	1.572493
H	-3.371260	-3.897689	-3.051853
H	-4.341060	-2.724995	-3.927009
H	-2.611256	-2.470586	-3.753276
H	-1.662141	-1.848330	-1.482438
F	-2.104187	1.582338	5.224290
F	-0.763797	-0.659220	5.996641
F	0.075564	-2.445069	4.110244
F	-4.174691	5.313337	-1.122876
F	2.659602	0.171720	-3.382406
H	-1.031234	-6.633600	-1.668915
H	-1.852437	-5.480797	-2.670983
H	-3.953384	-5.765832	-1.269037
H	-3.120563	-6.961541	-0.293547
H	-3.440122	-7.284274	-1.989470
H	1.154508	-4.367200	-0.691575
H	0.485467	-2.773990	-0.606321
H	0.473835	-4.565689	-3.101668
H	1.484895	-3.152739	-2.846119
H	-0.245732	-2.969847	-3.044575
H	-2.143367	-5.410822	1.603940
H	-0.753865	-4.410735	1.916745
H	-0.200939	-6.788643	2.407403
H	0.762623	-6.253257	1.043737
H	-0.647568	-7.264139	0.778287

[X8]‡

71

C	-0.563101	-0.178823	-1.877882
C	-0.336036	0.021966	-0.521471
C	1.003694	0.203759	-0.194340
C	2.037342	0.129635	-1.119962
C	1.752600	-0.103302	-2.453489
C	0.432550	-0.255381	-2.837531
B	-1.629507	0.151957	0.515000
C	-2.299275	1.604444	0.058564
C	-3.486147	1.765400	-0.641622
C	-3.943254	2.993957	-1.103616
C	-3.193655	4.133607	-0.880155
C	-1.992806	4.024061	-0.197262
C	-1.579218	2.779472	0.243285
F	-4.293616	0.715846	-0.932039
F	-5.106312	3.085618	-1.774557
F	-1.251268	5.122443	0.024160
F	-0.395235	2.742443	0.894737
F	1.391877	0.466588	1.068666
F	3.313819	0.289386	-0.736682
F	0.132632	-0.474485	-4.130357
F	-1.828755	-0.304645	-2.351798
C	-1.220734	0.079049	2.116585
C	-1.721815	0.893678	3.121176
C	-1.475094	0.688775	4.473517
C	-0.717187	-0.396343	4.872943
C	-0.219407	-1.258637	3.909618
C	-0.487790	-1.009303	2.575508
F	-2.519327	1.947884	2.837754
F	0.013508	-1.912279	1.699165
Si	-1.183921	-4.656213	-0.971955
C	-1.954193	-4.438206	0.705905
C	-1.175940	-5.178430	1.794850
C	-1.148380	-6.412860	-1.587981
C	-2.462940	-7.170959	-1.418545
C	0.383331	-3.698701	-1.259326
C	0.948625	-3.783116	-2.674281
O	-2.383752	-3.944768	-2.145891
C	-3.043098	-2.880624	-2.031601
O	-2.519030	-1.009495	0.281521
C	-3.592659	-1.189227	1.178812
C	-4.053934	-2.528570	-3.021803
C	-5.438325	-2.459246	-2.349761
H	-3.807208	-1.529686	-3.388620
H	-4.039400	-3.230266	-3.851613
H	-4.305236	-1.885835	0.733686
H	-3.264402	-1.610518	2.133375
H	-4.123294	-0.258593	1.387986
H	-5.448299	-1.742345	-1.534750
H	-6.165406	-2.141854	-3.091827
H	-5.736944	-3.432101	-1.967614
H	-2.867546	-2.217075	-1.170687
F	-1.982338	1.519551	5.399581
F	-0.474324	-0.617346	6.172306
F	0.508315	-2.326544	4.283828
F	-3.619049	5.326163	-1.320776
F	2.737734	-0.184372	-3.357815
H	-0.343857	-6.925497	-1.054391
H	-0.843664	-6.404695	-2.636213
H	-3.276173	-6.688453	-1.959838
H	-2.758282	-7.235000	-0.372417
H	-2.380209	-8.188835	-1.796924
H	1.112192	-4.075308	-0.536689
H	0.205272	-2.663366	-0.969778
H	1.186564	-4.808059	-2.953743
H	1.864017	-3.200309	-2.760865
H	0.247956	-3.395251	-3.412145
H	-2.988199	-4.787248	0.674638
H	-1.988249	-3.373243	0.925728
H	-1.634191	-5.019275	2.769593
H	-0.148364	-4.826708	1.864406
H	-1.149154	-6.252896	1.617729

X9

94

Si	-1.084179	-4.373579	-4.964218
H	-3.153536	-3.119588	-6.149055
C	-0.189023	-3.206199	-6.117428
C	0.884698	-3.914735	-6.945870
C	-1.892149	-5.781838	-5.897087
C	-2.421498	-6.942409	-5.057882
C	0.039436	-5.116129	-3.666587
C	1.199122	-4.319895	-3.078378
H	0.266872	-2.411792	-5.528231
H	-0.902002	-2.712815	-6.779730
H	1.439133	-3.198660	-7.550306
H	0.454618	-4.648258	-7.625152
H	1.607399	-4.433502	-6.317385
H	-1.100279	-6.151719	-6.554451
H	-2.659178	-5.409275	-6.576258
H	-1.629404	-7.402860	-4.471015
H	-2.848531	-7.718006	-5.692101
H	-3.196361	-6.629691	-4.361347
H	0.452024	-5.961562	-4.229421
H	-0.550501	-5.585575	-2.881189
H	1.749411	-3.774518	-3.842863
H	1.903344	-4.988500	-2.585910
H	0.875080	-3.601839	-2.332195
C	-1.240349	1.631192	-0.905872
C	-0.528319	0.875684	0.022400
C	0.852504	0.959388	-0.111391
C	1.485484	1.680580	-1.115879
C	0.729681	2.393339	-2.028380
C	-0.648800	2.373712	-1.915898
B	-1.355478	0.080424	1.224311
C	-1.872425	1.346465	2.178910
C	-3.173905	1.823257	2.257947
C	-3.529894	2.959705	2.975601
C	-2.560526	3.683296	3.644073
C	-1.244227	3.255701	3.584733
C	-0.937917	2.117611	2.860333
F	-4.201831	1.202909	1.635381
F	-4.811169	3.369678	3.022104
F	-0.288954	3.950455	4.228041
F	0.367259	1.761616	2.840594
F	1.684150	0.325247	0.741051
F	2.826711	1.700719	-1.208934
F	-1.396025	3.071595	-2.791343
F	-2.588680	1.690269	-0.861398
C	-0.455412	-1.043906	2.052114
C	-0.491177	-1.255911	3.422645
C	0.127223	-2.328652	4.054411
C	0.796726	-3.273115	3.298876
C	0.838153	-3.122447	1.922731
C	0.211455	-2.034555	1.341655
F	-1.164573	-0.420099	4.244973
F	0.282911	-1.978182	-0.010446
Si	-2.288429	-2.430904	-2.662696
C	-3.794896	-1.361243	-2.417080
C	-5.154103	-1.922710	-2.020527
C	-2.080541	-3.723820	-1.338989
C	-3.023227	-4.921200	-1.401857
C	-0.840784	-1.312946	-3.020092
C	-1.102654	-0.276580	-4.110685
O	-2.418719	-3.410856	-4.222473
C	-3.546085	-3.087447	-5.136266
O	-2.439466	-0.680134	0.585567
C	-3.209608	-1.518059	1.412443
C	-4.707666	-4.023843	-4.950678
C	-5.914557	-3.535195	-5.736643
H	-4.435229	-5.024503	-5.273343
H	-4.948729	-4.093508	-3.892108
H	-4.132243	-1.769144	0.889786
H	-2.690300	-2.452774	1.655990
H	-3.481689	-1.045304	2.358800
H	-5.696174	-3.466029	-6.801952
H	-6.749708	-4.220902	-5.614945
H	-6.238663	-2.551985	-5.397935
H	-3.822065	-2.060616	-4.927783
F	0.062846	-2.473728	5.389441
F	1.391388	-4.322259	3.886369
F	1.477249	-4.040106	1.170439
F	-2.887886	4.784100	4.337946
F	1.322742	3.091437	-3.008957
H	-2.231039	-3.175714	-0.409405
H	-1.045454	-4.053559	-1.300569
H	-2.886478	-5.502247	-2.311345
H	-4.068070	-4.621851	-1.367354
H	-2.849435	-5.590620	-0.560558
H	0.041721	-1.900914	-3.261708
H	-0.600639	-0.812490	-2.082640
H	-1.421028	-0.731020	-5.047944
H	-0.202065	0.297876	-4.321391
H	-1.871878	0.431281	-3.813960
H	-3.898460	-0.653459	-3.239404
H	-3.416622	-0.757758	-1.582471
H	-5.805444	-1.118219	-1.681889
H	-5.078533	-2.635951	-1.203884
H	-5.659753	-2.416926	-2.845857

[X9]‡

94

Si	0.477646	-2.313103	-3.018997
H	-1.578671	-1.119953	-4.134059
C	1.303648	-1.132500	-4.225992
C	2.363585	-1.804844	-5.098605
C	-0.267468	-3.774308	-3.940317
C	-0.741506	-4.931540	-3.063246
C	1.759060	-3.074962	-1.874474
C	2.873694	-2.243078	-1.253841
H	1.759075	-0.316746	-3.665779
H	0.549524	-0.664082	-4.860783
H	2.853917	-1.078296	-5.745804
H	1.933944	-2.571499	-5.741035
H	3.140809	-2.278377	-4.500132
H	0.529228	-4.130281	-4.598459
H	-1.058193	-3.447311	-4.615751
H	0.089871	-5.387987	-2.529632
H	-1.212012	-5.713739	-3.658497
H	-1.464470	-4.613156	-2.314091
H	2.213931	-3.797233	-2.562282
H	1.276523	-3.700760	-1.123720
H	3.328326	-1.569198	-1.978410
H	3.664496	-2.887178	-0.870830
H	2.528275	-1.641884	-0.419495
C	0.805971	3.528920	0.648530
C	1.414472	2.691505	1.580423
C	2.793928	2.847715	1.670454
C	3.521170	3.714548	0.863906
C	2.867489	4.500354	-0.067265
C	1.490308	4.407226	-0.173476
B	0.470747	1.796397	2.599586
C	-0.254737	3.007236	3.481186
C	-1.556579	3.470941	3.336686
C	-2.057015	4.575420	4.016163
C	-1.236630	5.288056	4.869851
C	0.076651	4.878713	5.033520
C	0.530554	3.771906	4.338859
F	-2.444487	2.865939	2.513891
F	-3.331528	4.966172	3.841626
F	0.889506	5.562588	5.855282
F	1.827335	3.444458	4.531928
F	3.527779	2.157434	2.565121
F	4.854742	3.806878	0.986992
F	0.838212	5.167742	-1.068808
F	-0.535574	3.508649	0.493639
C	1.288147	0.693245	3.521672
C	1.081094	0.449663	4.872382
C	1.668458	-0.603043	5.563892
C	2.487029	-1.491578	4.891465
C	2.703245	-1.309366	3.535381
C	2.098461	-0.243852	2.892831
F	0.253442	1.224848	5.607117
F	2.342918	-0.146760	1.564670
Si	-0.677021	-0.218406	-0.327804
C	-2.303419	0.704644	-0.477082
C	-3.660710	0.062372	-0.220321

C	-0.390780	-1.817312	0.595227
C	-1.574506	-2.763502	0.747634
C	0.769020	0.694768	-1.065337
C	0.413163	1.672457	-2.179880
O	-0.817794	-1.446096	-2.216723
C	-1.941628	-1.161408	-3.106840
O	-0.531329	0.956465	1.835233
C	-1.400491	0.242204	2.711512
C	-3.073014	-2.154891	-2.992492
C	-4.289916	-1.676740	-3.768218
H	-2.760005	-3.125428	-3.364565
H	-3.325255	-2.298513	-1.943933
H	-2.320010	0.008613	2.187141
H	-0.950394	-0.685307	3.064491
H	-1.657097	0.834081	3.584808
H	-4.058796	-1.540541	-4.824735
H	-5.100016	-2.399610	-3.699327
H	-4.659383	-0.725777	-3.385281
H	-2.289876	-0.163492	-2.868813
F	1.431285	-0.782147	6.873582
F	3.055510	-2.518065	5.537707
F	3.486410	-2.170844	2.862633
F	-1.702376	6.358606	5.527611
F	3.554053	5.340123	-0.852724
H	0.037209	-1.579477	1.563168
H	0.404575	-2.318677	0.053869
H	-2.004262	-3.025565	-0.215959
H	-2.365520	-2.334852	1.357462
H	-1.259574	-3.689509	1.227682
H	1.476064	-0.046310	-1.428279
H	1.288600	1.207503	-0.264611
H	-0.087590	1.179753	-3.010082
H	1.312699	2.141925	-2.575897
H	-0.239136	2.467159	-1.830326
H	-2.302897	1.190523	-1.452788
H	-2.151273	1.521859	0.225484
H	-4.439308	0.823817	-0.244636
H	-3.716248	-0.420222	0.751121
H	-3.917306	-0.684125	-0.962987

X10

108

C	-1.881082	-1.568789	-4.492993
C	-1.647886	-2.502045	-3.319299
O	-2.842972	-2.988876	-2.717121
O	-0.836937	-3.564546	-3.755061
C	-3.694877	-3.738346	-3.575496
C	-2.784278	-0.390927	-4.180389
Si	0.453667	-4.216491	-2.891686
C	1.179953	-5.489477	-4.062532
C	1.752451	-4.906299	-5.351458
C	-0.130095	-5.037302	-1.303153
C	-1.224580	-6.083593	-1.484595
C	1.679936	-2.850924	-2.497351
C	2.884707	-3.316857	-1.683265
H	-0.898216	-1.213681	-4.803970
H	-2.268904	-2.148935	-5.331159
H	-1.174508	-1.951666	-2.501818
H	-4.458757	-4.180346	-2.941435
H	-4.181734	-3.107788	-4.321822
H	-3.150471	-4.534296	-4.082651
H	-3.785751	-0.713810	-3.901336
H	-2.391080	0.206604	-3.360204
H	-2.873183	0.263614	-5.045509
H	0.402870	-6.221177	-4.296166
H	1.952625	-6.043860	-3.523140
H	2.570741	-4.216002	-5.149150
H	0.994278	-4.354973	-5.906280
H	2.140203	-5.684352	-6.009975
H	-0.462121	-4.258754	-0.614202
H	0.744968	-5.489276	-0.828257
H	-0.904230	-6.889868	-2.144039
H	-2.119002	-5.643537	-1.921005
H	-1.512579	-6.539001	-0.535985
H	1.163811	-2.053153	-1.961991
H	2.011354	-2.406099	-3.437772
H	3.431726	-4.110819	-2.192550
H	2.583803	-3.703136	-0.709958
H	3.586209	-2.503128	-1.505123
C	0.773354	-0.579419	1.419367
C	0.936395	0.797107	1.539058
C	1.497834	1.199541	2.743226
C	1.892358	0.318050	3.743759
C	1.731386	-1.042709	3.559849
C	1.162192	-1.496583	2.380545
B	0.376528	1.756118	0.340698
C	0.971777	1.383916	-1.136705
C	0.238002	1.733142	-2.264238
C	0.662225	1.499942	-3.562991
C	1.889096	0.893588	-3.775316
C	2.665517	0.541832	-2.685376
C	2.197264	0.789131	-1.402778
F	-0.959985	2.348324	-2.129705
F	-0.092563	1.858013	-4.617157
F	3.861343	-0.038464	-2.885787
F	3.012417	0.403138	-0.395442
F	1.676274	2.508437	3.027114
F	2.416605	0.772669	4.893642
F	0.989122	-2.819108	2.192271
F	0.204148	-1.095202	0.303834
C	0.568205	3.362515	0.555589
C	1.809623	3.979692	0.474741
C	1.997654	5.348161	0.572599
C	0.897286	6.170387	0.758969
C	-0.362405	5.604636	0.846996
C	-0.499732	4.227123	0.741998
F	2.920852	3.229746	0.314272
F	-1.767650	3.756537	0.843532
Si	-3.504743	-1.407125	1.064518
C	-3.268542	-0.314921	-0.414458
C	-4.010716	1.015729	-0.423999
C	-2.624520	-3.041394	0.854856
C	-3.212325	-4.262703	1.553813
C	-5.258179	-1.541723	1.705306
C	-5.806923	-0.339863	2.471140
O	-2.684348	-0.426237	2.325569
C	-2.055101	-0.570473	3.409734
O	-1.603344	0.475579	4.016295
C	-1.844313	1.791990	3.423322
C	-1.847099	-1.859031	4.110673
C	-3.084943	-2.227917	4.936649
H	-1.639070	-2.629143	3.374939
H	-0.981974	-1.761255	4.759034
H	-1.282594	2.477735	4.041454
H	-1.487661	1.798267	2.402204
H	-2.907324	2.003524	3.461965
H	-3.287461	-1.475486	5.694423
H	-3.968044	-2.338459	4.313827
H	-2.902334	-3.175840	5.434686
H	-0.810778	1.527467	0.300407
F	3.224954	5.889948	0.494884
F	1.052941	7.498713	0.857792
F	-1.434977	6.395841	1.031433
F	2.318587	0.649186	-5.022773
F	2.097406	-1.911316	4.515026
H	-1.572356	-2.914958	1.110532
H	-2.620728	-3.206305	-0.224893
H	-4.220327	-4.474417	1.201735
H	-3.268049	-4.144698	2.634804
H	-2.606699	-5.145217	1.356053
H	-5.341682	-2.442680	2.314730
H	-5.876065	-1.735189	0.823437
H	-5.247138	-0.160626	3.387765
H	-6.845943	-0.503367	2.753741
H	-5.766779	0.572920	1.882257
H	-3.549616	-0.918843	-1.279129
H	-2.195488	-0.146519	-0.519279
H	-5.090407	0.874616	-0.410307
H	-3.766294	1.582997	-1.320157
H	-3.742734	1.634915	0.429474

X11

67

C	-5.351520	-5.708230	-4.651980

O	-5.967264	-4.619315	-4.679271
Si	-7.630029	-4.215565	-4.057764
C	-8.633147	-4.290875	-5.622732
C	-10.022848	-3.667337	-5.492761
C	-3.978723	-5.791780	-5.151019
C	-3.485733	-7.190669	-5.475595
C	-7.306136	-2.502481	-3.391444
C	-8.438510	-1.970572	-2.511042
C	-8.017397	-5.467784	-2.736856
C	-7.080067	-5.449679	-1.531257
H	-3.863586	-5.087040	-5.975125
H	-3.377519	-5.365534	-4.332855
H	-5.837823	-6.582218	-4.215679
H	-4.081386	-7.648374	-6.259078
H	-2.455254	-7.141543	-5.815854
H	-3.518415	-7.834311	-4.600878
H	-7.130427	-1.829530	-4.232755
H	-6.374469	-2.524327	-2.822388
H	-8.613249	-2.609980	-1.647499
H	-9.377699	-1.894818	-3.054924
H	-8.200119	-0.976549	-2.135776
H	-8.055950	-6.458613	-3.187717
H	-9.043300	-5.266576	-2.417971
H	-6.051766	-5.684656	-1.806024
H	-7.387051	-6.189761	-0.794410
H	-7.068921	-4.479658	-1.037799
H	-8.716078	-5.335730	-5.915552
H	-8.068266	-3.791734	-6.412544
H	-9.970059	-2.597456	-5.299769
H	-10.601362	-4.123584	-4.691380
H	-10.586158	-3.804713	-6.414282
C	-9.312467	-8.252940	-8.103711
C	-8.252374	-7.881440	-7.287528
C	-7.432288	-6.900530	-7.824996
C	-7.644783	-6.287147	-9.047122
C	-8.729317	-6.677630	-9.815047
C	-9.569455	-7.669438	-9.337437
B	-7.955378	-8.442489	-5.786679
C	-6.628772	-9.381259	-5.672930
C	-6.036960	-10.084590	-6.712206
C	-4.897353	-10.861996	-6.557634
C	-4.316230	-10.981265	-5.306217
C	-4.880338	-10.310257	-4.233898
C	-6.010731	-9.537508	-4.440290
F	-6.570065	-10.058630	-7.952994
F	-4.360794	-11.516834	-7.600467
F	-4.314254	-10.403474	-3.016774
F	-6.504209	-8.896512	-3.348424
F	-6.333189	-6.482014	-7.137981
F	-6.816606	-5.326956	-9.497095
F	-10.615090	-8.063520	-10.081824
F	-10.160913	-9.234519	-7.730994
C	-9.253196	-9.123866	-5.071330
C	-9.452549	-10.487429	-4.902422
C	-10.547492	-11.026152	-4.241422
C	-11.523929	-10.183193	-3.738176
C	-11.383243	-8.815493	-3.903417
C	-10.269394	-8.325766	-4.565573
F	-8.571961	-11.381497	-5.404644
F	-10.215317	-6.979861	-4.718805
H	-7.702394	-7.447970	-5.133753
F	-10.682772	-12.355264	-4.098680
F	-12.592075	-10.683888	-3.100926
F	-12.328190	-7.984883	-3.426624
F	-3.215767	-11.728039	-5.135747
F	-8.959309	-6.105120	-11.005058

2.5 Experiments to support the silyl transfer pathway

To validate the newly described alternative pathway to overreduction via silyl transfer (Fig. S13, path C), a simple experiment has been devised (Fig. S18): to the optimized model reaction (of substrate **2**) an additional "dummy substrate" is added. This compound is an ester that is sterically hindered enough so that it can't be reduced using our standard methodology, but it can serve as a Lewis base in the hidrosilylation reaction all the way to the silane activation step, leading to a silylated intermediate (similar to A4'). This intermediate can transfer its silyl group to the already formed acetal **4**, leading to overreduction. Thus, by the addition of the dummy substrate, one should see an increase in overreduction of **2**. In our case, isopropyl pivalate (**65**) has been chosen as a dummy substrate, because we knew that it can't be reduced under these conditions.

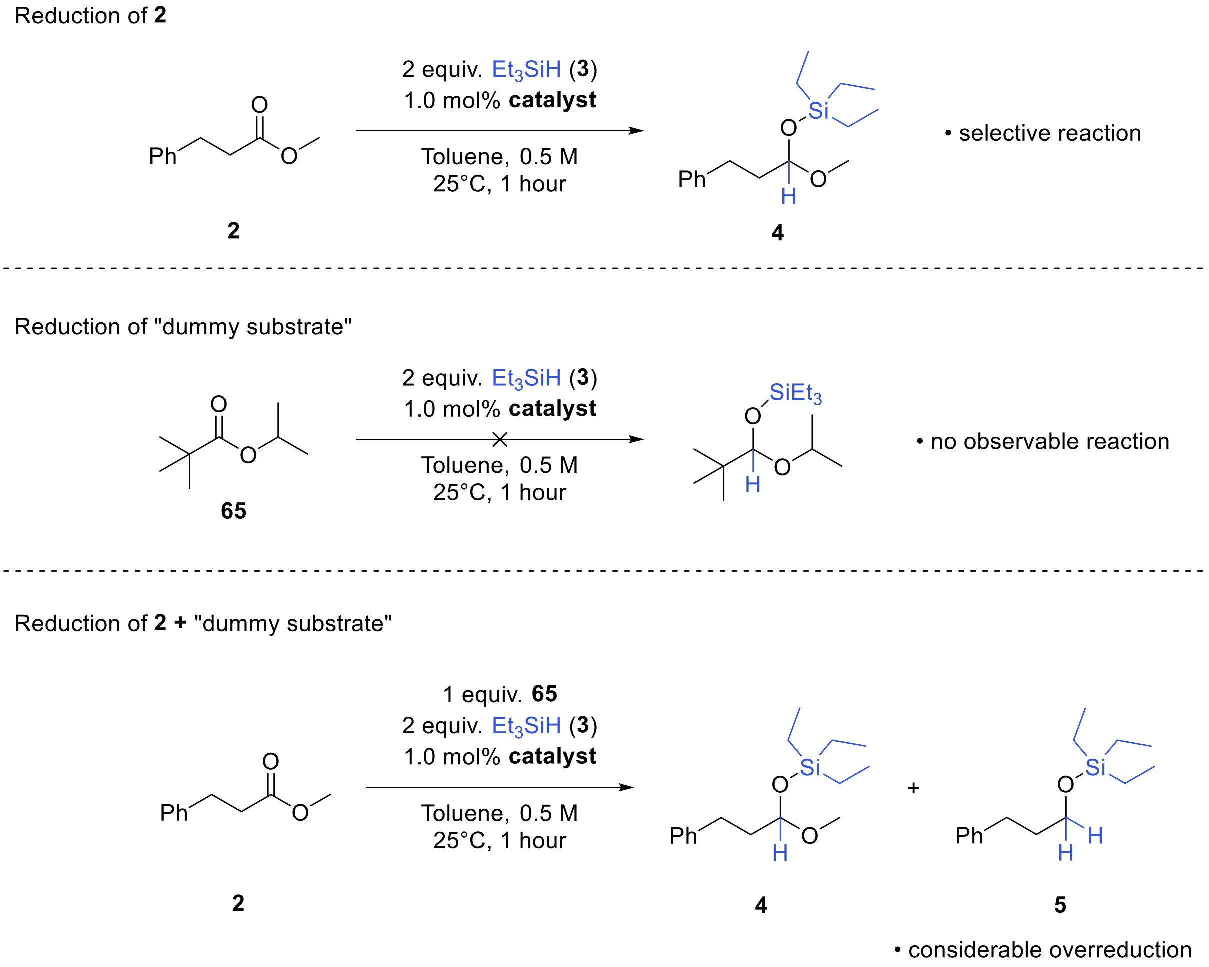

Fig. S18. Experiments to support the silyl transfer pathway

Table S11. Experiments using the dummy substrate

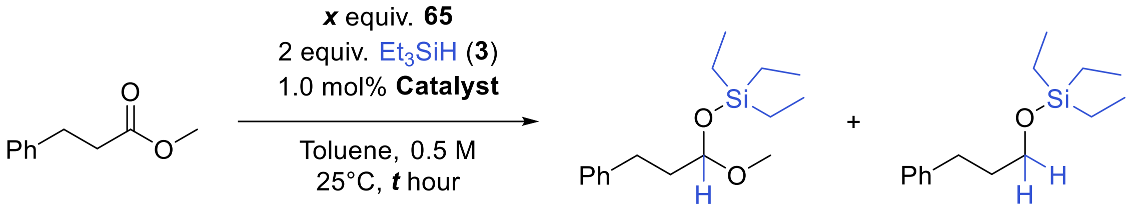

2 **4** **5**

Entry	Catalyst	Dummy substrate equiv. (*x*)	Reaction time (*t* / hour)	Conv. (%)	Yield of 4 (%)	Yield of 5 (%)
1	**1d**	0.0	1	99+	97	3
2	**1d**	1.0	1	99+	89	10
3	**1d**	0.0	4	99+	90	9
4	**1d**	1.0	4	99+	73	26
5	**1f**	0.0	1	93	93	0
6	**1f**	1.0	1	91	91	0
7	**1f**	0.0	4	99+	99+	0
8	**1f**	1.0	4	99+	99+	0

Experiments were executed according to the General procedure C. Conversion and yields were determined by ^{1}H q-NMR, using hexamethylbenzene (**S7**) as an internal standard.

As seen from Table S11, addition of the dummy substrate significantly increased overreduction when **1d** was used as a catalyst (Entries 2 and 4 vs. Entries 1 and 3). The more robust **1f** catalyst, on the other hand, maintained its selectivity even in the presence of the dummy substrate.

3. NMR Spectra

Compound S4: potassium (2-bromo-6-fluorophenyl)trifluoroborate (See compound data)

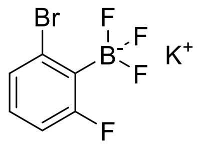

S4

^{1}H-NMR (300 MHz, DMSO-d^6) spectra of **S4**

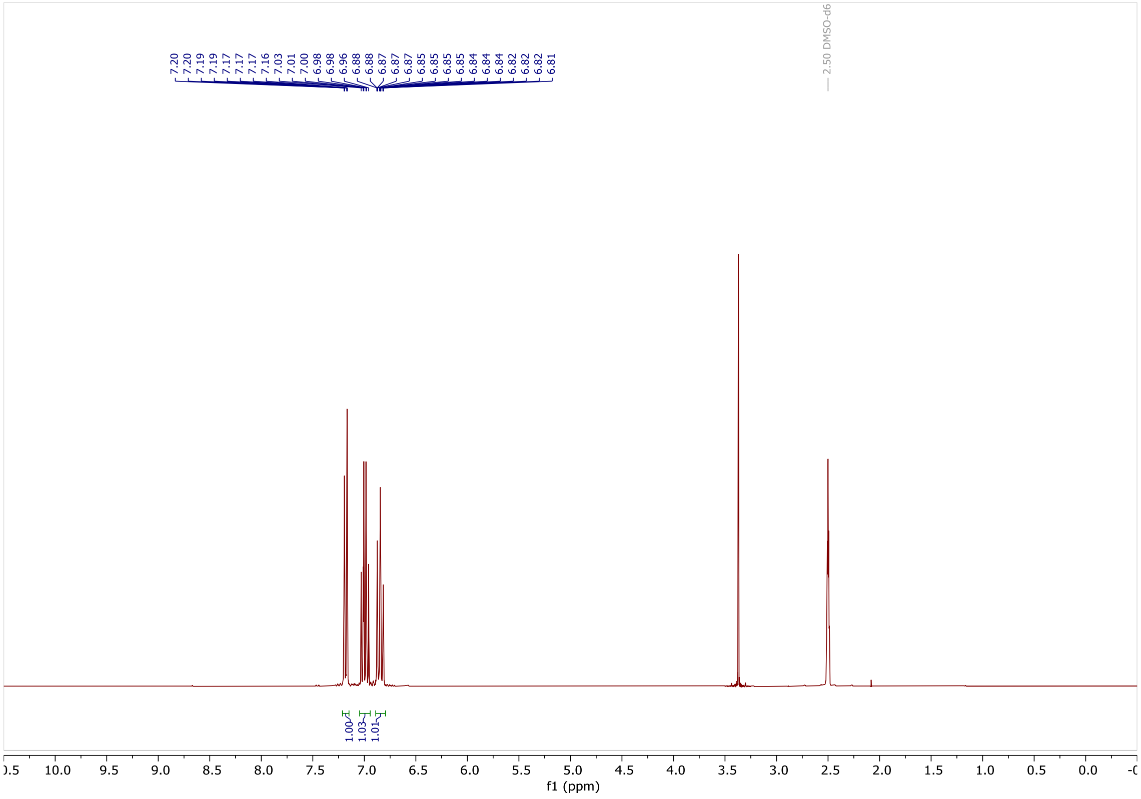

^{13}C-NMR (75 MHz, DMSO-d^6) spectra of **S4**

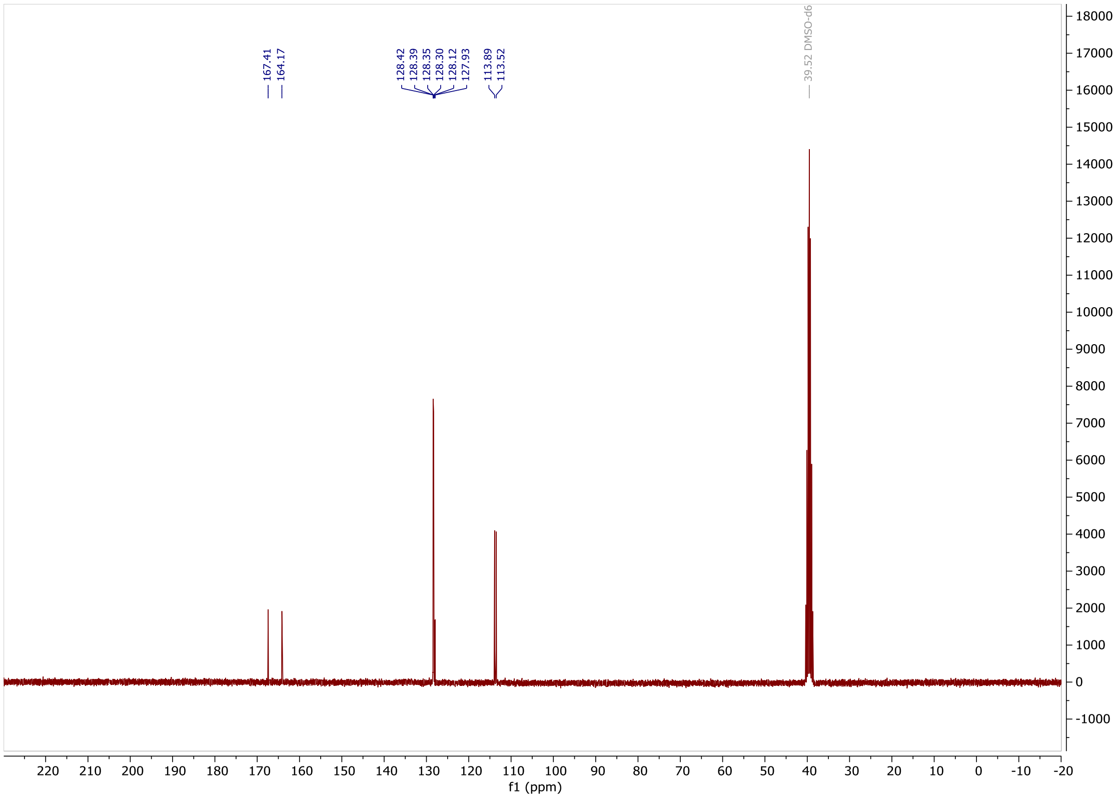

^{19}F-NMR (282 MHz, DMSO-d^6) spectra of **S4**

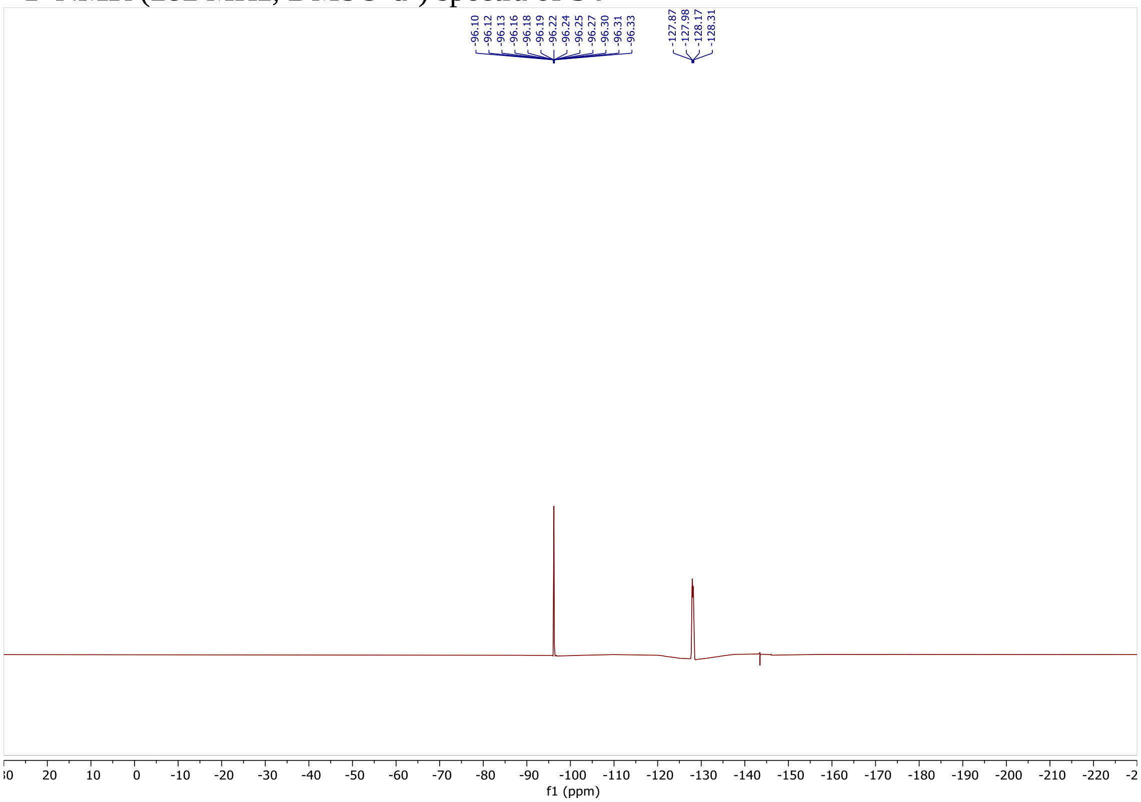

Compound 1b: (2,6-difluorophenyl)bis(2,3,5,6-tetrafluorophenyl)borane (See compound data)

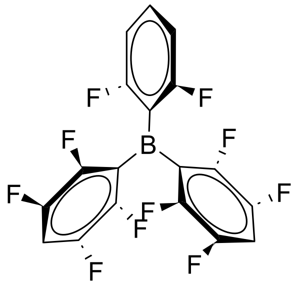

1b

^{1}H-NMR (500 MHz, C_6D_6) spectra of **1b**

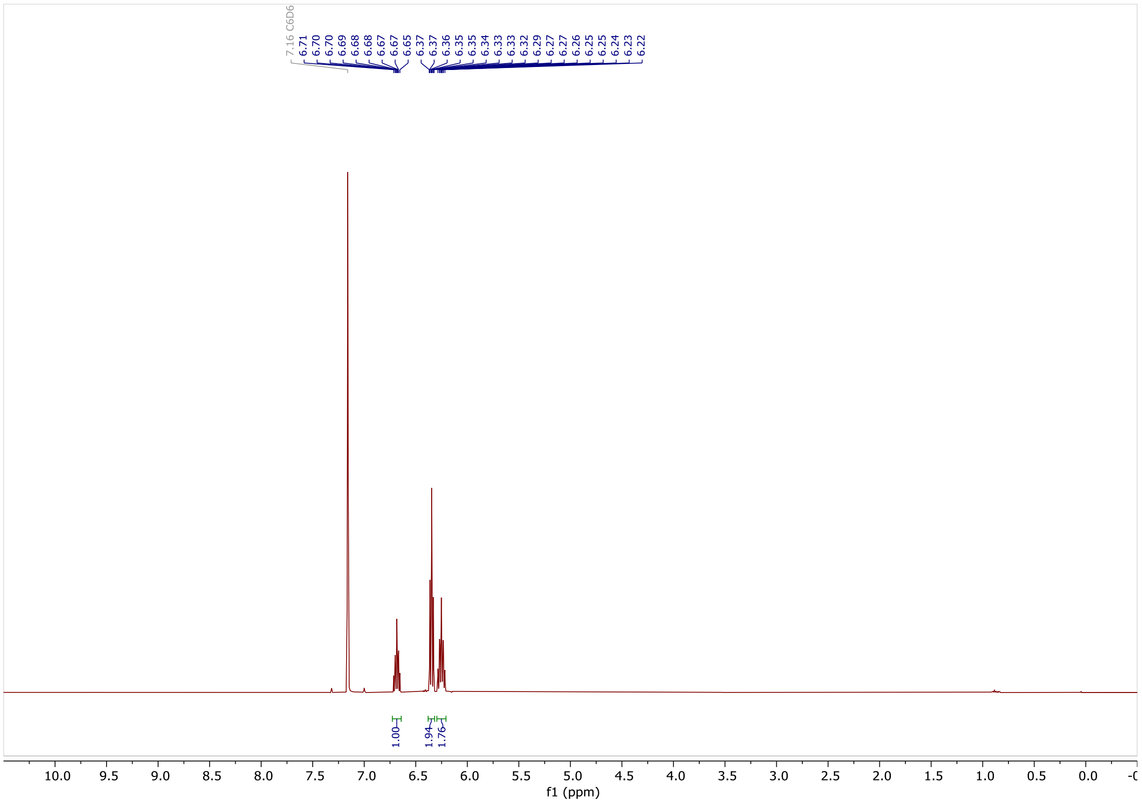

^{13}**C-NMR** (126 MHz, C_6D_6) spectra of **1b**

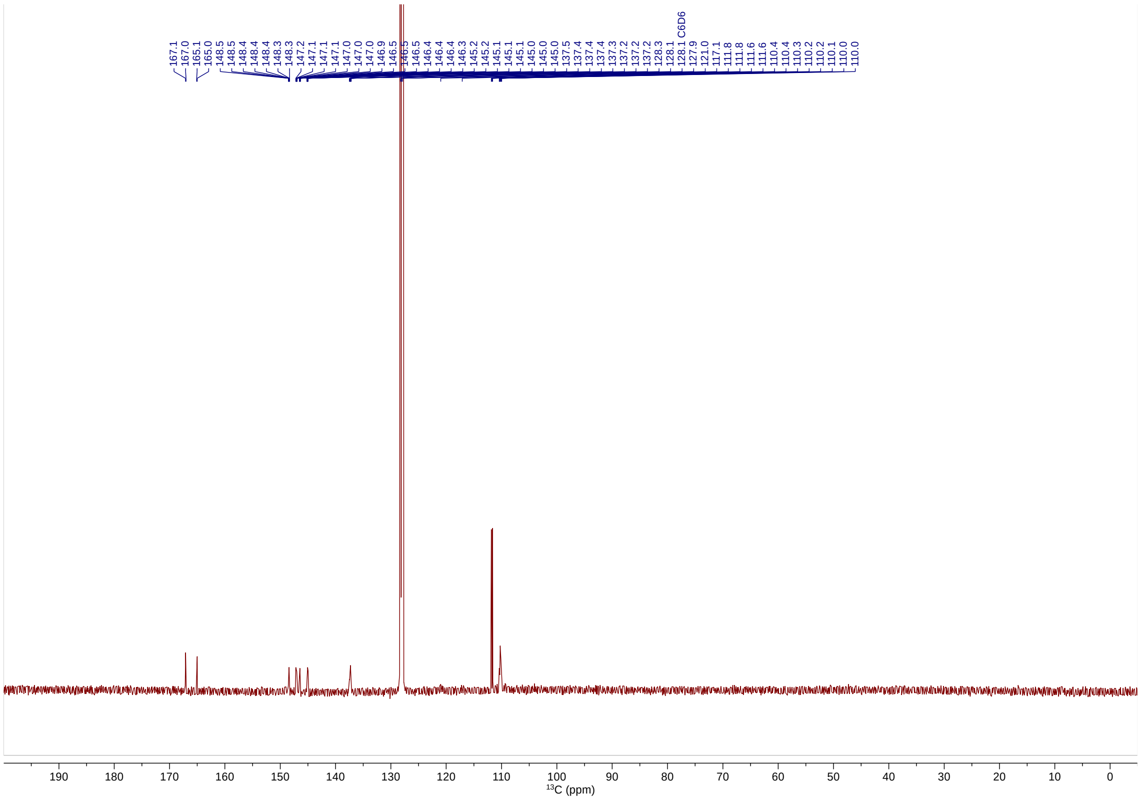

^{19}**F-NMR** (282 MHz, C_6D_6) spectra of **1b**

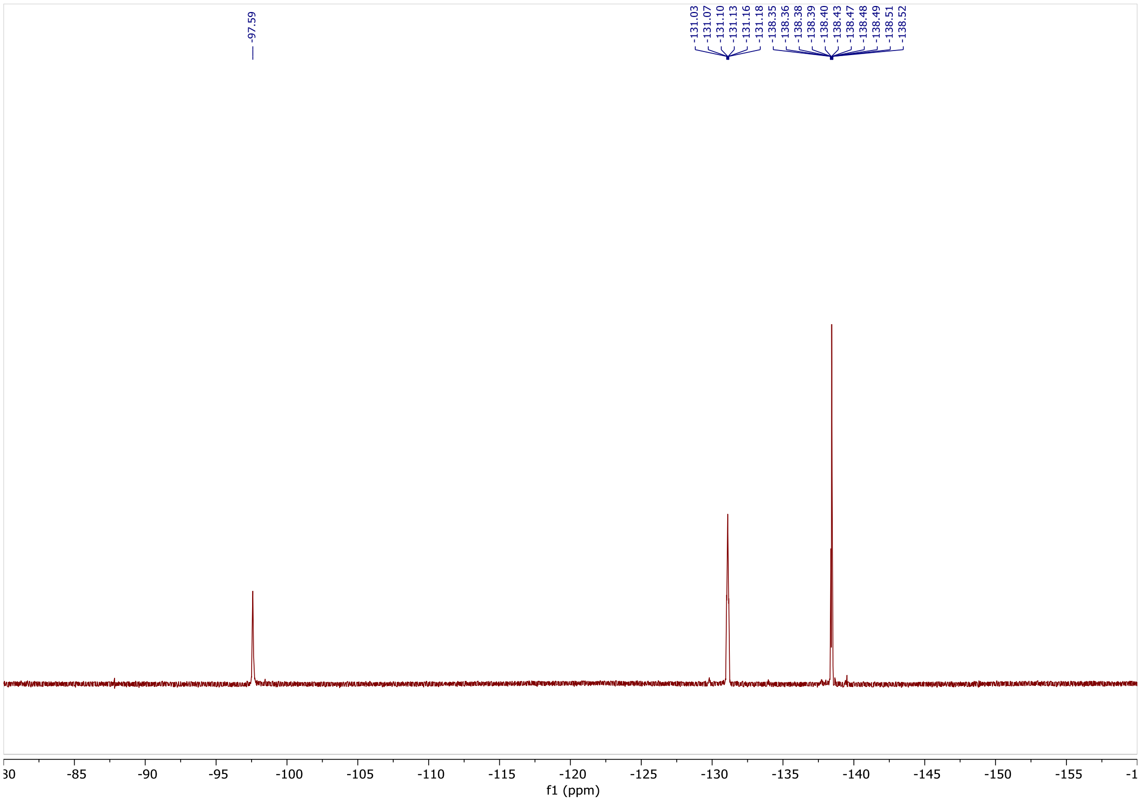

^{10}B-NMR (54 MHz, C_6D_6) spectra of **1b**

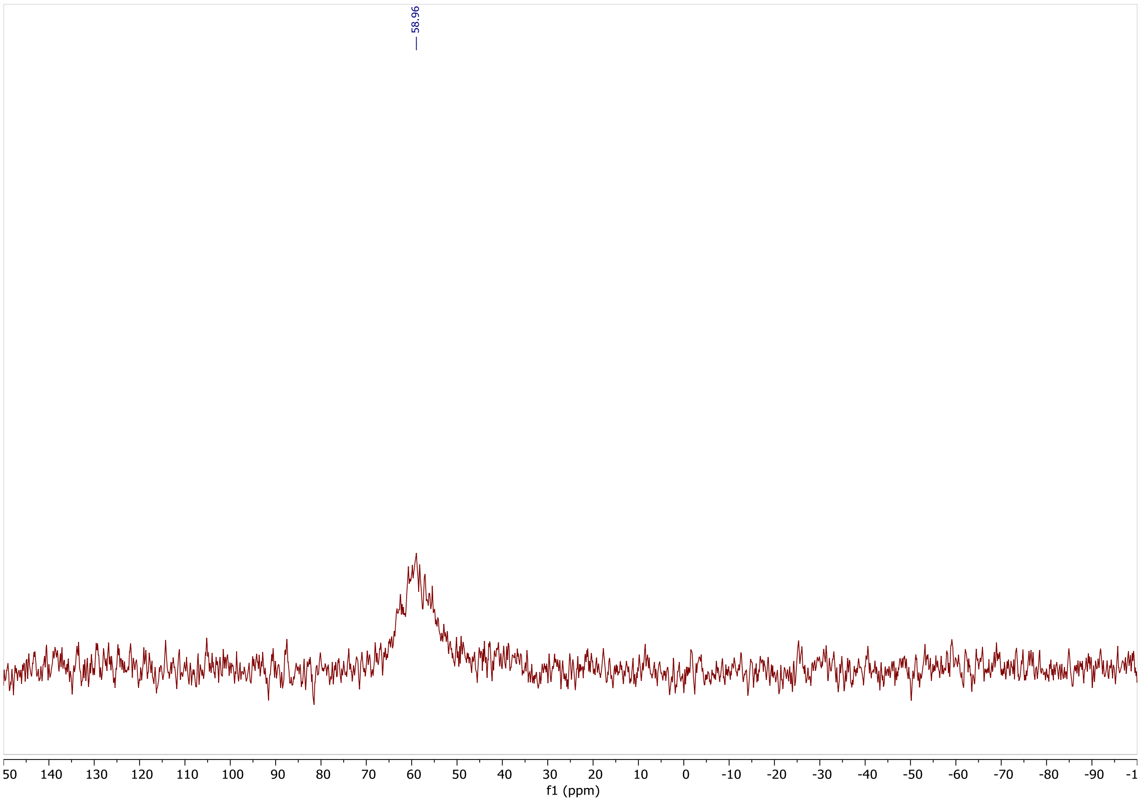

Compound 1c: (2,6-dibromophenyl)bis(2,3,5,6-tetrafluorophenyl)borane (See compound data)

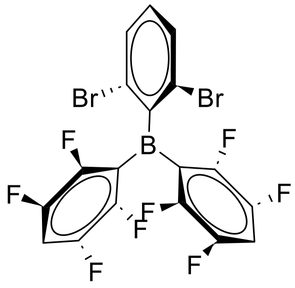

1c

^{1}H-NMR (500 MHz, C_6D_6) spectra of **1c**

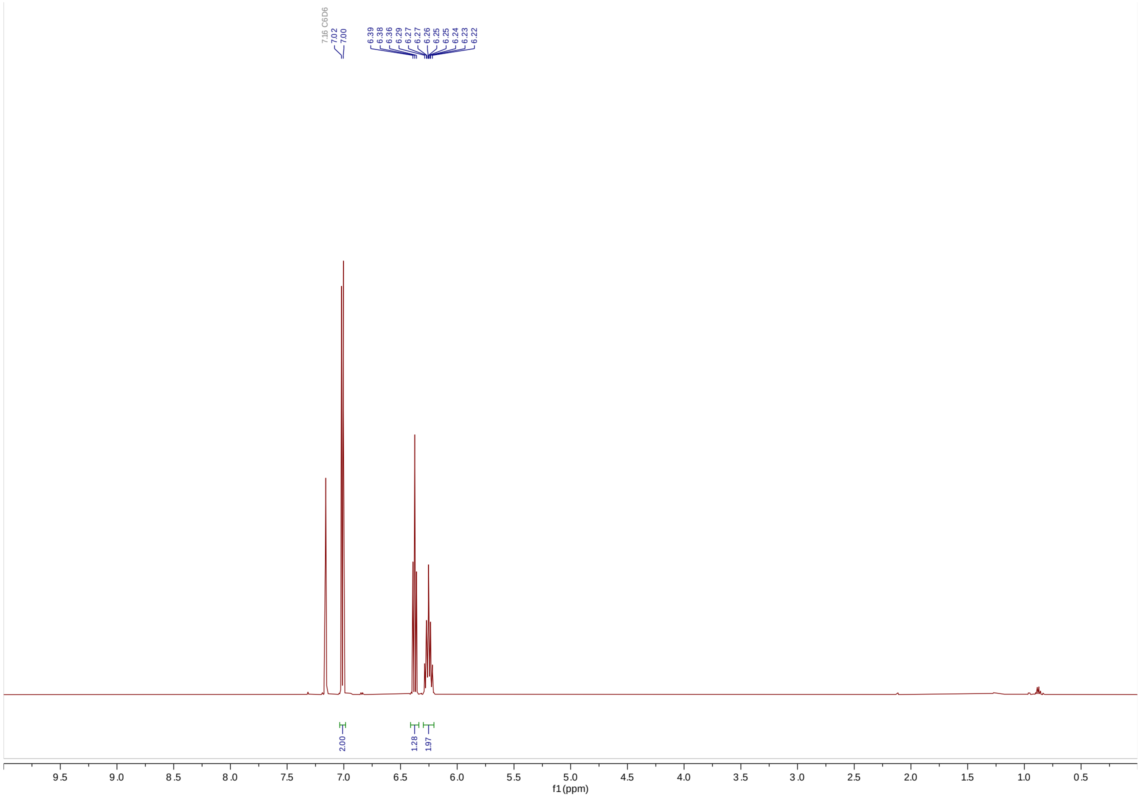

^{13}C-NMR (126 MHz, C_6D_6) spectra of **1c**

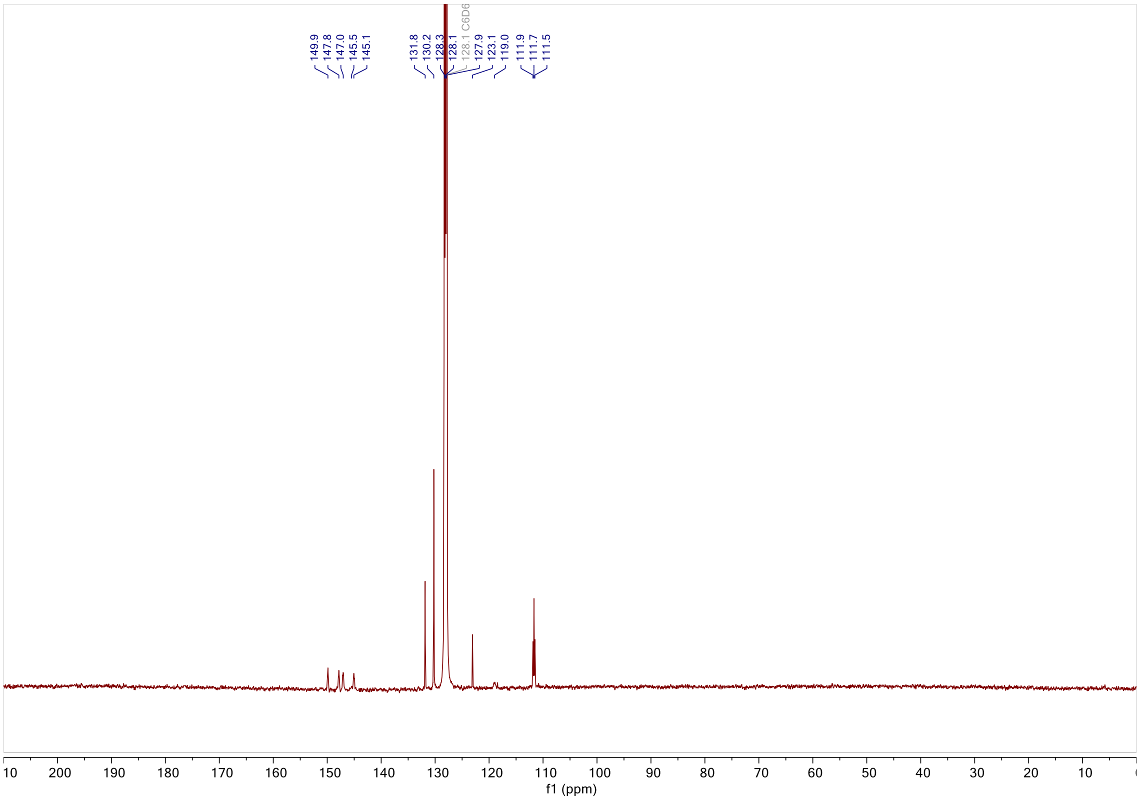

^{19}F-NMR (282 MHz, C_6D_6) spectra of **1c**

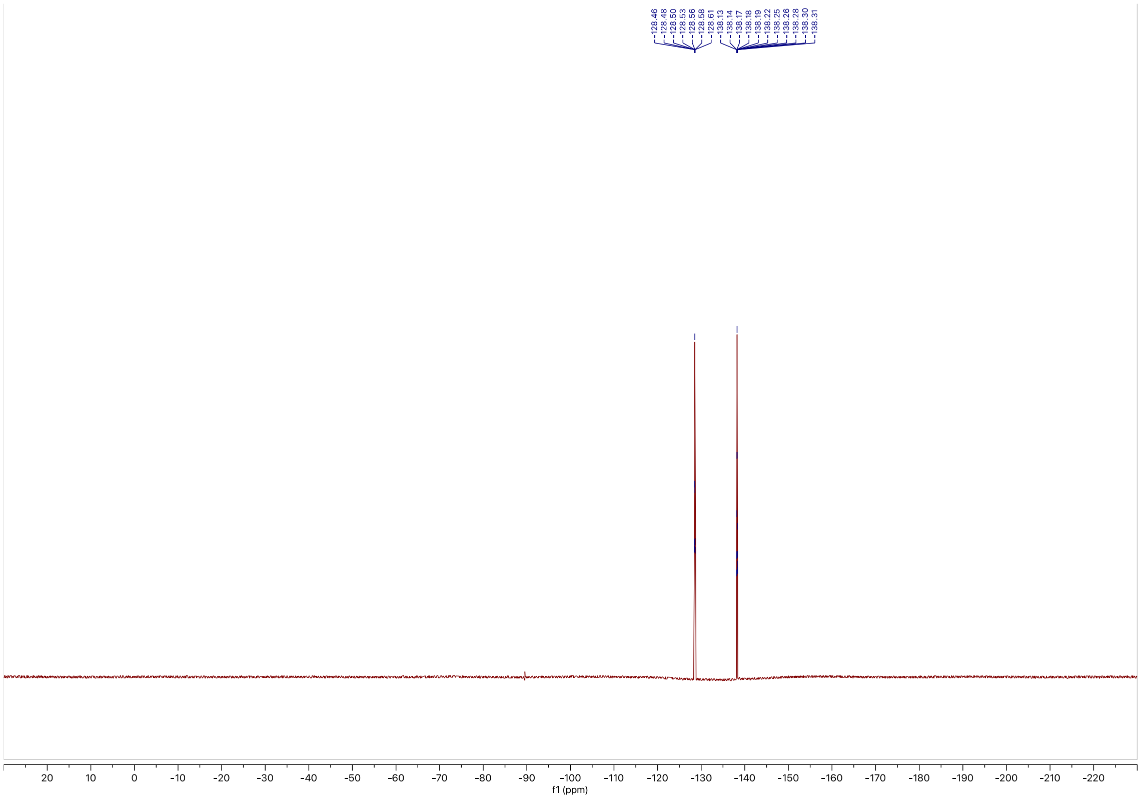

^{10}**B-NMR** (54 MHz, C_6D_6) spectra of **1c**

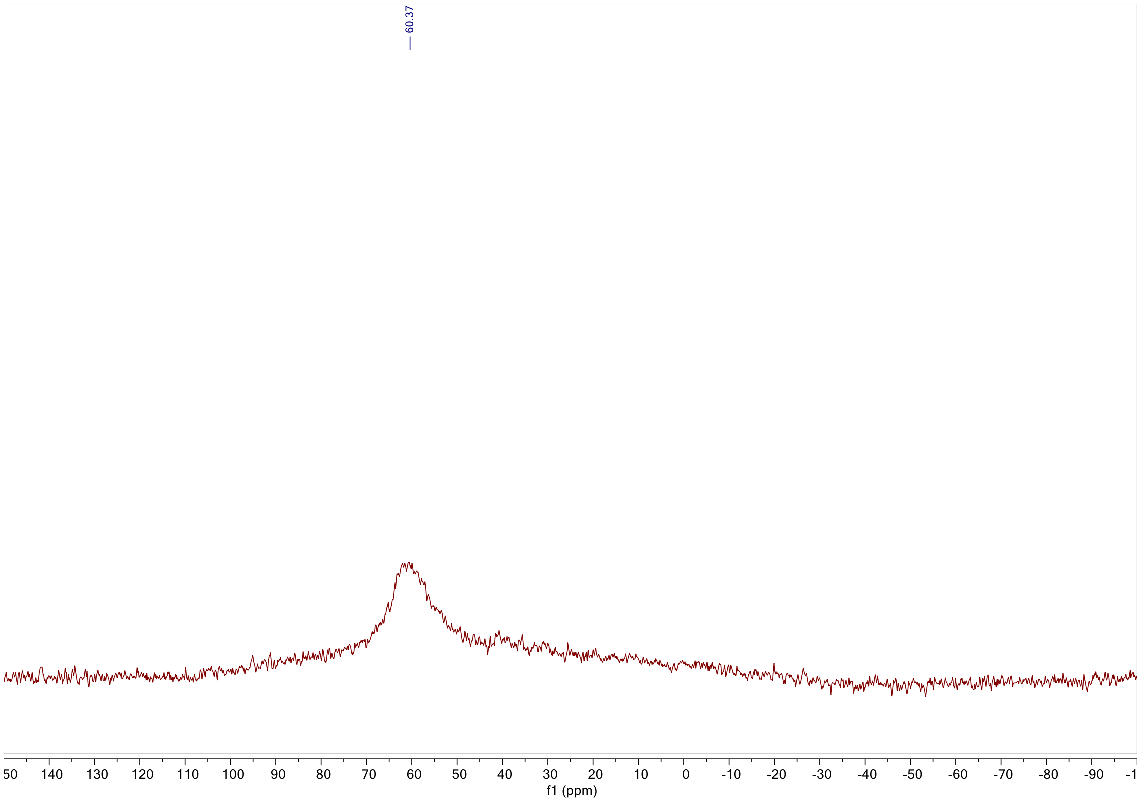

Compound 1d: (2-bromo-6-fluorophenyl)bis(2,3,5,6-tetrafluorophenyl)borane (See compound data)

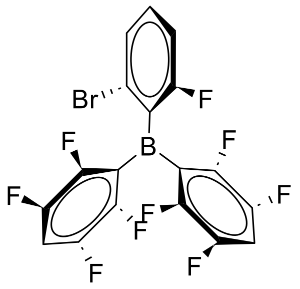

1d

^{1}H-NMR (500 MHz, C_6D_6) spectra of **1d**

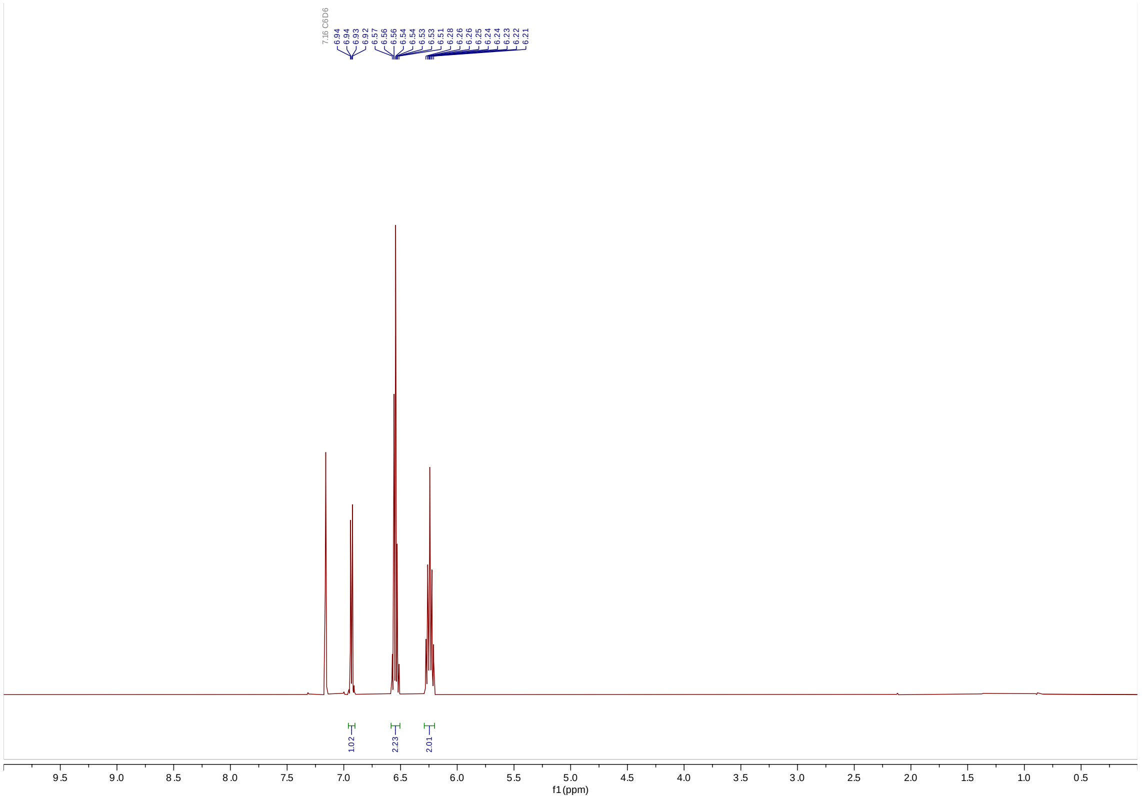

^{13}**C-NMR** (126 MHz, C_6D_6) spectra of **1d**

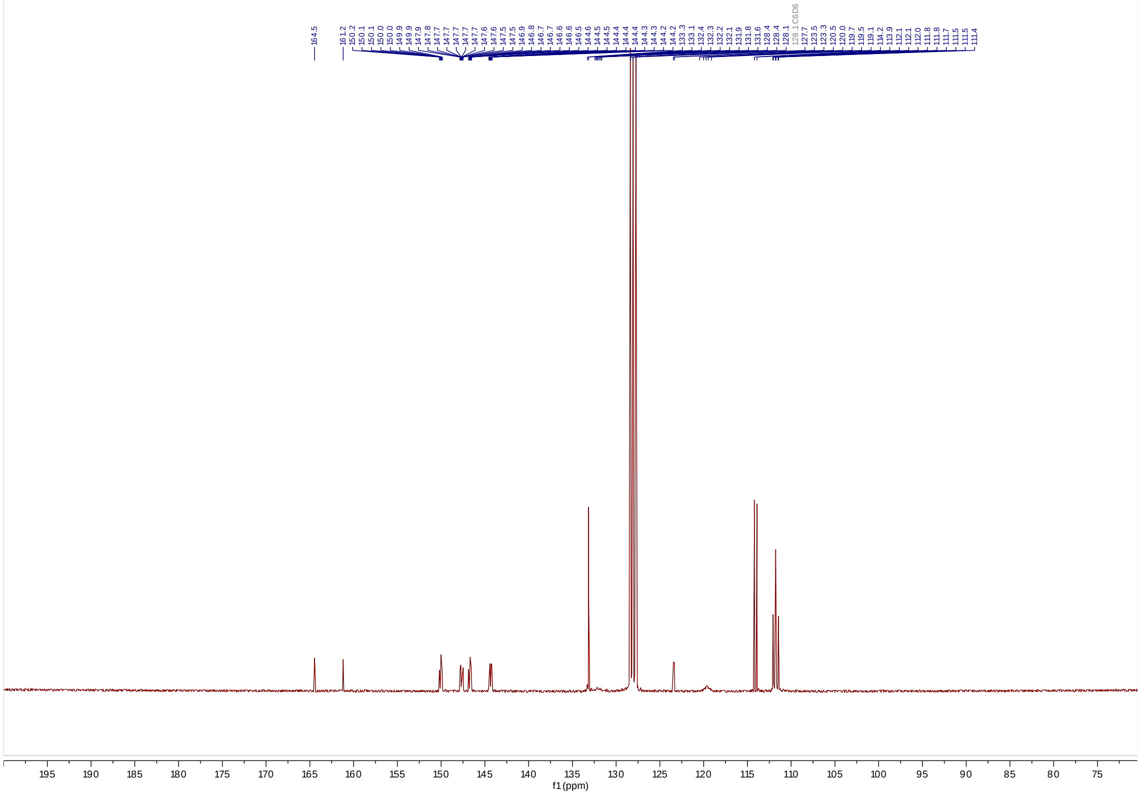

^{19}**F-NMR** (282 MHz, C_6D_6) spectra of **1d**

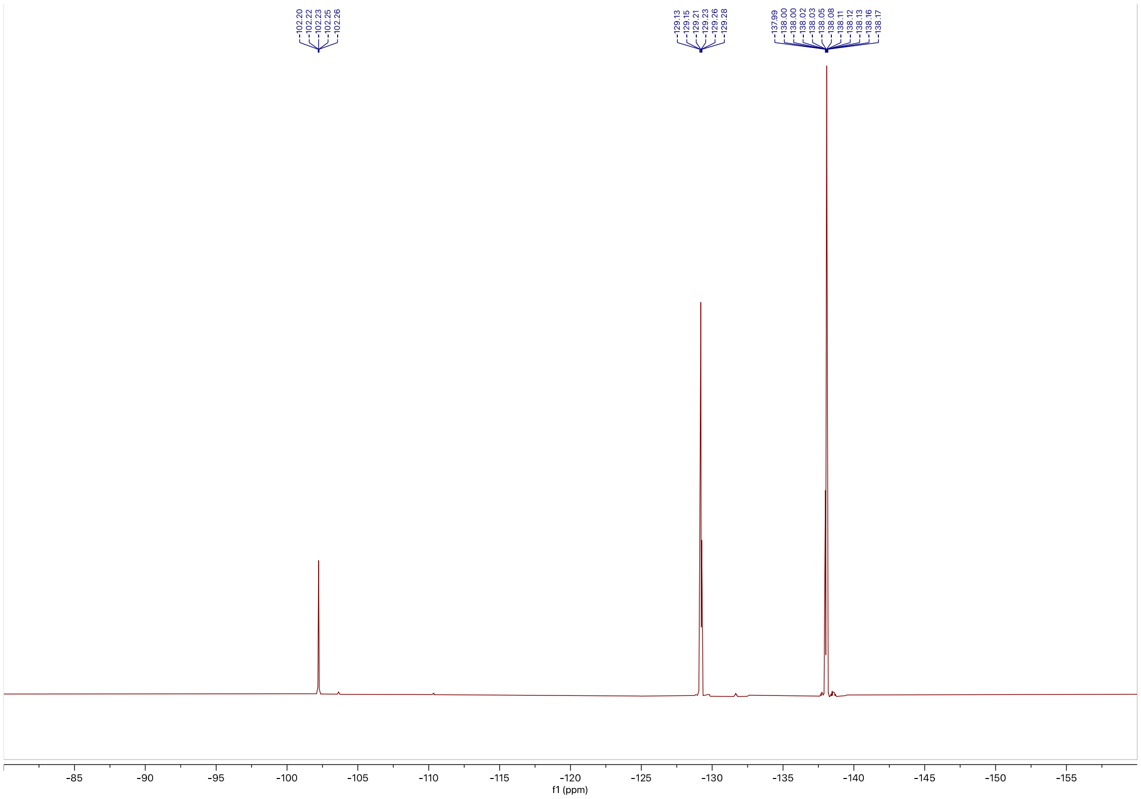

^{10}B-NMR (54 MHz, C_6D_6) spectra of **1d**

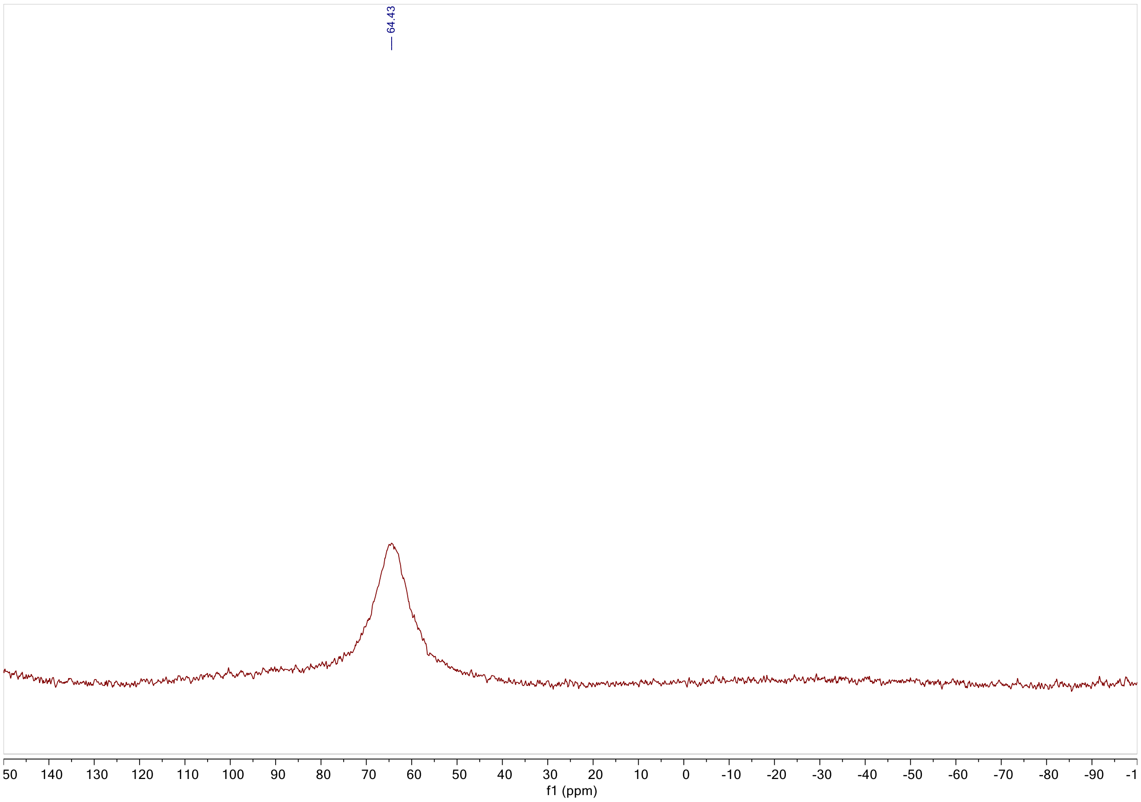

Compound 1e: (2-bromo-6-fluorophenyl)bis(perfluorophenyl)borane (See compound data)

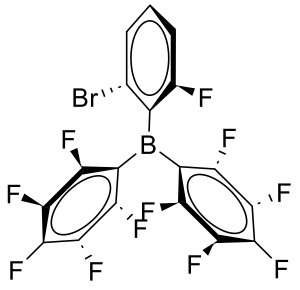

1e

[1]**H-NMR** (500 MHz, C_6D_6) spectra of **1e**

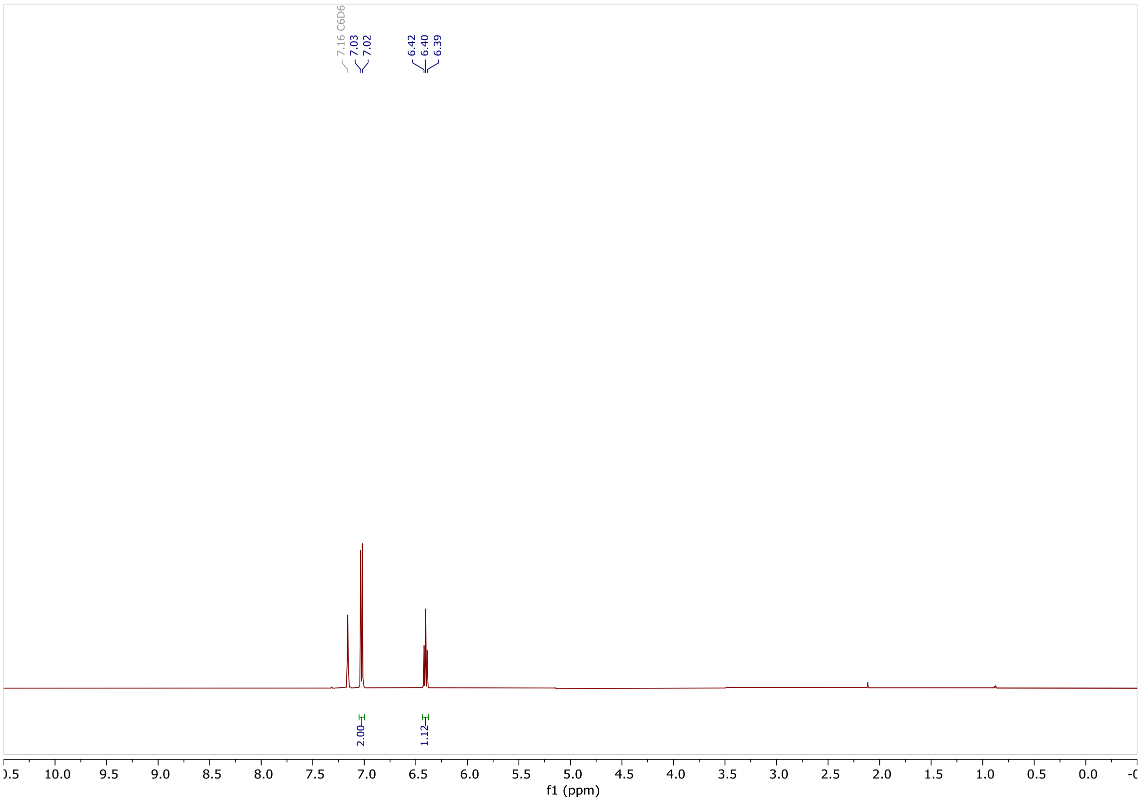

^{13}**C-NMR** (75 MHz, C_6D_6) spectra of **1e**

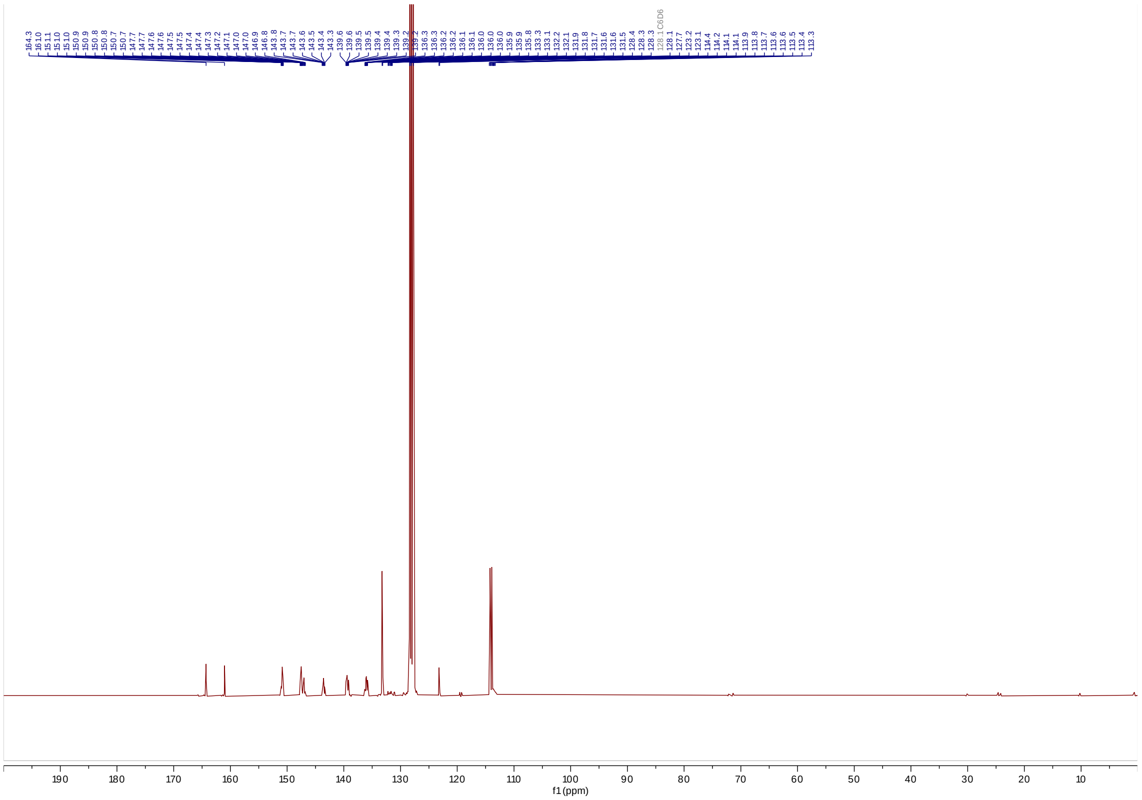

^{19}**F-NMR** (282 MHz, C_6D_6) spectra of **1e**

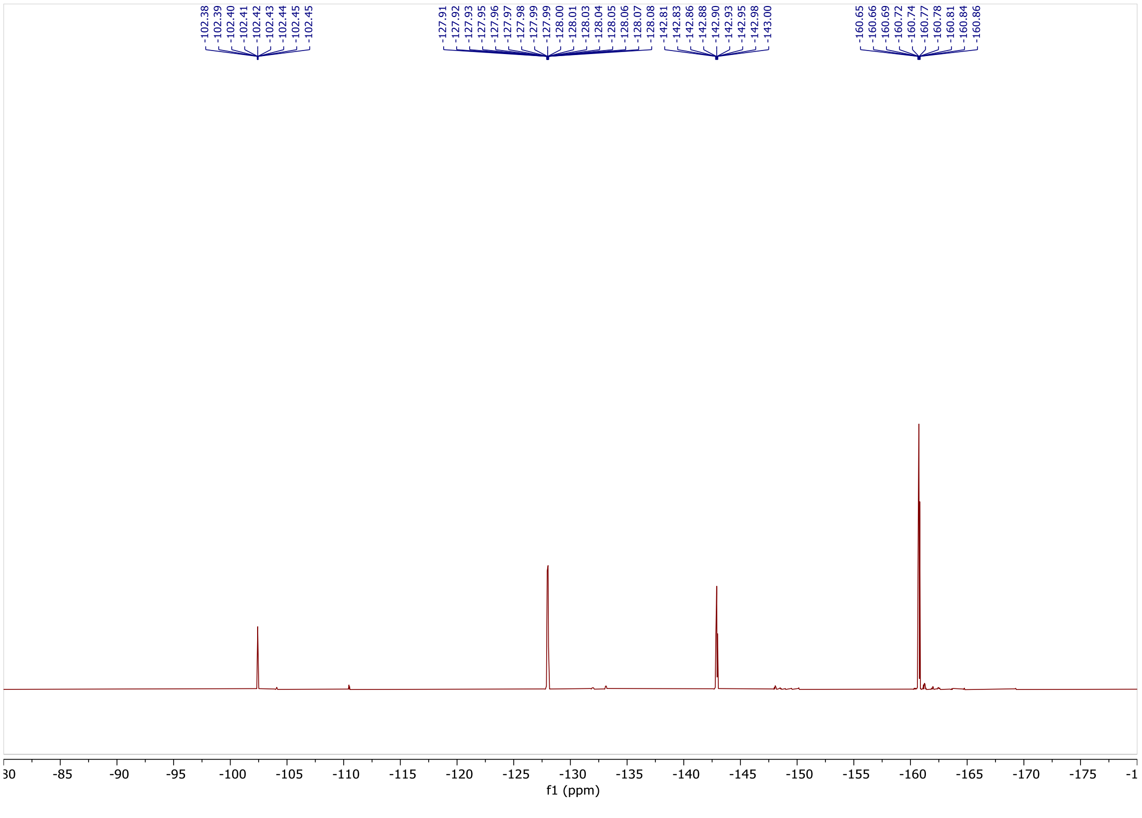

^{10}B-NMR (54 MHz, C_6D_6) spectra of **1e**

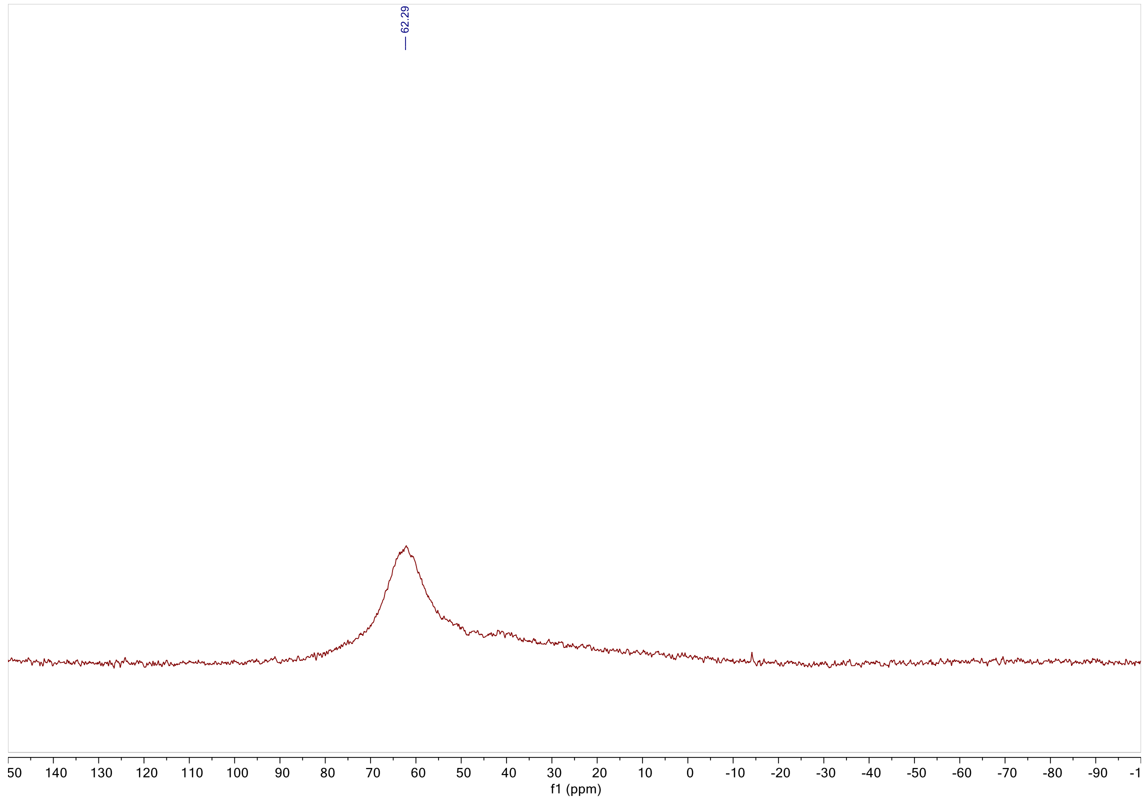

Compound 1f: (2-bromo-6-fluorophenyl)bis(2,3,6-trifluorophenyl)borane (See compound data)

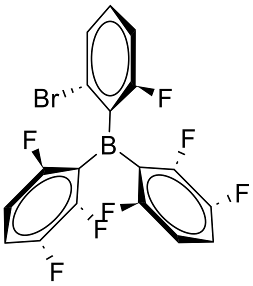

1f

^{1}H-NMR (500 MHz, C_6D_6) spectra of **1f**

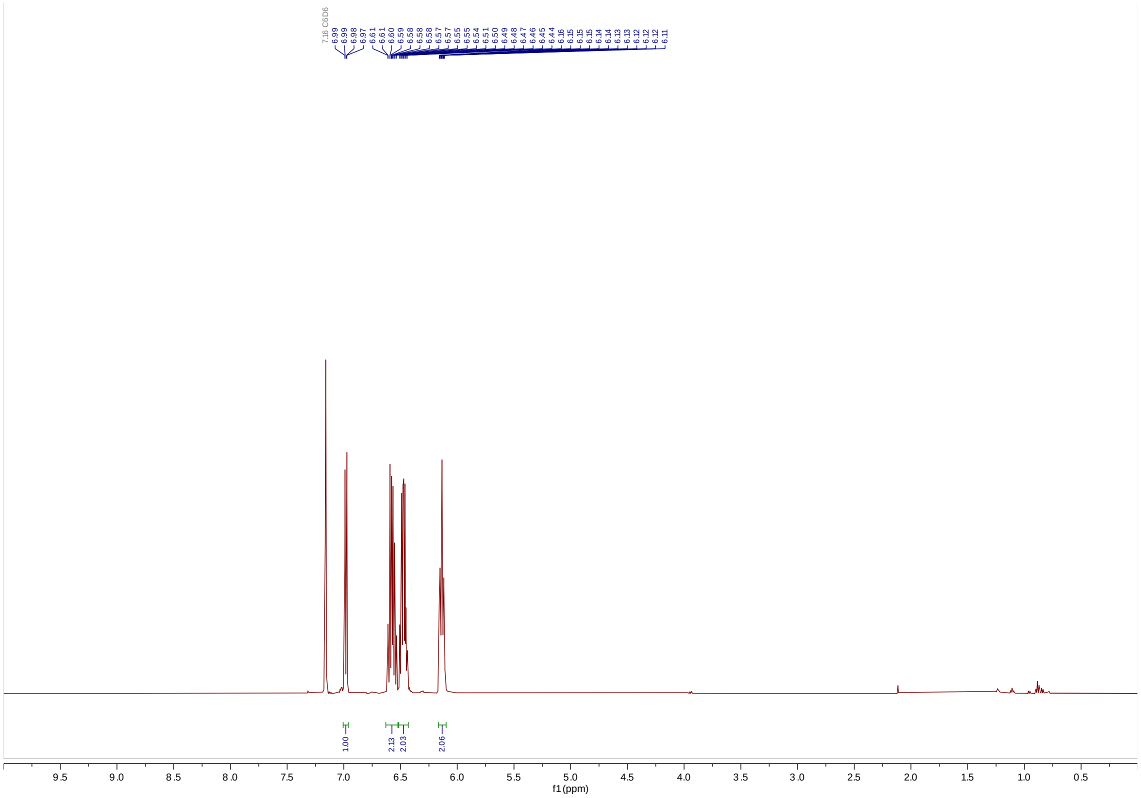

^{13}C-NMR (126 MHz, C_6D_6) spectra of **1f**

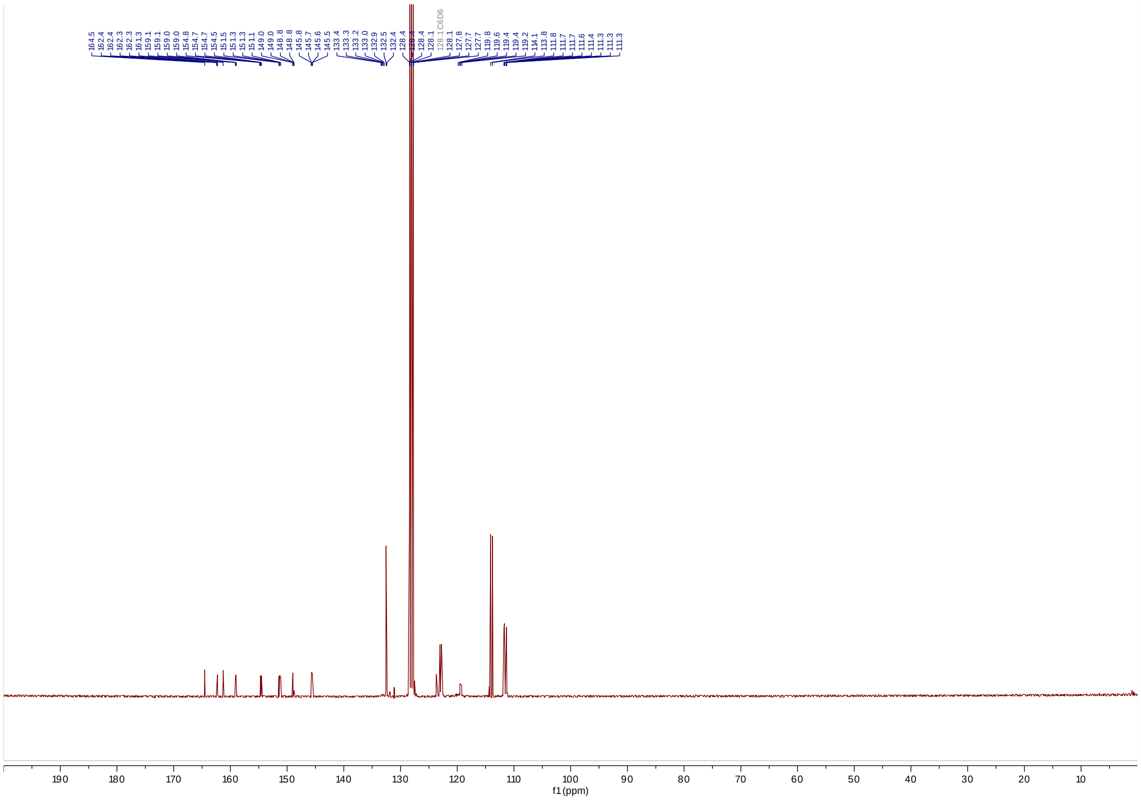

^{19}F-NMR (282 MHz, C_6D_6) spectra of **1f**

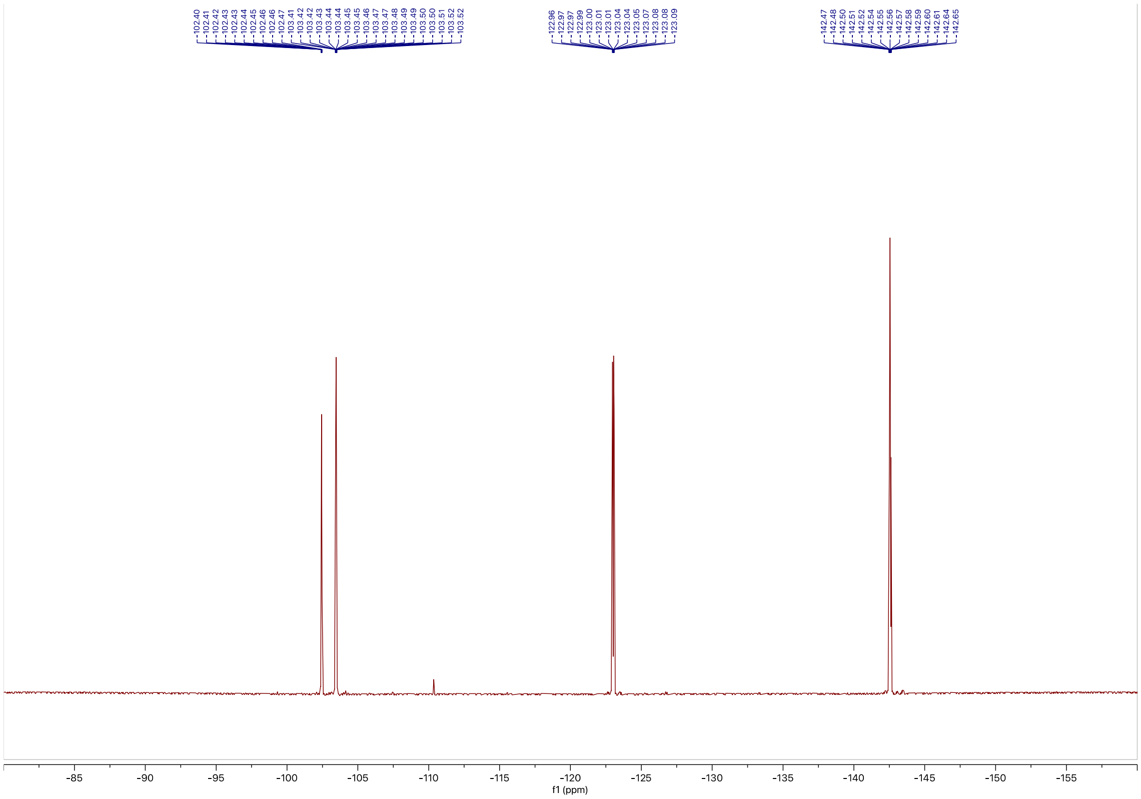

[10]B-NMR (54 MHz, C_6D_6) spectra of **1f**

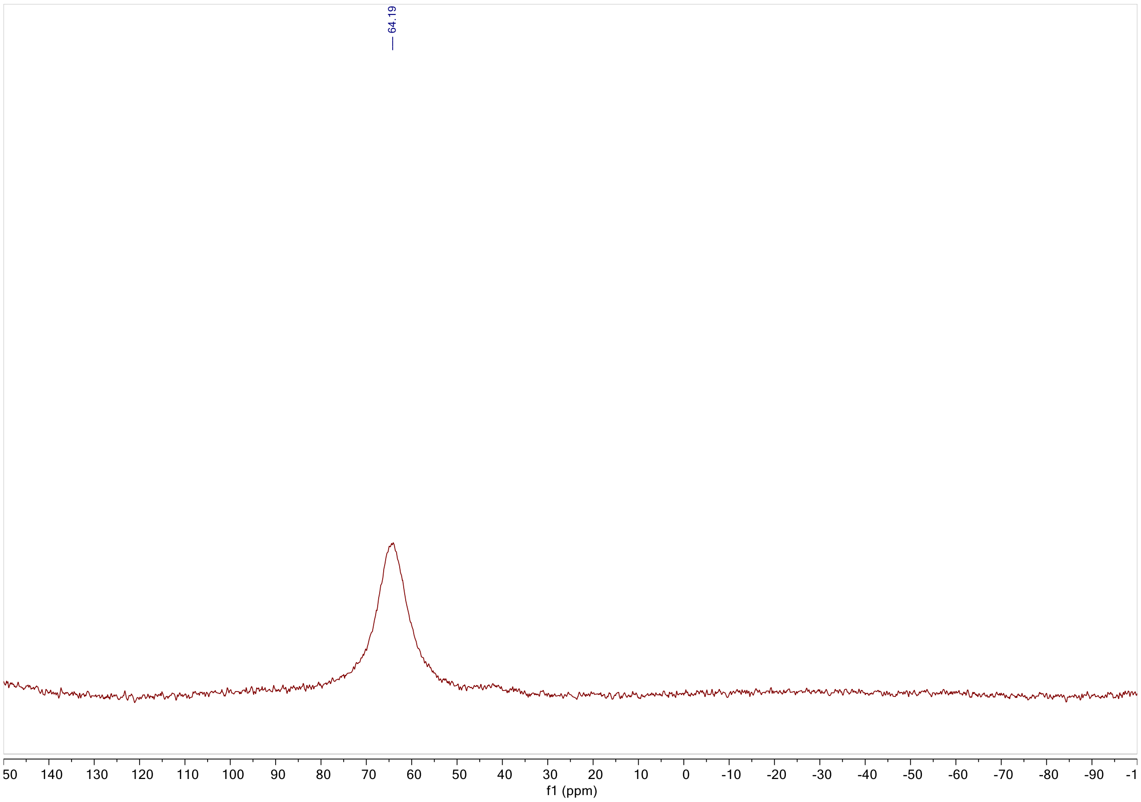

Compound 1g: (2-bromo-6-fluorophenyl)bis(2,4,6-trifluorophenyl)borane (See compound data)

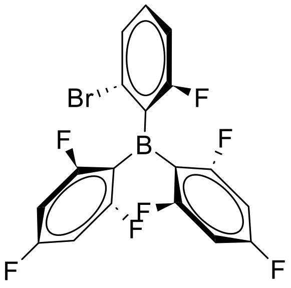

1g

^{1}H-NMR (500 MHz, C_6D_6) spectra of **1g**

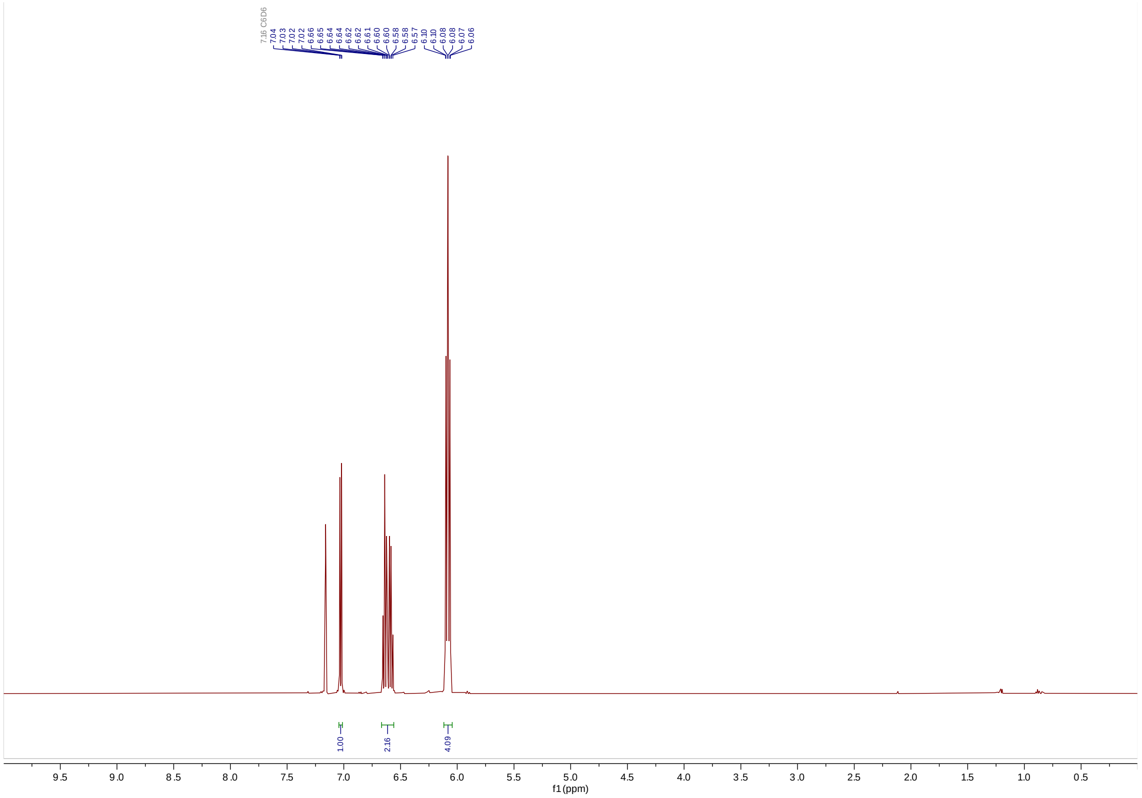

^{13}C-NMR (126 MHz, C_6D_6) spectra of **1g**

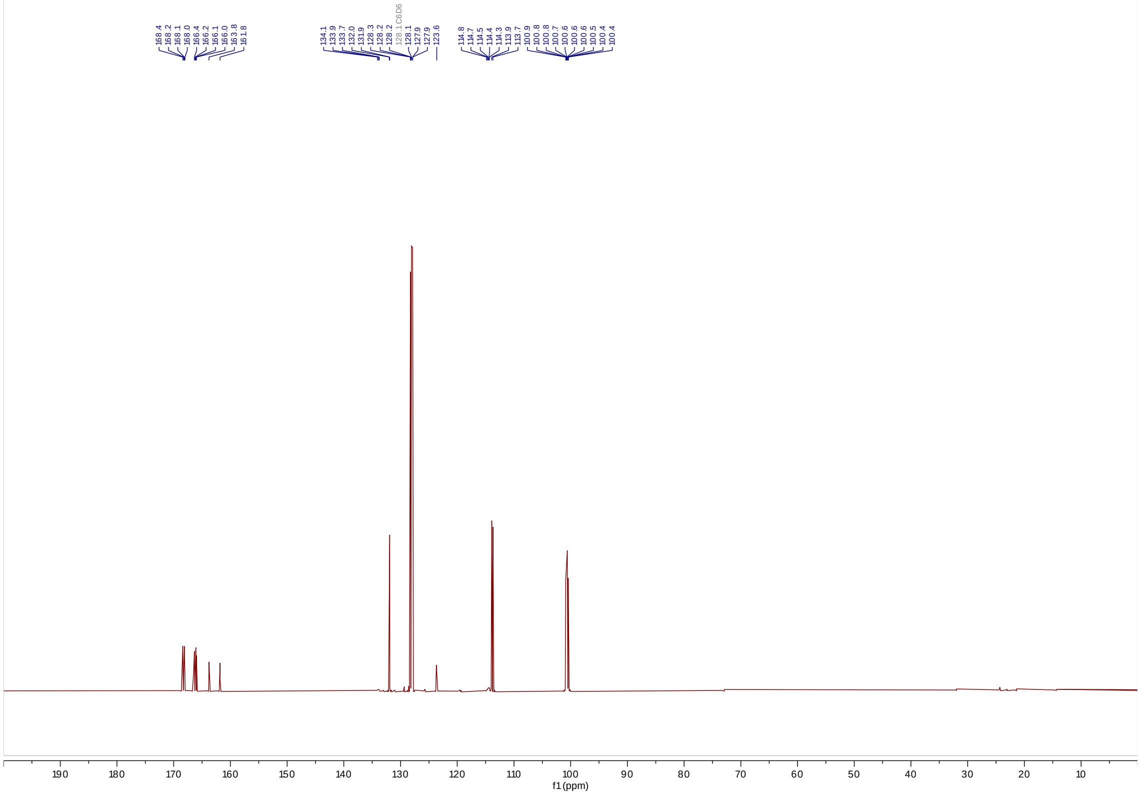

^{19}F-NMR (282 MHz, C_6D_6) spectra of **1g**

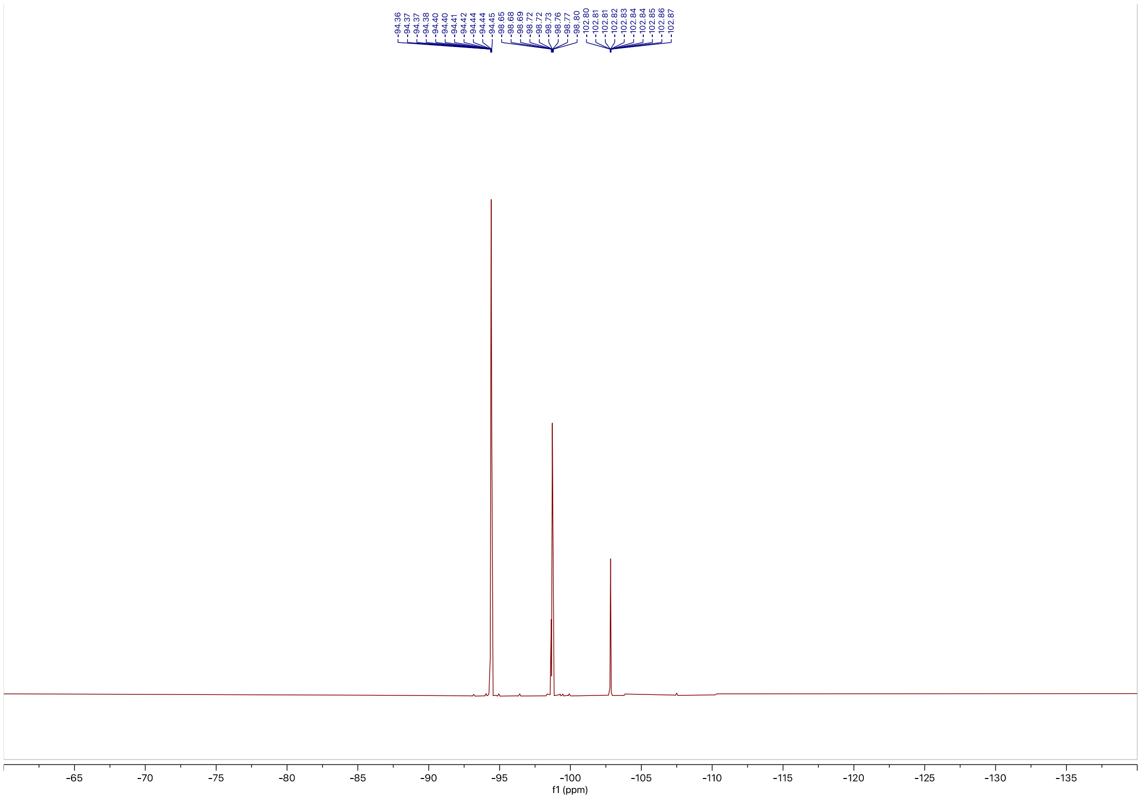

^{10}B-NMR (54 MHz, C_6D_6) spectra of **1g**

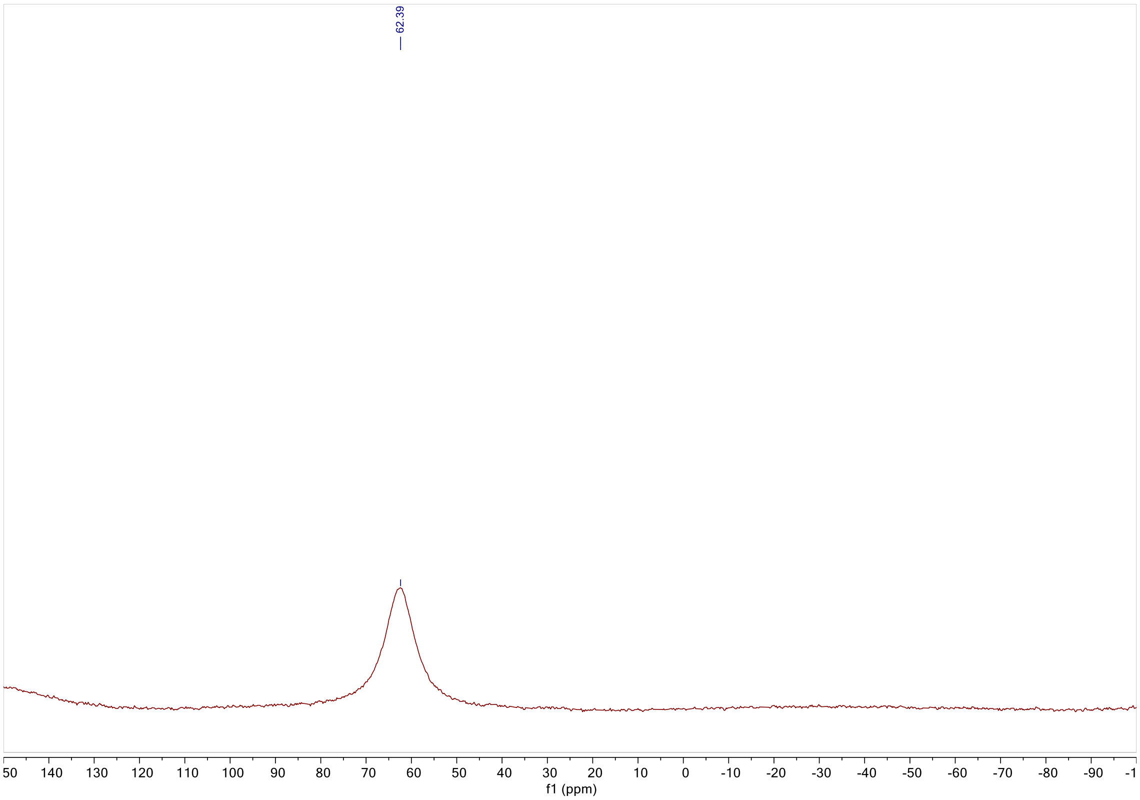

Compound 1h: (2-bromo-6-fluorophenyl)bis(2,6-difluorophenyl)borane (See compound data)

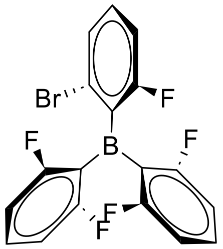

1h

^{1}H-NMR (500 MHz, C_6D_6) spectra of **1h**

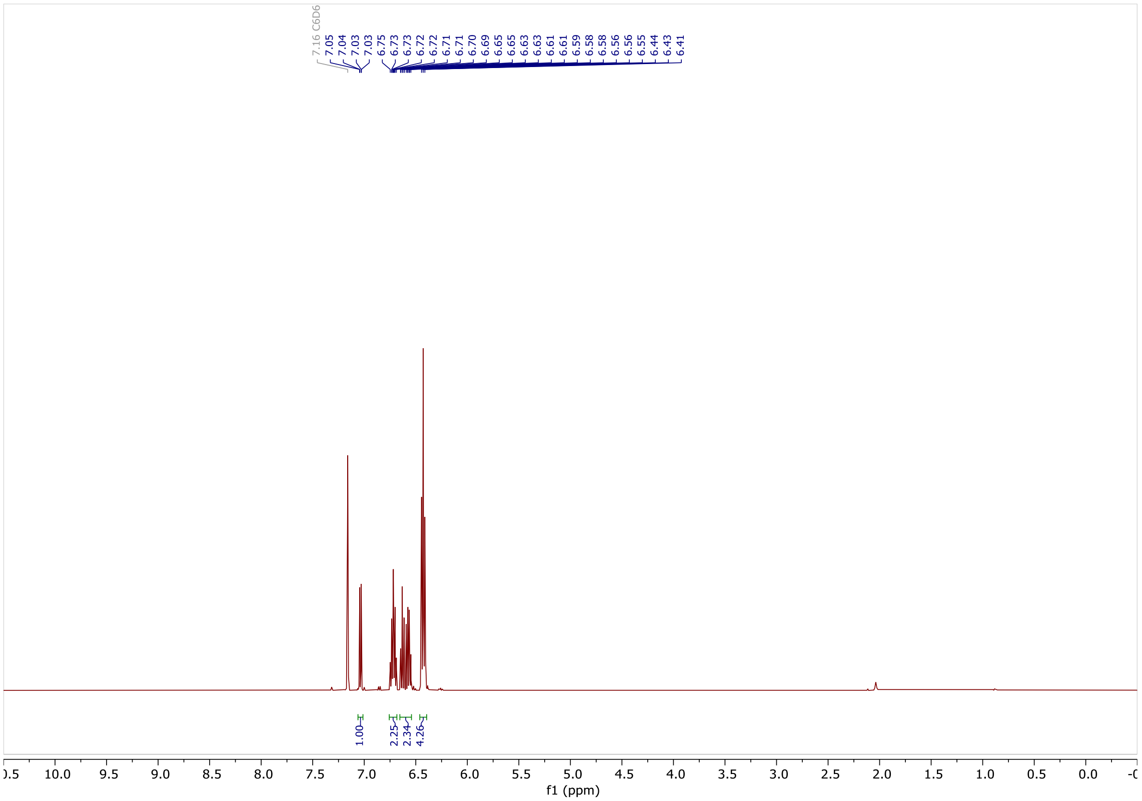

^{13}C-NMR (126 MHz, C_6D_6) spectra of **1h**

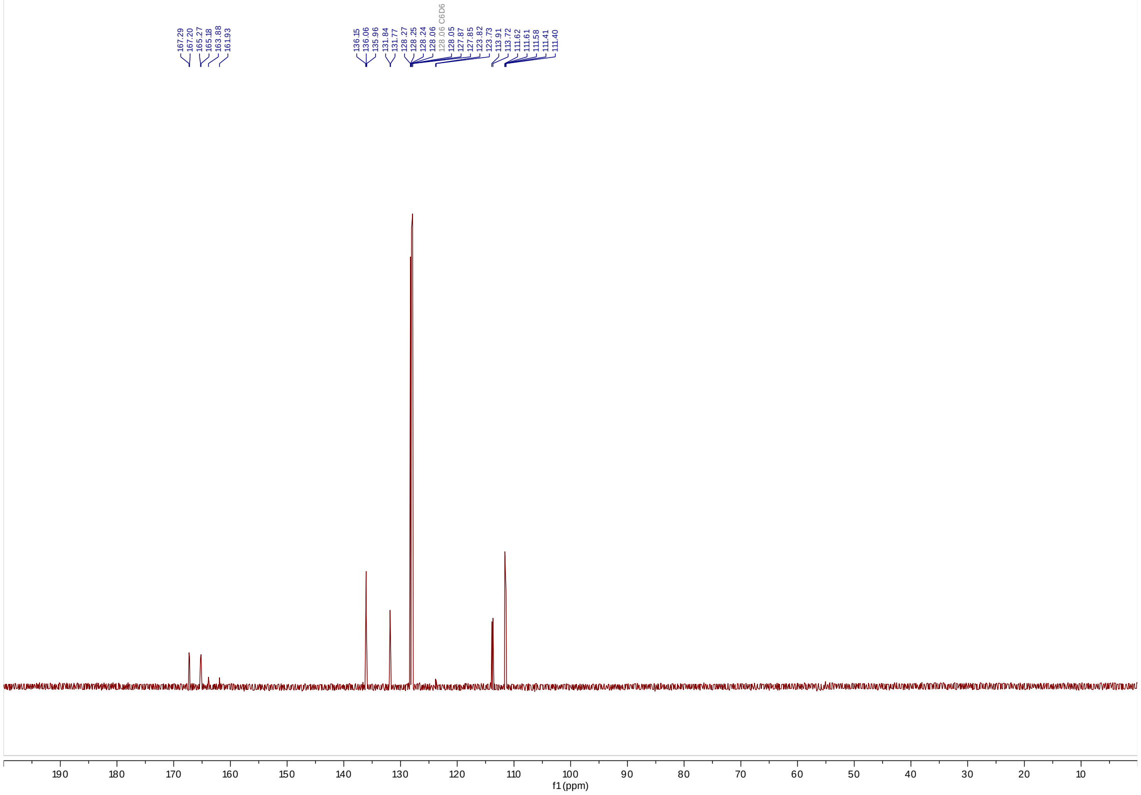

^{19}F-NMR (282 MHz, C_6D_6) spectra of **1h**

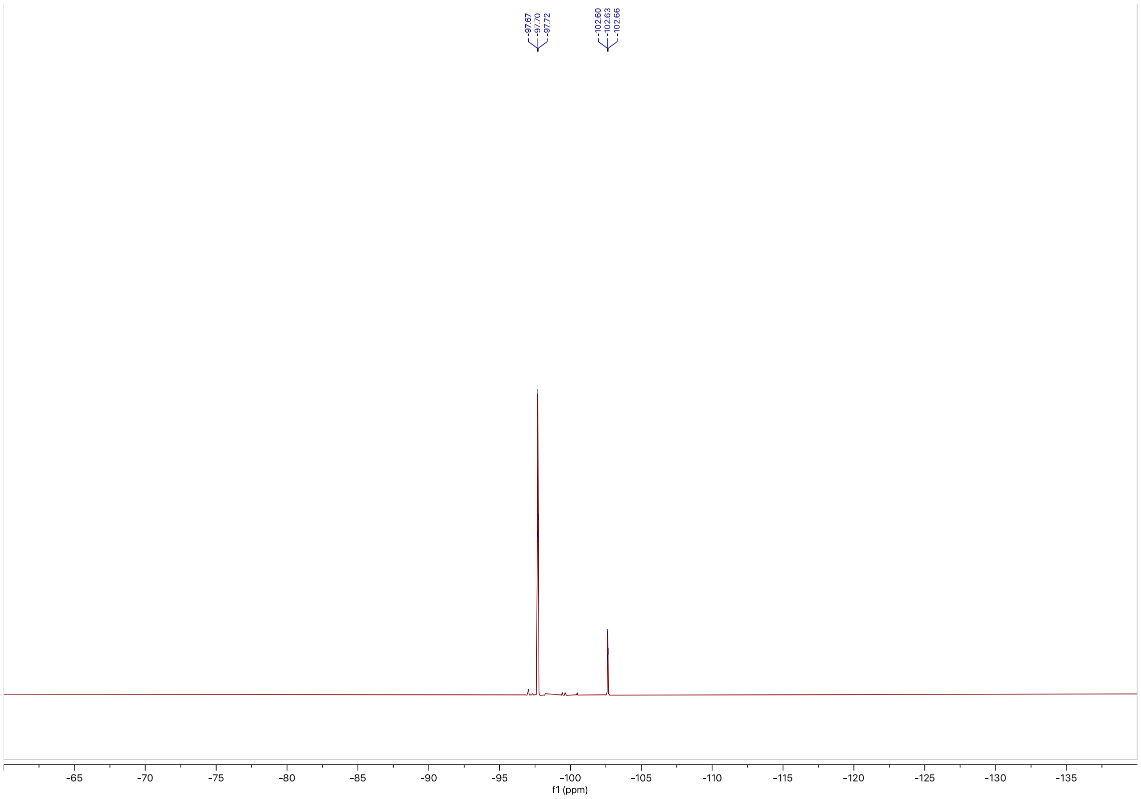

^{10}**B-NMR** (54 MHz, C_6D_6) spectra of **1h**

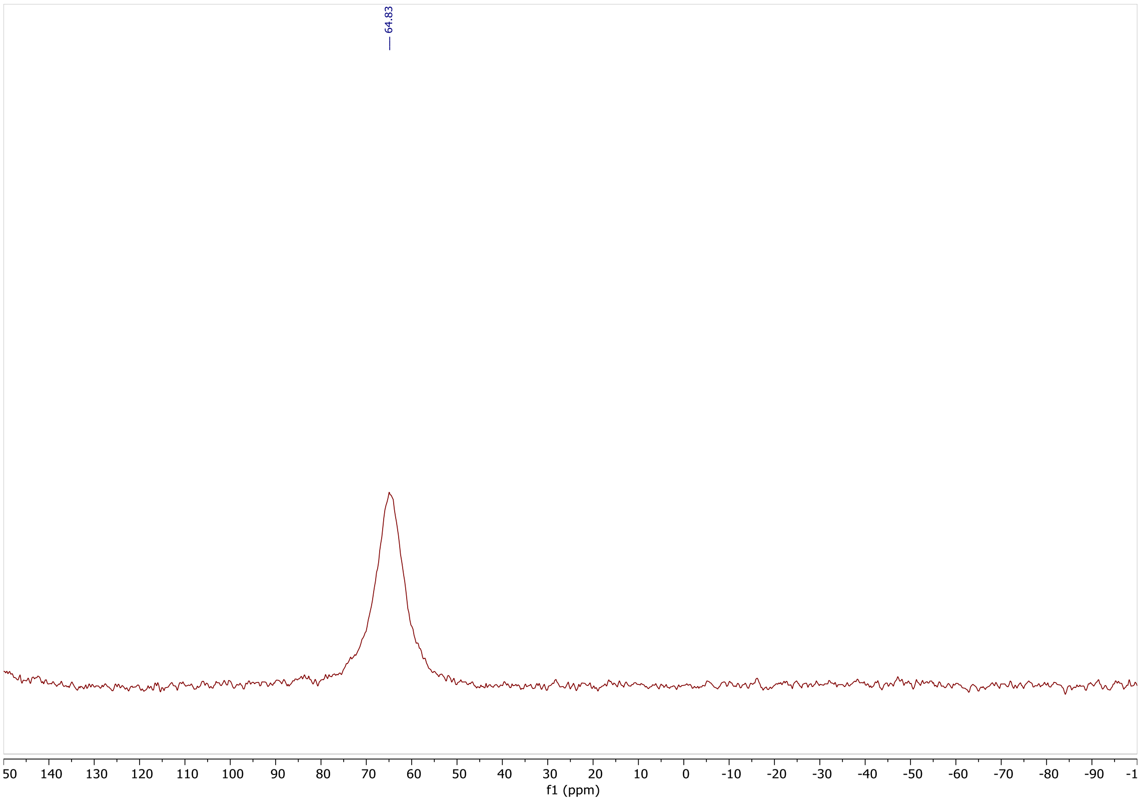

Compound S5: (2-chloro-6-fluorophenyl)bis(2,3,5,6-tetrafluorophenyl)borane (See compound data)

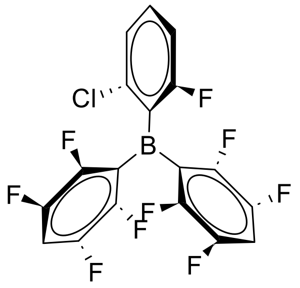

S5

^{1}H-NMR (500 MHz, C_6D_6) spectra of **S5**

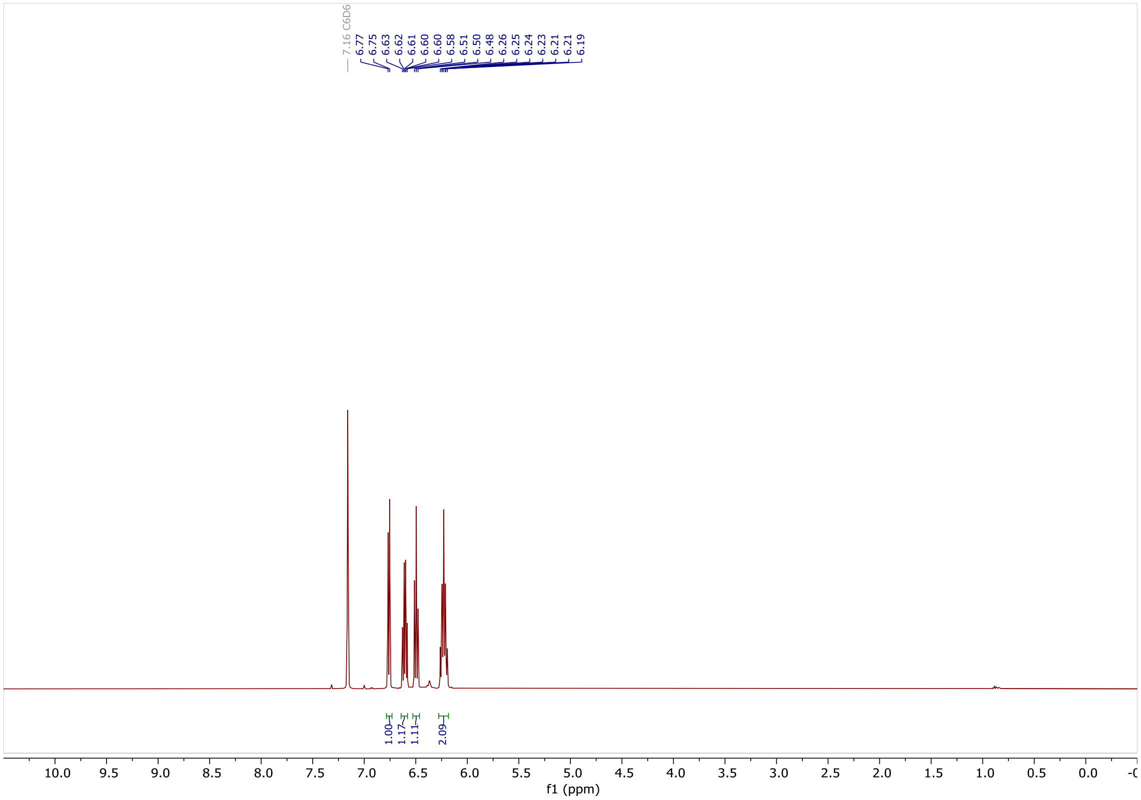

^{13}**C-NMR** (75 MHz, C_6D_6) spectra of **S5**

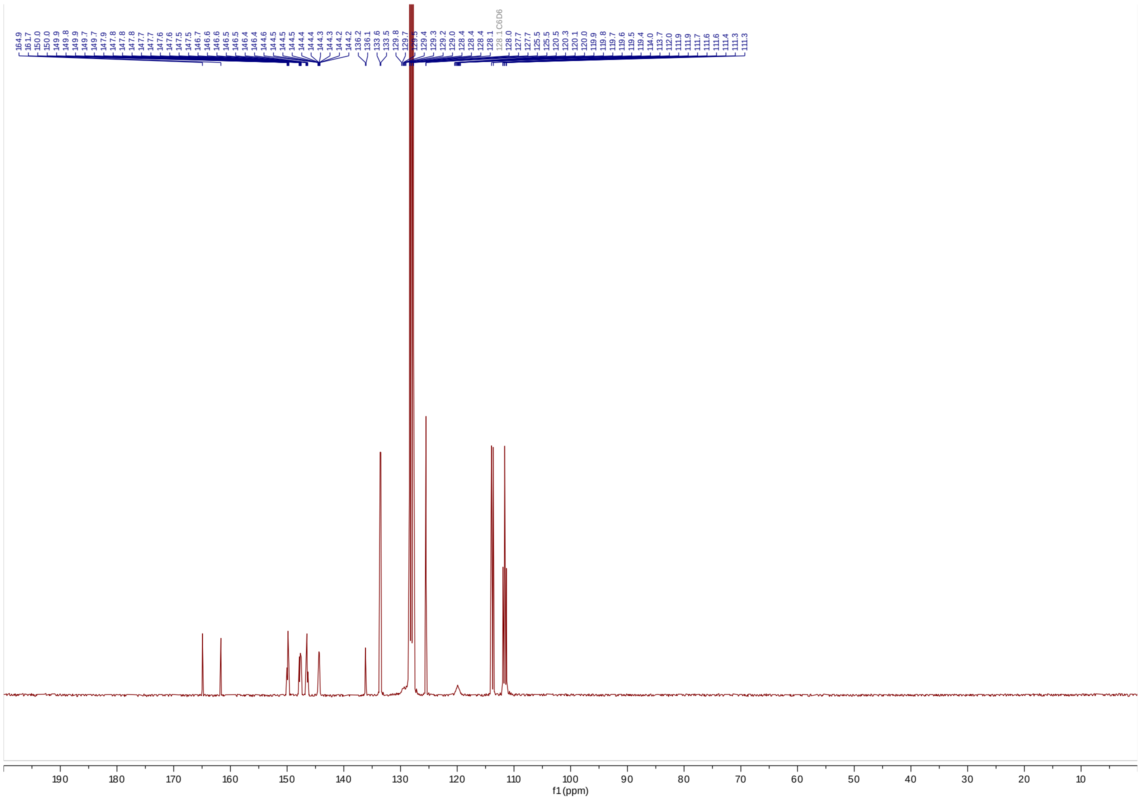

^{19}**F-NMR** (282 MHz, C_6D_6) spectra of **S5**

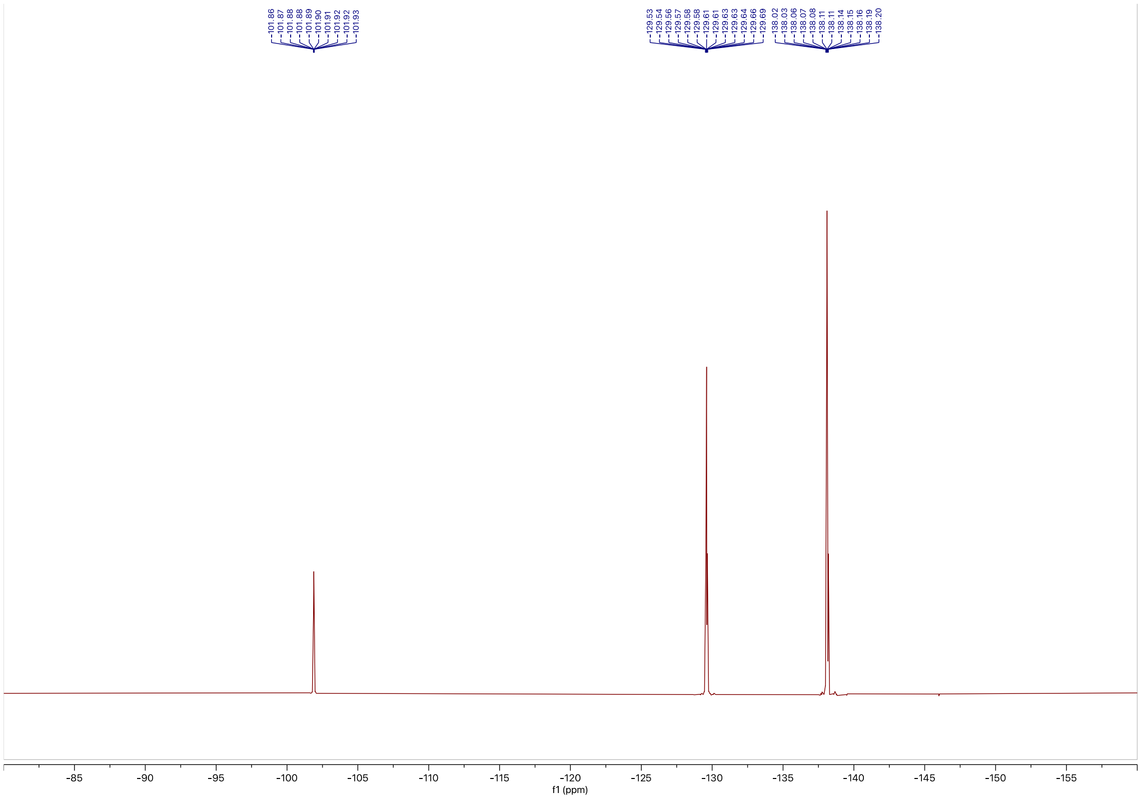

^{10}B-NMR (54 MHz, C_6D_6) spectra of **S5**

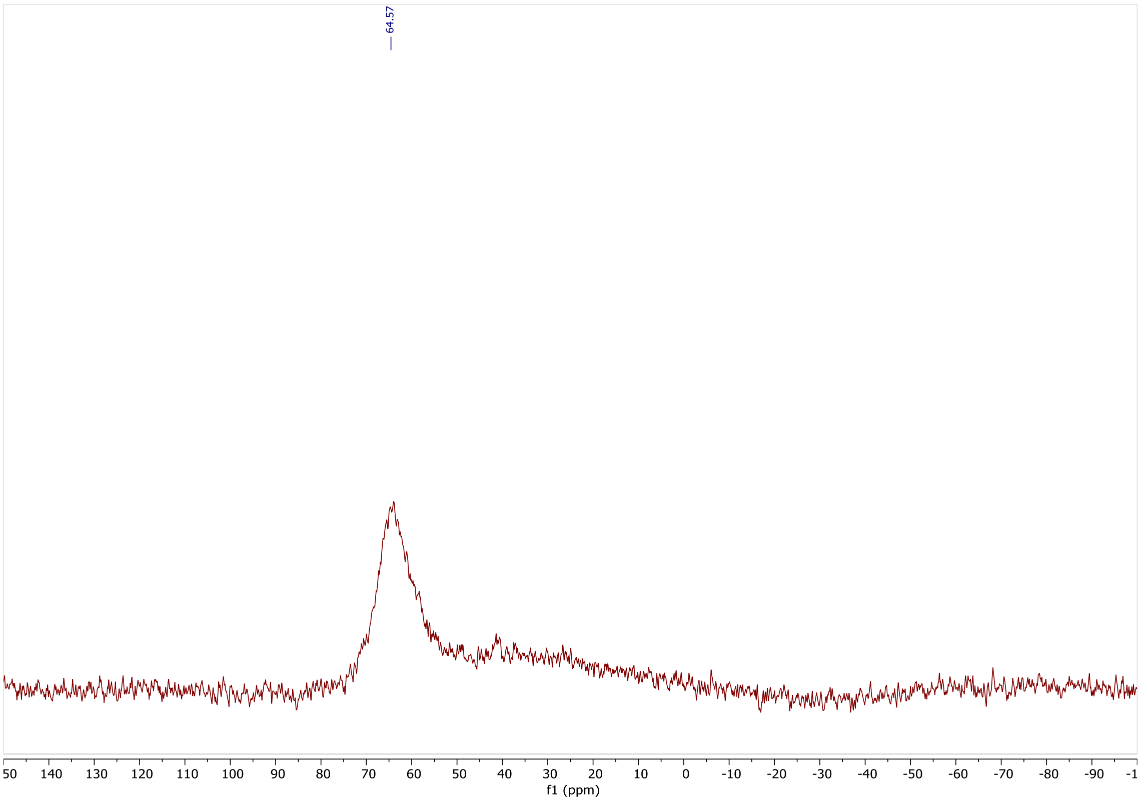

Compound S6: (2,6-dibromophenyl)bis(perfluorophenyl)borane (See compound data)

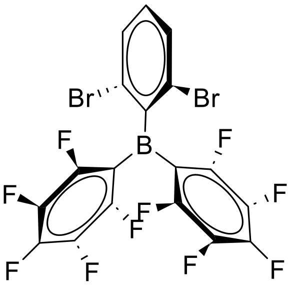

S6

^{1}H-NMR (500 MHz, C_6D_6) spectra of **S6**

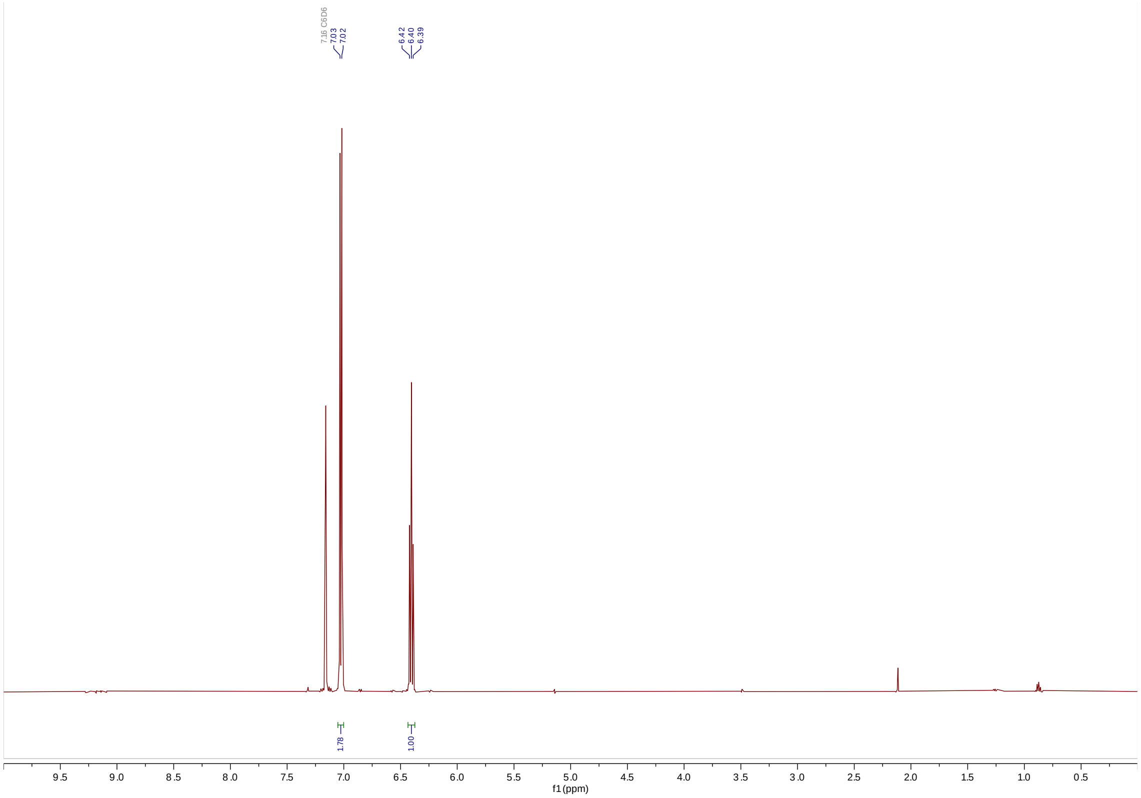

^{13}C-NMR (126 MHz, C_6D_6) spectra of **S6**

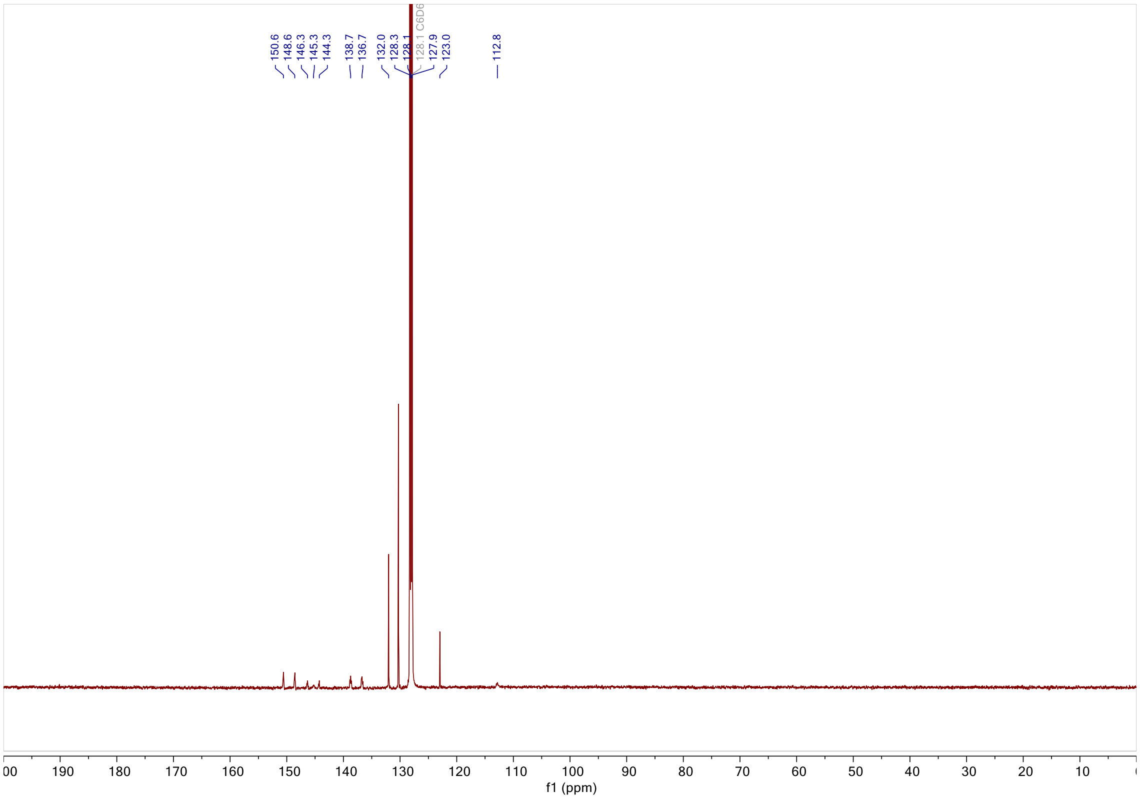

^{19}F-NMR (282 MHz, C_6D_6) spectra of **S6**

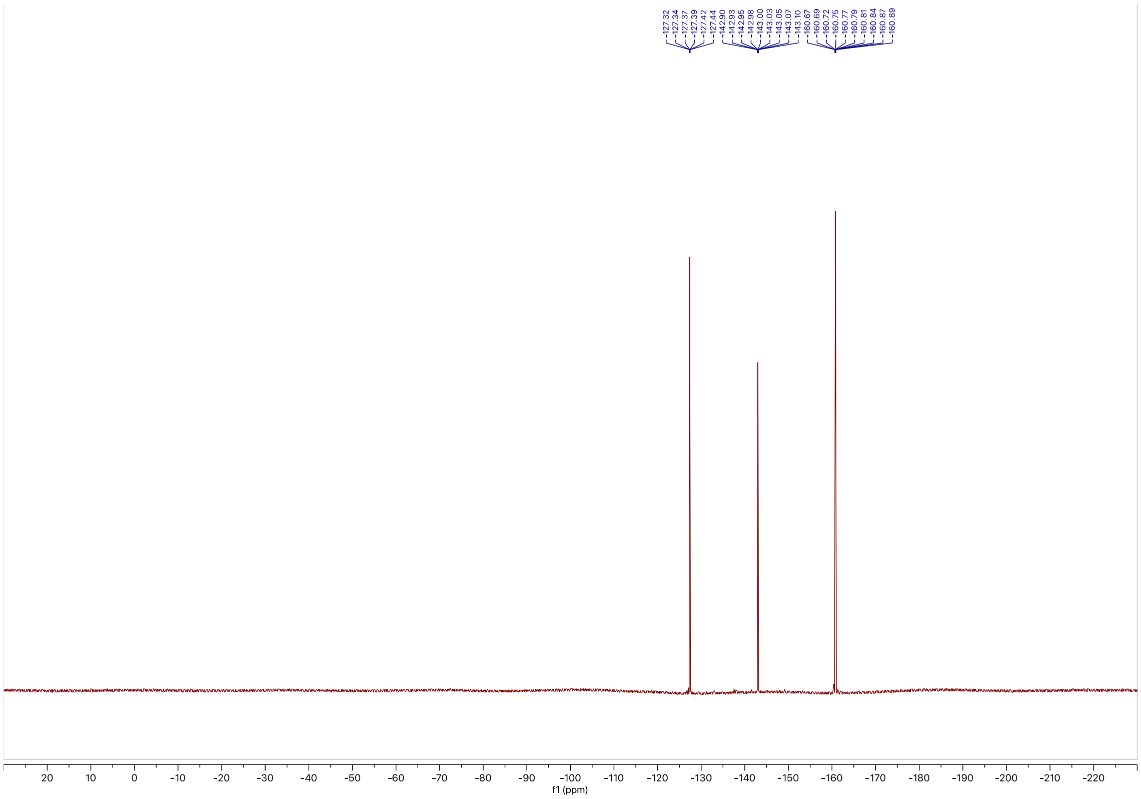

^{10}B-NMR (54 MHz, C_6D_6) spectra of **S6**

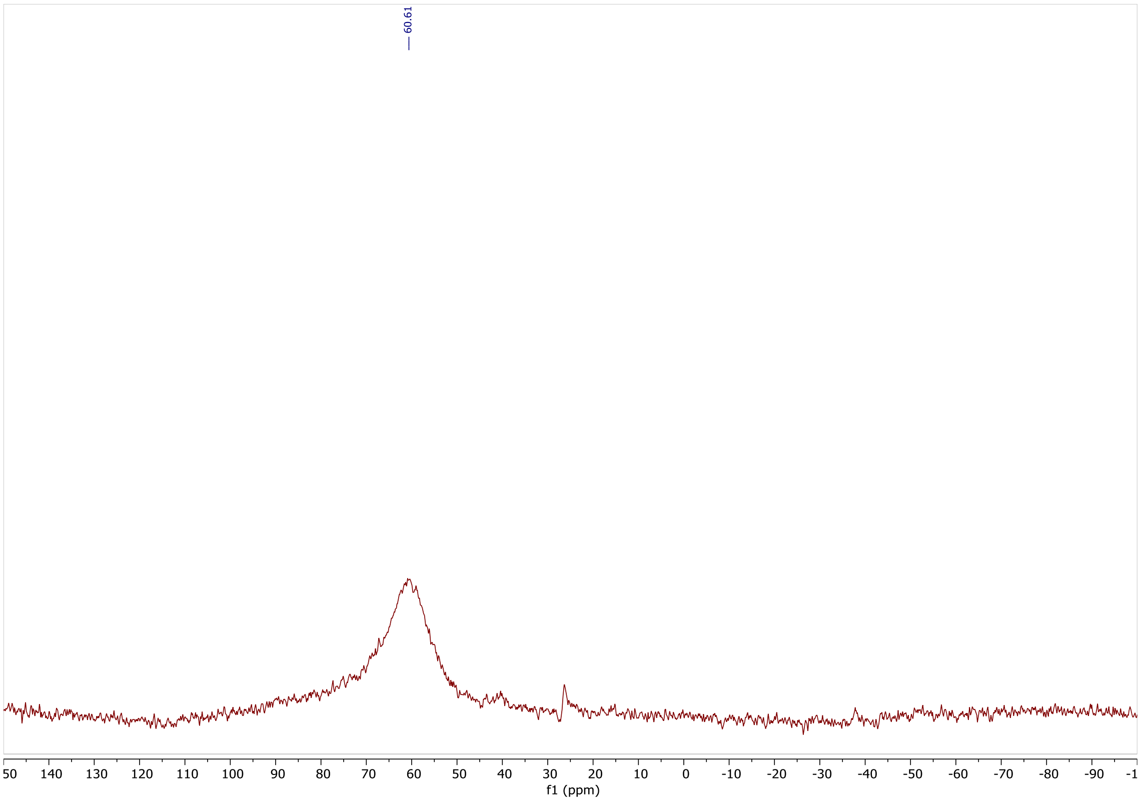

Compound 4: triethyl(1-methoxy-3-phenylpropoxy)silane (See compound data)

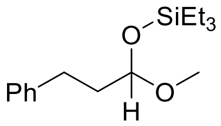

4

^{1}H-NMR (500 MHz, $CDCl_3$) spectra of **4**

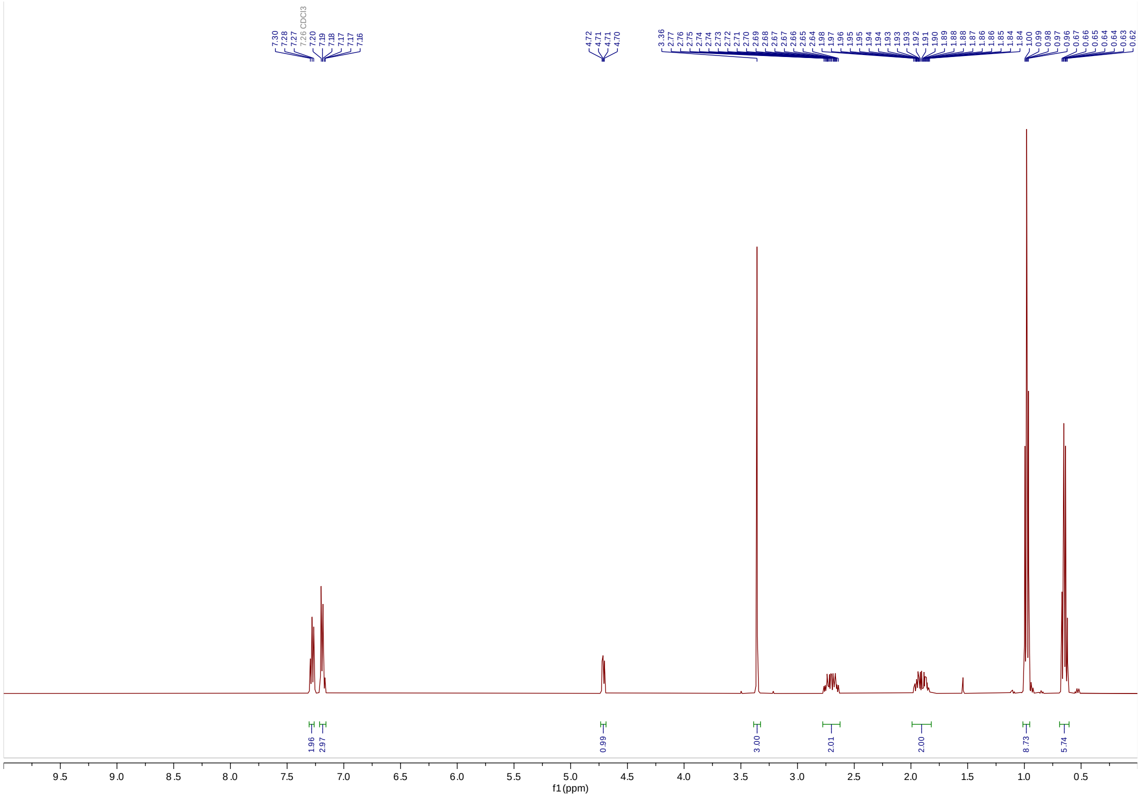

^{13}**C-NMR** (126 MHz, $CDCl_3$) spectra of **4**

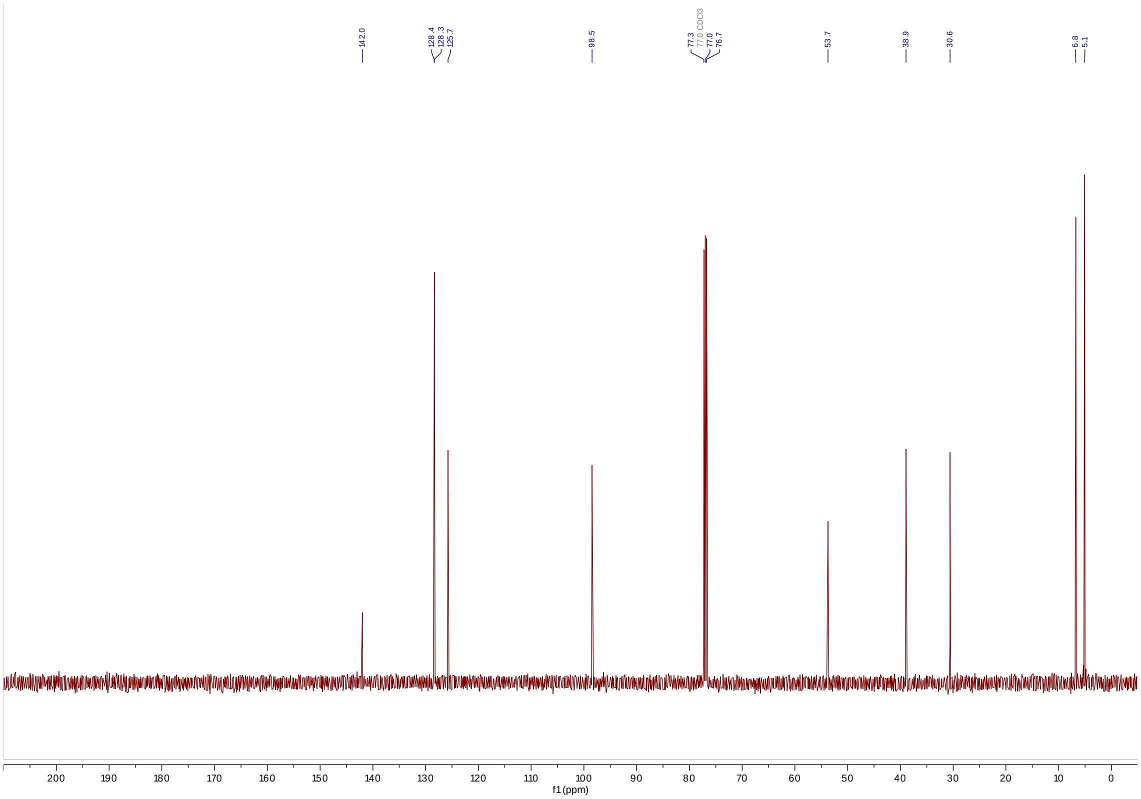

Compound 7: (1-ethoxy-3-phenylpropoxy)triethylsilane (See compound data)

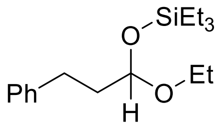

7

^{1}H-NMR (500 MHz, $CDCl_3$) spectra of **7**

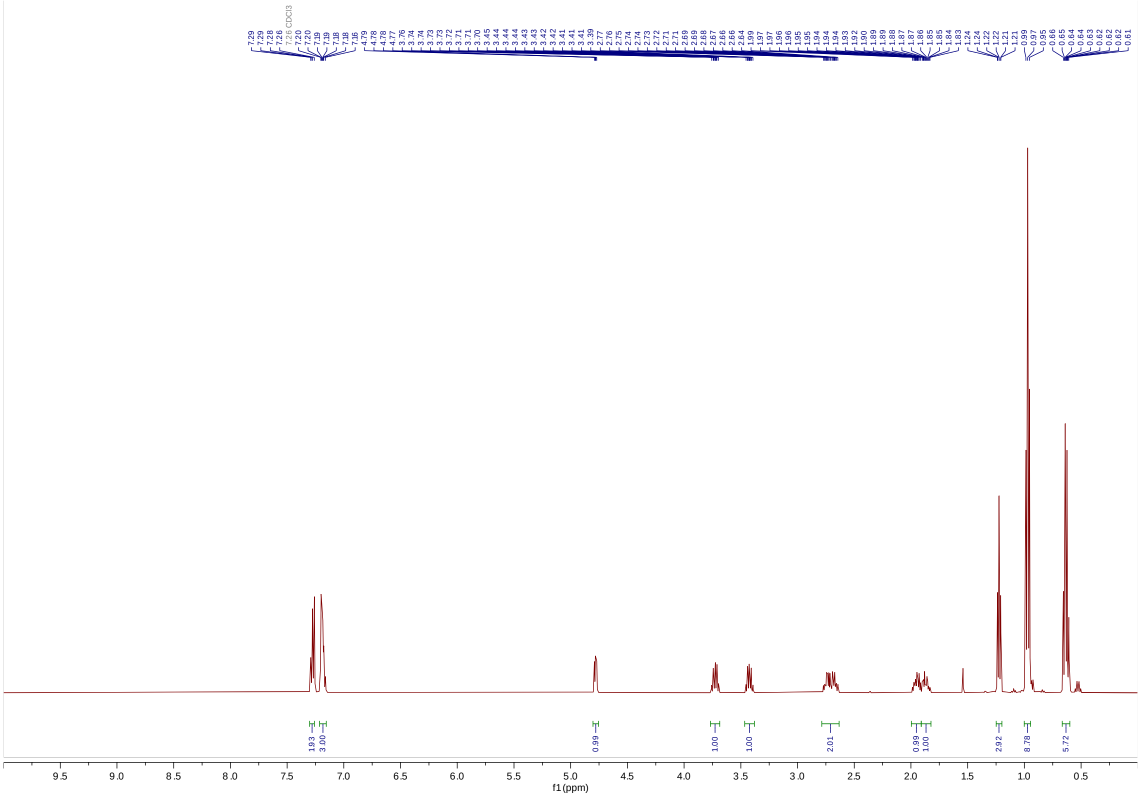

^{13}**C-NMR** (126 MHz, $CDCl_3$) spectra of **7**

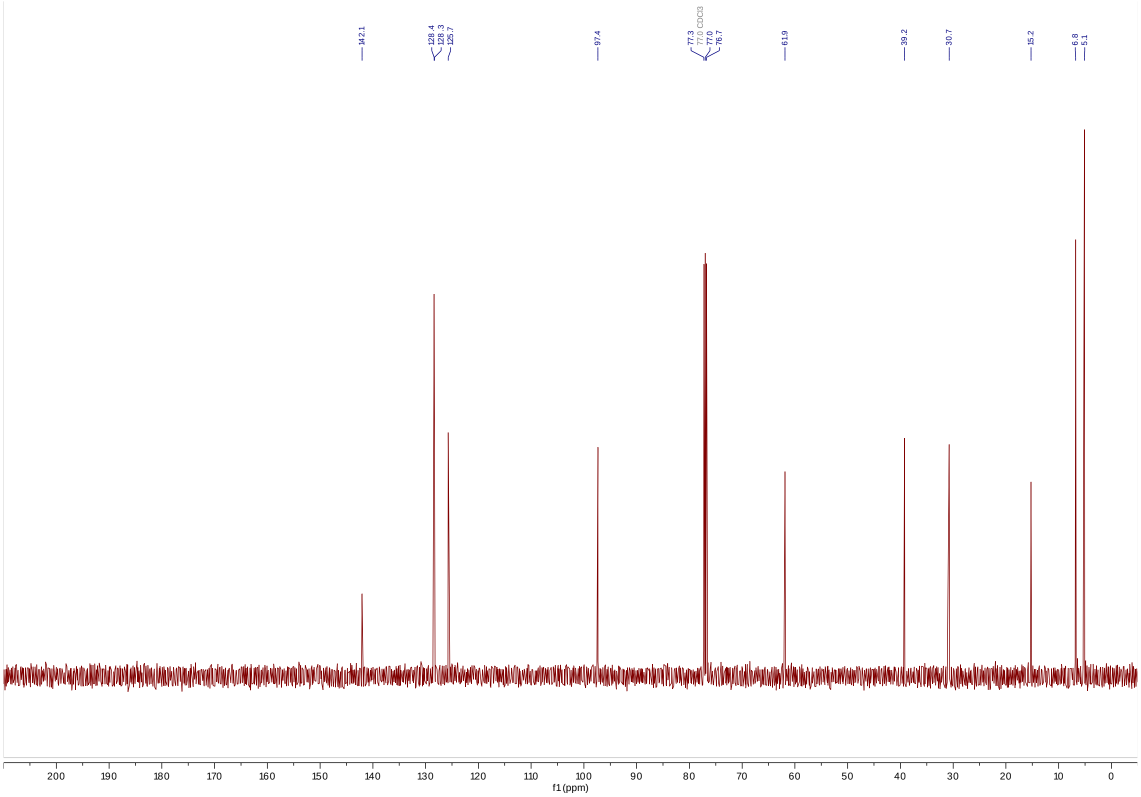

Compound 8: (1-ethoxy-3-phenylpropoxy)triethylsilane (See compound data)

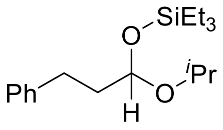

8

^{1}H-NMR (500 MHz, C_6D_6) spectra of **8**

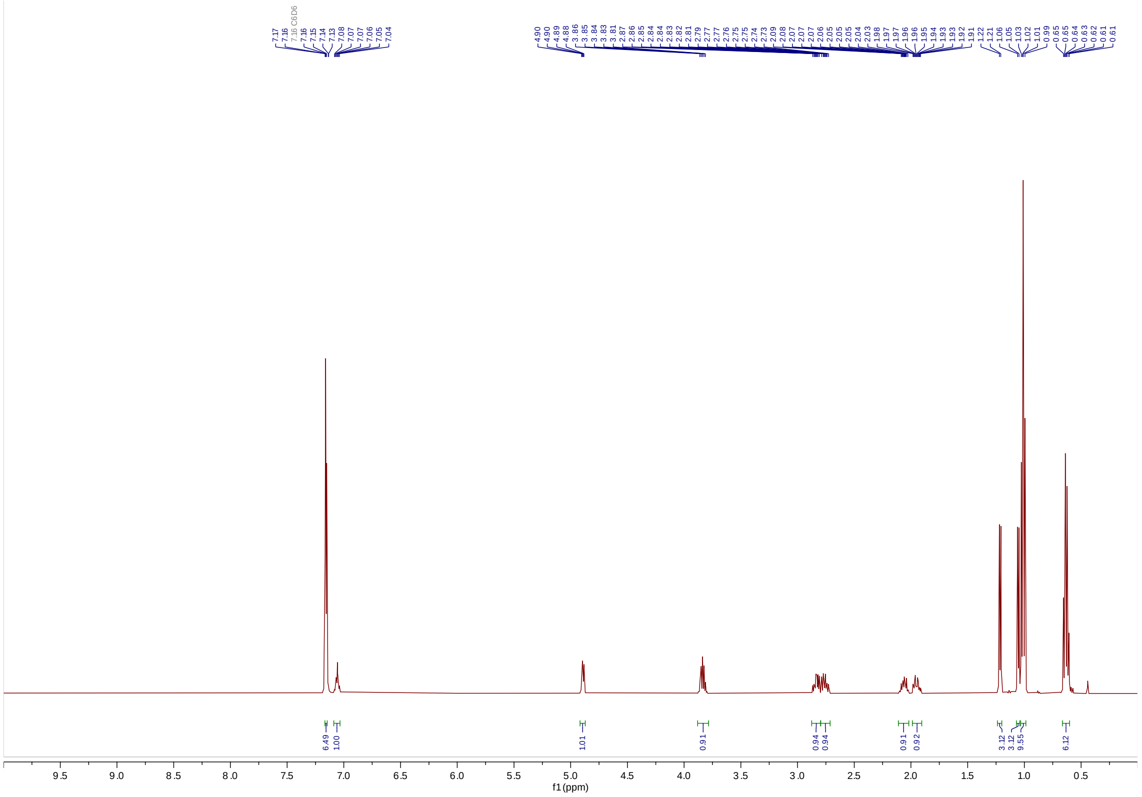

^{13}C-NMR (126 MHz, C_6D_6) spectra of **8**

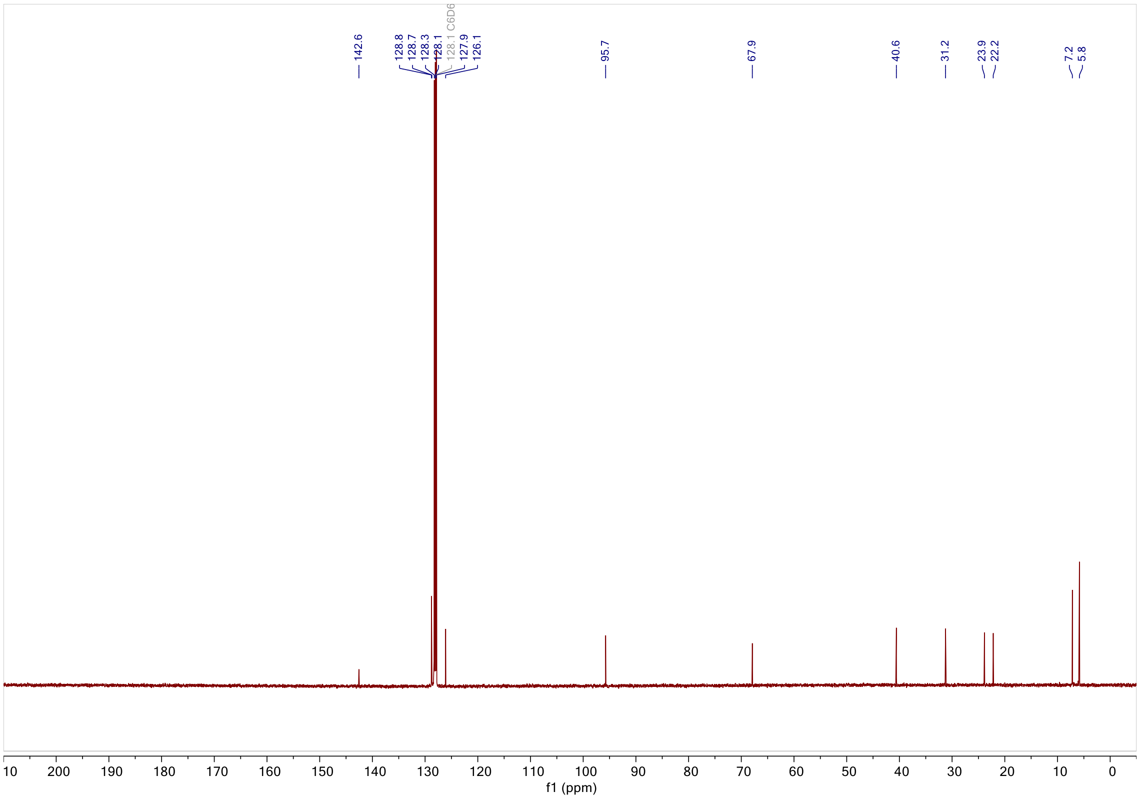

Compound 9: (1-(benzyloxy)-3-phenylpropoxy)triethylsilane (See compound data)

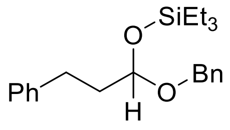

9

^{1}H-NMR (500 MHz, $CDCl_3$) spectra of **9**

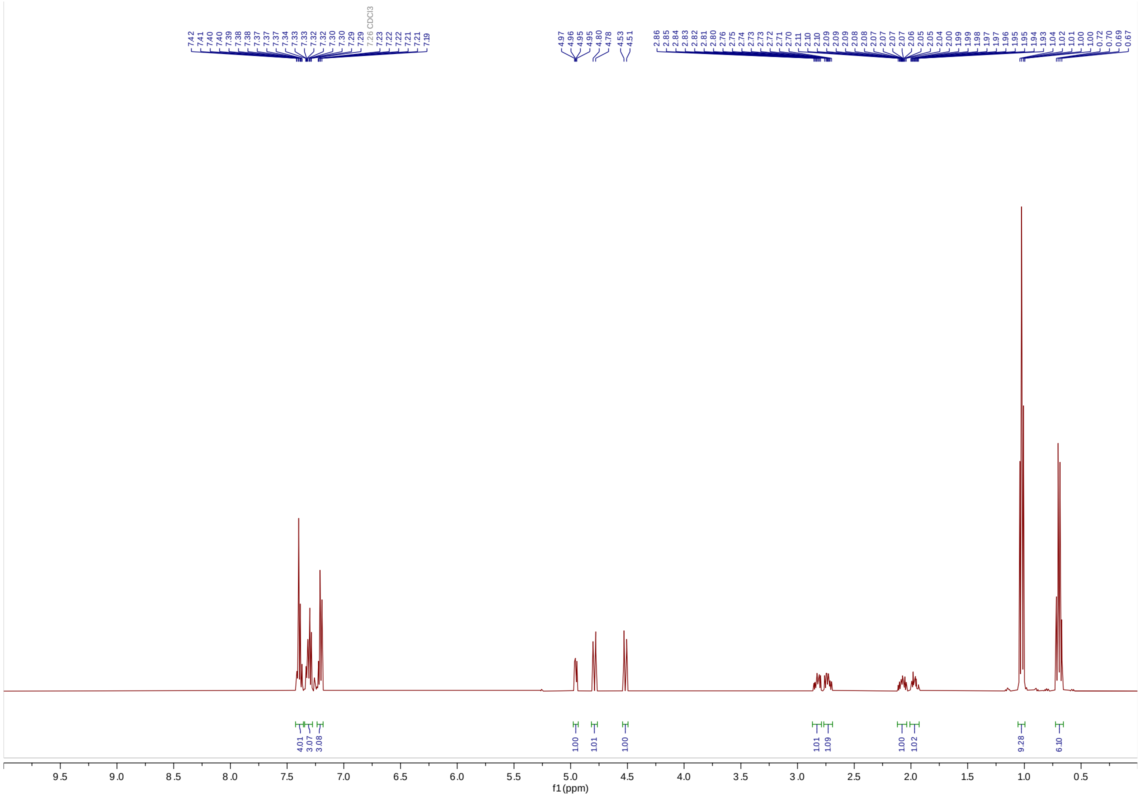

^{13}**C-NMR** (126 MHz, $CDCl_3$) spectra of **9**

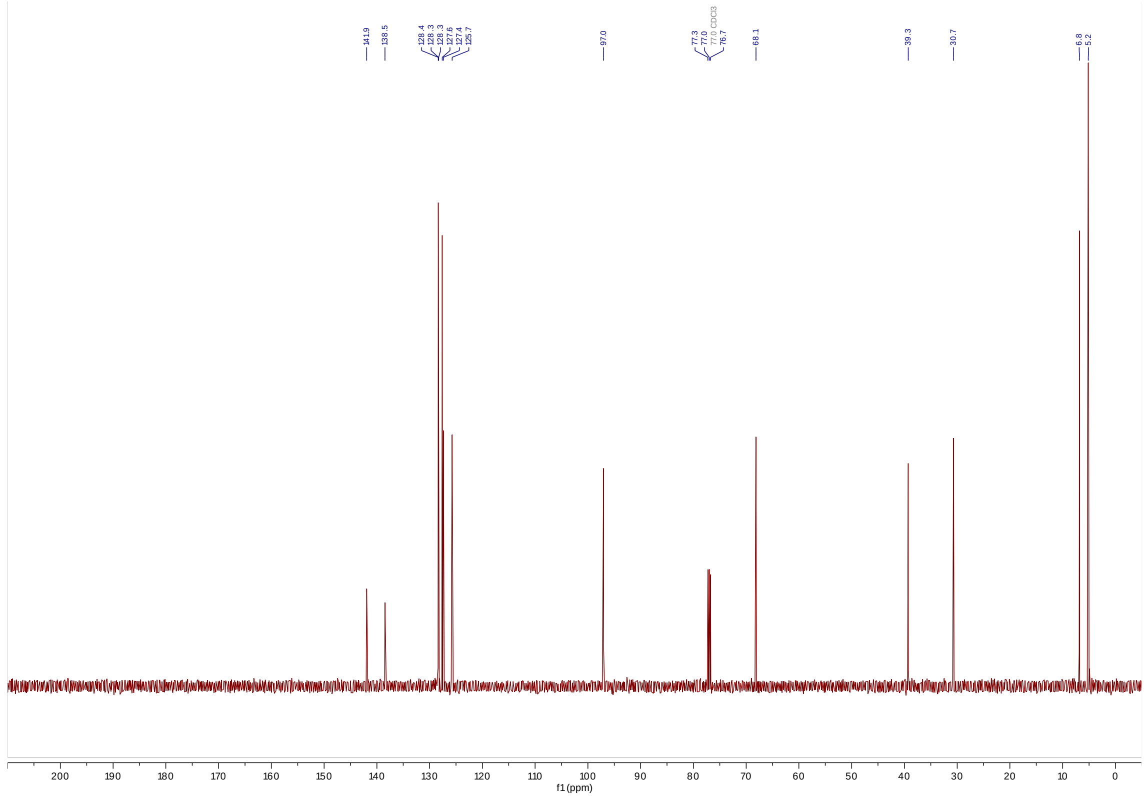

Compound 10: 3,3,7,7-tetraethyl-5-phenethyl-4,6-dioxa-3,7-disilanonane (See compound data)

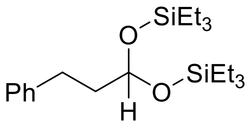

10

^{1}H-NMR (500 MHz, $CDCl_3$) spectra of **10**

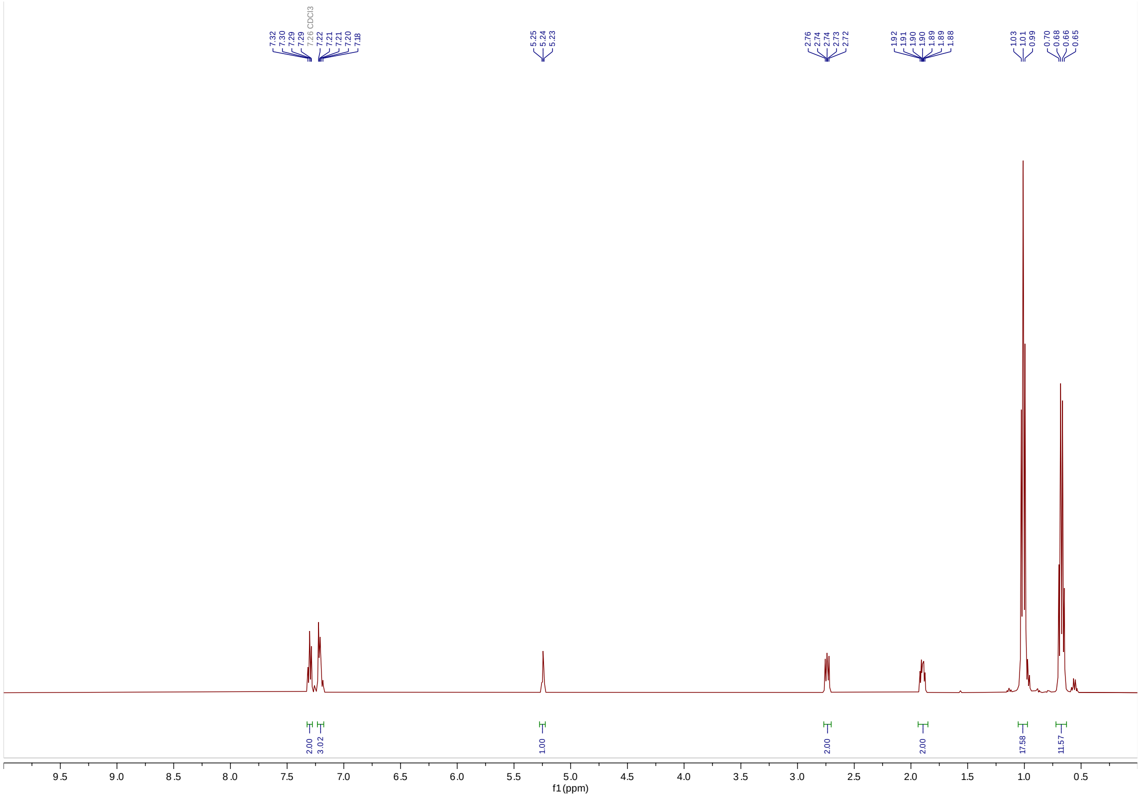

^{13}C-NMR (126 MHz, $CDCl_3$) spectra of **10**

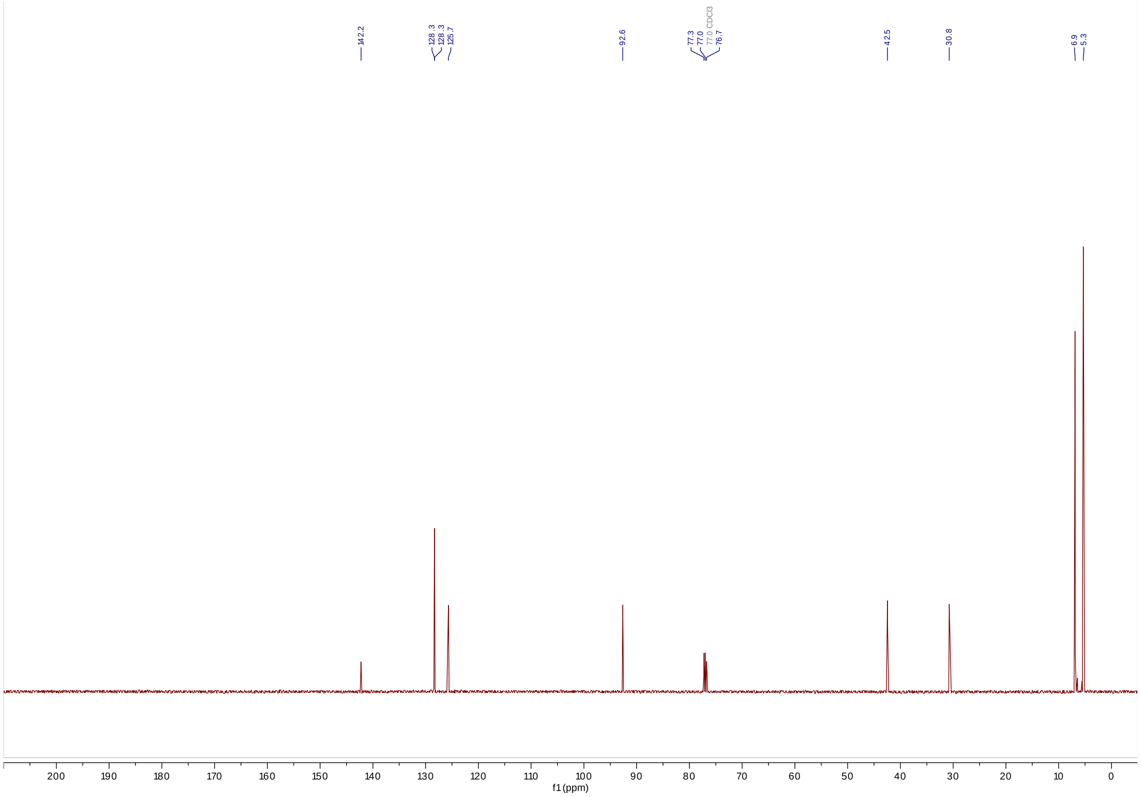

Compound 11: triethyl(methoxy(phenyl)methoxy)silane (See compound data)

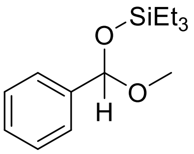

11

^{1}H-NMR (500 MHz, C_6D_6) spectra of **11**

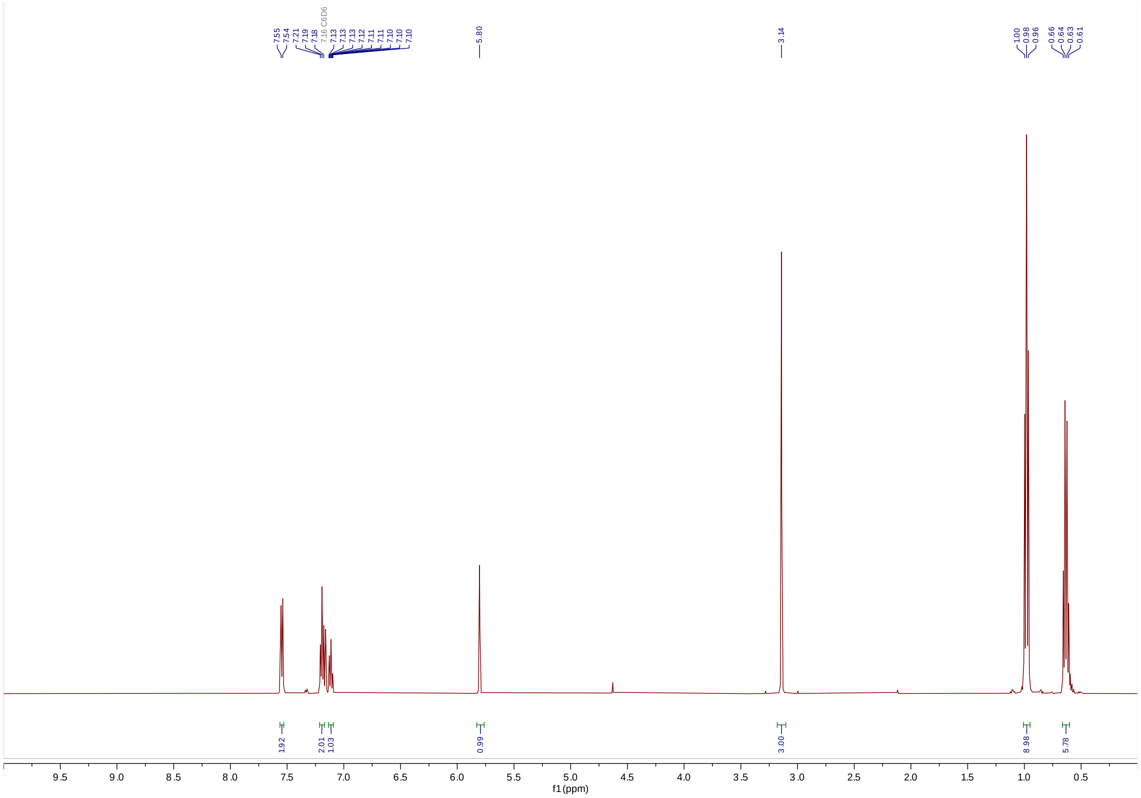

^{13}**C-NMR** (126 MHz, C_6D_6) spectra of **11**

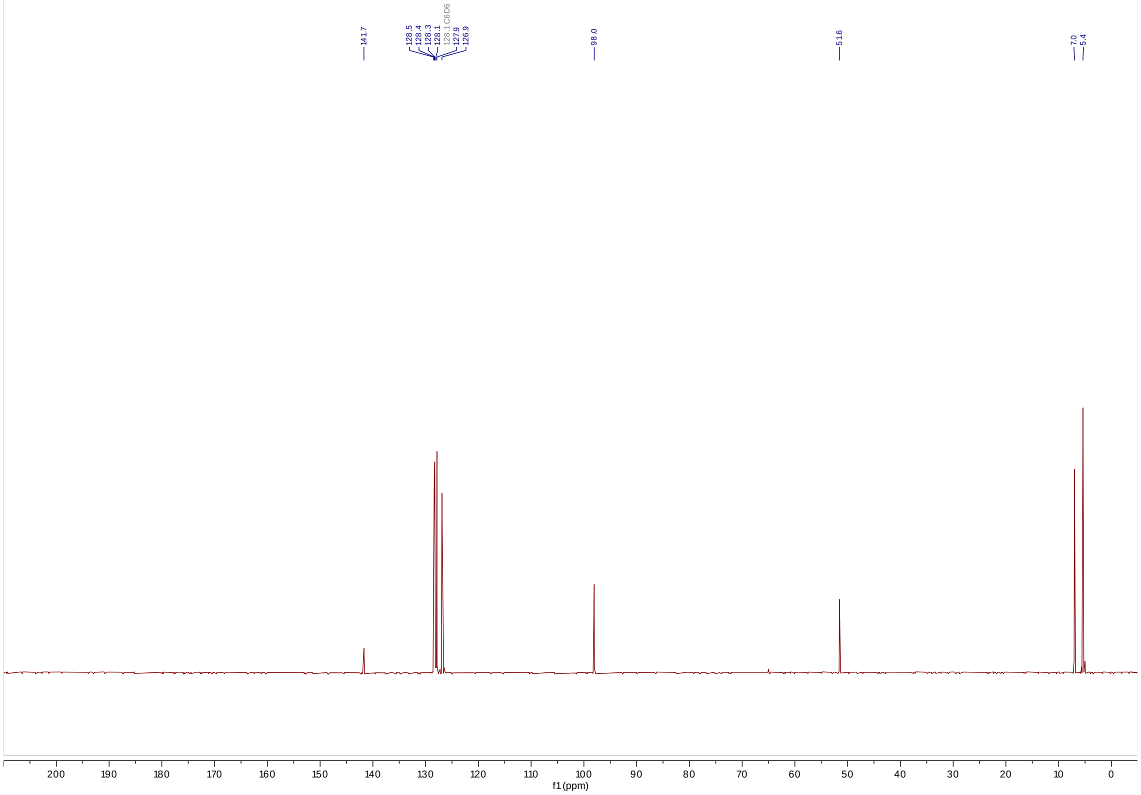

Compound 12: triethyl(methoxy(p-tolyl)methoxy)silane (See compound data)

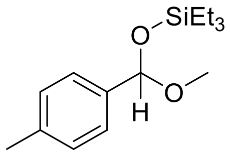

12

^{1}H-NMR (500 MHz, C_6D_6) spectra of **12**

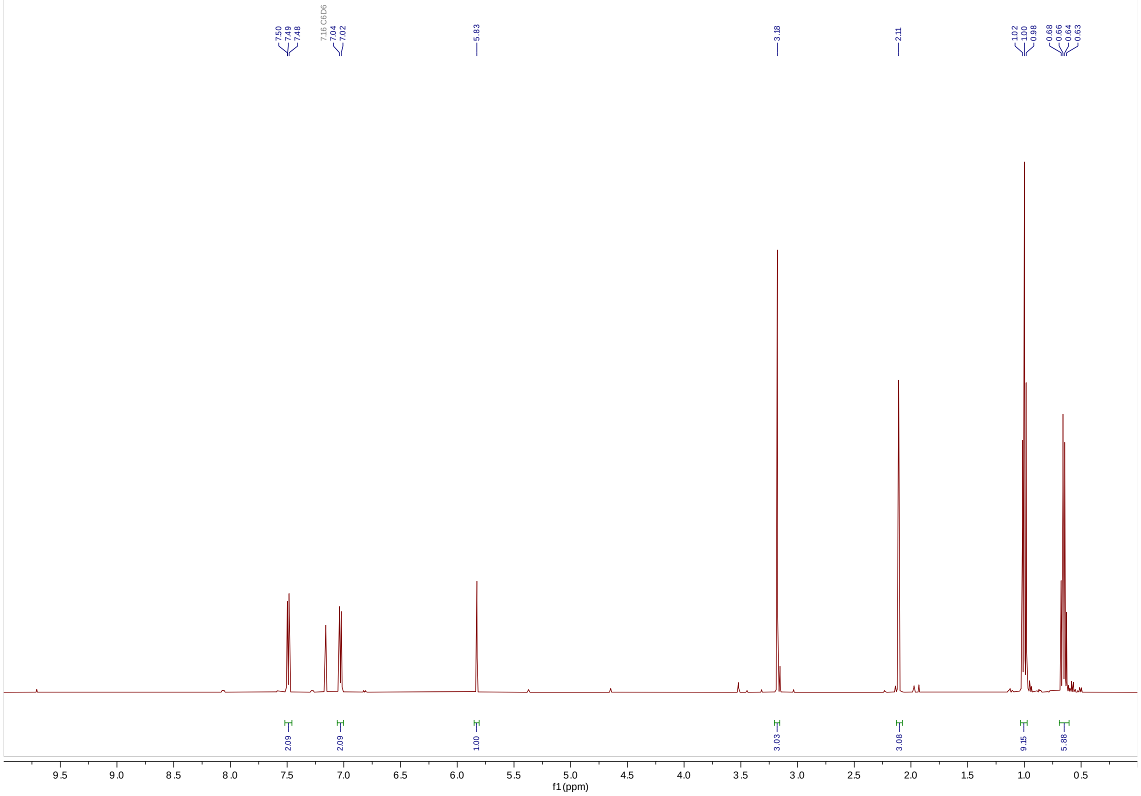

^{13}**C-NMR** (126 MHz, C_6D_6) spectra of **12**

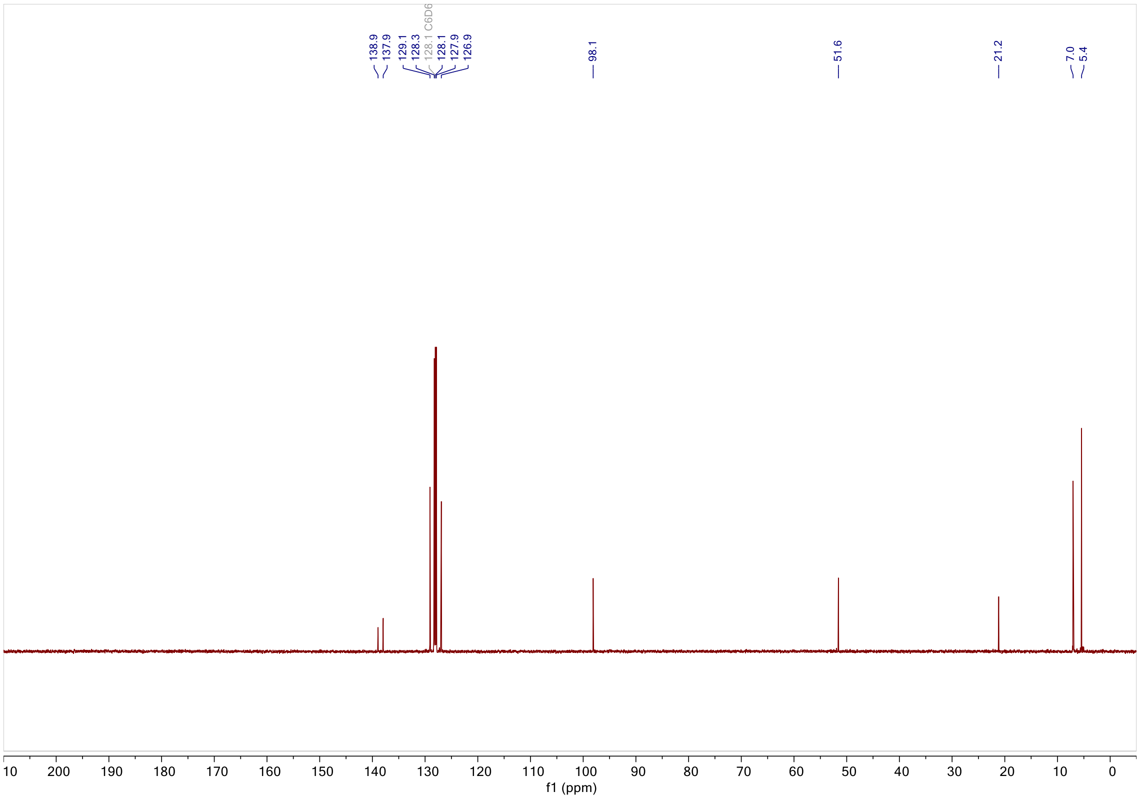

Compound 13: triethyl(methoxy(m-tolyl)methoxy)silane (See compound data)

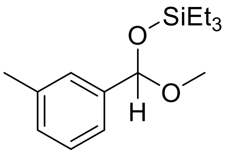

13

[1]**H-NMR** (500 MHz, C_6D_6) spectra of **13**

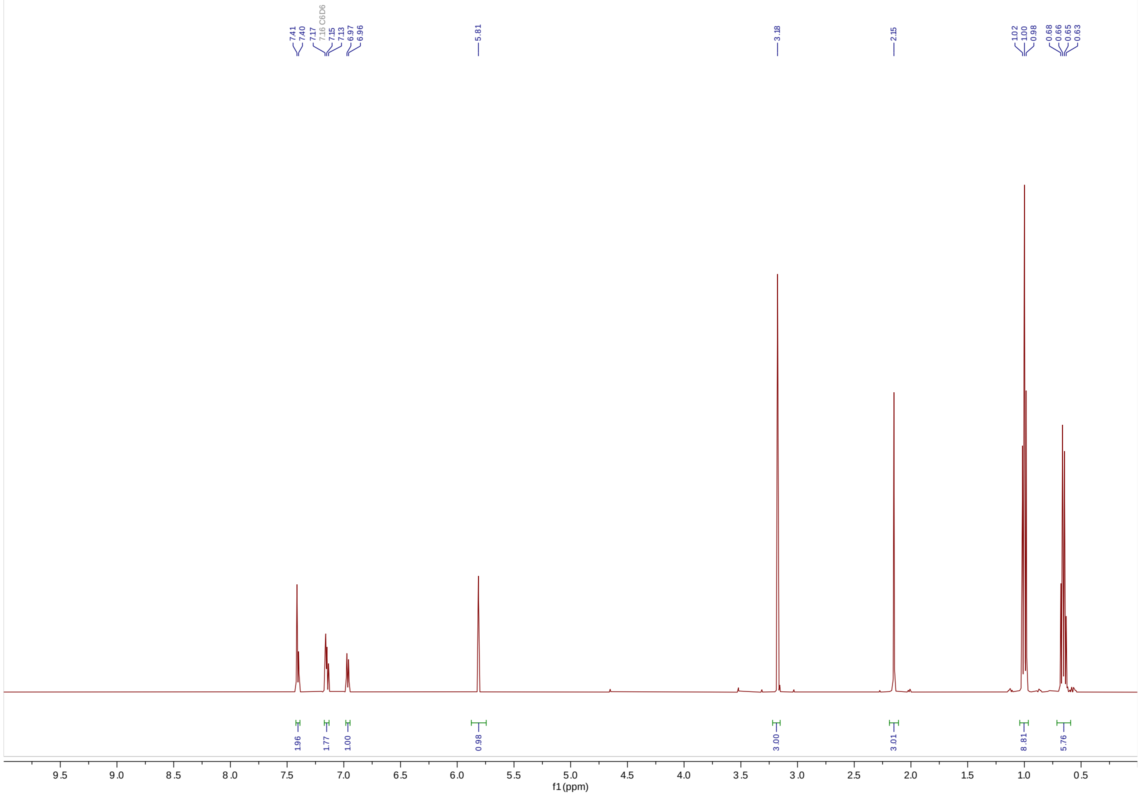

^{13}C-NMR (126 MHz, C_6D_6) spectra of **13**

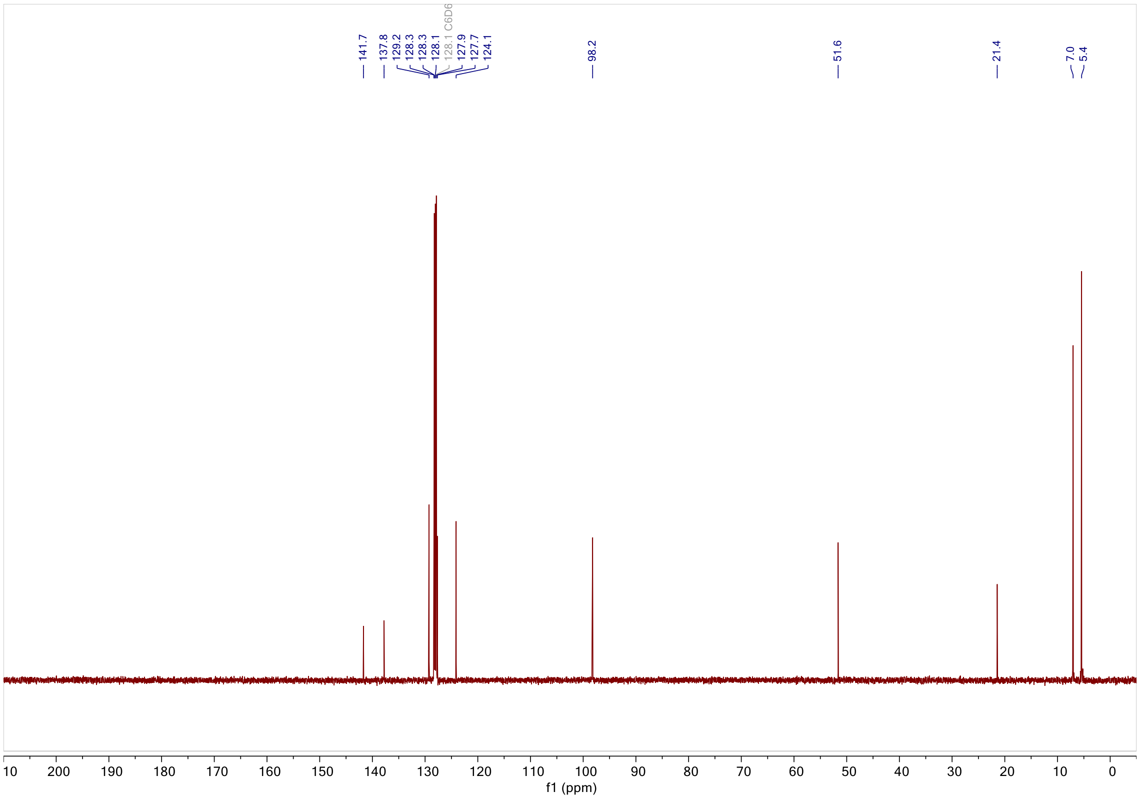

Compound 14: triethyl(methoxy(o-tolyl)methoxy)silane (See compound data)

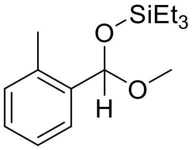

14

^{1}H-NMR (500 MHz, C_6D_6) spectra of **14**

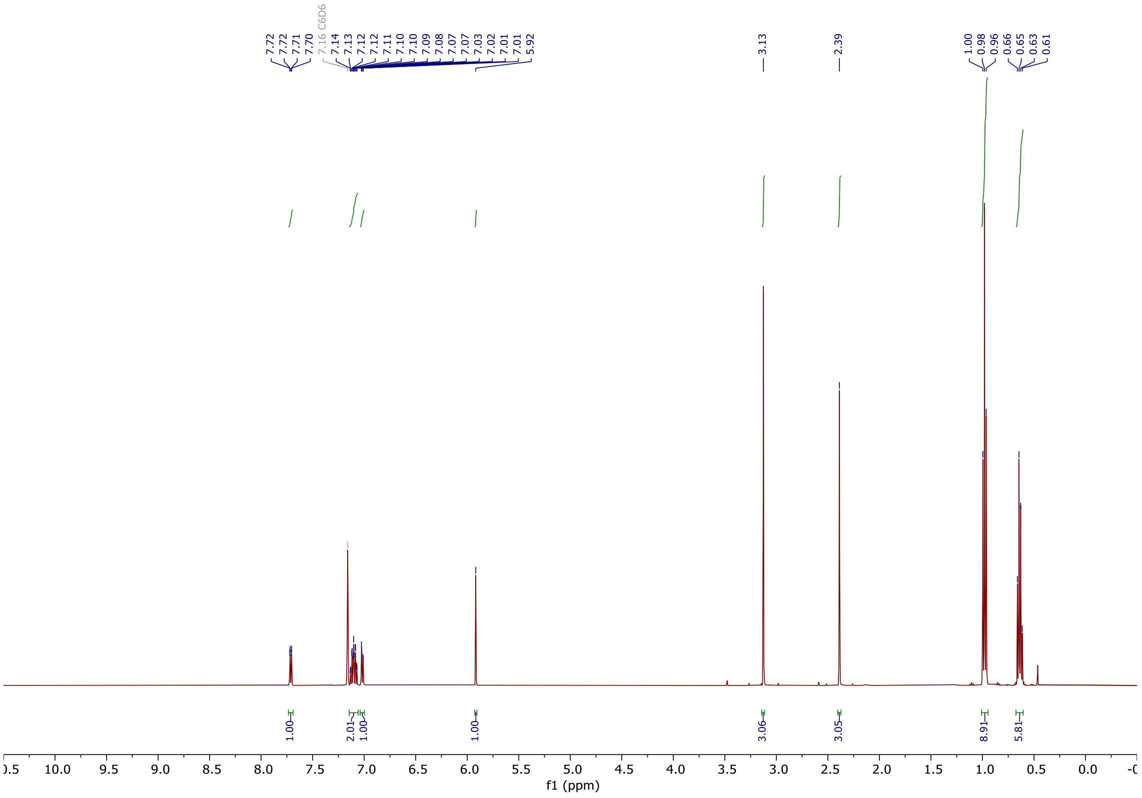

^{13}**C-NMR** (126 MHz, C_6D_6) spectra of **14**

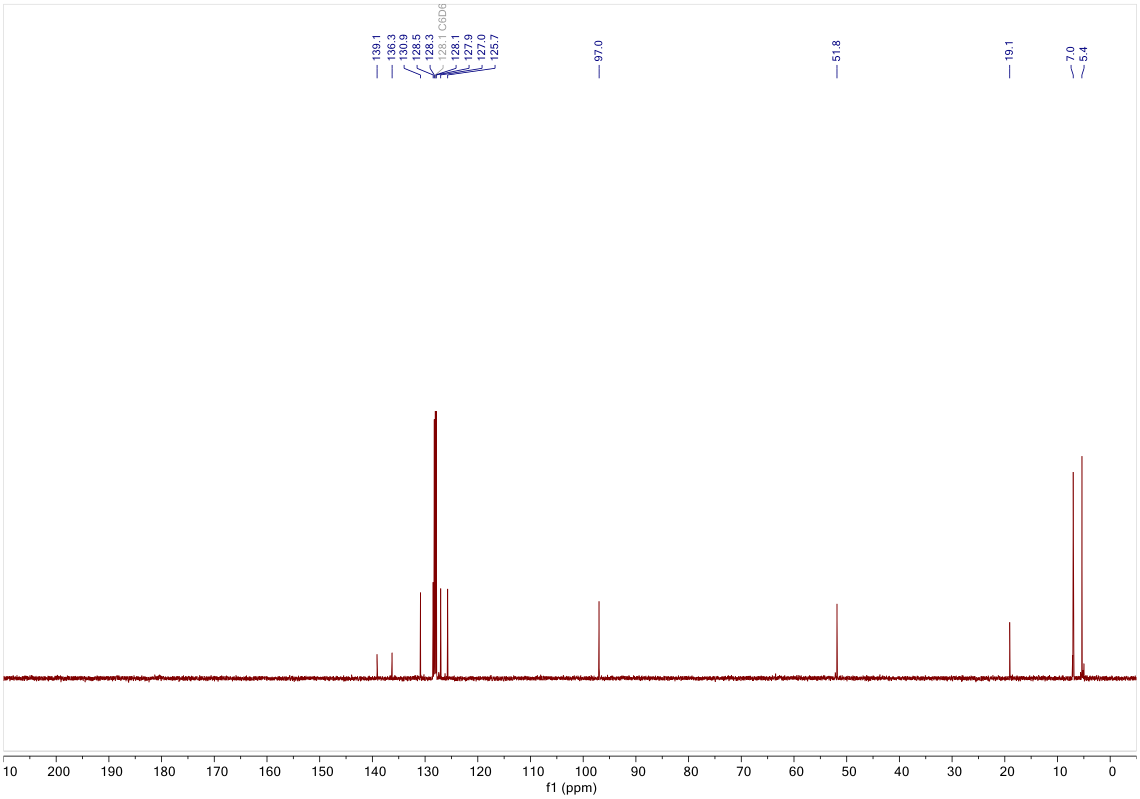

Compound 15: triethyl(methoxy(4-methoxyphenyl)methoxy)silane (See compound data)

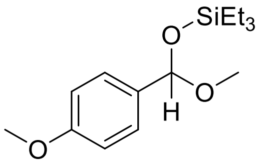

15

^{1}H-NMR (500 MHz, C_6D_6) spectra of **15**

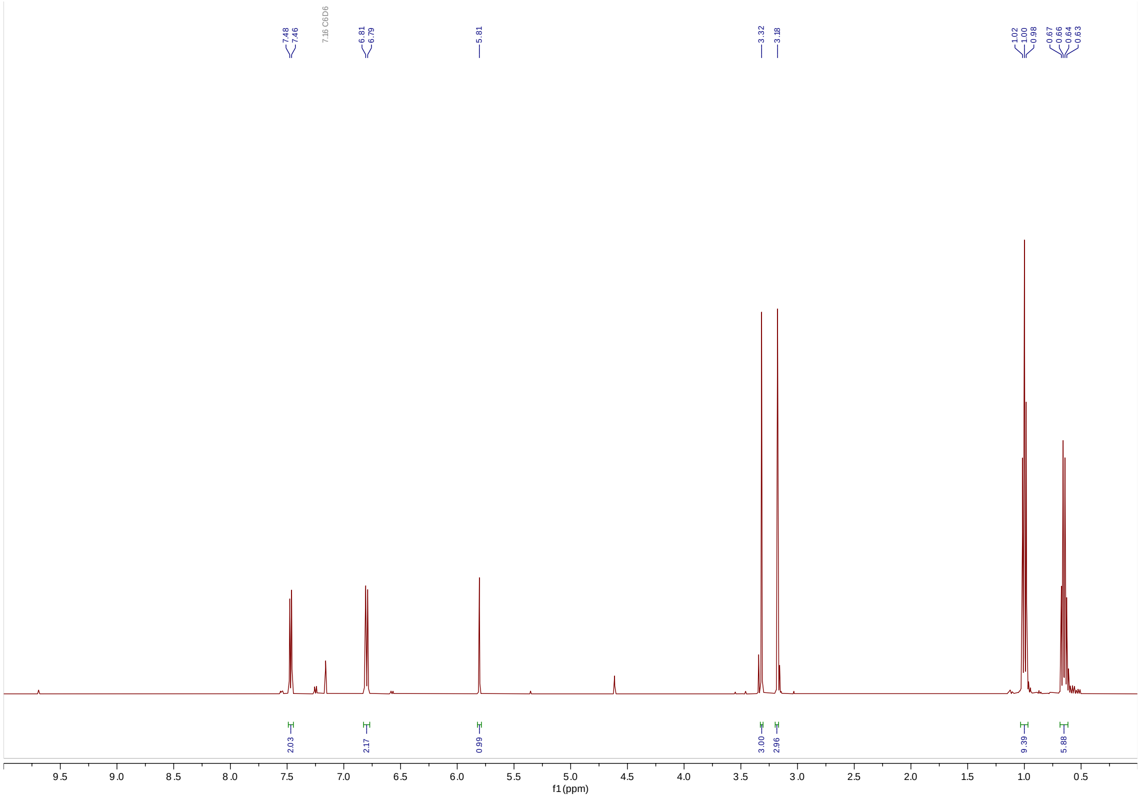

^{13}**C-NMR** (126 MHz, C_6D_6) spectra of **15**

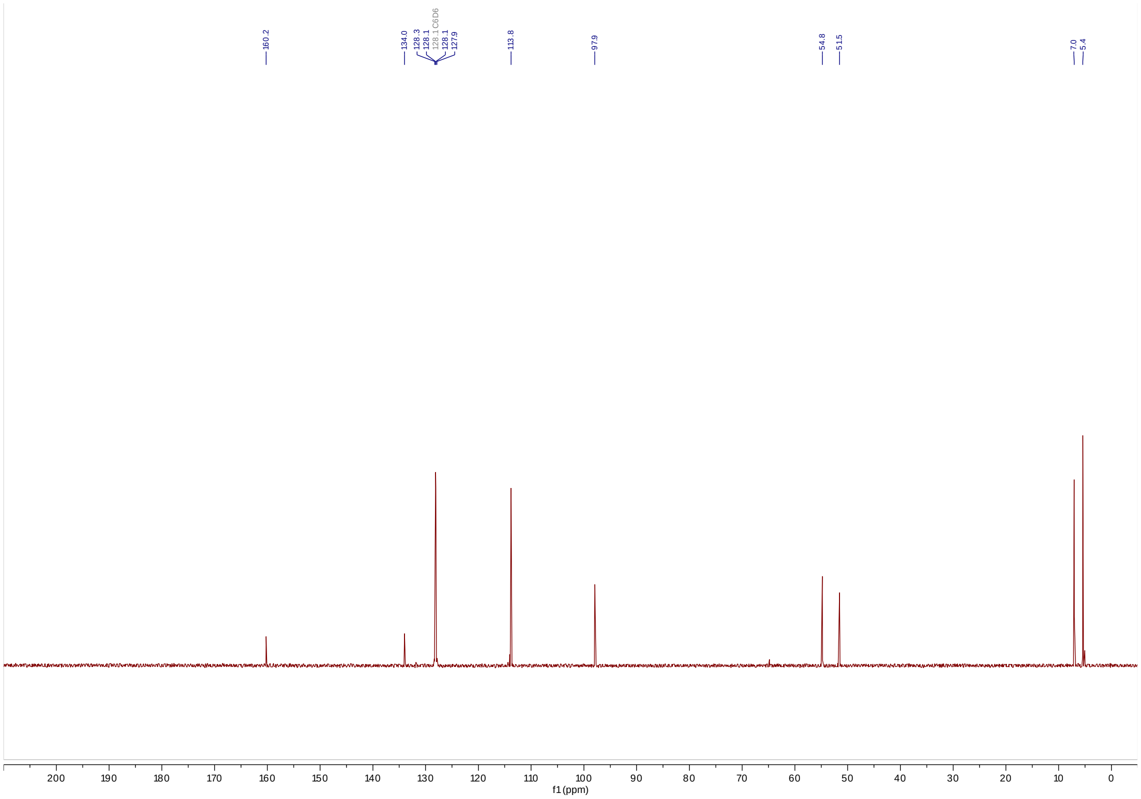

Compound 16: triethyl(methoxy(3-(trifluoromethyl)phenyl)methoxy)silane (See compound data)

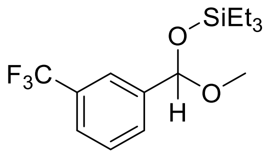

16

^{1}H-NMR (500 MHz, C_6D_6) spectra of **16**

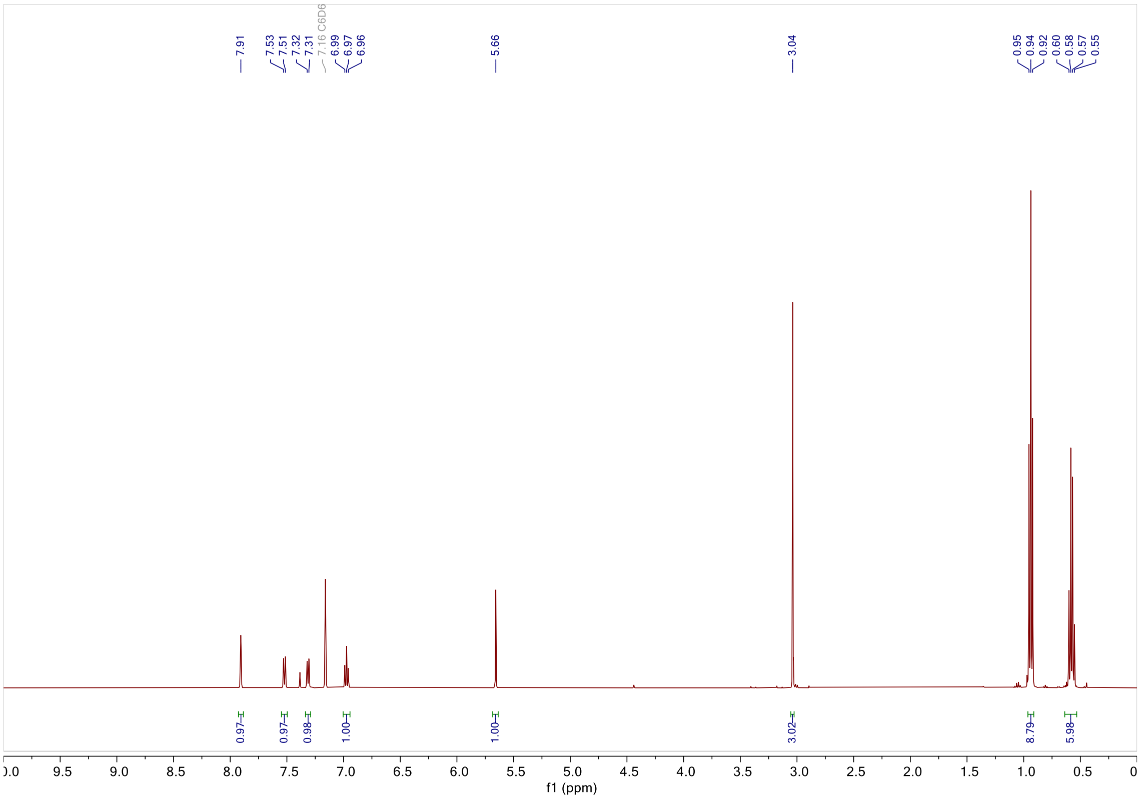

^{13}C-NMR (126 MHz, C_6D_6) spectra of **16**

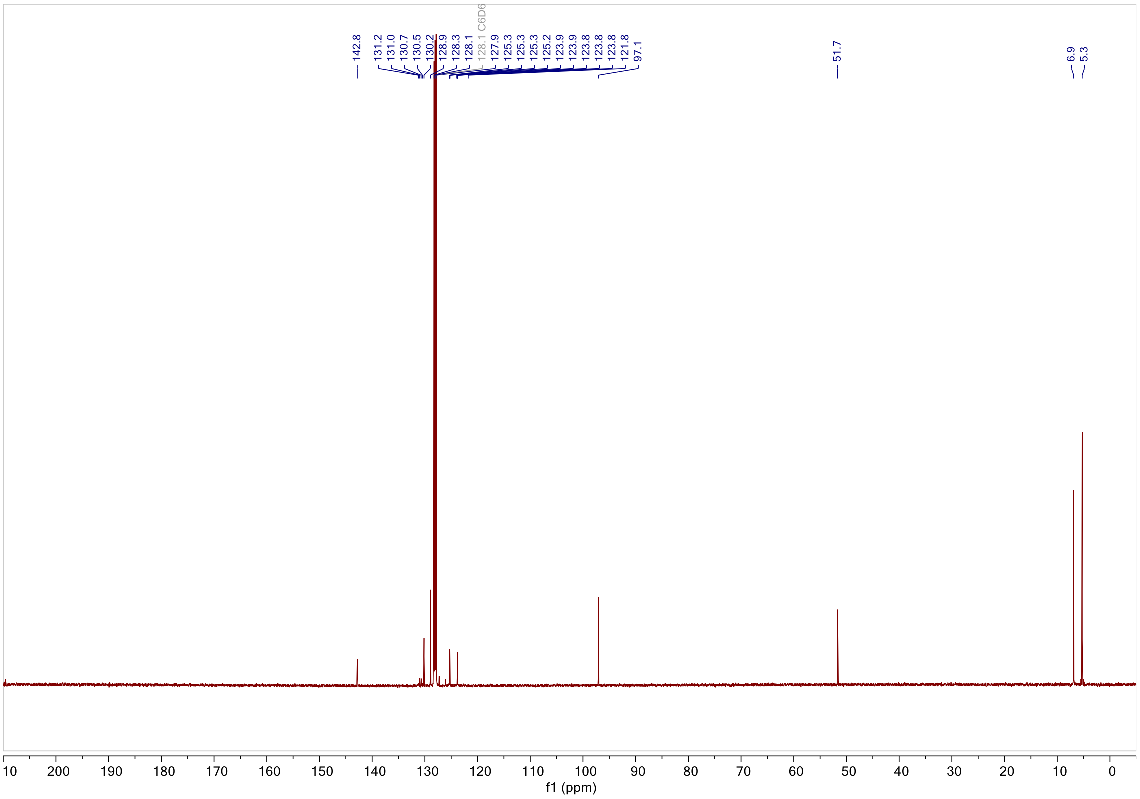

^{19}F-NMR (282 MHz, C_6D_6) spectra of **16**

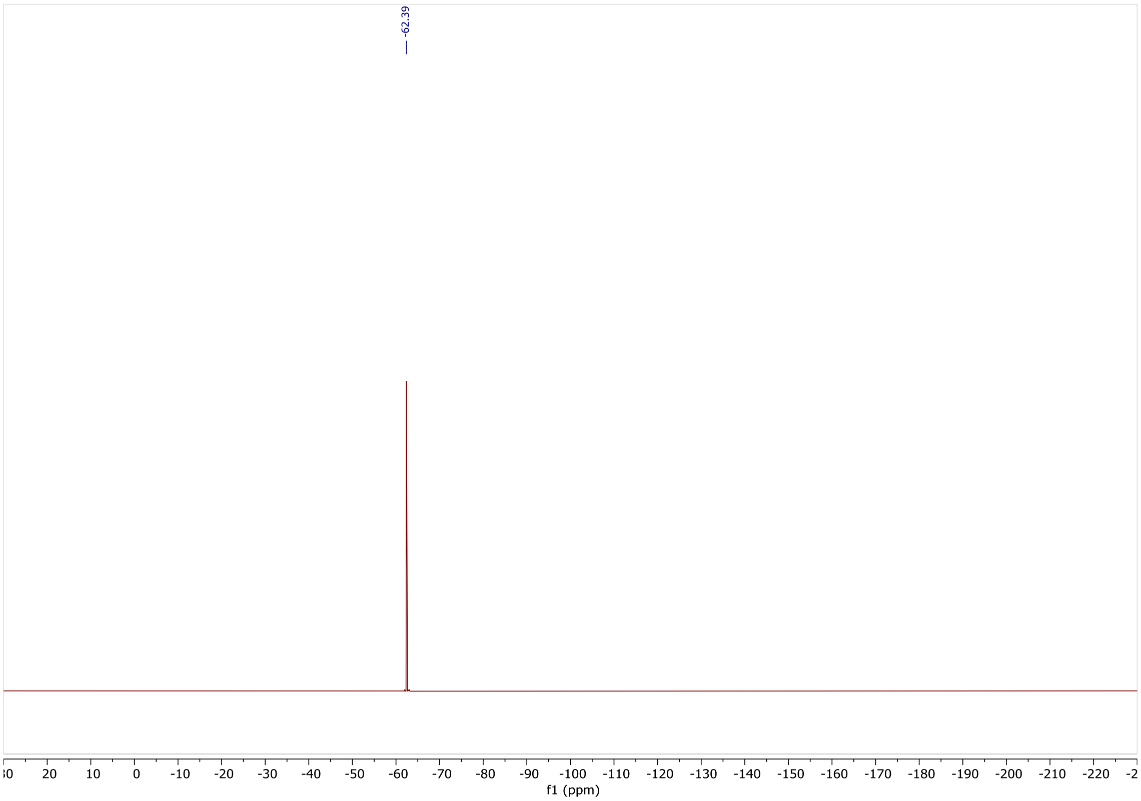

Compound 17: ((1-ethoxydecyl)oxy)triethylsilane (See compound data)

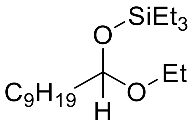

17

¹H-NMR (500 MHz, $CDCl_3$) spectra of **17**

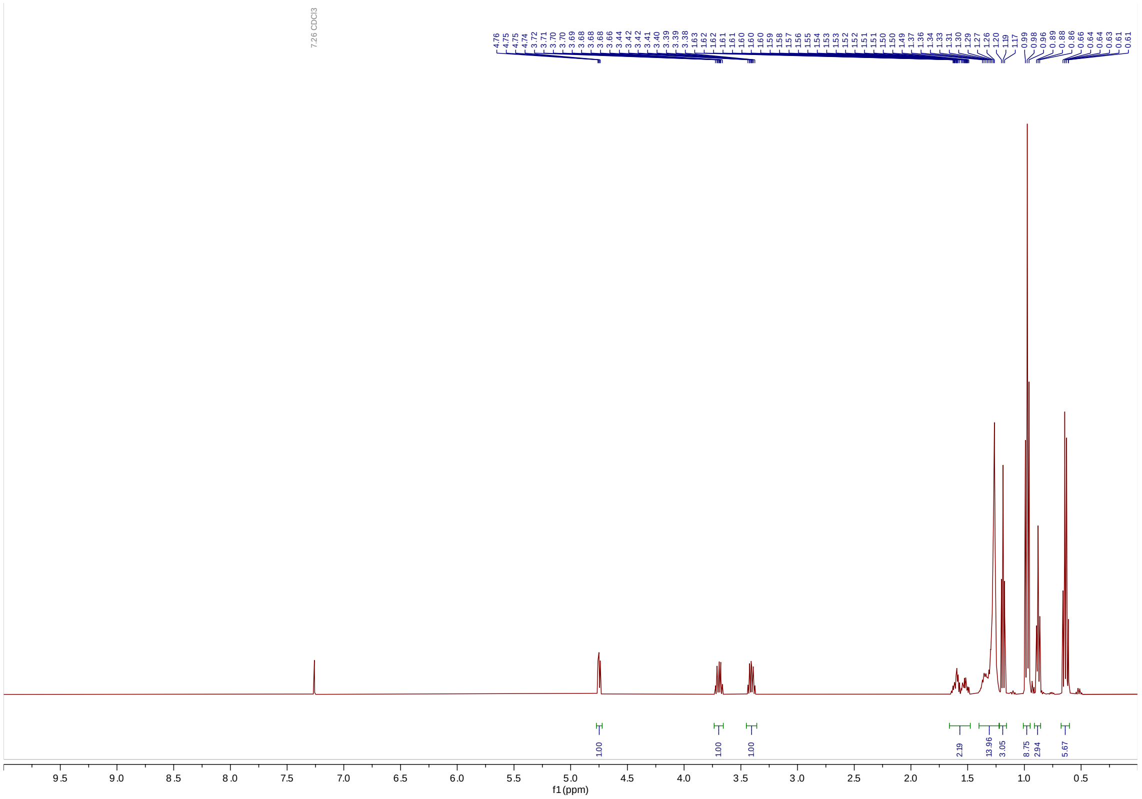

^{13}C-NMR (126 MHz, $CDCl_3$) spectra of **17**

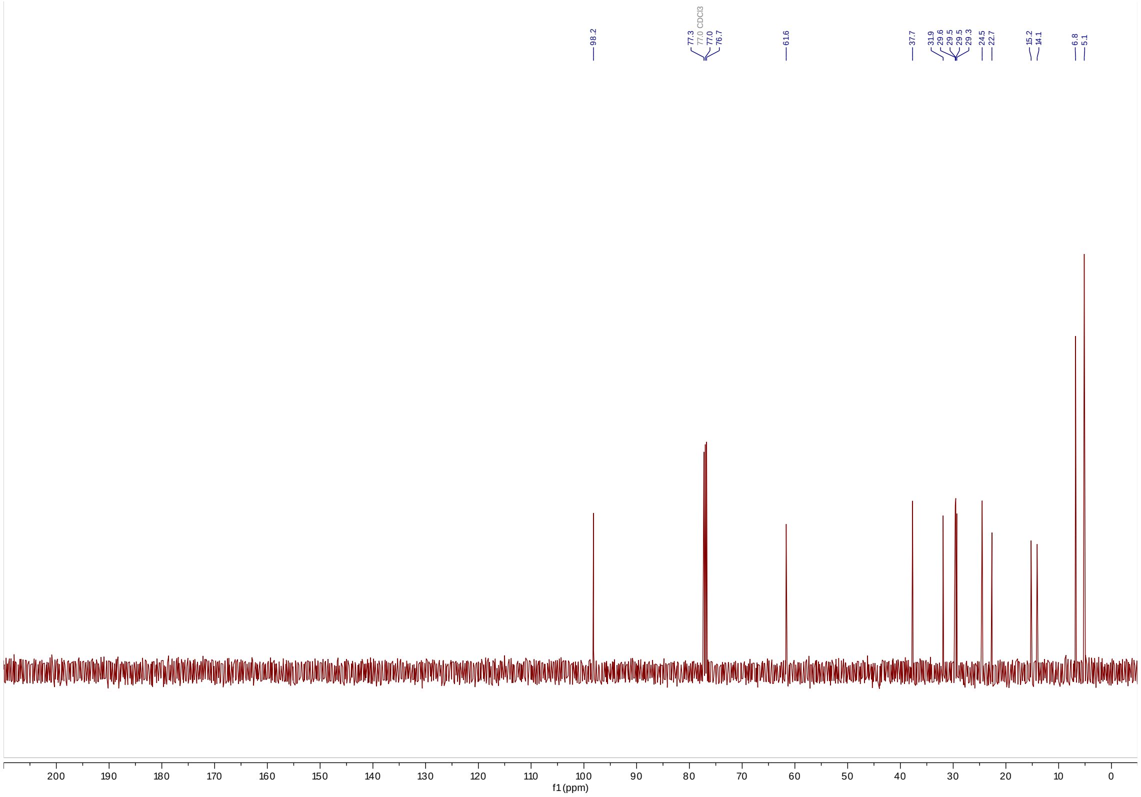

Compound 18: 3,3,13,13-tetraethyl-5,11-dinonyl-8-((1-((triethylsilyl)oxy)decyl)oxy)-4,6,10,12-tetraoxa-3,13-disilapentadecane (See compound data)

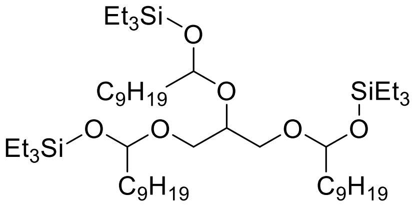

18

^{1}H-NMR (500 MHz, $CDCl_3$) spectra of **18**

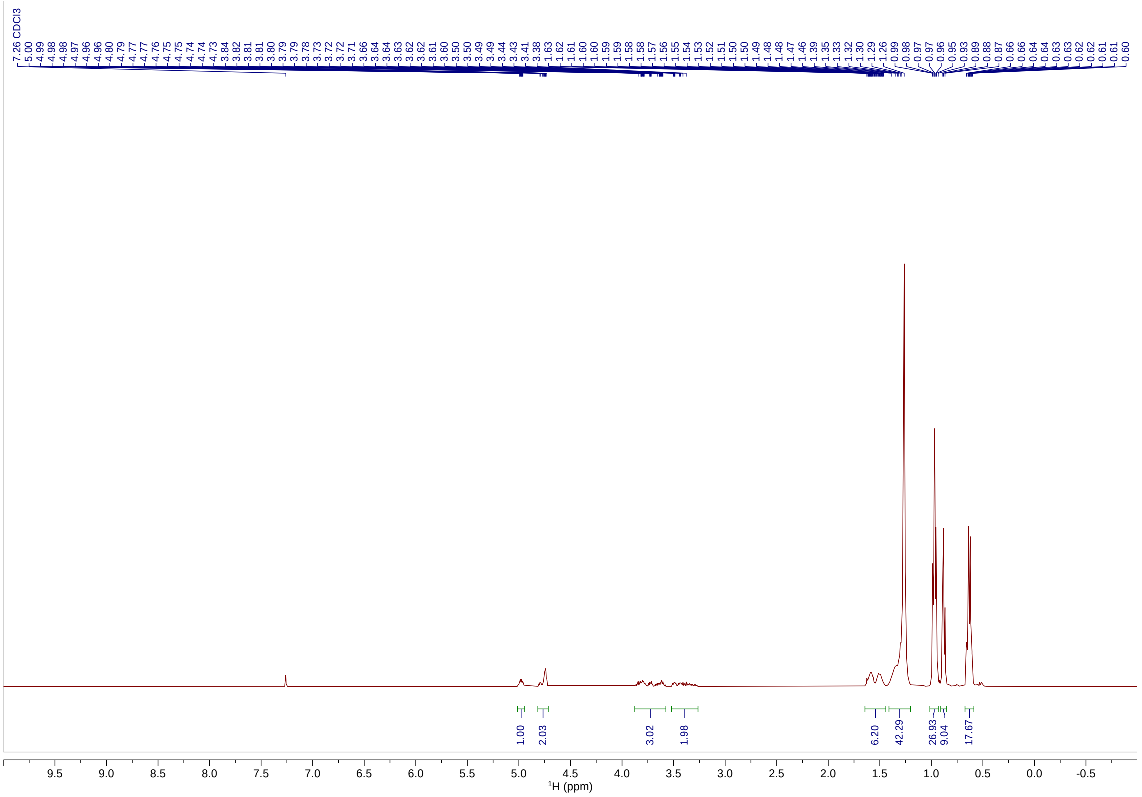

^{13}**C-NMR** (126 MHz, $CDCl_3$) spectra of **18**

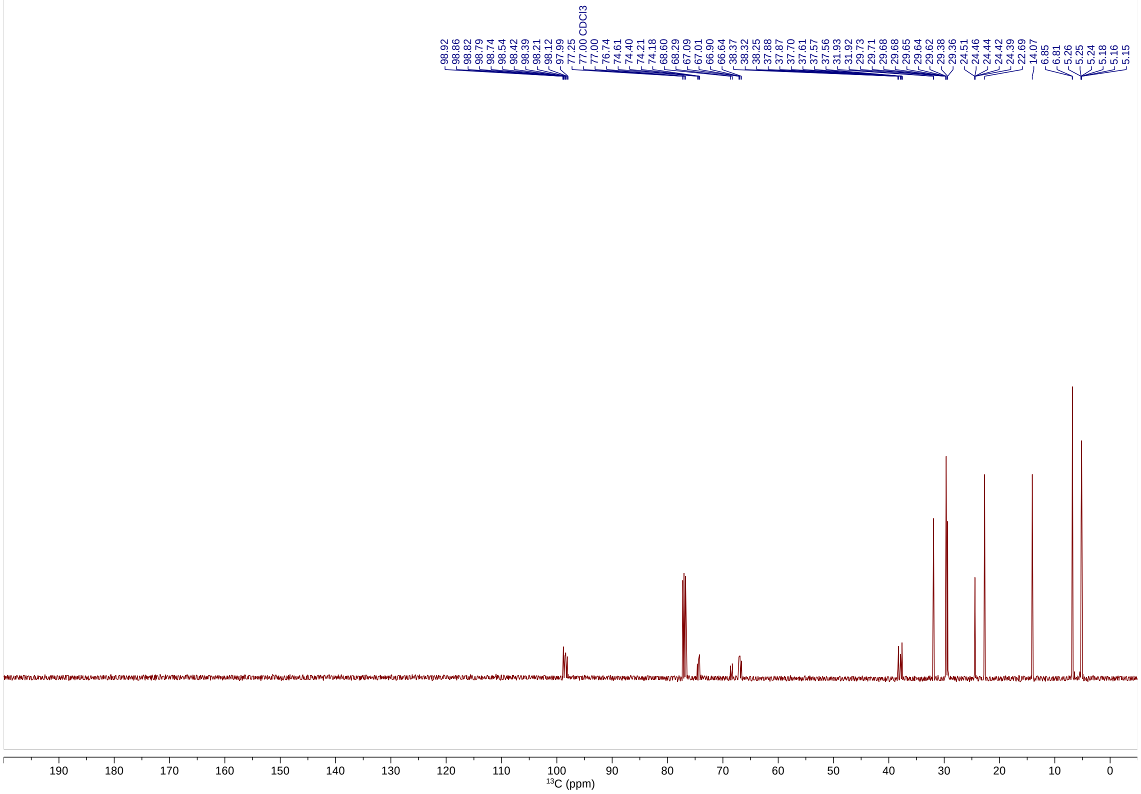

Compound 19: (3-bromo-1-ethoxypropoxy)triethylsilane (See compound data)

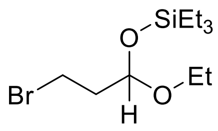

19

^{1}H-NMR (500 MHz, $CDCl_3$) spectra of **19**

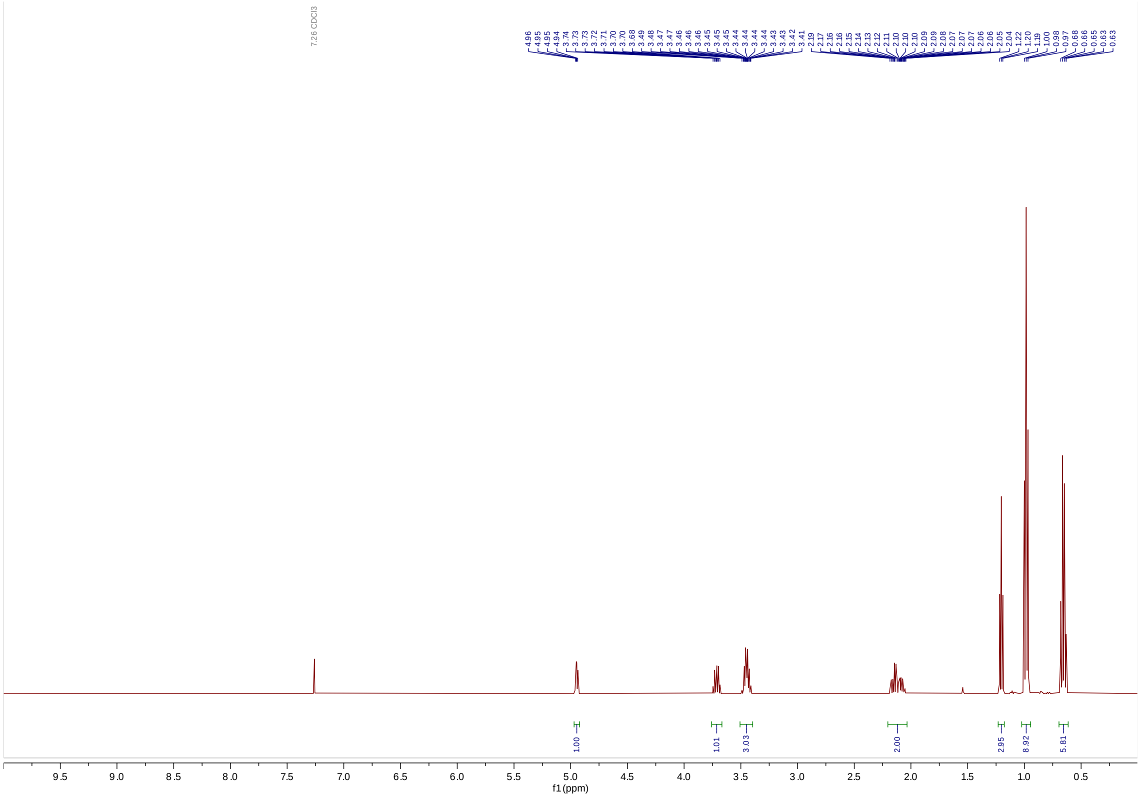

^{13}**C-NMR** (126 MHz, $CDCl_3$) spectra of **19**

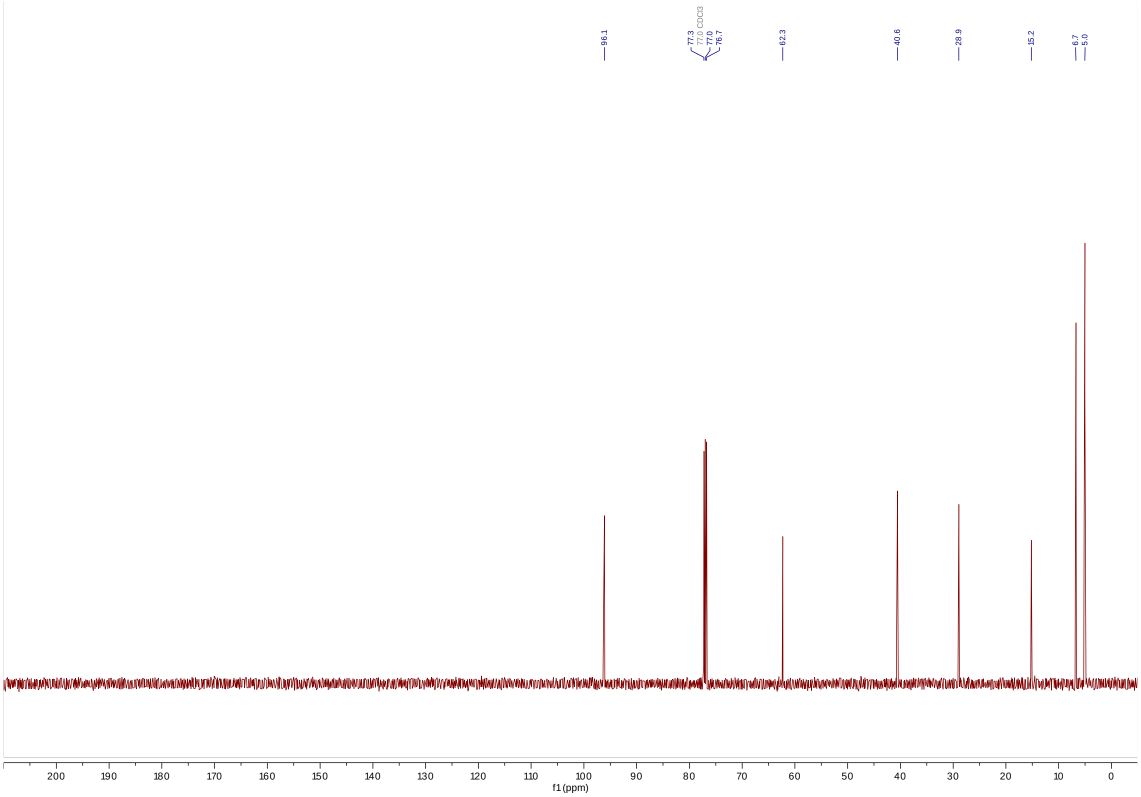

Compound 20: (4-bromo-1-ethoxybutoxy)triethylsilane (See compound data)

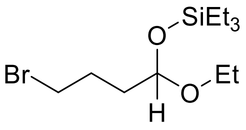

20

[1]**H-NMR** (500 MHz, $CDCl_3$) spectra of **20**

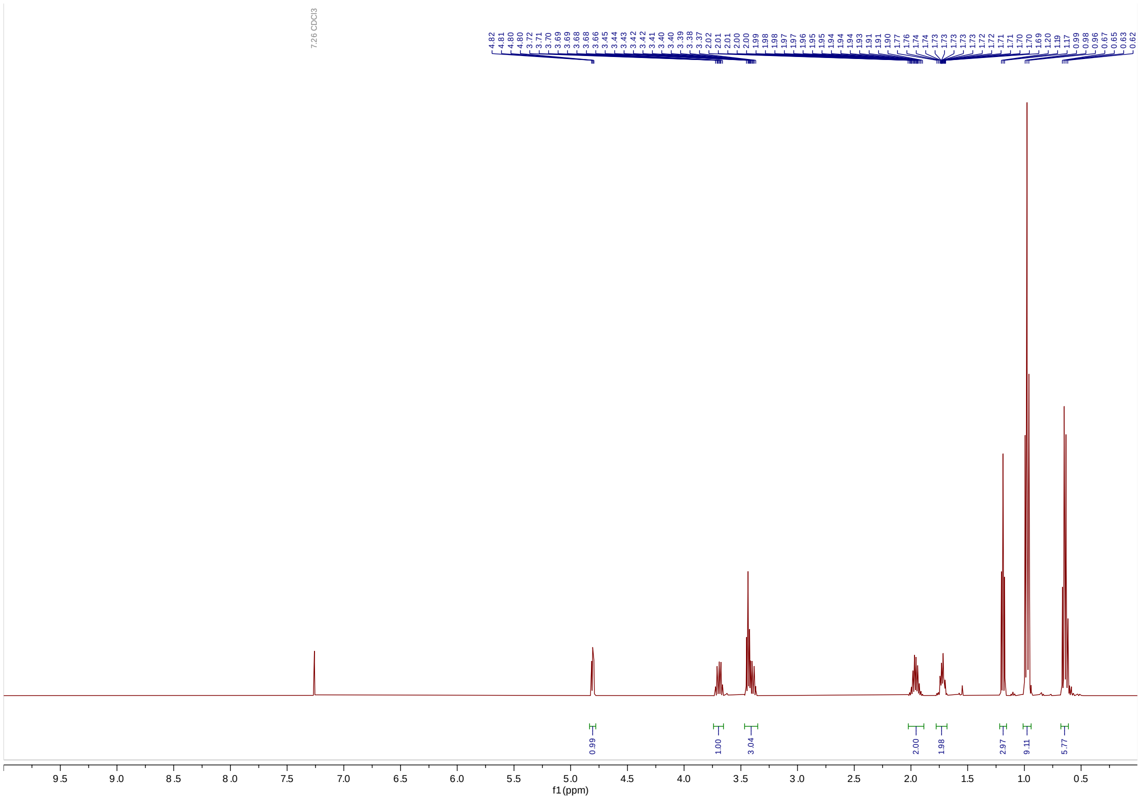

^{13}**C-NMR** (126 MHz, $CDCl_3$) spectra of **20**

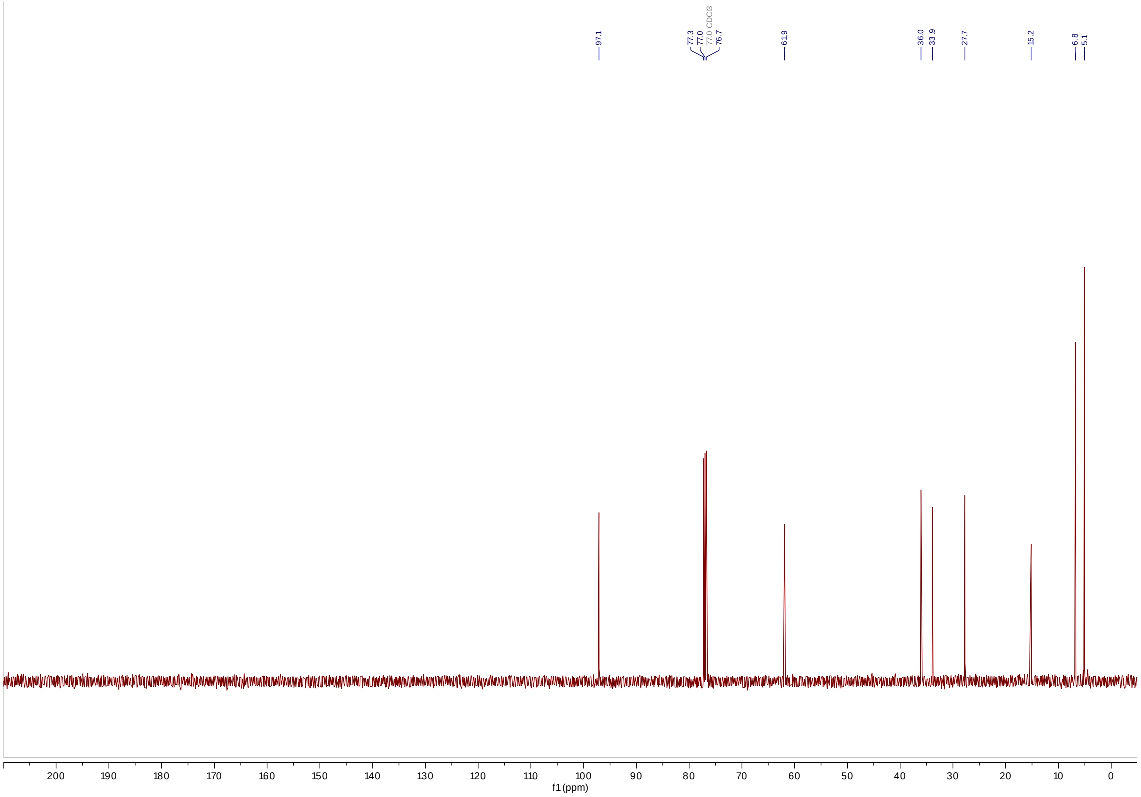

Compound 21: ((5-bromo-1-ethoxypentyl)oxy)triethylsilane (See compound data)

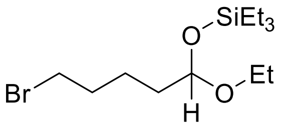

21

^{1}H-NMR (500 MHz, $CDCl_3$) spectra of **21**

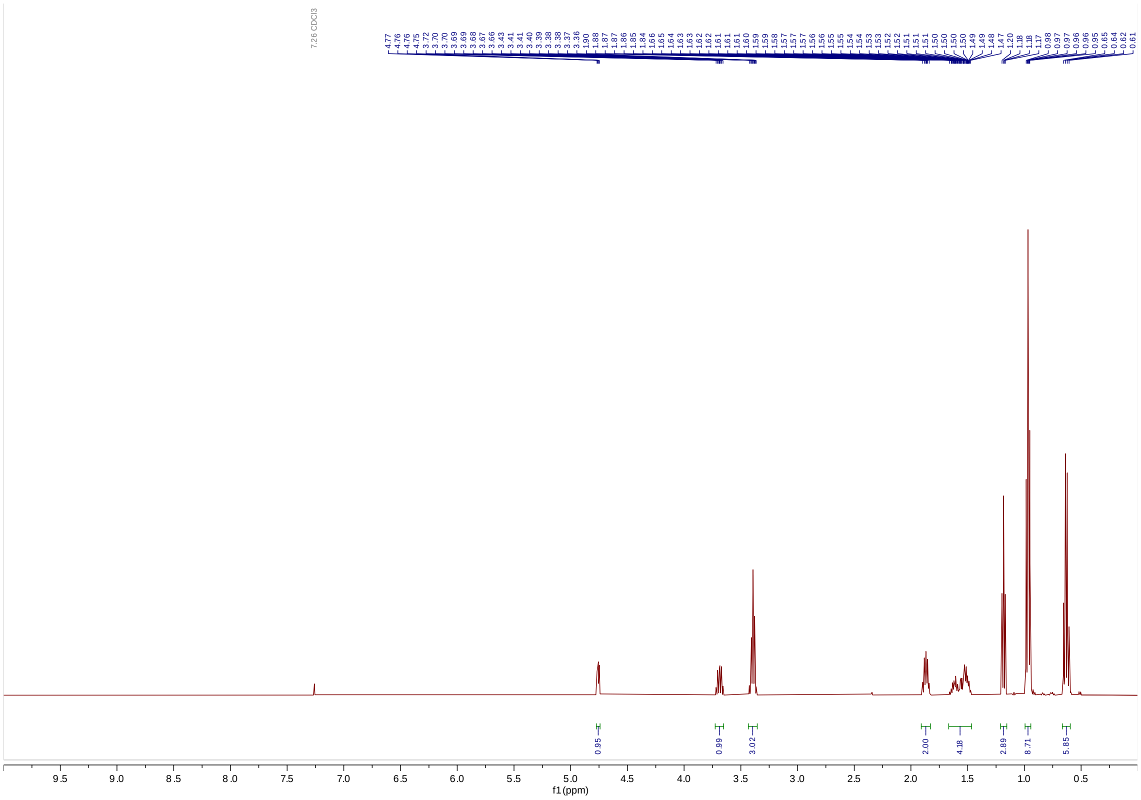

^{13}**C-NMR** (126 MHz, $CDCl_3$) spectra of **21**

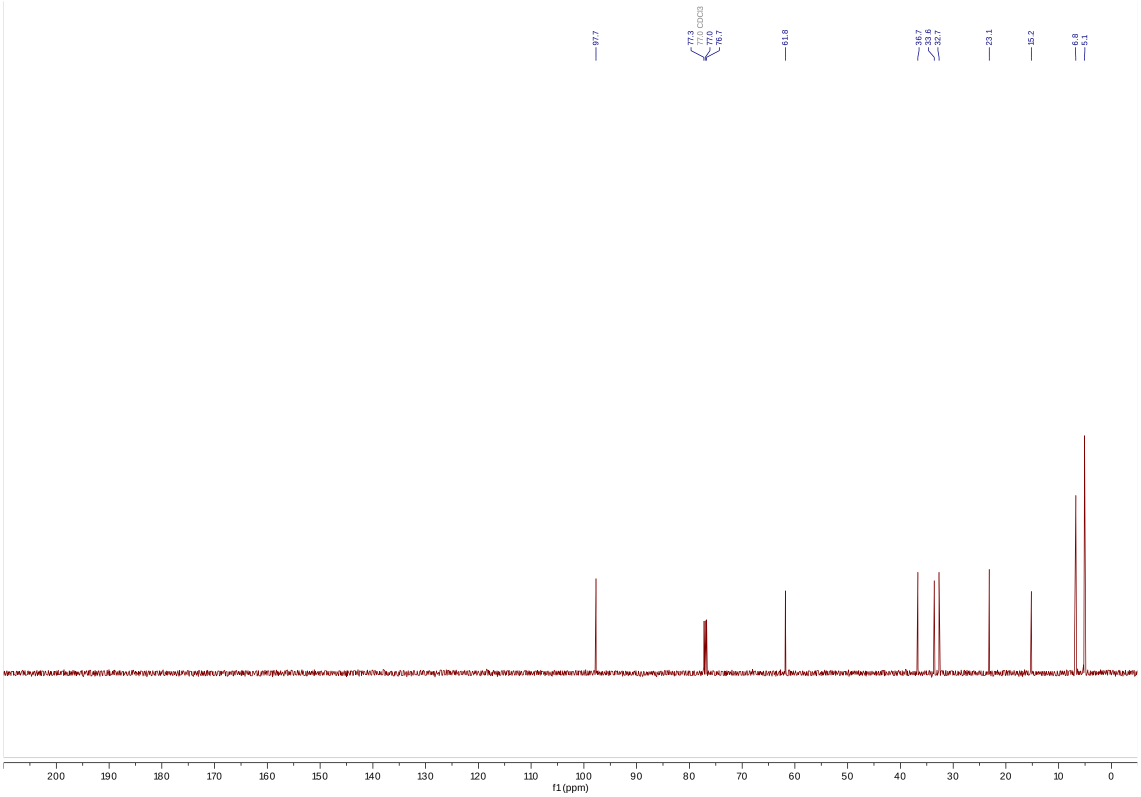

Compound 22: ((6-bromo-1-ethoxyhexyl)oxy)triethylsilane (See compound data)

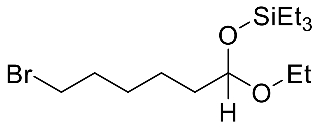

22

^{1}H-NMR (500 MHz, $CDCl_3$) spectra of **22**

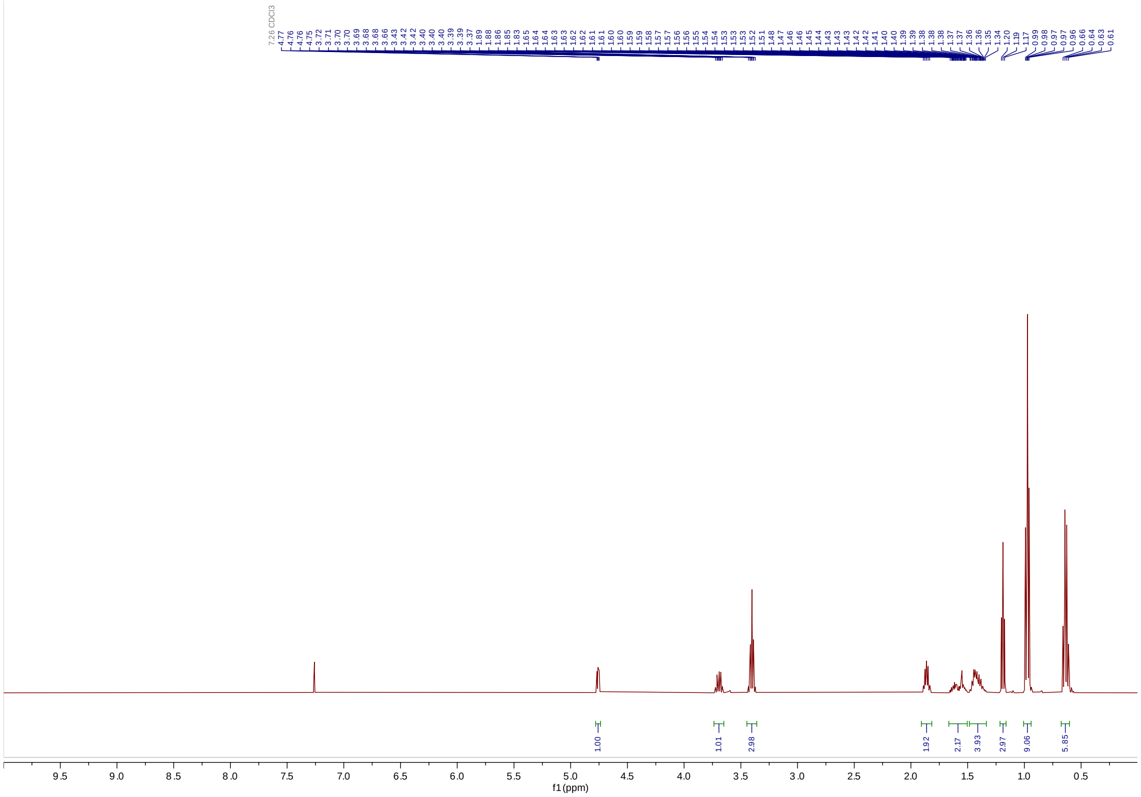

^{13}**C-NMR** (126 MHz, $CDCl_3$) spectra of **22**

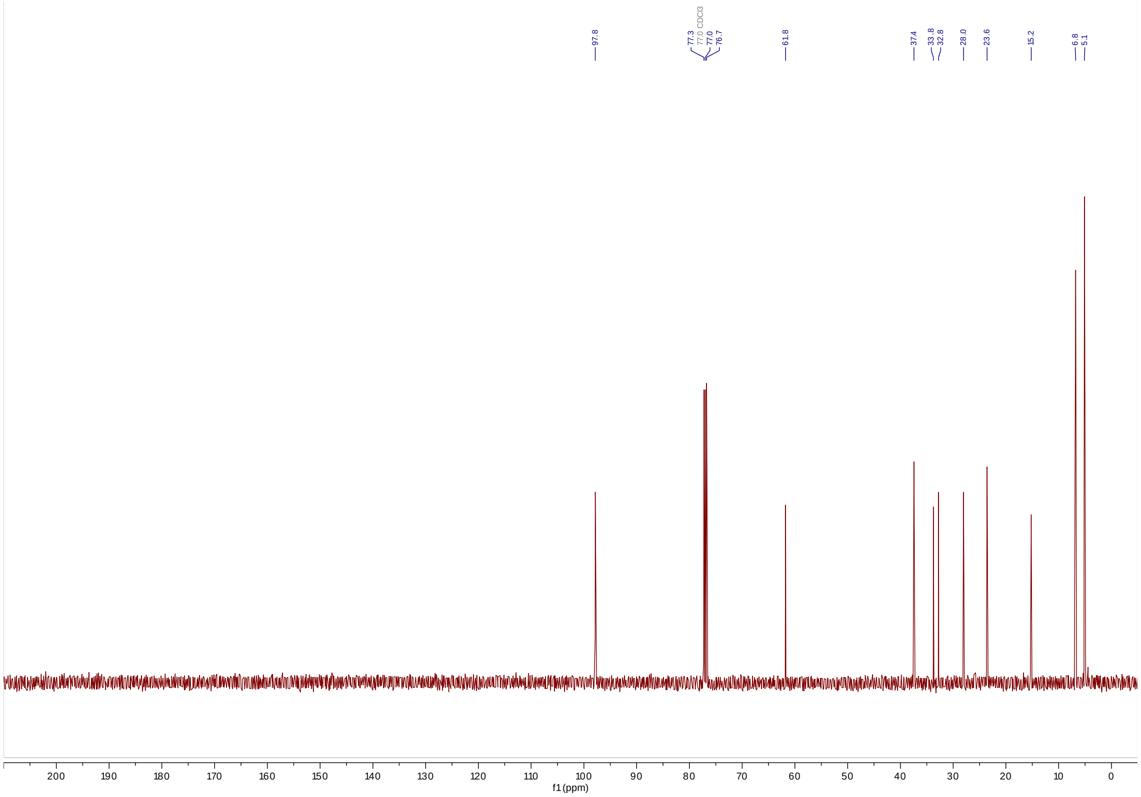

Compound 23: (4-bromo-1-isopropoxybutoxy)triethylsilane (See compound data)

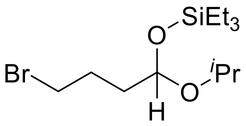

23

^{1}H-NMR (500 MHz, $CDCl_3$) spectra of **23**

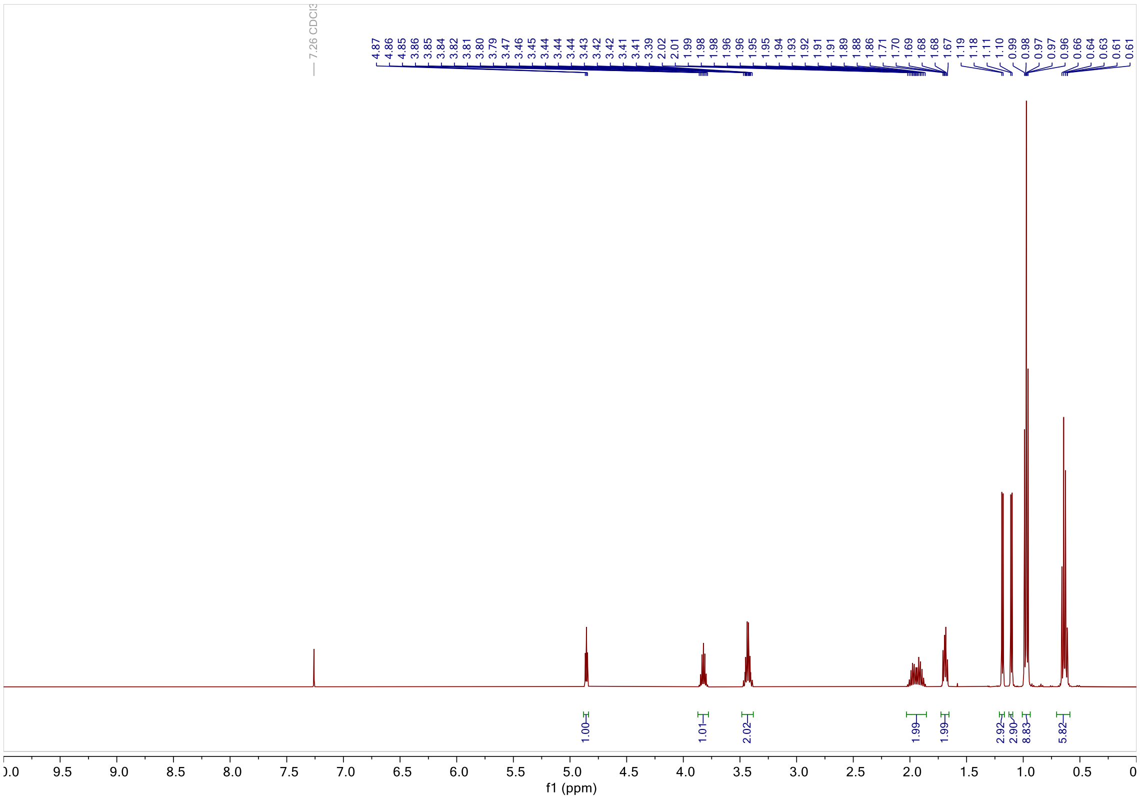

^{13}**C-NMR** (126 MHz, $CDCl_3$) spectra of **23**

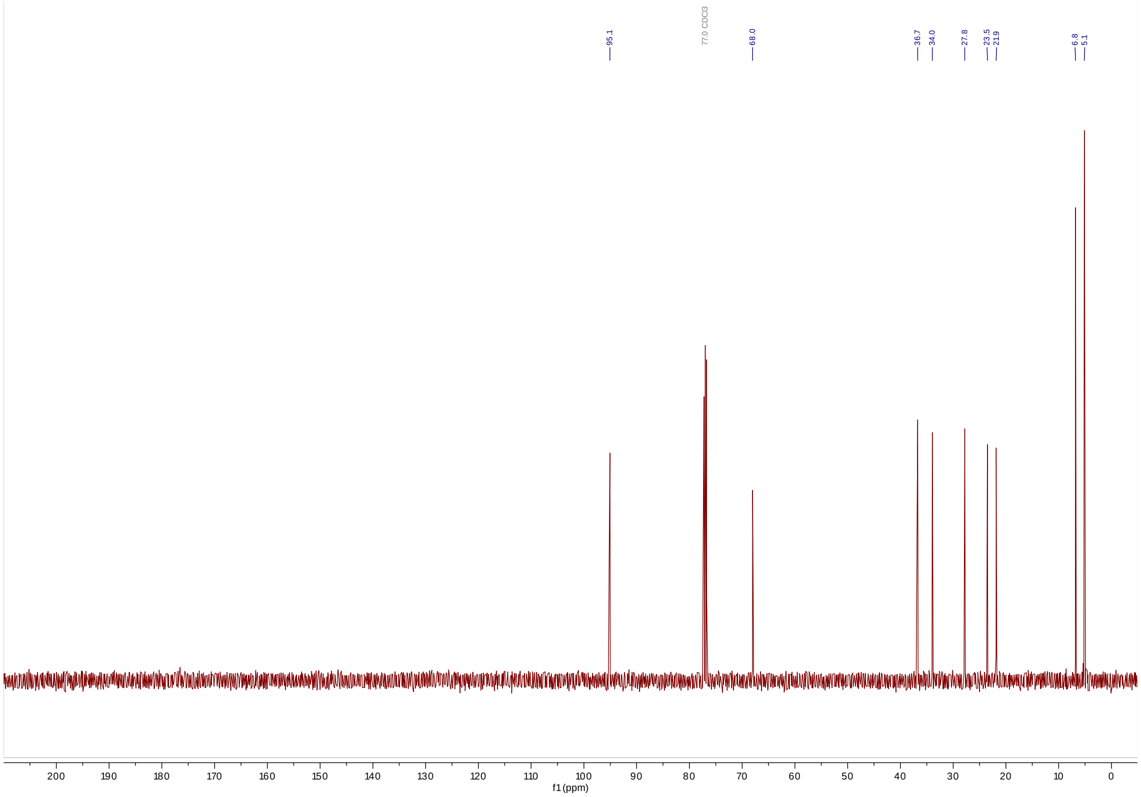

Compound 24: (2-bromo-1-ethoxyethoxy)triethylsilane (See compound data)

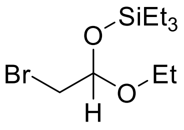

24

^{1}H-NMR (500 MHz, $CDCl_3$) spectra of **24**

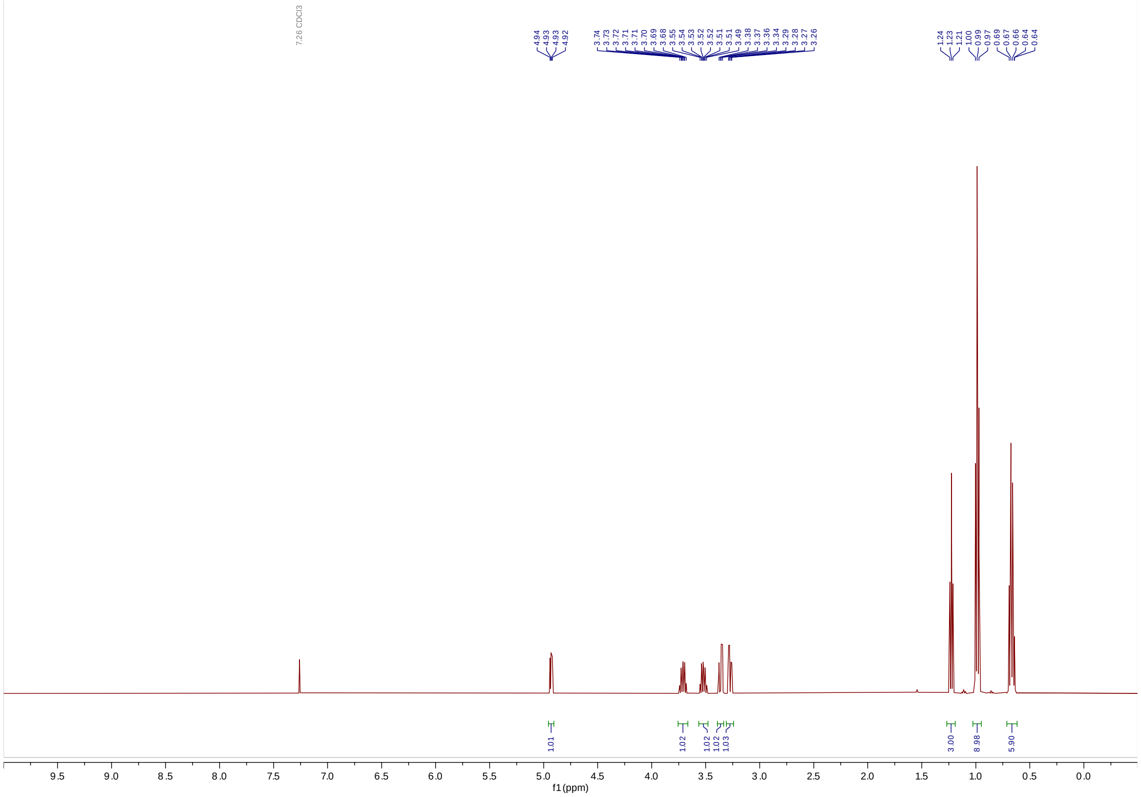

^{13}**C-NMR** (126 MHz, $CDCl_3$) spectra of **24**

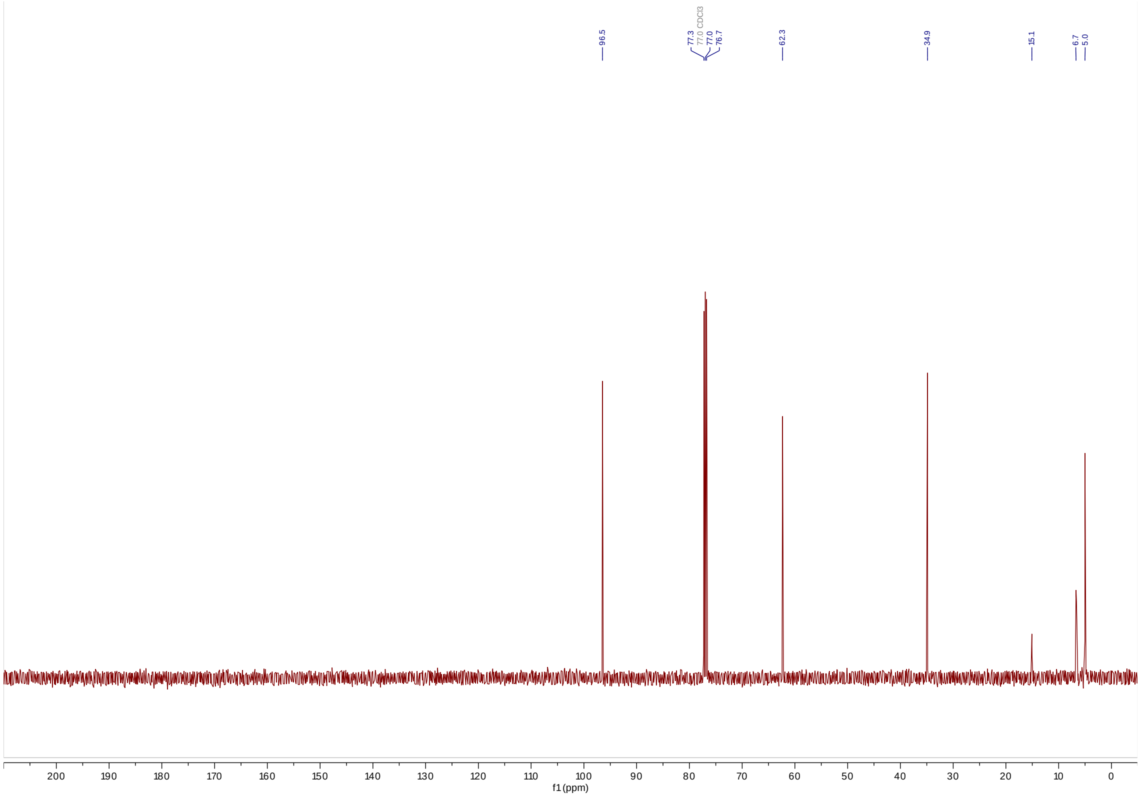

Compound 25: (2-bromo-1-ethoxyethoxy)triethylsilane (See compound data)

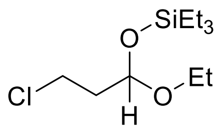

25

^{1}H-NMR (500 MHz, $CDCl_3$) spectra of **25**

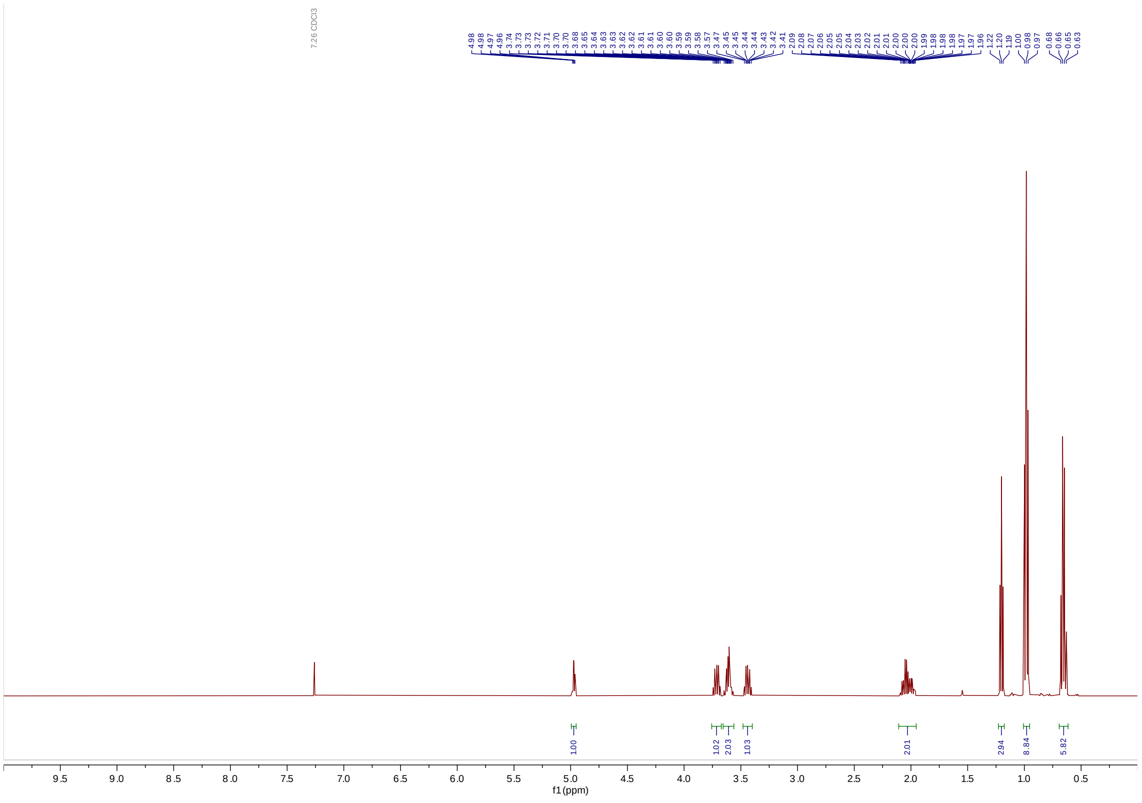

^{13}C-NMR (126 MHz, $CDCl_3$) spectra of **25**

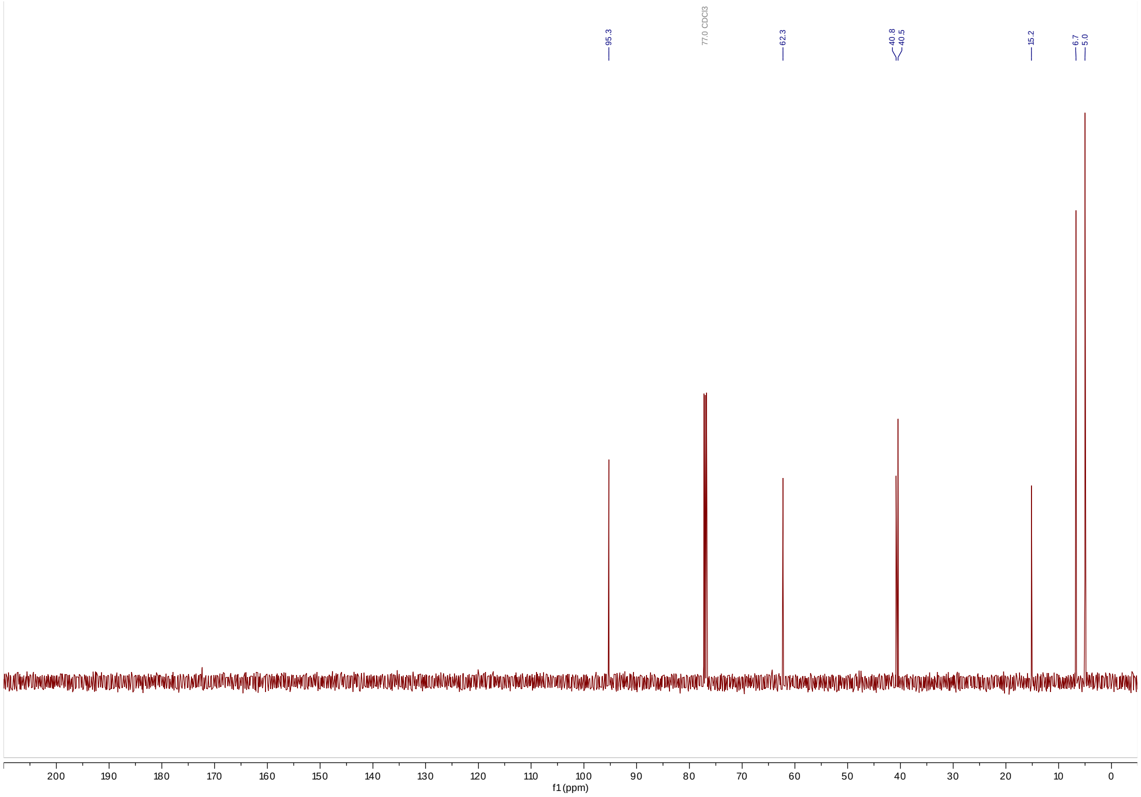

Compound 26: (2-bromo-1-methoxypropoxy)triethylsilane (See compound data)

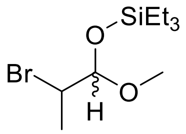

26

^{1}H-NMR (500 MHz, $CDCl_3$) spectra of **26**

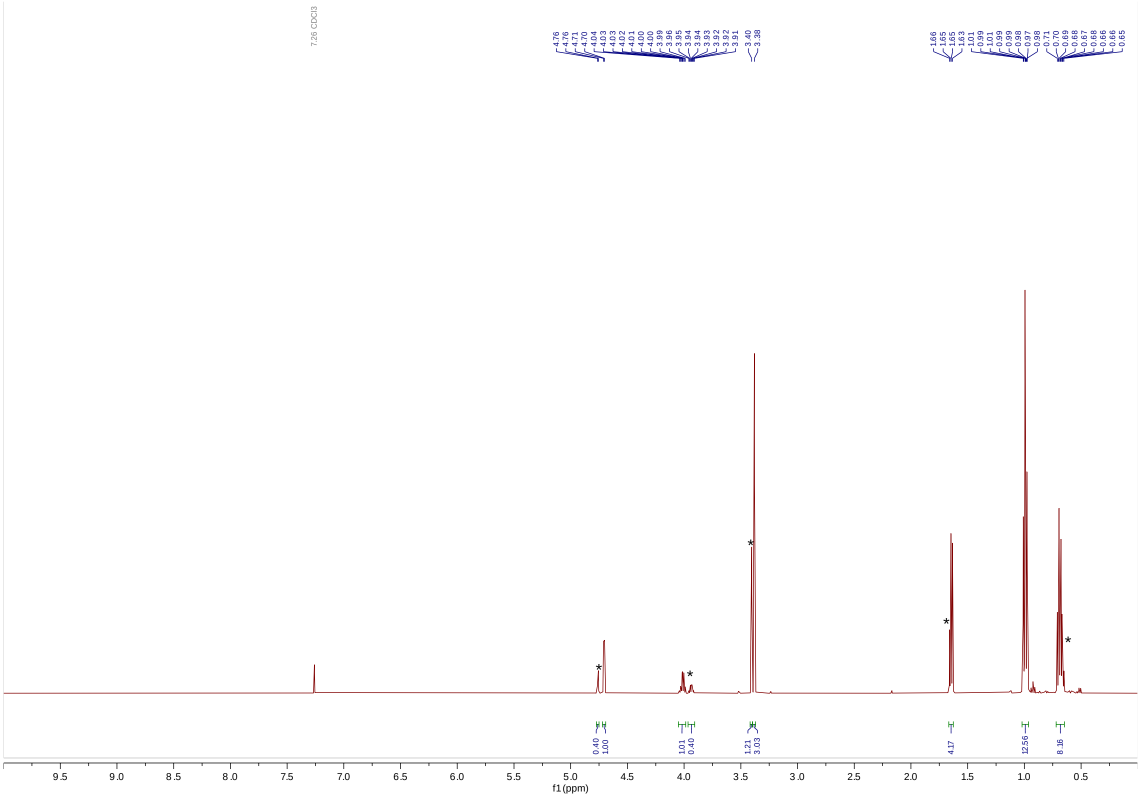

^{13}**C-NMR** (126 MHz, $CDCl_3$) spectra of **26**

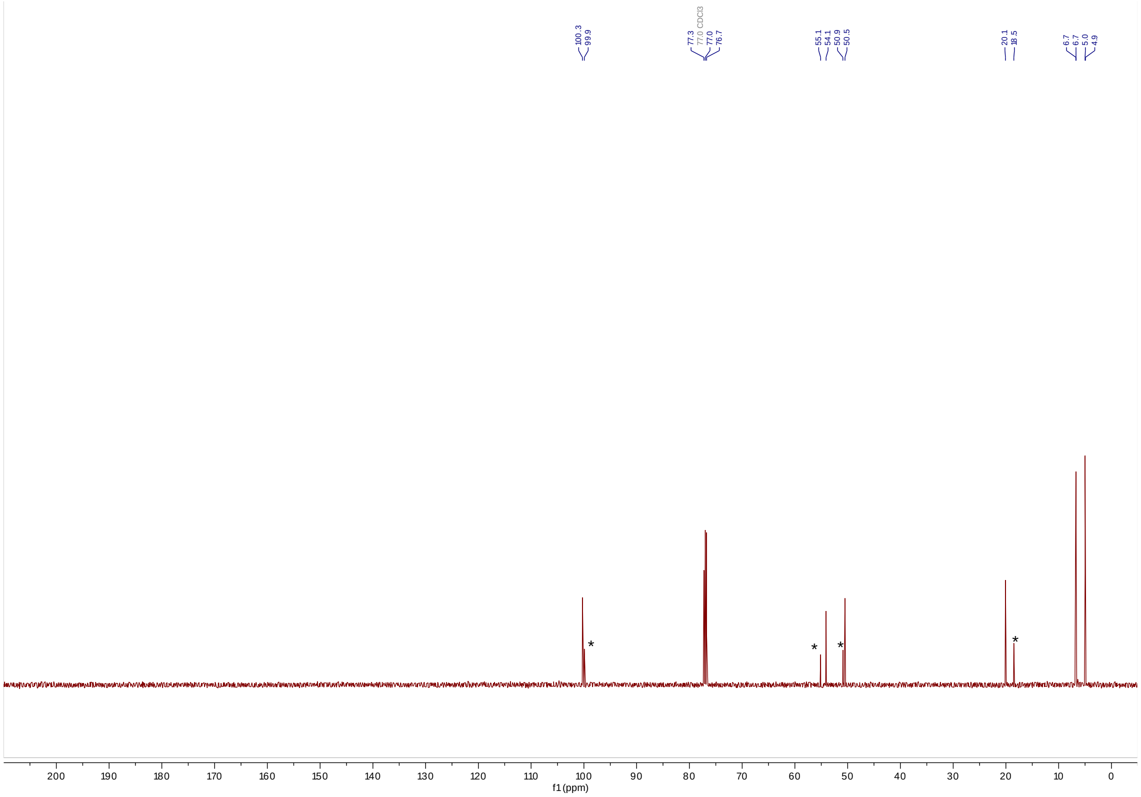

Compound 27: ((2-bromo-1-ethoxyoctyl)oxy)triethylsilane (See compound data)

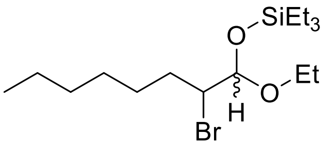

27

[1]**H-NMR** (500 MHz, $CDCl_3$) spectra of **27**

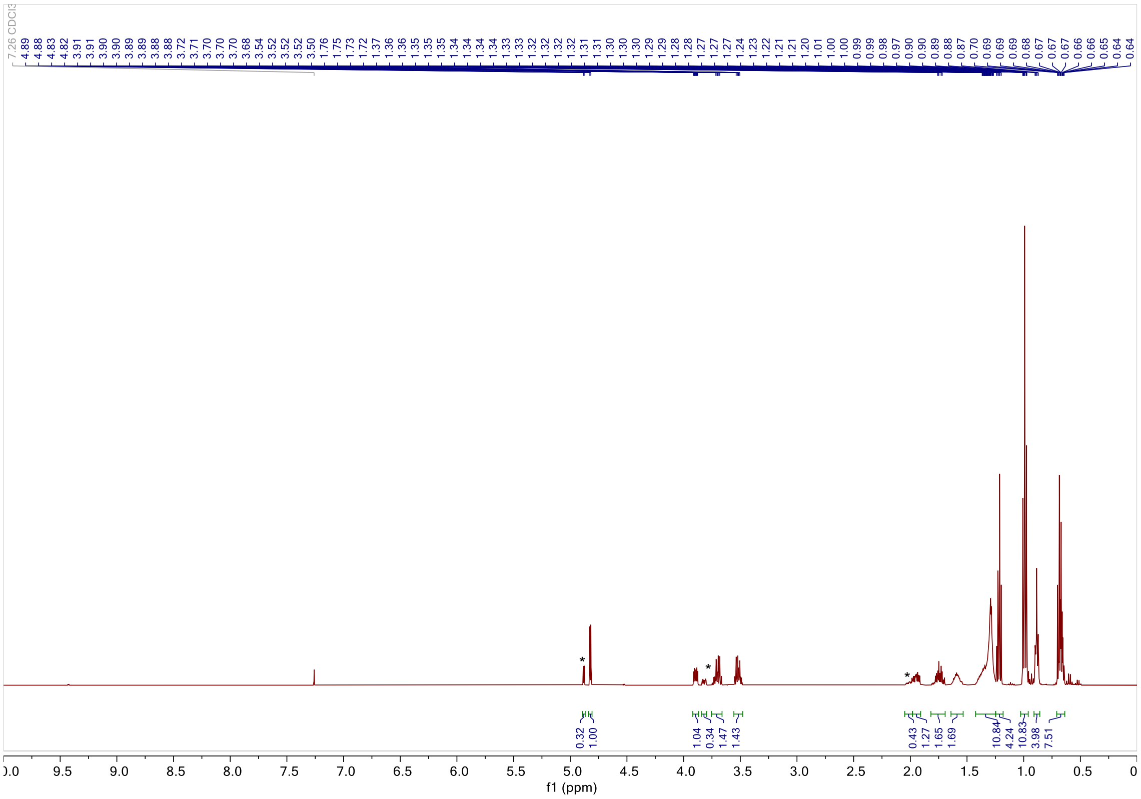

^{13}**C-NMR** (126 MHz, $CDCl_3$) spectra of **27**

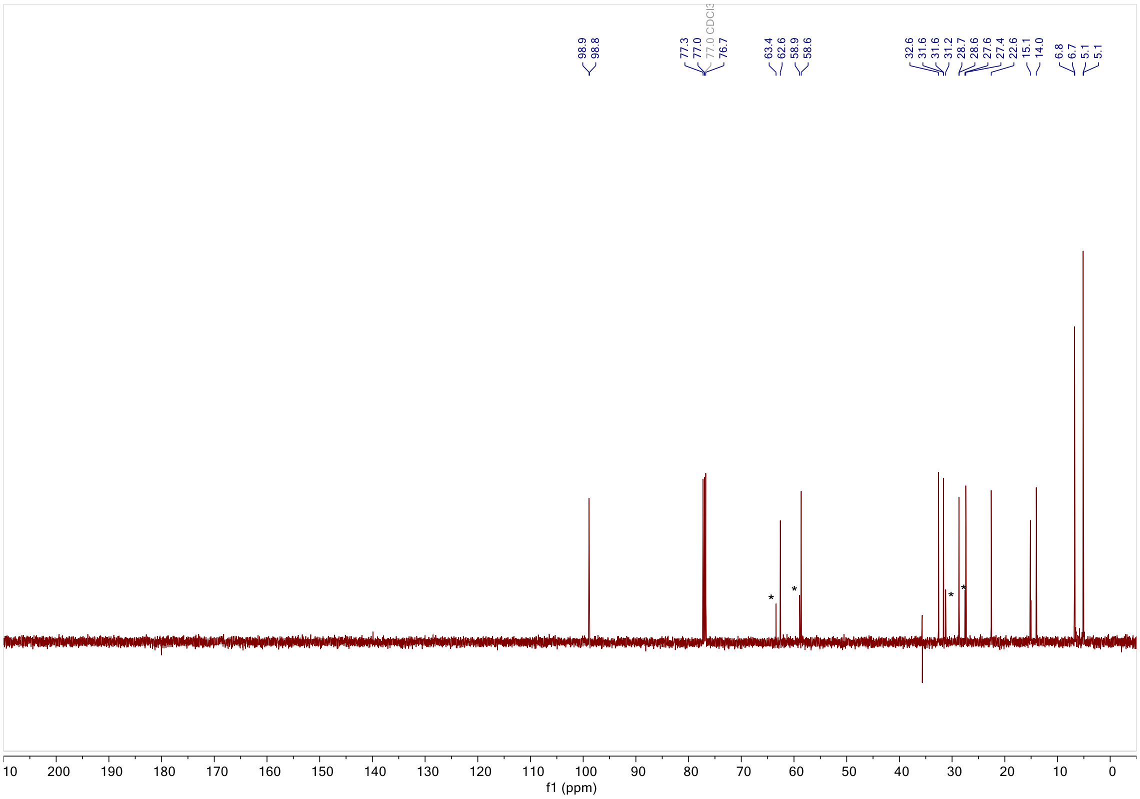

Compound 28: (4-bromo-1-ethoxy-2-methylbutoxy)triethylsilane (See compound data)

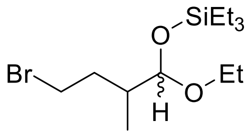

28

^{1}H-NMR (500 MHz, $CDCl_3$) spectra of **28**

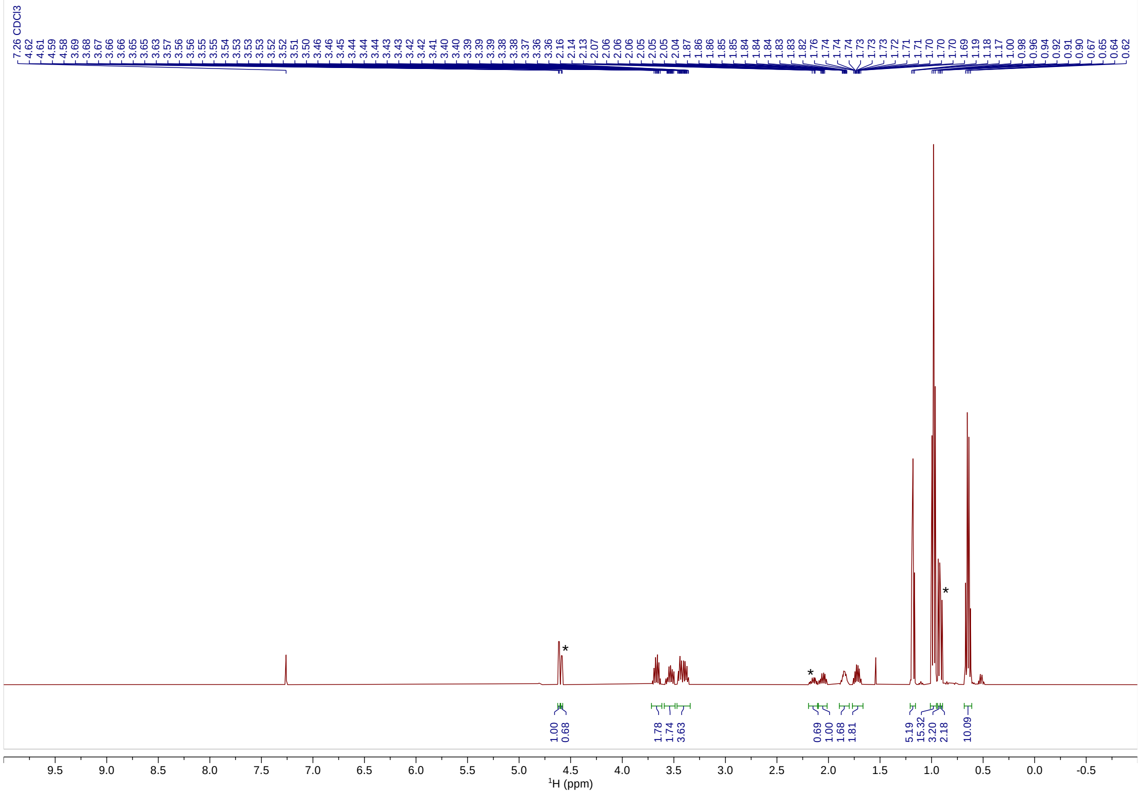

^{13}C-NMR (126 MHz, $CDCl_3$) spectra of **28**

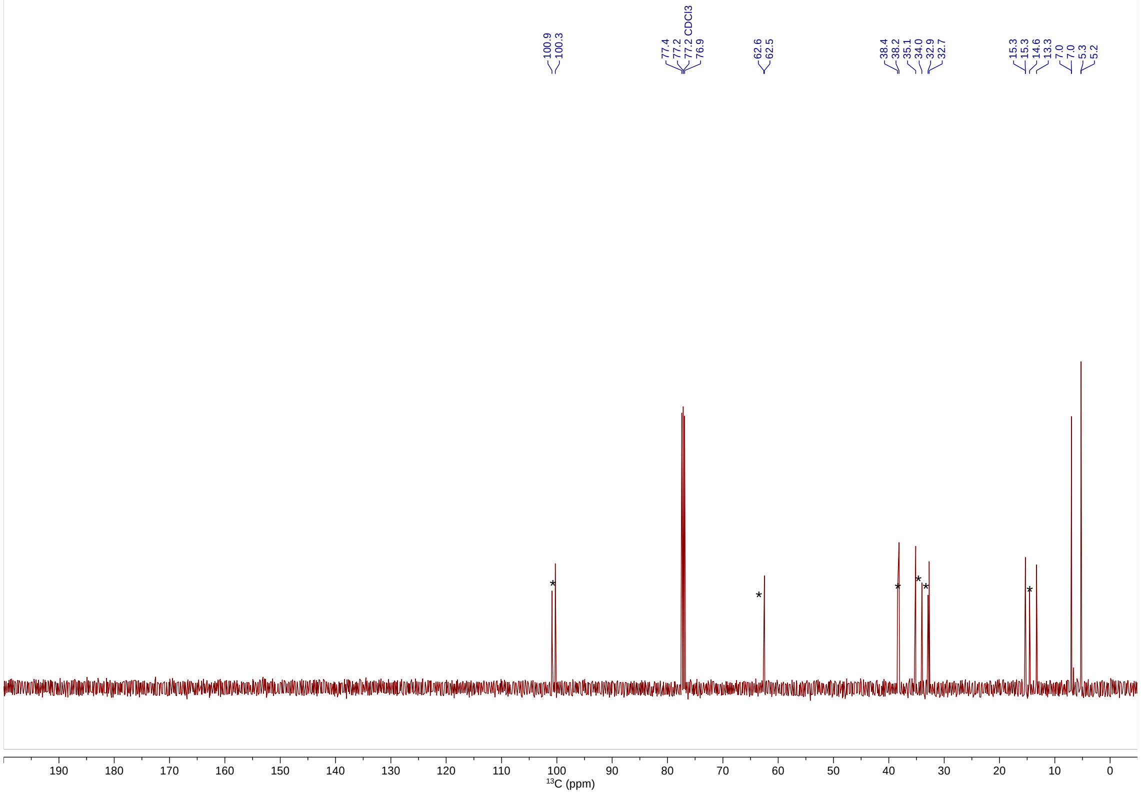

Compound 29: (4-bromo-1-ethoxy-2-fluorobutoxy)triethylsilane (See compound data)

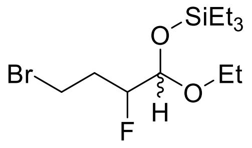

29

^{1}H-NMR (500 MHz, $CDCl_3$) spectra of **29**

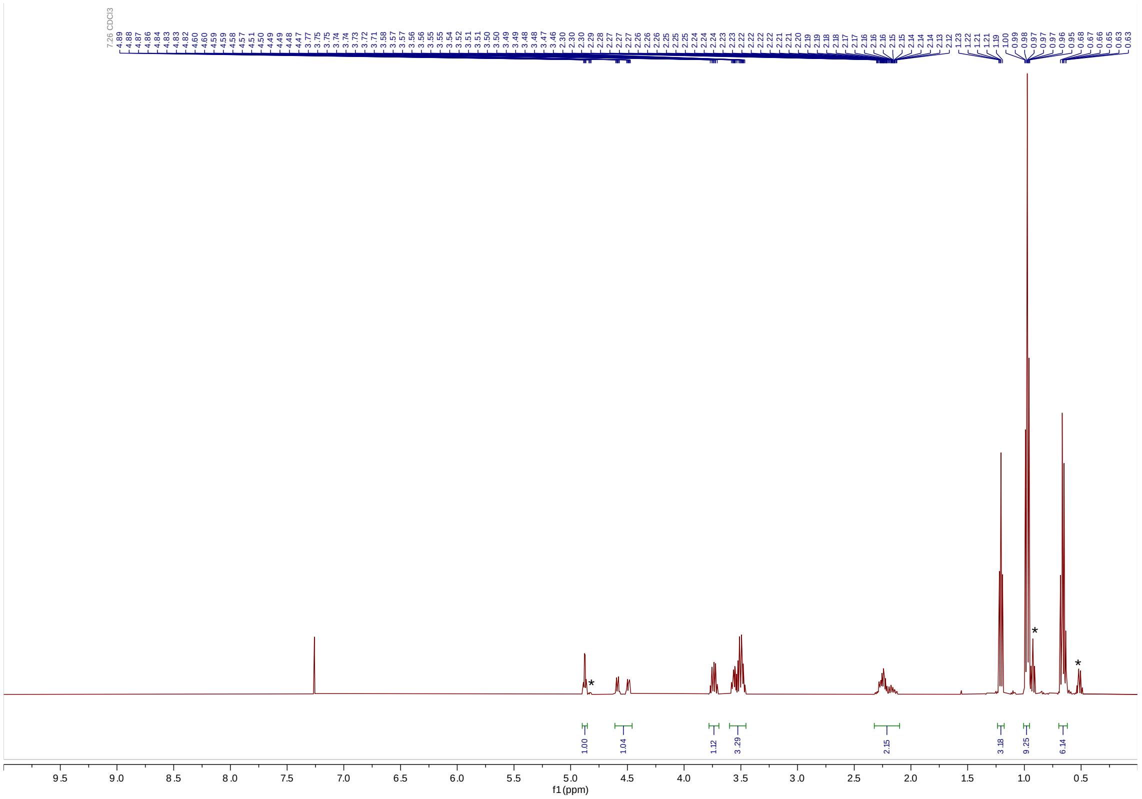

^{13}C-NMR (126 MHz, $CDCl_3$) spectra of **29**

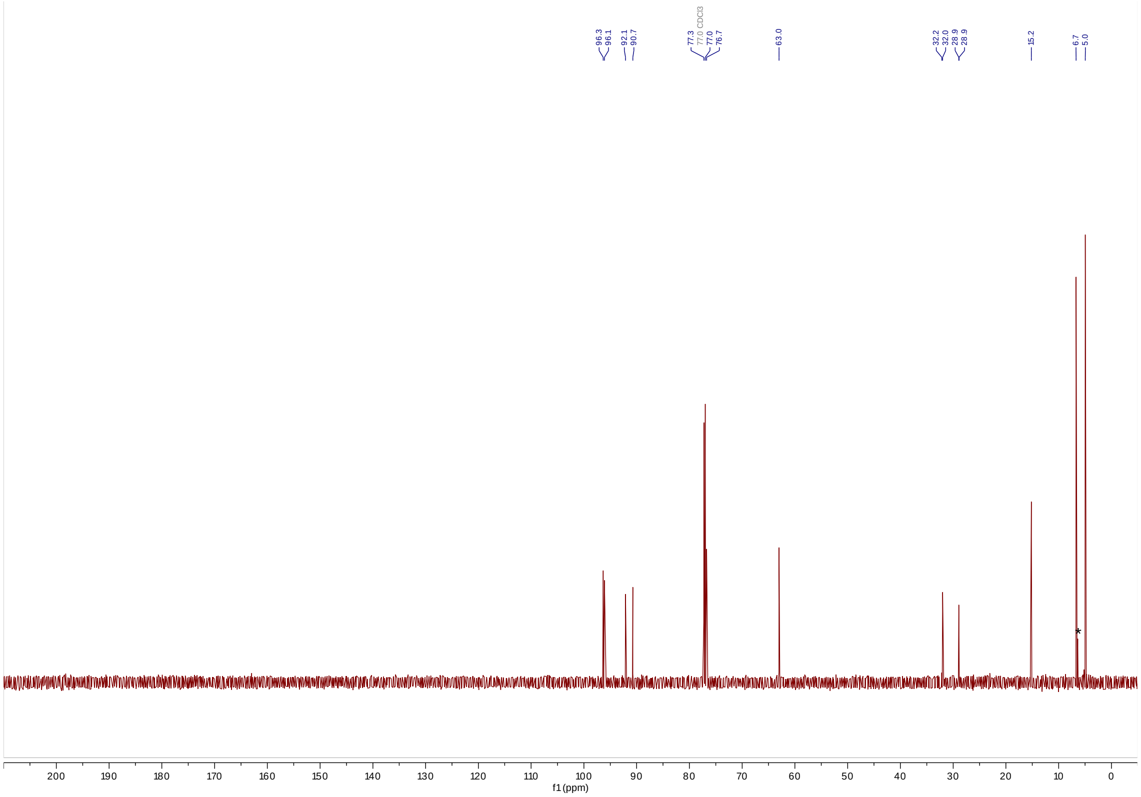

^{19}F-NMR (282 MHz, $CDCl_3$) spectra of **29**

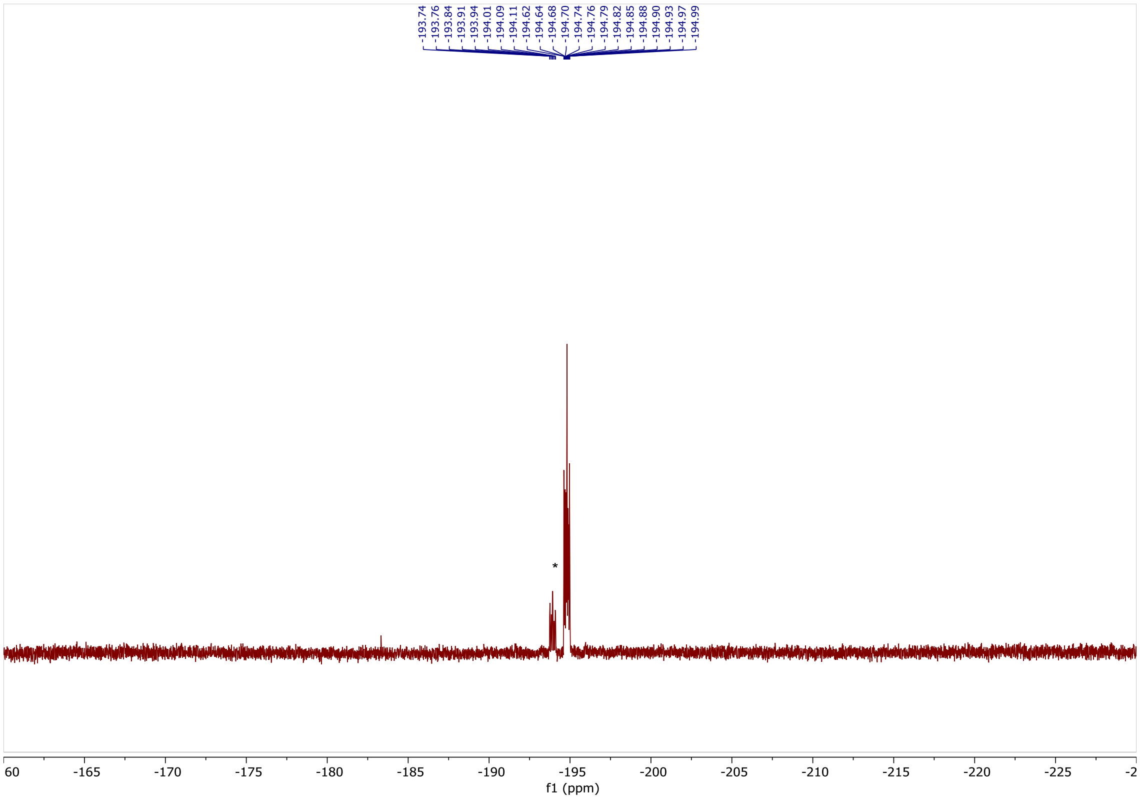

Compound 30: 4-bromo-1-ethoxy-2-methoxybutoxy)triethylsilane (See compound data)

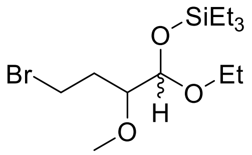

30

^{1}H-NMR (500 MHz, $CDCl_3$) spectra of **30**

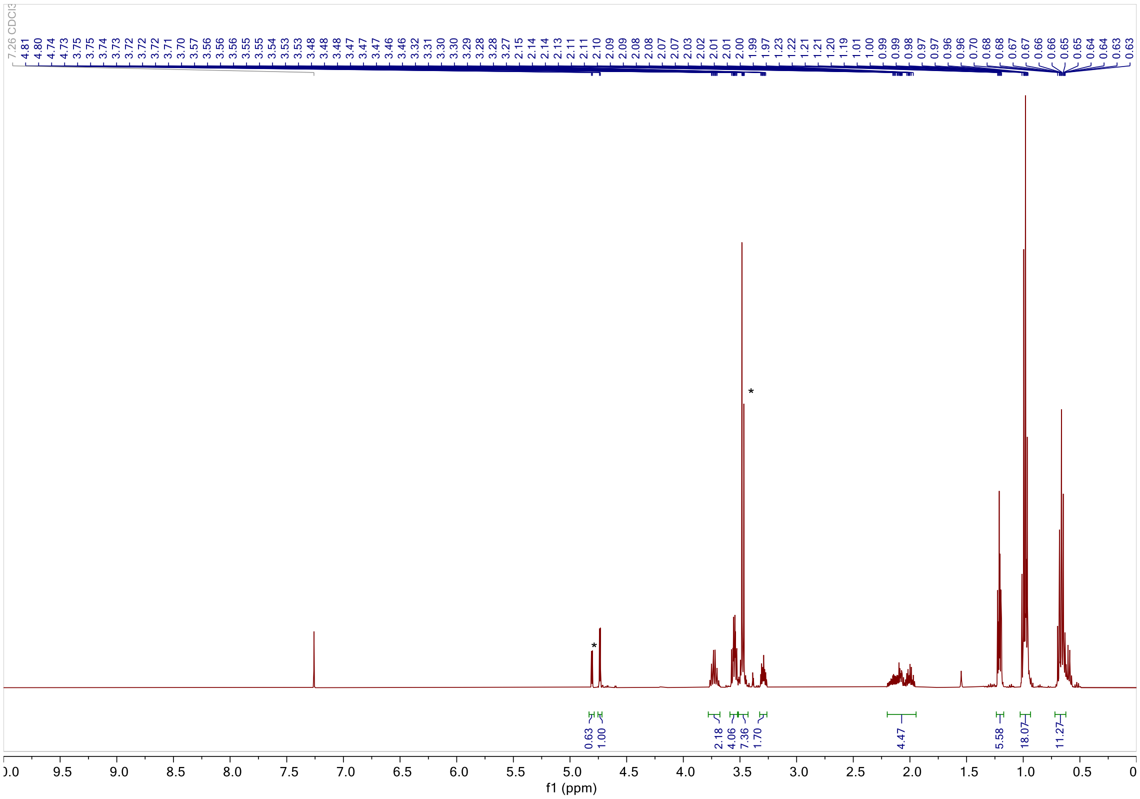

^{13}C-NMR (126 MHz, $CDCl_3$) spectra of **30**

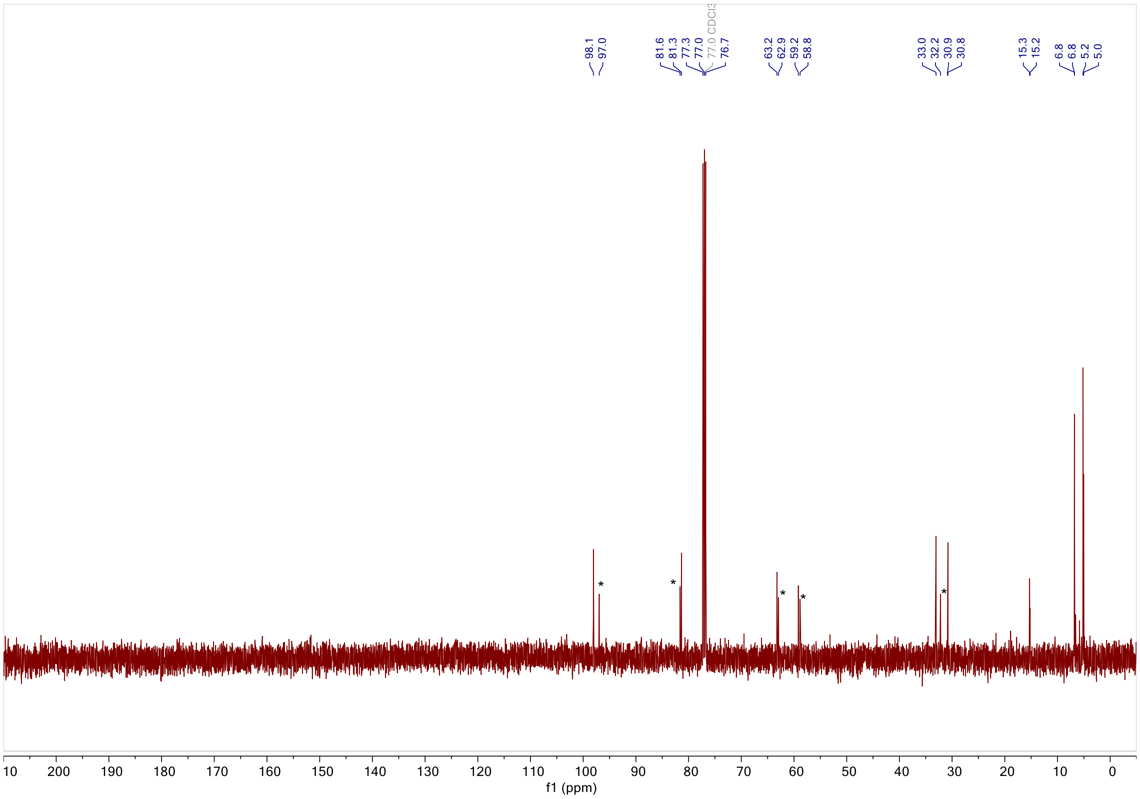

Compound 31: (4-bromo-1-ethoxy-2,2-difluorobutoxy)triethylsilane (See compound data)

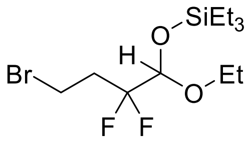

31

^{1}H-NMR (500 MHz, $CDCl_3$) spectra of **31**

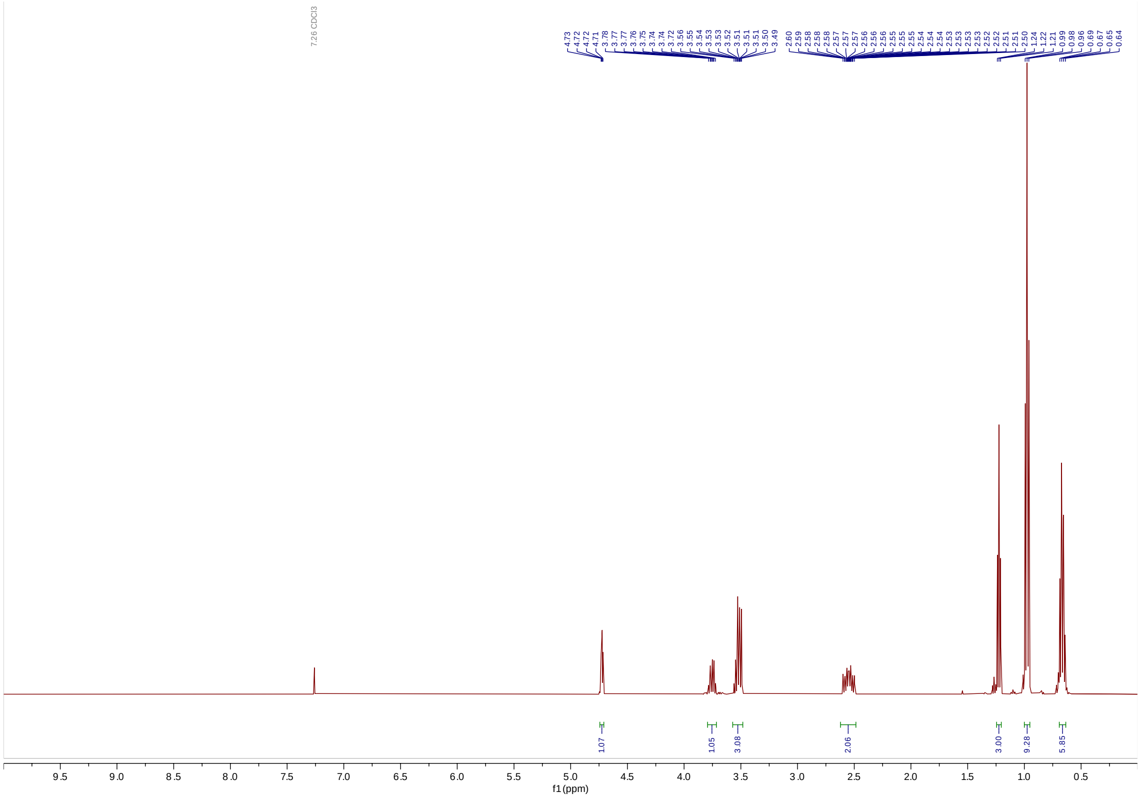

^{13}C-NMR (126 MHz, $CDCl_3$) spectra of **31**

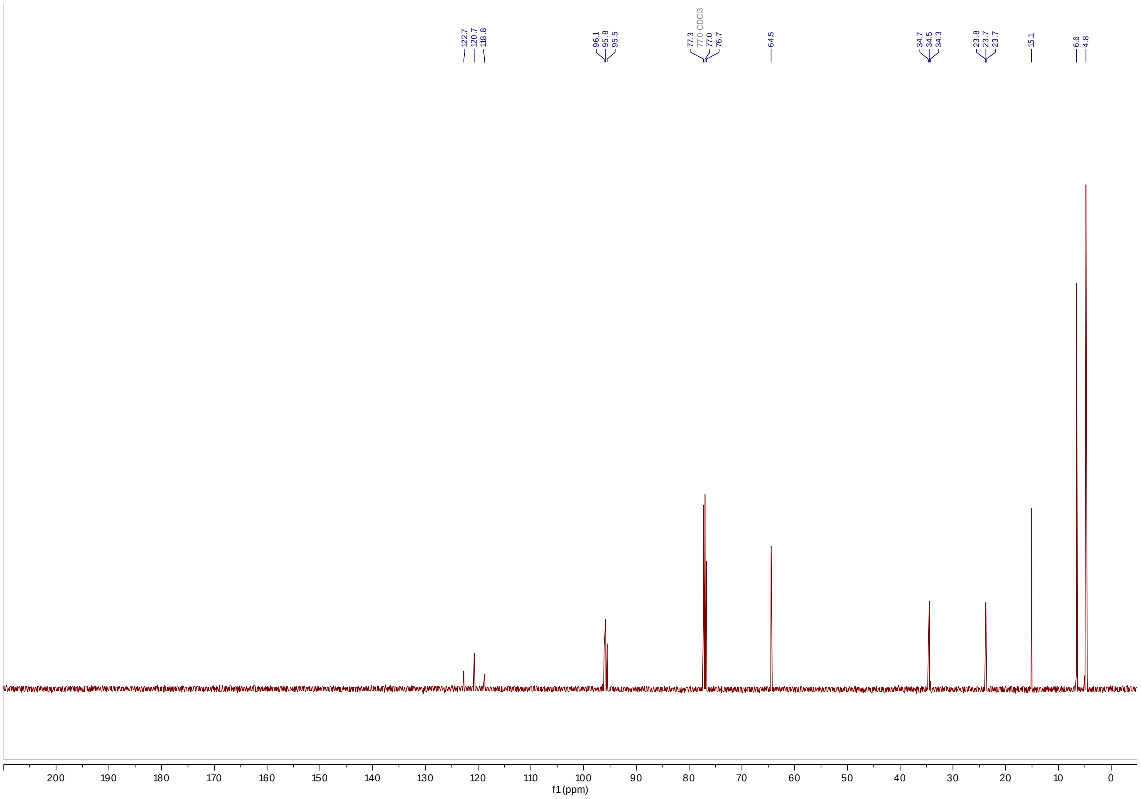

^{19}F-NMR (282 MHz, $CDCl_3$) spectra of **31**

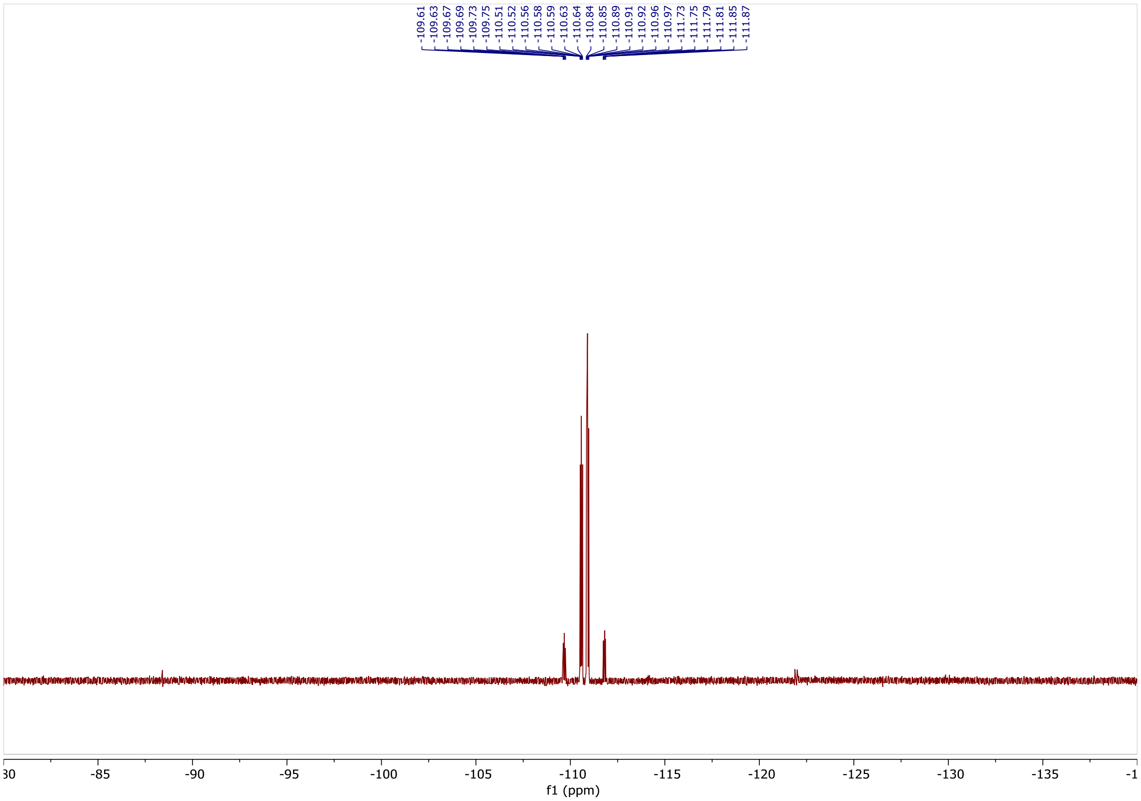

Compound 32: (1-ethoxy-2,2,2-trifluoroethoxy)triethylsilane (See compound data)

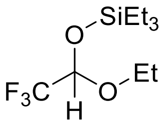

32

[1]**H-NMR** (500 MHz, $CDCl_3$) spectra of **32**

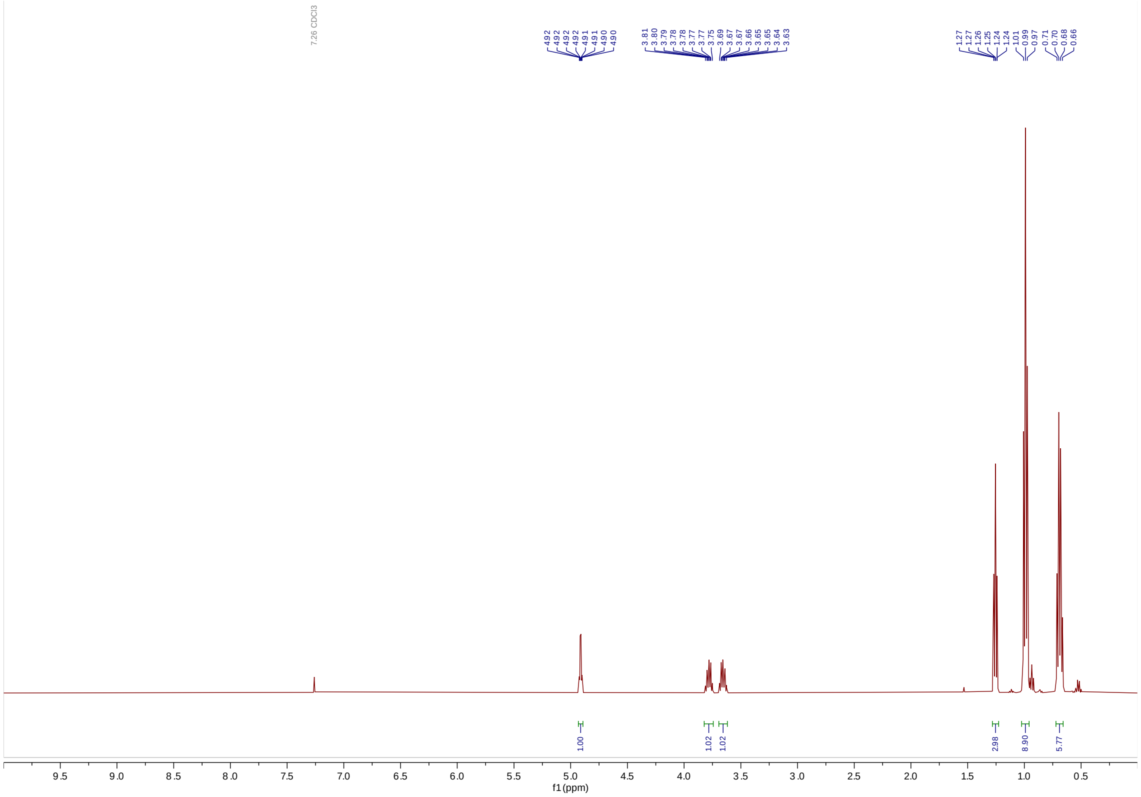

^{13}C-NMR (126 MHz, $CDCl_3$) spectra of **32**

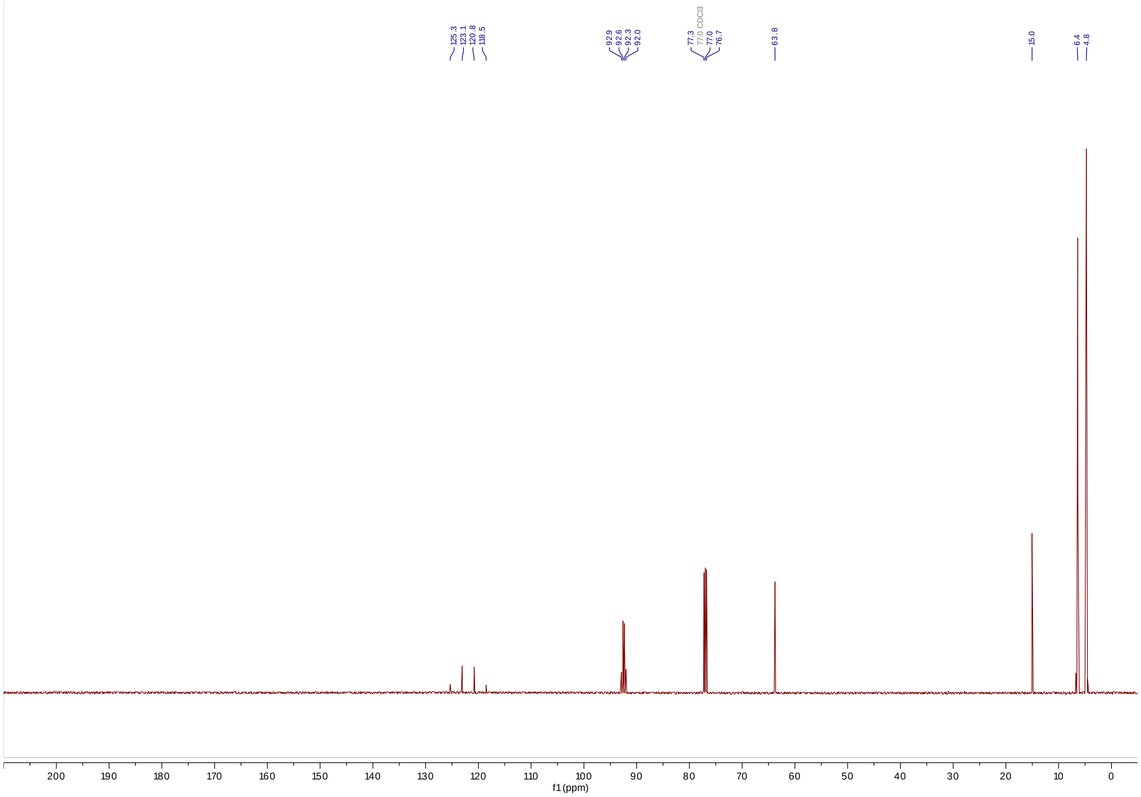

^{19}F-NMR (282 MHz, $CDCl_3$) spectra of **32**

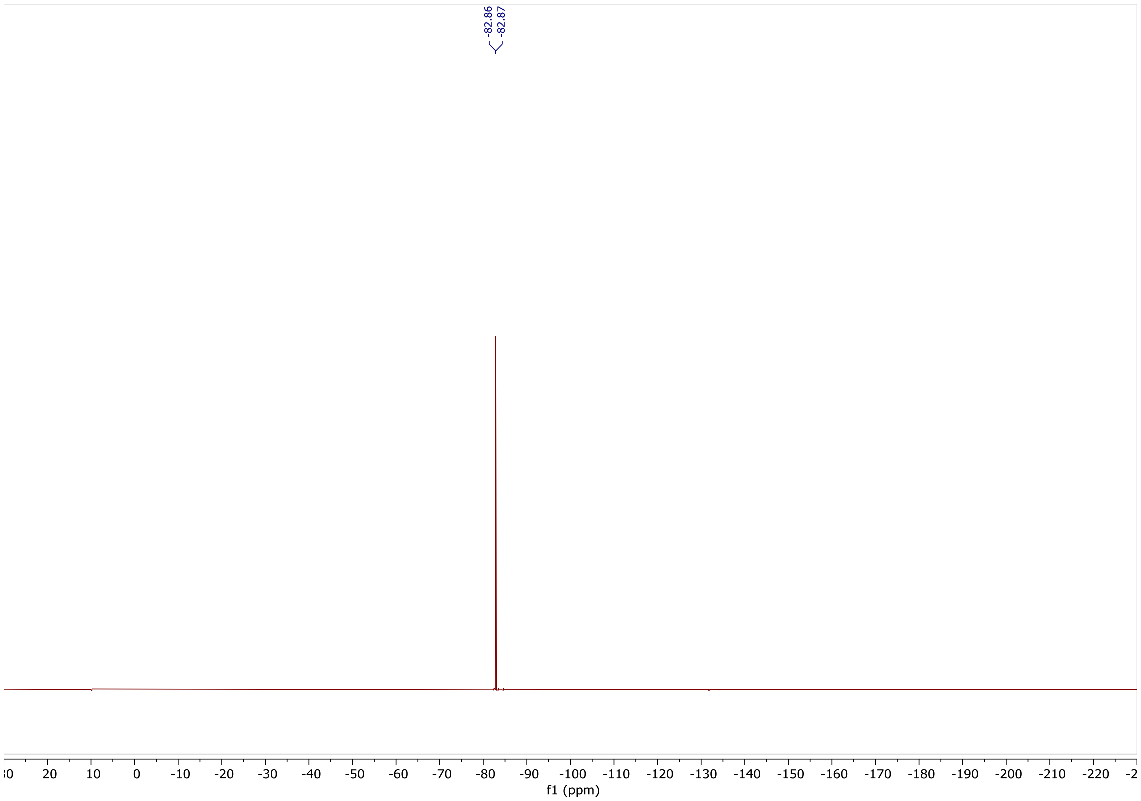

Compound 33: (2-(2-chloroethoxy)-1-ethoxyethoxy)triethylsilane (See compound data)

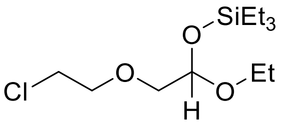

33

^{1}H-NMR (500 MHz, $CDCl_3$) spectra of **33**

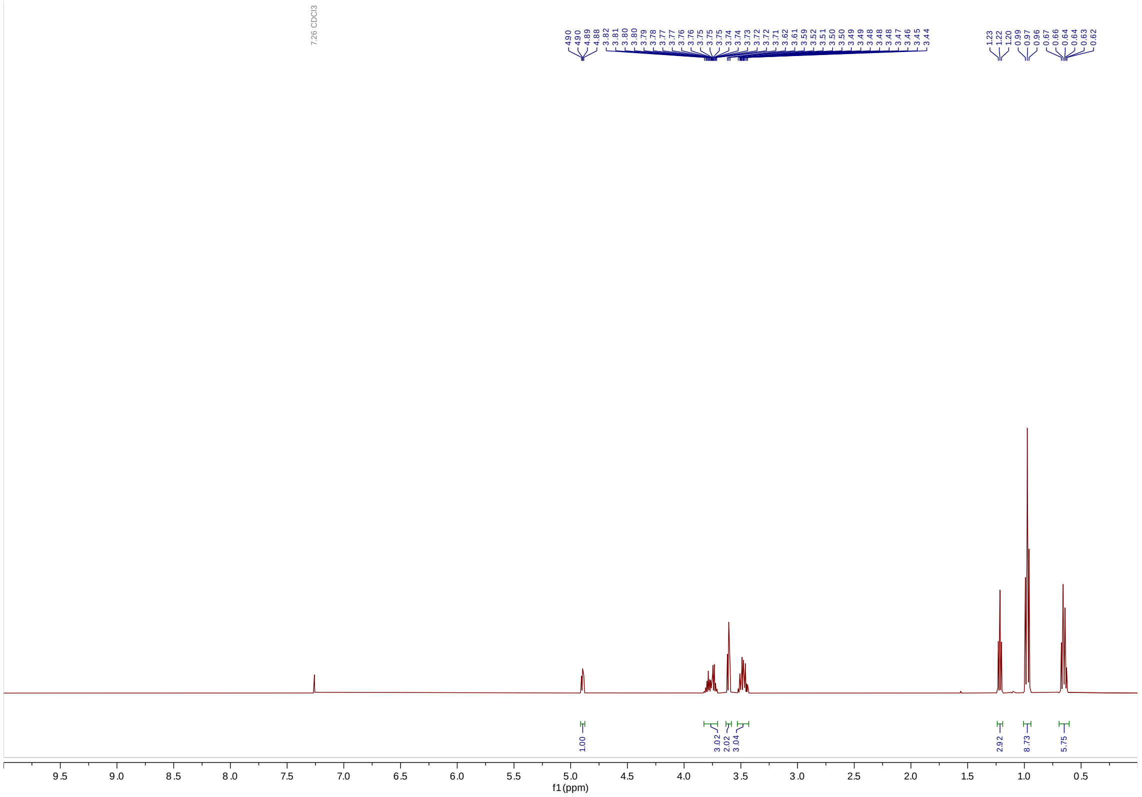

^{13}C-NMR (126 MHz, $CDCl_3$) spectra of **33**

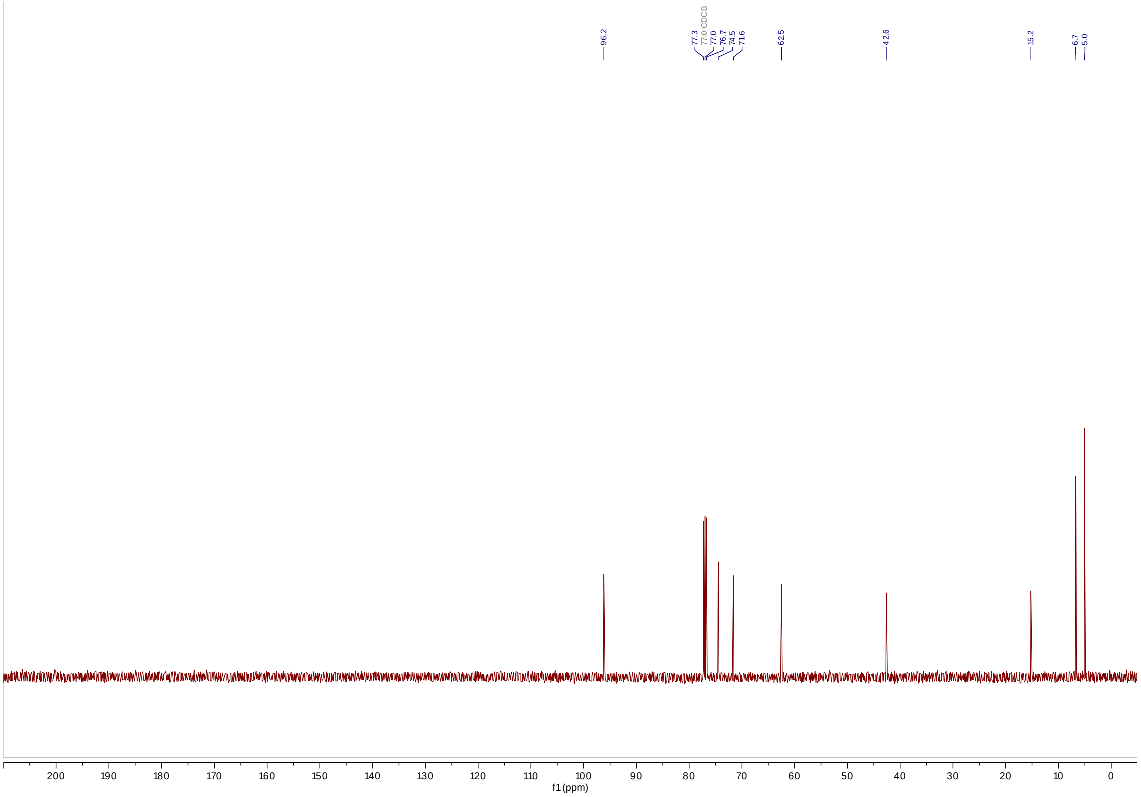

Compound 34: (2-(2-bromoethoxy)-1-ethoxyethoxy)triethylsilane (See compound data)

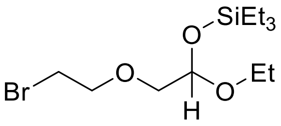

34

^{1}H-NMR (500 MHz, $CDCl_3$) spectra of **34**

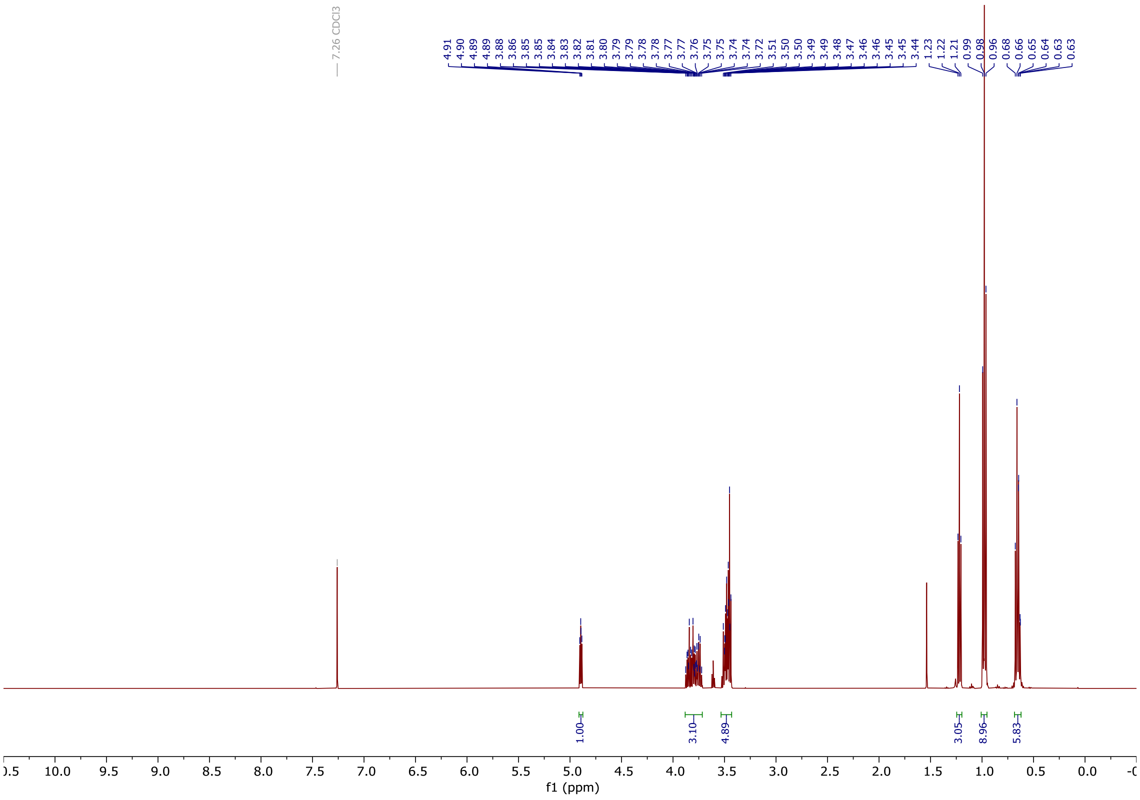

^{13}C-NMR (126 MHz, $CDCl_3$) spectra of **34**

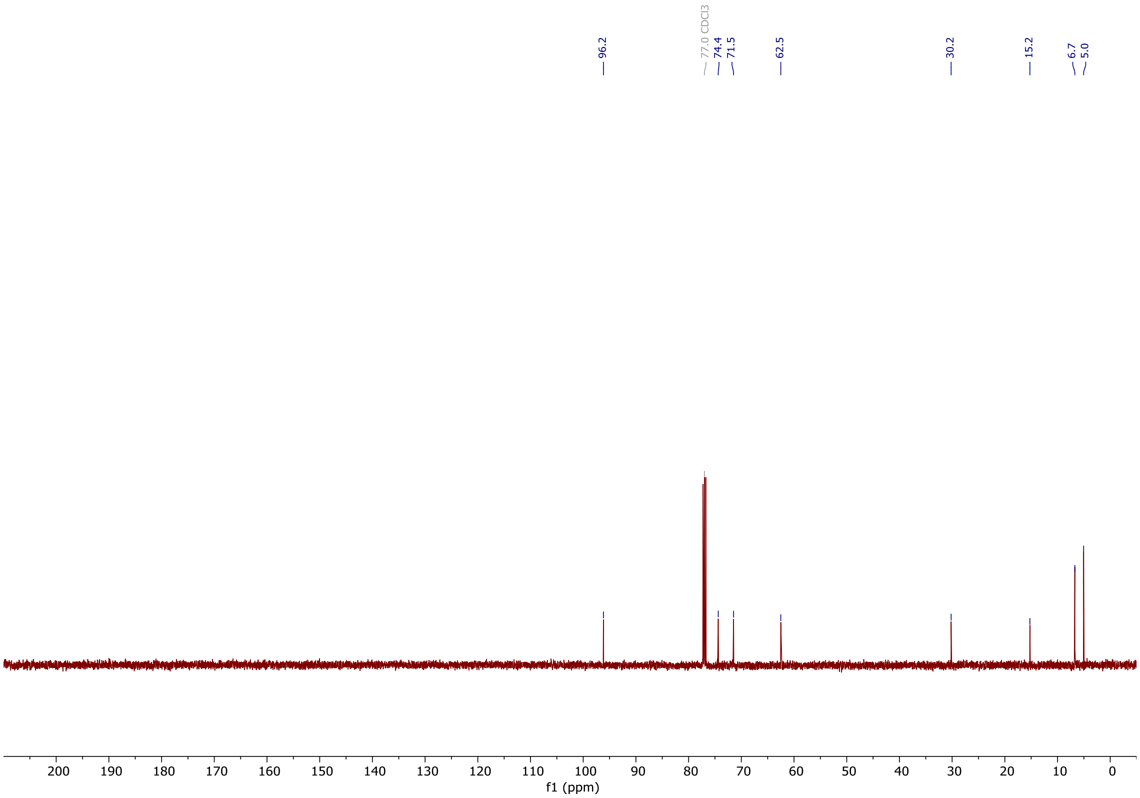

Compound 35: 5-ethoxy-3,3,12,12-tetraethyl-4,11-dioxa-3,12-disilatetradecane (See compound data)

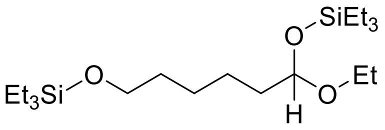

35

^{1}H-NMR (500 MHz, $CDCl_3$) spectra of **35**

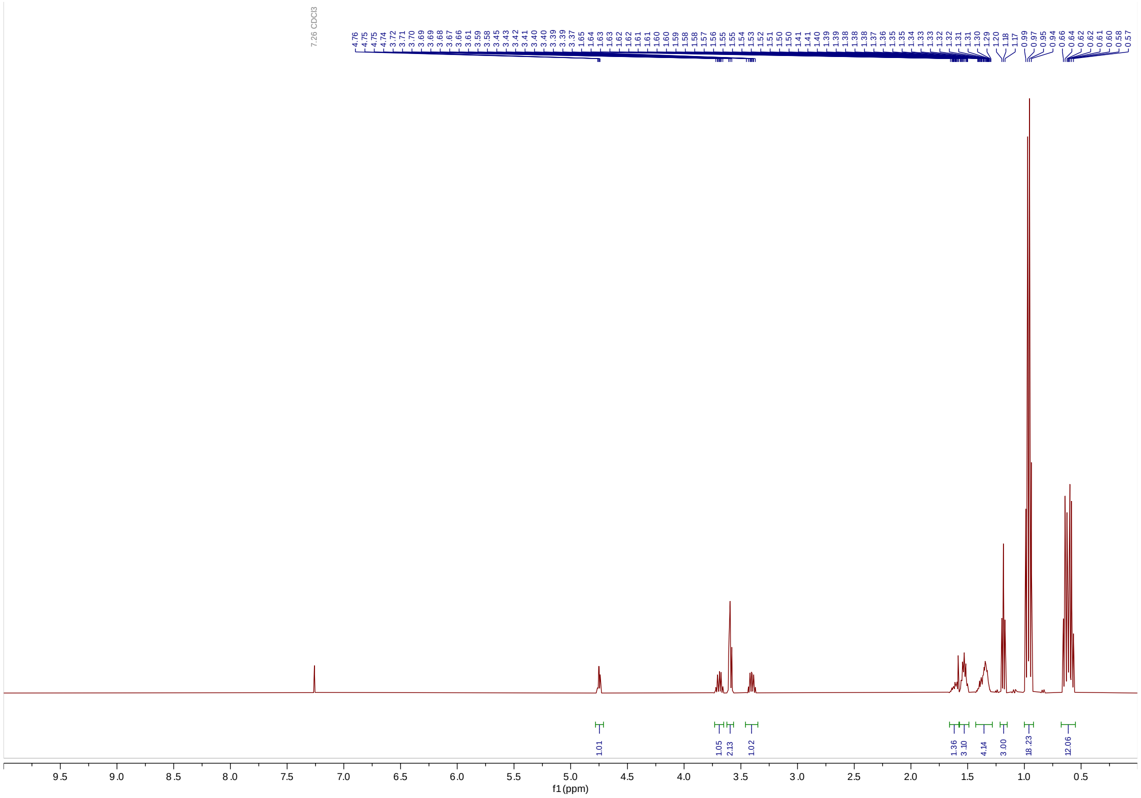

^{13}C-NMR (126 MHz, $CDCl_3$) spectra of **35**

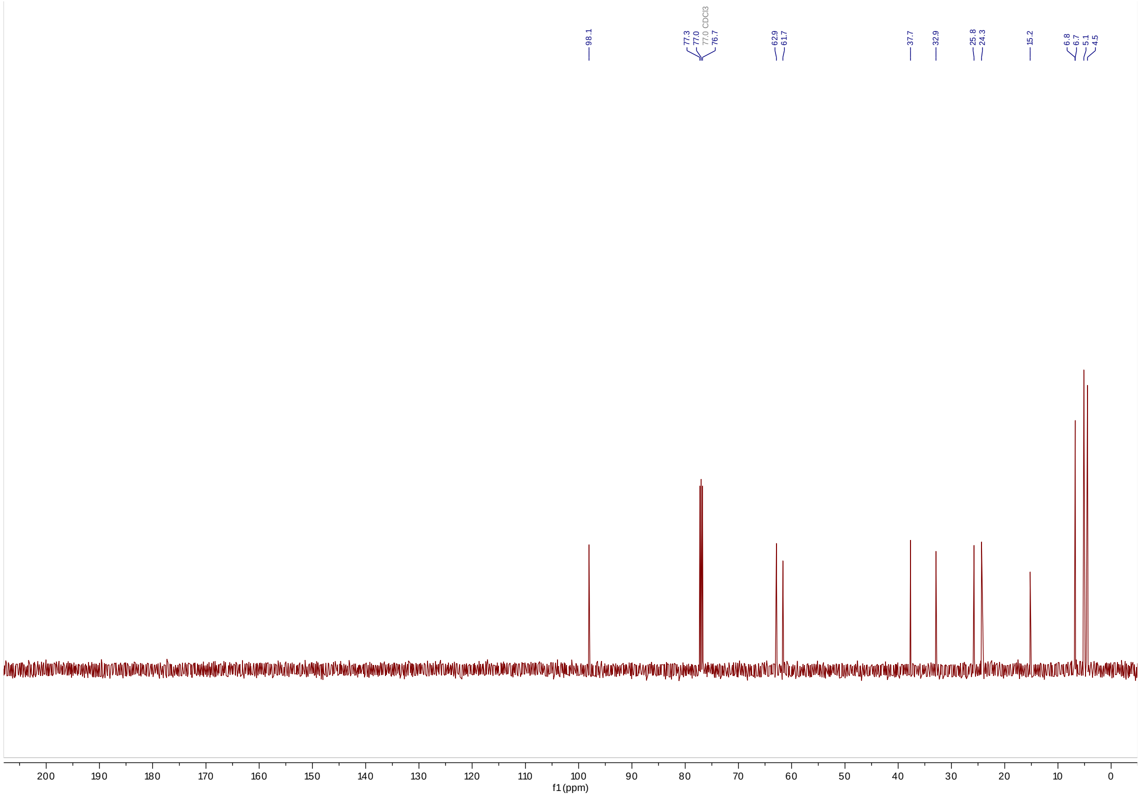

Compound 36: triethyl((tetrahydrofuran-2-yl)oxy)silane (See compound data)

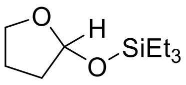

36

^{1}H-NMR (500 MHz, $CDCl_3$) spectra of **36**

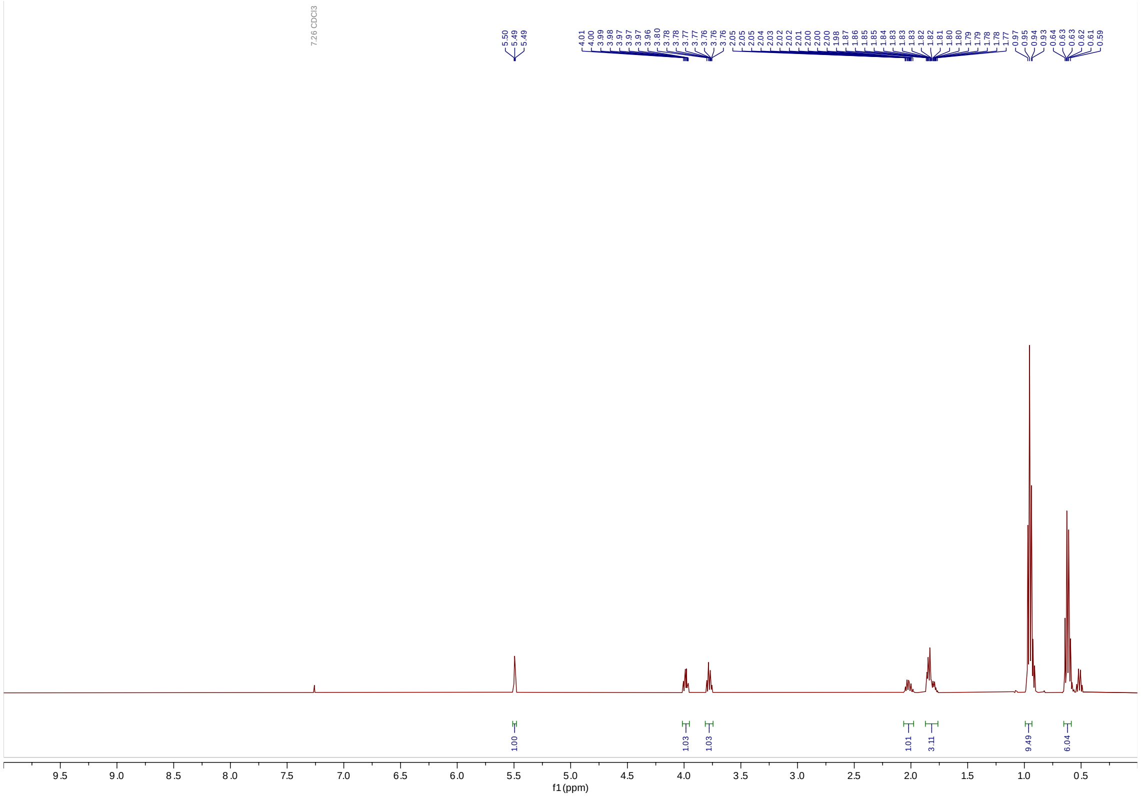

^{13}**C-NMR** (126 MHz, $CDCl_3$) spectra of **36**

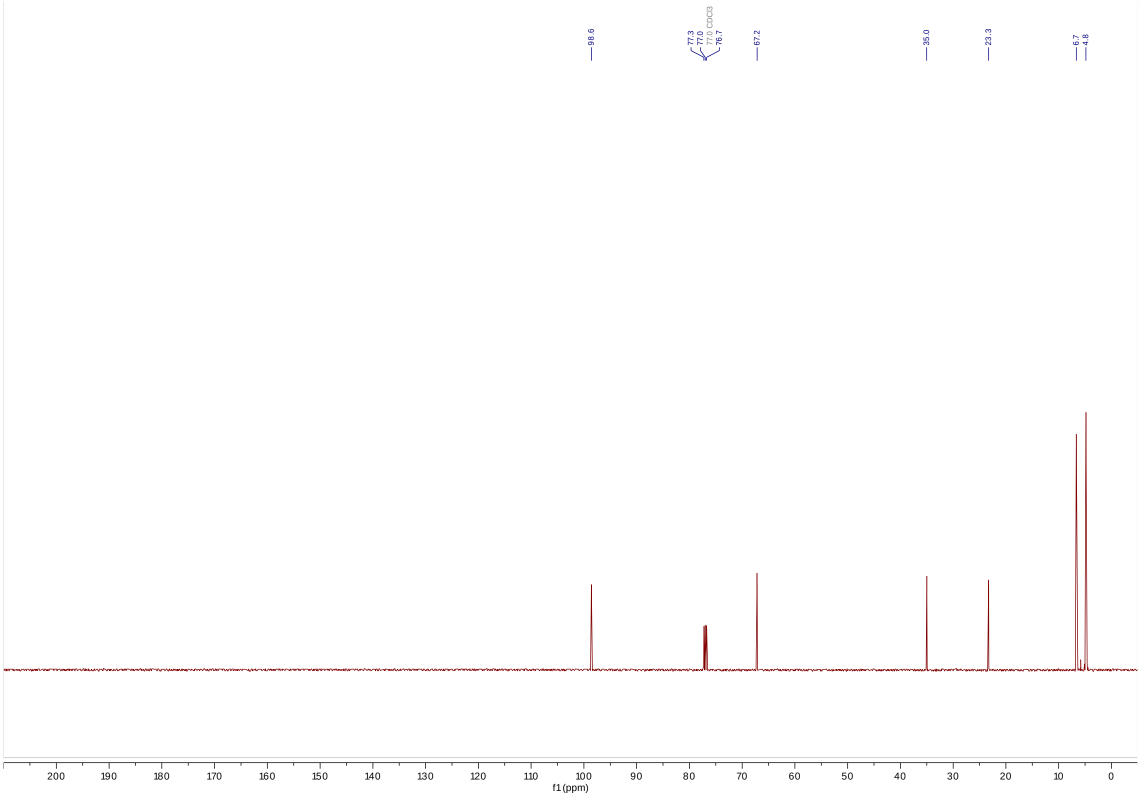

Compound 37: triethyl((tetrahydro-2H-pyran-2-yl)oxy)silane (See compound data)

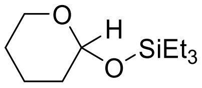

37

^{1}H-NMR (500 MHz, $CDCl_3$) spectra of **37**

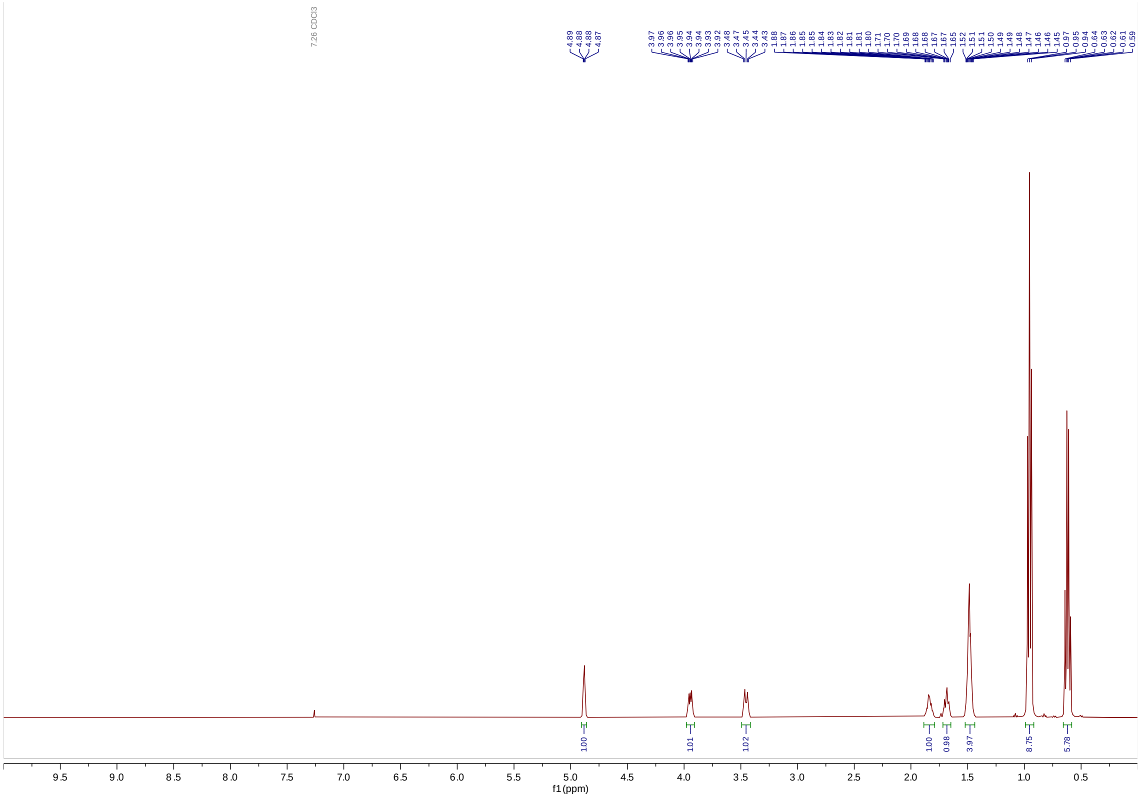

^{13}**C-NMR** (126 MHz, $CDCl_3$) spectra of **37**

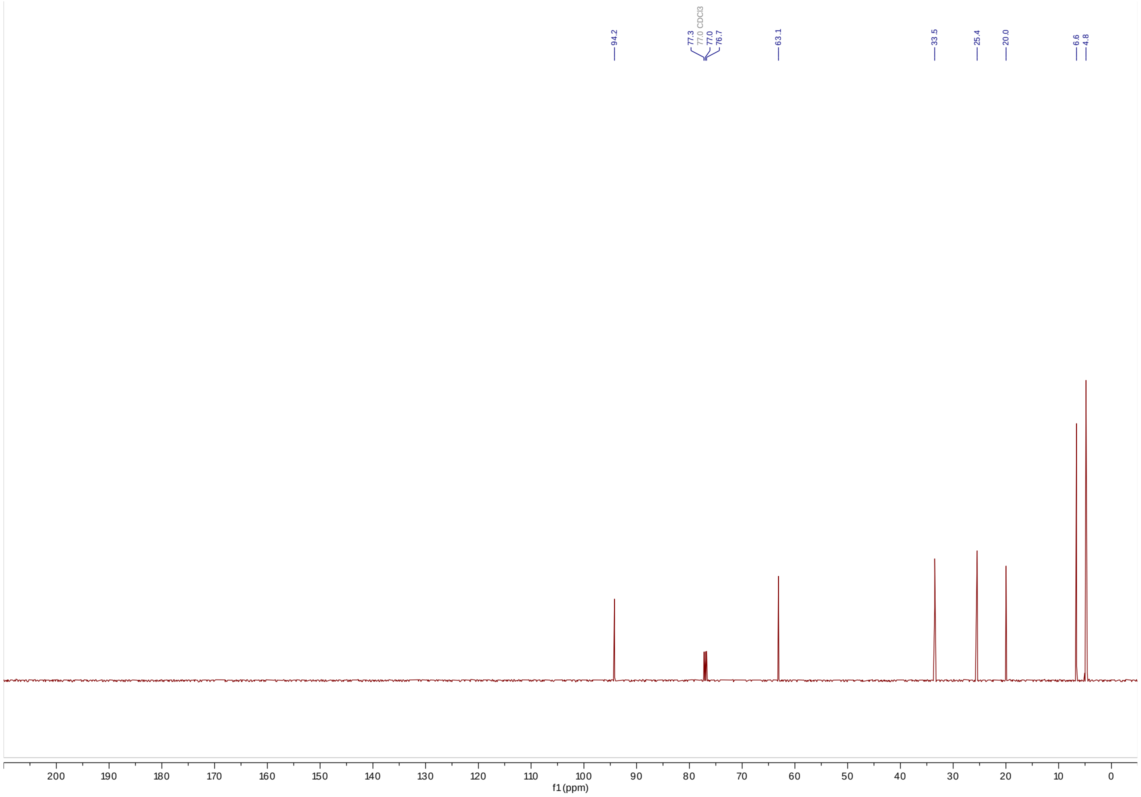

Compound 38: triethyl((oxacyclohexadecan-2-yl)oxy)silane (See compound data)

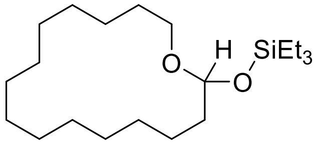

38

^{1}H-NMR (500 MHz, $CDCl_3$) spectra of **38**

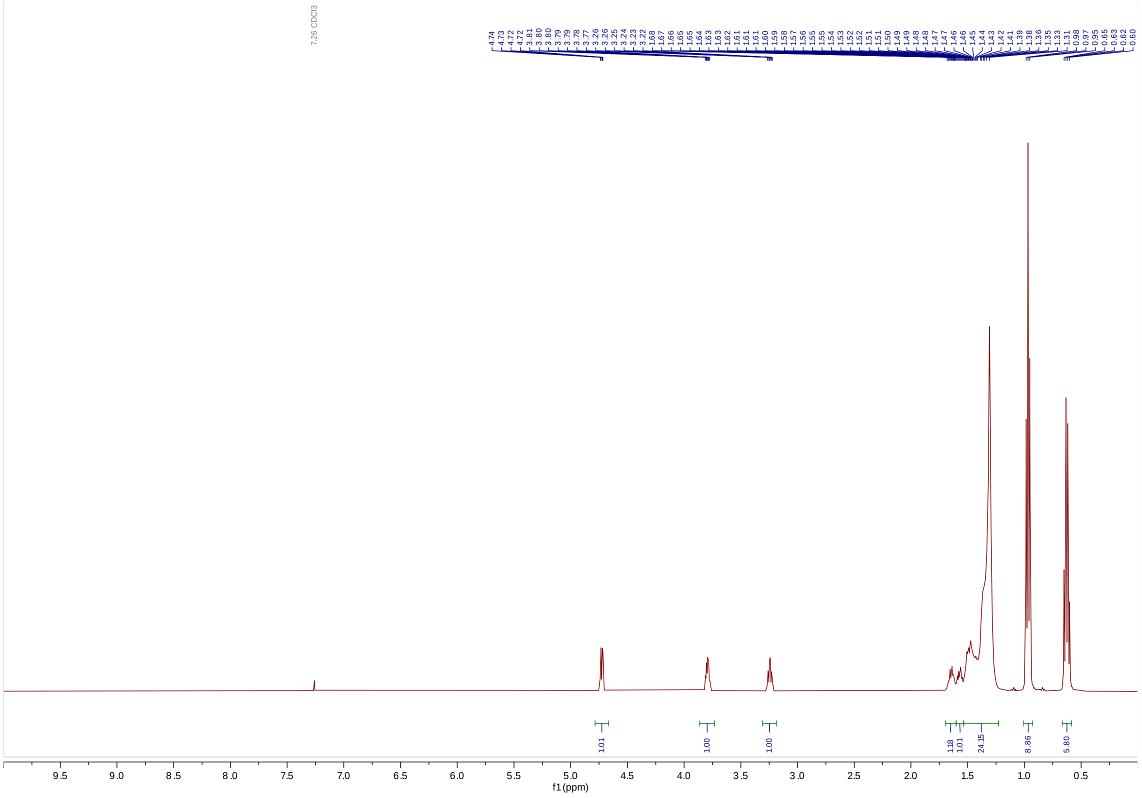

^{13}**C-NMR** (126 MHz, $CDCl_3$) spectra of **38**

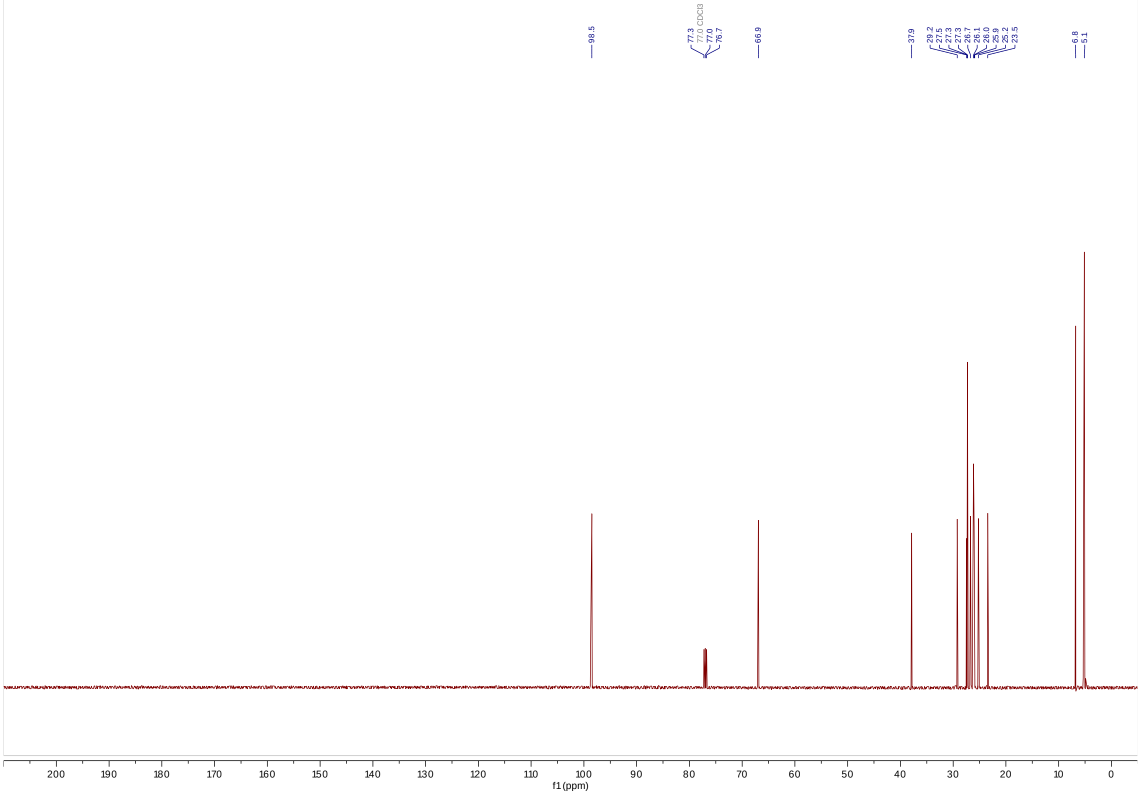

Compound 39: triethyl((oxacycloheptadec-8-en-2-yl)oxy)silane (See compound data)

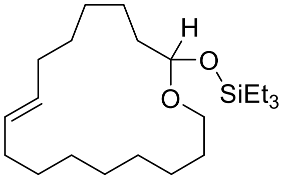

39

^{1}H-NMR (500 MHz, $CDCl_3$) spectra of **39**

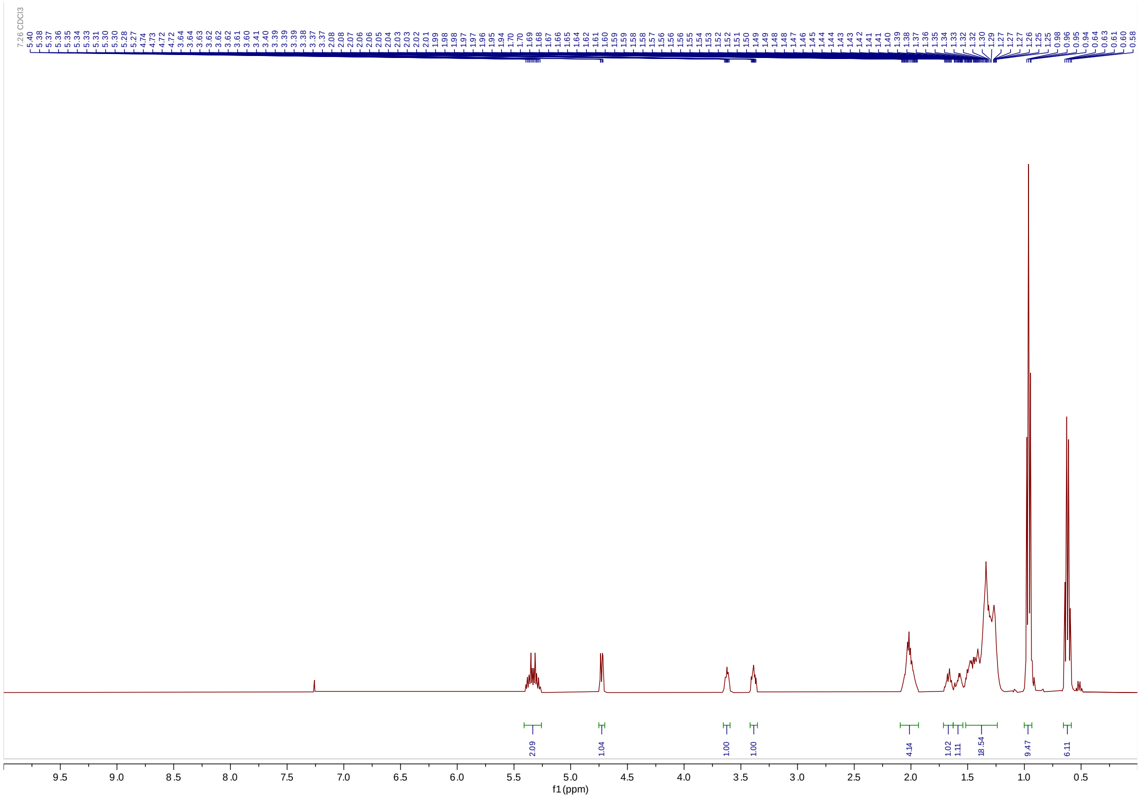

^{13}C-NMR (126 MHz, $CDCl_3$) spectra of **39**

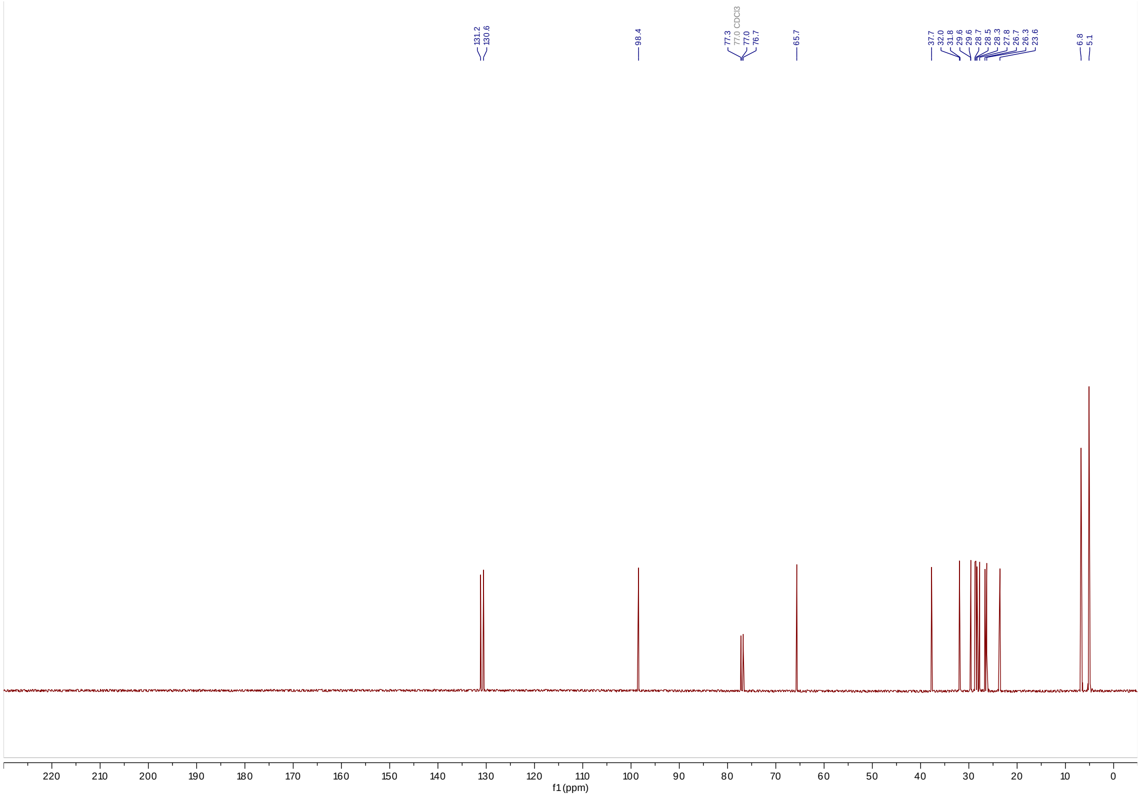

Compound 40: triethyl(((Z)-1-(((Z)-icos-11-en-1-yl)oxy)icos-11-en-1-yl)oxy)silane (See compound data)

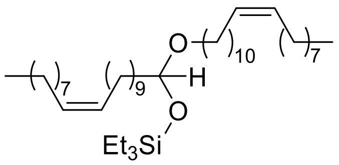

40

^{1}H-NMR (500 MHz, $CDCl_3$) spectra of **40**

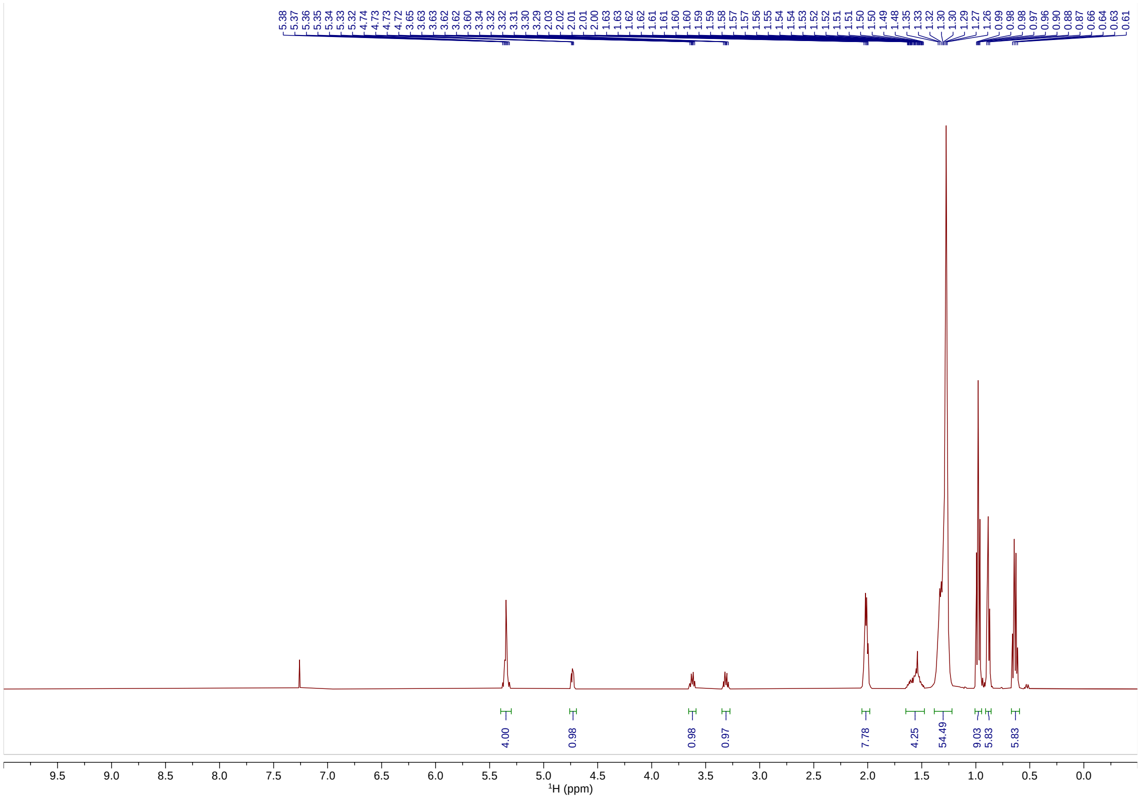

^{13}C-NMR (126 MHz, $CDCl_3$) spectra of **40**

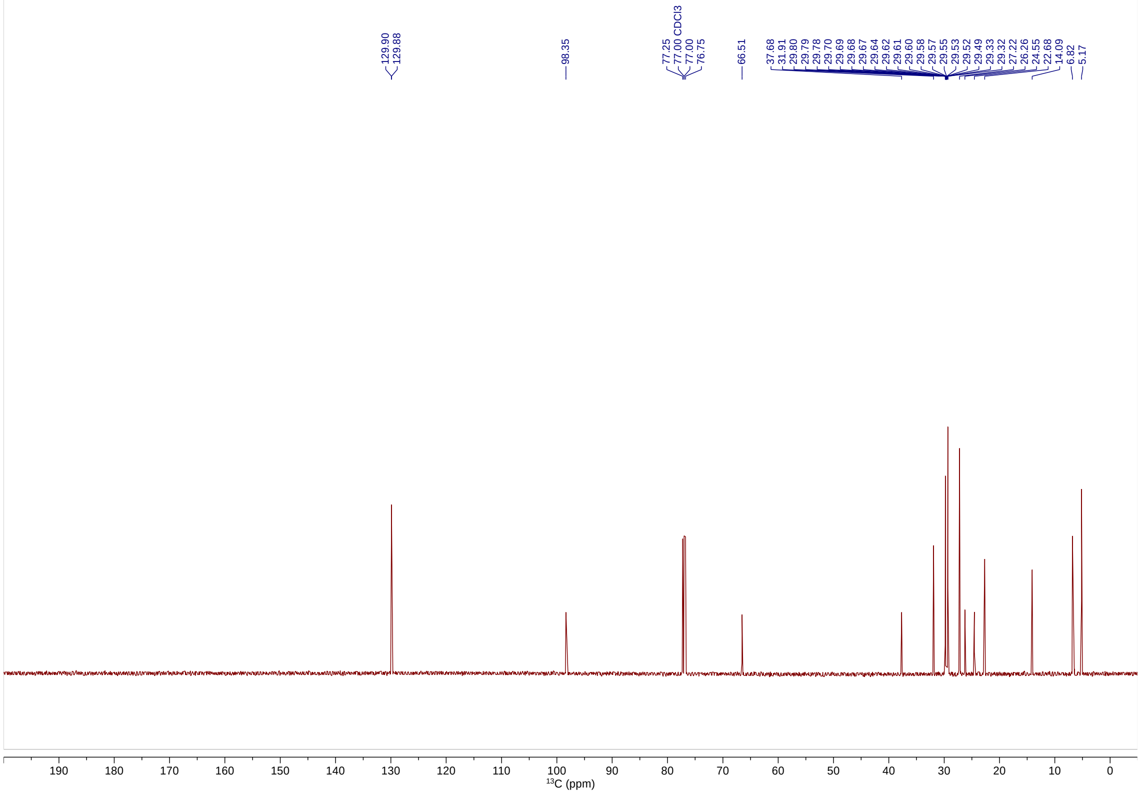

^{1}H-NMR (500 MHz, $CDCl_3$) spectra of **Jojoba oil**

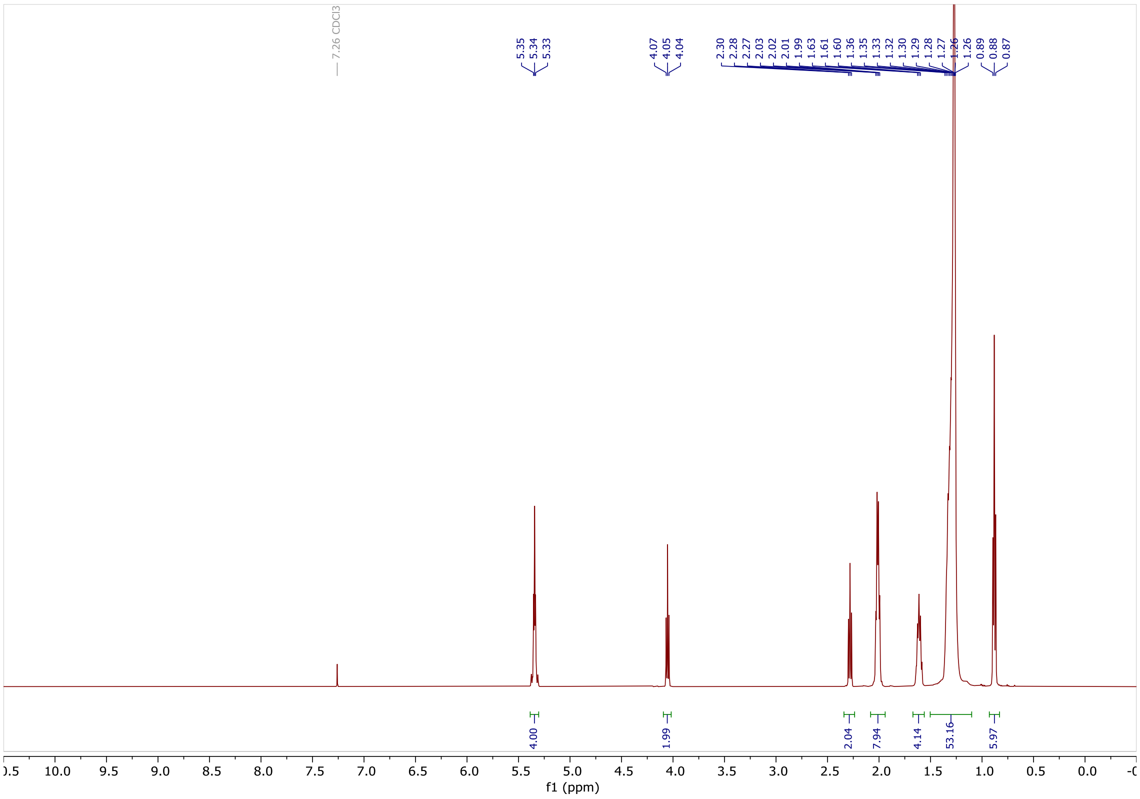

^{13}**C-NMR** (126 MHz, $CDCl_3$) spectra of **Jojoba oil**

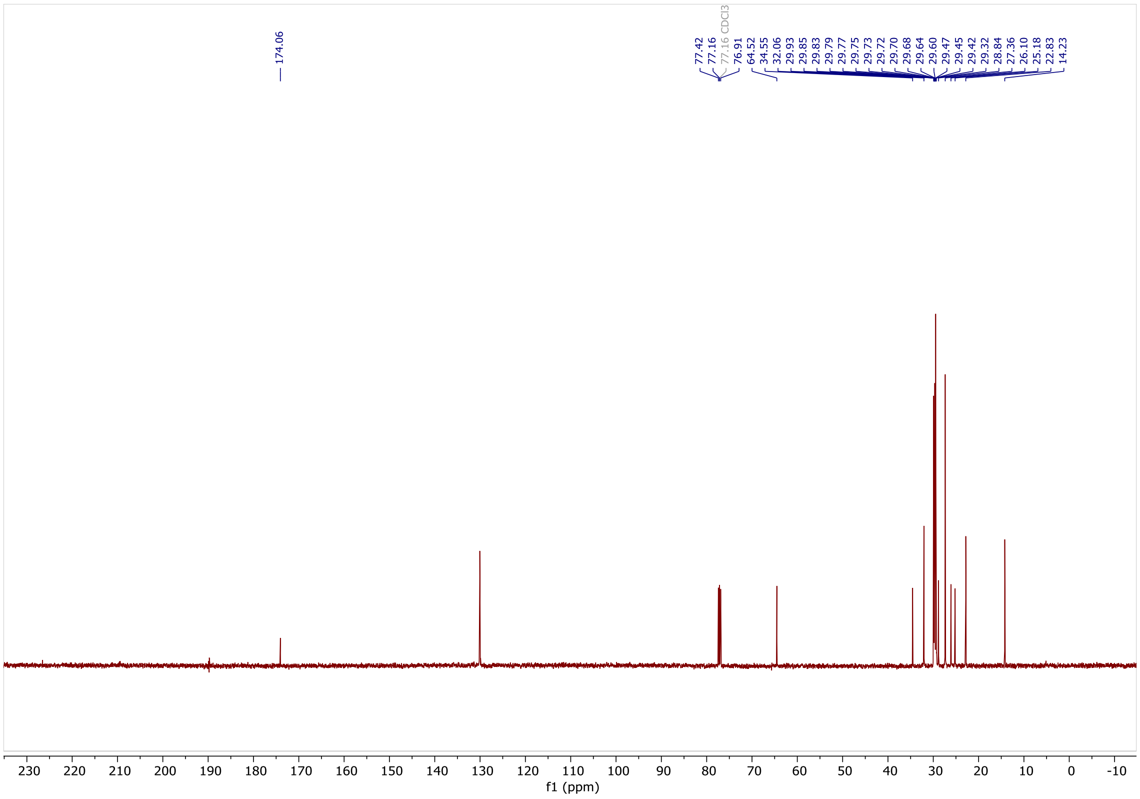

Compound 41: 3,3,13,13-tetraethyl-5,11-di((8Z,10E,12E)-heptadeca-8,10,12-trien-1-yl)-8-(((9Z,11E,13E)-1-((triethylsilyl)oxy)octadeca-9,11,13-trien-1-yl)oxy)-4,6,10,12-tetraoxa-3,13-disilapentadecane (See compound data)

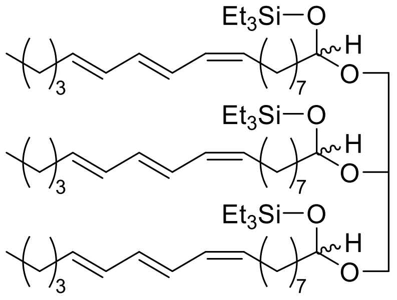

41

^{1}H-NMR (500 MHz, $CDCl_3$) spectra of **41**

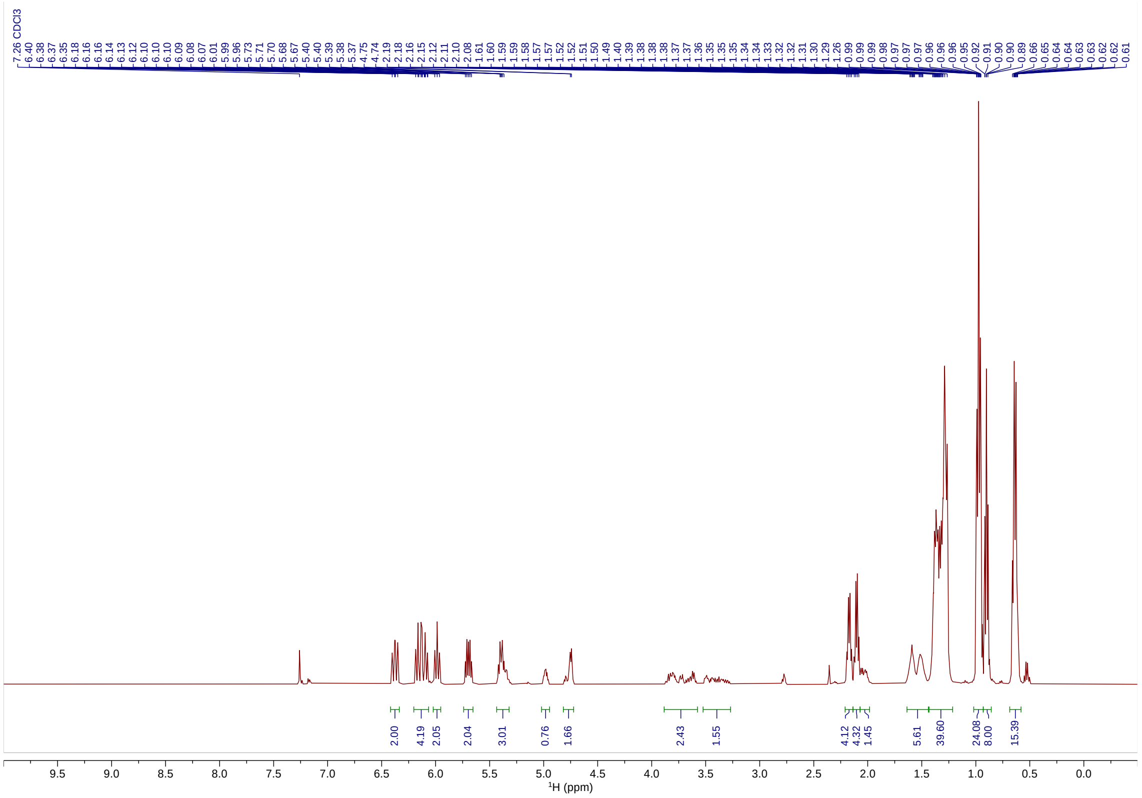

^{13}C-NMR (126 MHz, $CDCl_3$) spectra of **41**

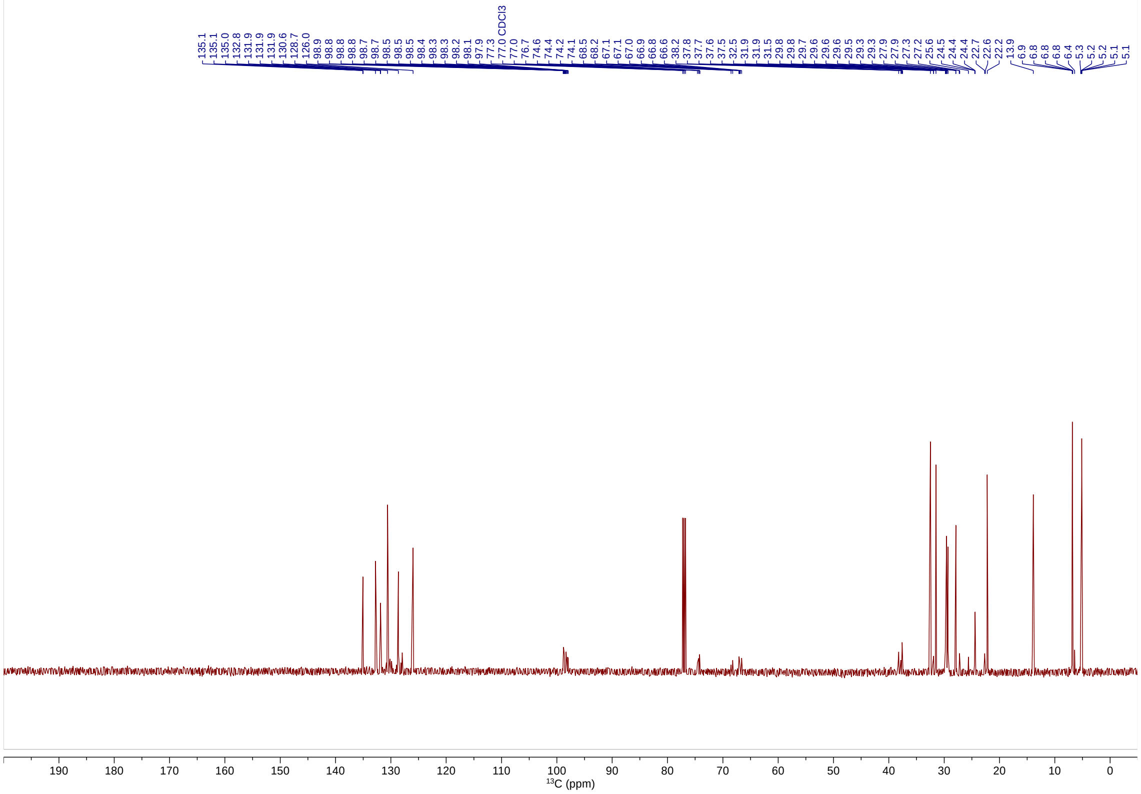

^{1}H-NMR (500 MHz, $CDCl_3$) spectra of **Tung oil**

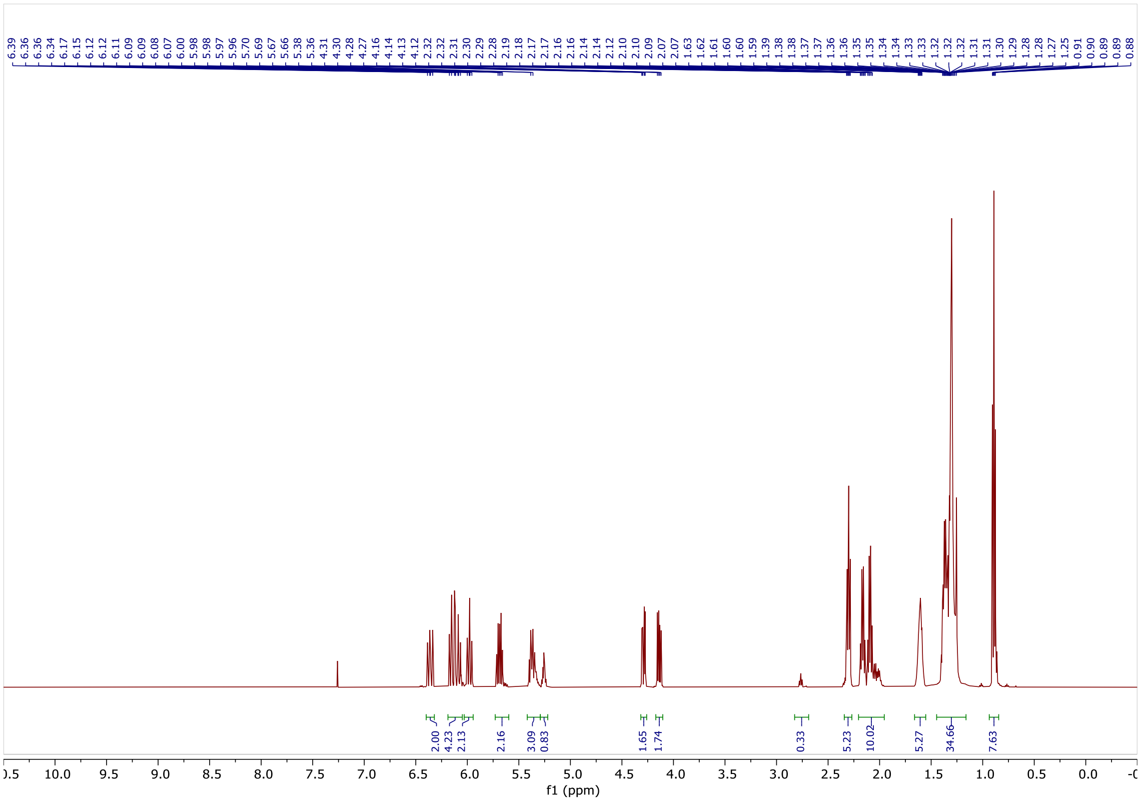

^{13}C-NMR (126 MHz, $CDCl_3$) spectra of **Tung oil**

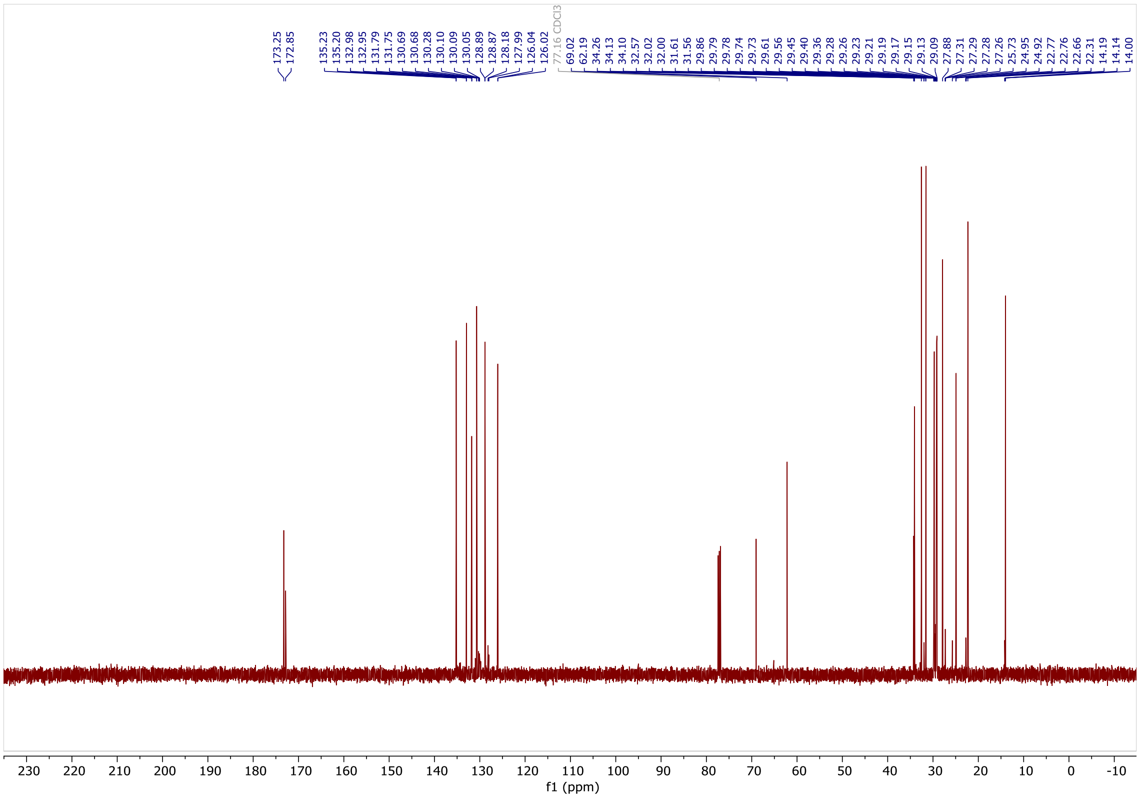

Compound 42: (1-(but-3-yn-1-yloxy)-3-phenylpropoxy)triethylsilane (See compound data)

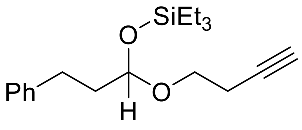

42

^{1}H-NMR (500 MHz, $CDCl_3$) spectra of **42**

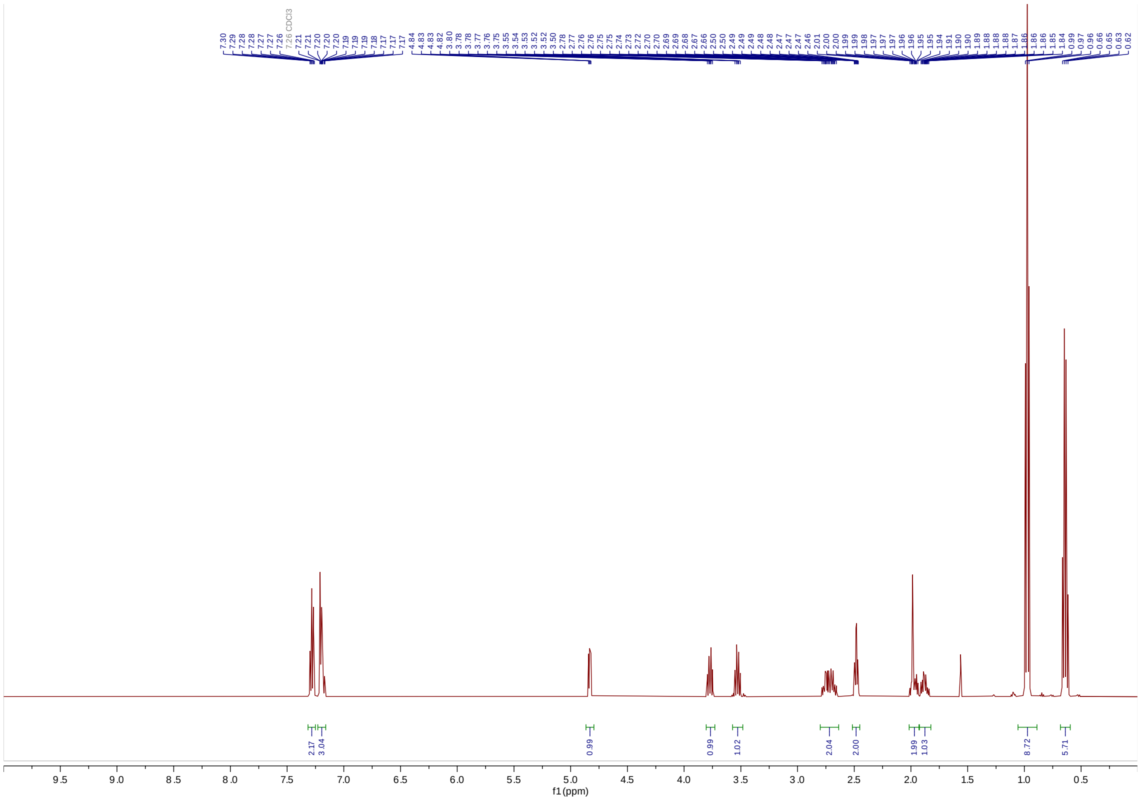

^{13}**C-NMR** (126 MHz, $CDCl_3$) spectra of **42**

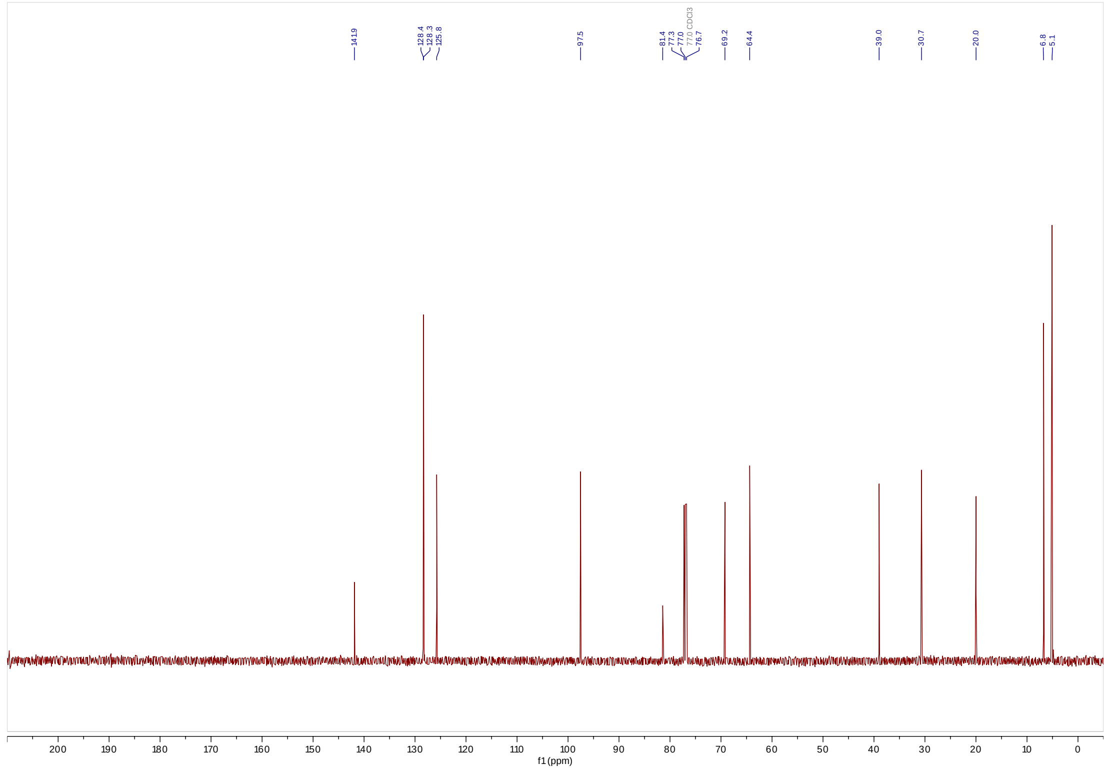

Compound 43: (1-(but-3-yn-1-yloxy)-3-phenylpropoxy)triethylsilane (See compound data)

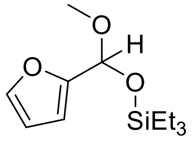

43

^{1}H-NMR (500 MHz, C_6D_6) spectra of **43**

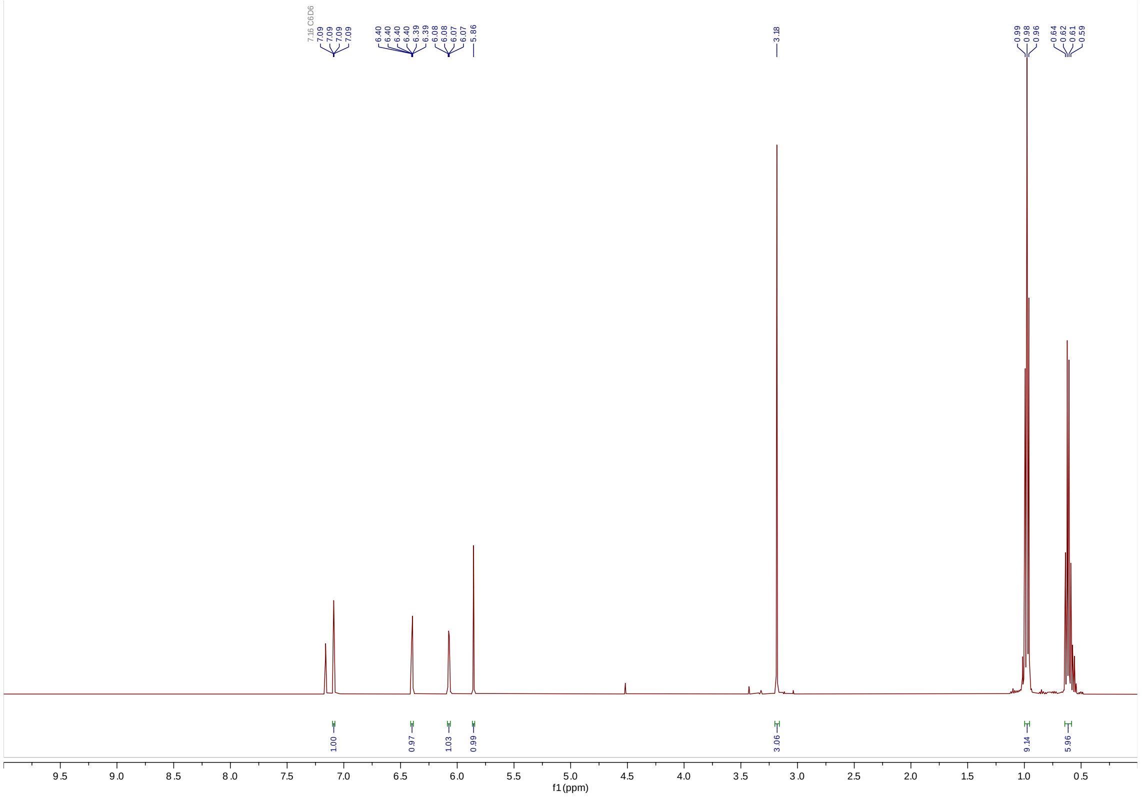

^{13}**C-NMR** (126 MHz, C_6D_6) spectra of **43**

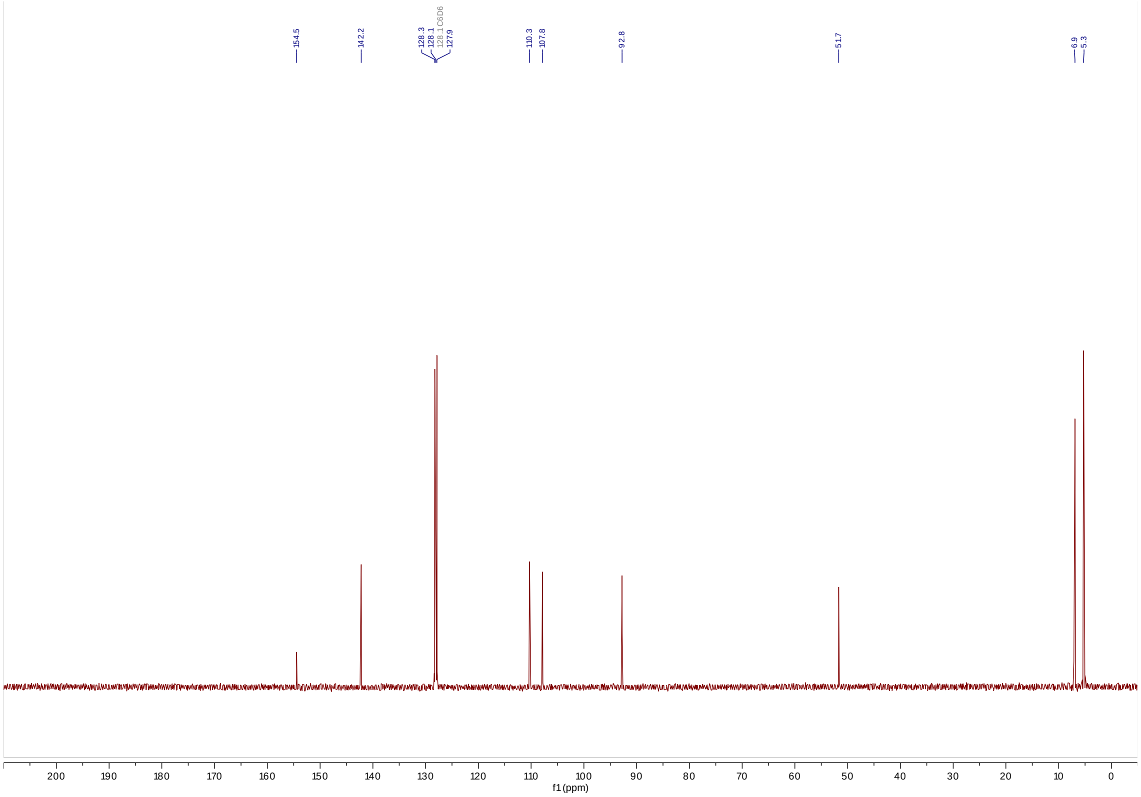

Compound 44: (1-ethoxy-2-(thiophen-2-yl)ethoxy)triethylsilane (See compound data)

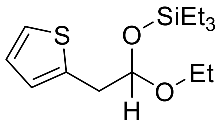

44

^{1}H-NMR (500 MHz, $CDCl_3$) spectra of **44**

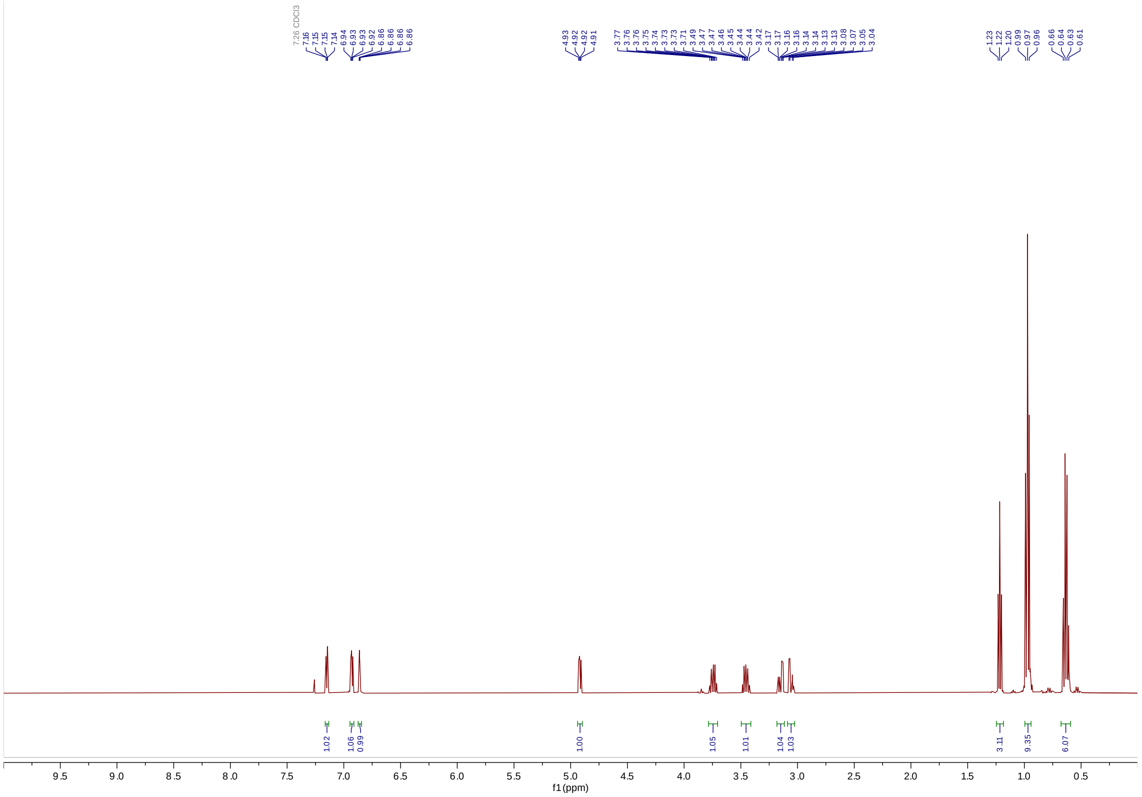

^{13}C-NMR (126 MHz, $CDCl_3$) spectra of **44**

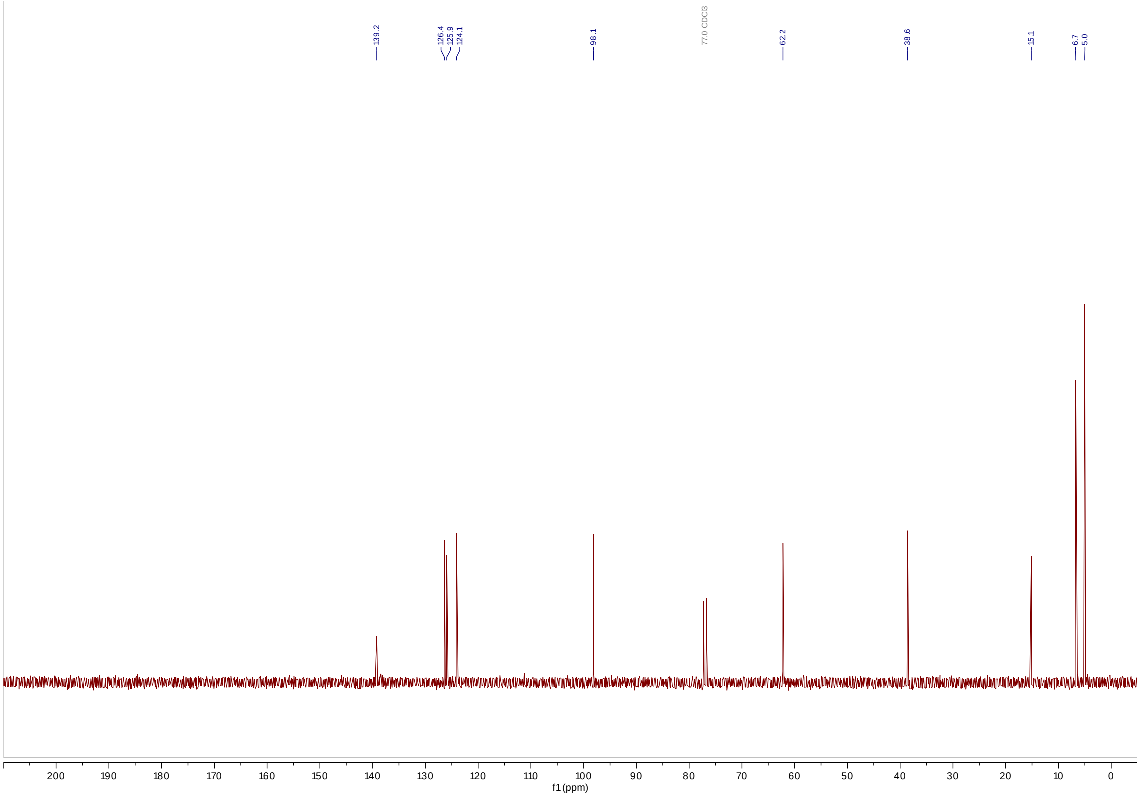

Compound 45: ((5-(1,2-dithiolan-3-yl)-1-methoxypentyl)oxy)triethylsilane (See compound data)

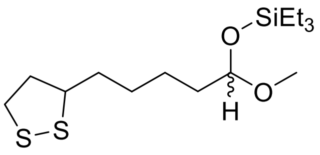

45

^{1}H-NMR (500 MHz, $CDCl_3$) spectra of **45**

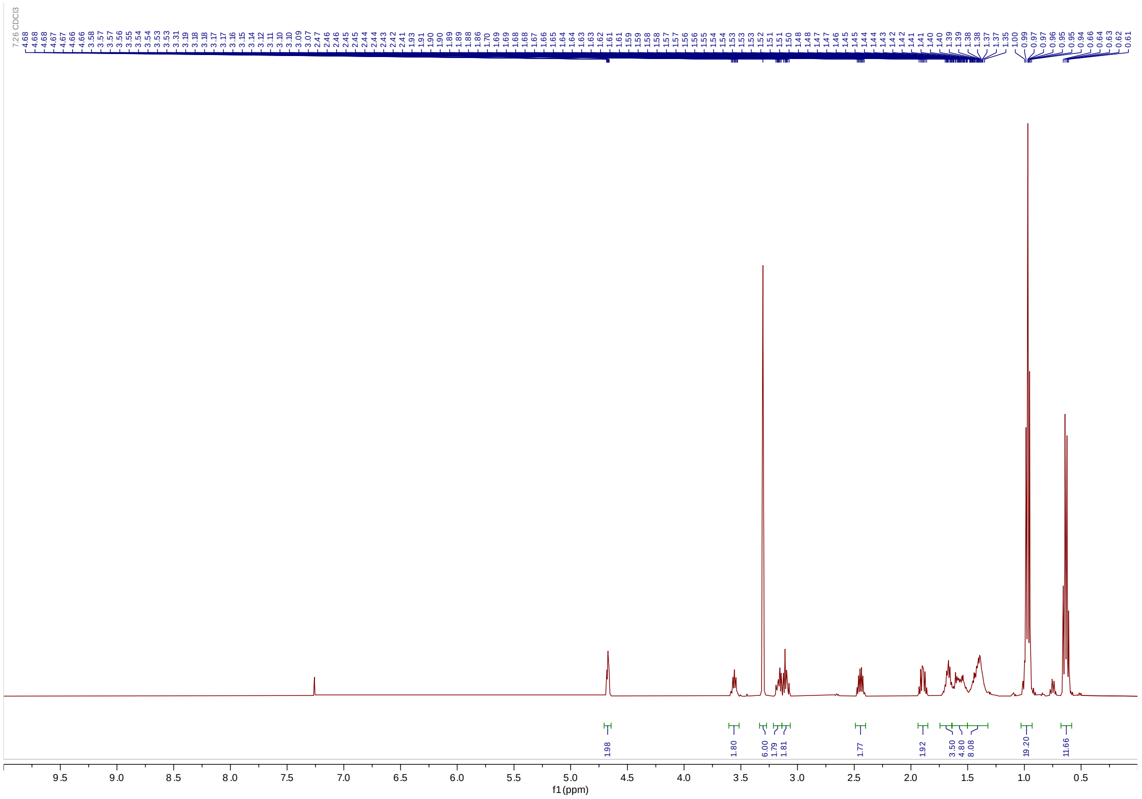

^{13}**C-NMR** (126 MHz, $CDCl_3$) spectra of **45**

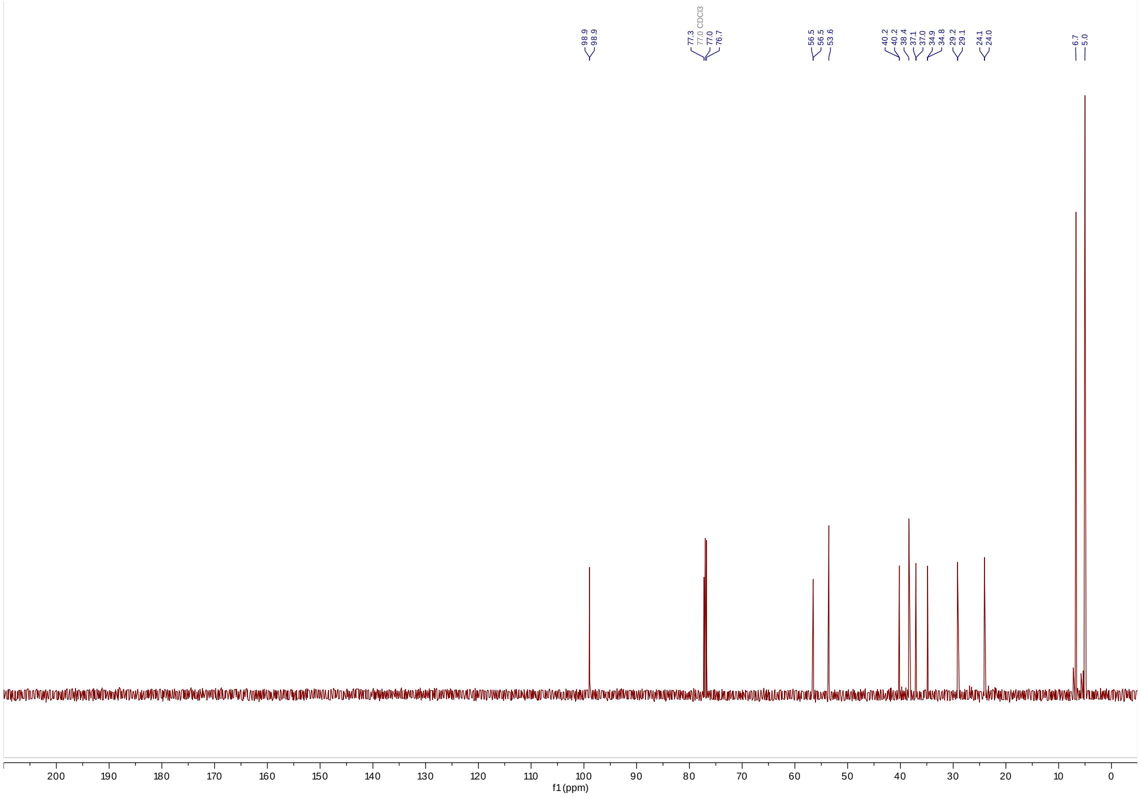

Compound 46: 3-(2-methoxy-2-((triethylsilyl)oxy)ethyl)-1*H*-indole (See compound data)

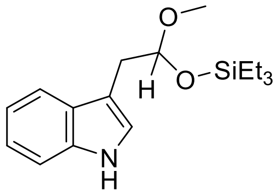

46

^{1}H-NMR (500 MHz, $CDCl_3$) spectra of **46**

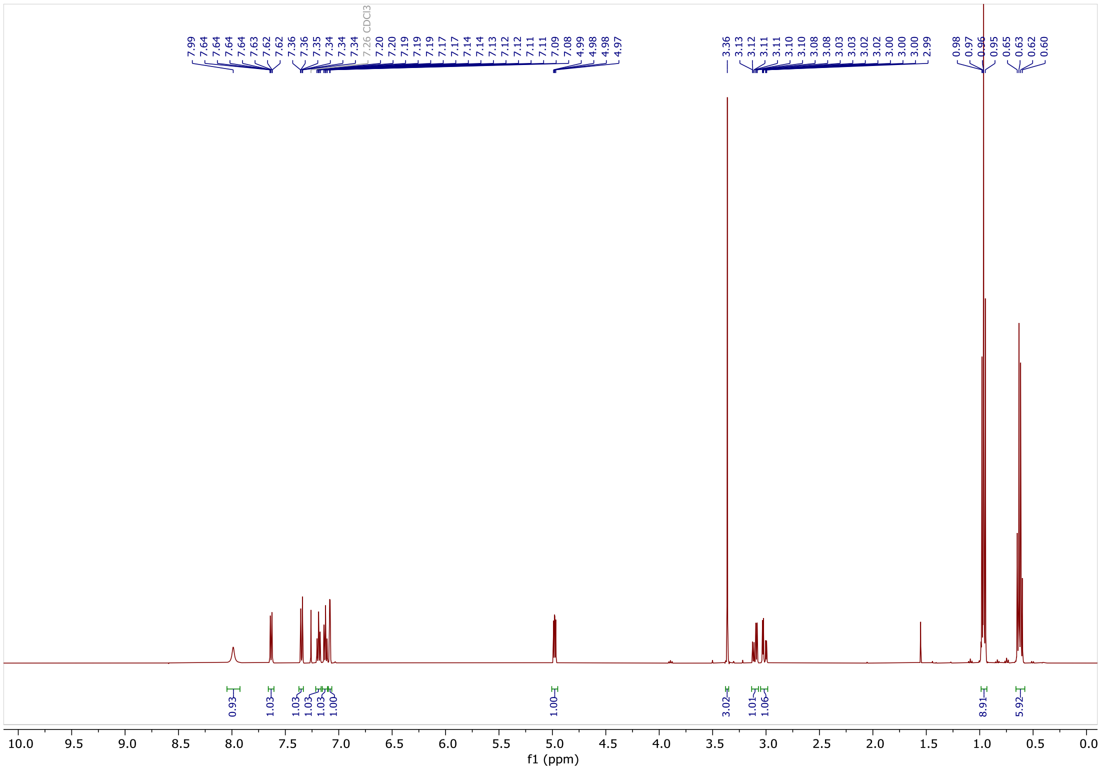

^{13}C-NMR (126 MHz, $CDCl_3$) spectra of **46**

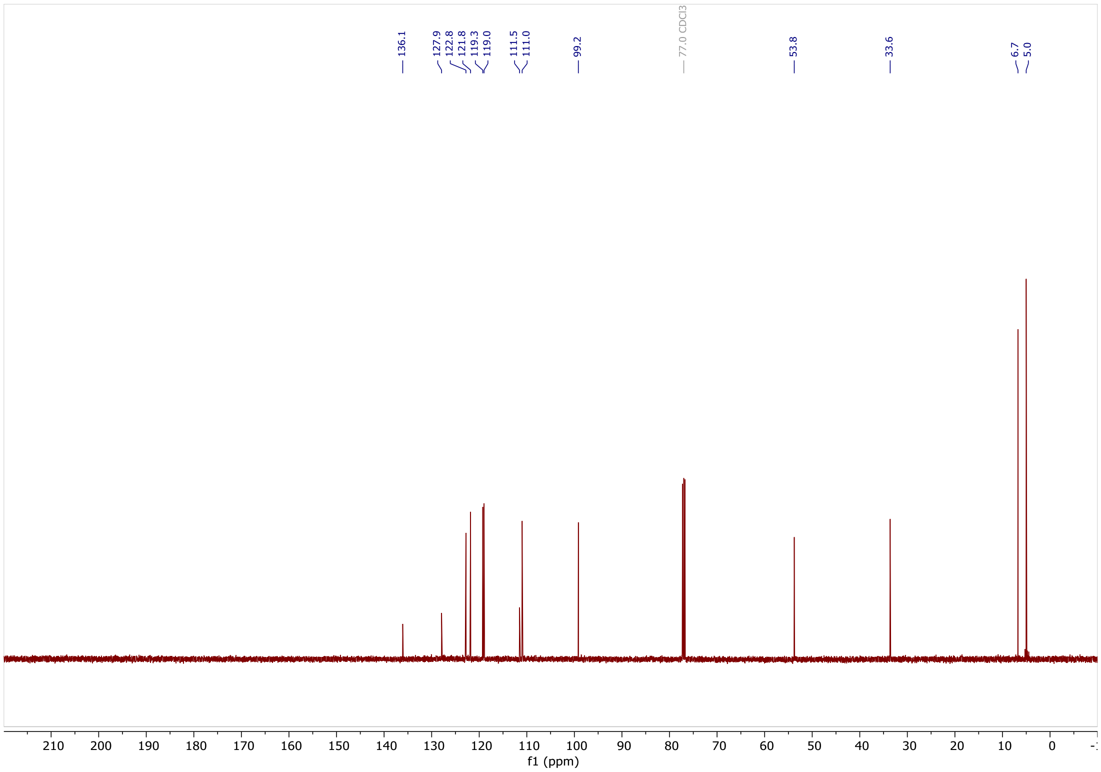

Compound 47: 2-(ethoxy((triethylsilyl)oxy)methyl)benzo[*d*]thiazole (See compound data)

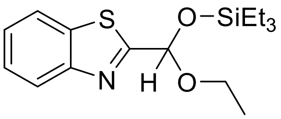

47

^{1}H-NMR (500 MHz, $CDCl_3$) spectra of **47**

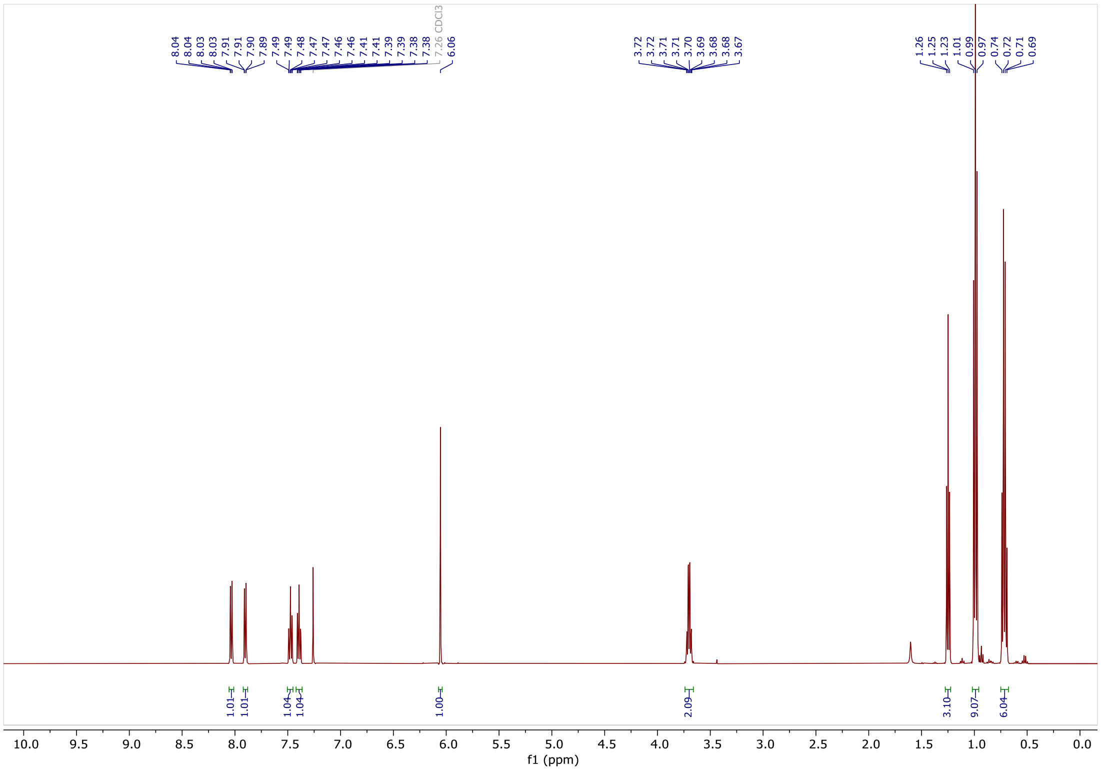

^{13}**C-NMR** (126 MHz, $CDCl_3$) spectra of **47**

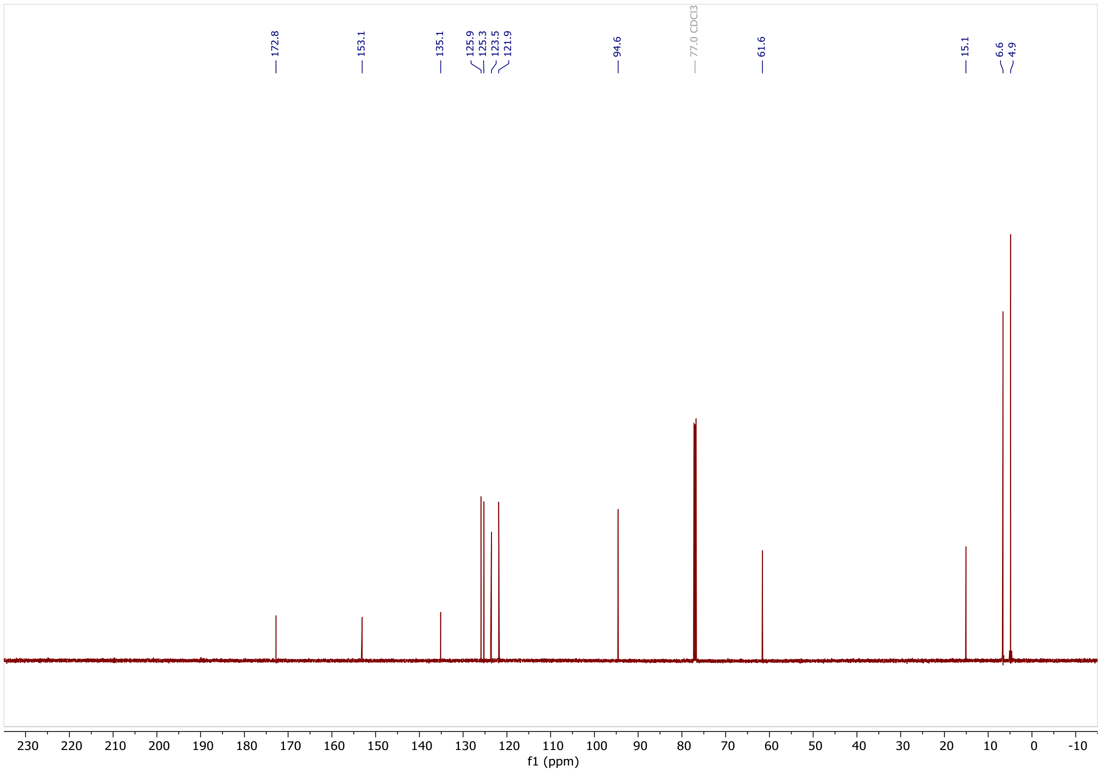

Compound 48: 3-bromo-5-(ethoxy((triethylsilyl)oxy)methyl)isoxazole (See compound data)

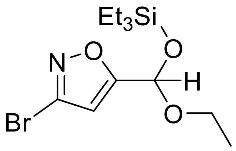

48

^{1}H-NMR (500 MHz, $CDCl_3$) spectra of **48**

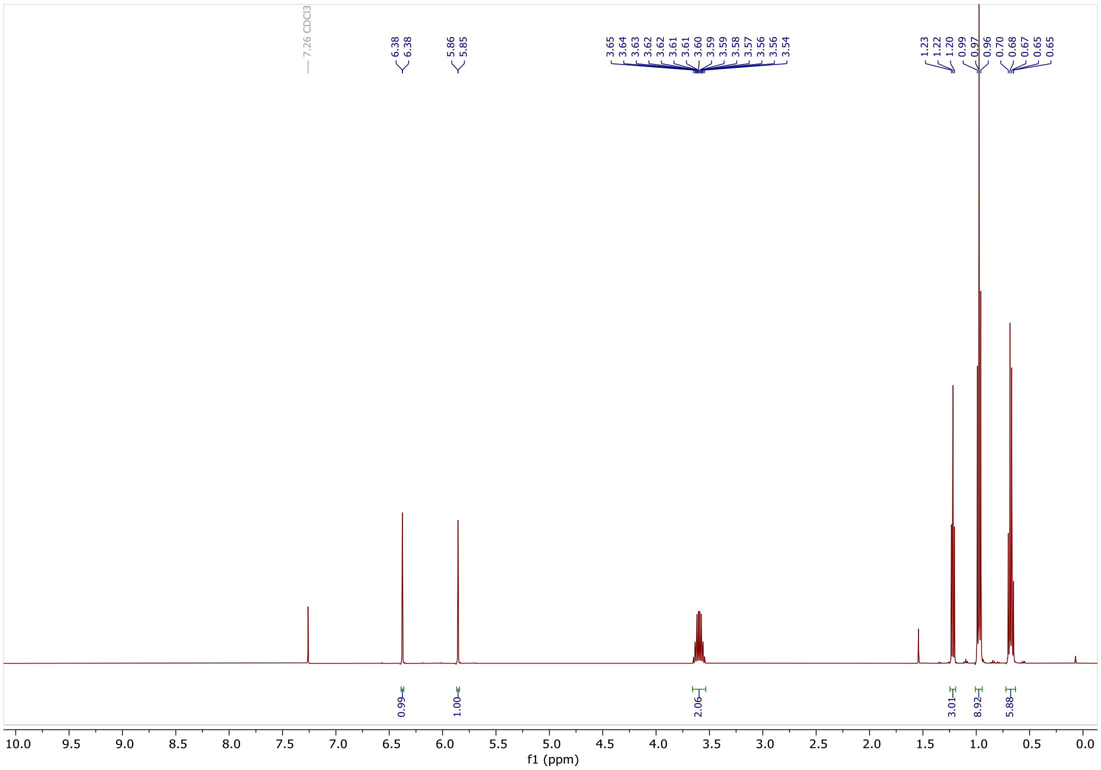

^{13}C-NMR (126 MHz, $CDCl_3$) spectra of **48**

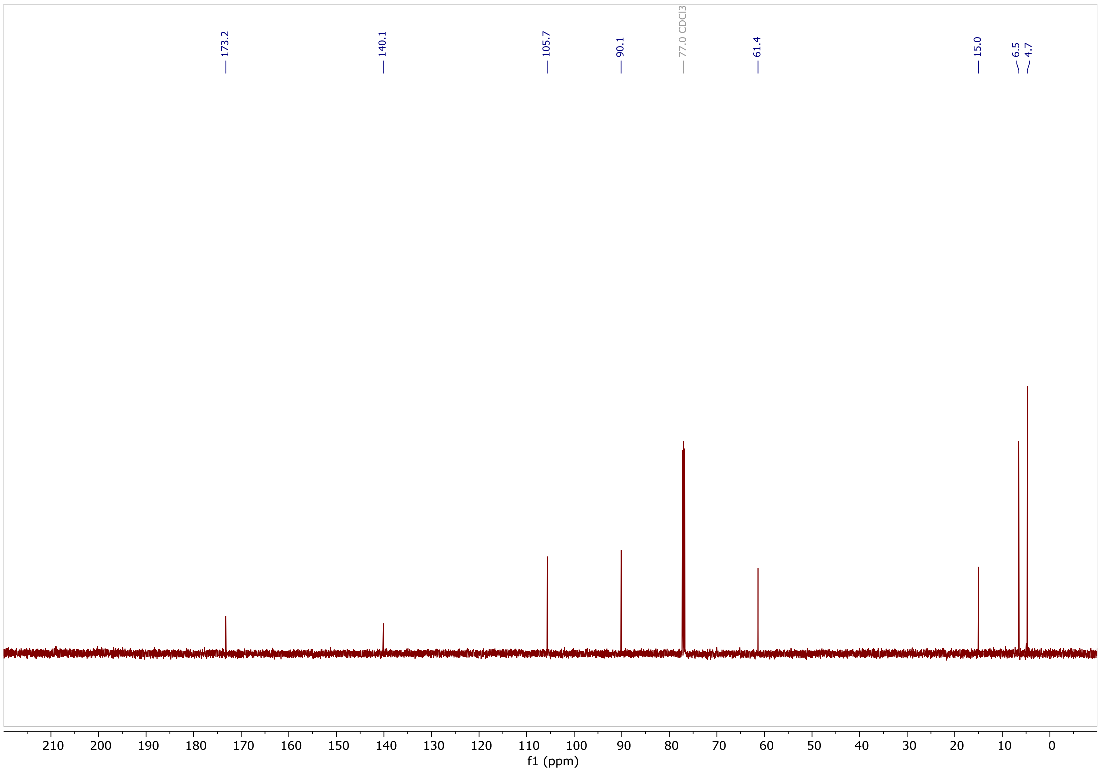

Compound 49: 2-(ethoxy((triethylsilyl)oxy)methyl)pyridine (See compound data)

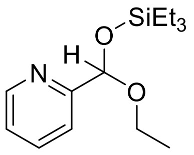

49

^{1}H-NMR (500 MHz, $CDCl_3$) spectra of **49**

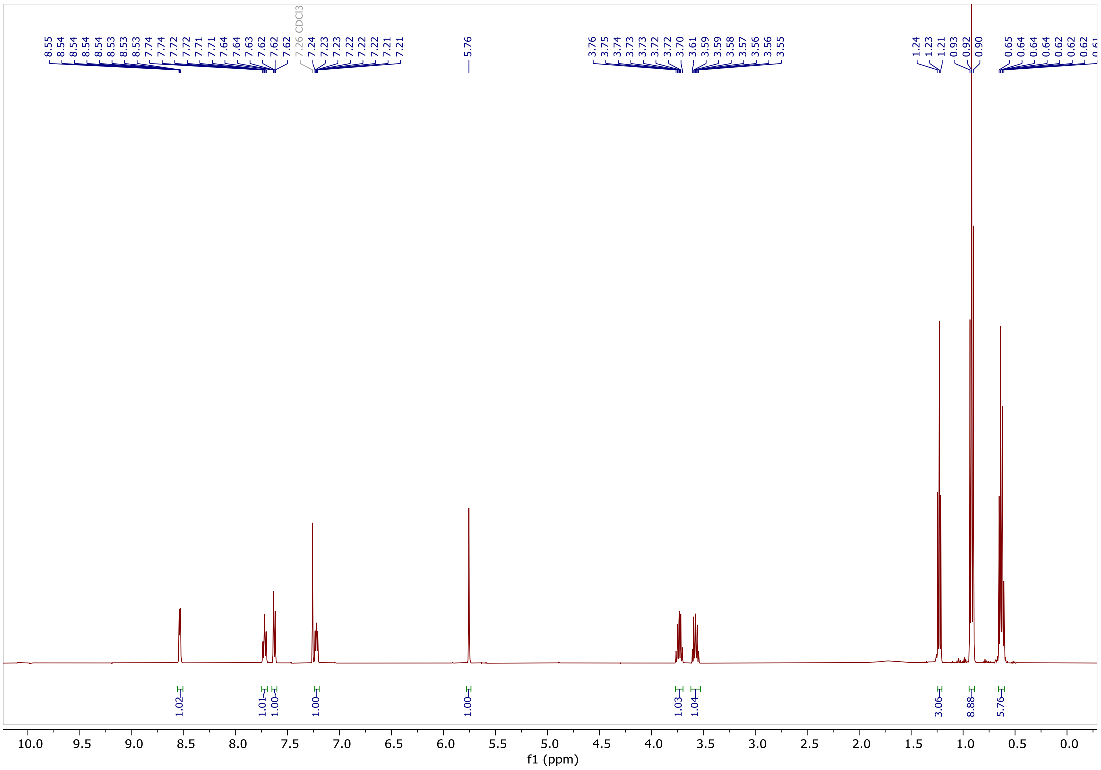

^{13}**C-NMR** (126 MHz, $CDCl_3$) spectra of **49**

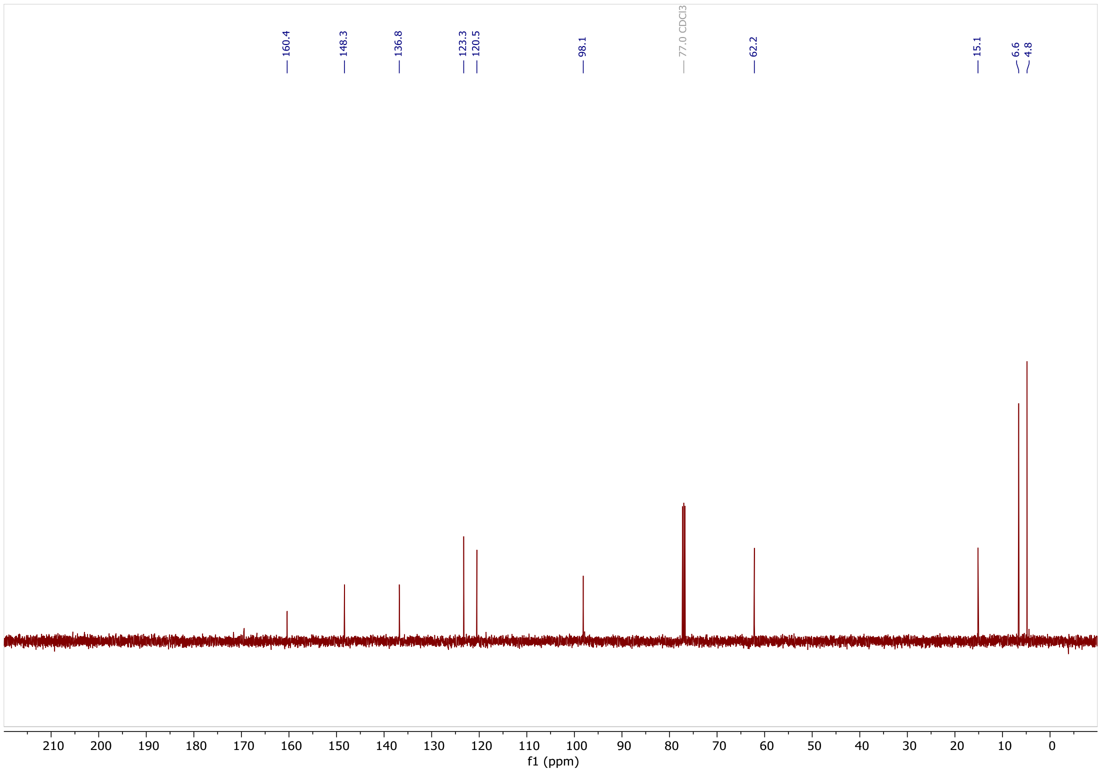

Compound 50: 6-bromo-4-(ethoxy((triethylsilyl)oxy)methyl)-2-methylquinoline (See compound data)

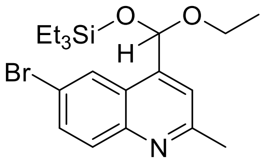

50

^{1}H-NMR (500 MHz, $CDCl_3$) spectra of **50**

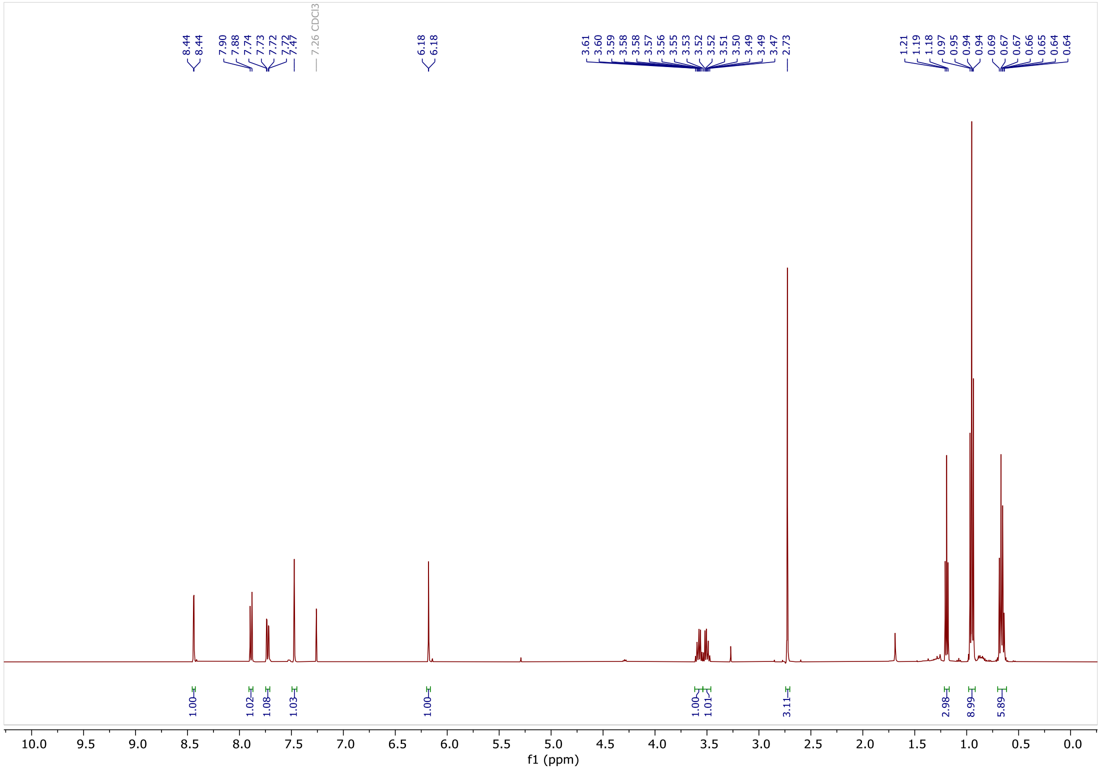

^{13}**C-NMR** (126 MHz, $CDCl_3$) spectra of **50**

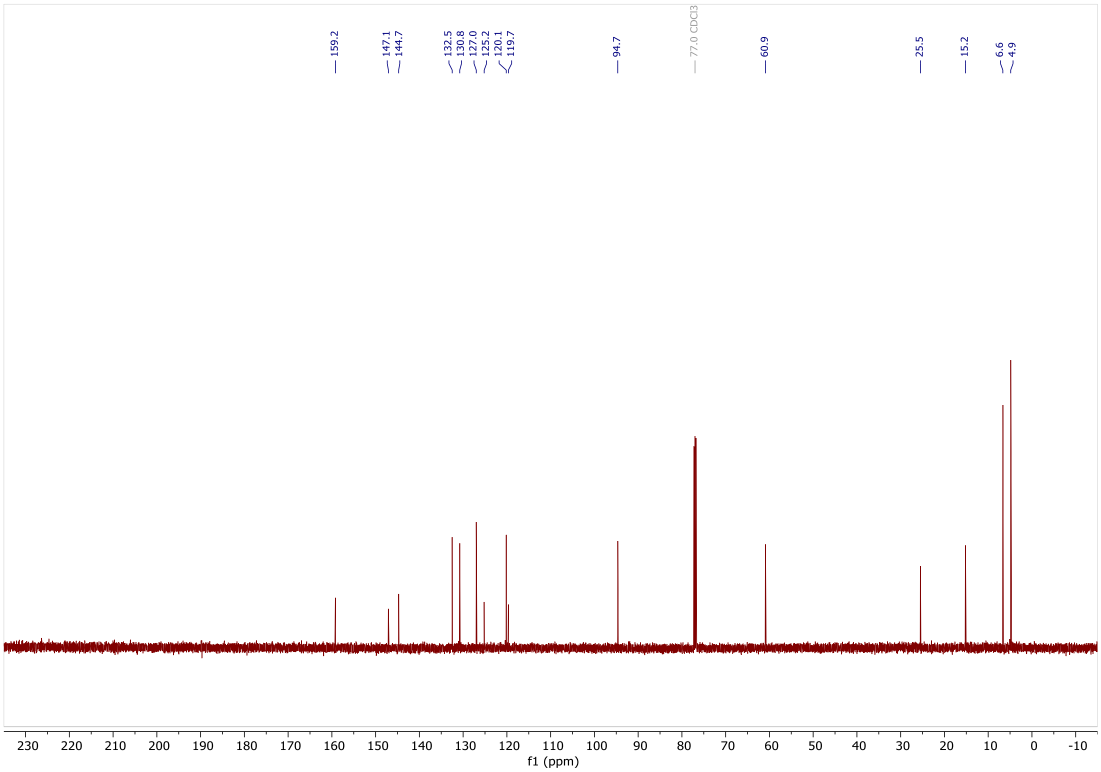

Compound 51: 1-benzhydryl-3-(methoxy((triethylsilyl)oxy)methyl)azetidine (See compound data)

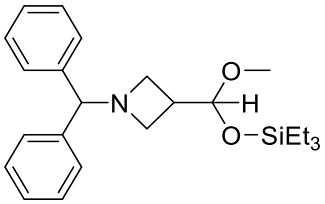

51

^{1}H-NMR (500 MHz, $CDCl_3$) spectra of **51**

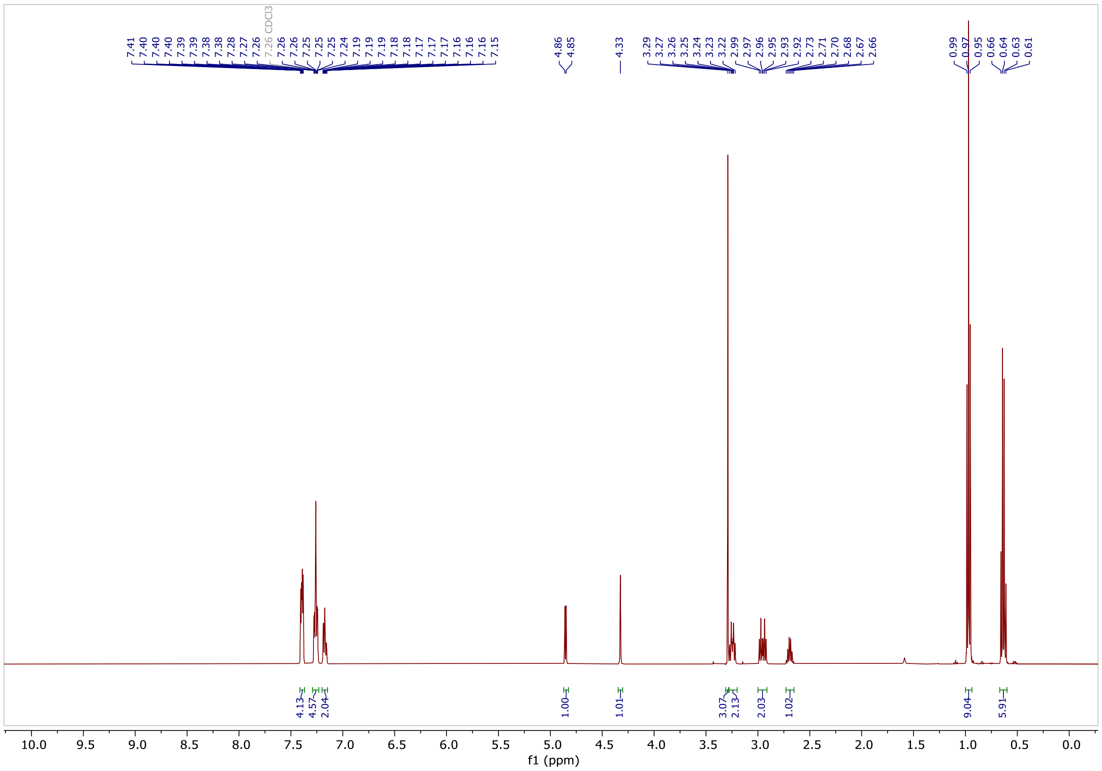

^{13}C-NMR (126 MHz, $CDCl_3$) spectra of **51**

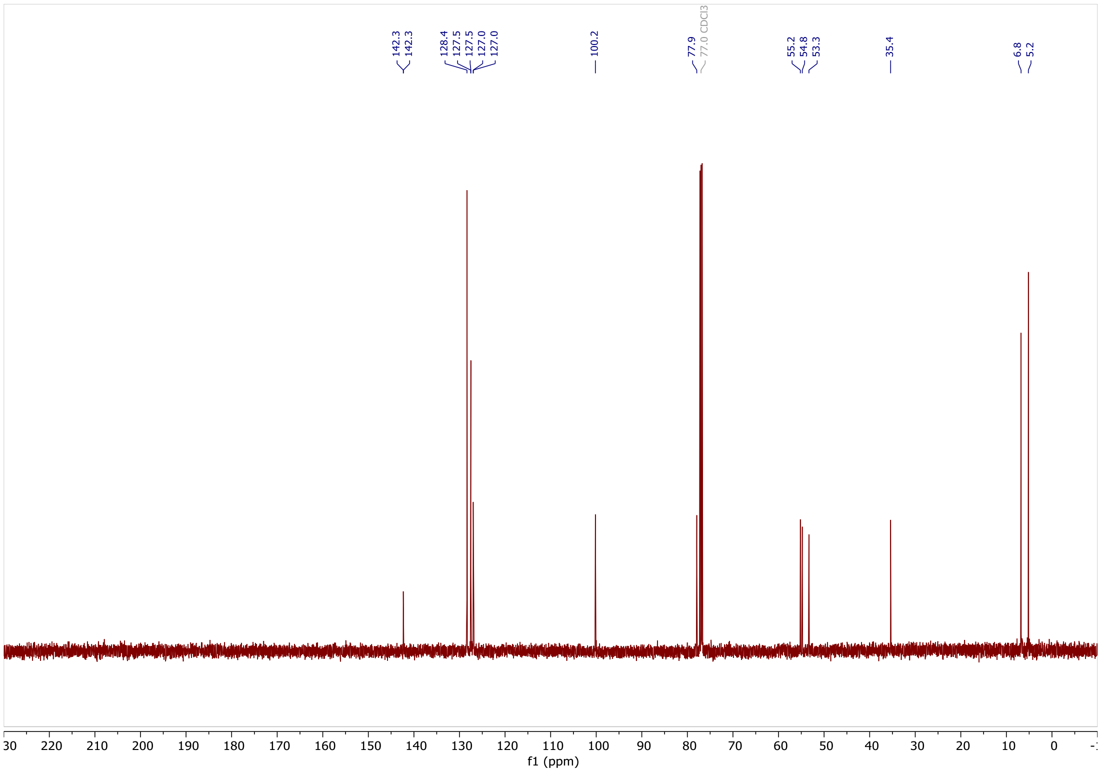

Compound 52: triethylsilyl (3-methoxy-3-((triethylsilyl)oxy)propyl)carbamate (See compound data)

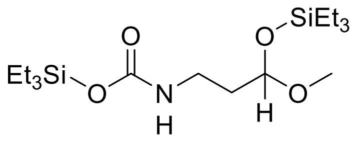

52

^{1}H-NMR (500 MHz, C_6D_6) spectra of **52**

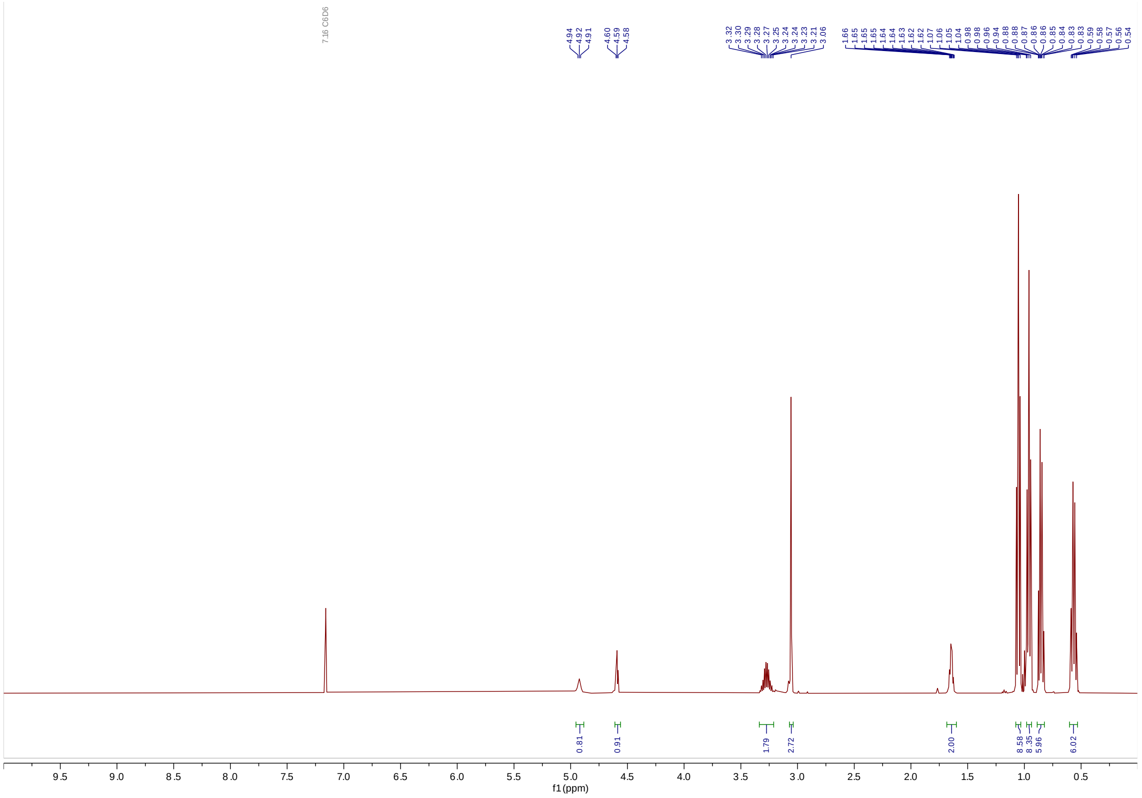

^{13}C-NMR (126 MHz, C_6D_6) spectra of **52**

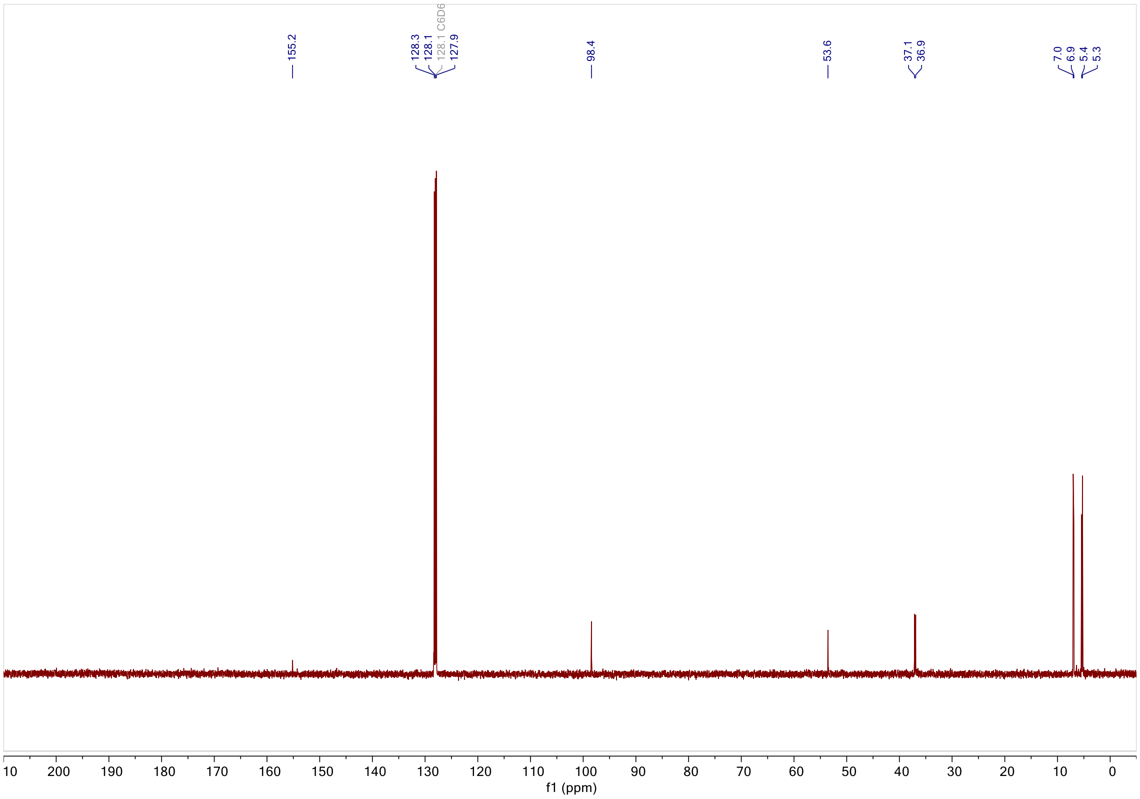

Compound 53: 2,4,6-trichloro-*N*-(4-ethoxy-4-((triethylsilyl)oxy)butyl)benzamide (See compound data)

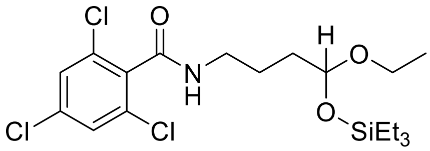

53

[1]**H-NMR** (500 MHz, C_6D_6) spectra of **53**

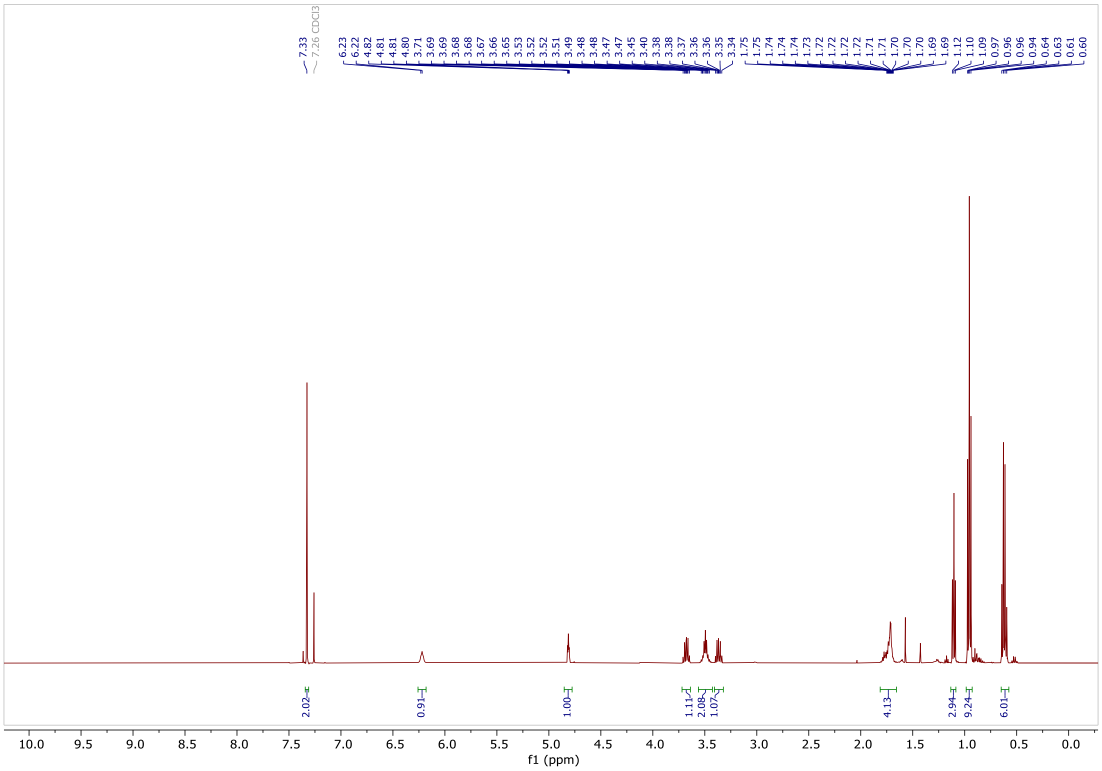

^{13}**C-NMR** (126 MHz, C_6D_6) spectra of **53**

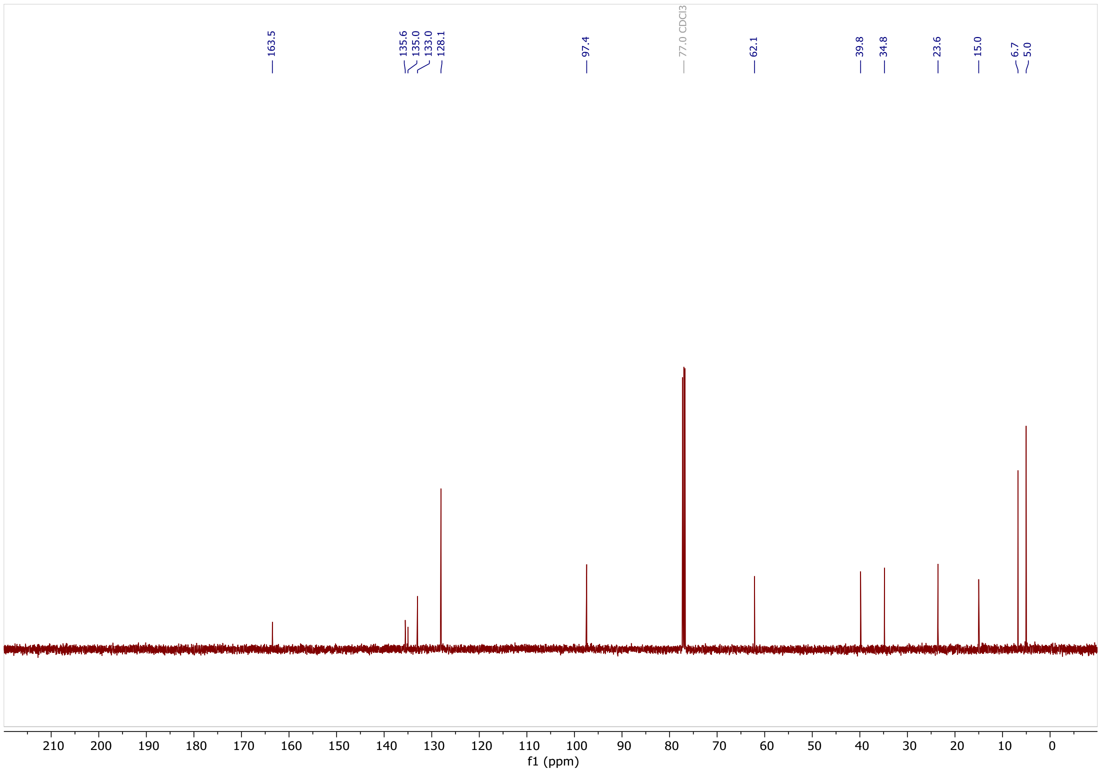

Compound 54: 2-(4-ethoxy-4-((triethylsilyl)oxy)butyl)isoindoline-1,3-dione (See compound data)

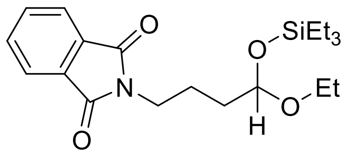

54

^{1}H-NMR (300 MHz, $CDCl_3$) spectra of **54**

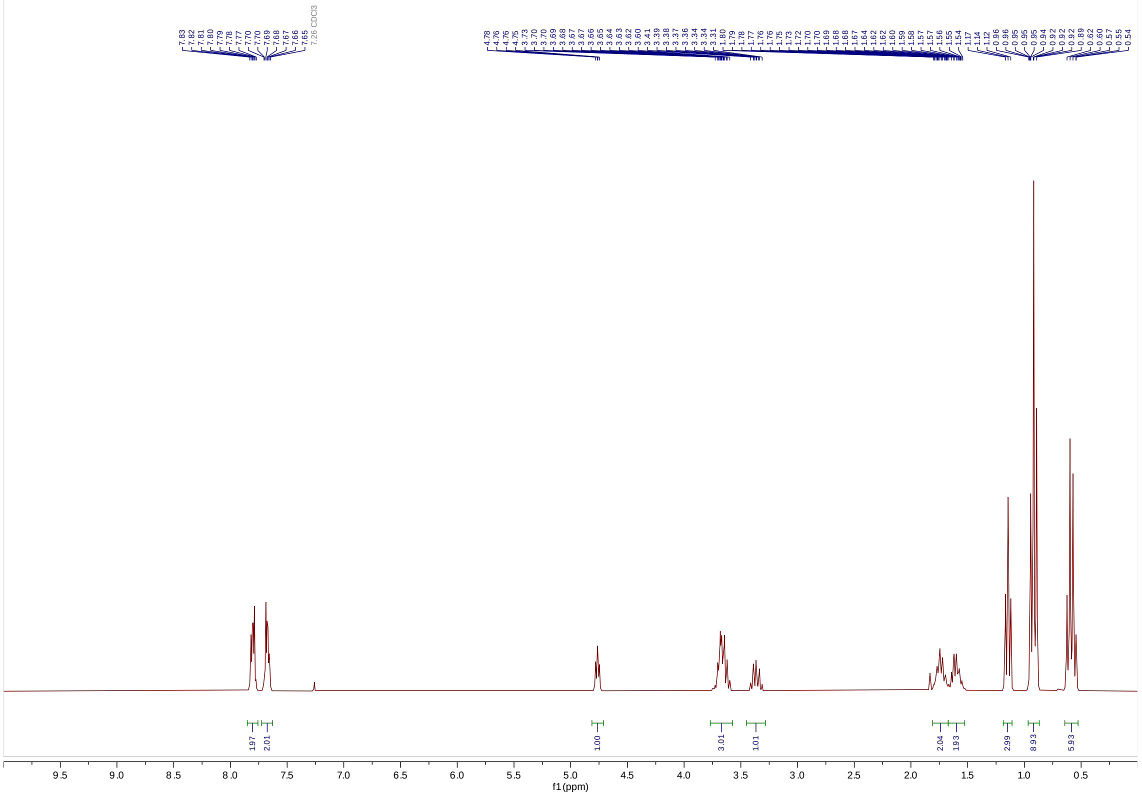

^{13}**C-NMR** (75 MHz, $CDCl_3$) spectra of **54**

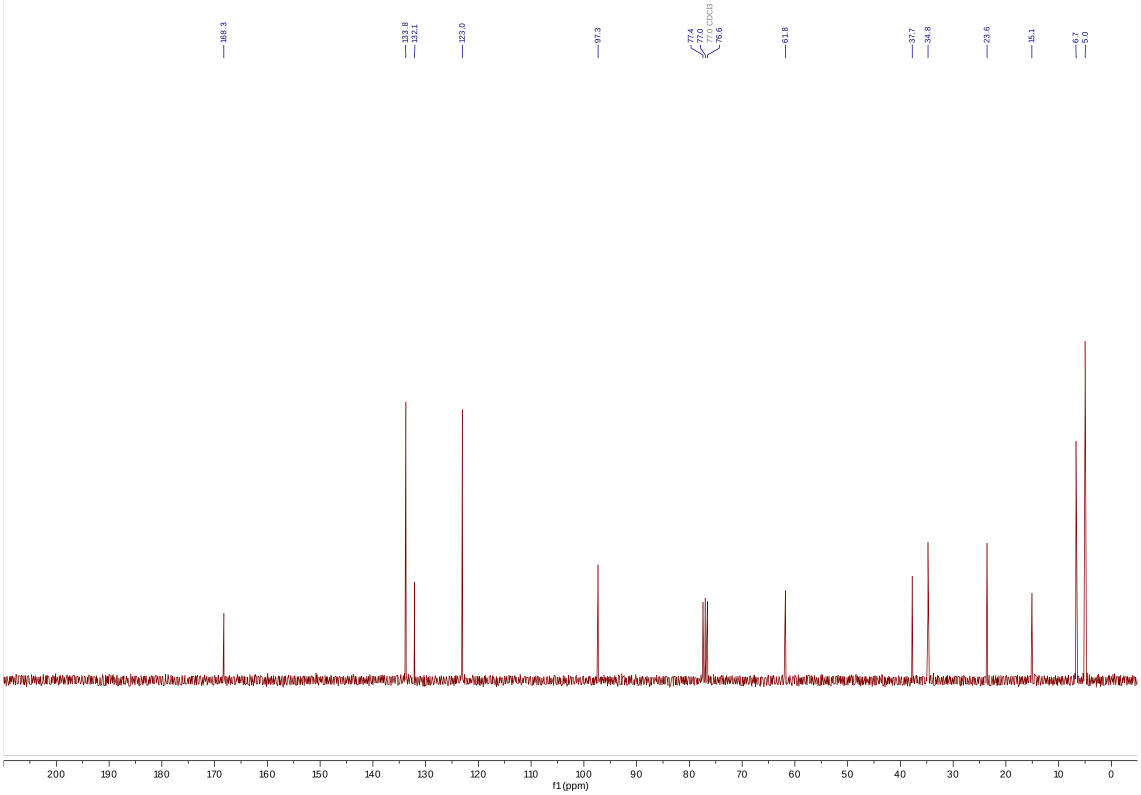

Compound 55: 4,5,6,7-tetrachloro-2-(4-ethoxy-4-((triethylsilyl)oxy)butyl)isoindoline-1,3-dione (See compound data)

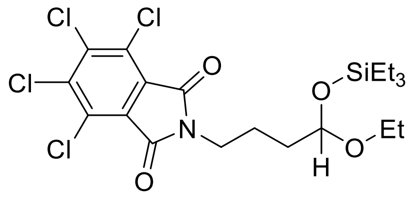

55

^{1}H-NMR (500 MHz, $CDCl_3$) spectra of **55**

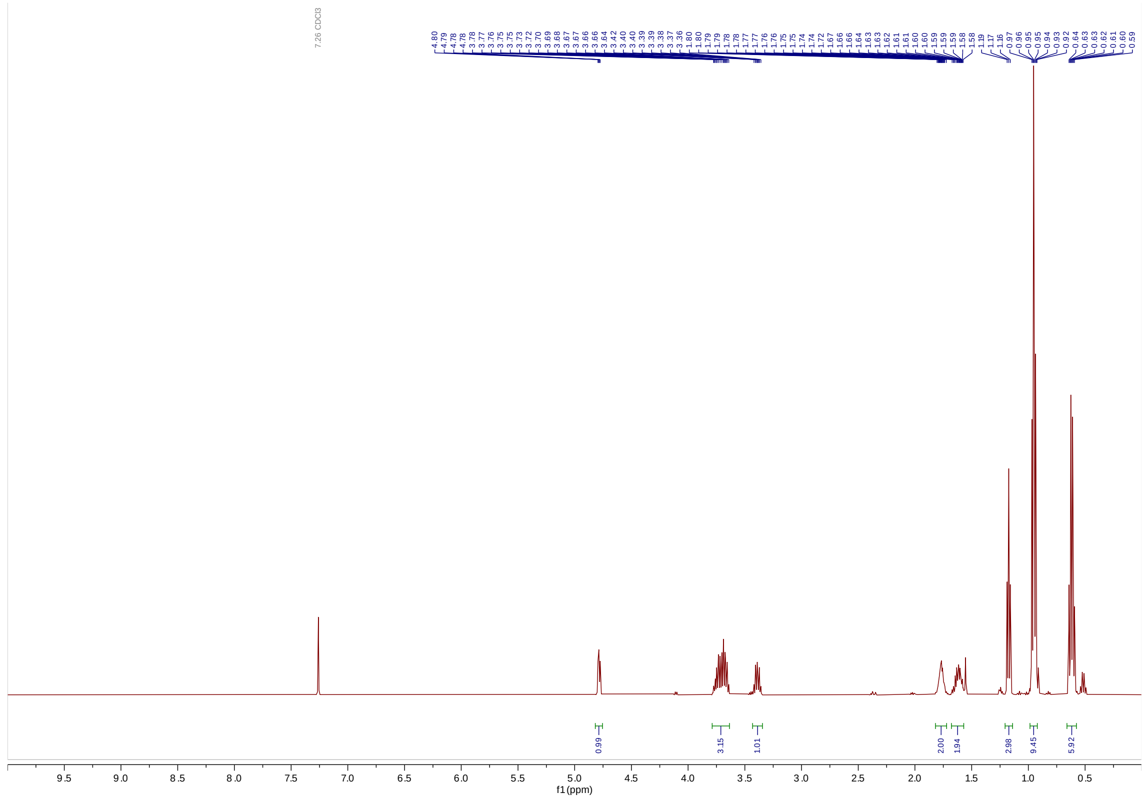

^{13}**C-NMR** (126 MHz, $CDCl_3$) spectra of **55**

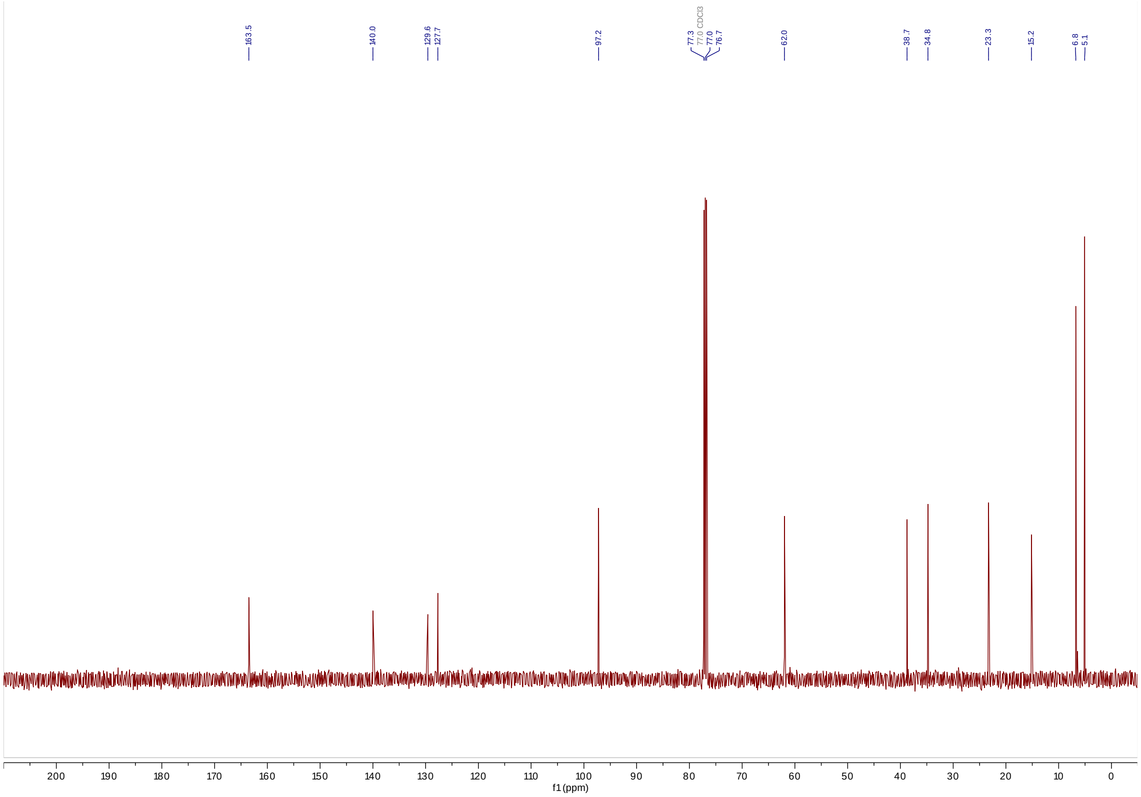

Compound 56: *N*,*N*-diisopropyl-6-methoxy-6-((triethylsilyl)oxy)hexanamide (See compound data)

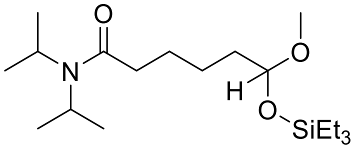

56

^{1}H-NMR (500 MHz, $CDCl_3$) spectra of **56**

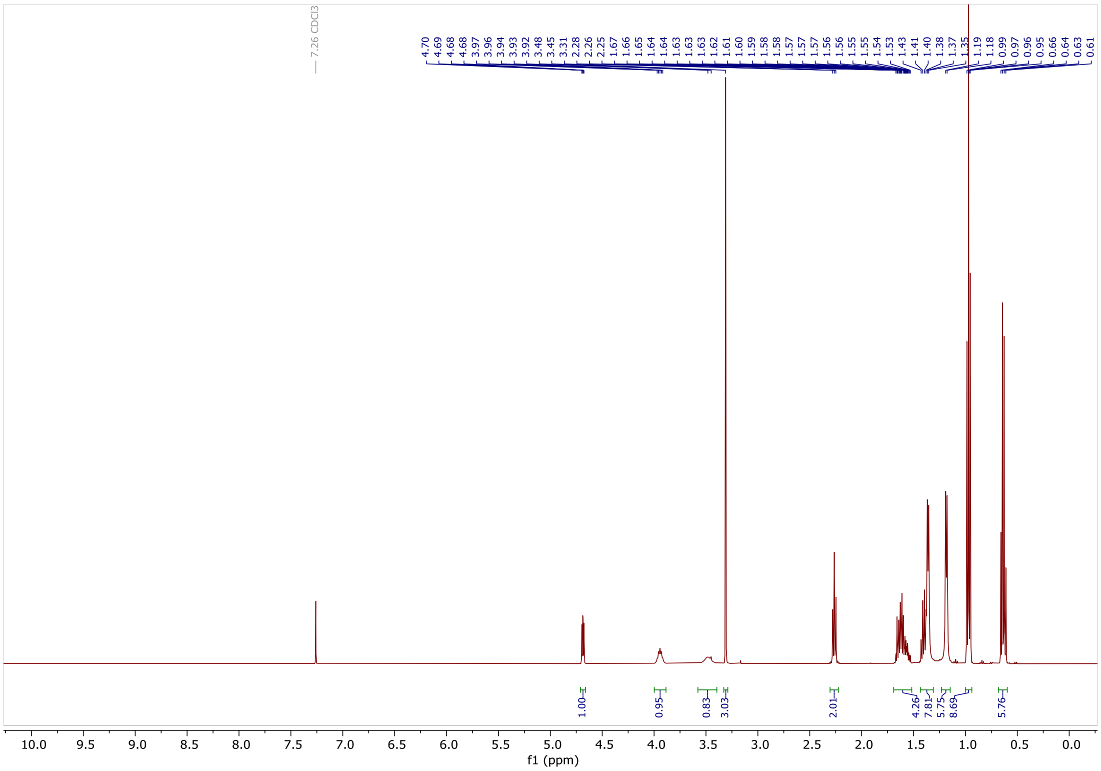

^{13}C-NMR (126 MHz, $CDCl_3$) spectra of **56**

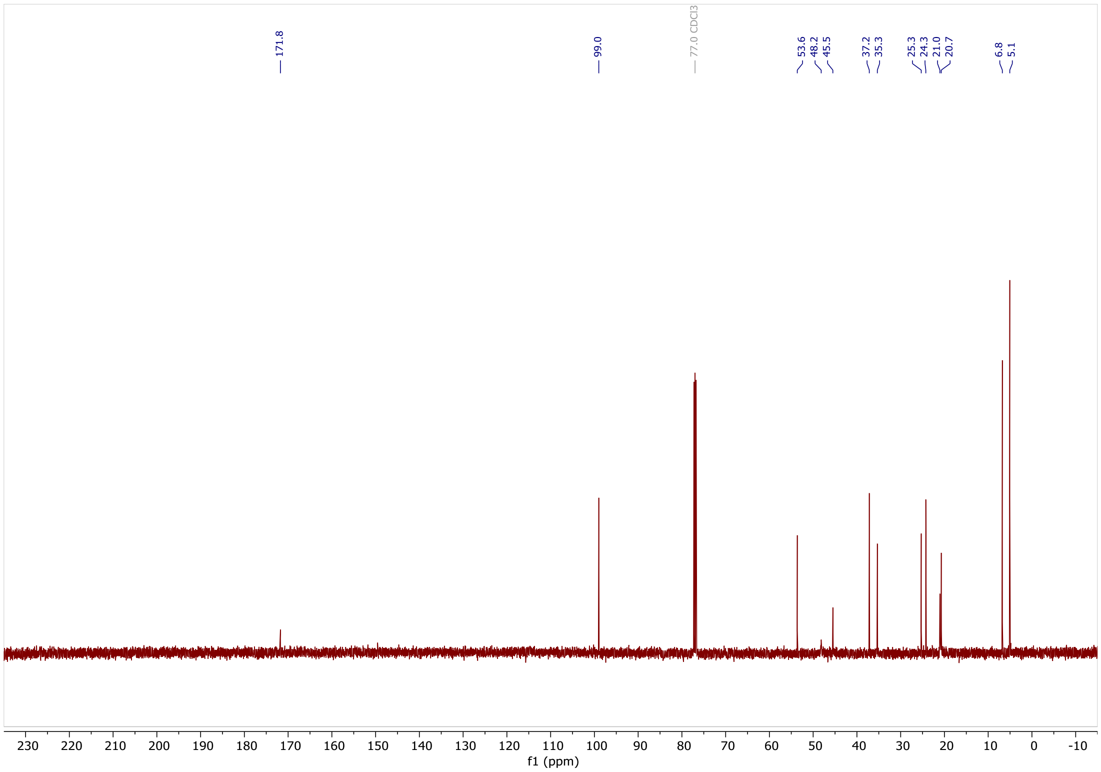

Compound 57: (ethoxy(4-nitrophenyl)methoxy)triethylsilane (See compound data)

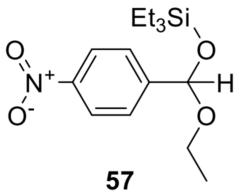

57

^{1}H-NMR (500 MHz, $CDCl_3$) spectra of **57**

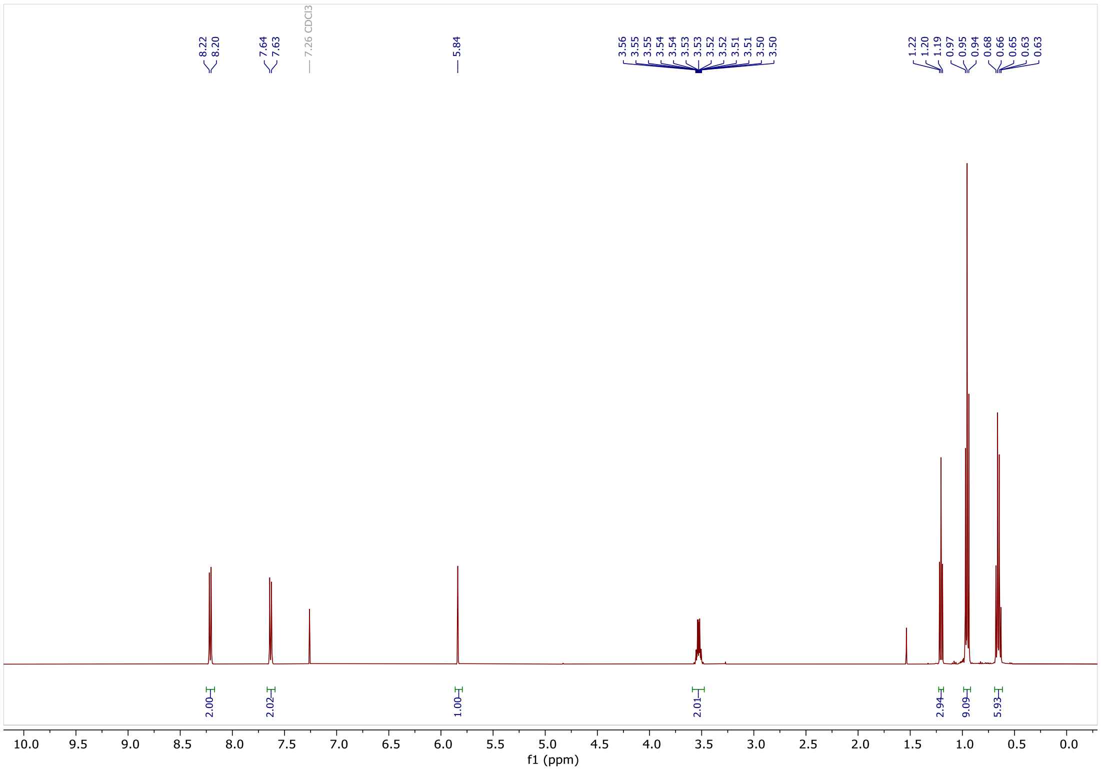

^{13}C-NMR (126 MHz, $CDCl_3$) spectra of **57**

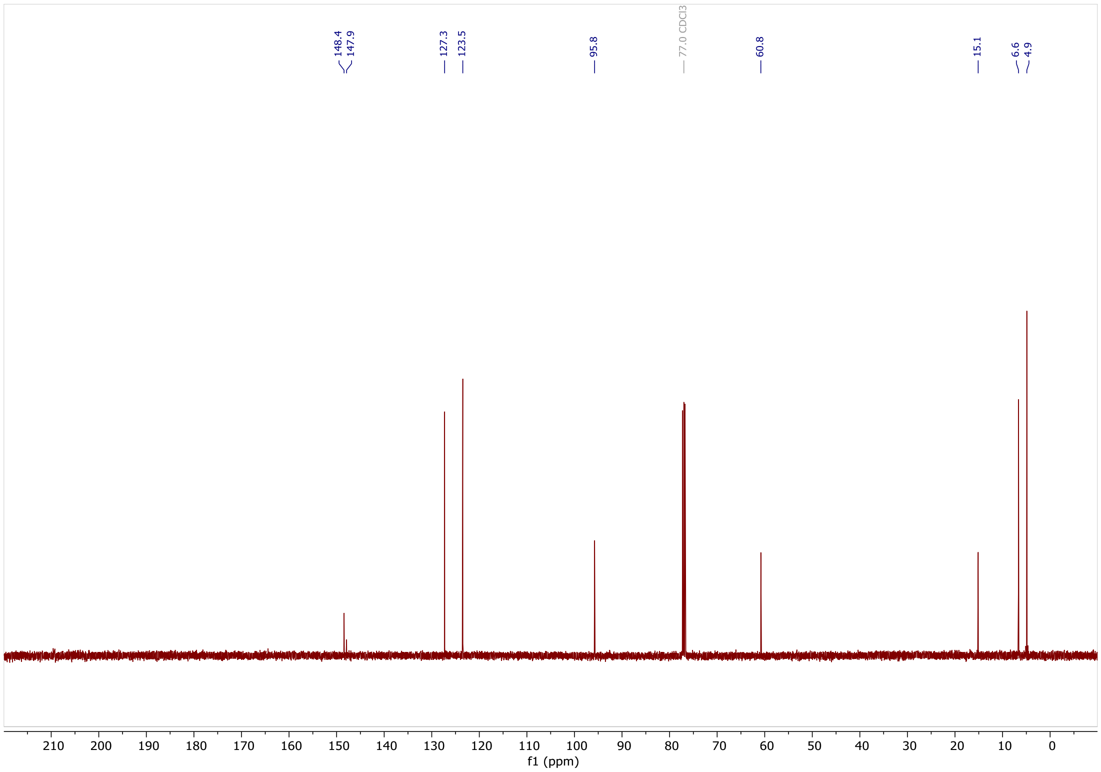

Compound 58: (1-ethoxy-2-(4-nitrophenyl)ethoxy)triethylsilane (See compound data)

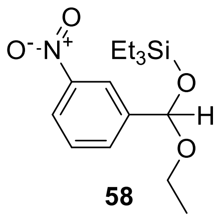

58

^{1}H-NMR (500 MHz, $CDCl_3$) spectra of **58**

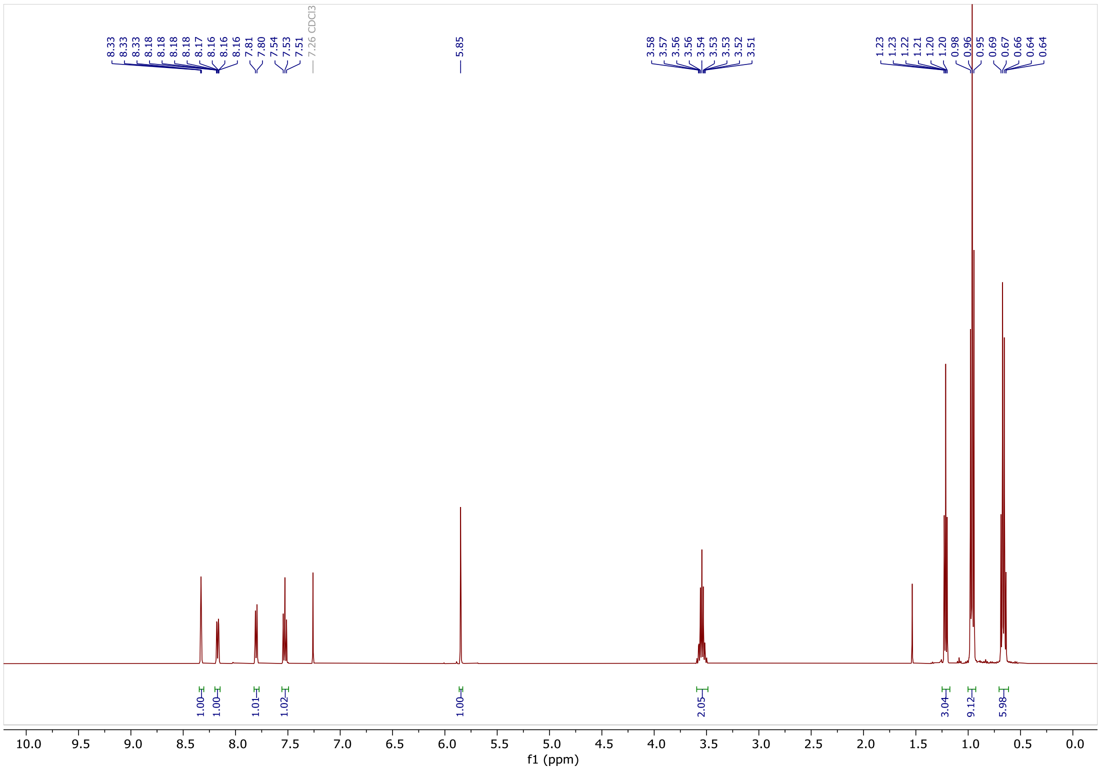

^{13}**C-NMR** (126 MHz, $CDCl_3$) spectra of **58**

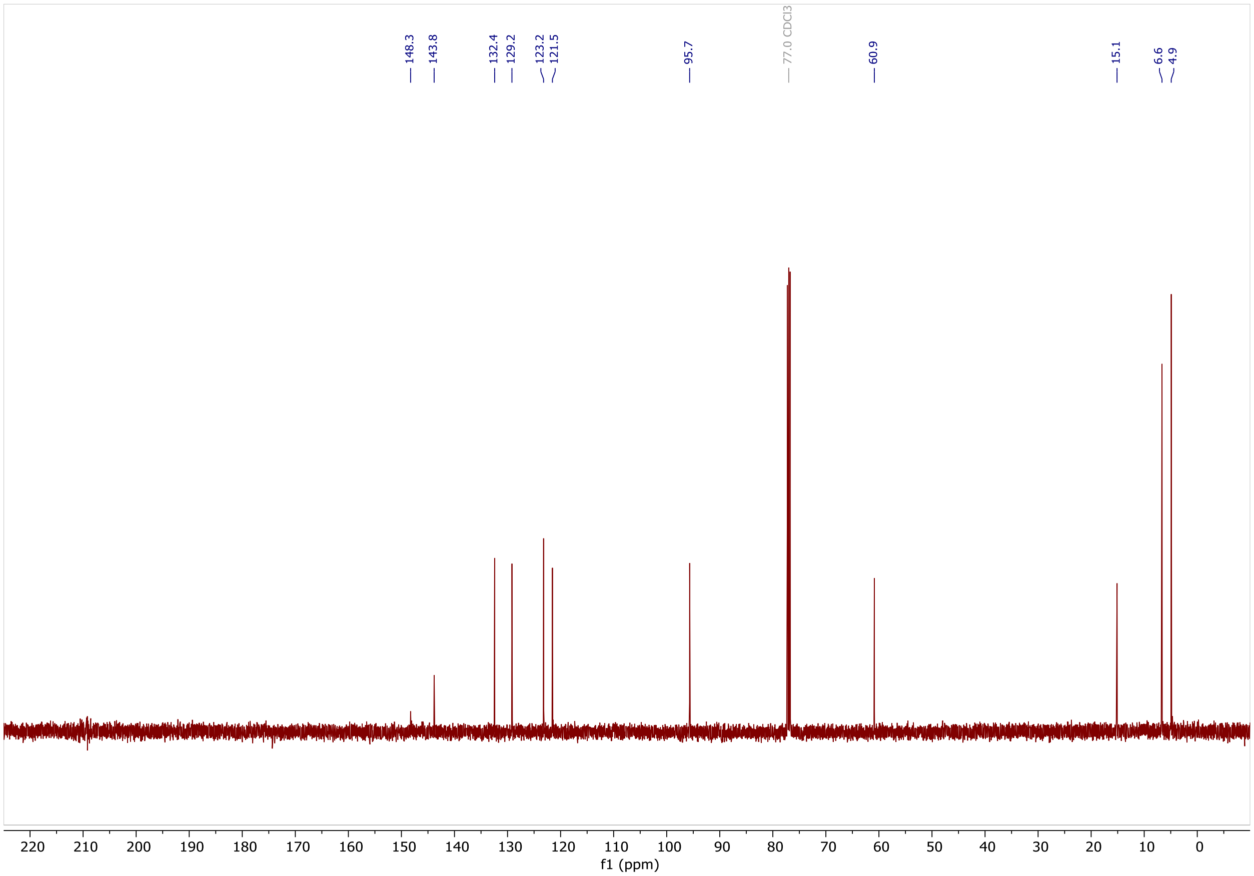

Compound 59: (1-ethoxy-2-(2-nitrophenyl)ethoxy)triethylsilane (See compound data)

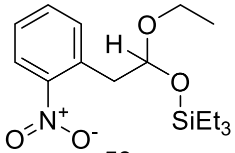

59

^{1}H-NMR (500 MHz, $CDCl_3$) spectra of **59**

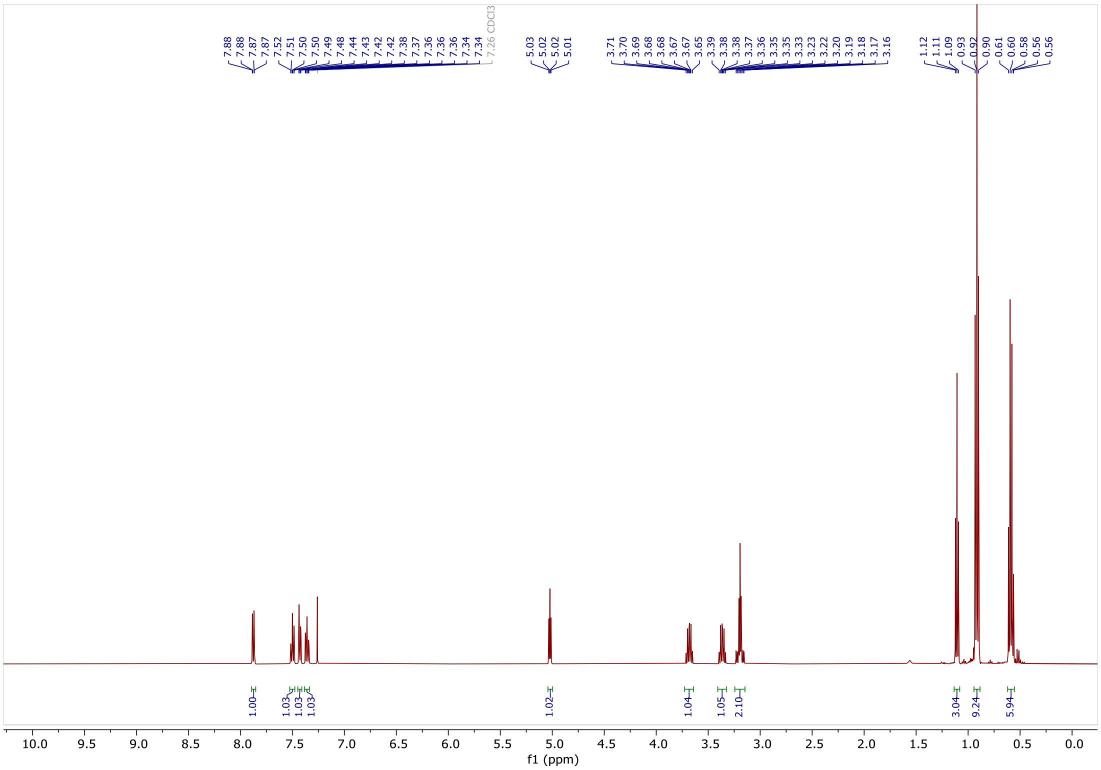

^{13}**C-NMR** (126 MHz, $CDCl_3$) spectra of **59**

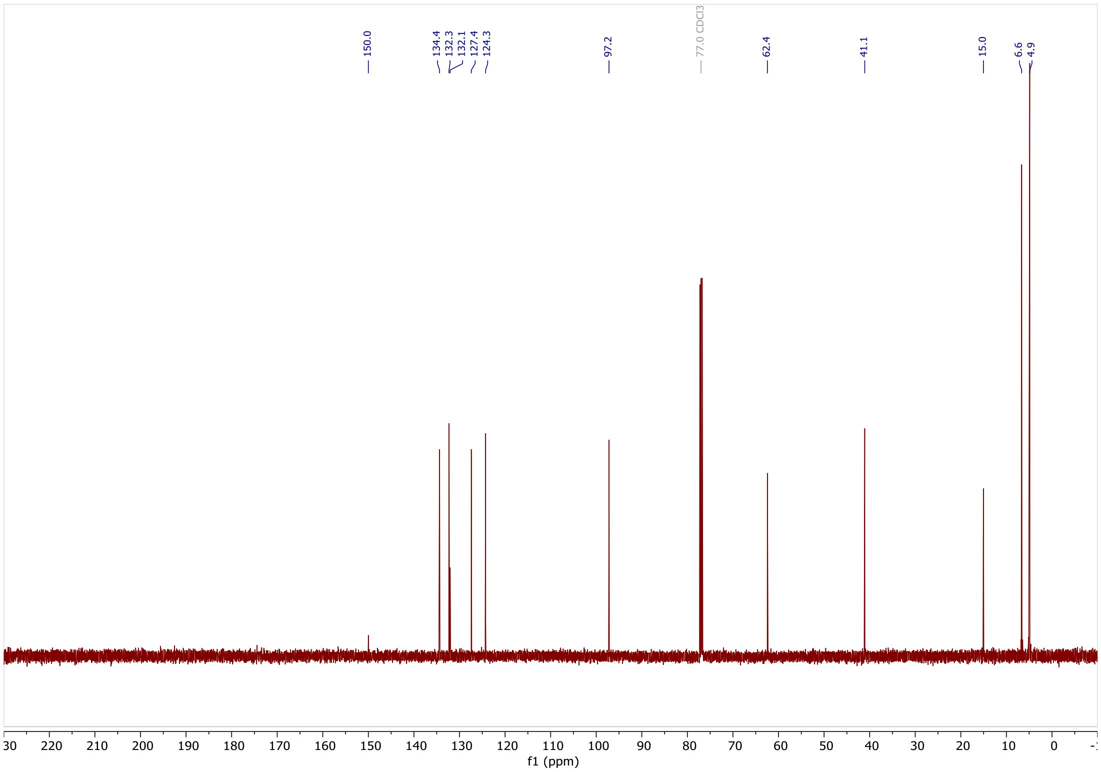

Compound 60: (1-ethoxy-3-nitropropoxy)triethylsilane (See compound data)

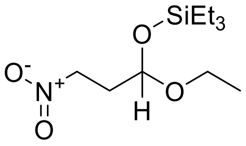

60

^{1}H-NMR (500 MHz, $CDCl_3$) spectra of **60**

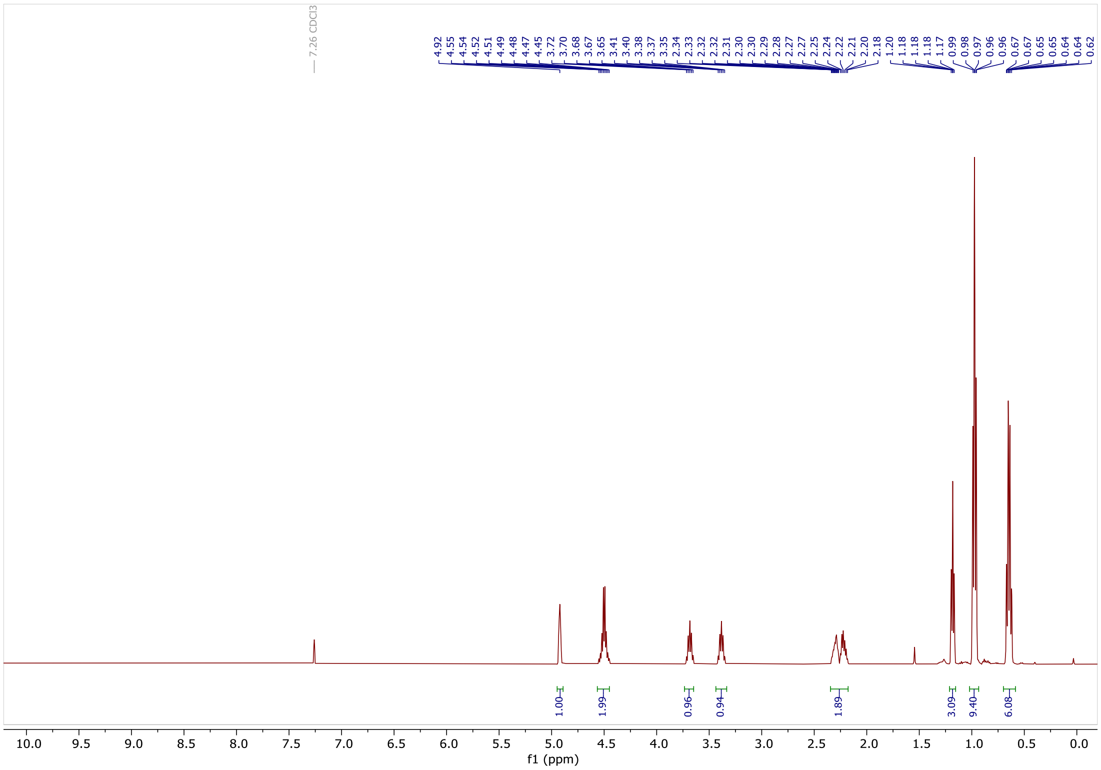

^{13}**C-NMR** (126 MHz, $CDCl_3$) spectra of **60**

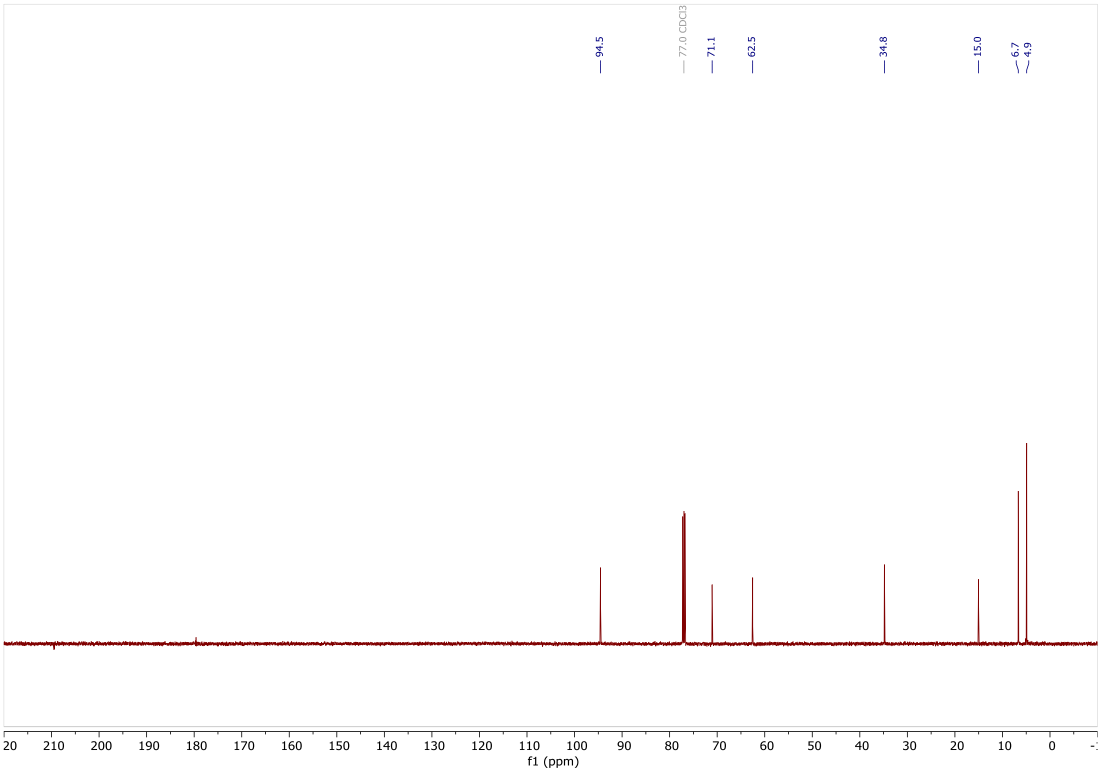

Compound 61: 4,10-bis(3-bromopropyl)-6,6,8,8-tetramethyl-3,5,7,9,11-pentaoxa-6,8-disilatridecane (See compound data)

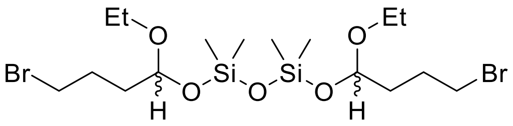

61

^{1}H-NMR (500 MHz, $CDCl_3$) spectra of **61**

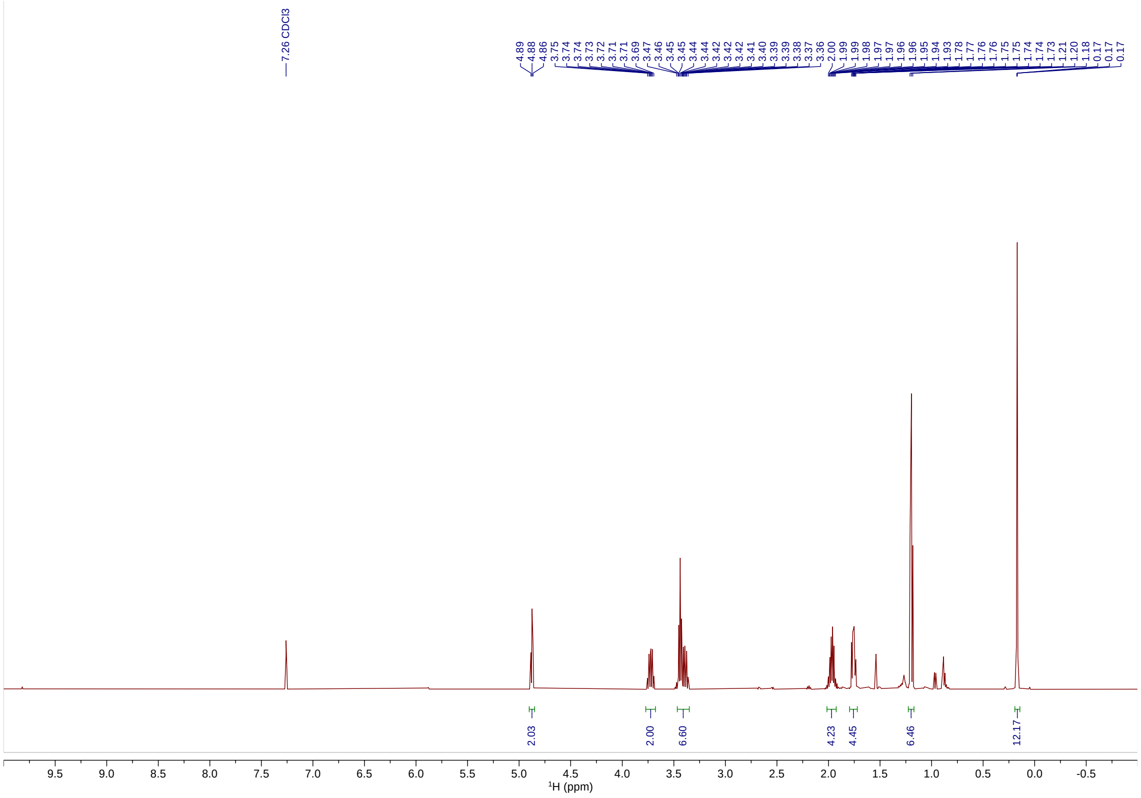

^{13}C-NMR (126 MHz, $CDCl_3$) spectra of **61**

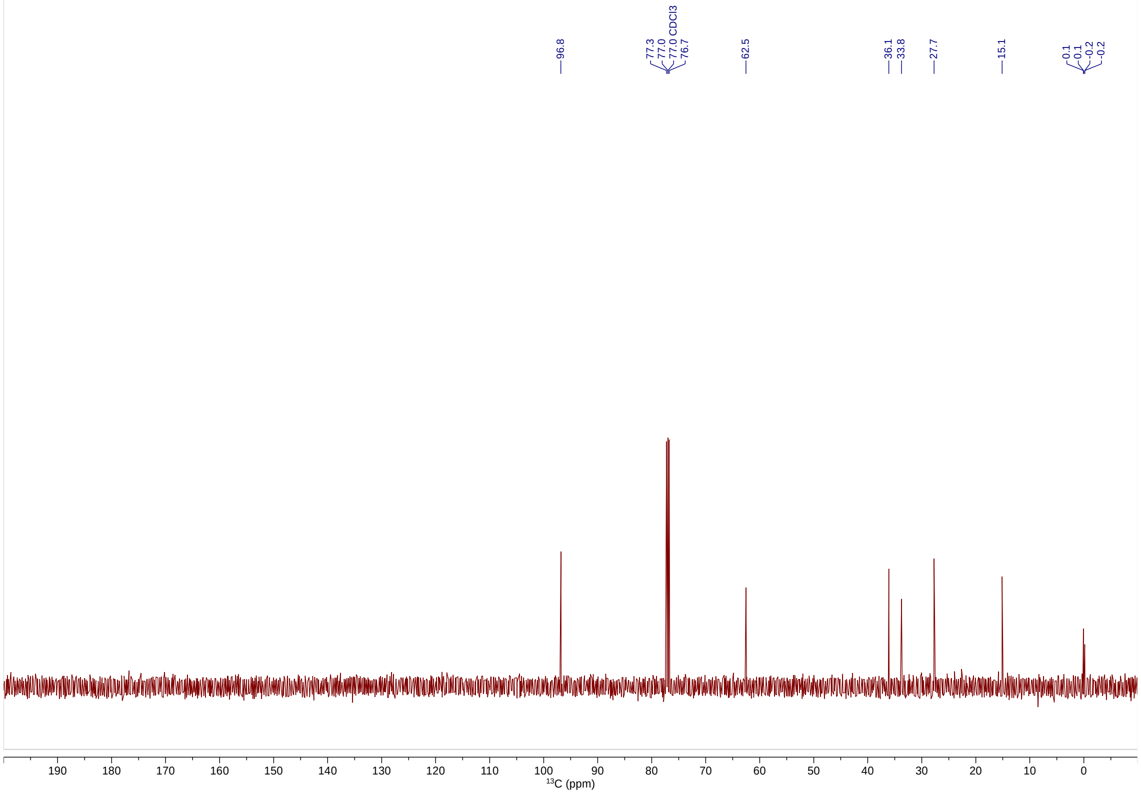

Compound 63: (((3*S*,3a*S*,5a*S*,9b*S*)-3,5a,9-trimethyl-2,3,3a,4,5,5a,6,9b-octahydronaphtho[1,2-*b*]furan-2,8-diyl)bis(oxy))bis(triethylsilane) (See compound data)

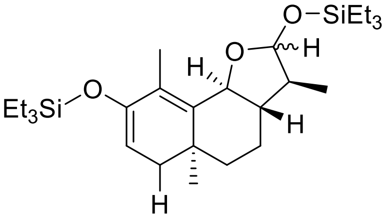

63

^{1}H-NMR (500 MHz, $CDCl_3$) spectra of **63**

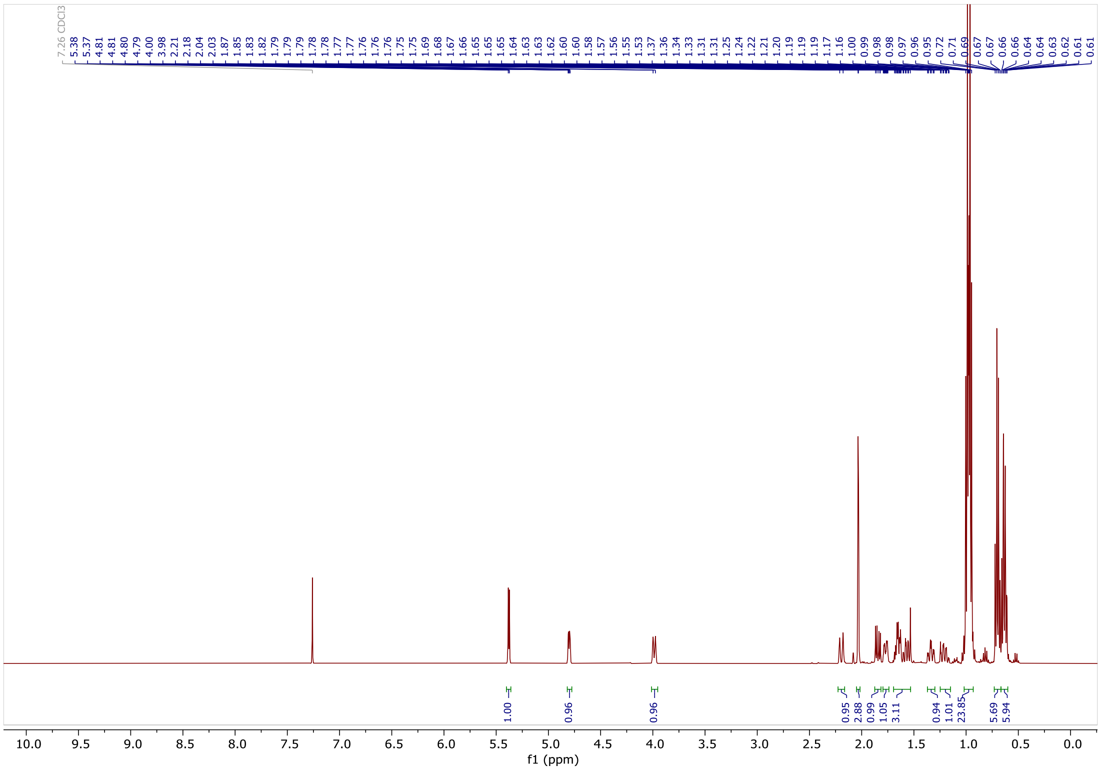

^{13}C-NMR (126 MHz, $CDCl_3$) spectra of 63

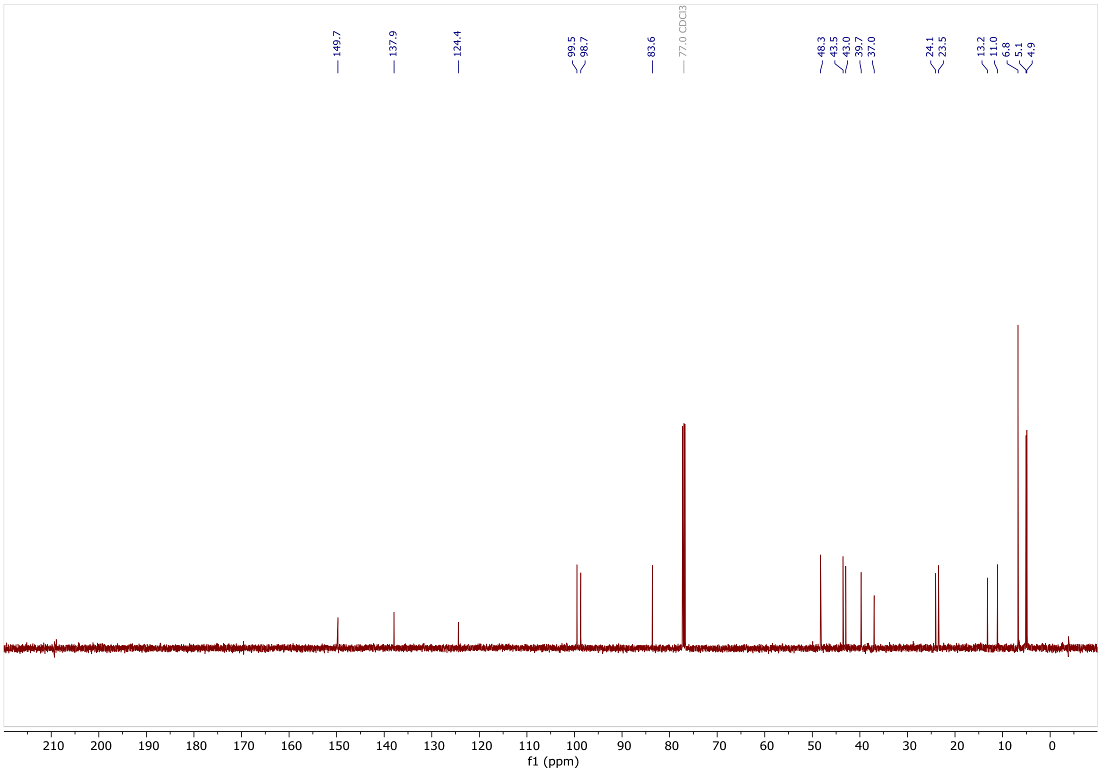

Compound 64: (3*S*,3a*S*,5a*S*,9b*S*)-3,5a,9-trimethyl-2-((triethylsilyl)oxy)-2,3,3a,5,5a,6,7,9b-octahydronaphtho[1,2-*b*]furan-8(4*H*)-one (See compound data)

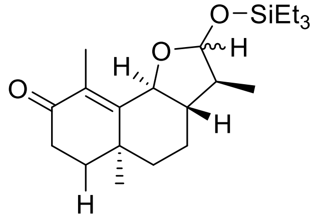

64

¹H-NMR (500 MHz, $CDCl_3$) spectra of **64**

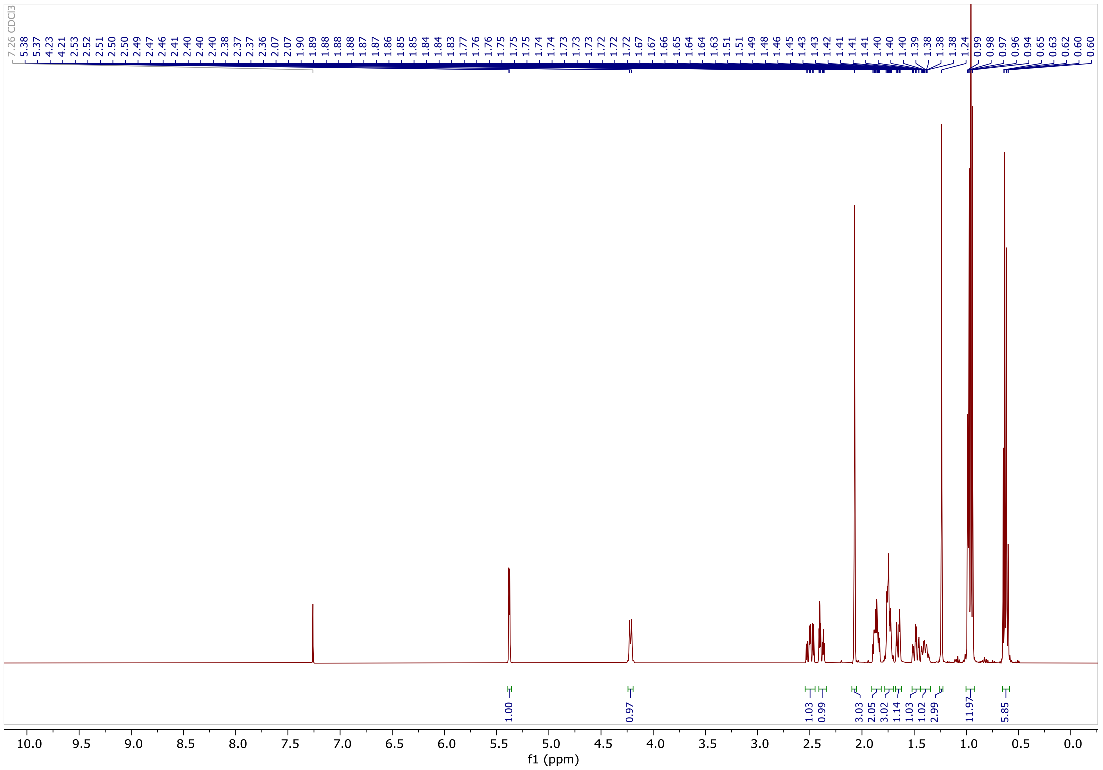

^{13}**C-NMR** (126 MHz, $CDCl_3$) spectra of **64**

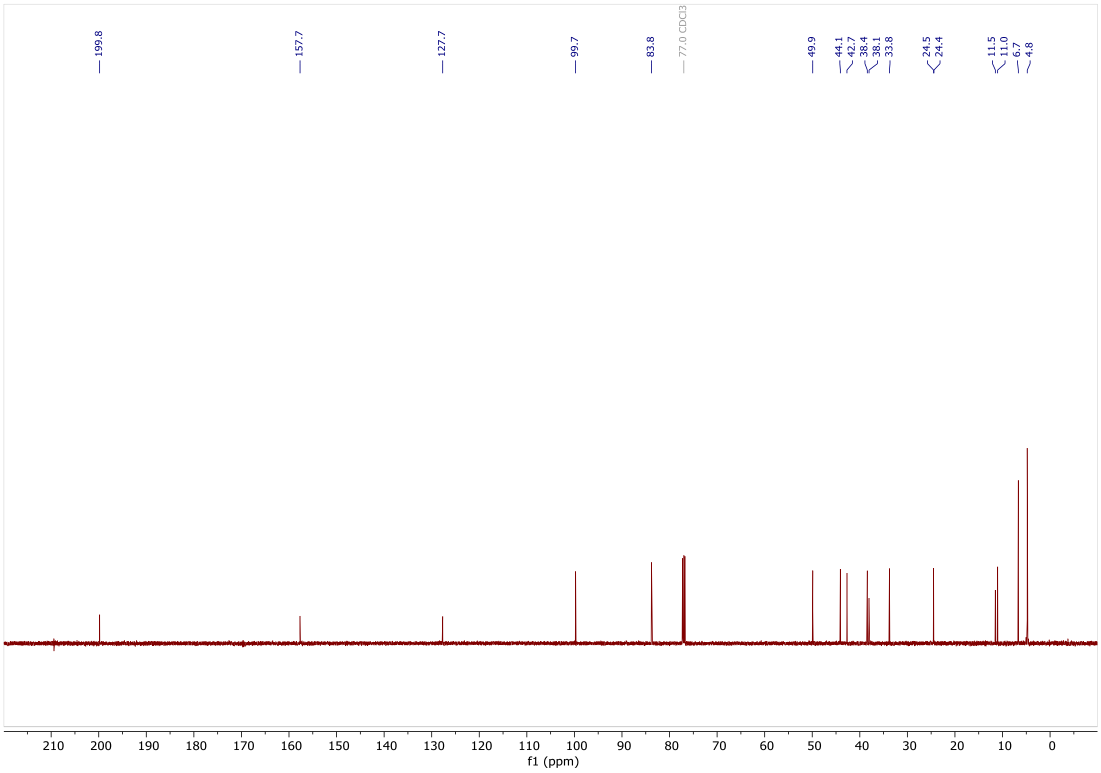

Compound 66: 1,3-bis(methoxy((triethylsilyl)oxy)methyl)bicyclo[1.1.1]pentane (See compound data)

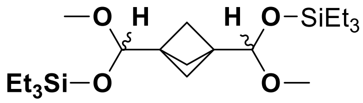

66

^{1}H-NMR (500 MHz, $CDCl_3$) spectra of **66**

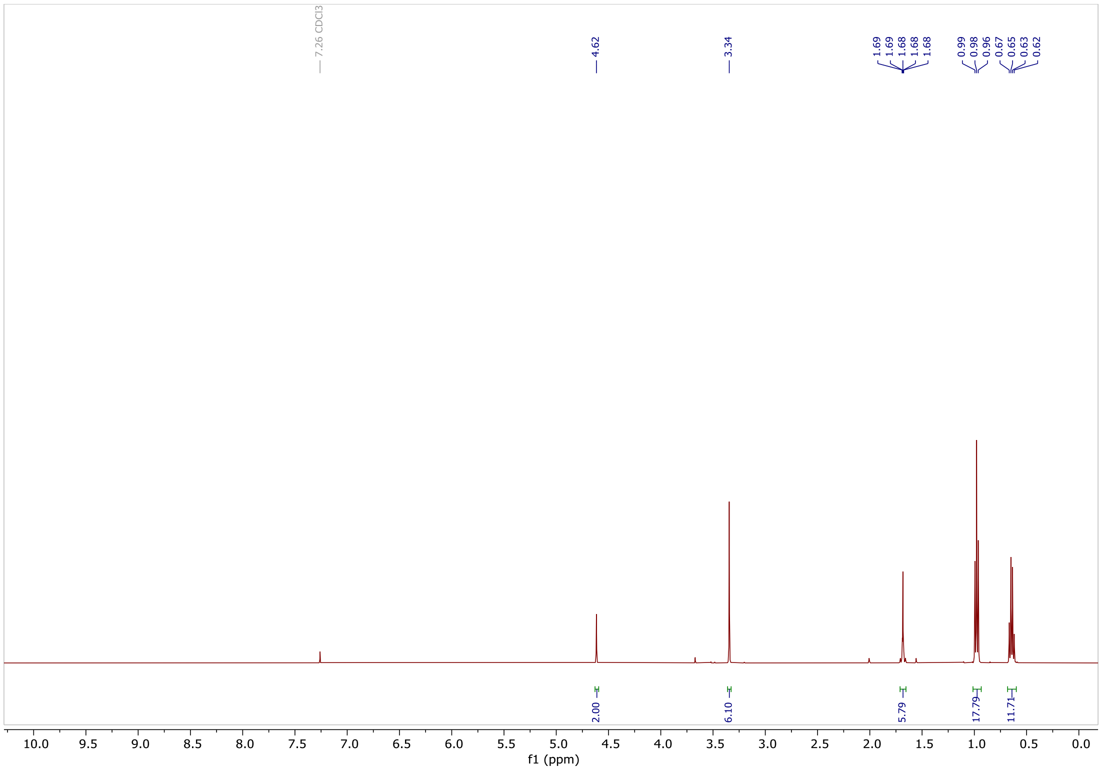

^{13}C-NMR (126 MHz, $CDCl_3$) spectra of **66**

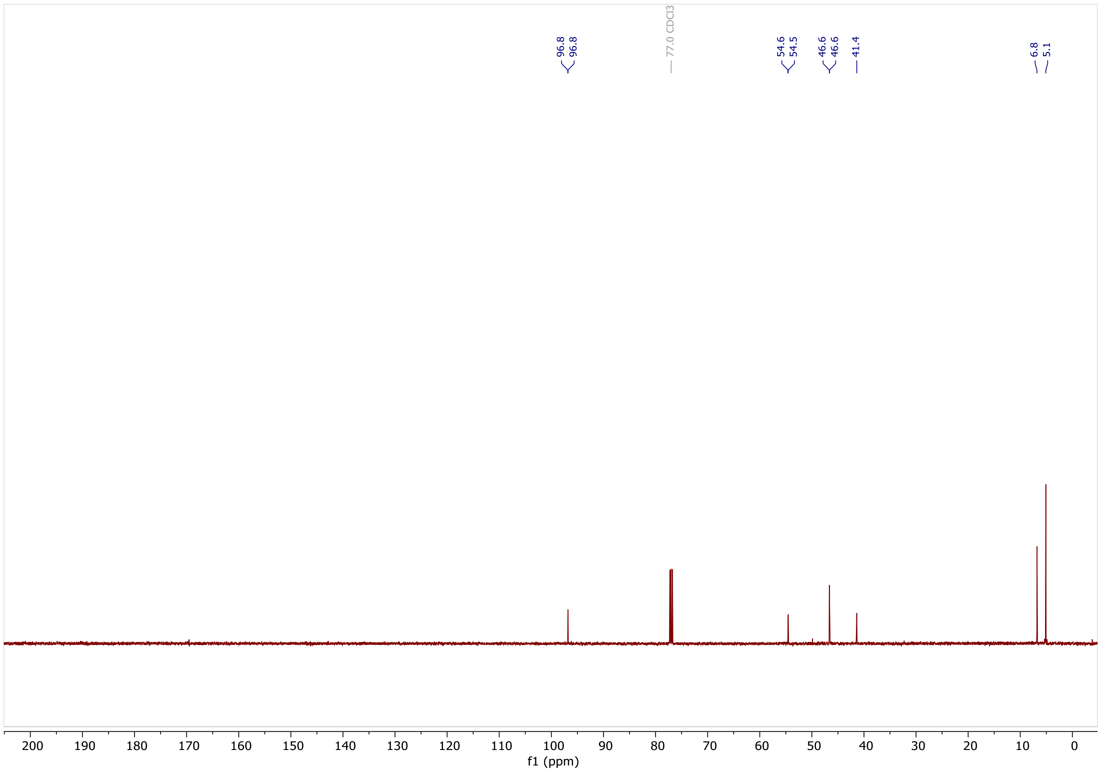

Compound 67: 1,4-bis(methoxy((triethylsilyl)oxy)methyl)cubane (See compound data)

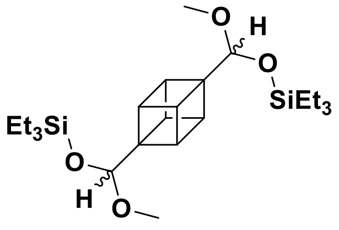

67

^{1}H-NMR (500 MHz, $CDCl_3$) spectra of **67**

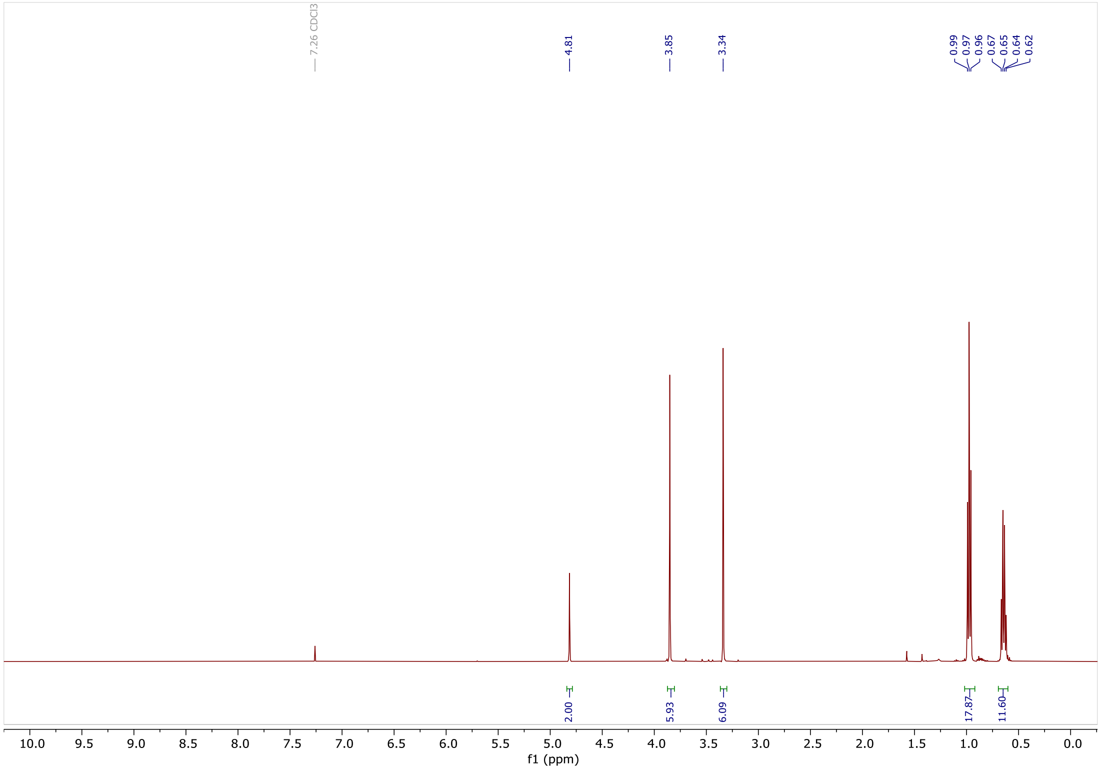

^{13}C-NMR (126 MHz, $CDCl_3$) spectra of **67**

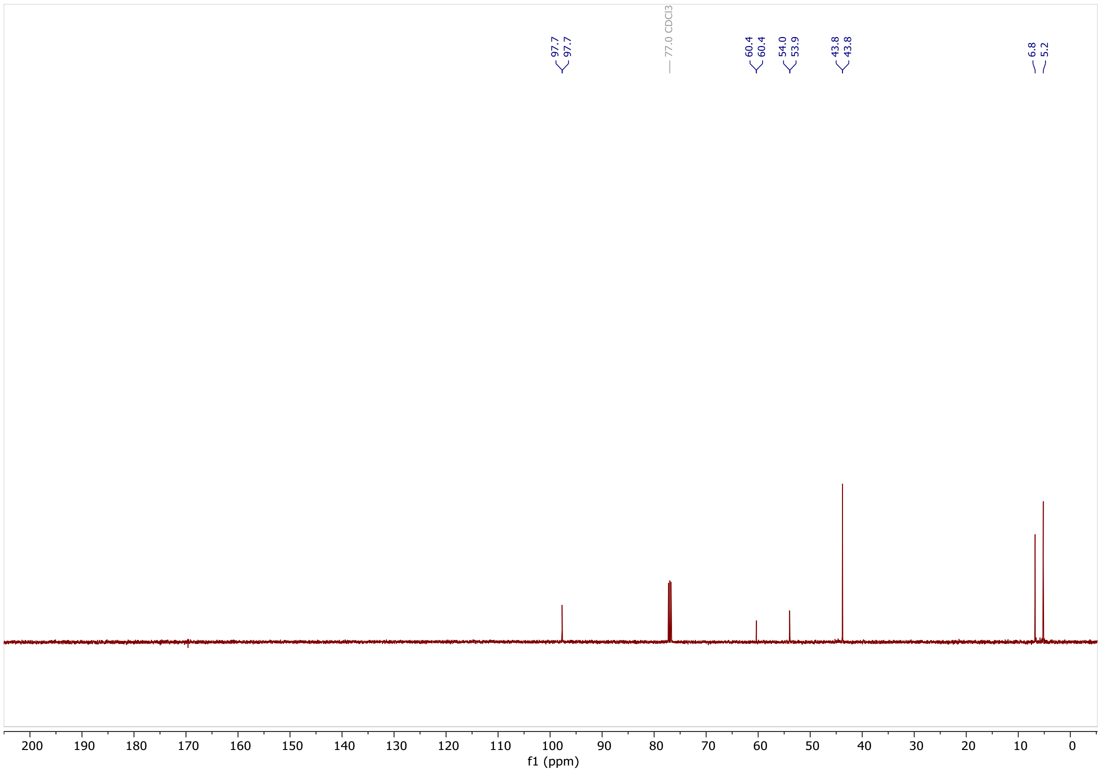

Compound S24: ethyl 4-bromo-2-methoxybutanoate (See compound data)

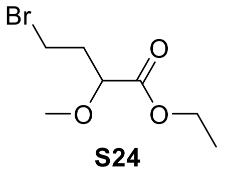

S24

^{1}H-NMR (500 MHz, $CDCl_3$) spectra of **S24**

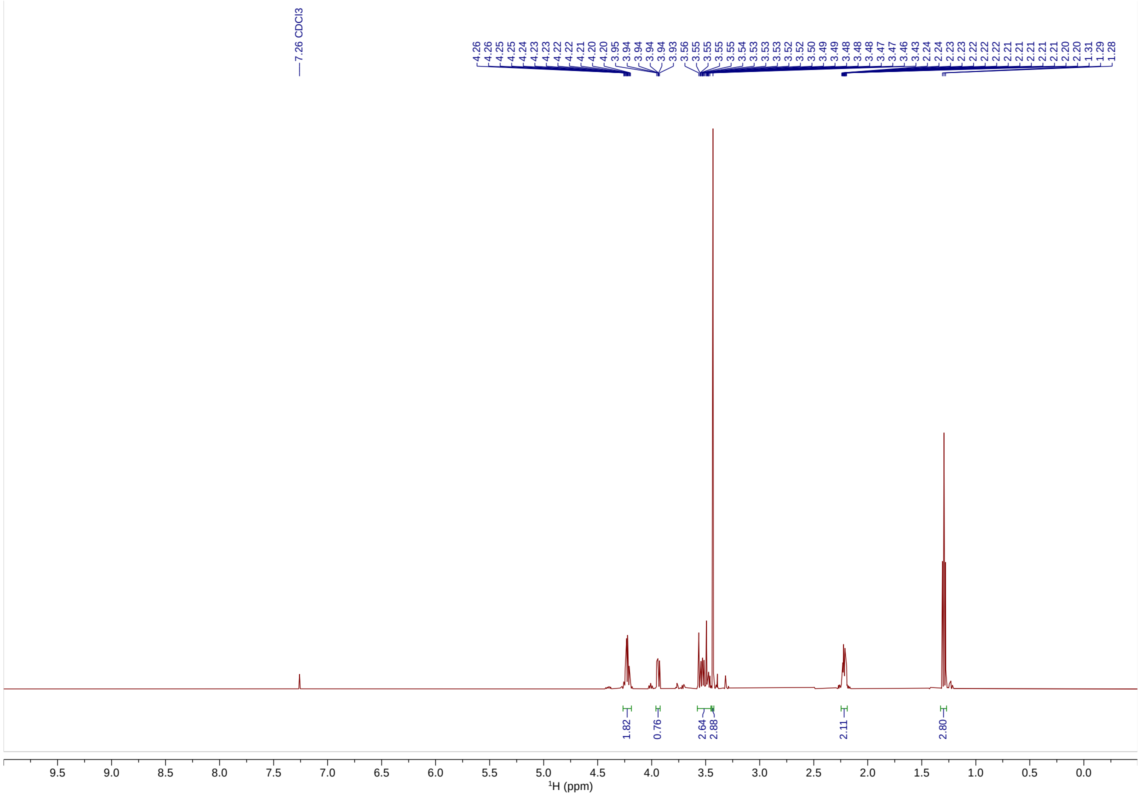

^{13}C-NMR (126 MHz, $CDCl_3$) spectra of **S24**

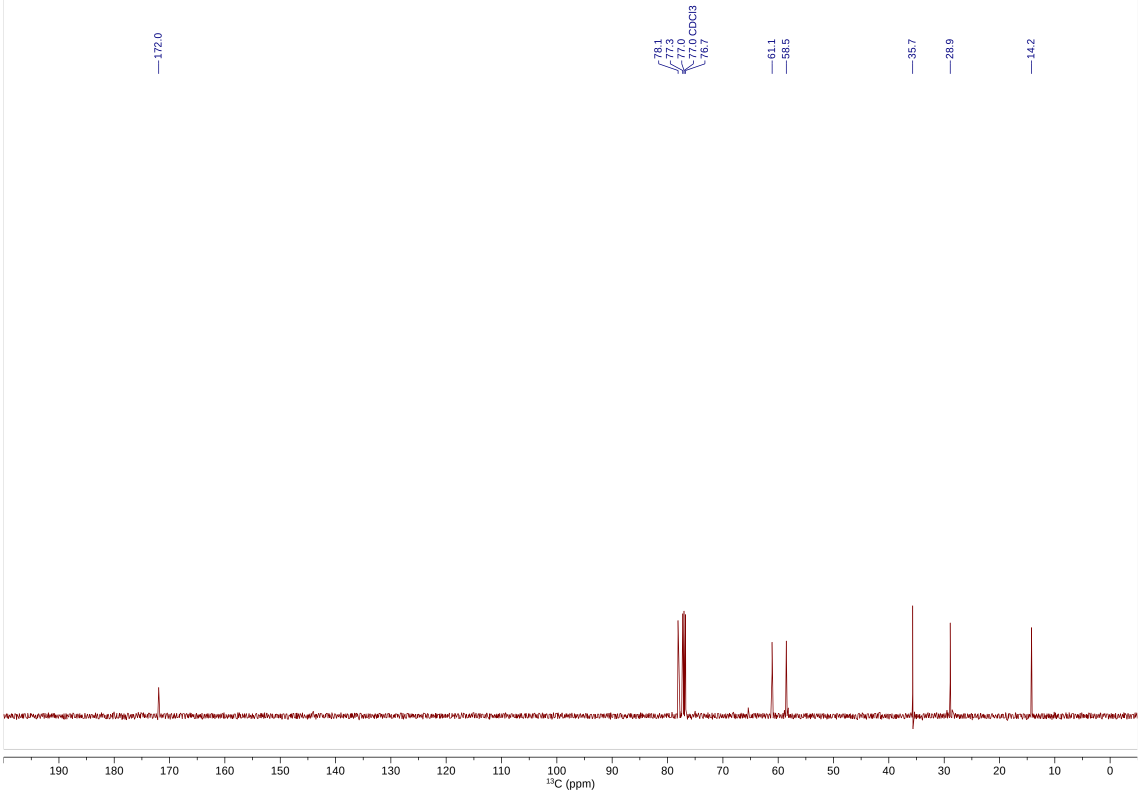

Compound S25: ethyl 4-(2,4,6-trichlorobenzamido)butanoate (See compound data)

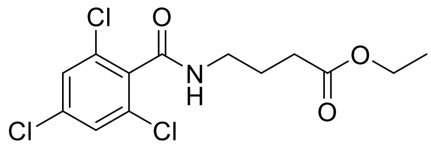

S25

^{1}H-NMR (500 MHz, $CDCl_3$) spectra of **S25**

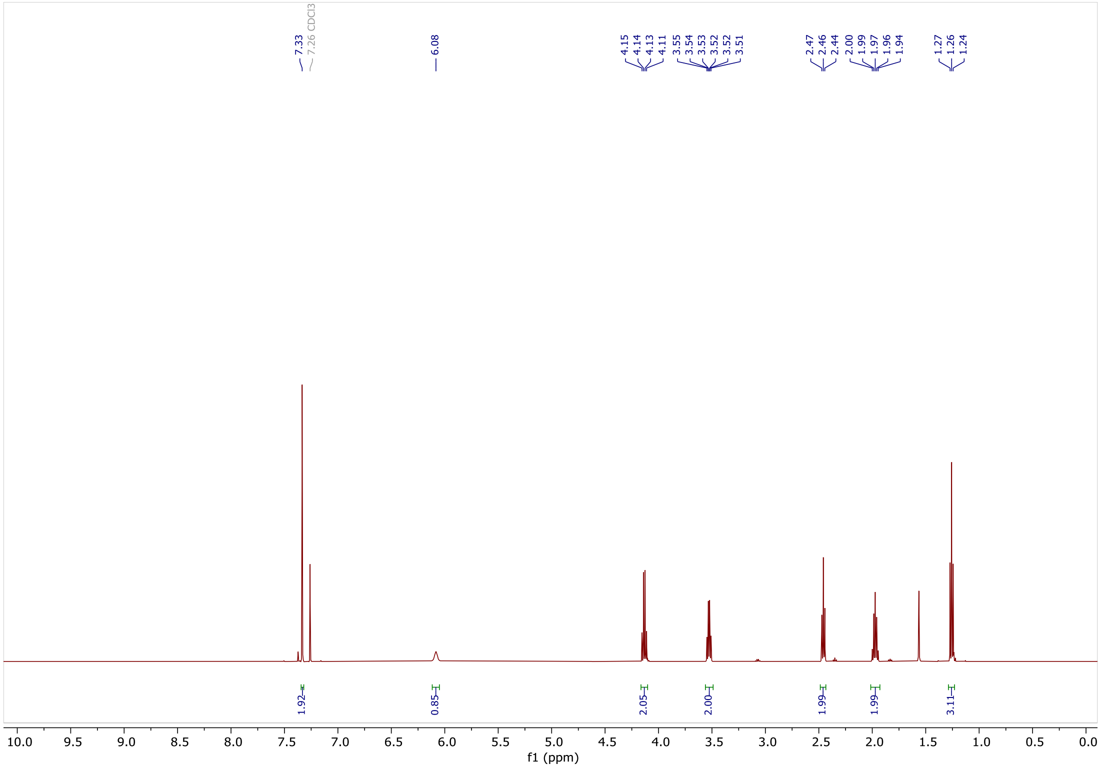

^{13}**C-NMR** (126 MHz, $CDCl_3$) spectra of **S25**

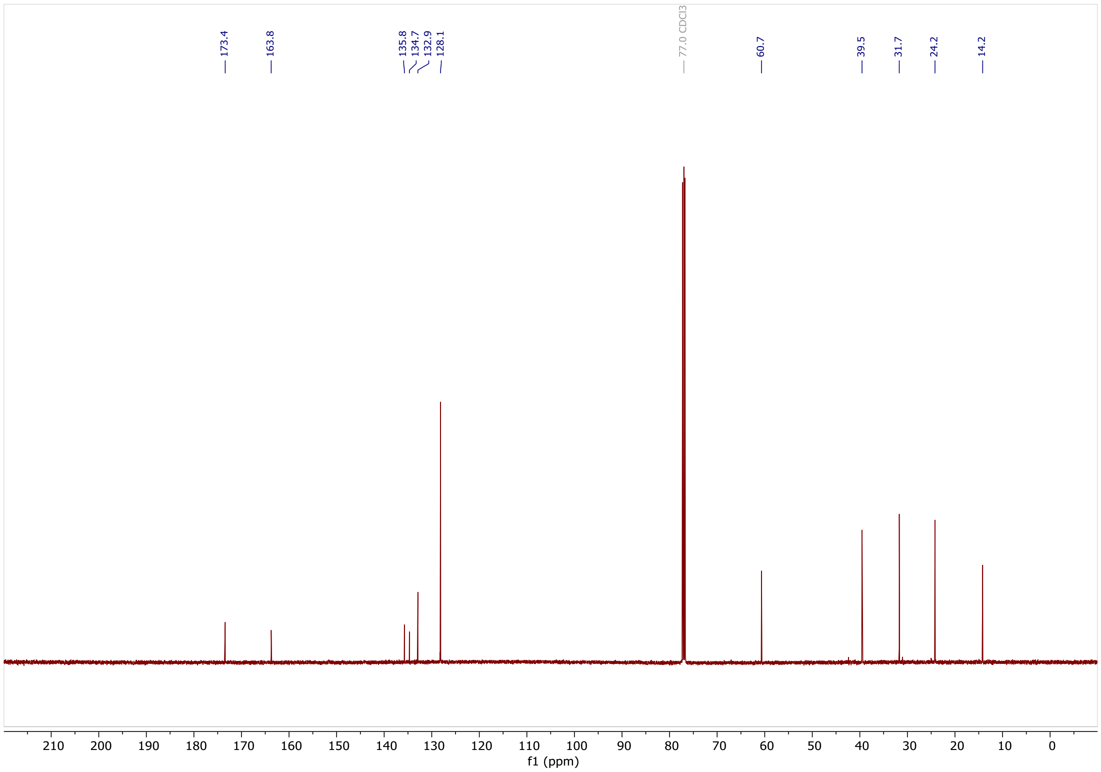

Compound S26: ethyl 4-(4,5,6,7-tetrachloro-1,3-dioxoisoindolin-2-yl)butanoate (See compound data)

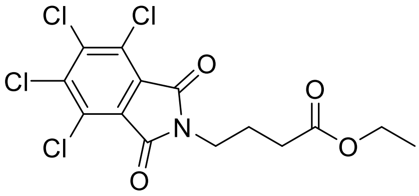

S26

^{1}H-NMR (500 MHz, $CDCl_3$) spectra of **S26**

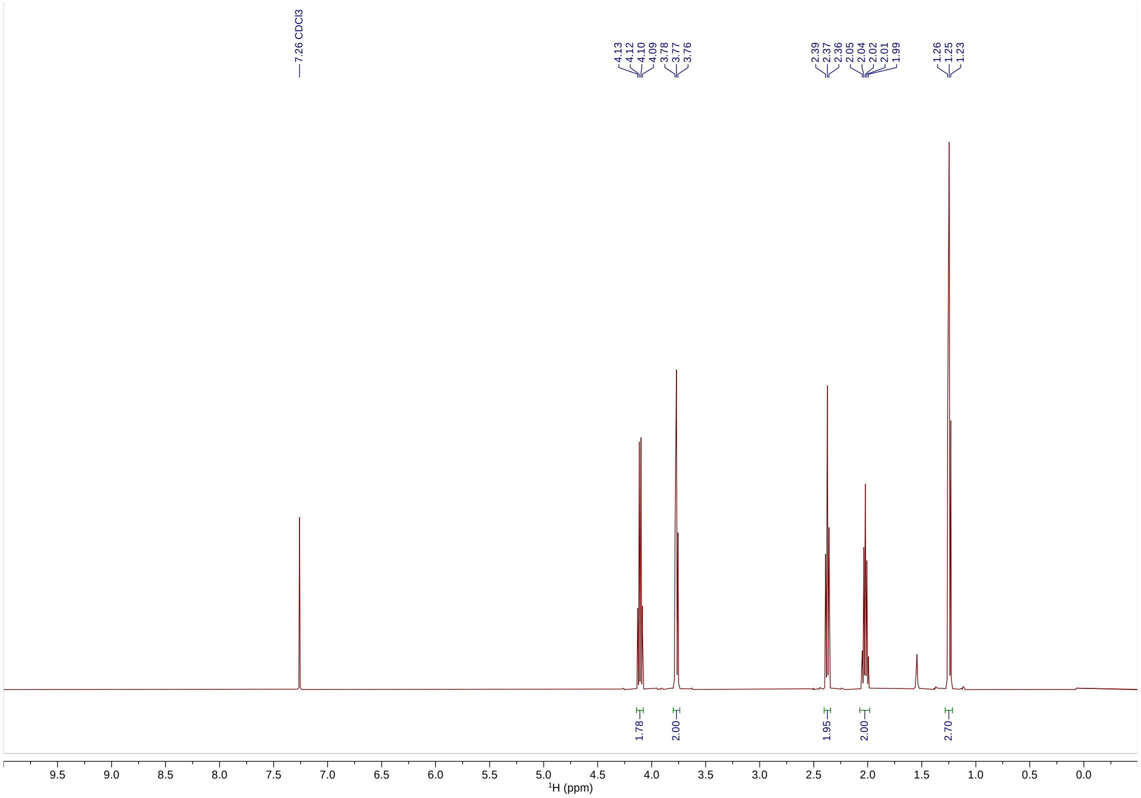

^{13}**C-NMR** (126 MHz, $CDCl_3$) spectra of **S26**

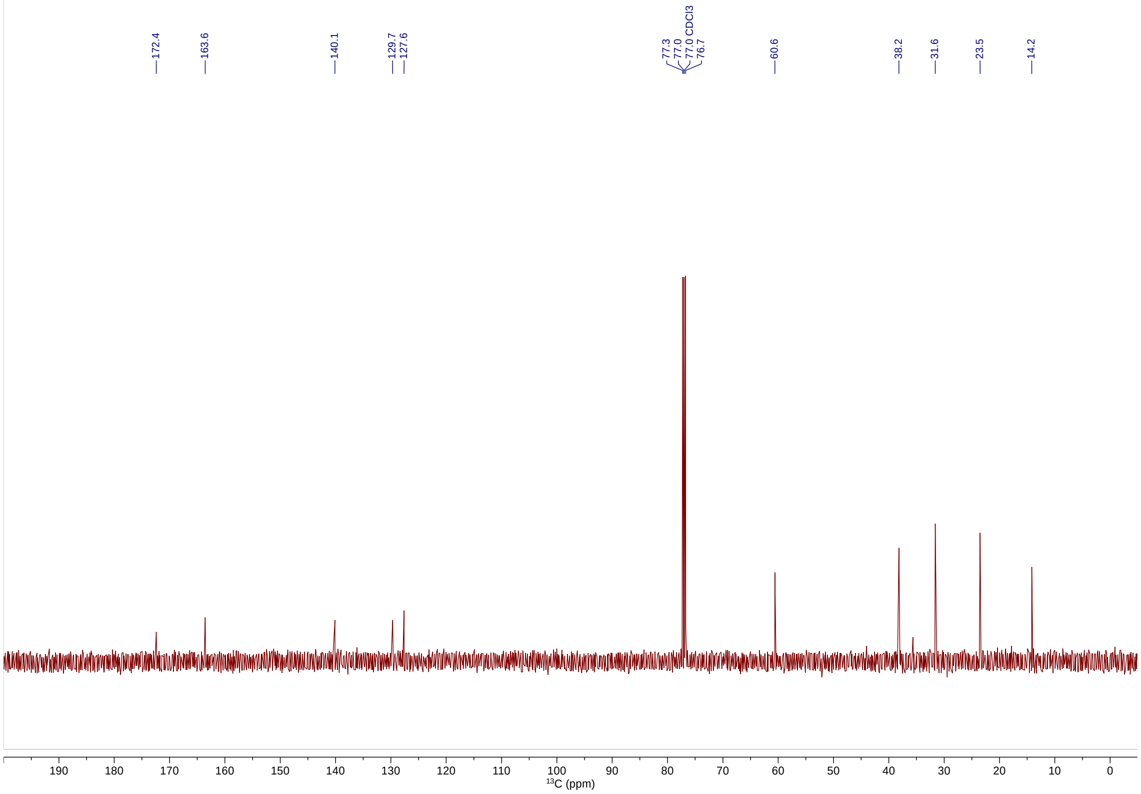

Compound 70: triethyl((1-methoxyoctyl)oxy)silane (See compound data)

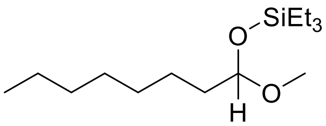

70

[1]**H-NMR** (500 MHz, $CDCl_3$) spectra of **70**

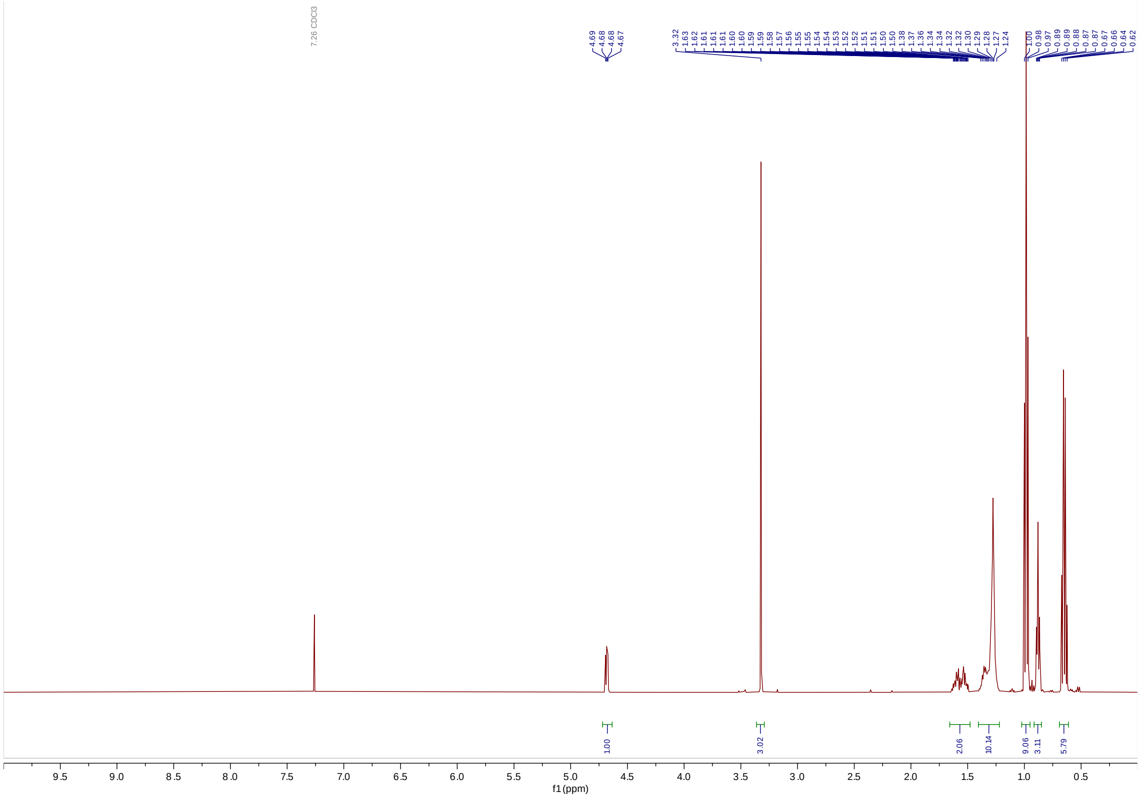

^{13}**C-NMR** (126 MHz, $CDCl_3$) spectra of **70**

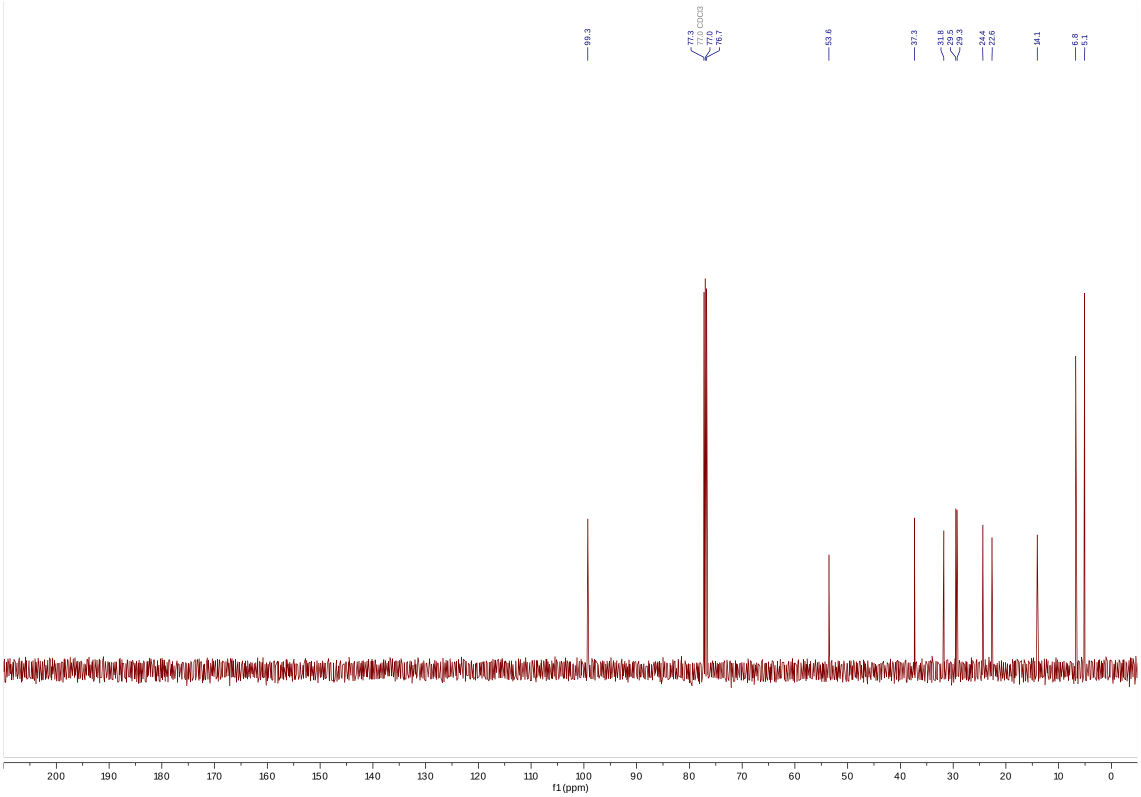

Compound 74: ((2*S*)-3-(benzyloxy)-1-methoxy-2-methylpropoxy)triethylsilane (See compound data)

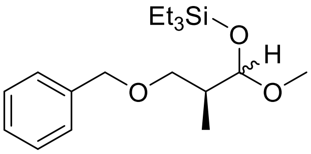

74

^{1}H-NMR (500 MHz, $CDCl_3$) spectra of **74**

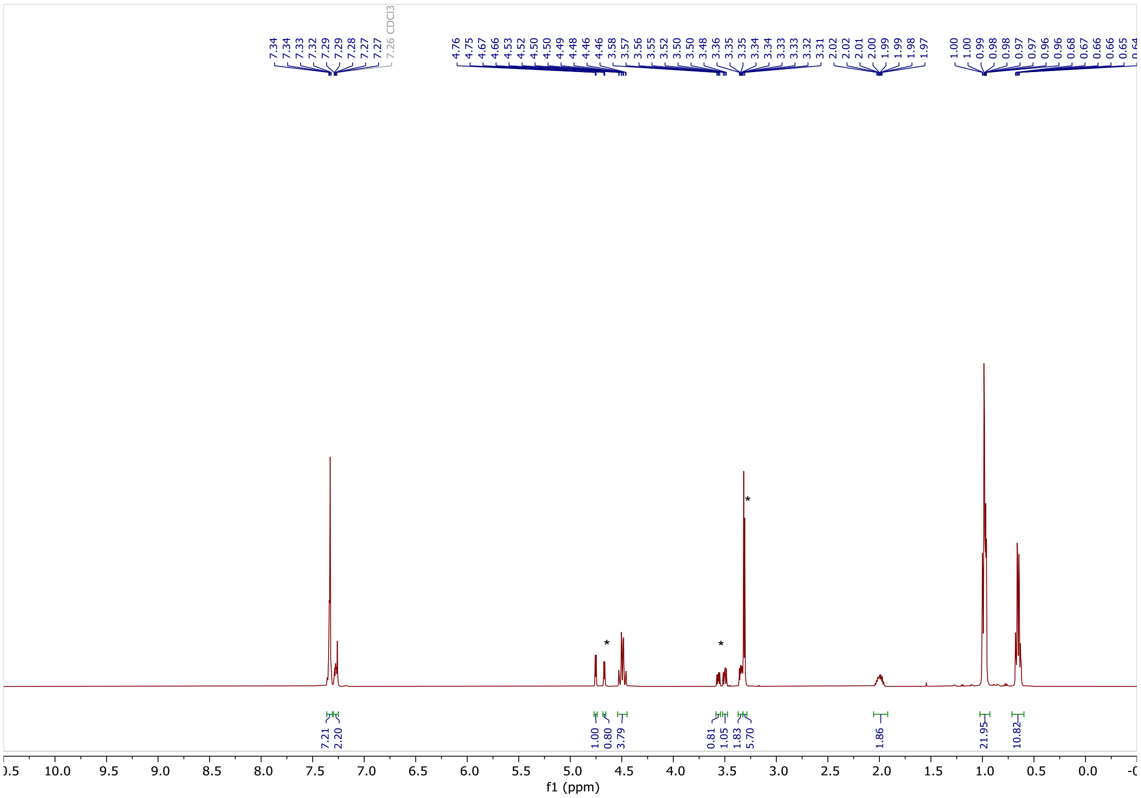

^{13}C-NMR (126 MHz, $CDCl_3$) spectra of **74**

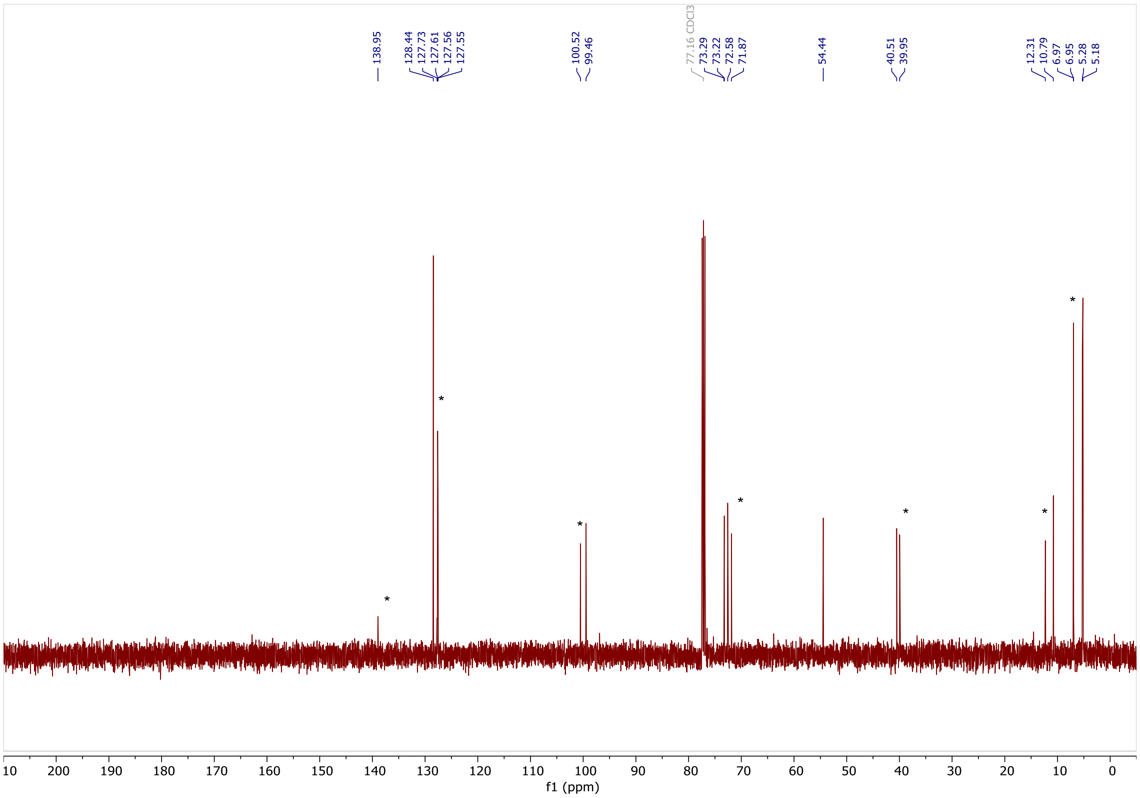

Compound S27: ((2*R*)-3-(benzyloxy)-1-methoxy-2-methylpropoxy)triethylsilane (See compound data)

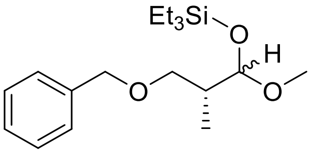

S27

^{1}H-NMR (500 MHz, $CDCl_3$) spectra of **S27**

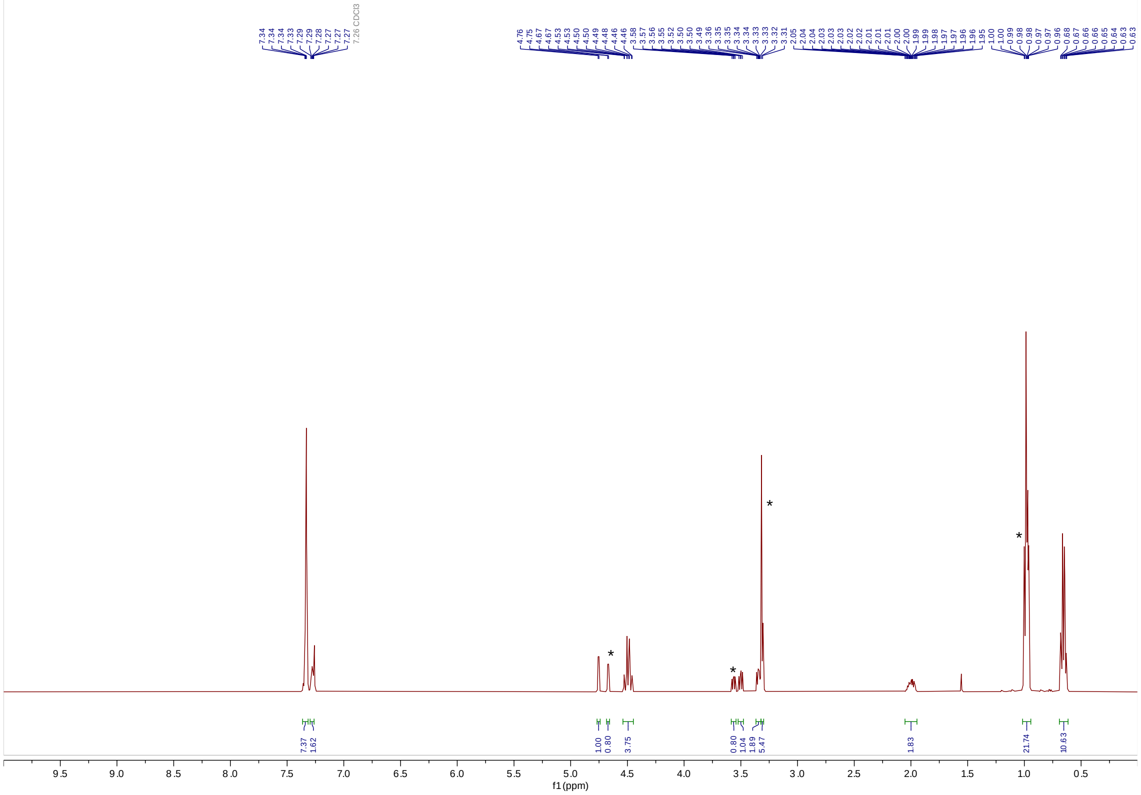

^{13}**C-NMR** (126 MHz, $CDCl_3$) spectra of **S27**

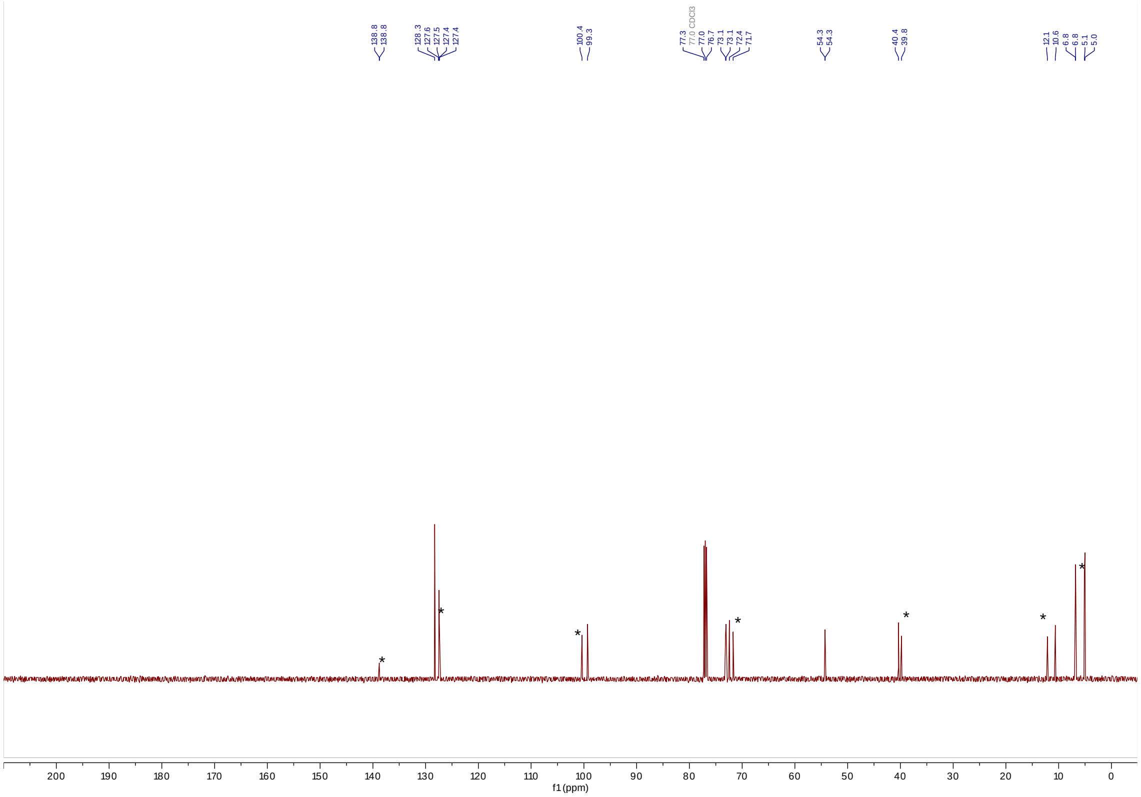

Compound 77: 6-ethoxy-6-oxohexyl 2-hexyldecanoate (See compound data)

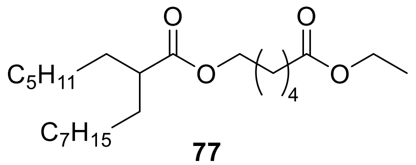

77

^{1}H-NMR (500 MHz, $CDCl_3$) spectra of **77**

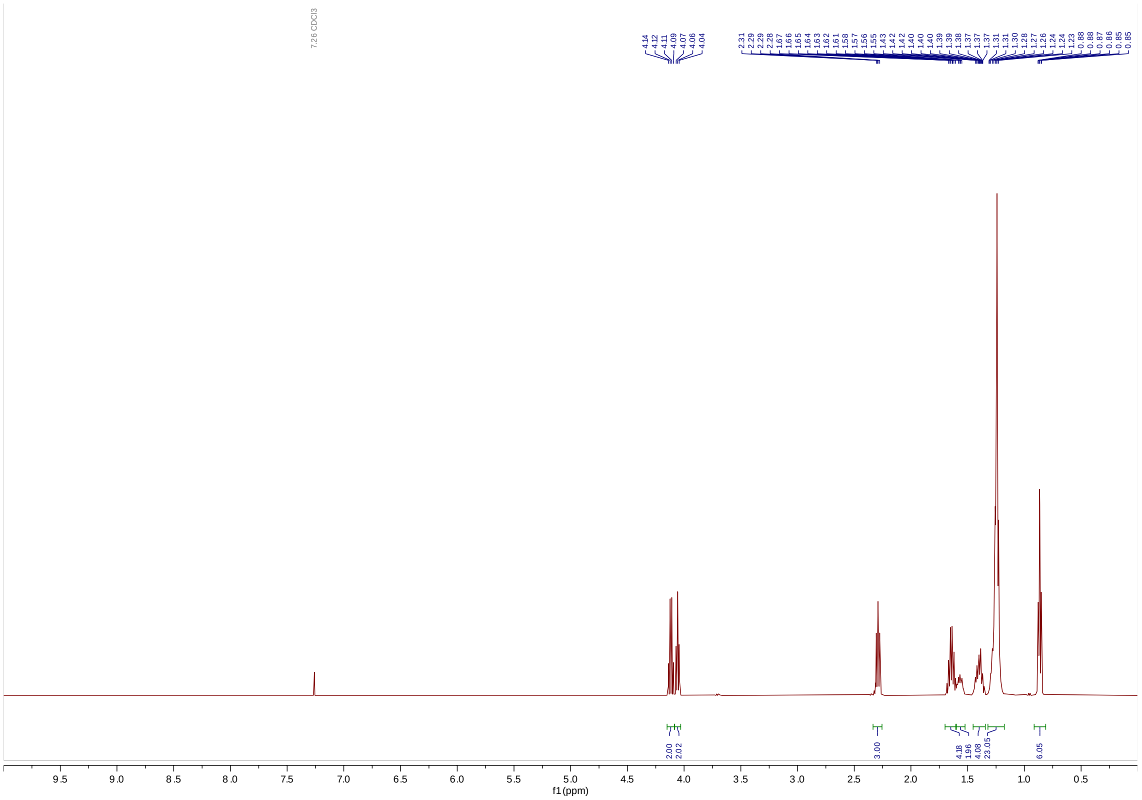

^{13}C-NMR (126 MHz, $CDCl_3$) spectra of **77**

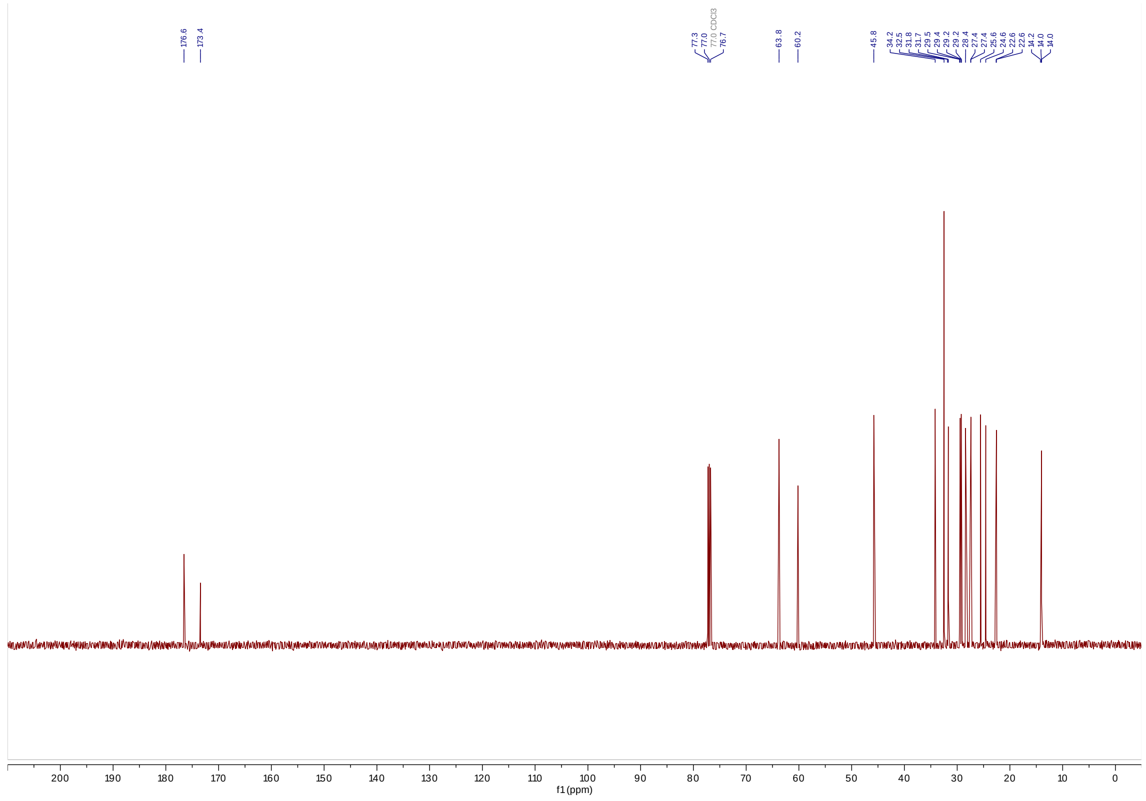

Compound 80: 6-ethoxy-6-((triethylsilyl)oxy)hexyl 2-hexyldecanoate (See compound data)

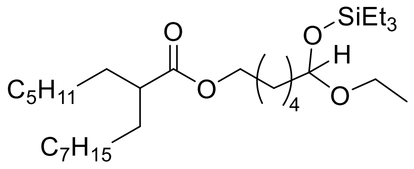

80

^{1}H-NMR (500 MHz, $CDCl_3$) spectra of **80**

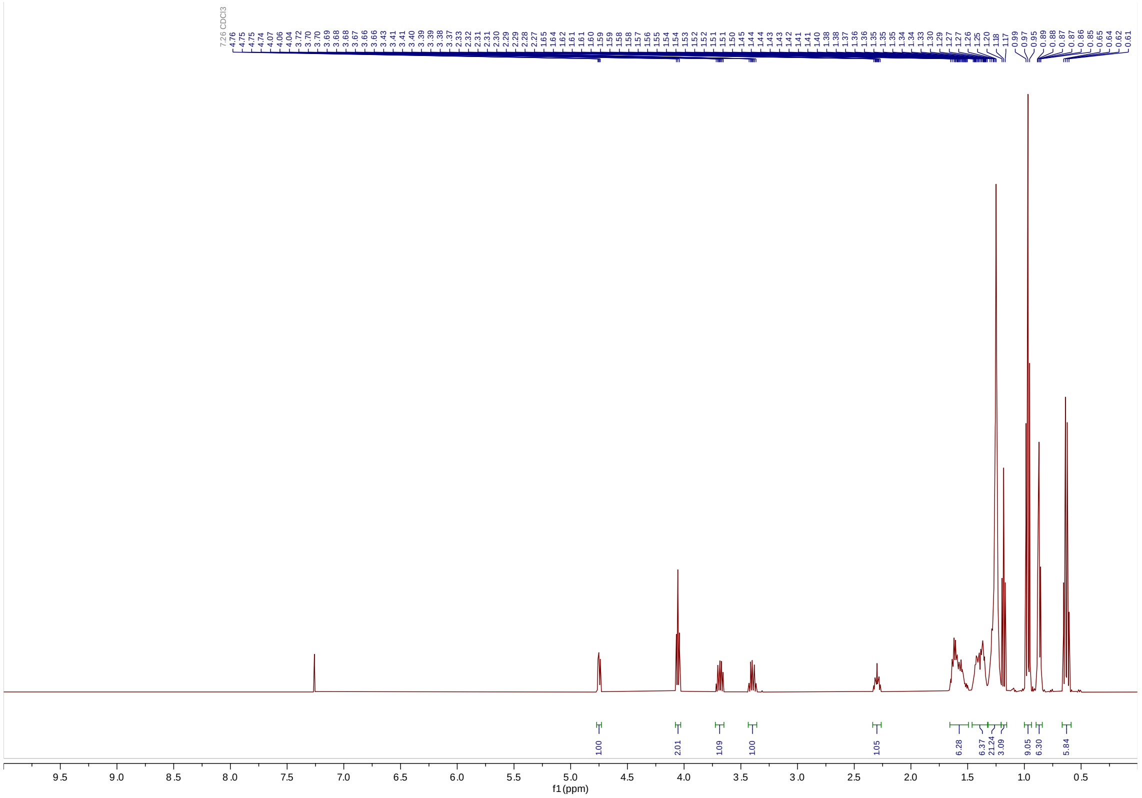

^{13}C-NMR (126 MHz, $CDCl_3$) spectra of **80**

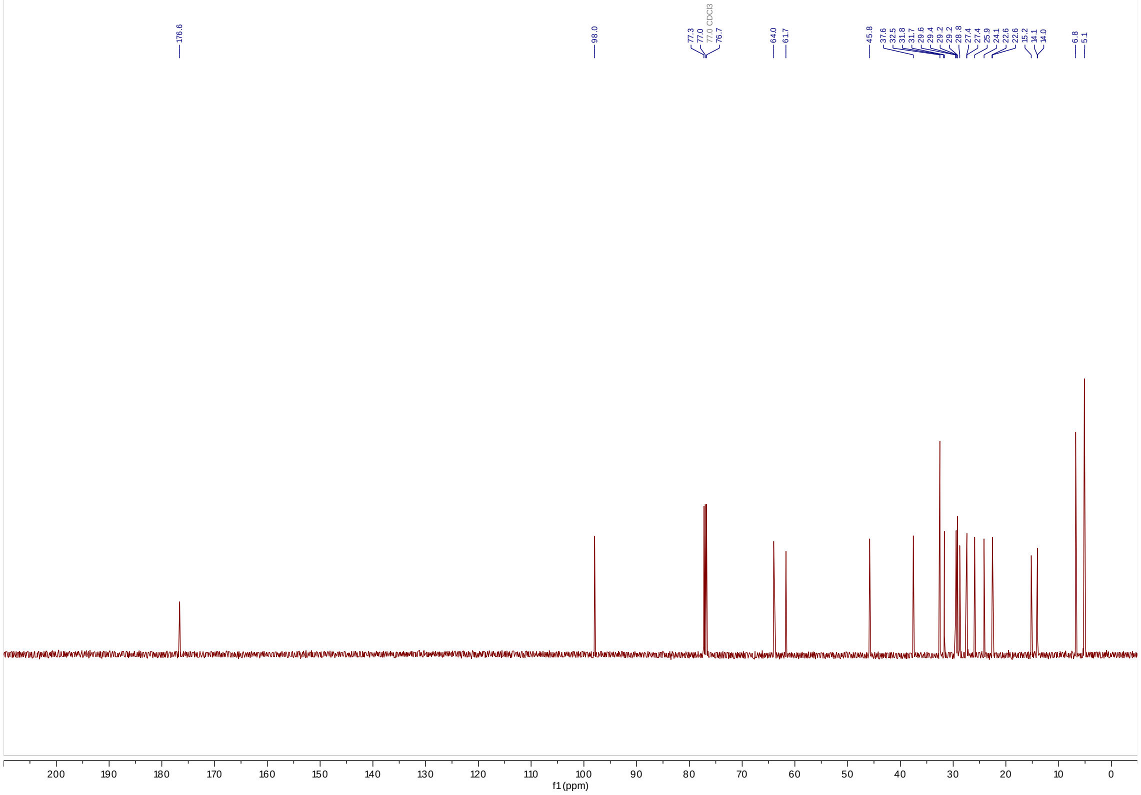

Compound 76: 6-oxohexyl 2-hexyldecanoate (See compound data)

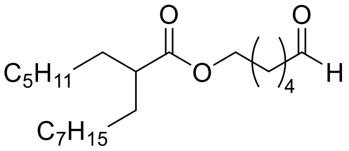

76

^{1}H-NMR (500 MHz, $CDCl_3$) spectra of **76**

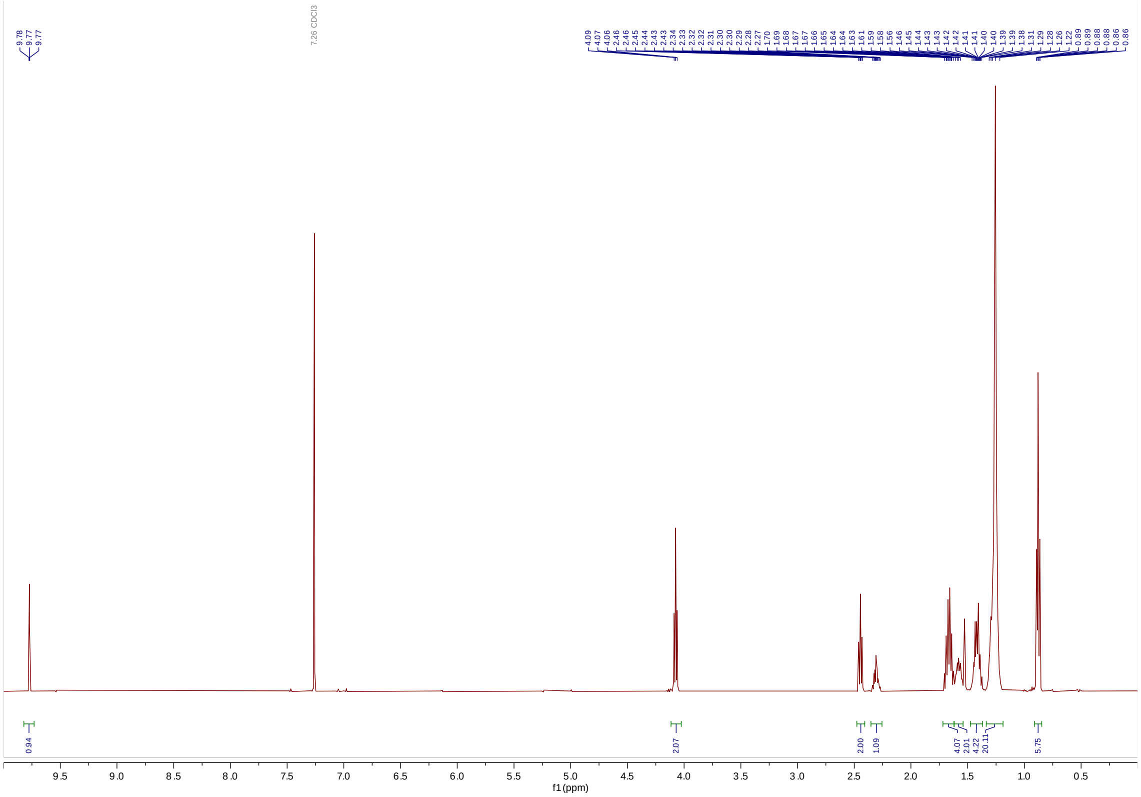

^{13}**C-NMR** (126 MHz, $CDCl_3$) spectra of **76**

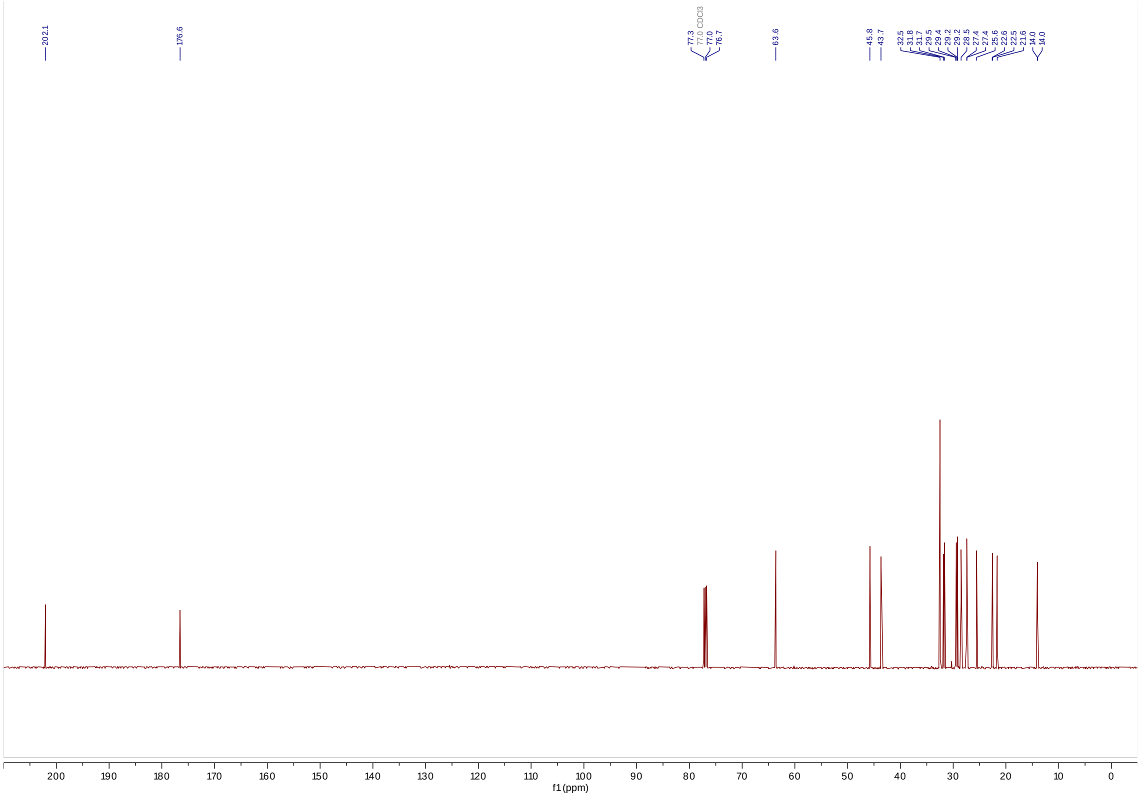

Compound 75: ALC-0315 (See compound data)

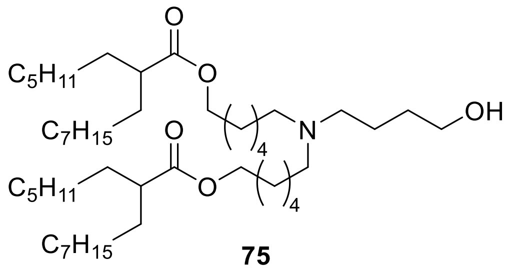

75

^{1}H-NMR (500 MHz, $CDCl_3$) spectra of **75**

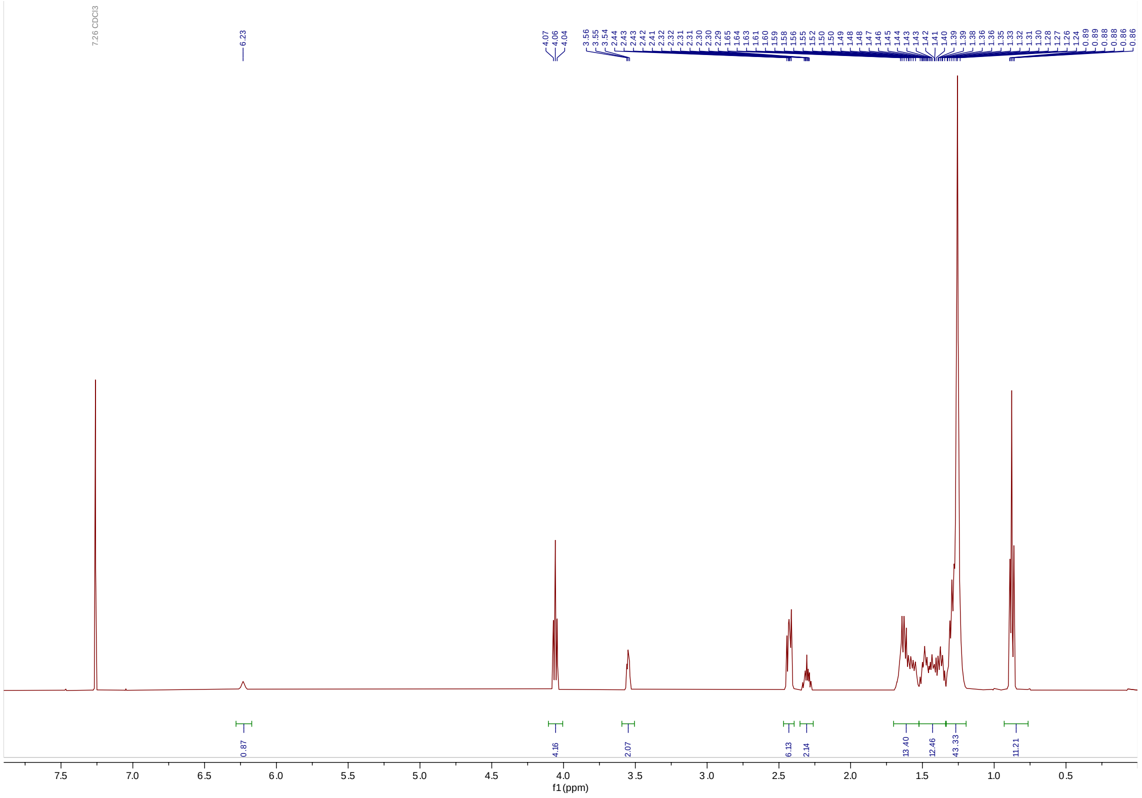

^{13}C-NMR (126 MHz, $CDCl_3$) spectra of **75**

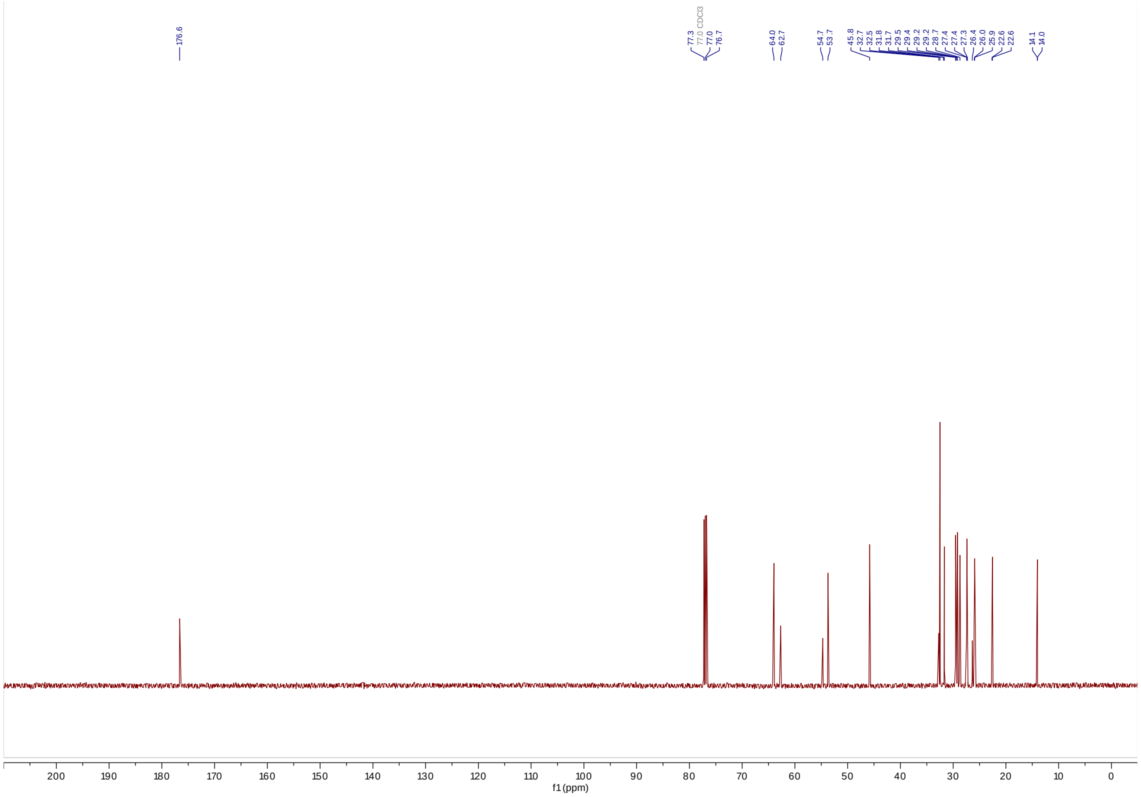

Compound S30: 3-methoxy-3-((triethylsilyl)oxy)propan-1-amine (See compound data)

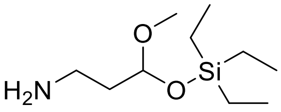

S30

^{1}H-NMR (500 MHz, $CDCl_3$) spectra of **S30**

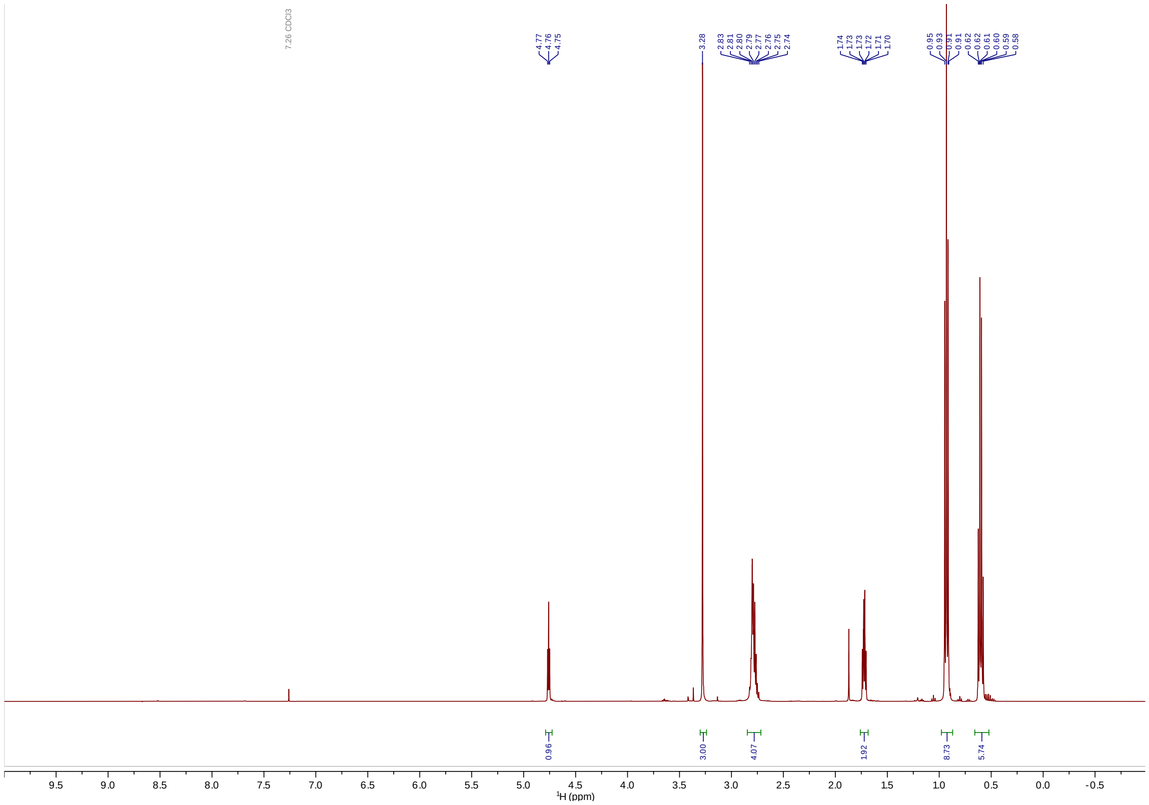

^{13}**C-NMR** (126 MHz, $CDCl_3$) spectra of **S30**

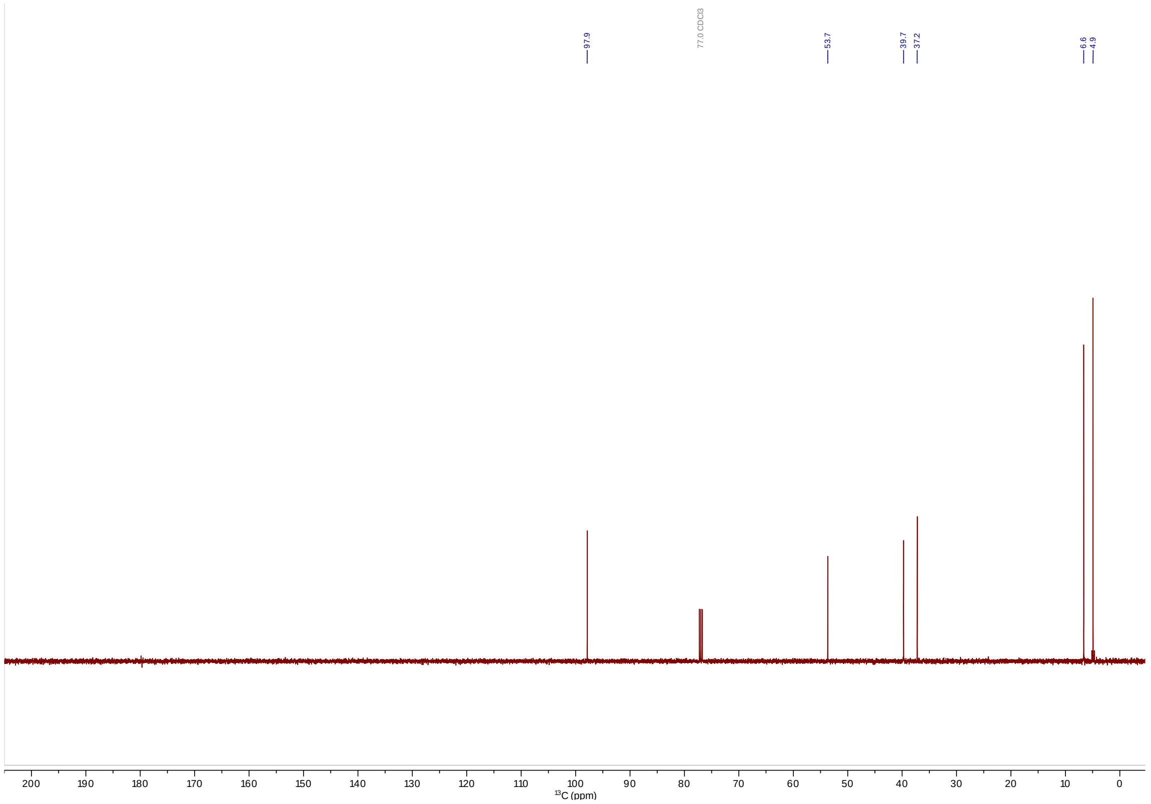

Compound S31: *N*-(3-methoxy-3-((triethylsilyl)oxy)propyl)acetamide (See compound data)

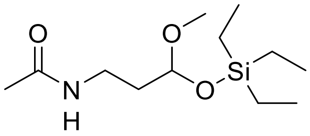

S31

^{1}H-NMR (500 MHz, $CDCl_3$) spectra of **S31**

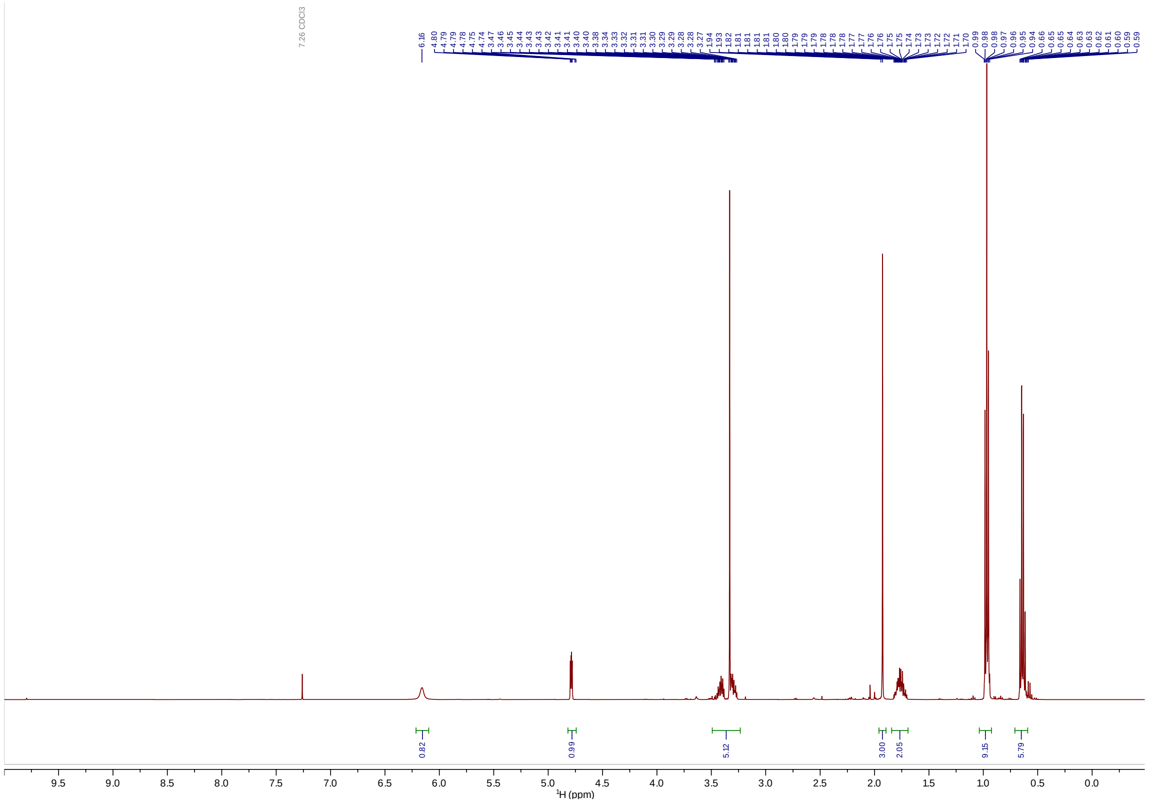

^{13}C-NMR (126 MHz, $CDCl_3$) spectra of **S31**

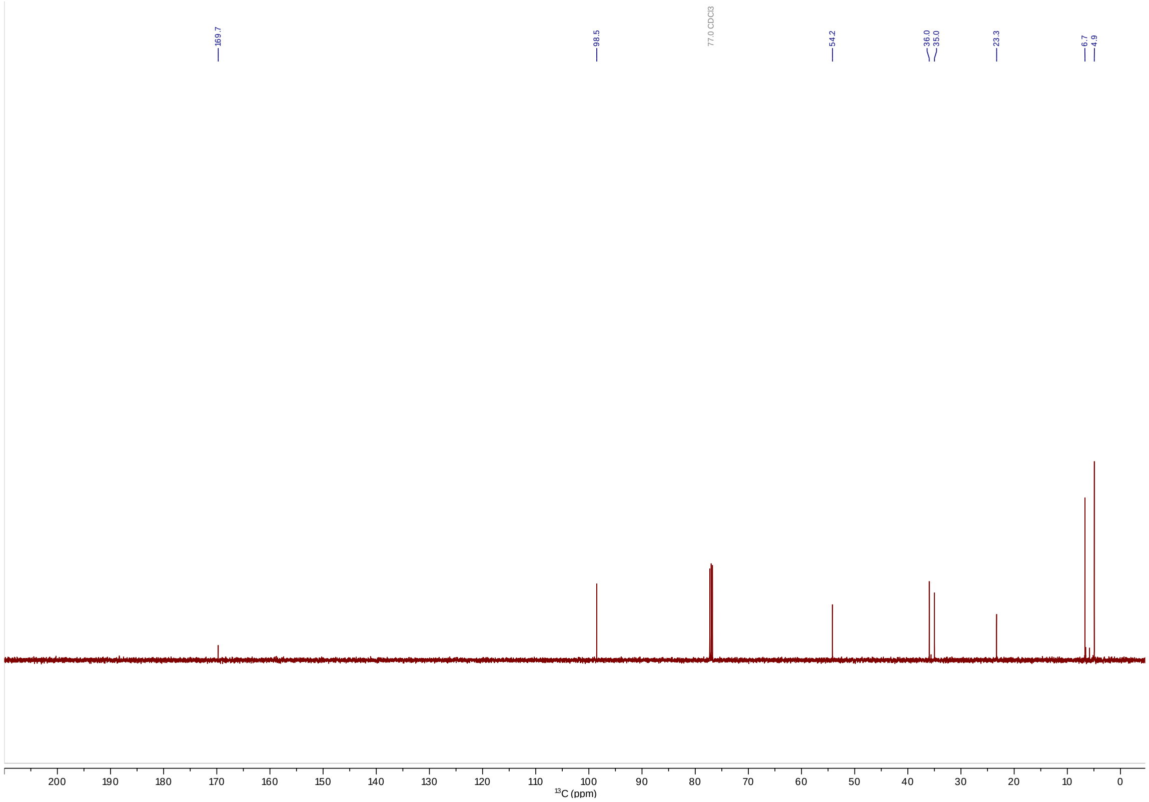

Supplement: Supplementary file 1 — ja4c14596_si_001.pdf [file ja4c14596_si_001.pdf]
